# Network pharmacology, molecular docking, and dynamics analyses to predict the antiviral activity of ginger constituents against coronavirus infection

**DOI:** 10.1038/s41598-024-60721-3

**Published:** 2024-05-27

**Authors:** Asmaa Samy, Afnan Hassan, Nesrine M. Hegazi, Mai Farid, Moustafa Elshafei

**Affiliations:** 1 Zewail City of Science and Technology, Giza, 12578 Egypt; 2 Biomedical Sciences Program, Zewail City of Science and Technology, Giza, 12578 Egypt; 3 Department of Phytochemistry and Plant Systematics, Pharmaceutical and Drug Industries Research Institute, National Research Centre, Cairo, 12622 Egypt

**Keywords:** COVID-19, SARS-CoV-2, Ginger, Network analysis, Molecular docking simulations, Molecular dynamics simulations, Infectious diseases, Computational chemistry

## Abstract

COVID-19 is a global pandemic that caused a dramatic loss of human life worldwide, leading to accelerated research for antiviral drug discovery. Herbal medicine is one of the most commonly used alternative medicine for the prevention and treatment of many conditions including respiratory system diseases. In this study, a computational pipeline was employed, including network pharmacology, molecular docking simulations, and molecular dynamics simulations, to analyze the common phytochemicals of ginger rhizomes and identify candidate constituents as viral inhibitors. Furthermore, experimental assays were performed to analyze the volatile and non-volatile compounds of ginger and to assess the antiviral activity of ginger oil and hydroalcoholic extract. Network pharmacology analysis showed that ginger compounds target human genes that are involved in related cellular processes to the viral infection. Docking analysis highlighted five pungent compounds and zingiberenol as potential inhibitors for the main protease (M^pro^), spike receptor-binding domain (RBD), and human angiotensin-converting enzyme 2 (ACE2). Then, (6)-gingerdiacetate was selected for molecular dynamics (MD) simulations as it exhibited the best binding interactions and free energies over the three target proteins. Trajectories analysis of the three complexes showed that RBD and ACE2 complexes with the ligand preserved similar patterns of root mean square deviation (RMSD) and radius of gyration (Rg) values to their respective native structures. Finally, experimental validation of the ginger hydroalcoholic extract confirmed the existence of (6)-gingerdiacetate and revealed the strong antiviral activity of the hydroalcoholic extract with IC_50_ of 2.727 $\upmu \hbox {g}/\hbox {ml}$. Our study provides insights into the potential antiviral activity of (6)-gingerdiacetate that may enhance the host immune response and block RBD binding to ACE2, thereby, inhibiting SARS-CoV-2 infection.

## Background

Coronavirus disease 2019 (COVID-19) is caused by Severe acute respiratory syndrome coronavirus 2 (SARS-CoV-2) originated in Wuhan, Hubei, China. The first case diagnosed with COVID-19 was reported on December 1, 2019, while unpublished reports showed that the first confirmed case traced back to November 17 according to the South China Morning Post^1^. After infecting the first human case with the virus, the disease spread rapidly among people without any animal carrier. So, the World Health Organization (WHO) characterized COVID-19 as a public health emergency of international concern (PHEIC) on January 30, 2020, and a pandemic on March 11, 2020^2^. Although several COVID-19 vaccines have been developed and widely used, WHO reported more than 700 million confirmed cases of COVID-19 including more than 6 million as of August 2023^3^.

SARS-CoV-2 is a member of Betacoronavirus that includes severe acute respiratory syndrome (SARS) and Middle Eastern respiratory syndrome (MERS) coronaviruses which are associated with fatal diseases as well^4^. They are enveloped viruses with a positive single stranded RNA. They express four structural proteins; Spike (S) protein, envelope (E) protein, membrane (M) protein, and nucleocapsid (N) protein^5^. The life cycle of SARS-CoV-2 consists of three stages; (1) attachment and entry, (2) replication and synthesis, and (3) viral assembly and release. Spike protein is responsible for the first phase using two domains where S1 called receptor binding domain (RBD) binds to host cell’s receptor and S2 is needed for cell membrane fusion. Structure analysis of RBD of SARS-CoV-2 shows high similarity to SARS-CoV RBD which proposes that SARS-CoV-2 may use human angiotensin-converting enzyme 2 (ACE2) for attachment as SARS-CoV does^6^. Studies suggest that blocking the interaction between ACE2 and spike protein can suppress the virus entry into the host cell and prevent the development of the disease. Importantly, studies showed that ACE2 catalytic activity is independent of SARS-CoV binding, meaning that it is possible to develop drugs that selectively bind to ACE2 domain used for viral attachment without affecting normal functions in the cell^7^. The genome of coronaviruses consists of around 30, 000 nucleotides that encode two polyproteins named; pp1a and pp1ab^8^, representing one of the longest genomes of viruses. They are cleaved by two proteases; papine-like protease (PL^pro^) and main protease (M^pro^). Both proteases are necessary for the virus’ survival in the host cell as they cleave the polyprotein to make RNA of structural and non-structural proteins^9,10^. It was found that M^pro^ is conserved among other Betacoronavirus (SARS-CoV and MERS)^11^. Therefore, the identification of protease inhibitors is a promising approach against coronavirus infection.

**Figure 1:**
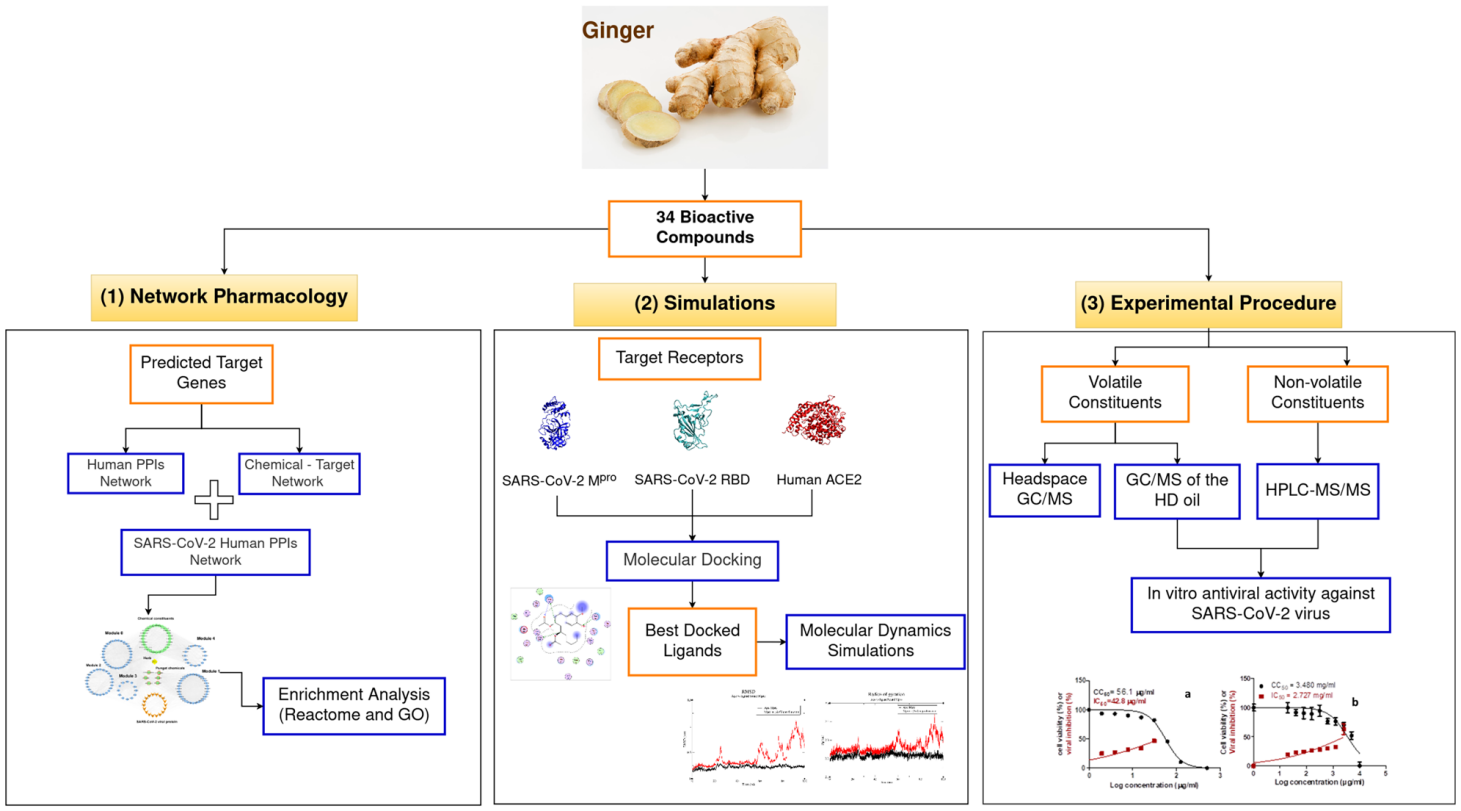
A block diagram represents the workflow of integrated network pharmacology, molecular docking, and dynamics analyses approach, followed by experimental validation to validate the antiviral activity of ginger bioactive compounds.

Accelerating anti-coronavirus drug development is urgently needed as the virus is rapidly evolved in many variants of concern such as Alpha, Beta, Gamma, Delta, and Omicron that may have increased viral infectivity, increased pathogenicity, different clinical symptoms, or higher resistance to vaccines and treatments^12^. Since antiquity, natural products and their derivatives have been used in traditional and ancient medicine to treat various diseases and infections. To date, more than 65% of the global population prefers using nature-derived medicines as the first line of treatment for many diseases, owing to their safety and fewer adverse effects^13,14^. Nature has provided a treasure trove of small molecules for drug discovery and development, especially for infectious diseases. So far, many phytochemicals have proved potential antibacterial and antiviral activities^15^. Recently, there has been a significant renaissance of interest in the natural product discipline for the rediscovery of novel and bioactive scaffolds. The National Health Commission in China has issued protocols for the use of herbal medicine as monotherapy or in combination with Western medicine^16,17^ based on previous experience and significant clinical results for the treatment of SARS-CoV and MERS^18^. One of the most commonly natural products used in Chinese medicine is Ginger (*Zingiber officinale*) as it has many effects including anticancer^19,20^, anticoagulant^21^, anti-inflammatory^22^, antioxidant^23–25^, antifungal^26^, and antimicrobial^27^. Many studies showed that ginger may help prevent or treat several diseases such as cardiovascular diseases^28^, neurodegenerative diseases^29,30^, and respiratory diseases^31,32^. Experimental studies showed the antiviral activity of ginger compounds against human respiratory syncytial virus (HRSV)^33^ and Chikungunya virus (CHIKV)^34^. Gingerenone A (Gin A) is a derived compound from ginger roots that can inhibit replication of influenza A virus (IAV) including subtypes (H1N1, H5N1, and H9N2)^35^. Additionally, a clinical trial showed that feeding patients with acute respiratory distress syndrome (ARDS) with an enteral diet enriched in ginger may improve oxygenation and decrease the duration of mechanical ventilation and length of stay in intensive care unit^36^. To date, many computational studies have been performed to investigate the potential antiviral activity of different natural products including ginger to help accelerate the drug discovery process against COVID-19. Muhammad et al.^37^ docked 50 derivatives of shogaols against SARS-CoV-2 M^pro^ where 39 of them achieved lower binding energies than chloroquine which was docked as a reference ligand. Gingerols were also docked against different SARS-CoV-2 receptors and showed potential inhibitory effects. For instance, (6)-gingerol can bind to M^pro^, spike protein, and RNA binding protein^38^, and Cathepsin K^39^. Haridas et al.^40^ proposed (6)-gingerol, (8)-gingerol, (10)-gingerol, (10)-shogaol, (8)-paradol, and (10)-paradol as potential inhibitors of viral entry by binding to the spike RBD and human ACE2 as well. Al-Sanea et al.^41^ assessed the anti-SARS-CoV-2 activity of methanolic extract of ginger, ginger silver nanoparticles (AgNPs), strawberry methanolic extract, and strawberry AgNPs using MTT assay and showed promising results for both ginger AgNPs and strawberry methanolic extract. Studies in Saudi Arabia^42^, Bangladesh^43^, Tunis^44^, several countries in Africa^45,46^, and Iran^47^ indicated that COVID-19 patients who consumed ginger alone or combined with other herbs exhibited lower hospitalization rate or reduced clinical symptoms. According to these previous studies, we performed a combined *in silico* approach of network pharmacology, molecular docking, and dynamics analyses to identify the most promising antiviral ginger constituents out of 34 ginger compounds and to help understand the mechanism underlying their potential antiviral activity as summarized in Fig. 1.

## Methods

### Collection of chemical compounds and target genes

Ginger has many constituents, varying from one country to another based on the place of the origin and whether the rhizomes are fresh or dry^48^. An extensive literature survey was conducted to retrieve the common phytochemicals of ginger rhizomes through PubMed^49^ and Google Scholar platforms. Lipinski rule of five (RO5)^50^ was used for absorption, distribution, metabolism, and excretion (ADME) analysis of ginger compounds using Swiss-ADME online server^51^. Only compounds meeting the RO5 criteria were included in the study analysis. For target gene retrieval, Similarity ensemble approach (SEA)^52^, TargetNet^53^, and SwissTragetPrediction^54^ databases were used to collect target genes of the corresponding chemicals. Each database has a different naming format for the output, so all queries were mapped to the HUGO Gene Nomenclature Committee (HGNC) gene symbol^55^. Then, they were pooled to remove any duplicate genes from different resources.

### Construction and analysis of the ginger network

Cytoscape v3.7.1 software^56^ was used to construct a network of herb-chemical-target interactions as an initial network. Protein-protein interactions (PPIs) of the predicted targets were retrieved from STRING v11.0 database^57^. STRING provides seven resources for physical and functional interactions. Among them, we selected experimental, databases, and coexpression resources with a minimum confidence score of 0.4. STRING network was superimposed on the initial network in Cytoscape to construct the ginger network.

Cytoscape plugin Network Analyzer^58^ was used to analyze the network and calculate its topological parameters such as degree centrality. To prioritize the hub genes, we removed any gene with a degree score lower than the average. The remaining genes with their corresponding chemicals and ginger compound were referred to as the ginger hub network. Then, “Cluster FI Network” option provided by Cytoscape plugin Reactome FI^59^ was used to cluster the hub network into sub-networks using spectral partition based network clustering algorithm^60^ and a size cutoff was used to filter out small modules.

To reveal biological insights into each network module, functional enrichment analysis including gene ontology (GO) terms and pathways was performed using “Analyze Module Functions” option provided by “Reactome FI” plugin. False discovery rate (FDR) value was limited to less than or equal to $1 \times 10^{-6}$ to prioritize the most significant terms.

Finally, SARS-CoV-2-human PPIs were retrieved from BioGRID 4.4 database^61^ and superimposed on the ginger hub network to highlight the viral protein interacting with human targets of ginger constituents.

### Molecular docking simulations

Molecular docking was performed to explore the potential binding of ginger constituents with SARS-CoV-2 proteins and human ACE2. Crystal structures of target proteins were retrieved from Protein Data Bank in PDB format^62^ as follows: (i) main protease with unliganded active site (M^pro^) [PDB ID: 6Y84]^63^. Molecular Operating Environment (MOE) v.2019.01 software^64^ was used for the preparation of proteins and ligands as well as data plotting. MOE SiteFinder module was used to select (M^pro^) binding pocket within its only chain. The site containing the catalytic dyade (His41 and Cys145 ) was selected with pocket coordinates (7.268, $-3.759$, 27.023) and size of 60 alpha spheres, (ii) spike receptor-binding domain (RBD) bound with ACE2 [PDB ID: 6M0J]^65^, only chain E was kept that represents spike RBD and binding pocket with coordinates ($-32.288$, 9.259, 28.774) and size of 60 alpha spheres was selected and (iii) human ACE2 receptor [PDB ID: 1R4L] as it is responsible for the virus attachment on the host cell^66^ Chain A was selected for ACE2 and binding pocket coordinates were centered on (42.699, 6.454, 25.753) with a pocket size of 60 alpha spheres (Table 1). The chemical structures of ligands were extracted from PubChem^67^ database in SDF format. Water molecules and ions were removed from protein structures and only main-chain amino acids were retained which are essential for binding of the co-crystallized ligand. Polar hydrogen atoms were added by employing the 3D protonation feature in MOE. ASN, GLN, and HIS flips were allowed during 3D protonation. Then, energy minimization was carried out for the three complexes employing AMBER10:EHT force field for energy minimization; where AMBER parameters are suitable for proteins and nucleic acids (ff10); EHT parameters are suitable for small molecules then they were refined to RMS Gradient of 0.1 Kcal/mol/Å. Docking was carried out in the same site of the co-crystallized ligand for both RBD and ACE2 structures while MOE SiteFinder was used to choose the docking site based on a literature search for M^pro^ structure. For docking scoring, triangle matcher placement was chosen; the first rescoring function was set to be London dG while GBVI/WSA dG was chosen to be the second rescoring function and finally, it was refined using forcefield retaining 30 docked structures for each compound. Root mean square deviation (RMSD) values between the docked conformation and the reference conformation presented in Å  were utilized^68^ to validate the docking performance.

**Table 1:** Binding pockets of the target proteins for docking simulations.

Target molecule	PDB ID	Chain	Coordinates	Size (alpha spheres)
SARS-CoV-2 M^pro^	6Y84	A	X: 7.268, Y:-3.759, Z: 27.023	60
SARS-CoV-2 RBD	6M0J	E	X: $-32.288$, Y: 9.259, Z: 28.774	60
Human ACE2	1R4L	A	X: 42.699, Y: 6.454, Z: 25.753	60

### Molecular dynamics (MD) simulations

Among all compounds, (6)-gingerdiacetate displayed the best binding interactions and free energies over the three target proteins. Accordingly, It was chosen to be subjected to 100 nanoseconds (ns) molecular dynamics (MD) simulation against the three target proteins to confirm its stable binding affinity towards them. MD simulations were performed using GROningen MAchine for Chemical Simulations (GROMACS) 2020.6 software package^69^ where the CHARMM36 forcefield was used for protein topology preparation and the official CHARMM General Force Field server (CGenFF) for ligand topology preparation. The solvation method used here was a dodecahedron box of common simple point charge (SPC) water model where explicit solvent periodic boundary conditions were applied. Charge neutralization using sodium and chloride ions was carried out for the solvated complexes. The systems were subjected to energy minimization to resolve any steric clashes or inappropriate geometry employing the steepest descent method through 5000 steps. System equilibration was also manipulated to ensure a reasonable starting structure using NVT; equilibration under constant number of particles, volume, and temperature (NVT) for 100 picoseconds (ps) using a Berendsen thermostat^70^. Also, re-equilibration was performed for another 100 ps under constant pressure (Isothermal-isobaric (NPT) ensemble) using the Parrinello-Rahman barostat using a time step of 2 femtoseconds (fs) for each equilibration round^71^. Finally, MD production phase was carried out for 100 ns using a time step of 2 fs at a constant temperature of 300 ^∘^K and constant pressure at 1 atm. For MD trajectories analysis, the complexes were re-centered and re-wrapped within the unit cells using the “trjconv” function of GROMACS. Then, they were analyzed using RMSD of the protein backbone vs the protein-ligand complex referenced to its initial position and the radius of gyration (Rg). GRaphing and Advanced Computation and Exploration of data (Grace) tool was used for data plotting.

### Experimental procedure

#### Plant material

Fresh ginger rhizomes were obtained from the Agricultural Research Center, Ministry of Agriculture, Cairo. A voucher specimen was deposited at the Herbarium of the National Research Centre under the accession number M205. We declare that the collection of plant material is in agreement with relevant institutional, national, and international guidelines and legislation.

#### Hydro-distillation extraction of ginger volatile oil

Fresh ginger rhizomes (200 g) were hydro-extracted with Clevenger-type apparatus for 3 h. The oily layer was isolated separately by *n*-hexane, then dried using (0.5 g) anhydrous Na_2_SO_4_, and finally stored in glass vials at $-20^{\circ }$C for further analysis.

#### GC–MS analysis of ginger volatile oil

GC–MS analysis was performed using an Agilent Technologies system (7890B), equipped with a mass spectrometer detector (5977A), headspace sampler (7697A), and HP-5MS capillary column (30 m $\times$ 0.25 mm internal diameter and $0.25\,\upmu \hbox {m}$ film thickness). Hydrogen gas was used as the carrier gas for the analysis at a flow rate of 1.0 ml/min with a splitless injection mode. The following temperature program was adopted: 50 ^∘^C for 2 min; rising at 10 ^∘^C /min to 250 ^∘^C; rising at 15 ^∘^C/min to 300 ^∘^C and held for 10 min. The injector and detector were held at 280 ^∘^C and 300 ^∘^C, respectively. Mass spectra were obtained by electron ionization (EI) at 70 eV; using a spectral range of *m/z* 30–550 and solvent delay 5 min. Identification of different constituents was determined by comparing the fragmentation pattern with those stored in Wiley and NIST Mass Spectral Library data.

#### Headspace GC–MS for volatile analysis

Three grams of fresh ginger rhizomes were placed into a 20 ml headspace vial and immediately sealed with silicone rubber septa and aluminum caps for the absorption of the volatile compounds. They were transferred to the headspace and heated up to 80 ^∘^C for 20 min while being agitated, and then introduced directly into the GC injector with a loop temperature of 120^∘^C, and transfer line temperature of 140 ^∘^C. GC–MS analysis was executed on an Agilent Technologies system (7890B), equipped with a mass spectrometer detector (5977A), headspace sampler (7697A), and HP-5MS capillary column (30 m $\times$ 0.25 mm internal diameter and $0.25\,\upmu \hbox {m}$ film thickness). Analyses were carried out as previously described in^72^.

#### Ginger extraction for LC–MS analysis

Fresh ginger rhizomes were cut into small parts (250 g) and extracted with methanol: water (7:3 v/v). The solvent was then evaporated under reduced pressure till dryness and kept refrigerated for further analysis.

#### LC–MS/MS analysis of ginger hydroalcoholic extract

Instrument: The analysis of the sample was performed using liquid chromatography–electrospray ionization–tandem mass spectrometry (LC–ESI–MS/MS) with an ExionLC AC system for separation and SCIEX Triple Quad 5500+ MS/MS system equipped with electrospray ionization (ESI) for detection.

Conditions: The separation was performed with a Ascentis$\circledR$ Express 90 Å C18 Column (2.1 $\times$ 150 mm, $2.7\,\upmu \hbox {m}$). For MS/MS analysis, negative ionization mode was applied with a scan (EMS–IDA–EPI) from 100 to 1000 Da for MS_1_ with the following parameters: curtain gas: 25 psi; IonSpray voltage: $-4500$; source temperature: 500 ^∘^C; ion source gas 1 & 2 were 45 psi and from 50 to 1000 Da for MS_2_ with a declustering potential: $-80$; collision energy: $-35$; collision energy spread: 15. Compounds’ identification was performed using MS-DIAL. The mobile phases consisted of two eluents A: 5 mM ammonium formate pH 8; B: acetonitrile (LC grade). The mobile phase gradient was programmed as follows: 5% B at 0–1 min, 5–100% B from 1–20 min, 100% B from 20 to 25 min, 5% at 25.01, 5% from 25.01–30 min. The flow rate was 0.3 ml/min and the injection volume was 5 $\upmu \hbox {l}$.

For MS/MS analysis, positive ionization mode was applied with a scan (EMS-IDA-EPI) from 100 to 1000 Da for MS_1_ with the following parameters: curtain gas: 25 psi; IonSpray voltage: 5500; source temperature: 500 ^∘^C; ion source gas 1 & 2 were 45 psi and from 50 to 1000 Da for MS_2_ with a declustering potential: 80; collision energy: 35; collision energy spread: 15. The mobile phases consisted of two eluents A: 5 mM ammonium formate pH 3; and B: acetonitrile (LC grade).

#### Quantitative determination of pungent substances in the ginger crude extract

The separation of gingerol and shagaol was carried out by Waters alliance HPLC equipped with a photodiode array detector and oven. The mobile phases consisted of two eluents A: water 0.5% Acetic acid; B: acetonitrile 0.5% acetic acid (HPLC grade). The mobile phase gradient was programmed as follows: (65% B–35% A) at 0-9.5 min, (75 % B–25% A) from 10 to 16 min, (65% B–35%A) from 16.10 to 20 min. The flow rate was 2.0 ml/min and the injection volume was 25 $\upmu \hbox {l}$. Column C18 (250 $\times$ 4.6 mm 5 $\upmu$m), column temperature 25 ^∘^C

#### MTT cytotoxicity assay

To assess the half-maximal cytotoxic concentration (CC50), stock solutions of the HD oil were prepared in 10% DMSO in ddH_2_O and diluted further to the working solutions with DMEM. The cytotoxic activity of the extracts was tested in VERO-E6 cells by using the 3-(4, 5-dimethylthiazol -2-yl)-2, 5-diphenyltetrazolium bromide (MTT) method with minor modifications^73^.

#### Determination of the antiviral activity of ginger, determination of the Inhibitory concentration 50 (IC50)

Determination of the antiviral activity of ginger hydroalcoholic extract and the hydrodistilled oil against SARS-CoV-2 was performed as described in^73^.

## Results

### Screening of ginger compounds and predicted targets

In general, ginger contains over 400 chemical compounds that vary from one place to another^74^. We retrieved a total of 34 constituents that were found as the most common phytochemicals of ginger rhizomes. All compounds meet the RO5 criteria and were used for network and docking analyses as listed in Table S1. In total, 5225 predicted targets were retrieved from SEA, TargetNet, and SwissTargetPrediction databases for the 34 ginger compounds (Table S2). Pooling and filtration resulted in 784 distinct genes where 29 genes are common among the three databases. Across all databases, the pungent compounds including gingerols and shogaols^75^ have the highest number of predicted targets.

### Biological insights into the mechanism of ginger constituents

**Figure 2:**
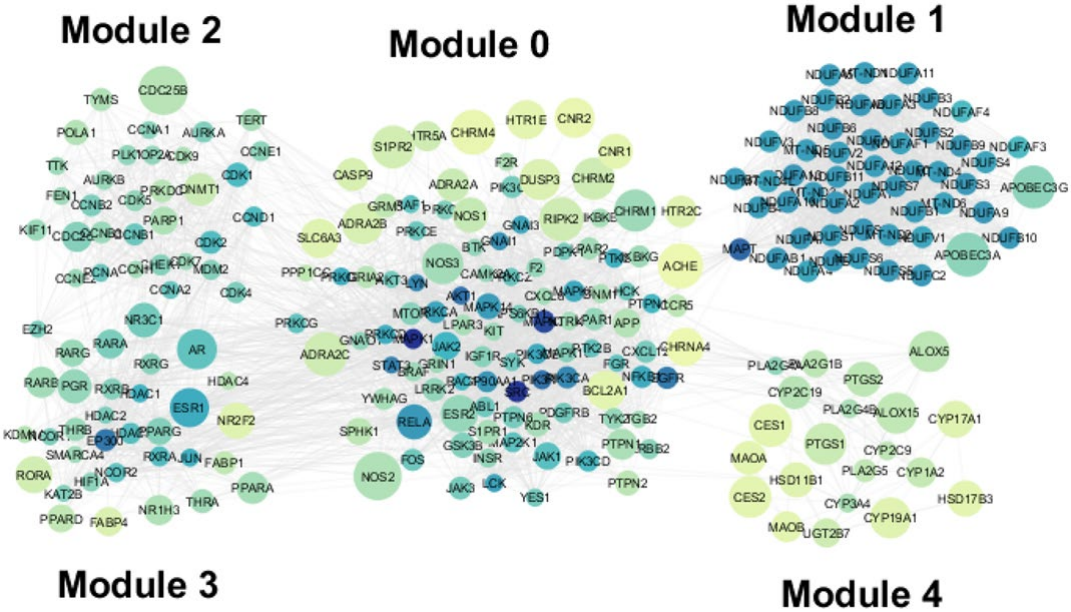
The hub targets network is clustered into five network modules, covering 245 hub nodes connected by 3500 edges. The node’s color depth is mapped to degree centrality and the node’s size is mapped to the count of corresponding chemicals.

Human protein-protein interactions (5849 queries) were downloaded from STRING connecting 696 targets (Table S3). PPIs network was merged with the herb-chemical-target network, resulting in the ginger network that consists of 731 nodes (1 herb, 34 chemicals, and 696 target proteins) connected by 9976 edges. The initial network was filtered based on degree centrality by removing any node with a degree below the average (23), resulting in 284 nodes (1 herb, 34 chemicals, and 249 hub targets) connected by 5740 edges. The hub network was clustered into 7 highly connected groups of genes defined as network modules (Table S4). Two modules of size 2 were removed as they were too small while the other five modules ranged in size from 21 to 107 nodes as shown in Fig. 2.

Functional enrichment analysis was performed to identify the most enriched cellular components (CC), biological processes (BP), molecular functions (MF), and pathways of each network module. The analysis resulted in 28, 102, and 65 for GO terms as CC, BP, and MF, respectively, and 409 pathways with FDR range from $1 \times 11^{-16}$ to $1 \times 10^{-6}$. The top GO terms and pathways of each module are given in Table 2 and Table 3, respectively. GO analysis identified that module 0 is enriched in plasma membrane (CC), signal transduction and inflammatory response (BP), and protein kinase and ATP binding (MF). Module 1 is enriched in mitochondrion (CC) as it is associated with aerobic respiration-related processes. Module 2 is mainly involved in cell cycle and cell division. Module 3 is associated with regulation of RNA metabolic pathways while module 4 is involved in lipid metabolism and xenobiotic metabolic process. Reactome pathway analysis provided more detailed terms such as PI3K-Akt, Rap1, Ras, MAPK, PDGFR-beta, and cAMP signaling pathways that are significantly enriched in module 0. Enriched pathways with each module are matching with the previously mentioned GO terms. Additionally, Reactome identified infectious disease pathways that are significantly enriched with gene sets of module 0 such as Human cytomegalovirus infection, Hepatitis B, and Human immunodeficiency virus 1 infection, and module 2 such as Human T-cell leukemia virus 1 infection.

**Table 2:** The enriched GO terms of the five modules covering hub genes.

Module	Category	Term	Gene count	FDR
0	CC	Plasma membrane	81	2.92E−14
0	CC	Cytoplasm	70	5.82E−14
1	CC	Mitochondrion	32	1.78E−15
2	CC	Nucleus	33	1.29E−14
3	CC	Nucleoplasm	32	3.55E−15
3	CC	Chromatin	26	3.55E−15
4	CC	Endoplasmic reticulum membrane	12	2.67E−09
0	BP	Signal transduction	34	6.00E−12
0	BP	Platelet activation	25	2.21E−14
0	BP	Negative regulation of apoptotic process	23	7.53E−11
0	BP	Inflammatory response	22	2.61E−11
0	BP	Positive regulation of cell migration	20	1.83E−13
1	BP	Mitochondrial electron transport, NADH to ubiquinone	46	7.11E−15
1	BP	Mitochondrial respiratory chain complex I assembly	46	7.11E−15
2	BP	Cell division	17	2.92E−14
2	BP	Regulation of cyclin-dependent protein serine/threonine kinase activity	12	2.92E−14
3	BP	Negative regulation of transcription by RNA polymerase II	25	2.41E−14
3	BP	Positive regulation of transcription by RNA polymerase II	28	2.41E−14
3	BP	Negative regulation of transcription, DNA-templated	17	5.43E−14
3	BP	Positive regulation of transcription, DNA-templated	18	5.77E−14
3	BP	Regulation of transcription by RNA polymerase II	17	4.92E−07
4	BP	Long-chain fatty acid biosynthetic process	6	2.02E−10
4	BP	Xenobiotic metabolic process	6	3.52E−08
0	MF	ATP binding	54	8.33E−15
0	MF	Protein kinase binding	26	4.12E−14
1	MF	NADH dehydrogenase (ubiquinone) activity	43	2.78E−15
1	MF	Electron transfer activity	6	9.82E−07
2	MF	Protein kinase binding	15	1.80E−12
3	MF	Transcription factor binding	18	4.33E−15
3	MF	Zinc ion binding	20	4.33E−15
3	MF	Nuclear receptor activity	18	4.33E−15
4	MF	Iron ion binding	8	5.03E−11

**Table 3:** The enriched Reactome pathways of the five modules covering hub genes.

Module	Pathway term	Gene count	FDR
0	PI3K-Akt signaling pathway(K)	44	6.66E−16
0	Chemokine signaling pathway(K)	35	6.66E−16
0	Human cytomegalovirus infection(K)	35	6.66E−16
0	Rap1 signaling pathway(K)	34	6.66E−16
0	Kaposi sarcoma-associated herpesvirus infection(K)	33	6.66E−16
0	Hepatitis B(K)	31	6.66E−16
0	Human immunodeficiency virus 1 infection(K)	31	6.66E−16
0	Ras signaling pathway(K)	30	6.66E−16
0	MAPK signaling pathway(K)	28	6.66E−16
0	PDGFR-beta signaling pathway(N)	28	6.66E−16
0	cAMP signaling pathway(K)	27	6.66E−16
0	ErbB signaling pathway(K)	25	6.66E−16
0	Coronavirus disease—COVID-19(K)	24	6.66E−16
0	mTOR signaling pathway(K)	21	6.66E−16
0	VEGF signaling pathway(K)	21	6.66E−16
1	The citric acid (TCA) cycle and respiratory electron transport(R)	47	2.22E−16
1	Oxidative phosphorylation(K)	38	2.22E−16
2	Cell cycle(K)	21	9.44E−15
2	Mitotic G1 phase and G1/S transition(R)	16	9.44E−15
2	Cellular senescence(K)	14	9.44E−15
2	Mitotic G2-G2/M phases(R)	12	1.74E−12
2	S Phase(R)	11	1.42E−11
2	Viral carcinogenesis(K)	10	4.56E−09
2	Human T-cell leukemia virus 1 infection(K)	10	8.42E−09
2	Cell cycle checkpoints(R)	9	4.07E−07
2	Human papillomavirus infection(K)	9	3.12E−06
3	RNA polymerase II transcription(R)	28	7.11E−15
3	SUMOylation(R)	17	7.11E−15
3	Thyroid hormone signaling pathway(K)	13	7.11E−15
4	Metabolic pathways(K)	19	4.89E−14

**Figure 3:**
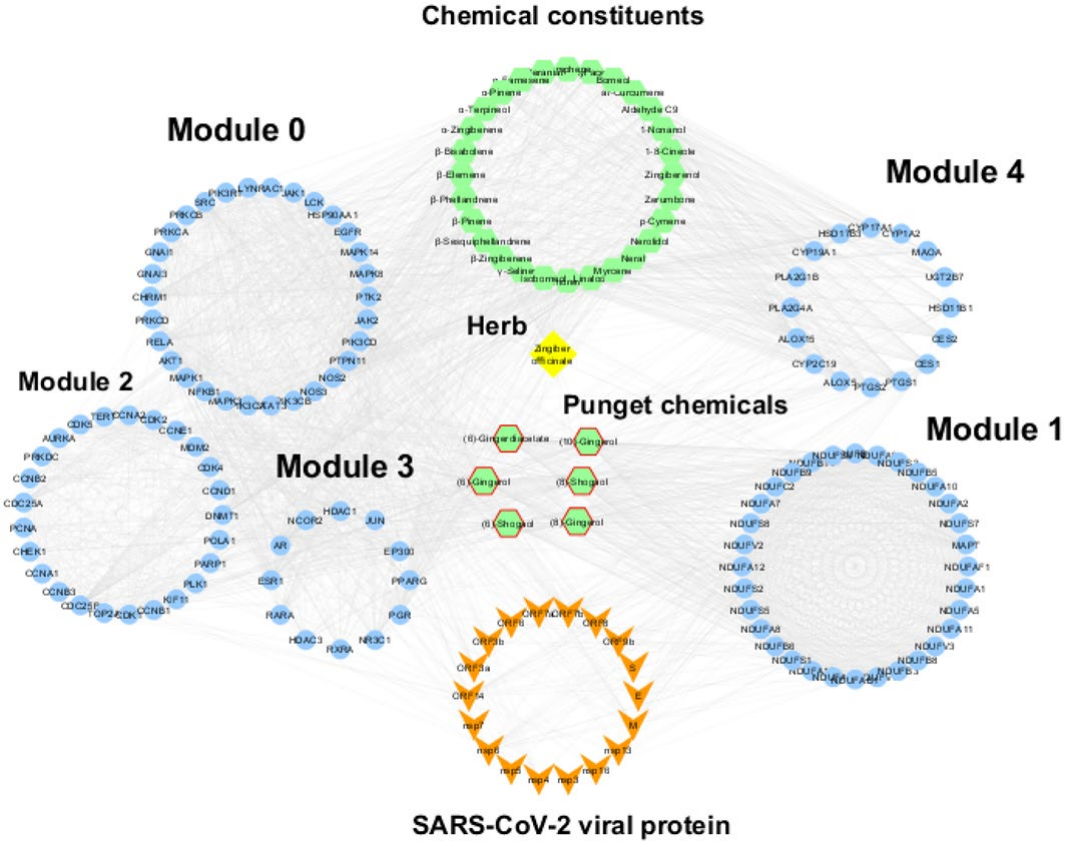
An integrative network consists of 307 nodes and 5889 edges. Each type of node is represented by a specific color and shape. Nodes with low degree centrality were filtered out for better visualization.

Finally, superimposing SARS-CoV-2-human PPIs on the ginger hub network highlighted 71 target genes interacting with 27 viral proteins (Table S5). Top 10 viral proteins include structural proteins such as membrane (M), envelope (E), and spike (S) which are connected to 30, 18, and 12 hub targets, respectively. Accessory proteins (ORF7b, ORF14, ORF7a, ORF9b, and ORF3a), and non-structural proteins (nsp6 and nsp4) are also ranked within top 10 with degree of 11 to 26. The top connected human targets to viral proteins belong to module 0 (SRC, PTPN1, and RAC1), module 1 (NDUFS1, NDUFS2, and NDUFV2), and CES1 from module 4 as they are connected to at least seven viral proteins. For an integrative view, a full network was constructed to combine all types of nodes (1 herb, 34 chemicals, 245 corresponding hub targets, and 27 SARS-CoV-2 viral proteins) with different interaction types as shown in Fig. 3.

### Identification of candidate chemicals against COVID-19 infection based on docking analysis

**Figure 4:**
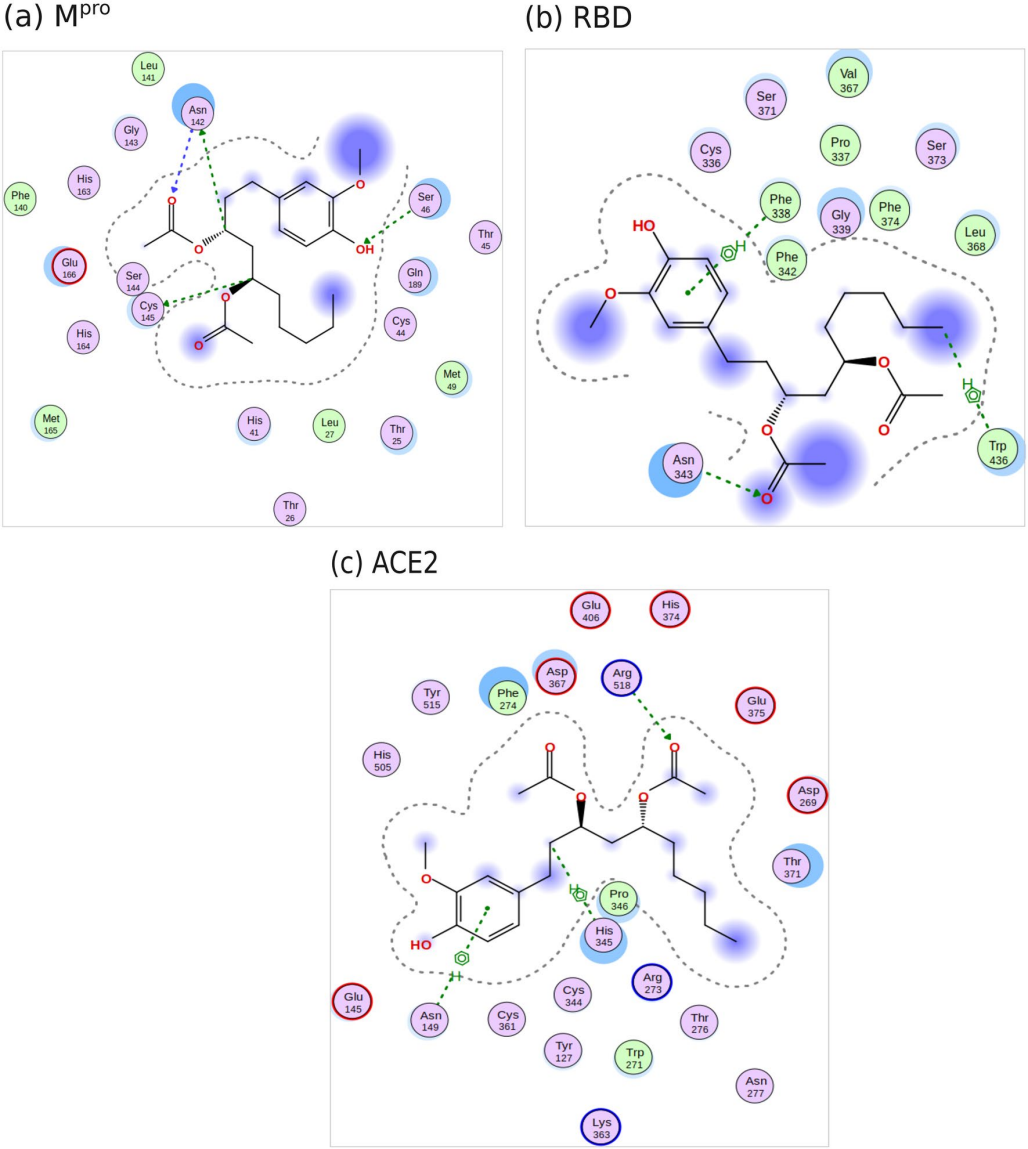
Two-dimensional representation of protein-ligand interactions between (6)-gingerdiacetate and the target proteins; (**a**) M^pro^ (PDB ID: 6Y84); (**b**) RBD of spike protein (PDB ID: 6M0JE); (**c**) human ACE2 (PDB ID: 1R4L). Images were created using MOE v.2019.01.

Molecular docking simulations showed that gingerols and shogaols which are the major phenolic compounds in ginger performed better than other compounds against the three target proteins (Table S6). The six top-ranked constituents based on docking score $\Delta$G (Kcal / Mol) with two SARS-CoV-2 receptors and human receptor protein ACE2 are listed in Table 4. (6)-Gingerdiacetate has the best docking scores of $-8.19$ Kcal/mol and $-7.23$ Kcal/mol against M^pro^ (6Y84) and ACE2 (1R4L), respectively. For the RBD of spike protein (6M0JE), (10)-gingerol and (8)-gingerol scored $-6.91$ Kcal/mol and $-6.55$ Kcal/mol, respectively, which are slightly lower than (6)-gingerdiacetate score of $-6.22$ Kcal/mol, meaning that they have better affinity towards RBD than the other 2 targets unlike (6)-gingerdiacetate that shows better affinity towards M^pro^ and ACE2. The interactions formed between the three top-ranked ligands and the target proteins were visualized using MOE to select the most favorable compound for further analysis. We found that (6)-gingerdiacetate formed four hydrogen bonds with M^pro^ pocket (Ser40, Asn142, Cys145) as shown in Fig. 4a while each of (10)-gingerol and (8)-gingerol formed only two hydrogen bonds. Although (10)-gingerol has a better score with RBD compared to (6)-gingerdiacetate, it only formed hydrophobic interactions with the protein structure, without forming any hydrogen bonds. Similarly, (6)-gingerdiacetate and (10)-gingerol have almost the same docking score with ACE; however, only (6)-gingerdiacetate formed hydrogen interactions with ACE2 (Fig. 4c). Accordingly, (6)-gingerdiacetate showed the best interactions with the best binding scores against the three target proteins. In turn, to assess the postulate that (6)-gingerdiacetate is the best candidate as a potential inhibitor targeting SARS-CoV-2 M^pro^, spike RBD and Human ACE2, 100 ns MD simulations were carried out to confirm the stability of the three complexes.

**Table 4:** Docking scores in (kcal/mol) of top six ranked ginger constituents against target proteins.

Compound	Docking score $\Delta$G (kcal/mol)		
	M^pro^	RBD	ACE2
(6)-Gingerdiacetate	$-7.23$	$-6.22$	$-8.19$
(10)-Gingerol	$-6.79$	$-6.91$	$-8.11$
(8)-Gingerol	$-6.74$	$-6.55$	$-7.86$
(6)-Gingerol	$-6.29$	$-6.28$	$-6.77$
(6)-Shogaol	$-6.16$	$-5.89$	$-7.51$
(8)-Shogaol	$-6.58$	$-5.98$	$-7.22$
Zingiberenol	$$-5.85$$	$$-5.12 $$	$$-6.12$$

### MD analysis of the best docked ligand with target proteins

**Figure 5:**
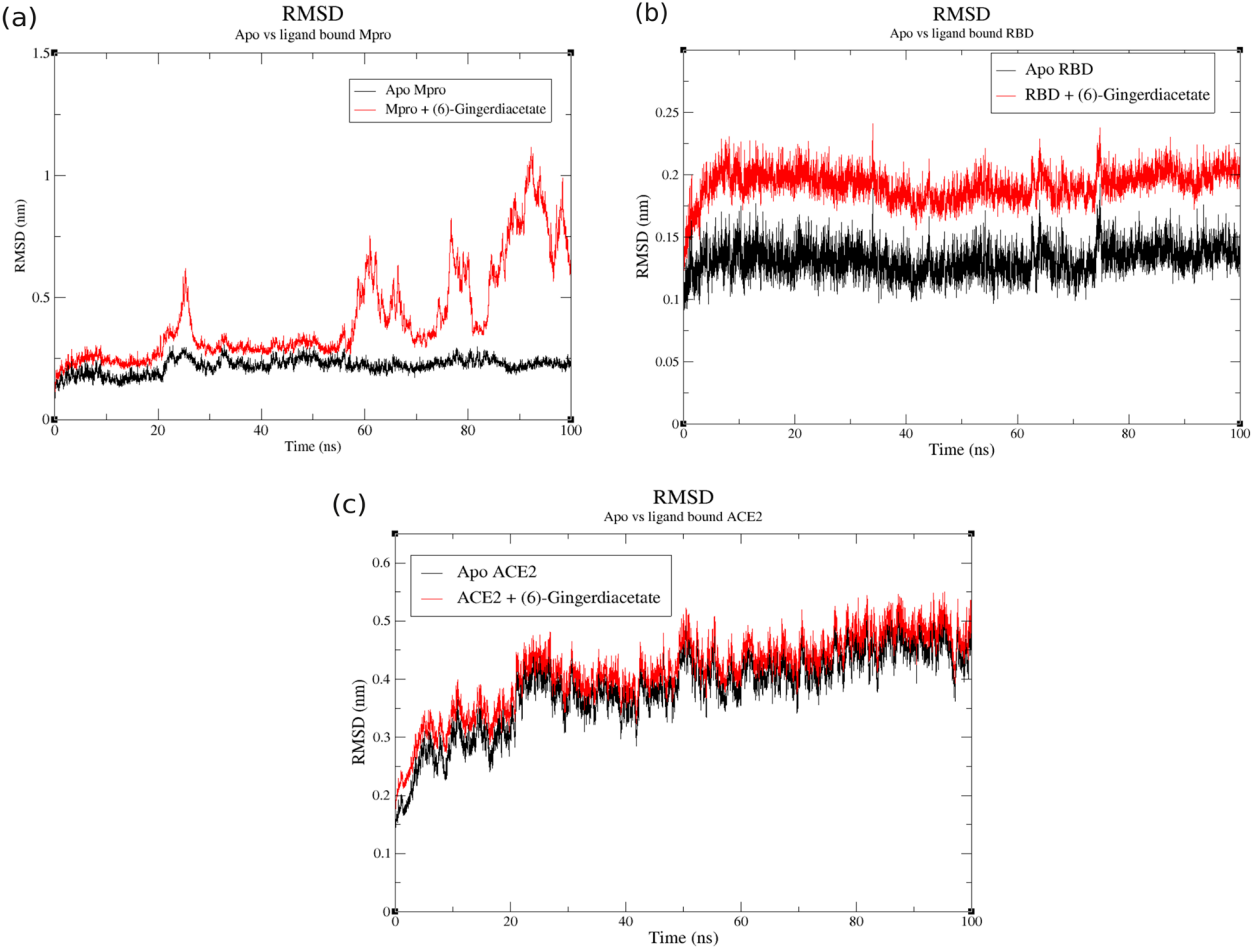
RMSD plots of target proteins in apo structure vs (6)-gingerdiacetate-protein complexes over 100 ns simulation run: (**a**) apo and ligand-bound M^pro^; (**b**) apo and ligand-bound RBD; (**c**) apo and ligand-bound ACE2. Apo structures and protein-ligand complexes are colored in black and red, respectively.

**Figure 6:**
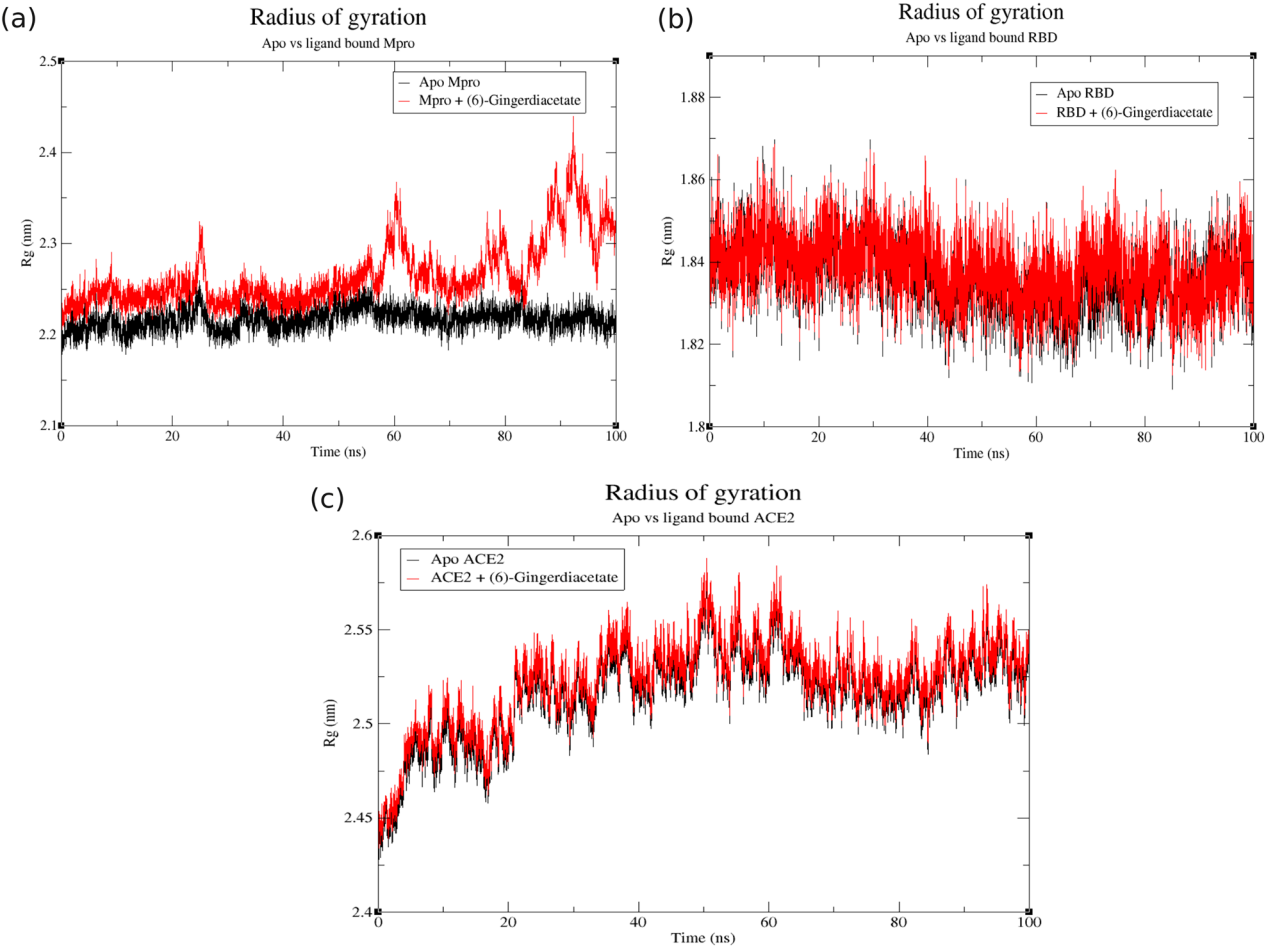
Radius of gyration (Rg) plots of target proteins in apo structure vs (6)-gingerdiacetate-protein complexes over 100 ns simulation run: (**a**) apo and ligand-bound M^pro^, (**b**) apo and ligand-bound RBD; (**c**) apo and ligand-bound ACE2. Apo structures and protein-ligand complexes are colored in black and red, respectively.

According to docking analysis, (6)-gingerdiacetate was selected for MD simulations as it has the minimum binding energy scores and best hydrogen interactions as well with the viral and human receptors. MD trajectories over 100 ns for the three complexes were analyzed to investigate the stability of the proteins with bound ligand using RMSD parameter as shown in Fig. 5. Interestingly, M^pro^-ligand complex showed similar deviation as the native structure in the simulation period from 25 ns to 55 ns with very low fluctuations, but afterward it shows a very high fluctuation till the end of simulation with RMSD between 0.5 nm to 1 nm, indicating that the ligand-complex lost its stability after about 55 ns and the ligand left the binding pocket of the receptor as shown in Fig. 5a. On the other hand, RBD and ACE2-ligand complexes were stable and showed similar RMSD patterns to their native structures (Fig. 5b, c), respectively) with very low fluctuation $\sim 0.05$ nm for the RBD throughout the whole 100 ns simulation. ACE2 complex reached stability throughout the last 50 ns in the simulation with $\sim 0.05$ nm fluctuation as well. To confirm the complexes’ stability and evaluate the effect of ligand binding on the protein structure, radius of gyration (Rg) was calculated for each protein in apo and ligand-bound form as shown in Fig. 6. Similar to RMSD plots, M^pro^-ligand complex shows less stability than the native system at $\sim 60$ ns confirming its unstable binding in long simulation (Fig. 6a). RBD and its respective ligand complex have the same pattern of Rg values which ranges from 1.82 nm to 1.86 nm (Fig. 6b). The binding of the ligand to ACE2 did not change its structure as both systems exhibited almost the same pattern as well with a bit higher values of Rg (2.5 nm to 2.6 nm), confirming the stability of these two complexes (Fig. 6c). Overall, MD analysis of 100 ns for RBD and ACE2 systems show consistency with their corresponding docking results while only the first 50 ns of M^pro^ are consistent with docking analysis.

### GC–MS analysis of the hydro-distilled oil (HD) of ginger rhizomes

Results of the GC–MS analysis of the hydro-distilled (HD) oil of ginger rhizomes are listed in Table 5. Overall, 33 volatile components were identified belonging to different metabolite classes such as monoterpenes and sesquiterpenes hydrocarbons, and their oxygenated congeners. Compounds identification was based on their mass spectral data (MS) and relative retention time compared to those of Wiley spectral library collection and NIST library databases.

**Table 5:** Volatile composition of the hydro-distilled (HD) oil of ginger rhizomes as analyzed by GC–MS.

Peak	Rt	Name	Percentage
Monoterpene hydrocarbons			
1.	3.999	$\beta$-myrcene	0.95
2.	**4.604**	**d**-**limonene**	1.41
3.	4.809	Sabinene	7.4
Total monoterpene hydrocarbons			9.76
Oxygenated monoterpenes			
4.	4.87	Eucalyptol (1,8-cineole)	7.03
5.	11.82	Isopulegol	0.35
6.	14.12	Linalool	0.81
7.	17.35	**Z-citral**	13
8.	17.9	**l**-**borneol**	4.42
9.	17.98	$\alpha$-Terpineol	2.63
10.	18.82	**E-citral (geranial)**	7.33
11.	20.05	$\beta$-Citronellol	0.81
12.	22.04	Geraniol	1.61
Total oxygenated monoterpenes			37.99
Ketones			
13.	7.86	6-Methyl-5-hepten-2-one	0.64
14.	15.38	2-Undecanone	0.45
Total ketones			
Monoterpene ester			
15.	14.48	Endobornyl acetate	0.36
Total ketones			0.45
Monoterpene esters			
16.	14.48	Endobornyl acetate	0.36
Total monoterpene esters			0.36
Sesquiterpene hydrocarbon			
17.	16.34	$\gamma$-Elemene	0.37
18.	18.24	$\gamma$-Muurolene	0.62
19.	18.38	valencene	0.36
20.	18.75	$\beta$-Sesquiphellandrene	29.38
21.	18.87	$\beta$-Bisabolene	1.33
22.	19.61	$\alpha$-Farnesene	2.27
23.	19.93	$\alpha$-Curcumene	8.12
Total sesquiterpene hydrocarbon			42.45
Oxygenated sesquiterpenes			
24.	25.55	Ledene oxide-(II)	0.32
25.	26.86	Nerolidol	0.63
26.	27.45	Elemol	1.42
27.	28.07	***Trans*-sesquisabinene hydrate**	0.99
28.	28.3	***Cis*-sesquisabinene hydrate**	1.44
29.	29.59	Cubebol	0.4
30.	29.96	Zingiberenol	0.84
31.	30.76	Eudesmol	0.67
32.	31.23	Caryophyllene oxide	0.67
33.	35.87	$\alpha$-Copaen-4-ol	0.43
Total oxygenated sesquiterpenes			7.81

### Headspace GC–MS analysis of fresh ginger rhizomes

Further, headspace GC–MS analysis was performed to compare the volatiles of HD oil to those of the fresh ginger rhizomes, Table 6.

**Table 6:** Volatile composition of the Headspace GC–MS analysis of ginger rhizomes.

Peak	Rt	Name	Percentage
Monoterpene hydrocarbons			
1.	2.402	$\alpha$-Pinene	6.73
2.	2.747	Camphene	25.13
3.	3.943	$\beta$-Myrcene	3.51
4.	4.511	d-limonene	4.3
5.	4.684	Sabinene	30.64
Total monoterpene hydrocarbons			70.31
Monoterpene ester			
6.	3.151	Linalyl acetate	0.5
Total monoterpene esters			0.5
Oxygenated monoterpenes			
7.	4.958	Eucalyptol (1,8-cineole)	2.06
8.	17.229	*cis*-Verbenol	0.88
Total oxygenated monoterpenes			2.94
Aldehydes			
9.	6.783	Octanal	1.11
10.	7.562	2-Heptenal, (E)-	0.79
11.	9.532	Nonanal (aldehyde C9)	2.16
12.	12.529	Decanal	0.53
13.	16.341	2-Decenal, (E)-	1.73
14.	20.607	2,4-Decadienal, (E,Z)-	1.28
Total aldehdes			7.6
Alcohols			
15.	10.329	2-Heptyn-1-ol	0.5
16.	21.858	1-Octanol, 2-butyl-	3.26
Total alcohols			3.76
Sesquiterpene hydrocarbon			
17.	11.937	Aromandendrene	0.7
18.	12.329	$\alpha$-Copaene	0.72
19.	18.625	Zingiberene	7.94
20.	18.776	$\beta$-Bisabolene	1.42
21.	19.519	$\alpha$-Farnesene	1.03
22.	19.821	Ylangene	2.5
Total sesquiterpene hydrocarbon			12.89
Ketones			
23.	15.38	2-Undecanone	0.58
Total ketones			0.58

### Phytochemical analysis of hydroalcoholic extract of ginger by LC/MS–MS

For the characterization of the nonvolatile constituents present in the hydroalcoholic extract of ginger rhizomes, liquid chromatography coupled to tandem mass spectrometry (LC–MS/MS) was recruited. The LC–MS/MS analysis of the hydroalcoholic extract of ginger rhizomes reflected the prevalence of 6-, 8-, and 10-gingerol, (6)-gingerdiacetate, and 6-, 8- and 10-shogaol, with fragmentation patterns agreeing with that reported in the literature (Table 7).

**Table 7:** LC/MS–MS analysis of hydroalcoholic extract of ginger.

Compound	Rt	[M − H]−	[M − H]+	MS/MS	References
10-Gingerol	2.88	349.1		349, 193, 179, 155	^76^
6-Gingerol	12.57	293.1		193, 139, 275	^76^
8-Shogaol	12.68		305.1	137	^77^
8-Gingerol	15.75	321.1		193, 127, 178	^76^
6-Shagaol	16.29	275.1		275, 139	^76^
(6)-Gingerdiacetate	17.35		403.1 [M+Na]+	403,261	^78^
10-Shagaol	21.37	331.1		331,195	^76^

### Quantitative determination of the pungent principles of ginger hydroalcoholic extract by HPLC

**Figure 7:**
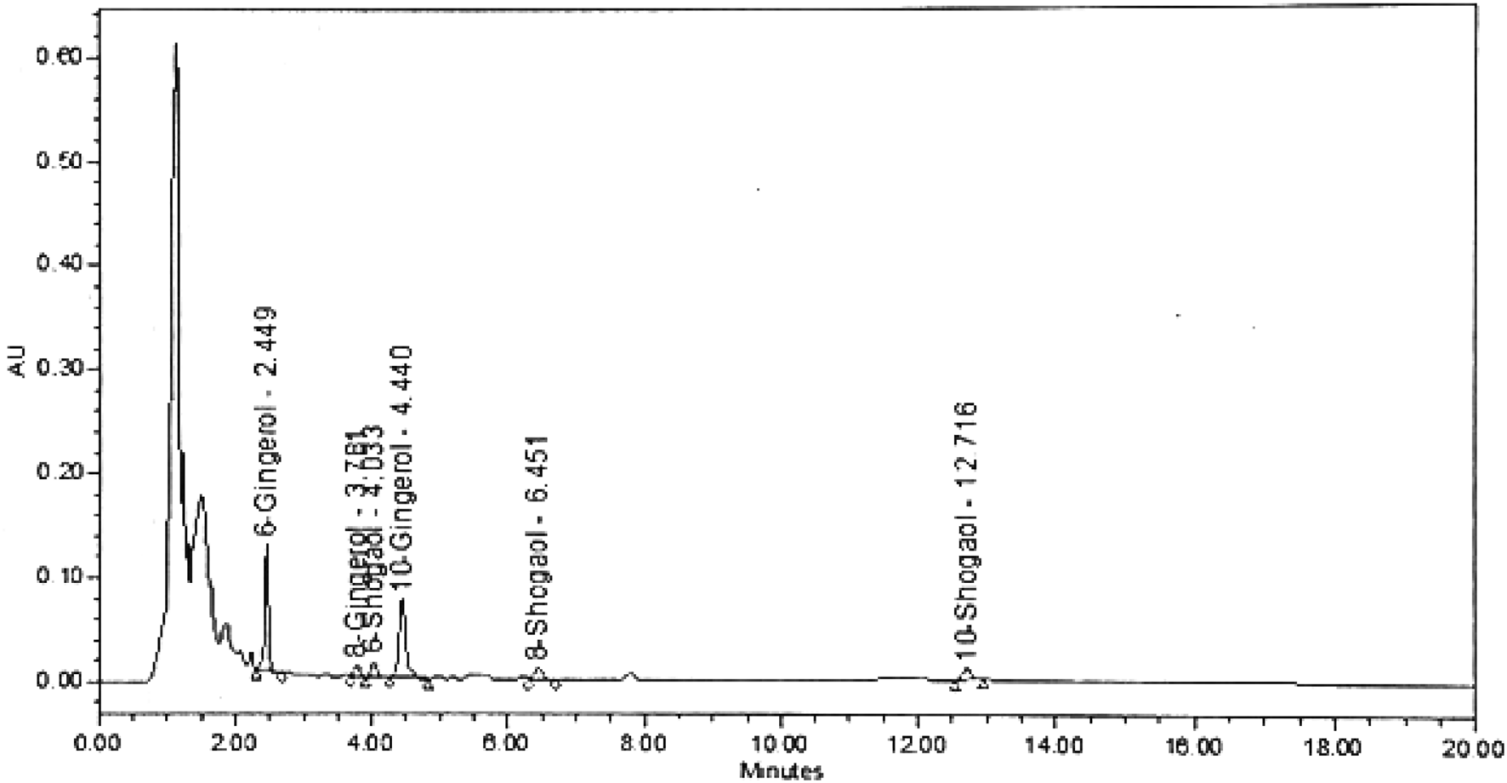
HPLC chromatogram of the ginger hydroalcoholic extract.

Quantitative determination of the pungent principles revealed the prevalence of 10-gingerol followed by the 6-gingerol as listed in Table 8 and shown in Fig. 7

**Table 8:** Quantitative determination of pungent substances in the ginger hydroalcoholic extract.

No	Compounds	RT	Area%	Concentrations %
1	6-Gingerol	2.449	35.26	0.091
2	8-Gingerol	3.781	4.99	0.012
3	10-Gingerol	4.44	39.04	0.031
4	6-Shogaol	4.033	7.22	0.077
5	8-Shogoal	6.451	7.42	0.016
6	10-Shogoal	12.716	6.06	0.021
Sum of Gingerols				0.135
Sum of Shogaols				0.115
Sum of pungent substances: 6,8,10-gingerols, 6,8,10-shogoals				0.25

### In vitro antiviral activity against SARS-CoV-2 virus

**Figure 8:**
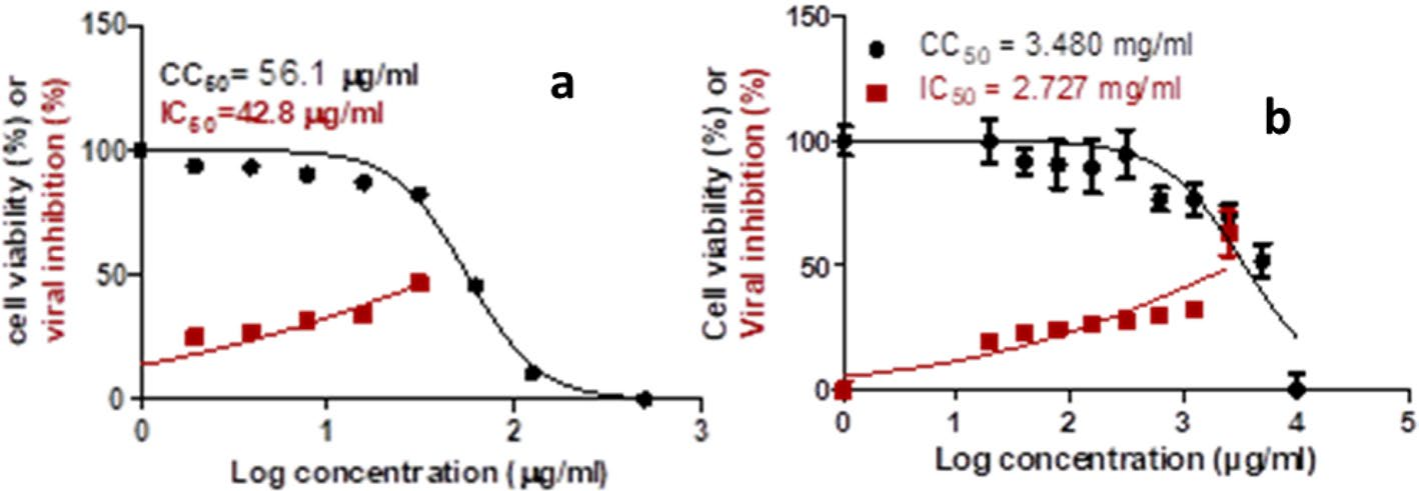
Dose response and inhibition curves for ginger hydro-distilled oil (**a**) and ginger hydroalcoholic extract (**b**) showing the half maximal cytotoxic concentration; CC_50_ in Vero E6 cells and inhibitory concentration 50% (IC_50_) against NRC-03-nhCoV which was calculated by using the nonlinear regression analysis of the GraphPad Prism.

Initially, the cytotoxicity assay was performed to determine the safety of the ginger and the HD oil. The CC_50_ of the ginger hydroalcoholic extract on Vero E6 cells was 3.480 $\upmu \hbox {g}/\hbox {ml}$ while the CC_50_ of the HD ginger oil on Vero E6 cells was 56.1 $\upmu \hbox {g}/\hbox {ml}$ (Fig. 8). Following, the extract and the HD oil were tested for the inhibition of the SARS-CoV-2 virus replication. Inhibitory concentration (IC_50_) was calculated to determine the dose that causes inhibition of 50% of the virus replication. The crude ginger extract showed strong antiviral activity as reflected by its IC_50_ value calculated as 2.727 $\upmu \hbox {g}/\hbox {ml}$ whereas the HD ginger oil showed low antiviral activity as reflected by its IC_50_ value calculated as 42.8 $\upmu \hbox {g}/\hbox {ml}$ (Fig. 8).

## Discussion

Natural products are considered as major sources for drug discovery for infectious diseases and other therapeutic areas^15,79^. Many nature-based existing drugs have been clinically investigated to be used in the treatment of COVID-19 such as the anti-malaria drugs; chloroquine and hydroxychloroquine. However, it was found that treatment with chloroquine caused acute toxicity cases as reported by the United States^80^ and Nigeria^81^ media. Hydroxychloroquine showed an inhibitory effect on SARS-CoV-2 infection in vitro and less toxicity effect as well but receiving an overdose may result in poisoning^82^. Therefore, investigating other natural products that have higher efficiency and minimal side effects is needed. In this study, we conducted a computational analysis of network pharmacology, molecular docking, and molecular dynamics simulations to identify potent ginger compounds that could be capable of preventing viral replication by targeting the viral or the host proteins, followed by experimental validation to predict the antiviral activity of ginger bioactive compounds.

Analysis of the integrative network (herb-chemicals-targets-viral proteins) provides insights into the therapeutic effects of ginger compounds. Functional enrichment analysis showed that hub targets are significantly involved in (i) cellular processes such as cell cycle and cell communication, (ii) innate immune response and inflammatory response, (iii) regulation of biological processes such as RNA transcription by polymerase II, cell migration, and apoptosis, and (iv) cellular metabolic process such as respiratory electron transport chain, xenobiotic metabolic process, and lipid metabolism. Reactome pathway analysis showed significantly enriched infectious disease pathways and signaling pathways that are closely linked to modulation of the immune system such as chemokine signaling pathways, Rap1 signaling pathway, Ras signaling pathway, and MAPK signaling pathway^83^. These results suggest that ginger compounds may treat or reduce the severity of COVID-19 by protecting the host cell and enhancing the defense mechanism against viral infection.

Molecular docking was performed to assess the binding between both viral proteins and human ACE2 against the candidate chemicals. Docking results suggest (6)-gingerdiacetate, (10)-gingerol, and (8)-gingerol as promising compounds because of their high negative binding scores with all three receptors. A closer look at their interaction with the proteins’ pockets postulates that among 34 compounds, (6)-gingerdiacetate was the top hit against the three target receptors with the highest binding energy score and the best interactions. The binding of (6)-gingerdiacetate with target receptors is stabilized by different interaction types as shown in Fig. 4. To confirm the stability of the identified compound with the three target proteins a 100 ns MD simulation was performed. MD analysis confirms the very high stability of (6)-gingerdiacetate with both spike RBD and human ACE2 while it loses its stability after $\sim 50$ ns from binding with SARS-CoV-2 M^pro^.

Moreover, we conducted experimental assays for ginger analysis. GC–MS analysis of HD oil of ginger showed that sesquiterpene hydrocarbons as the predominant class ($\approx$ 43 %), followed by oxygenated monoterpenes ($\approx$ 38%). Whereas the monoterpene hydrocarbons and the oxygenated sesquiterpenes were found at much lower percentages ($\approx$ 10%, and $\approx$ 8% respectively). Concerning the predominant sesquiterpenes, $\beta$-sesquiphellandrene was the major detected compound (29.38%) agreeing with the former reports^84^. $\beta$-sesquiphellandrene is believed to be a major contributor to the anticancer properties of ginger and showed in vitro antiviral activity against rhinovirus 1B^84^. While $\alpha$-curcumene contributed to 8.12% of the volatile constituents of the HD ginger oil and is reported as an antioxidant and anti-inflammatory agent^85^. Other detected sesquiterpene hydrocarbons were $\beta$-elemene, $\gamma$-elemene, valencene, $\gamma$-muurolene, $\beta$-bisabolene, and $\alpha$-farnesene. The second abundant volatile class was the oxygenated monoterpenes accounting for almost 38% of the oil constituents. Citral isomers constituted 13.0 & 7.3% of the volatile content which is thought to be partly responsible for the antiviral activity of *Lippia citriodora* against the yellow fever virus^86^. Following was eucalyptol (1, 8 cineole) comprising 7% of the volatiles. Clinically, eucalyptol inhalation was effective in asthmatic patients through its anti-inflammatory and analgesic properties^87^ and showed antiviral activity against influenza A virus^88^. Whereas oxygenated sesquiterpenes comprised only 7.8% of the total volatiles, and were exemplified by sesquisabinene hydrate isomers, elemol, zingiberenol, and others as tabulated in Table 5. Comparably, the headspace GC-MS analysis revealed the predominance of monoterpene hydrocarbons (70.31%) followed by sesquiterpene hydrocarbon (14.31%), with the occurrence of other miscellaneous compounds such as aldehydes, ketones, and alcohols. Sabinene was the major detected compound in the fresh sample rhizomes of ginger (30.64%) followed by camphene (25.13%) then zingiberene (7.94%) which was previously reported to interact with key residues in the catalytic domain of the M^pro^ in the docking analyses^89^. While $\beta$-myrcene, D-limonene, sabinene, eucalyptol (1,8-Cineole), $\beta$-bisabolene, $\alpha$-farnesene are common in both hydro-distilled (HD) oil and fresh ginger rhizomes albeit with different percentages. Concerning the nonvolatile constituents, 6-gingerol along with 8-, and 10-gingerols were predominant metabolites, in addition to (6)-gingerdiacetate and shogaols compounds (Table 7). Considering the in vitro antiviral potential of ginger rhizomes, the hydroalcoholic extract showed better antiviral activity than the HD oil. Such higher activity of the hydroalcoholic extract could be attributed to the occurrence of (6)-gingerdiacetate.

While most of the previous studies focused on gingerols and shogaols, suggesting them as potent candidates against COVID-19^37–40,89,90^, there is limited research on other gingerols derivatives such as (6)-gingerdiacetate that exhibited a higher antioxidant effect than (6)-ginerdiol, (6)-shogaol, (6)-gingerol, and (6)-dehydrogingerdione^91^. As COVID-19 infection induces cytokine storm as an inflammatory response that causes damage to lung tissues and cell death^92^, antioxidant therapy can be a potential approach for management or treatment of COVID-19^93–95^. Our computational and experimental findings shed light on the potential effect of (6)-gingerdiacetate as a drug candidate for inhibiting SARS-CoV-2 invasion to host cells.

## Conclusions

In summary, we performed network analysis, molecular docking, and molecular dynamics simulations to predict the anti-SARS-CoV-2 activity of the common bioactive constituents of ginger. Network pharmacology revealed that ginger compounds may indirectly affect the virus survival in the host cell through multi-biological processes including cell cycle, cell communication, apoptosis, and inflammatory responses. Docking and MD simulations identified (6)-gingerdiacetate as a promising compound that binds to SARS-CoV-2 spike RBD and human ACE2 and may prevent the viral attachment to the host cell. Lastly, (6)-gingerdiacetate was detected experimentally in the ginger hydroalcoholic extract that showed higher antiviral activity than the HD ginger oil. Such higher activity of the hydroalcoholic extract could be attributed to the occurrence of (6)-gingerdiacetate. Yet further studies are warranted on the individual compounds post-isolation to validate the therapeutic effects of the candidate chemical against the pandemic.

### Supplementary Information

Supplementary Tables.

## Data Availability

All data and results supporting the conclusions of this article are included within the article and its supplementary information file.

## Abbreviations

COVID-19: Coronvirus disease 2019
SARS-CoV-2: Severe acute respiratory syndrome coronavirus 2
WHO: World Health Organization
PHEIC: Public health emergency of international concern
SRAS: Severe acute respiratory syndrome
MERS: Middle Eastern respiratory syndrome
RBD: Receptor binding domain
ACE2: Angiotensin-converting enzyme 2
${\hbox {PL}}^{\hbox {pro}}$: Papine like protease
$${\hbox {M}}^{\hbox {pro}}$$: Main protease
HRSV: Human respiratory syncytial virus
CHIKV: Chikungunya virus
IAV: Influenza A virus
Gin A: Gingerenone A
ARDS: Acute respiratory distress syndrome
AgNPs: Silver nanoparticles
Ro5: Rule of five
ADME: Absorption, distribution, metabolism, and excretion
SEA: Similarity ensemble approach
HGNC: HUGO Gene Nomenclature Committee
PPIs: Protein–protein interactions
GO: Gene ontology
FDR: False discovery rate
PDB: Protein Data Bank
MOE: Molecular Operating Environment
RMSD: Root mean square deviation
MD: Molecular dynamics
GROMACS: GROningen MAchine for Chemical Simulations
CGenFF: CHARMM General Force Field server
Rg: Radius of gyration
Grace: GRaphing and Advanced Computation and Exploration of data
CC: Cellular components
BP: Biological processes
MF: Molecular functions
NSP: Non-structural protein
