# Supplementary material for: Network pharmacology, molecular docking, and dynamics analyses to predict the antiviral activity of ginger constituents against coronavirus infection
Source: Sci Rep. 2024 May 27;14:12059. doi: 10.1038/s41598-024-60721-3 (PMC11130167; doi:10.1038/s41598-024-60721-3)
Supplement: Supplementary file 1 — Supplementary Tables. [file 41598_2024_60721_MOESM1_ESM.pdf]

Table S1: Lipinski Rule of Five (RO5) of Common phytochemicals of ginger rhizomes constituents as potential inhibitors of SARS-CoV-2

Compound	Properties					
	MW (<=500 g/mol)	HBA (<=10)	HBD (<=5)	MlogP (<=4.15)	#violations (<2)	Meet RO5 Criteria
ar-Curcumene	202.34	0	0	5.75	1	Yes
α-Zingiberene	204.35	0	0	4.53	1	Yes
β-Zingiberene	204.35	0	0	4.53	1	Yes
β-Bisabolene	204.35	0	0	4.53	1	Yes
β-Sesquiphellandrene	204.35	0	0	4.53	1	Yes
β-Elemene	204.35	0	0	4.53	1	Yes
α-Farnesene	204.35	0	0	4.84	1	Yes
γ-Selinene	204.35	0	0	4.63	1	Yes
Zerumbone	218.33	1	0	3.37	0	Yes
Zingiberenol	222.37	1	1	3.56	0	Yes
α-Pinene	136.23	0	0	4.29	1	Yes
Camphene	136.23	0	0	4.29	1	Yes
Myrcene	136.23	0	0	3.56	0	Yes
β-Phellandrene	136.23	0	0	3.27	0	Yes
Limonene	136.23	0	0	3.27	0	Yes
β-Pinene	136.23	0	0	4.29	1	Yes
Aldehyde C9	142.24	1	0	2.39	0	Yes
p-Cymene	134.22	0	0	4.47	1	Yes
Linalool	154.25	1	1	2.59	0	Yes
1-Nonanol	144.25	1	1	2.54	0	Yes
Nerolidol	222.37	1	1	3.86	0	Yes
1,8-Cineole	154.25	1	0	2.45	0	Yes
Isoborneol	154.25	1	1	2.45	0	Yes
Borneol	154.25	1	1	2.45	0	Yes
Bornyl acetate	196.29	2	0	2.76	0	Yes
α-Terpineol	154.25	1	1	2.3	0	Yes
Neral	152.23	1	0	2.49	0	Yes
Geranial	152.23	1	0	2.49	0	Yes
(6)-Gingerol	294.39	4	2	2.14	0	Yes
(8)-Gingerol	322.44	4	2	2.61	0	Yes
(10)-Gingerol	350.49	4	2	3.06	0	Yes
(6)-Shogaol	276.37	3	1	2.9	0	Yes
(8)-Shogaol	304.42	3	1	3.37	0	Yes
(6)-Gingerdiacetate	380.48	6	1	2.95	0	Yes

Table S2: List of predicted target genes of each ginger compound from three databases (SEA, SwissTargetPrediction, and TargetNet)			
**ChemicalName**	**UniprotID**	**TargetName**	**Source**
(10)-Gingerol	O00217	NDUFS8	SEA
(10)-Gingerol	O00398	P2RY10	SEA
(10)-Gingerol	O00483	NDUFA4	SEA
(10)-Gingerol	O14561	NDUFAB1	SEA
(10)-Gingerol	O15239	NDUFA1	SEA
(10)-Gingerol	O43181	NDUFS4	SEA
(10)-Gingerol	O43674	NDUFB5	SEA
(10)-Gingerol	O43676	NDUFB3	SEA
(10)-Gingerol	O43677	NDUFC1	SEA
(10)-Gingerol	O43678	NDUFA2	SEA
(10)-Gingerol	O43920	NDUFS5	SEA
(10)-Gingerol	O75251	NDUFS7	SEA
(10)-Gingerol	O75306	NDUFS2	SEA
(10)-Gingerol	O75380	NDUFS6	SEA
(10)-Gingerol	O75438	NDUFB1	SEA
(10)-Gingerol	O75489	NDUFS3	SEA
(10)-Gingerol	O75608	LYPLA1	SEA
(10)-Gingerol	O75751	SLC22A3	SEA
(10)-Gingerol	O95136	S1PR2	SEA
(10)-Gingerol	O95139	NDUFB6	SEA
(10)-Gingerol	O95167	NDUFA3	SEA
(10)-Gingerol	O95168	NDUFB4	SEA
(10)-Gingerol	O95169	NDUFB8	SEA
(10)-Gingerol	O95178	NDUFB2	SEA
(10)-Gingerol	O95182	NDUFA7	SEA
(10)-Gingerol	O95298	NDUFC2	SEA
(10)-Gingerol	O95299	NDUFA10	SEA
(10)-Gingerol	O95372	LYPLA2	SEA
(10)-Gingerol	O95977	S1PR4	SEA
(10)-Gingerol	O96000	NDUFB10	SEA
(10)-Gingerol	P01100	FOS	SEA
(10)-Gingerol	P03886	MT-ND1	SEA
(10)-Gingerol	P03891	MT-ND2	SEA
(10)-Gingerol	P03897	MT-ND3	SEA
(10)-Gingerol	P03901	MT-ND4L	SEA
(10)-Gingerol	P03905	MT-ND4	SEA
(10)-Gingerol	P03915	MT-ND5	SEA
(10)-Gingerol	P03923	MT-ND6	SEA
(10)-Gingerol	P04278	SHBG	SEA
(10)-Gingerol	P05067	APP	SEA

(10)-Gingerol	P08908	HTR1A	SEA
(10)-Gingerol	P09917	ALOX5	SEA
(10)-Gingerol	P0C869	PLA2G4B	SEA
(10)-Gingerol	P10636	MAPT	SEA
(10)-Gingerol	P10826	RARB	SEA
(10)-Gingerol	P10827	THRA	SEA
(10)-Gingerol	P10828	THRB	SEA
(10)-Gingerol	P16050	ALOX15	SEA
(10)-Gingerol	P17568	NDUFB7	SEA
(10)-Gingerol	P19404	NDUFV2	SEA
(10)-Gingerol	P19838	NFKB1	SEA
(10)-Gingerol	P21453	S1PR1	SEA
(10)-Gingerol	P21554	CNR1	SEA
(10)-Gingerol	P23280	CA6	SEA
(10)-Gingerol	P23945	FSHR	SEA
(10)-Gingerol	P28331	NDUFS1	SEA
(10)-Gingerol	P34972	CNR2	SEA
(10)-Gingerol	P35218	CA5A	SEA
(10)-Gingerol	P37058	HSD17B3	SEA
(10)-Gingerol	P39877	PLA2G5	SEA
(10)-Gingerol	P43088	PTGFR	SEA
(10)-Gingerol	P43116	PTGER2	SEA
(10)-Gingerol	P48061	CXCL12	SEA
(10)-Gingerol	P49821	NDUFV1	SEA
(10)-Gingerol	P51970	NDUFA8	SEA
(10)-Gingerol	P56181	NDUFV3	SEA
(10)-Gingerol	P56556	NDUFA6	SEA
(10)-Gingerol	P61981	YWHAG	SEA
(10)-Gingerol	Q04760	GLO1	SEA
(10)-Gingerol	Q07001	CHRND	SEA
(10)-Gingerol	Q09472	EP300	SEA
(10)-Gingerol	Q14330	GPR18	SEA
(10)-Gingerol	Q15399	TLR1	SEA
(10)-Gingerol	Q15722	LTB4R	SEA
(10)-Gingerol	Q16236	NFE2L2	SEA
(10)-Gingerol	Q16718	NDUFA5	SEA
(10)-Gingerol	Q16795	NDUFA9	SEA
(10)-Gingerol	Q16827	PTPRO	SEA
(10)-Gingerol	Q53EL6	PDCD4	SEA
(10)-Gingerol	Q5NUL3	FFAR4	SEA
(10)-Gingerol	Q7RTX1	TAS1R1	SEA
(10)-Gingerol	Q7Z2W7	TRPM8	SEA
(10)-Gingerol	Q86Y39	NDUFA11	SEA

(10)-Gingerol	Q8N183	NDUFAF2	SEA
(10)-Gingerol	Q8NER1	TRPV1	SEA
(10)-Gingerol	Q8WVY7	UBLCP1	SEA
(10)-Gingerol	Q92633	LPAR1	SEA
(10)-Gingerol	Q99500	S1PR3	SEA
(10)-Gingerol	Q9BU61	NDUFAF3	SEA
(10)-Gingerol	Q9BXC1	GPR174	SEA
(10)-Gingerol	Q9H228	S1PR5	SEA
(10)-Gingerol	Q9HBW0	LPAR2	SEA
(10)-Gingerol	Q9NP87	POLM	SEA
(10)-Gingerol	Q9NRX3	NDUFA4L2	SEA
(10)-Gingerol	Q9NX14	NDUFB11	SEA
(10)-Gingerol	Q9NYA1	SPHK1	SEA
(10)-Gingerol	Q9P032	NDUFAF4	SEA
(10)-Gingerol	Q9P0J0	NDUFA13	SEA
(10)-Gingerol	Q9UBM7	DHCR7	SEA
(10)-Gingerol	Q9UBT6	POLK	SEA
(10)-Gingerol	Q9UGP5	POLL	SEA
(10)-Gingerol	Q9UI09	NDUFA12	SEA
(10)-Gingerol	Q9Y253	POLH	SEA
(10)-Gingerol	Q9Y375	NDUFAF1	SEA
(10)-Gingerol	Q9Y6K9	IKBKG	SEA
(10)-Gingerol	Q9Y6M9	NDUFB9	SEA
(10)-Gingerol	P22303	ACHE	SwissTargetPrediction
(10)-Gingerol	P00813	ADA	SwissTargetPrediction
(10)-Gingerol	P18054	ALOX12	SwissTargetPrediction
(10)-Gingerol	P16050	ALOX15	SwissTargetPrediction
(10)-Gingerol	P09917	ALOX5	SwissTargetPrediction
(10)-Gingerol	P05067	APP	SwissTargetPrediction
(10)-Gingerol	P10275	AR	SwissTargetPrediction
(10)-Gingerol	O14965	AURKA	SwissTargetPrediction
(10)-Gingerol	Q06187	BTK	SwissTargetPrediction
(10)-Gingerol	P21730	C5AR1	SwissTargetPrediction
(10)-Gingerol	P00918	CA2	SwissTargetPrediction
(10)-Gingerol	P14635	CCNB1	SwissTargetPrediction
(10)-Gingerol	O95067	CCNB2	SwissTargetPrediction
(10)-Gingerol	Q8WWL7	CCNB3	SwissTargetPrediction
(10)-Gingerol	P24864	CCNE1	SwissTargetPrediction
(10)-Gingerol	P32246	CCR1	SwissTargetPrediction
(10)-Gingerol	P28907	CD38	SwissTargetPrediction
(10)-Gingerol	P06493	CDK1	SwissTargetPrediction
(10)-Gingerol	P24941	CDK2	SwissTargetPrediction
(10)-Gingerol	P24941	CDK2	SwissTargetPrediction

(10)-Gingerol	Q00535	CDK5	SwissTargetPrediction
(10)-Gingerol	P00746	CFD	SwissTargetPrediction
(10)-Gingerol	P21554	CNR1	SwissTargetPrediction
(10)-Gingerol	P34972	CNR2	SwissTargetPrediction
(10)-Gingerol	P49238	CX3CR1	SwissTargetPrediction
(10)-Gingerol	Q08345	DDR1	SwissTargetPrediction
(10)-Gingerol	Q16832	DDR2	SwissTargetPrediction
(10)-Gingerol	P00374	DHFR	SwissTargetPrediction
(10)-Gingerol	P42892	ECE1	SwissTargetPrediction
(10)-Gingerol	P29320	EPHA3	SwissTargetPrediction
(10)-Gingerol	P54753	EPHB3	SwissTargetPrediction
(10)-Gingerol	P04626	ERBB2	SwissTargetPrediction
(10)-Gingerol	P28715	ERCC5	SwissTargetPrediction
(10)-Gingerol	O75460	ERN1	SwissTargetPrediction
(10)-Gingerol	P03372	ESR1	SwissTargetPrediction
(10)-Gingerol	Q92731	ESR2	SwissTargetPrediction
(10)-Gingerol	Q15910	EZH2	SwissTargetPrediction
(10)-Gingerol	P39748	FEN1	SwissTargetPrediction
(10)-Gingerol	P09769	FGR	SwissTargetPrediction
(10)-Gingerol	P49354	FNTA	SwissTargetPrediction
(10)-Gingerol	P49356	FNTB	SwissTargetPrediction
(10)-Gingerol	P47869	GABRA2	SwissTargetPrediction
(10)-Gingerol	Q9GZN0	GPR88	SwissTargetPrediction
(10)-Gingerol	P41594	GRM5	SwissTargetPrediction
(10)-Gingerol	P08631	HCK	SwissTargetPrediction
(10)-Gingerol	P28845	HSD11B1	SwissTargetPrediction
(10)-Gingerol	P14061	HSD17B1	SwissTargetPrediction
(10)-Gingerol	P08908	HTR1A	SwissTargetPrediction
(10)-Gingerol	P08069	IGF1R	SwissTargetPrediction
(10)-Gingerol	P40189	IL6ST	SwissTargetPrediction
(10)-Gingerol	P06213	INSR	SwissTargetPrediction
(10)-Gingerol	Q08881	ITK	SwissTargetPrediction
(10)-Gingerol	O14649	KCNK3	SwissTargetPrediction
(10)-Gingerol	P35968	KDR	SwissTargetPrediction
(10)-Gingerol	P52732	KIF11	SwissTargetPrediction
(10)-Gingerol	Q05469	LIPE	SwissTargetPrediction
(10)-Gingerol	Q15722	LTB4R	SwissTargetPrediction
(10)-Gingerol	P07948	LYN	SwissTargetPrediction
(10)-Gingerol	O75608	LYPLA1	SwissTargetPrediction
(10)-Gingerol	O95372	LYPLA2	SwissTargetPrediction
(10)-Gingerol	Q02750	MAP2K1	SwissTargetPrediction
(10)-Gingerol	P53779	MAPK10	SwissTargetPrediction
(10)-Gingerol	P27361	MAPK3	SwissTargetPrediction

(10)-Gingerol	Q00987	MDM2	SwissTargetPrediction
(10)-Gingerol	P42345	MTOR	SwissTargetPrediction
(10)-Gingerol	P43490	NAMPT	SwissTargetPrediction
(10)-Gingerol	P49146	NPY2R	SwissTargetPrediction
(10)-Gingerol	P04150	NR3C1	SwissTargetPrediction
(10)-Gingerol	P56373	P2RX3	SwissTargetPrediction
(10)-Gingerol	P09874	PARP1	SwissTargetPrediction
(10)-Gingerol	Q9Y233	PDE10A	SwissTargetPrediction
(10)-Gingerol	O00408	PDE2A	SwissTargetPrediction
(10)-Gingerol	Q07343	PDE4B	SwissTargetPrediction
(10)-Gingerol	Q08499	PDE4D	SwissTargetPrediction
(10)-Gingerol	Q9HBH1	PDF	SwissTargetPrediction
(10)-Gingerol	P16234	PDGFRA	SwissTargetPrediction
(10)-Gingerol	P09619	PDGFRB	SwissTargetPrediction
(10)-Gingerol	Q15118	PDK1	SwissTargetPrediction
(10)-Gingerol	Q16875	PFKFB3	SwissTargetPrediction
(10)-Gingerol	P42336	PIK3CA	SwissTargetPrediction
(10)-Gingerol	P42336	PIK3CA	SwissTargetPrediction
(10)-Gingerol	P42338	PIK3CB	SwissTargetPrediction
(10)-Gingerol	O00329	PIK3CD	SwissTargetPrediction
(10)-Gingerol	P48736	PIK3CG	SwissTargetPrediction
(10)-Gingerol	P27986	PIK3R1	SwissTargetPrediction
(10)-Gingerol	Q9P1W9	PIM2	SwissTargetPrediction
(10)-Gingerol	Q03181	PPARD	SwissTargetPrediction
(10)-Gingerol	P37231	PPARG	SwissTargetPrediction
(10)-Gingerol	P42785	PRCP	SwissTargetPrediction
(10)-Gingerol	P78527	PRKDC	SwissTargetPrediction
(10)-Gingerol	O14684	PTGES	SwissTargetPrediction
(10)-Gingerol	P43088	PTGFR	SwissTargetPrediction
(10)-Gingerol	Q13464	ROCK1	SwissTargetPrediction
(10)-Gingerol	O75116	ROCK2	SwissTargetPrediction
(10)-Gingerol	P04278	SHBG	SwissTargetPrediction
(10)-Gingerol	Q01959	SLC6A3	SwissTargetPrediction
(10)-Gingerol	P12931	SRC	SwissTargetPrediction
(10)-Gingerol	P43405	SYK	SwissTargetPrediction
(10)-Gingerol	P24557	TBXAS1	SwissTargetPrediction
(10)-Gingerol	P21980	TGM2	SwissTargetPrediction
(10)-Gingerol	Q9H2K2	TNKS2	SwissTargetPrediction
(10)-Gingerol	P02766	TTR	SwissTargetPrediction
(10)-Gingerol	Q06418	TYRO3	SwissTargetPrediction
(10)-Gingerol	P40337	VHL	SwissTargetPrediction
(10)-Gingerol	P30291	WEE1	SwissTargetPrediction
(10)-Gingerol	P56704	WNT3A	SwissTargetPrediction

(10)-Gingerol	P07947	YES1	SwissTargetPrediction
(10)-Gingerol	P16050	ALOX15	TargetNet
(10)-Gingerol	P09917	ALOX5	TargetNet
(10)-Gingerol	P51452	DUSP3	TargetNet
(10)-Gingerol	O00519	FAAH	TargetNet
(10)-Gingerol	Q15722	LTB4R	TargetNet
(10)-Gingerol	P23141	CES1	TargetNet
(10)-Gingerol	P30305	CDC25B	TargetNet
(10)-Gingerol	P14555	PLA2G2A	TargetNet
(10)-Gingerol	P23219	PTGS1	TargetNet
(10)-Gingerol	P21453	S1PR1	TargetNet
(10)-Gingerol	O95136	S1PR2	TargetNet
(10)-Gingerol	Q99500	S1PR3	TargetNet
(10)-Gingerol	O95977	S1PR4	TargetNet
(10)-Gingerol	Q9H228	S1PR5	TargetNet
(10)-Gingerol	P35610	SOAT1	TargetNet
(10)-Gingerol	P10827	THRA	TargetNet
(10)-Gingerol	P10828	THRB	TargetNet
(6)-Gingerdiacetate	O00217	NDUFS8	SEA
(6)-Gingerdiacetate	O00483	NDUFA4	SEA
(6)-Gingerdiacetate	O14561	NDUFAB1	SEA
(6)-Gingerdiacetate	O15239	NDUFA1	SEA
(6)-Gingerdiacetate	O43181	NDUFS4	SEA
(6)-Gingerdiacetate	O43674	NDUFB5	SEA
(6)-Gingerdiacetate	O43676	NDUFB3	SEA
(6)-Gingerdiacetate	O43677	NDUFC1	SEA
(6)-Gingerdiacetate	O43678	NDUFA2	SEA
(6)-Gingerdiacetate	O43920	NDUFS5	SEA
(6)-Gingerdiacetate	O60218	AKR1B10	SEA
(6)-Gingerdiacetate	O60603	TLR2	SEA
(6)-Gingerdiacetate	O75251	NDUFS7	SEA
(6)-Gingerdiacetate	O75306	NDUFS2	SEA
(6)-Gingerdiacetate	O75380	NDUFS6	SEA
(6)-Gingerdiacetate	O75438	NDUFB1	SEA
(6)-Gingerdiacetate	O75489	NDUFS3	SEA
(6)-Gingerdiacetate	O75608	LYPLA1	SEA
(6)-Gingerdiacetate	O75751	SLC22A3	SEA
(6)-Gingerdiacetate	O95136	S1PR2	SEA
(6)-Gingerdiacetate	O95139	NDUFB6	SEA
(6)-Gingerdiacetate	O95167	NDUFA3	SEA
(6)-Gingerdiacetate	O95168	NDUFB4	SEA
(6)-Gingerdiacetate	O95169	NDUFB8	SEA
(6)-Gingerdiacetate	O95178	NDUFB2	SEA

(6)-Gingerdiacetate	O95182	NDUFA7	SEA
(6)-Gingerdiacetate	O95298	NDUFC2	SEA
(6)-Gingerdiacetate	O95299	NDUFA10	SEA
(6)-Gingerdiacetate	O95372	LYPLA2	SEA
(6)-Gingerdiacetate	O95977	S1PR4	SEA
(6)-Gingerdiacetate	O96000	NDUFB10	SEA
(6)-Gingerdiacetate	P01100	FOS	SEA
(6)-Gingerdiacetate	P02766	TTR	SEA
(6)-Gingerdiacetate	P03886	MT-ND1	SEA
(6)-Gingerdiacetate	P03891	MT-ND2	SEA
(6)-Gingerdiacetate	P03897	MT-ND3	SEA
(6)-Gingerdiacetate	P03901	MT-ND4L	SEA
(6)-Gingerdiacetate	P03905	MT-ND4	SEA
(6)-Gingerdiacetate	P03915	MT-ND5	SEA
(6)-Gingerdiacetate	P03923	MT-ND6	SEA
(6)-Gingerdiacetate	P04278	SHBG	SEA
(6)-Gingerdiacetate	P05067	APP	SEA
(6)-Gingerdiacetate	P09417	QDPR	SEA
(6)-Gingerdiacetate	P09917	ALOX5	SEA
(6)-Gingerdiacetate	P10636	MAPT	SEA
(6)-Gingerdiacetate	P10826	RARB	SEA
(6)-Gingerdiacetate	P11926	ODC1	SEA
(6)-Gingerdiacetate	P16050	ALOX15	SEA
(6)-Gingerdiacetate	P17568	NDUFB7	SEA
(6)-Gingerdiacetate	P19404	NDUFV2	SEA
(6)-Gingerdiacetate	P19838	NFKB1	SEA
(6)-Gingerdiacetate	P23280	CA6	SEA
(6)-Gingerdiacetate	P23945	FSHR	SEA
(6)-Gingerdiacetate	P28331	NDUFS1	SEA
(6)-Gingerdiacetate	P34972	CNR2	SEA
(6)-Gingerdiacetate	P35218	CA5A	SEA
(6)-Gingerdiacetate	P48061	CXCL12	SEA
(6)-Gingerdiacetate	P49821	NDUFV1	SEA
(6)-Gingerdiacetate	P51452	DUSP3	SEA
(6)-Gingerdiacetate	P51970	NDUFA8	SEA
(6)-Gingerdiacetate	P56181	NDUFV3	SEA
(6)-Gingerdiacetate	P56556	NDUFA6	SEA
(6)-Gingerdiacetate	P61981	YWHAG	SEA
(6)-Gingerdiacetate	Q04760	GLO1	SEA
(6)-Gingerdiacetate	Q09472	EP300	SEA
(6)-Gingerdiacetate	Q14330	GPR18	SEA
(6)-Gingerdiacetate	Q15399	TLR1	SEA
(6)-Gingerdiacetate	Q16236	NFE2L2	SEA

(6)-Gingerdiacetate	Q16718	NDUFA5	SEA
(6)-Gingerdiacetate	Q16795	NDUFA9	SEA
(6)-Gingerdiacetate	Q53EL6	PDCD4	SEA
(6)-Gingerdiacetate	Q6P179	ERAP2	SEA
(6)-Gingerdiacetate	Q7RTX1	TAS1R1	SEA
(6)-Gingerdiacetate	Q7Z2W7	TRPM8	SEA
(6)-Gingerdiacetate	Q86Y39	NDUFA11	SEA
(6)-Gingerdiacetate	Q8N183	NDUFAF2	SEA
(6)-Gingerdiacetate	Q8NER1	TRPV1	SEA
(6)-Gingerdiacetate	Q8WVY7	UBLCP1	SEA
(6)-Gingerdiacetate	Q96PF2	TSSK2	SEA
(6)-Gingerdiacetate	Q96RG2	PASK	SEA
(6)-Gingerdiacetate	Q99500	S1PR3	SEA
(6)-Gingerdiacetate	Q9BU61	NDUFAF3	SEA
(6)-Gingerdiacetate	Q9NRX3	NDUFA4L2	SEA
(6)-Gingerdiacetate	Q9NX14	NDUFB11	SEA
(6)-Gingerdiacetate	Q9NYA1	SPHK1	SEA
(6)-Gingerdiacetate	Q9P032	NDUFAF4	SEA
(6)-Gingerdiacetate	Q9P0J0	NDUFA13	SEA
(6)-Gingerdiacetate	Q9UBM7	DHCR7	SEA
(6)-Gingerdiacetate	Q9UI09	NDUFA12	SEA
(6)-Gingerdiacetate	Q9UQM7	CAMK2A	SEA
(6)-Gingerdiacetate	Q9Y2D0	CA5B	SEA
(6)-Gingerdiacetate	Q9Y375	NDUFAF1	SEA
(6)-Gingerdiacetate	Q9Y6K9	IKBKG	SEA
(6)-Gingerdiacetate	Q9Y6M9	NDUFB9	SEA
(6)-Gingerdiacetate	P00519	ABL1	SwissTargetPrediction
(6)-Gingerdiacetate	Q04771	ACVR1	SwissTargetPrediction
(6)-Gingerdiacetate	O14672	ADAM10	SwissTargetPrediction
(6)-Gingerdiacetate	Q9UNA0	ADAMTS5	SwissTargetPrediction
(6)-Gingerdiacetate	P29274	ADORA2A	SwissTargetPrediction
(6)-Gingerdiacetate	P29275	ADORA2B	SwissTargetPrediction
(6)-Gingerdiacetate	P35348	ADRA1A	SwissTargetPrediction
(6)-Gingerdiacetate	P18054	ALOX12	SwissTargetPrediction
(6)-Gingerdiacetate	P16050	ALOX15	SwissTargetPrediction
(6)-Gingerdiacetate	P09917	ALOX5	SwissTargetPrediction
(6)-Gingerdiacetate	Q96BI3	APH1A	SwissTargetPrediction
(6)-Gingerdiacetate	Q8WW43	APH1B	SwissTargetPrediction
(6)-Gingerdiacetate	P05067	APP	SwissTargetPrediction
(6)-Gingerdiacetate	O14965	AURKA	SwissTargetPrediction
(6)-Gingerdiacetate	Q96GD4	AURKB	SwissTargetPrediction
(6)-Gingerdiacetate	P37288	AVPR1A	SwissTargetPrediction
(6)-Gingerdiacetate	P13497	BMP1	SwissTargetPrediction

(6)-Gingerdiacetate	P12644	BMP4	SwissTargetPrediction
(6)-Gingerdiacetate	P15056	BRAF	SwissTargetPrediction
(6)-Gingerdiacetate	P00915	CA1	SwissTargetPrediction
(6)-Gingerdiacetate	Q8N1Q1	CA13	SwissTargetPrediction
(6)-Gingerdiacetate	P00918	CA2	SwissTargetPrediction
(6)-Gingerdiacetate	P23280	CA6	SwissTargetPrediction
(6)-Gingerdiacetate	P43166	CA7	SwissTargetPrediction
(6)-Gingerdiacetate	P07384	CAPN1	SwissTargetPrediction
(6)-Gingerdiacetate	P32239	CCKBR	SwissTargetPrediction
(6)-Gingerdiacetate	P24941	CDK2	SwissTargetPrediction
(6)-Gingerdiacetate	O14757	CHEK1	SwissTargetPrediction
(6)-Gingerdiacetate	P21554	CNR1	SwissTargetPrediction
(6)-Gingerdiacetate	P34972	CNR2	SwissTargetPrediction
(6)-Gingerdiacetate	P07711	CTSL	SwissTargetPrediction
(6)-Gingerdiacetate	P15538	CYP11B1	SwissTargetPrediction
(6)-Gingerdiacetate	P19099	CYP11B2	SwissTargetPrediction
(6)-Gingerdiacetate	P11511	CYP19A1	SwissTargetPrediction
(6)-Gingerdiacetate	P27487	DPP4	SwissTargetPrediction
(6)-Gingerdiacetate	O75460	ERN1	SwissTargetPrediction
(6)-Gingerdiacetate	P03372	ESR1	SwissTargetPrediction
(6)-Gingerdiacetate	Q92731	ESR2	SwissTargetPrediction
(6)-Gingerdiacetate	O00519	FAAH	SwissTargetPrediction
(6)-Gingerdiacetate	P17948	FLT1	SwissTargetPrediction
(6)-Gingerdiacetate	P49354	FNTA	SwissTargetPrediction
(6)-Gingerdiacetate	P49356	FNTB	SwissTargetPrediction
(6)-Gingerdiacetate	Q05586	GRIN1	SwissTargetPrediction
(6)-Gingerdiacetate	Q13224	GRIN2B	SwissTargetPrediction
(6)-Gingerdiacetate	P49840	GSK3A	SwissTargetPrediction
(6)-Gingerdiacetate	P49841	GSK3B	SwissTargetPrediction
(6)-Gingerdiacetate	Q969S8	HDAC10	SwissTargetPrediction
(6)-Gingerdiacetate	Q96DB2	HDAC11	SwissTargetPrediction
(6)-Gingerdiacetate	Q92769	HDAC2	SwissTargetPrediction
(6)-Gingerdiacetate	O15379	HDAC3	SwissTargetPrediction
(6)-Gingerdiacetate	Q9UQL6	HDAC5	SwissTargetPrediction
(6)-Gingerdiacetate	Q8WUI4	HDAC7	SwissTargetPrediction
(6)-Gingerdiacetate	Q9UKV0	HDAC9	SwissTargetPrediction
(6)-Gingerdiacetate	O60760	HPGDS	SwissTargetPrediction
(6)-Gingerdiacetate	P28845	HSD11B1	SwissTargetPrediction
(6)-Gingerdiacetate	P14061	HSD17B1	SwissTargetPrediction
(6)-Gingerdiacetate	P37059	HSD17B2	SwissTargetPrediction
(6)-Gingerdiacetate	P07900	HSP90AA1	SwissTargetPrediction
(6)-Gingerdiacetate	P08908	HTR1A	SwissTargetPrediction
(6)-Gingerdiacetate	P05362	ICAM1	SwissTargetPrediction

(6)-Gingerdiacetate	P20701	ITGAL	SwissTargetPrediction
(6)-Gingerdiacetate	P05107	ITGB2	SwissTargetPrediction
(6)-Gingerdiacetate	O60674	JAK2	SwissTargetPrediction
(6)-Gingerdiacetate	P22460	KCNA5	SwissTargetPrediction
(6)-Gingerdiacetate	O75608	LYPLA1	SwissTargetPrediction
(6)-Gingerdiacetate	O95372	LYPLA2	SwissTargetPrediction
(6)-Gingerdiacetate	Q02750	MAP2K1	SwissTargetPrediction
(6)-Gingerdiacetate	P53779	MAPK10	SwissTargetPrediction
(6)-Gingerdiacetate	P49137	MAPKAPK2	SwissTargetPrediction
(6)-Gingerdiacetate	P50281	MMP14	SwissTargetPrediction
(6)-Gingerdiacetate	P42345	MTOR	SwissTargetPrediction
(6)-Gingerdiacetate	O75376	NCOR1	SwissTargetPrediction
(6)-Gingerdiacetate	Q92542	NCSTN	SwissTargetPrediction
(6)-Gingerdiacetate	P04150	NR3C1	SwissTargetPrediction
(6)-Gingerdiacetate	P56373	P2RX3	SwissTargetPrediction
(6)-Gingerdiacetate	Q9Y233	PDE10A	SwissTargetPrediction
(6)-Gingerdiacetate	Q07343	PDE4B	SwissTargetPrediction
(6)-Gingerdiacetate	Q08499	PDE4D	SwissTargetPrediction
(6)-Gingerdiacetate	P09619	PDGFRB	SwissTargetPrediction
(6)-Gingerdiacetate	Q15118	PDK1	SwissTargetPrediction
(6)-Gingerdiacetate	Q16875	PFKFB3	SwissTargetPrediction
(6)-Gingerdiacetate	P42336	PIK3CA	SwissTargetPrediction
(6)-Gingerdiacetate	P48736	PIK3CG	SwissTargetPrediction
(6)-Gingerdiacetate	P04054	PLA2G1B	SwissTargetPrediction
(6)-Gingerdiacetate	P53350	PLK1	SwissTargetPrediction
(6)-Gingerdiacetate	P09884	POLA1	SwissTargetPrediction
(6)-Gingerdiacetate	P37231	PPARG	SwissTargetPrediction
(6)-Gingerdiacetate	P14222	PRF1	SwissTargetPrediction
(6)-Gingerdiacetate	P49768	PSEN1	SwissTargetPrediction
(6)-Gingerdiacetate	P49810	PSEN2	SwissTargetPrediction
(6)-Gingerdiacetate	Q9NZ42	PSENEN	SwissTargetPrediction
(6)-Gingerdiacetate	P43088	PTGFR	SwissTargetPrediction
(6)-Gingerdiacetate	P04049	RAF1	SwissTargetPrediction
(6)-Gingerdiacetate	P07949	RET	SwissTargetPrediction
(6)-Gingerdiacetate	P23443	RPS6KB1	SwissTargetPrediction
(6)-Gingerdiacetate	Q9UBE0	SAE1	SwissTargetPrediction
(6)-Gingerdiacetate	O00767	SCD	SwissTargetPrediction
(6)-Gingerdiacetate	P04278	SHBG	SwissTargetPrediction
(6)-Gingerdiacetate	O14975	SLC27A2	SwissTargetPrediction
(6)-Gingerdiacetate	Q99808	SLC29A1	SwissTargetPrediction
(6)-Gingerdiacetate	P23975	SLC6A2	SwissTargetPrediction
(6)-Gingerdiacetate	Q01959	SLC6A3	SwissTargetPrediction
(6)-Gingerdiacetate	Q9NYA1	SPHK1	SwissTargetPrediction

(6)-Gingerdiacetate	Q9NRA0	SPHK2	SwissTargetPrediction
(6)-Gingerdiacetate	P42226	STAT6	SwissTargetPrediction
(6)-Gingerdiacetate	P29371	TACR3	SwissTargetPrediction
(6)-Gingerdiacetate	P21731	TBXA2R	SwissTargetPrediction
(6)-Gingerdiacetate	P36897	TGFBR1	SwissTargetPrediction
(6)-Gingerdiacetate	P02766	TTR	SwissTargetPrediction
(6)-Gingerdiacetate	P30291	WEE1	SwissTargetPrediction
(6)-Gingerdiacetate	P09917	ALOX5	TargetNet
(6)-Gingerdiacetate	Q8N1Q1	CA13	TargetNet
(6)-Gingerdiacetate	Q9ULX7	CA14	TargetNet
(6)-Gingerdiacetate	P23280	CA6	TargetNet
(6)-Gingerdiacetate	P43166	CA7	TargetNet
(6)-Gingerdiacetate	P51452	DUSP3	TargetNet
(6)-Gingerdiacetate	Q04609	FOLH1	TargetNet
(6)-Gingerdiacetate	P30305	CDC25B	TargetNet
(6)-Gingerdiacetate	O95136	S1PR2	TargetNet
(6)-Gingerdiacetate	P10827	THRA	TargetNet
(6)-Gingerdiacetate	P10828	THRB	TargetNet
(6)-Gingerol	O00217	NDUFS8	SEA
(6)-Gingerol	O00398	P2RY10	SEA
(6)-Gingerol	O00483	NDUFA4	SEA
(6)-Gingerol	O14561	NDUFAB1	SEA
(6)-Gingerol	O15239	NDUFA1	SEA
(6)-Gingerol	O43181	NDUFS4	SEA
(6)-Gingerol	O43674	NDUFB5	SEA
(6)-Gingerol	O43676	NDUFB3	SEA
(6)-Gingerol	O43677	NDUFC1	SEA
(6)-Gingerol	O43678	NDUFA2	SEA
(6)-Gingerol	O43920	NDUFS5	SEA
(6)-Gingerol	O75251	NDUFS7	SEA
(6)-Gingerol	O75306	NDUFS2	SEA
(6)-Gingerol	O75380	NDUFS6	SEA
(6)-Gingerol	O75438	NDUFB1	SEA
(6)-Gingerol	O75489	NDUFS3	SEA
(6)-Gingerol	O75608	LYPLA1	SEA
(6)-Gingerol	O75751	SLC22A3	SEA
(6)-Gingerol	O95136	S1PR2	SEA
(6)-Gingerol	O95139	NDUFB6	SEA
(6)-Gingerol	O95167	NDUFA3	SEA
(6)-Gingerol	O95168	NDUFB4	SEA
(6)-Gingerol	O95169	NDUFB8	SEA
(6)-Gingerol	O95178	NDUFB2	SEA
(6)-Gingerol	O95182	NDUFA7	SEA

(6)-Gingerol	O95298	NDUFC2	SEA
(6)-Gingerol	O95299	NDUFA10	SEA
(6)-Gingerol	O95372	LYPLA2	SEA
(6)-Gingerol	O95977	S1PR4	SEA
(6)-Gingerol	O96000	NDUFB10	SEA
(6)-Gingerol	P01100	FOS	SEA
(6)-Gingerol	P03886	MT-ND1	SEA
(6)-Gingerol	P03891	MT-ND2	SEA
(6)-Gingerol	P03897	MT-ND3	SEA
(6)-Gingerol	P03901	MT-ND4L	SEA
(6)-Gingerol	P03905	MT-ND4	SEA
(6)-Gingerol	P03915	MT-ND5	SEA
(6)-Gingerol	P03923	MT-ND6	SEA
(6)-Gingerol	P04278	SHBG	SEA
(6)-Gingerol	P05067	APP	SEA
(6)-Gingerol	P09417	QDPR	SEA
(6)-Gingerol	P09917	ALOX5	SEA
(6)-Gingerol	P10636	MAPT	SEA
(6)-Gingerol	P10827	THRA	SEA
(6)-Gingerol	P10828	THRB	SEA
(6)-Gingerol	P16050	ALOX15	SEA
(6)-Gingerol	P17568	NDUFB7	SEA
(6)-Gingerol	P19404	NDUFV2	SEA
(6)-Gingerol	P19838	NFKB1	SEA
(6)-Gingerol	P23280	CA6	SEA
(6)-Gingerol	P28331	NDUFS1	SEA
(6)-Gingerol	P34972	CNR2	SEA
(6)-Gingerol	P35218	CA5A	SEA
(6)-Gingerol	P39877	PLA2G5	SEA
(6)-Gingerol	P48061	CXCL12	SEA
(6)-Gingerol	P49821	NDUFV1	SEA
(6)-Gingerol	P51970	NDUFA8	SEA
(6)-Gingerol	P56181	NDUFV3	SEA
(6)-Gingerol	P56556	NDUFA6	SEA
(6)-Gingerol	P61981	YWHAG	SEA
(6)-Gingerol	Q04760	GLO1	SEA
(6)-Gingerol	Q09472	EP300	SEA
(6)-Gingerol	Q14330	GPR18	SEA
(6)-Gingerol	Q15399	TLR1	SEA
(6)-Gingerol	Q15722	LTB4R	SEA
(6)-Gingerol	Q16236	NFE2L2	SEA
(6)-Gingerol	Q16718	NDUFA5	SEA
(6)-Gingerol	Q16795	NDUFA9	SEA

(6)-Gingerol	Q495T6	MMEL1	SEA
(6)-Gingerol	Q7RTX1	TAS1R1	SEA
(6)-Gingerol	Q7Z2W7	TRPM8	SEA
(6)-Gingerol	Q86Y39	NDUFA11	SEA
(6)-Gingerol	Q8N183	NDUFAF2	SEA
(6)-Gingerol	Q8NER1	TRPV1	SEA
(6)-Gingerol	Q8WVY7	UBLCP1	SEA
(6)-Gingerol	Q92633	LPAR1	SEA
(6)-Gingerol	Q99500	S1PR3	SEA
(6)-Gingerol	Q9BU61	NDUFAF3	SEA
(6)-Gingerol	Q9BXC1	GPR174	SEA
(6)-Gingerol	Q9NRX3	NDUFA4L2	SEA
(6)-Gingerol	Q9NX14	NDUFB11	SEA
(6)-Gingerol	Q9NYA1	SPHK1	SEA
(6)-Gingerol	Q9P032	NDUFAF4	SEA
(6)-Gingerol	Q9P0J0	NDUFA13	SEA
(6)-Gingerol	Q9UBM7	DHCR7	SEA
(6)-Gingerol	Q9UI09	NDUFA12	SEA
(6)-Gingerol	Q9Y2D0	CA5B	SEA
(6)-Gingerol	Q9Y375	NDUFAF1	SEA
(6)-Gingerol	Q9Y6K9	IKBKG	SEA
(6)-Gingerol	Q9Y6M9	NDUFB9	SEA
(6)-Gingerol	O60706	ABCC9	SwissTargetPrediction
(6)-Gingerol	P22303	ACHE	SwissTargetPrediction
(6)-Gingerol	P00813	ADA	SwissTargetPrediction
(6)-Gingerol	Q9Y243	AKT3	SwissTargetPrediction
(6)-Gingerol	Q9UM73	ALK	SwissTargetPrediction
(6)-Gingerol	P09917	ALOX5	SwissTargetPrediction
(6)-Gingerol	P10275	AR	SwissTargetPrediction
(6)-Gingerol	P00918	CA2	SwissTargetPrediction
(6)-Gingerol	P22748	CA4	SwissTargetPrediction
(6)-Gingerol	P23280	CA6	SwissTargetPrediction
(6)-Gingerol	Q14790	CASP8	SwissTargetPrediction
(6)-Gingerol	P78396	CCNA1	SwissTargetPrediction
(6)-Gingerol	P20248	CCNA2	SwissTargetPrediction
(6)-Gingerol	P14635	CCNB1	SwissTargetPrediction
(6)-Gingerol	P14635	CCNB1	SwissTargetPrediction
(6)-Gingerol	O95067	CCNB2	SwissTargetPrediction
(6)-Gingerol	Q8WWL7	CCNB3	SwissTargetPrediction
(6)-Gingerol	P24863	CCNC	SwissTargetPrediction
(6)-Gingerol	P24863	CCNC	SwissTargetPrediction
(6)-Gingerol	P24864	CCNE1	SwissTargetPrediction
(6)-Gingerol	P51946	CCNH	SwissTargetPrediction

(6)-Gingerol	O60563	CCNT1	SwissTargetPrediction
(6)-Gingerol	O60563	CCNT1	SwissTargetPrediction
(6)-Gingerol	P06493	CDK1	SwissTargetPrediction
(6)-Gingerol	P06493	CDK1	SwissTargetPrediction
(6)-Gingerol	P24941	CDK2	SwissTargetPrediction
(6)-Gingerol	P24941	CDK2	SwissTargetPrediction
(6)-Gingerol	P24941	CDK2	SwissTargetPrediction
(6)-Gingerol	Q00535	CDK5	SwissTargetPrediction
(6)-Gingerol	Q15078	CDK5R1	SwissTargetPrediction
(6)-Gingerol	P50613	CDK7	SwissTargetPrediction
(6)-Gingerol	P49336	CDK8	SwissTargetPrediction
(6)-Gingerol	P00746	CFD	SwissTargetPrediction
(6)-Gingerol	O14757	CHEK1	SwissTargetPrediction
(6)-Gingerol	P21554	CNR1	SwissTargetPrediction
(6)-Gingerol	P34972	CNR2	SwissTargetPrediction
(6)-Gingerol	P34998	CRHR1	SwissTargetPrediction
(6)-Gingerol	P49238	CX3CR1	SwissTargetPrediction
(6)-Gingerol	P25025	CXCR2	SwissTargetPrediction
(6)-Gingerol	P11511	CYP19A1	SwissTargetPrediction
(6)-Gingerol	P33316	DUT	SwissTargetPrediction
(6)-Gingerol	Q13627	DYRK1A	SwissTargetPrediction
(6)-Gingerol	P42892	ECE1	SwissTargetPrediction
(6)-Gingerol	P54760	EPHB4	SwissTargetPrediction
(6)-Gingerol	P04626	ERBB2	SwissTargetPrediction
(6)-Gingerol	O75460	ERN1	SwissTargetPrediction
(6)-Gingerol	P03372	ESR1	SwissTargetPrediction
(6)-Gingerol	P62942	FKBP1A	SwissTargetPrediction
(6)-Gingerol	O14976	GAK	SwissTargetPrediction
(6)-Gingerol	Q6DWJ6	GPR139	SwissTargetPrediction
(6)-Gingerol	P49840	GSK3A	SwissTargetPrediction
(6)-Gingerol	P49841	GSK3B	SwissTargetPrediction
(6)-Gingerol	P13807	GYS1	SwissTargetPrediction
(6)-Gingerol	O15379	HDAC3	SwissTargetPrediction
(6)-Gingerol	P04035	HMGCR	SwissTargetPrediction
(6)-Gingerol	P28845	HSD11B1	SwissTargetPrediction
(6)-Gingerol	P14061	HSD17B1	SwissTargetPrediction
(6)-Gingerol	P07900	HSP90AA1	SwissTargetPrediction
(6)-Gingerol	P08908	HTR1A	SwissTargetPrediction
(6)-Gingerol	P08069	IGF1R	SwissTargetPrediction
(6)-Gingerol	Q08881	ITK	SwissTargetPrediction
(6)-Gingerol	P23458	JAK1	SwissTargetPrediction
(6)-Gingerol	O60674	JAK2	SwissTargetPrediction
(6)-Gingerol	P52333	JAK3	SwissTargetPrediction

(6)-Gingerol	P48048	KCNJ1	SwissTargetPrediction
(6)-Gingerol	P35968	KDR	SwissTargetPrediction
(6)-Gingerol	P52732	KIF11	SwissTargetPrediction
(6)-Gingerol	P06239	LCK	SwissTargetPrediction
(6)-Gingerol	P53667	LIMK1	SwissTargetPrediction
(6)-Gingerol	Q9UIQ6	LNPEP	SwissTargetPrediction
(6)-Gingerol	Q5S007	LRRK2	SwissTargetPrediction
(6)-Gingerol	O75608	LYPLA1	SwissTargetPrediction
(6)-Gingerol	O95372	LYPLA2	SwissTargetPrediction
(6)-Gingerol	P21397	MAOA	SwissTargetPrediction
(6)-Gingerol	P27338	MAOB	SwissTargetPrediction
(6)-Gingerol	Q02750	MAP2K1	SwissTargetPrediction
(6)-Gingerol	P27361	MAPK3	SwissTargetPrediction
(6)-Gingerol	Q9HBH9	MKNK2	SwissTargetPrediction
(6)-Gingerol	P42345	MTOR	SwissTargetPrediction
(6)-Gingerol	P42345	MTOR	SwissTargetPrediction
(6)-Gingerol	Q9Y618	NCOR2	SwissTargetPrediction
(6)-Gingerol	P35228	NOS2	SwissTargetPrediction
(6)-Gingerol	P04150	NR3C1	SwissTargetPrediction
(6)-Gingerol	P04629	NTRK1	SwissTargetPrediction
(6)-Gingerol	P09874	PARP1	SwissTargetPrediction
(6)-Gingerol	Q9Y233	PDE10A	SwissTargetPrediction
(6)-Gingerol	O00408	PDE2A	SwissTargetPrediction
(6)-Gingerol	Q07343	PDE4B	SwissTargetPrediction
(6)-Gingerol	Q9HBH1	PDF	SwissTargetPrediction
(6)-Gingerol	Q15118	PDK1	SwissTargetPrediction
(6)-Gingerol	O15530	PDPK1	SwissTargetPrediction
(6)-Gingerol	O00750	PIK3C2B	SwissTargetPrediction
(6)-Gingerol	P42336	PIK3CA	SwissTargetPrediction
(6)-Gingerol	P42338	PIK3CB	SwissTargetPrediction
(6)-Gingerol	P48736	PIK3CG	SwissTargetPrediction
(6)-Gingerol	Q9P1W9	PIM2	SwissTargetPrediction
(6)-Gingerol	Q86V86	PIM3	SwissTargetPrediction
(6)-Gingerol	P09884	POLA1	SwissTargetPrediction
(6)-Gingerol	P37231	PPARG	SwissTargetPrediction
(6)-Gingerol	Q05513	PRKCZ	SwissTargetPrediction
(6)-Gingerol	P78527	PRKDC	SwissTargetPrediction
(6)-Gingerol	O14684	PTGES	SwissTargetPrediction
(6)-Gingerol	P18031	PTPN1	SwissTargetPrediction
(6)-Gingerol	P07949	RET	SwissTargetPrediction
(6)-Gingerol	P51812	RPS6KA3	SwissTargetPrediction
(6)-Gingerol	P04278	SHBG	SwissTargetPrediction
(6)-Gingerol	Q01959	SLC6A3	SwissTargetPrediction

(6)-Gingerol	P12931	SRC	SwissTargetPrediction
(6)-Gingerol	Q9H2K2	TNKS2	SwissTargetPrediction
(6)-Gingerol	Q9H4B7	TUBB1	SwissTargetPrediction
(6)-Gingerol	P29597	TYK2	SwissTargetPrediction
(6)-Gingerol	P30291	WEE1	SwissTargetPrediction
(6)-Gingerol	P56704	WNT3A	SwissTargetPrediction
(6)-Gingerol	P16050	ALOX15	TargetNet
(6)-Gingerol	P09917	ALOX5	TargetNet
(6)-Gingerol	P23280	CA6	TargetNet
(6)-Gingerol	P43166	CA7	TargetNet
(6)-Gingerol	P51452	DUSP3	TargetNet
(6)-Gingerol	P23141	CES1	TargetNet
(6)-Gingerol	P14174	MIF	TargetNet
(6)-Gingerol	P30305	CDC25B	TargetNet
(6)-Gingerol	P08183	ABCB1	TargetNet
(6)-Gingerol	P43088	PTGFR	TargetNet
(6)-Gingerol	P21453	S1PR1	TargetNet
(6)-Gingerol	O95136	S1PR2	TargetNet
(6)-Gingerol	Q9H228	S1PR5	TargetNet
(6)-Gingerol	P10827	THRA	TargetNet
(6)-Gingerol	P10828	THRB	TargetNet
(6)-Shogaol	O00217	NDUFS8	SEA
(6)-Shogaol	O00483	NDUFA4	SEA
(6)-Shogaol	O00519	FAAH	SEA
(6)-Shogaol	O14561	NDUFAB1	SEA
(6)-Shogaol	O15239	NDUFA1	SEA
(6)-Shogaol	O43181	NDUFS4	SEA
(6)-Shogaol	O43674	NDUFB5	SEA
(6)-Shogaol	O43676	NDUFB3	SEA
(6)-Shogaol	O43677	NDUFC1	SEA
(6)-Shogaol	O43678	NDUFA2	SEA
(6)-Shogaol	O43920	NDUFS5	SEA
(6)-Shogaol	O60218	AKR1B10	SEA
(6)-Shogaol	O75251	NDUFS7	SEA
(6)-Shogaol	O75306	NDUFS2	SEA
(6)-Shogaol	O75380	NDUFS6	SEA
(6)-Shogaol	O75438	NDUFB1	SEA
(6)-Shogaol	O75489	NDUFS3	SEA
(6)-Shogaol	O75608	LYPLA1	SEA
(6)-Shogaol	O75751	SLC22A3	SEA
(6)-Shogaol	O94759	TRPM2	SEA
(6)-Shogaol	O95069	KCNK2	SEA
(6)-Shogaol	O95139	NDUFB6	SEA

(6)-Shogaol	O95167	NDUFA3	SEA
(6)-Shogaol	O95168	NDUFB4	SEA
(6)-Shogaol	O95169	NDUFB8	SEA
(6)-Shogaol	O95178	NDUFB2	SEA
(6)-Shogaol	O95182	NDUFA7	SEA
(6)-Shogaol	O95298	NDUFC2	SEA
(6)-Shogaol	O95299	NDUFA10	SEA
(6)-Shogaol	O95372	LYPLA2	SEA
(6)-Shogaol	O96000	NDUFB10	SEA
(6)-Shogaol	P01100	FOS	SEA
(6)-Shogaol	P02766	TTR	SEA
(6)-Shogaol	P03886	MT-ND1	SEA
(6)-Shogaol	P03891	MT-ND2	SEA
(6)-Shogaol	P03897	MT-ND3	SEA
(6)-Shogaol	P03901	MT-ND4L	SEA
(6)-Shogaol	P03905	MT-ND4	SEA
(6)-Shogaol	P03915	MT-ND5	SEA
(6)-Shogaol	P03923	MT-ND6	SEA
(6)-Shogaol	P04278	SHBG	SEA
(6)-Shogaol	P05067	APP	SEA
(6)-Shogaol	P05412	JUN	SEA
(6)-Shogaol	P05413	FABP3	SEA
(6)-Shogaol	P06276	BCHE	SEA
(6)-Shogaol	P09417	QDPR	SEA
(6)-Shogaol	P09917	ALOX5	SEA
(6)-Shogaol	P10636	MAPT	SEA
(6)-Shogaol	P10826	RARB	SEA
(6)-Shogaol	P10827	THRA	SEA
(6)-Shogaol	P10828	THRB	SEA
(6)-Shogaol	P11388	TOP2A	SEA
(6)-Shogaol	P11926	ODC1	SEA
(6)-Shogaol	P16050	ALOX15	SEA
(6)-Shogaol	P17568	NDUFB7	SEA
(6)-Shogaol	P18054	ALOX12	SEA
(6)-Shogaol	P19404	NDUFV2	SEA
(6)-Shogaol	P19438	TNFRSF1A	SEA
(6)-Shogaol	P19838	NFKB1	SEA
(6)-Shogaol	P21554	CNR1	SEA
(6)-Shogaol	P21728	DRD1	SEA
(6)-Shogaol	P22303	ACHE	SEA
(6)-Shogaol	P23141	CES1	SEA
(6)-Shogaol	P23280	CA6	SEA
(6)-Shogaol	P23945	FSHR	SEA

(6)-Shogaol	P28331	NDUFS1	SEA
(6)-Shogaol	P34972	CNR2	SEA
(6)-Shogaol	P35218	CA5A	SEA
(6)-Shogaol	P35236	PTPN7	SEA
(6)-Shogaol	P37058	HSD17B3	SEA
(6)-Shogaol	P39877	PLA2G5	SEA
(6)-Shogaol	P40925	MDH1	SEA
(6)-Shogaol	P48061	CXCL12	SEA
(6)-Shogaol	P49821	NDUFV1	SEA
(6)-Shogaol	P51452	DUSP3	SEA
(6)-Shogaol	P51970	NDUFA8	SEA
(6)-Shogaol	P56181	NDUFV3	SEA
(6)-Shogaol	P56556	NDUFA6	SEA
(6)-Shogaol	P61981	YWHAG	SEA
(6)-Shogaol	P68402	PAFAH1B2	SEA
(6)-Shogaol	Q04760	GLO1	SEA
(6)-Shogaol	Q09472	EP300	SEA
(6)-Shogaol	Q14330	GPR18	SEA
(6)-Shogaol	Q15399	TLR1	SEA
(6)-Shogaol	Q15722	LTB4R	SEA
(6)-Shogaol	Q16236	NFE2L2	SEA
(6)-Shogaol	Q16718	NDUFA5	SEA
(6)-Shogaol	Q16795	NDUFA9	SEA
(6)-Shogaol	Q16827	PTPRO	SEA
(6)-Shogaol	Q5NUL3	FFAR4	SEA
(6)-Shogaol	Q7Z2W7	TRPM8	SEA
(6)-Shogaol	Q86Y39	NDUFA11	SEA
(6)-Shogaol	Q8N183	NDUFAF2	SEA
(6)-Shogaol	Q8NER1	TRPV1	SEA
(6)-Shogaol	Q8TDS5	OXER1	SEA
(6)-Shogaol	Q92633	LPAR1	SEA
(6)-Shogaol	Q9BU61	NDUFAF3	SEA
(6)-Shogaol	Q9BY08	EBPL	SEA
(6)-Shogaol	Q9H4B7	TUBB1	SEA
(6)-Shogaol	Q9HBW0	LPAR2	SEA
(6)-Shogaol	Q9NRX3	NDUFA4L2	SEA
(6)-Shogaol	Q9NX14	NDUFB11	SEA
(6)-Shogaol	Q9P032	NDUFAF4	SEA
(6)-Shogaol	Q9P0J0	NDUFA13	SEA
(6)-Shogaol	Q9UBM7	DHCR7	SEA
(6)-Shogaol	Q9UBT6	POLK	SEA
(6)-Shogaol	Q9UBY5	LPAR3	SEA
(6)-Shogaol	Q9UI09	NDUFA12	SEA

(6)-Shogaol	Q9Y253	POLH	SEA
(6)-Shogaol	Q9Y2D0	CA5B	SEA
(6)-Shogaol	Q9Y375	NDUFAF1	SEA
(6)-Shogaol	Q9Y6K9	IKBKG	SEA
(6)-Shogaol	Q9Y6M9	NDUFB9	SEA
(6)-Shogaol	P00519	ABL1	SwissTargetPrediction
(6)-Shogaol	P78536	ADAM17	SwissTargetPrediction
(6)-Shogaol	P25100	ADRA1D	SwissTargetPrediction
(6)-Shogaol	P15121	AKR1B1	SwissTargetPrediction
(6)-Shogaol	O60218	AKR1B10	SwissTargetPrediction
(6)-Shogaol	P05091	ALDH2	SwissTargetPrediction
(6)-Shogaol	Q9UM73	ALK	SwissTargetPrediction
(6)-Shogaol	P09917	ALOX5	SwissTargetPrediction
(6)-Shogaol	P15144	ANPEP	SwissTargetPrediction
(6)-Shogaol	P10275	AR	SwissTargetPrediction
(6)-Shogaol	P15056	BRAF	SwissTargetPrediction
(6)-Shogaol	P06493	CDK1	SwissTargetPrediction
(6)-Shogaol	P24941	CDK2	SwissTargetPrediction
(6)-Shogaol	P11802	CDK4	SwissTargetPrediction
(6)-Shogaol	Q00535	CDK5	SwissTargetPrediction
(6)-Shogaol	Q15078	CDK5R1	SwissTargetPrediction
(6)-Shogaol	O14757	CHEK1	SwissTargetPrediction
(6)-Shogaol	P36544	CHRNA7	SwissTargetPrediction
(6)-Shogaol	P49759	CLK1	SwissTargetPrediction
(6)-Shogaol	P49760	CLK2	SwissTargetPrediction
(6)-Shogaol	P49761	CLK3	SwissTargetPrediction
(6)-Shogaol	Q9HAZ1	CLK4	SwissTargetPrediction
(6)-Shogaol	P05093	CYP17A1	SwissTargetPrediction
(6)-Shogaol	P33261	CYP2C19	SwissTargetPrediction
(6)-Shogaol	P08684	CYP3A4	SwissTargetPrediction
(6)-Shogaol	Q13627	DYRK1A	SwissTargetPrediction
(6)-Shogaol	Q9Y463	DYRK1B	SwissTargetPrediction
(6)-Shogaol	P08246	ELANE	SwissTargetPrediction
(6)-Shogaol	P34913	EPHX2	SwissTargetPrediction
(6)-Shogaol	O75460	ERN1	SwissTargetPrediction
(6)-Shogaol	P03372	ESR1	SwissTargetPrediction
(6)-Shogaol	P11362	FGFR1	SwissTargetPrediction
(6)-Shogaol	Q8TDU6	GPBAR1	SwissTargetPrediction
(6)-Shogaol	P42261	GRIA1	SwissTargetPrediction
(6)-Shogaol	Q14416	GRM2	SwissTargetPrediction
(6)-Shogaol	P08631	HCK	SwissTargetPrediction
(6)-Shogaol	Q969S8	HDAC10	SwissTargetPrediction
(6)-Shogaol	Q96DB2	HDAC11	SwissTargetPrediction

(6)-Shogaol	O15379	HDAC3	SwissTargetPrediction
(6)-Shogaol	Q9UQL6	HDAC5	SwissTargetPrediction
(6)-Shogaol	Q9H3N8	HRH4	SwissTargetPrediction
(6)-Shogaol	P14061	HSD17B1	SwissTargetPrediction
(6)-Shogaol	P08908	HTR1A	SwissTargetPrediction
(6)-Shogaol	P52333	JAK3	SwissTargetPrediction
(6)-Shogaol	O75164	KDM4A	SwissTargetPrediction
(6)-Shogaol	Q9H3R0	KDM4C	SwissTargetPrediction
(6)-Shogaol	P35968	KDR	SwissTargetPrediction
(6)-Shogaol	P11150	LIPC	SwissTargetPrediction
(6)-Shogaol	Q05469	LIPE	SwissTargetPrediction
(6)-Shogaol	P06858	LPL	SwissTargetPrediction
(6)-Shogaol	P09960	LTA4H	SwissTargetPrediction
(6)-Shogaol	Q16539	MAPK14	SwissTargetPrediction
(6)-Shogaol	P14174	MIF	SwissTargetPrediction
(6)-Shogaol	P08473	MME	SwissTargetPrediction
(6)-Shogaol	P39900	MMP12	SwissTargetPrediction
(6)-Shogaol	P45452	MMP13	SwissTargetPrediction
(6)-Shogaol	P50281	MMP14	SwissTargetPrediction
(6)-Shogaol	P51512	MMP16	SwissTargetPrediction
(6)-Shogaol	P08254	MMP3	SwissTargetPrediction
(6)-Shogaol	P09237	MMP7	SwissTargetPrediction
(6)-Shogaol	P22894	MMP8	SwissTargetPrediction
(6)-Shogaol	P14780	MMP9	SwissTargetPrediction
(6)-Shogaol	Q2M385	MPEG1	SwissTargetPrediction
(6)-Shogaol	P42345	MTOR	SwissTargetPrediction
(6)-Shogaol	Q9Y618	NCOR2	SwissTargetPrediction
(6)-Shogaol	Q9Y5S8	NOX1	SwissTargetPrediction
(6)-Shogaol	Q9NPH5	NOX4	SwissTargetPrediction
(6)-Shogaol	P09874	PARP1	SwissTargetPrediction
(6)-Shogaol	Q86U86	PBRM1	SwissTargetPrediction
(6)-Shogaol	P12004	PCNA	SwissTargetPrediction
(6)-Shogaol	O00408	PDE2A	SwissTargetPrediction
(6)-Shogaol	Q14432	PDE3A	SwissTargetPrediction
(6)-Shogaol	Q13370	PDE3B	SwissTargetPrediction
(6)-Shogaol	O76074	PDE5A	SwissTargetPrediction
(6)-Shogaol	P16234	PDGFRA	SwissTargetPrediction
(6)-Shogaol	P09619	PDGFRB	SwissTargetPrediction
(6)-Shogaol	P09619	PDGFRB	SwissTargetPrediction
(6)-Shogaol	Q15118	PDK1	SwissTargetPrediction
(6)-Shogaol	Q9UBF8	PI4KB	SwissTargetPrediction
(6)-Shogaol	P42336	PIK3CA	SwissTargetPrediction
(6)-Shogaol	P42338	PIK3CB	SwissTargetPrediction

(6)-Shogaol	O00329	PIK3CD	SwissTargetPrediction
(6)-Shogaol	P48736	PIK3CG	SwissTargetPrediction
(6)-Shogaol	P11309	PIM1	SwissTargetPrediction
(6)-Shogaol	Q13093	PLA2G7	SwissTargetPrediction
(6)-Shogaol	P53350	PLK1	SwissTargetPrediction
(6)-Shogaol	P14222	PRF1	SwissTargetPrediction
(6)-Shogaol	P17252	PRKCA	SwissTargetPrediction
(6)-Shogaol	Q05513	PRKCZ	SwissTargetPrediction
(6)-Shogaol	P78527	PRKDC	SwissTargetPrediction
(6)-Shogaol	Q05397	PTK2	SwissTargetPrediction
(6)-Shogaol	O75116	ROCK2	SwissTargetPrediction
(6)-Shogaol	P51449	RORC	SwissTargetPrediction
(6)-Shogaol	P43003	SLC1A3	SwissTargetPrediction
(6)-Shogaol	Q6P1M0	SLC27A4	SwissTargetPrediction
(6)-Shogaol	P51531	SMARCA2	SwissTargetPrediction
(6)-Shogaol	P51532	SMARCA4	SwissTargetPrediction
(6)-Shogaol	P12931	SRC	SwissTargetPrediction
(6)-Shogaol	Q12772	SREBF2	SwissTargetPrediction
(6)-Shogaol	Q7Z2W7	TRPM8	SwissTargetPrediction
(6)-Shogaol	P02766	TTR	SwissTargetPrediction
(6)-Shogaol	Q9UBT2	UBA2	SwissTargetPrediction
(6)-Shogaol	P55072	VCP	SwissTargetPrediction
(6)-Shogaol	P30291	WEE1	SwissTargetPrediction
(6)-Shogaol	P47898	HTR5A	TargetNet
(6)-Shogaol	P22303	ACHE	TargetNet
(6)-Shogaol	P16050	ALOX15	TargetNet
(6)-Shogaol	P09917	ALOX5	TargetNet
(6)-Shogaol	Q9UNQ0	ABCG2	TargetNet
(6)-Shogaol	P21554	CNR1	TargetNet
(6)-Shogaol	P34972	CNR2	TargetNet
(6)-Shogaol	P34972	CNR2	TargetNet
(6)-Shogaol	Q8N1Q1	CA13	TargetNet
(6)-Shogaol	Q9ULX7	CA14	TargetNet
(6)-Shogaol	P22748	CA4	TargetNet
(6)-Shogaol	P23280	CA6	TargetNet
(6)-Shogaol	P43166	CA7	TargetNet
(6)-Shogaol	Q9GZU7	CTDSP1	TargetNet
(6)-Shogaol	P24468	NR2F2	TargetNet
(6)-Shogaol	P26358	DNMT1	TargetNet
(6)-Shogaol	P51452	DUSP3	TargetNet
(6)-Shogaol	O00519	FAAH	TargetNet
(6)-Shogaol	O00519	FAAH	TargetNet
(6)-Shogaol	O60755	GALR3	TargetNet

(6)-Shogaol	Q9HC97	GPR35	TargetNet
(6)-Shogaol	Q9UBN7	HDAC6	TargetNet
(6)-Shogaol	Q15722	LTB4R	TargetNet
(6)-Shogaol	P23141	CES1	TargetNet
(6)-Shogaol	P14174	MIF	TargetNet
(6)-Shogaol	P30305	CDC25B	TargetNet
(6)-Shogaol	P35228	NOS2	TargetNet
(6)-Shogaol	P35398	RORA	TargetNet
(6)-Shogaol	P43116	PTGER2	TargetNet
(6)-Shogaol	P43088	PTGFR	TargetNet
(6)-Shogaol	O43353	RIPK2	TargetNet
(6)-Shogaol	P21453	S1PR1	TargetNet
(6)-Shogaol	O95136	S1PR2	TargetNet
(6)-Shogaol	O95977	S1PR4	TargetNet
(6)-Shogaol	Q9H228	S1PR5	TargetNet
(6)-Shogaol	P10827	THRA	TargetNet
(6)-Shogaol	P10828	THRB	TargetNet
(6)-Shogaol	P18031	PTPN1	TargetNet
(8)-Gingerol	O00217	NDUFS8	SEA
(8)-Gingerol	O00398	P2RY10	SEA
(8)-Gingerol	O00483	NDUFA4	SEA
(8)-Gingerol	O14561	NDUFAB1	SEA
(8)-Gingerol	O15239	NDUFA1	SEA
(8)-Gingerol	O43181	NDUFS4	SEA
(8)-Gingerol	O43674	NDUFB5	SEA
(8)-Gingerol	O43676	NDUFB3	SEA
(8)-Gingerol	O43677	NDUFC1	SEA
(8)-Gingerol	O43678	NDUFA2	SEA
(8)-Gingerol	O43920	NDUFS5	SEA
(8)-Gingerol	O75251	NDUFS7	SEA
(8)-Gingerol	O75306	NDUFS2	SEA
(8)-Gingerol	O75380	NDUFS6	SEA
(8)-Gingerol	O75438	NDUFB1	SEA
(8)-Gingerol	O75489	NDUFS3	SEA
(8)-Gingerol	O75608	LYPLA1	SEA
(8)-Gingerol	O75751	SLC22A3	SEA
(8)-Gingerol	O95136	S1PR2	SEA
(8)-Gingerol	O95139	NDUFB6	SEA
(8)-Gingerol	O95167	NDUFA3	SEA
(8)-Gingerol	O95168	NDUFB4	SEA
(8)-Gingerol	O95169	NDUFB8	SEA
(8)-Gingerol	O95178	NDUFB2	SEA
(8)-Gingerol	O95182	NDUFA7	SEA

(8)-Gingerol	O95298	NDUFC2	SEA
(8)-Gingerol	O95299	NDUFA10	SEA
(8)-Gingerol	O95372	LYPLA2	SEA
(8)-Gingerol	O95977	S1PR4	SEA
(8)-Gingerol	O96000	NDUFB10	SEA
(8)-Gingerol	P01100	FOS	SEA
(8)-Gingerol	P03886	MT-ND1	SEA
(8)-Gingerol	P03891	MT-ND2	SEA
(8)-Gingerol	P03897	MT-ND3	SEA
(8)-Gingerol	P03901	MT-ND4L	SEA
(8)-Gingerol	P03905	MT-ND4	SEA
(8)-Gingerol	P03915	MT-ND5	SEA
(8)-Gingerol	P03923	MT-ND6	SEA
(8)-Gingerol	P04278	SHBG	SEA
(8)-Gingerol	P05067	APP	SEA
(8)-Gingerol	P08908	HTR1A	SEA
(8)-Gingerol	P09917	ALOX5	SEA
(8)-Gingerol	P0C869	PLA2G4B	SEA
(8)-Gingerol	P10636	MAPT	SEA
(8)-Gingerol	P10826	RARB	SEA
(8)-Gingerol	P10827	THRA	SEA
(8)-Gingerol	P10828	THRB	SEA
(8)-Gingerol	P16050	ALOX15	SEA
(8)-Gingerol	P17568	NDUFB7	SEA
(8)-Gingerol	P19404	NDUFV2	SEA
(8)-Gingerol	P19838	NFKB1	SEA
(8)-Gingerol	P21453	S1PR1	SEA
(8)-Gingerol	P21554	CNR1	SEA
(8)-Gingerol	P23280	CA6	SEA
(8)-Gingerol	P23945	FSHR	SEA
(8)-Gingerol	P28331	NDUFS1	SEA
(8)-Gingerol	P34972	CNR2	SEA
(8)-Gingerol	P35218	CA5A	SEA
(8)-Gingerol	P37058	HSD17B3	SEA
(8)-Gingerol	P39877	PLA2G5	SEA
(8)-Gingerol	P43088	PTGFR	SEA
(8)-Gingerol	P43116	PTGER2	SEA
(8)-Gingerol	P48061	CXCL12	SEA
(8)-Gingerol	P49821	NDUFV1	SEA
(8)-Gingerol	P51970	NDUFA8	SEA
(8)-Gingerol	P56181	NDUFV3	SEA
(8)-Gingerol	P56556	NDUFA6	SEA
(8)-Gingerol	P61981	YWHAG	SEA

(8)-Gingerol	Q04760	GLO1	SEA
(8)-Gingerol	Q07001	CHRND	SEA
(8)-Gingerol	Q09472	EP300	SEA
(8)-Gingerol	Q14330	GPR18	SEA
(8)-Gingerol	Q15399	TLR1	SEA
(8)-Gingerol	Q15722	LTB4R	SEA
(8)-Gingerol	Q16236	NFE2L2	SEA
(8)-Gingerol	Q16718	NDUFA5	SEA
(8)-Gingerol	Q16795	NDUFA9	SEA
(8)-Gingerol	Q16827	PTPRO	SEA
(8)-Gingerol	Q53EL6	PDCD4	SEA
(8)-Gingerol	Q5NUL3	FFAR4	SEA
(8)-Gingerol	Q7RTX1	TAS1R1	SEA
(8)-Gingerol	Q7Z2W7	TRPM8	SEA
(8)-Gingerol	Q86Y39	NDUFA11	SEA
(8)-Gingerol	Q8N183	NDUFAF2	SEA
(8)-Gingerol	Q8NER1	TRPV1	SEA
(8)-Gingerol	Q8WVY7	UBLCP1	SEA
(8)-Gingerol	Q92633	LPAR1	SEA
(8)-Gingerol	Q99500	S1PR3	SEA
(8)-Gingerol	Q9BU61	NDUFAF3	SEA
(8)-Gingerol	Q9BXC1	GPR174	SEA
(8)-Gingerol	Q9H228	S1PR5	SEA
(8)-Gingerol	Q9HBW0	LPAR2	SEA
(8)-Gingerol	Q9NP87	POLM	SEA
(8)-Gingerol	Q9NRX3	NDUFA4L2	SEA
(8)-Gingerol	Q9NX14	NDUFB11	SEA
(8)-Gingerol	Q9NYA1	SPHK1	SEA
(8)-Gingerol	Q9P032	NDUFAF4	SEA
(8)-Gingerol	Q9P0J0	NDUFA13	SEA
(8)-Gingerol	Q9UBM7	DHCR7	SEA
(8)-Gingerol	Q9UBT6	POLK	SEA
(8)-Gingerol	Q9UGP5	POLL	SEA
(8)-Gingerol	Q9UI09	NDUFA12	SEA
(8)-Gingerol	Q9Y253	POLH	SEA
(8)-Gingerol	Q9Y375	NDUFAF1	SEA
(8)-Gingerol	Q9Y6K9	IKBKG	SEA
(8)-Gingerol	Q9Y6M9	NDUFB9	SEA
(8)-Gingerol	P22303	ACHE	SwissTargetPrediction
(8)-Gingerol	P29274	ADORA2A	SwissTargetPrediction
(8)-Gingerol	P18054	ALOX12	SwissTargetPrediction
(8)-Gingerol	P09917	ALOX5	SwissTargetPrediction
(8)-Gingerol	P10275	AR	SwissTargetPrediction

(8)-Gingerol	Q13535	ATR	SwissTargetPrediction
(8)-Gingerol	O14965	AURKA	SwissTargetPrediction
(8)-Gingerol	Q96GD4	AURKB	SwissTargetPrediction
(8)-Gingerol	O60885	BRD4	SwissTargetPrediction
(8)-Gingerol	P00918	CA2	SwissTargetPrediction
(8)-Gingerol	P22748	CA4	SwissTargetPrediction
(8)-Gingerol	Q16602	CALCRL	SwissTargetPrediction
(8)-Gingerol	P14635	CCNB1	SwissTargetPrediction
(8)-Gingerol	O95067	CCNB2	SwissTargetPrediction
(8)-Gingerol	Q8WWL7	CCNB3	SwissTargetPrediction
(8)-Gingerol	P32246	CCR1	SwissTargetPrediction
(8)-Gingerol	P06493	CDK1	SwissTargetPrediction
(8)-Gingerol	P24941	CDK2	SwissTargetPrediction
(8)-Gingerol	Q00535	CDK5	SwissTargetPrediction
(8)-Gingerol	Q15078	CDK5R1	SwissTargetPrediction
(8)-Gingerol	P49336	CDK8	SwissTargetPrediction
(8)-Gingerol	P49336	CDK8	SwissTargetPrediction
(8)-Gingerol	P49336	CDK8	SwissTargetPrediction
(8)-Gingerol	P00746	CFD	SwissTargetPrediction
(8)-Gingerol	O14757	CHEK1	SwissTargetPrediction
(8)-Gingerol	P21554	CNR1	SwissTargetPrediction
(8)-Gingerol	P34972	CNR2	SwissTargetPrediction
(8)-Gingerol	P07339	CTSD	SwissTargetPrediction
(8)-Gingerol	P11511	CYP19A1	SwissTargetPrediction
(8)-Gingerol	Q16850	CYP51A1	SwissTargetPrediction
(8)-Gingerol	P33316	DUT	SwissTargetPrediction
(8)-Gingerol	P42892	ECE1	SwissTargetPrediction
(8)-Gingerol	Q15370	ELOB	SwissTargetPrediction
(8)-Gingerol	Q15369	ELOC	SwissTargetPrediction
(8)-Gingerol	P04626	ERBB2	SwissTargetPrediction
(8)-Gingerol	P28715	ERCC5	SwissTargetPrediction
(8)-Gingerol	O75460	ERN1	SwissTargetPrediction
(8)-Gingerol	P03372	ESR1	SwissTargetPrediction
(8)-Gingerol	Q92731	ESR2	SwissTargetPrediction
(8)-Gingerol	Q12884	FAP	SwissTargetPrediction
(8)-Gingerol	P39748	FEN1	SwissTargetPrediction
(8)-Gingerol	P28845	HSD11B1	SwissTargetPrediction
(8)-Gingerol	P14061	HSD17B1	SwissTargetPrediction
(8)-Gingerol	P07900	HSP90AA1	SwissTargetPrediction
(8)-Gingerol	P08908	HTR1A	SwissTargetPrediction
(8)-Gingerol	P08069	IGF1R	SwissTargetPrediction
(8)-Gingerol	P40189	IL6ST	SwissTargetPrediction
(8)-Gingerol	P06213	INSR	SwissTargetPrediction

(8)-Gingerol	Q08881	ITK	SwissTargetPrediction
(8)-Gingerol	P23458	JAK1	SwissTargetPrediction
(8)-Gingerol	O60674	JAK2	SwissTargetPrediction
(8)-Gingerol	P52333	JAK3	SwissTargetPrediction
(8)-Gingerol	P48048	KCNJ1	SwissTargetPrediction
(8)-Gingerol	O14649	KCNK3	SwissTargetPrediction
(8)-Gingerol	P35968	KDR	SwissTargetPrediction
(8)-Gingerol	P52732	KIF11	SwissTargetPrediction
(8)-Gingerol	P53667	LIMK1	SwissTargetPrediction
(8)-Gingerol	O75608	LYPLA1	SwissTargetPrediction
(8)-Gingerol	O95372	LYPLA2	SwissTargetPrediction
(8)-Gingerol	P21397	MAOA	SwissTargetPrediction
(8)-Gingerol	P27338	MAOB	SwissTargetPrediction
(8)-Gingerol	Q02750	MAP2K1	SwissTargetPrediction
(8)-Gingerol	P53779	MAPK10	SwissTargetPrediction
(8)-Gingerol	P27361	MAPK3	SwissTargetPrediction
(8)-Gingerol	Q00987	MDM2	SwissTargetPrediction
(8)-Gingerol	P42345	MTOR	SwissTargetPrediction
(8)-Gingerol	P43490	NAMPT	SwissTargetPrediction
(8)-Gingerol	O75469	NR1I2	SwissTargetPrediction
(8)-Gingerol	P04150	NR3C1	SwissTargetPrediction
(8)-Gingerol	P09874	PARP1	SwissTargetPrediction
(8)-Gingerol	Q9Y233	PDE10A	SwissTargetPrediction
(8)-Gingerol	O00408	PDE2A	SwissTargetPrediction
(8)-Gingerol	Q14432	PDE3A	SwissTargetPrediction
(8)-Gingerol	Q07343	PDE4B	SwissTargetPrediction
(8)-Gingerol	Q9HBH1	PDF	SwissTargetPrediction
(8)-Gingerol	Q15118	PDK1	SwissTargetPrediction
(8)-Gingerol	O15055	PER2	SwissTargetPrediction
(8)-Gingerol	O00750	PIK3C2B	SwissTargetPrediction
(8)-Gingerol	P42336	PIK3CA	SwissTargetPrediction
(8)-Gingerol	P42336	PIK3CA	SwissTargetPrediction
(8)-Gingerol	P42338	PIK3CB	SwissTargetPrediction
(8)-Gingerol	O00329	PIK3CD	SwissTargetPrediction
(8)-Gingerol	P48736	PIK3CG	SwissTargetPrediction
(8)-Gingerol	P27986	PIK3R1	SwissTargetPrediction
(8)-Gingerol	P09884	POLA1	SwissTargetPrediction
(8)-Gingerol	P37231	PPARG	SwissTargetPrediction
(8)-Gingerol	P42785	PRCP	SwissTargetPrediction
(8)-Gingerol	P48147	PREP	SwissTargetPrediction
(8)-Gingerol	P78527	PRKDC	SwissTargetPrediction
(8)-Gingerol	O14684	PTGES	SwissTargetPrediction
(8)-Gingerol	P43088	PTGFR	SwissTargetPrediction

(8)-Gingerol	P07949	RET	SwissTargetPrediction
(8)-Gingerol	Q13464	ROCK1	SwissTargetPrediction
(8)-Gingerol	O75116	ROCK2	SwissTargetPrediction
(8)-Gingerol	P04278	SHBG	SwissTargetPrediction
(8)-Gingerol	O00400	SLC33A1	SwissTargetPrediction
(8)-Gingerol	P23975	SLC6A2	SwissTargetPrediction
(8)-Gingerol	Q01959	SLC6A3	SwissTargetPrediction
(8)-Gingerol	P12931	SRC	SwissTargetPrediction
(8)-Gingerol	P43405	SYK	SwissTargetPrediction
(8)-Gingerol	P42681	TXK	SwissTargetPrediction
(8)-Gingerol	P29597	TYK2	SwissTargetPrediction
(8)-Gingerol	Q06418	TYRO3	SwissTargetPrediction
(8)-Gingerol	P40337	VHL	SwissTargetPrediction
(8)-Gingerol	P40337	VHL	SwissTargetPrediction
(8)-Gingerol	P30291	WEE1	SwissTargetPrediction
(8)-Gingerol	P56704	WNT3A	SwissTargetPrediction
(8)-Gingerol	P07947	YES1	SwissTargetPrediction
(8)-Gingerol	P16050	ALOX15	TargetNet
(8)-Gingerol	P09917	ALOX5	TargetNet
(8)-Gingerol	P51452	DUSP3	TargetNet
(8)-Gingerol	O00519	FAAH	TargetNet
(8)-Gingerol	Q15722	LTB4R	TargetNet
(8)-Gingerol	P23141	CES1	TargetNet
(8)-Gingerol	P30305	CDC25B	TargetNet
(8)-Gingerol	P35228	NOS2	TargetNet
(8)-Gingerol	P14555	PLA2G2A	TargetNet
(8)-Gingerol	P43088	PTGFR	TargetNet
(8)-Gingerol	P23219	PTGS1	TargetNet
(8)-Gingerol	P21453	S1PR1	TargetNet
(8)-Gingerol	O95136	S1PR2	TargetNet
(8)-Gingerol	Q99500	S1PR3	TargetNet
(8)-Gingerol	O95977	S1PR4	TargetNet
(8)-Gingerol	Q9H228	S1PR5	TargetNet
(8)-Gingerol	P35610	SOAT1	TargetNet
(8)-Gingerol	P10827	THRA	TargetNet
(8)-Gingerol	P10828	THRB	TargetNet
(8)-Shogaol	O00217	NDUFS8	SEA
(8)-Shogaol	O00483	NDUFA4	SEA
(8)-Shogaol	O00519	FAAH	SEA
(8)-Shogaol	O00748	CES2	SEA
(8)-Shogaol	O14561	NDUFAB1	SEA
(8)-Shogaol	O15239	NDUFA1	SEA
(8)-Shogaol	O15269	SPTLC1	SEA

(8)-Shogaol	O15270	SPTLC2	SEA
(8)-Shogaol	O15496	PLA2G10	SEA
(8)-Shogaol	O43181	NDUFS4	SEA
(8)-Shogaol	O43674	NDUFB5	SEA
(8)-Shogaol	O43676	NDUFB3	SEA
(8)-Shogaol	O43677	NDUFC1	SEA
(8)-Shogaol	O43678	NDUFA2	SEA
(8)-Shogaol	O43920	NDUFS5	SEA
(8)-Shogaol	O60218	AKR1B10	SEA
(8)-Shogaol	O75251	NDUFS7	SEA
(8)-Shogaol	O75306	NDUFS2	SEA
(8)-Shogaol	O75380	NDUFS6	SEA
(8)-Shogaol	O75438	NDUFB1	SEA
(8)-Shogaol	O75489	NDUFS3	SEA
(8)-Shogaol	O75608	LYPLA1	SEA
(8)-Shogaol	O75751	SLC22A3	SEA
(8)-Shogaol	O94759	TRPM2	SEA
(8)-Shogaol	O95069	KCNK2	SEA
(8)-Shogaol	O95136	S1PR2	SEA
(8)-Shogaol	O95139	NDUFB6	SEA
(8)-Shogaol	O95167	NDUFA3	SEA
(8)-Shogaol	O95168	NDUFB4	SEA
(8)-Shogaol	O95169	NDUFB8	SEA
(8)-Shogaol	O95178	NDUFB2	SEA
(8)-Shogaol	O95182	NDUFA7	SEA
(8)-Shogaol	O95298	NDUFC2	SEA
(8)-Shogaol	O95299	NDUFA10	SEA
(8)-Shogaol	O95372	LYPLA2	SEA
(8)-Shogaol	O95977	S1PR4	SEA
(8)-Shogaol	O96000	NDUFB10	SEA
(8)-Shogaol	P01100	FOS	SEA
(8)-Shogaol	P02766	TTR	SEA
(8)-Shogaol	P03886	MT-ND1	SEA
(8)-Shogaol	P03891	MT-ND2	SEA
(8)-Shogaol	P03897	MT-ND3	SEA
(8)-Shogaol	P03901	MT-ND4L	SEA
(8)-Shogaol	P03905	MT-ND4	SEA
(8)-Shogaol	P03915	MT-ND5	SEA
(8)-Shogaol	P03923	MT-ND6	SEA
(8)-Shogaol	P04035	HMGCR	SEA
(8)-Shogaol	P04278	SHBG	SEA
(8)-Shogaol	P05067	APP	SEA
(8)-Shogaol	P05412	JUN	SEA

(8)-Shogaol	P05413	FABP3	SEA
(8)-Shogaol	P06276	BCHE	SEA
(8)-Shogaol	P06746	POLB	SEA
(8)-Shogaol	P08908	HTR1A	SEA
(8)-Shogaol	P09417	QDPR	SEA
(8)-Shogaol	P09884	POLA1	SEA
(8)-Shogaol	P09917	ALOX5	SEA
(8)-Shogaol	P0C869	PLA2G4B	SEA
(8)-Shogaol	P10636	MAPT	SEA
(8)-Shogaol	P10826	RARB	SEA
(8)-Shogaol	P10827	THRA	SEA
(8)-Shogaol	P10828	THRB	SEA
(8)-Shogaol	P11388	TOP2A	SEA
(8)-Shogaol	P11926	ODC1	SEA
(8)-Shogaol	P16050	ALOX15	SEA
(8)-Shogaol	P17568	NDUFB7	SEA
(8)-Shogaol	P18054	ALOX12	SEA
(8)-Shogaol	P19404	NDUFV2	SEA
(8)-Shogaol	P19438	TNFRSF1A	SEA
(8)-Shogaol	P19838	NFKB1	SEA
(8)-Shogaol	P21453	S1PR1	SEA
(8)-Shogaol	P21554	CNR1	SEA
(8)-Shogaol	P22303	ACHE	SEA
(8)-Shogaol	P23141	CES1	SEA
(8)-Shogaol	P23280	CA6	SEA
(8)-Shogaol	P23945	FSHR	SEA
(8)-Shogaol	P28331	NDUFS1	SEA
(8)-Shogaol	P34913	EPHX2	SEA
(8)-Shogaol	P34972	CNR2	SEA
(8)-Shogaol	P35218	CA5A	SEA
(8)-Shogaol	P35236	PTPN7	SEA
(8)-Shogaol	P35520	CBS	SEA
(8)-Shogaol	P37058	HSD17B3	SEA
(8)-Shogaol	P39877	PLA2G5	SEA
(8)-Shogaol	P40925	MDH1	SEA
(8)-Shogaol	P43657	LPAR6	SEA
(8)-Shogaol	P48061	CXCL12	SEA
(8)-Shogaol	P49821	NDUFV1	SEA
(8)-Shogaol	P51452	DUSP3	SEA
(8)-Shogaol	P51970	NDUFA8	SEA
(8)-Shogaol	P56181	NDUFV3	SEA
(8)-Shogaol	P56556	NDUFA6	SEA
(8)-Shogaol	P61981	YWHAG	SEA

(8)-Shogaol	P68402	PAFAH1B2	SEA
(8)-Shogaol	Q04760	GLO1	SEA
(8)-Shogaol	Q09472	EP300	SEA
(8)-Shogaol	Q11130	FUT7	SEA
(8)-Shogaol	Q13822	ENPP2	SEA
(8)-Shogaol	Q14330	GPR18	SEA
(8)-Shogaol	Q15399	TLR1	SEA
(8)-Shogaol	Q15722	LTB4R	SEA
(8)-Shogaol	Q16236	NFE2L2	SEA
(8)-Shogaol	Q16718	NDUFA5	SEA
(8)-Shogaol	Q16795	NDUFA9	SEA
(8)-Shogaol	Q16827	PTPRO	SEA
(8)-Shogaol	Q5NUL3	FFAR4	SEA
(8)-Shogaol	Q7Z2W7	TRPM8	SEA
(8)-Shogaol	Q86Y39	NDUFA11	SEA
(8)-Shogaol	Q8N183	NDUFAF2	SEA
(8)-Shogaol	Q8NER1	TRPV1	SEA
(8)-Shogaol	Q8TDS5	OXER1	SEA
(8)-Shogaol	Q8WVY7	UBLCP1	SEA
(8)-Shogaol	Q92633	LPAR1	SEA
(8)-Shogaol	Q99500	S1PR3	SEA
(8)-Shogaol	Q99677	LPAR4	SEA
(8)-Shogaol	Q9BU61	NDUFAF3	SEA
(8)-Shogaol	Q9H228	S1PR5	SEA
(8)-Shogaol	Q9H4B7	TUBB1	SEA
(8)-Shogaol	Q9HBW0	LPAR2	SEA
(8)-Shogaol	Q9NRX3	NDUFA4L2	SEA
(8)-Shogaol	Q9NX14	NDUFB11	SEA
(8)-Shogaol	Q9NYA1	SPHK1	SEA
(8)-Shogaol	Q9P032	NDUFAF4	SEA
(8)-Shogaol	Q9P0J0	NDUFA13	SEA
(8)-Shogaol	Q9UBM7	DHCR7	SEA
(8)-Shogaol	Q9UBT6	POLK	SEA
(8)-Shogaol	Q9UBY5	LPAR3	SEA
(8)-Shogaol	Q9UI09	NDUFA12	SEA
(8)-Shogaol	Q9Y253	POLH	SEA
(8)-Shogaol	Q9Y2D0	CA5B	SEA
(8)-Shogaol	Q9Y375	NDUFAF1	SEA
(8)-Shogaol	Q9Y4D2	DAGLA	SEA
(8)-Shogaol	Q9Y6K9	IKBKG	SEA
(8)-Shogaol	Q9Y6M9	NDUFB9	SEA
(8)-Shogaol	P00519	ABL1	SwissTargetPrediction
(8)-Shogaol	P78536	ADAM17	SwissTargetPrediction

(8)-Shogaol	P30542	ADORA1	SwissTargetPrediction
(8)-Shogaol	P29274	ADORA2A	SwissTargetPrediction
(8)-Shogaol	P29275	ADORA2B	SwissTargetPrediction
(8)-Shogaol	P15121	AKR1B1	SwissTargetPrediction
(8)-Shogaol	O60218	AKR1B10	SwissTargetPrediction
(8)-Shogaol	Q9UM73	ALK	SwissTargetPrediction
(8)-Shogaol	P16050	ALOX15	SwissTargetPrediction
(8)-Shogaol	P09917	ALOX5	SwissTargetPrediction
(8)-Shogaol	P05186	ALPL	SwissTargetPrediction
(8)-Shogaol	P10275	AR	SwissTargetPrediction
(8)-Shogaol	O14965	AURKA	SwissTargetPrediction
(8)-Shogaol	P13497	BMP1	SwissTargetPrediction
(8)-Shogaol	P15056	BRAF	SwissTargetPrediction
(8)-Shogaol	P78396	CCNA1	SwissTargetPrediction
(8)-Shogaol	P20248	CCNA2	SwissTargetPrediction
(8)-Shogaol	P14635	CCNB1	SwissTargetPrediction
(8)-Shogaol	O95067	CCNB2	SwissTargetPrediction
(8)-Shogaol	Q8WWL7	CCNB3	SwissTargetPrediction
(8)-Shogaol	P24385	CCND1	SwissTargetPrediction
(8)-Shogaol	P24864	CCNE1	SwissTargetPrediction
(8)-Shogaol	O96020	CCNE2	SwissTargetPrediction
(8)-Shogaol	P30304	CDC25A	SwissTargetPrediction
(8)-Shogaol	P06493	CDK1	SwissTargetPrediction
(8)-Shogaol	P24941	CDK2	SwissTargetPrediction
(8)-Shogaol	P24941	CDK2	SwissTargetPrediction
(8)-Shogaol	P11802	CDK4	SwissTargetPrediction
(8)-Shogaol	Q00535	CDK5	SwissTargetPrediction
(8)-Shogaol	Q15078	CDK5R1	SwissTargetPrediction
(8)-Shogaol	P43681	CHRNA4	SwissTargetPrediction
(8)-Shogaol	P17787	CHRNB2	SwissTargetPrediction
(8)-Shogaol	P50416	CPT1A	SwissTargetPrediction
(8)-Shogaol	P34998	CRHR1	SwissTargetPrediction
(8)-Shogaol	P25774	CTSS	SwissTargetPrediction
(8)-Shogaol	P00374	DHFR	SwissTargetPrediction
(8)-Shogaol	P21917	DRD4	SwissTargetPrediction
(8)-Shogaol	Q13627	DYRK1A	SwissTargetPrediction
(8)-Shogaol	Q9GZT9	EGLN1	SwissTargetPrediction
(8)-Shogaol	P08246	ELANE	SwissTargetPrediction
(8)-Shogaol	P34913	EPHX2	SwissTargetPrediction
(8)-Shogaol	P04626	ERBB2	SwissTargetPrediction
(8)-Shogaol	O75460	ERN1	SwissTargetPrediction
(8)-Shogaol	P03372	ESR1	SwissTargetPrediction
(8)-Shogaol	O00519	FAAH	SwissTargetPrediction

(8)-Shogaol	P11362	FGFR1	SwissTargetPrediction
(8)-Shogaol	P49354	FNTA	SwissTargetPrediction
(8)-Shogaol	P49356	FNTB	SwissTargetPrediction
(8)-Shogaol	Q14416	GRM2	SwissTargetPrediction
(8)-Shogaol	Q969S8	HDAC10	SwissTargetPrediction
(8)-Shogaol	Q96DB2	HDAC11	SwissTargetPrediction
(8)-Shogaol	O15379	HDAC3	SwissTargetPrediction
(8)-Shogaol	Q9H3N8	HRH4	SwissTargetPrediction
(8)-Shogaol	P14061	HSD17B1	SwissTargetPrediction
(8)-Shogaol	P08908	HTR1A	SwissTargetPrediction
(8)-Shogaol	P50406	HTR6	SwissTargetPrediction
(8)-Shogaol	O75164	KDM4A	SwissTargetPrediction
(8)-Shogaol	Q9H3R0	KDM4C	SwissTargetPrediction
(8)-Shogaol	P35968	KDR	SwissTargetPrediction
(8)-Shogaol	P00338	LDHA	SwissTargetPrediction
(8)-Shogaol	Q9Y5X9	LIPG	SwissTargetPrediction
(8)-Shogaol	P09960	LTA4H	SwissTargetPrediction
(8)-Shogaol	Q16539	MAPK14	SwissTargetPrediction
(8)-Shogaol	P27361	MAPK3	SwissTargetPrediction
(8)-Shogaol	Q14680	MELK	SwissTargetPrediction
(8)-Shogaol	Q99685	MGLL	SwissTargetPrediction
(8)-Shogaol	P14174	MIF	SwissTargetPrediction
(8)-Shogaol	P39900	MMP12	SwissTargetPrediction
(8)-Shogaol	P45452	MMP13	SwissTargetPrediction
(8)-Shogaol	P08254	MMP3	SwissTargetPrediction
(8)-Shogaol	P09237	MMP7	SwissTargetPrediction
(8)-Shogaol	P22894	MMP8	SwissTargetPrediction
(8)-Shogaol	P14780	MMP9	SwissTargetPrediction
(8)-Shogaol	Q2M385	MPEG1	SwissTargetPrediction
(8)-Shogaol	P42345	MTOR	SwissTargetPrediction
(8)-Shogaol	Q9Y618	NCOR2	SwissTargetPrediction
(8)-Shogaol	Q96PY6	NEK1	SwissTargetPrediction
(8)-Shogaol	Q9Y5S8	NOX1	SwissTargetPrediction
(8)-Shogaol	Q9NPH5	NOX4	SwissTargetPrediction
(8)-Shogaol	P20393	NR1D1	SwissTargetPrediction
(8)-Shogaol	P04150	NR3C1	SwissTargetPrediction
(8)-Shogaol	P09874	PARP1	SwissTargetPrediction
(8)-Shogaol	P12004	PCNA	SwissTargetPrediction
(8)-Shogaol	O00408	PDE2A	SwissTargetPrediction
(8)-Shogaol	Q14432	PDE3A	SwissTargetPrediction
(8)-Shogaol	Q13370	PDE3B	SwissTargetPrediction
(8)-Shogaol	O76074	PDE5A	SwissTargetPrediction
(8)-Shogaol	Q13946	PDE7A	SwissTargetPrediction

(8)-Shogaol	P16234	PDGFRA	SwissTargetPrediction
(8)-Shogaol	P09619	PDGFRB	SwissTargetPrediction
(8)-Shogaol	Q15118	PDK1	SwissTargetPrediction
(8)-Shogaol	Q16875	PFKFB3	SwissTargetPrediction
(8)-Shogaol	P42336	PIK3CA	SwissTargetPrediction
(8)-Shogaol	O00329	PIK3CD	SwissTargetPrediction
(8)-Shogaol	P48736	PIK3CG	SwissTargetPrediction
(8)-Shogaol	P11309	PIM1	SwissTargetPrediction
(8)-Shogaol	P47712	PLA2G4A	SwissTargetPrediction
(8)-Shogaol	Q15149	PLEC	SwissTargetPrediction
(8)-Shogaol	P53350	PLK1	SwissTargetPrediction
(8)-Shogaol	P28062	PSMB8	SwissTargetPrediction
(8)-Shogaol	Q05397	PTK2	SwissTargetPrediction
(8)-Shogaol	P04049	RAF1	SwissTargetPrediction
(8)-Shogaol	Q04206	RELA	SwissTargetPrediction
(8)-Shogaol	Q13464	ROCK1	SwissTargetPrediction
(8)-Shogaol	O75116	ROCK2	SwissTargetPrediction
(8)-Shogaol	O00767	SCD	SwissTargetPrediction
(8)-Shogaol	P12931	SRC	SwissTargetPrediction
(8)-Shogaol	Q12772	SREBF2	SwissTargetPrediction
(8)-Shogaol	P36897	TGFBR1	SwissTargetPrediction
(8)-Shogaol	Q7Z2W7	TRPM8	SwissTargetPrediction
(8)-Shogaol	Q8NER1	TRPV1	SwissTargetPrediction
(8)-Shogaol	P55072	VCP	SwissTargetPrediction
(8)-Shogaol	P30291	WEE1	SwissTargetPrediction
(8)-Shogaol	P22303	ACHE	TargetNet
(8)-Shogaol	P16050	ALOX15	TargetNet
(8)-Shogaol	P09917	ALOX5	TargetNet
(8)-Shogaol	P09917	ALOX5	TargetNet
(8)-Shogaol	Q9UNQ0	ABCG2	TargetNet
(8)-Shogaol	P21554	CNR1	TargetNet
(8)-Shogaol	P34972	CNR2	TargetNet
(8)-Shogaol	P34972	CNR2	TargetNet
(8)-Shogaol	Q9ULX7	CA14	TargetNet
(8)-Shogaol	P22748	CA4	TargetNet
(8)-Shogaol	P23280	CA6	TargetNet
(8)-Shogaol	P43166	CA7	TargetNet
(8)-Shogaol	P24468	NR2F2	TargetNet
(8)-Shogaol	P05177	CYP1A2	TargetNet
(8)-Shogaol	P33261	CYP2C19	TargetNet
(8)-Shogaol	P51452	DUSP3	TargetNet
(8)-Shogaol	O00519	FAAH	TargetNet
(8)-Shogaol	O00519	FAAH	TargetNet

(8)-Shogaol	O60755	GALR3	TargetNet
(8)-Shogaol	Q15722	LTB4R	TargetNet
(8)-Shogaol	P23141	CES1	TargetNet
(8)-Shogaol	P14174	MIF	TargetNet
(8)-Shogaol	Q99685	MGLL	TargetNet
(8)-Shogaol	P30305	CDC25B	TargetNet
(8)-Shogaol	P08173	CHRM4	TargetNet
(8)-Shogaol	P35228	NOS2	TargetNet
(8)-Shogaol	O43353	RIPK2	TargetNet
(8)-Shogaol	P21453	S1PR1	TargetNet
(8)-Shogaol	O95136	S1PR2	TargetNet
(8)-Shogaol	Q99500	S1PR3	TargetNet
(8)-Shogaol	O95977	S1PR4	TargetNet
(8)-Shogaol	Q9H228	S1PR5	TargetNet
(8)-Shogaol	P35610	SOAT1	TargetNet
(8)-Shogaol	P10827	THRA	TargetNet
(8)-Shogaol	P10828	THRB	TargetNet
(8)-Shogaol	P18031	PTPN1	TargetNet
(8)-Shogaol	P35916	FLT4	TargetNet
1-8-Cineole	P22303	ACHE	SwissTargetPrediction
1-8-Cineole	P35348	ADRA1A	SwissTargetPrediction
1-8-Cineole	P35368	ADRA1B	SwissTargetPrediction
1-8-Cineole	P25100	ADRA1D	SwissTargetPrediction
1-8-Cineole	P08913	ADRA2A	SwissTargetPrediction
1-8-Cineole	P18089	ADRA2B	SwissTargetPrediction
1-8-Cineole	P18825	ADRA2C	SwissTargetPrediction
1-8-Cineole	Q96BI3	APH1A	SwissTargetPrediction
1-8-Cineole	Q8WW43	APH1B	SwissTargetPrediction
1-8-Cineole	P10275	AR	SwissTargetPrediction
1-8-Cineole	P00915	CA1	SwissTargetPrediction
1-8-Cineole	O43570	CA12	SwissTargetPrediction
1-8-Cineole	P00918	CA2	SwissTargetPrediction
1-8-Cineole	P22748	CA4	SwissTargetPrediction
1-8-Cineole	Q16790	CA9	SwissTargetPrediction
1-8-Cineole	P30304	CDC25A	SwissTargetPrediction
1-8-Cineole	P30305	CDC25B	SwissTargetPrediction
1-8-Cineole	P23141	CES1	SwissTargetPrediction
1-8-Cineole	O00748	CES2	SwissTargetPrediction
1-8-Cineole	P11229	CHRM1	SwissTargetPrediction
1-8-Cineole	P08172	CHRM2	SwissTargetPrediction
1-8-Cineole	P20309	CHRM3	SwissTargetPrediction
1-8-Cineole	P08173	CHRM4	SwissTargetPrediction
1-8-Cineole	P08912	CHRM5	SwissTargetPrediction

1-8-Cineole	P21554	CNR1	SwissTargetPrediction
1-8-Cineole	P50416	CPT1A	SwissTargetPrediction
1-8-Cineole	P07858	CTSB	SwissTargetPrediction
1-8-Cineole	P43235	CTSK	SwissTargetPrediction
1-8-Cineole	P07711	CTSL	SwissTargetPrediction
1-8-Cineole	P11511	CYP19A1	SwissTargetPrediction
1-8-Cineole	Q16850	CYP51A1	SwissTargetPrediction
1-8-Cineole	Q05193	DNM1	SwissTargetPrediction
1-8-Cineole	P03372	ESR1	SwissTargetPrediction
1-8-Cineole	Q92731	ESR2	SwissTargetPrediction
1-8-Cineole	O00519	FAAH	SwissTargetPrediction
1-8-Cineole	P04062	GBA	SwissTargetPrediction
1-8-Cineole	Q8TDU6	GPBAR1	SwissTargetPrediction
1-8-Cineole	Q16665	HIF1A	SwissTargetPrediction
1-8-Cineole	P04035	HMGCR	SwissTargetPrediction
1-8-Cineole	P28845	HSD11B1	SwissTargetPrediction
1-8-Cineole	P37058	HSD17B3	SwissTargetPrediction
1-8-Cineole	P07900	HSP90AA1	SwissTargetPrediction
1-8-Cineole	Q92542	NCSTN	SwissTargetPrediction
1-8-Cineole	Q96P20	NLRP3	SwissTargetPrediction
1-8-Cineole	Q9UHC9	NPC1L1	SwissTargetPrediction
1-8-Cineole	P55055	NR1H2	SwissTargetPrediction
1-8-Cineole	Q13133	NR1H3	SwissTargetPrediction
1-8-Cineole	Q96RI1	NR1H4	SwissTargetPrediction
1-8-Cineole	Q14994	NR1I3	SwissTargetPrediction
1-8-Cineole	P41145	OPRK1	SwissTargetPrediction
1-8-Cineole	P35372	OPRM1	SwissTargetPrediction
1-8-Cineole	P19021	PAM	SwissTargetPrediction
1-8-Cineole	P09884	POLA1	SwissTargetPrediction
1-8-Cineole	O75688	PPM1B	SwissTargetPrediction
1-8-Cineole	P36873	PPP1CC	SwissTargetPrediction
1-8-Cineole	P17252	PRKCA	SwissTargetPrediction
1-8-Cineole	Q02156	PRKCE	SwissTargetPrediction
1-8-Cineole	P05129	PRKCG	SwissTargetPrediction
1-8-Cineole	P24723	PRKCH	SwissTargetPrediction
1-8-Cineole	Q04759	PRKCQ	SwissTargetPrediction
1-8-Cineole	P49768	PSEN1	SwissTargetPrediction
1-8-Cineole	P49810	PSEN2	SwissTargetPrediction
1-8-Cineole	Q9NZ42	PSENEN	SwissTargetPrediction
1-8-Cineole	P18031	PTPN1	SwissTargetPrediction
1-8-Cineole	P04278	SHBG	SwissTargetPrediction
1-8-Cineole	Q15465	SHH	SwissTargetPrediction
1-8-Cineole	Q8IXJ6	SIRT2	SwissTargetPrediction

1-8-Cineole	Q14534	SQLE	SwissTargetPrediction
1-8-Cineole	Q7Z2W7	TRPM8	SwissTargetPrediction
1-8-Cineole	P16662	UGT2B7	SwissTargetPrediction
1-8-Cineole	P11473	VDR	SwissTargetPrediction
1-8-Cineole	P28566	HTR1E	TargetNet
1-8-Cineole	P28223	HTR2A	TargetNet
1-8-Cineole	P28335	HTR2C	TargetNet
1-8-Cineole	P47898	HTR5A	TargetNet
1-8-Cineole	P22303	ACHE	TargetNet
1-8-Cineole	P22303	ACHE	TargetNet
1-8-Cineole	P05186	ALPL	TargetNet
1-8-Cineole	P08913	ADRA2A	TargetNet
1-8-Cineole	P18089	ADRA2B	TargetNet
1-8-Cineole	P18825	ADRA2C	TargetNet
1-8-Cineole	P18825	ADRA2C	TargetNet
1-8-Cineole	P21397	MAOA	TargetNet
1-8-Cineole	P27338	MAOB	TargetNet
1-8-Cineole	P10275	AR	TargetNet
1-8-Cineole	P10275	AR	TargetNet
1-8-Cineole	P16050	ALOX15	TargetNet
1-8-Cineole	P09917	ALOX5	TargetNet
1-8-Cineole	P11511	CYP19A1	TargetNet
1-8-Cineole	P35869	AHR	TargetNet
1-8-Cineole	Q16548	BCL2A1	TargetNet
1-8-Cineole	P21554	CNR1	TargetNet
1-8-Cineole	P34972	CNR2	TargetNet
1-8-Cineole	P34972	CNR2	TargetNet
1-8-Cineole	P34972	CNR2	TargetNet
1-8-Cineole	O43570	CA12	TargetNet
1-8-Cineole	Q8N1Q1	CA13	TargetNet
1-8-Cineole	Q9ULX7	CA14	TargetNet
1-8-Cineole	P22748	CA4	TargetNet
1-8-Cineole	P35218	CA5A	TargetNet
1-8-Cineole	Q9Y2D0	CA5B	TargetNet
1-8-Cineole	P23280	CA6	TargetNet
1-8-Cineole	P43166	CA7	TargetNet
1-8-Cineole	Q16790	CA9	TargetNet
1-8-Cineole	Q494W8	CHRFAM7A	TargetNet
1-8-Cineole	O00748	CES2	TargetNet
1-8-Cineole	P28845	HSD11B1	TargetNet
1-8-Cineole	P28845	HSD11B1	TargetNet
1-8-Cineole	P24468	NR2F2	TargetNet
1-8-Cineole	P05177	CYP1A2	TargetNet

1-8-Cineole	P21728	DRD1	TargetNet
1-8-Cineole	P21918	DRD5	TargetNet
1-8-Cineole	Q9UHL4	DPP7	TargetNet
1-8-Cineole	P27487	DPP4	TargetNet
1-8-Cineole	P31941	APOBEC3A	TargetNet
1-8-Cineole	Q9HC16	APOBEC3G	TargetNet
1-8-Cineole	P51452	DUSP3	TargetNet
1-8-Cineole	P03372	ESR1	TargetNet
1-8-Cineole	Q92731	ESR2	TargetNet
1-8-Cineole	P14324	FDPS	TargetNet
1-8-Cineole	Q9HC97	GPR35	TargetNet
1-8-Cineole	Q9H3N8	HRH4	TargetNet
1-8-Cineole	P56524	HDAC4	TargetNet
1-8-Cineole	Q05469	LIPE	TargetNet
1-8-Cineole	P23141	CES1	TargetNet
1-8-Cineole	P14174	MIF	TargetNet
1-8-Cineole	Q99685	MGLL	TargetNet
1-8-Cineole	P30305	CDC25B	TargetNet
1-8-Cineole	P11229	CHRM1	TargetNet
1-8-Cineole	P11229	CHRM1	TargetNet
1-8-Cineole	P08172	CHRM2	TargetNet
1-8-Cineole	P08172	CHRM2	TargetNet
1-8-Cineole	P20309	CHRM3	TargetNet
1-8-Cineole	P08173	CHRM4	TargetNet
1-8-Cineole	P08912	CHRM5	TargetNet
1-8-Cineole	P43681	CHRNA4	TargetNet
1-8-Cineole	P43681	CHRNA4	TargetNet
1-8-Cineole	P36544	CHRNA7	TargetNet
1-8-Cineole	P36544	CHRNA7	TargetNet
1-8-Cineole	P29475	NOS1	TargetNet
1-8-Cineole	P29475	NOS1	TargetNet
1-8-Cineole	P29474	NOS3	TargetNet
1-8-Cineole	P35228	NOS2	TargetNet
1-8-Cineole	P35228	NOS2	TargetNet
1-8-Cineole	P35398	RORA	TargetNet
1-8-Cineole	O60240	PLIN1	TargetNet
1-8-Cineole	Q00G26	PLIN5	TargetNet
1-8-Cineole	P11086	PNMT	TargetNet
1-8-Cineole	P06401	PGR	TargetNet
1-8-Cineole	P23219	PTGS1	TargetNet
1-8-Cineole	P35354	PTGS2	TargetNet
1-8-Cineole	O43353	RIPK2	TargetNet
1-8-Cineole	Q99720	SIGMAR1	TargetNet

1-8-Cineole	Q99720	SIGMAR1	TargetNet
1-8-Cineole	Q99720	SIGMAR1	TargetNet
1-8-Cineole	Q01959	SLC6A3	TargetNet
1-8-Cineole	Q01959	SLC6A3	TargetNet
1-8-Cineole	P23975	SLC6A2	TargetNet
1-8-Cineole	P31645	SLC6A4	TargetNet
1-8-Cineole	O95136	S1PR2	TargetNet
1-8-Cineole	Q9H228	S1PR5	TargetNet
1-8-Cineole	P05093	CYP17A1	TargetNet
1-8-Cineole	P08842	STS	TargetNet
1-8-Cineole	P37058	HSD17B3	TargetNet
1-8-Cineole	Q9NR96	TLR9	TargetNet
1-8-Cineole	Q04206	RELA	TargetNet
1-Nonanol	O00519	FAAH	SEA
1-Nonanol	O00748	CES2	SEA
1-Nonanol	O15244	SLC22A2	SEA
1-Nonanol	O15245	SLC22A1	SEA
1-Nonanol	O60603	TLR2	SEA
1-Nonanol	O95136	S1PR2	SEA
1-Nonanol	O95749	GGPS1	SEA
1-Nonanol	O95977	S1PR4	SEA
1-Nonanol	P00325	ADH1B	SEA
1-Nonanol	P00326	ADH1C	SEA
1-Nonanol	P04054	PLA2G1B	SEA
1-Nonanol	P04062	GBA	SEA
1-Nonanol	P05413	FABP3	SEA
1-Nonanol	P07099	EPHX1	SEA
1-Nonanol	P07327	ADH1A	SEA
1-Nonanol	P08754	GNAI3	SEA
1-Nonanol	P09471	GNAO1	SEA
1-Nonanol	P10826	RARB	SEA
1-Nonanol	P14324	FDPS	SEA
1-Nonanol	P14555	PLA2G2A	SEA
1-Nonanol	P16278	GLB1	SEA
1-Nonanol	P19021	PAM	SEA
1-Nonanol	P23141	CES1	SEA
1-Nonanol	P28838	LAP3	SEA
1-Nonanol	P37058	HSD17B3	SEA
1-Nonanol	P39877	PLA2G5	SEA
1-Nonanol	P40394	ADH7	SEA
1-Nonanol	P43351	RAD52	SEA
1-Nonanol	P63096	GNAI1	SEA
1-Nonanol	Q02083	NAAA	SEA

1-Nonanol	Q05193	DNM1	SEA
1-Nonanol	Q4U2R8	SLC22A6	SEA
1-Nonanol	Q8TCC7	SLC22A8	SEA
1-Nonanol	Q9HBW0	LPAR2	SEA
1-Nonanol	Q9HCG7	GBA2	SEA
1-Nonanol	Q9NYA1	SPHK1	SEA
1-Nonanol	Q9UBY5	LPAR3	SEA
1-Nonanol	Q9Y4P1	ATG4B	SEA
1-Nonanol	P00813	ADA	SwissTargetPrediction
1-Nonanol	Q96BI3	APH1A	SwissTargetPrediction
1-Nonanol	Q8WW43	APH1B	SwissTargetPrediction
1-Nonanol	P10275	AR	SwissTargetPrediction
1-Nonanol	O14965	AURKA	SwissTargetPrediction
1-Nonanol	P56817	BACE1	SwissTargetPrediction
1-Nonanol	O60885	BRD4	SwissTargetPrediction
1-Nonanol	P32247	BRS3	SwissTargetPrediction
1-Nonanol	P00915	CA1	SwissTargetPrediction
1-Nonanol	P00918	CA2	SwissTargetPrediction
1-Nonanol	P22748	CA4	SwissTargetPrediction
1-Nonanol	P35218	CA5A	SwissTargetPrediction
1-Nonanol	P14635	CCNB1	SwissTargetPrediction
1-Nonanol	O95067	CCNB2	SwissTargetPrediction
1-Nonanol	Q8WWL7	CCNB3	SwissTargetPrediction
1-Nonanol	P51681	CCR5	SwissTargetPrediction
1-Nonanol	P30304	CDC25A	SwissTargetPrediction
1-Nonanol	P30305	CDC25B	SwissTargetPrediction
1-Nonanol	P06493	CDK1	SwissTargetPrediction
1-Nonanol	P06493	CDK1	SwissTargetPrediction
1-Nonanol	P32297	CHRNA3	SwissTargetPrediction
1-Nonanol	P30926	CHRNB4	SwissTargetPrediction
1-Nonanol	P21554	CNR1	SwissTargetPrediction
1-Nonanol	P34972	CNR2	SwissTargetPrediction
1-Nonanol	P21964	COMT	SwissTargetPrediction
1-Nonanol	P15538	CYP11B1	SwissTargetPrediction
1-Nonanol	P19099	CYP11B2	SwissTargetPrediction
1-Nonanol	P05093	CYP17A1	SwissTargetPrediction
1-Nonanol	P11511	CYP19A1	SwissTargetPrediction
1-Nonanol	P27487	DPP4	SwissTargetPrediction
1-Nonanol	P25101	EDNRA	SwissTargetPrediction
1-Nonanol	P00533	EGFR	SwissTargetPrediction
1-Nonanol	P07099	EPHX1	SwissTargetPrediction
1-Nonanol	P34913	EPHX2	SwissTargetPrediction
1-Nonanol	P03372	ESR1	SwissTargetPrediction

1-Nonanol	Q92731	ESR2	SwissTargetPrediction
1-Nonanol	O00519	FAAH	SwissTargetPrediction
1-Nonanol	P12104	FABP2	SwissTargetPrediction
1-Nonanol	P05413	FABP3	SwissTargetPrediction
1-Nonanol	P15090	FABP4	SwissTargetPrediction
1-Nonanol	Q01469	FABP5	SwissTargetPrediction
1-Nonanol	O14842	FFAR1	SwissTargetPrediction
1-Nonanol	P11413	G6PD	SwissTargetPrediction
1-Nonanol	P47869	GABRA2	SwissTargetPrediction
1-Nonanol	P31644	GABRA5	SwissTargetPrediction
1-Nonanol	P47870	GABRB2	SwissTargetPrediction
1-Nonanol	P28472	GABRB3	SwissTargetPrediction
1-Nonanol	P18507	GABRG2	SwissTargetPrediction
1-Nonanol	P18507	GABRG2	SwissTargetPrediction
1-Nonanol	P47871	GCGR	SwissTargetPrediction
1-Nonanol	P15104	GLUL	SwissTargetPrediction
1-Nonanol	Q8TDU6	GPBAR1	SwissTargetPrediction
1-Nonanol	P41594	GRM5	SwissTargetPrediction
1-Nonanol	P49841	GSK3B	SwissTargetPrediction
1-Nonanol	P50747	HLCS	SwissTargetPrediction
1-Nonanol	P04035	HMGCR	SwissTargetPrediction
1-Nonanol	P28845	HSD11B1	SwissTargetPrediction
1-Nonanol	P37059	HSD17B2	SwissTargetPrediction
1-Nonanol	O60725	ICMT	SwissTargetPrediction
1-Nonanol	P14902	IDO1	SwissTargetPrediction
1-Nonanol	P23458	JAK1	SwissTargetPrediction
1-Nonanol	O60674	JAK2	SwissTargetPrediction
1-Nonanol	P52333	JAK3	SwissTargetPrediction
1-Nonanol	P22001	KCNA3	SwissTargetPrediction
1-Nonanol	Q12809	KCNH2	SwissTargetPrediction
1-Nonanol	Q16539	MAPK14	SwissTargetPrediction
1-Nonanol	Q92542	NCSTN	SwissTargetPrediction
1-Nonanol	P29475	NOS1	SwissTargetPrediction
1-Nonanol	Q9UHC9	NPC1L1	SwissTargetPrediction
1-Nonanol	Q96RI1	NR1H4	SwissTargetPrediction
1-Nonanol	Q14994	NR1I3	SwissTargetPrediction
1-Nonanol	P04150	NR3C1	SwissTargetPrediction
1-Nonanol	P08235	NR3C2	SwissTargetPrediction
1-Nonanol	O15055	PER2	SwissTargetPrediction
1-Nonanol	P06401	PGR	SwissTargetPrediction
1-Nonanol	P11309	PIM1	SwissTargetPrediction
1-Nonanol	Q86V86	PIM3	SwissTargetPrediction
1-Nonanol	P09884	POLA1	SwissTargetPrediction

1-Nonanol	Q07869	PPARA	SwissTargetPrediction
1-Nonanol	Q03181	PPARD	SwissTargetPrediction
1-Nonanol	P17252	PRKCA	SwissTargetPrediction
1-Nonanol	P05771	PRKCB	SwissTargetPrediction
1-Nonanol	Q05655	PRKCD	SwissTargetPrediction
1-Nonanol	Q02156	PRKCE	SwissTargetPrediction
1-Nonanol	P05129	PRKCG	SwissTargetPrediction
1-Nonanol	P24723	PRKCH	SwissTargetPrediction
1-Nonanol	Q04759	PRKCQ	SwissTargetPrediction
1-Nonanol	P78527	PRKDC	SwissTargetPrediction
1-Nonanol	P49768	PSEN1	SwissTargetPrediction
1-Nonanol	P49810	PSEN2	SwissTargetPrediction
1-Nonanol	Q9NZ42	PSENEN	SwissTargetPrediction
1-Nonanol	P23219	PTGS1	SwissTargetPrediction
1-Nonanol	P35354	PTGS2	SwissTargetPrediction
1-Nonanol	P06737	PYGL	SwissTargetPrediction
1-Nonanol	O95267	RASGRP1	SwissTargetPrediction
1-Nonanol	O75582	RPS6KA5	SwissTargetPrediction
1-Nonanol	P08185	SERPINA6	SwissTargetPrediction
1-Nonanol	P04278	SHBG	SwissTargetPrediction
1-Nonanol	Q15465	SHH	SwissTargetPrediction
1-Nonanol	Q99720	SIGMAR1	SwissTargetPrediction
1-Nonanol	Q4U2R8	SLC22A6	SwissTargetPrediction
1-Nonanol	P23975	SLC6A2	SwissTargetPrediction
1-Nonanol	Q01959	SLC6A3	SwissTargetPrediction
1-Nonanol	P31645	SLC6A4	SwissTargetPrediction
1-Nonanol	Q9NYA1	SPHK1	SwissTargetPrediction
1-Nonanol	P36897	TGFBR1	SwissTargetPrediction
1-Nonanol	P10828	THRB	SwissTargetPrediction
1-Nonanol	O75762	TRPA1	SwissTargetPrediction
1-Nonanol	Q7Z2W7	TRPM8	SwissTargetPrediction
1-Nonanol	Q8NER1	TRPV1	SwissTargetPrediction
1-Nonanol	P29597	TYK2	SwissTargetPrediction
1-Nonanol	P16662	UGT2B7	SwissTargetPrediction
1-Nonanol	P11473	VDR	SwissTargetPrediction
1-Nonanol	P28566	HTR1E	TargetNet
1-Nonanol	P28335	HTR2C	TargetNet
1-Nonanol	P46098	HTR3A	TargetNet
1-Nonanol	P47898	HTR5A	TargetNet
1-Nonanol	P22303	ACHE	TargetNet
1-Nonanol	P22303	ACHE	TargetNet
1-Nonanol	P05186	ALPL	TargetNet
1-Nonanol	P18089	ADRA2B	TargetNet

1-Nonanol	P18825	ADRA2C	TargetNet
1-Nonanol	P21397	MAOA	TargetNet
1-Nonanol	P27338	MAOB	TargetNet
1-Nonanol	P05067	APP	TargetNet
1-Nonanol	P10275	AR	TargetNet
1-Nonanol	P09917	ALOX5	TargetNet
1-Nonanol	P11511	CYP19A1	TargetNet
1-Nonanol	P35869	AHR	TargetNet
1-Nonanol	Q16548	BCL2A1	TargetNet
1-Nonanol	P34913	EPHX2	TargetNet
1-Nonanol	P21554	CNR1	TargetNet
1-Nonanol	P21554	CNR1	TargetNet
1-Nonanol	P34972	CNR2	TargetNet
1-Nonanol	P34972	CNR2	TargetNet
1-Nonanol	P34972	CNR2	TargetNet
1-Nonanol	P00915	CA1	TargetNet
1-Nonanol	O43570	CA12	TargetNet
1-Nonanol	Q8N1Q1	CA13	TargetNet
1-Nonanol	Q9ULX7	CA14	TargetNet
1-Nonanol	P00918	CA2	TargetNet
1-Nonanol	P22748	CA4	TargetNet
1-Nonanol	P35218	CA5A	TargetNet
1-Nonanol	Q9Y2D0	CA5B	TargetNet
1-Nonanol	P23280	CA6	TargetNet
1-Nonanol	P43166	CA7	TargetNet
1-Nonanol	Q16790	CA9	TargetNet
1-Nonanol	P68400	CSNK2A1	TargetNet
1-Nonanol	P55211	CASP9	TargetNet
1-Nonanol	O00748	CES2	TargetNet
1-Nonanol	P24468	NR2F2	TargetNet
1-Nonanol	P05177	CYP1A2	TargetNet
1-Nonanol	P21728	DRD1	TargetNet
1-Nonanol	P21918	DRD5	TargetNet
1-Nonanol	P31941	APOBEC3A	TargetNet
1-Nonanol	Q9HC16	APOBEC3G	TargetNet
1-Nonanol	P51452	DUSP3	TargetNet
1-Nonanol	P14324	FDPS	TargetNet
1-Nonanol	O00519	FAAH	TargetNet
1-Nonanol	O00519	FAAH	TargetNet
1-Nonanol	O60755	GALR3	TargetNet
1-Nonanol	Q04609	FOLH1	TargetNet
1-Nonanol	P42262	GRIA2	TargetNet
1-Nonanol	Q9HC97	GPR35	TargetNet

1-Nonanol	Q9Y5N1	HRH3	TargetNet
1-Nonanol	P56524	HDAC4	TargetNet
1-Nonanol	Q8TDS4	HCAR2	TargetNet
1-Nonanol	Q15722	LTB4R	TargetNet
1-Nonanol	P23141	CES1	TargetNet
1-Nonanol	P14174	MIF	TargetNet
1-Nonanol	Q14416	GRM2	TargetNet
1-Nonanol	Q99685	MGLL	TargetNet
1-Nonanol	P30305	CDC25B	TargetNet
1-Nonanol	P11229	CHRM1	TargetNet
1-Nonanol	P11229	CHRM1	TargetNet
1-Nonanol	P08172	CHRM2	TargetNet
1-Nonanol	P20309	CHRM3	TargetNet
1-Nonanol	P08173	CHRM4	TargetNet
1-Nonanol	P08912	CHRM5	TargetNet
1-Nonanol	Q8IXJ6	SIRT2	TargetNet
1-Nonanol	P43681	CHRNA4	TargetNet
1-Nonanol	P29475	NOS1	TargetNet
1-Nonanol	P29475	NOS1	TargetNet
1-Nonanol	P29474	NOS3	TargetNet
1-Nonanol	P35228	NOS2	TargetNet
1-Nonanol	P35228	NOS2	TargetNet
1-Nonanol	P35398	RORA	TargetNet
1-Nonanol	P11086	PNMT	TargetNet
1-Nonanol	P04054	PLA2G1B	TargetNet
1-Nonanol	P14555	PLA2G2A	TargetNet
1-Nonanol	Q9Y5X4	NR2E3	TargetNet
1-Nonanol	O14684	PTGES	TargetNet
1-Nonanol	P23219	PTGS1	TargetNet
1-Nonanol	P23219	PTGS1	TargetNet
1-Nonanol	P35354	PTGS2	TargetNet
1-Nonanol	P35354	PTGS2	TargetNet
1-Nonanol	P35354	PTGS2	TargetNet
1-Nonanol	O60725	ICMT	TargetNet
1-Nonanol	P00491	PNP	TargetNet
1-Nonanol	O43353	RIPK2	TargetNet
1-Nonanol	Q99720	SIGMAR1	TargetNet
1-Nonanol	Q01959	SLC6A3	TargetNet
1-Nonanol	P23975	SLC6A2	TargetNet
1-Nonanol	P21453	S1PR1	TargetNet
1-Nonanol	O95136	S1PR2	TargetNet
1-Nonanol	Q99500	S1PR3	TargetNet
1-Nonanol	O95977	S1PR4	TargetNet

1-Nonanol	Q9H228	S1PR5	TargetNet
1-Nonanol	P05093	CYP17A1	TargetNet
1-Nonanol	P35610	SOAT1	TargetNet
1-Nonanol	P35610	SOAT1	TargetNet
1-Nonanol	P10828	THRB	TargetNet
1-Nonanol	Q9NR96	TLR9	TargetNet
1-Nonanol	Q96RJ0	TAAR1	TargetNet
1-Nonanol	Q04206	RELA	TargetNet
Aldehyde C9	O00519	FAAH	SEA
Aldehyde C9	O00748	CES2	SEA
Aldehyde C9	O15244	SLC22A2	SEA
Aldehyde C9	O15245	SLC22A1	SEA
Aldehyde C9	O95136	S1PR2	SEA
Aldehyde C9	O95749	GGPS1	SEA
Aldehyde C9	O95977	S1PR4	SEA
Aldehyde C9	P00325	ADH1B	SEA
Aldehyde C9	P00326	ADH1C	SEA
Aldehyde C9	P05413	FABP3	SEA
Aldehyde C9	P07099	EPHX1	SEA
Aldehyde C9	P07327	ADH1A	SEA
Aldehyde C9	P08754	GNAI3	SEA
Aldehyde C9	P09471	GNAO1	SEA
Aldehyde C9	P14324	FDPS	SEA
Aldehyde C9	P19021	PAM	SEA
Aldehyde C9	P23141	CES1	SEA
Aldehyde C9	P34913	EPHX2	SEA
Aldehyde C9	P39877	PLA2G5	SEA
Aldehyde C9	P40394	ADH7	SEA
Aldehyde C9	P43657	LPAR6	SEA
Aldehyde C9	P63096	GNAI1	SEA
Aldehyde C9	P68402	PAFAH1B2	SEA
Aldehyde C9	Q05193	DNM1	SEA
Aldehyde C9	Q14330	GPR18	SEA
Aldehyde C9	Q2M385	MPEG1	SEA
Aldehyde C9	Q4U2R8	SLC22A6	SEA
Aldehyde C9	Q5R387	PLA2G2C	SEA
Aldehyde C9	Q8TCC7	SLC22A8	SEA
Aldehyde C9	Q92633	LPAR1	SEA
Aldehyde C9	Q99500	S1PR3	SEA
Aldehyde C9	Q99677	LPAR4	SEA
Aldehyde C9	Q99685	MGLL	SEA
Aldehyde C9	Q9HBW0	LPAR2	SEA
Aldehyde C9	Q9UBY5	LPAR3	SEA

Aldehyde C9	P00519	ABL1	SwissTargetPrediction
Aldehyde C9	P22303	ACHE	SwissTargetPrediction
Aldehyde C9	P07327	ADH1A	SwissTargetPrediction
Aldehyde C9	P00325	ADH1B	SwissTargetPrediction
Aldehyde C9	P00326	ADH1C	SwissTargetPrediction
Aldehyde C9	P08319	ADH4	SwissTargetPrediction
Aldehyde C9	P40394	ADH7	SwissTargetPrediction
Aldehyde C9	P15121	AKR1B1	SwissTargetPrediction
Aldehyde C9	P00352	ALDH1A1	SwissTargetPrediction
Aldehyde C9	P18054	ALOX12	SwissTargetPrediction
Aldehyde C9	P16050	ALOX15	SwissTargetPrediction
Aldehyde C9	P09917	ALOX5	SwissTargetPrediction
Aldehyde C9	P10275	AR	SwissTargetPrediction
Aldehyde C9	Q13510	ASAH1	SwissTargetPrediction
Aldehyde C9	Q96GD4	AURKB	SwissTargetPrediction
Aldehyde C9	P00915	CA1	SwissTargetPrediction
Aldehyde C9	P00918	CA2	SwissTargetPrediction
Aldehyde C9	P22748	CA4	SwissTargetPrediction
Aldehyde C9	P24385	CCND1	SwissTargetPrediction
Aldehyde C9	P41597	CCR2	SwissTargetPrediction
Aldehyde C9	P51681	CCR5	SwissTargetPrediction
Aldehyde C9	P30305	CDC25B	SwissTargetPrediction
Aldehyde C9	O00311	CDC7	SwissTargetPrediction
Aldehyde C9	P06493	CDK1	SwissTargetPrediction
Aldehyde C9	P11802	CDK4	SwissTargetPrediction
Aldehyde C9	P19835	CEL	SwissTargetPrediction
Aldehyde C9	P23141	CES1	SwissTargetPrediction
Aldehyde C9	O00748	CES2	SwissTargetPrediction
Aldehyde C9	P11597	CETP	SwissTargetPrediction
Aldehyde C9	P11229	CHRM1	SwissTargetPrediction
Aldehyde C9	P08172	CHRM2	SwissTargetPrediction
Aldehyde C9	P20309	CHRM3	SwissTargetPrediction
Aldehyde C9	P34972	CNR2	SwissTargetPrediction
Aldehyde C9	P05093	CYP17A1	SwissTargetPrediction
Aldehyde C9	P11511	CYP19A1	SwissTargetPrediction
Aldehyde C9	P33261	CYP2C19	SwissTargetPrediction
Aldehyde C9	Q9UBU7	DBF4	SwissTargetPrediction
Aldehyde C9	Q92630	DYRK2	SwissTargetPrediction
Aldehyde C9	P00533	EGFR	SwissTargetPrediction
Aldehyde C9	Q99814	EPAS1	SwissTargetPrediction
Aldehyde C9	P07099	EPHX1	SwissTargetPrediction
Aldehyde C9	P34913	EPHX2	SwissTargetPrediction
Aldehyde C9	P03372	ESR1	SwissTargetPrediction

Aldehyde C9	Q92731	ESR2	SwissTargetPrediction
Aldehyde C9	P12104	FABP2	SwissTargetPrediction
Aldehyde C9	P05413	FABP3	SwissTargetPrediction
Aldehyde C9	P15090	FABP4	SwissTargetPrediction
Aldehyde C9	Q01469	FABP5	SwissTargetPrediction
Aldehyde C9	O14842	FFAR1	SwissTargetPrediction
Aldehyde C9	Q9UBS5	GABBR1	SwissTargetPrediction
Aldehyde C9	P47869	GABRA2	SwissTargetPrediction
Aldehyde C9	P47870	GABRB2	SwissTargetPrediction
Aldehyde C9	P18507	GABRG2	SwissTargetPrediction
Aldehyde C9	Q8TDU6	GPBAR1	SwissTargetPrediction
Aldehyde C9	Q13255	GRM1	SwissTargetPrediction
Aldehyde C9	P49841	GSK3B	SwissTargetPrediction
Aldehyde C9	Q8TF76	HASPIN	SwissTargetPrediction
Aldehyde C9	P50747	HLCS	SwissTargetPrediction
Aldehyde C9	P28845	HSD11B1	SwissTargetPrediction
Aldehyde C9	P07900	HSP90AA1	SwissTargetPrediction
Aldehyde C9	P0DMV8	HSPA1A	SwissTargetPrediction
Aldehyde C9	P05362	ICAM1	SwissTargetPrediction
Aldehyde C9	O14920	IKBKB	SwissTargetPrediction
Aldehyde C9	P23458	JAK1	SwissTargetPrediction
Aldehyde C9	O60674	JAK2	SwissTargetPrediction
Aldehyde C9	P52732	KIF11	SwissTargetPrediction
Aldehyde C9	P21397	MAOA	SwissTargetPrediction
Aldehyde C9	P27338	MAOB	SwissTargetPrediction
Aldehyde C9	P48039	MTNR1A	SwissTargetPrediction
Aldehyde C9	P49286	MTNR1B	SwissTargetPrediction
Aldehyde C9	Q02083	NAAA	SwissTargetPrediction
Aldehyde C9	Q6P988	NOTUM	SwissTargetPrediction
Aldehyde C9	Q9UHC9	NPC1L1	SwissTargetPrediction
Aldehyde C9	P16083	NQO2	SwissTargetPrediction
Aldehyde C9	Q96RI1	NR1H4	SwissTargetPrediction
Aldehyde C9	Q14994	NR1I3	SwissTargetPrediction
Aldehyde C9	P04150	NR3C1	SwissTargetPrediction
Aldehyde C9	P08235	NR3C2	SwissTargetPrediction
Aldehyde C9	P22736	NR4A1	SwissTargetPrediction
Aldehyde C9	Q6QHF9	PAOX	SwissTargetPrediction
Aldehyde C9	Q9UGN5	PARP2	SwissTargetPrediction
Aldehyde C9	P06401	PGR	SwissTargetPrediction
Aldehyde C9	P11309	PIM1	SwissTargetPrediction
Aldehyde C9	Q9P1W9	PIM2	SwissTargetPrediction
Aldehyde C9	Q86V86	PIM3	SwissTargetPrediction
Aldehyde C9	P06746	POLB	SwissTargetPrediction

Aldehyde C9	Q07869	PPARA	SwissTargetPrediction
Aldehyde C9	Q03181	PPARD	SwissTargetPrediction
Aldehyde C9	Q14289	PTK2B	SwissTargetPrediction
Aldehyde C9	P17706	PTPN2	SwissTargetPrediction
Aldehyde C9	P08575	PTPRC	SwissTargetPrediction
Aldehyde C9	P10586	PTPRF	SwissTargetPrediction
Aldehyde C9	O75116	ROCK2	SwissTargetPrediction
Aldehyde C9	P16581	SELE	SwissTargetPrediction
Aldehyde C9	Q4U2R8	SLC22A6	SwissTargetPrediction
Aldehyde C9	Q9NRA0	SPHK2	SwissTargetPrediction
Aldehyde C9	P18405	SRD5A1	SwissTargetPrediction
Aldehyde C9	P31213	SRD5A2	SwissTargetPrediction
Aldehyde C9	P24557	TBXAS1	SwissTargetPrediction
Aldehyde C9	O95551	TDP2	SwissTargetPrediction
Aldehyde C9	O75762	TRPA1	SwissTargetPrediction
Aldehyde C9	P02766	TTR	SwissTargetPrediction
Aldehyde C9	P19971	TYMP	SwissTargetPrediction
Aldehyde C9	P11473	VDR	SwissTargetPrediction
Aldehyde C9	P28566	HTR1E	TargetNet
Aldehyde C9	P22303	ACHE	TargetNet
Aldehyde C9	P22303	ACHE	TargetNet
Aldehyde C9	P05186	ALPL	TargetNet
Aldehyde C9	P18089	ADRA2B	TargetNet
Aldehyde C9	P18825	ADRA2C	TargetNet
Aldehyde C9	P21397	MAOA	TargetNet
Aldehyde C9	P27338	MAOB	TargetNet
Aldehyde C9	P15144	ANPEP	TargetNet
Aldehyde C9	P09917	ALOX5	TargetNet
Aldehyde C9	P11511	CYP19A1	TargetNet
Aldehyde C9	P21554	CNR1	TargetNet
Aldehyde C9	P34972	CNR2	TargetNet
Aldehyde C9	P34972	CNR2	TargetNet
Aldehyde C9	P34972	CNR2	TargetNet
Aldehyde C9	O43570	CA12	TargetNet
Aldehyde C9	Q8N1Q1	CA13	TargetNet
Aldehyde C9	Q9ULX7	CA14	TargetNet
Aldehyde C9	P00918	CA2	TargetNet
Aldehyde C9	P22748	CA4	TargetNet
Aldehyde C9	P35218	CA5A	TargetNet
Aldehyde C9	Q9Y2D0	CA5B	TargetNet
Aldehyde C9	P23280	CA6	TargetNet
Aldehyde C9	P43166	CA7	TargetNet
Aldehyde C9	Q16790	CA9	TargetNet

Aldehyde C9	P68400	CSNK2A1	TargetNet
Aldehyde C9	P55211	CASP9	TargetNet
Aldehyde C9	P43235	CTSK	TargetNet
Aldehyde C9	O00748	CES2	TargetNet
Aldehyde C9	P24468	NR2F2	TargetNet
Aldehyde C9	P05177	CYP1A2	TargetNet
Aldehyde C9	P21918	DRD5	TargetNet
Aldehyde C9	P31941	APOBEC3A	TargetNet
Aldehyde C9	Q9HC16	APOBEC3G	TargetNet
Aldehyde C9	P51452	DUSP3	TargetNet
Aldehyde C9	P14324	FDPS	TargetNet
Aldehyde C9	O00519	FAAH	TargetNet
Aldehyde C9	O00519	FAAH	TargetNet
Aldehyde C9	O60755	GALR3	TargetNet
Aldehyde C9	Q04609	FOLH1	TargetNet
Aldehyde C9	Q9HC97	GPR35	TargetNet
Aldehyde C9	P25021	HRH2	TargetNet
Aldehyde C9	P56524	HDAC4	TargetNet
Aldehyde C9	P23141	CES1	TargetNet
Aldehyde C9	P14174	MIF	TargetNet
Aldehyde C9	Q14416	GRM2	TargetNet
Aldehyde C9	Q99685	MGLL	TargetNet
Aldehyde C9	P30305	CDC25B	TargetNet
Aldehyde C9	P11229	CHRM1	TargetNet
Aldehyde C9	P11229	CHRM1	TargetNet
Aldehyde C9	P08172	CHRM2	TargetNet
Aldehyde C9	P08172	CHRM2	TargetNet
Aldehyde C9	P20309	CHRM3	TargetNet
Aldehyde C9	P20309	CHRM3	TargetNet
Aldehyde C9	P08173	CHRM4	TargetNet
Aldehyde C9	P08912	CHRM5	TargetNet
Aldehyde C9	P29475	NOS1	TargetNet
Aldehyde C9	P29474	NOS3	TargetNet
Aldehyde C9	P35228	NOS2	TargetNet
Aldehyde C9	P35228	NOS2	TargetNet
Aldehyde C9	O60240	PLIN1	TargetNet
Aldehyde C9	Q00G26	PLIN5	TargetNet
Aldehyde C9	P04054	PLA2G1B	TargetNet
Aldehyde C9	P14555	PLA2G2A	TargetNet
Aldehyde C9	P20648	ATP4A	TargetNet
Aldehyde C9	P23219	PTGS1	TargetNet
Aldehyde C9	P23219	PTGS1	TargetNet
Aldehyde C9	P35354	PTGS2	TargetNet

Aldehyde C9	P35354	PTGS2	TargetNet
Aldehyde C9	O43353	RIPK2	TargetNet
Aldehyde C9	Q99720	SIGMAR1	TargetNet
Aldehyde C9	P21453	S1PR1	TargetNet
Aldehyde C9	O95136	S1PR2	TargetNet
Aldehyde C9	Q99500	S1PR3	TargetNet
Aldehyde C9	O95977	S1PR4	TargetNet
Aldehyde C9	Q9H228	S1PR5	TargetNet
Aldehyde C9	P35610	SOAT1	TargetNet
Aldehyde C9	P35610	SOAT1	TargetNet
Aldehyde C9	Q9NR96	TLR9	TargetNet
ar-Curcumene	P21757	MSR1	SEA
ar-Curcumene	P28566	HTR1E	TargetNet
ar-Curcumene	P18825	ADRA2C	TargetNet
ar-Curcumene	P16050	ALOX15	TargetNet
ar-Curcumene	P09917	ALOX5	TargetNet
ar-Curcumene	P09917	ALOX5	TargetNet
ar-Curcumene	P11511	CYP19A1	TargetNet
ar-Curcumene	Q16548	BCL2A1	TargetNet
ar-Curcumene	O43570	CA12	TargetNet
ar-Curcumene	P23280	CA6	TargetNet
ar-Curcumene	P55211	CASP9	TargetNet
ar-Curcumene	O00748	CES2	TargetNet
ar-Curcumene	P24468	NR2F2	TargetNet
ar-Curcumene	P10635	CYP2D6	TargetNet
ar-Curcumene	P31941	APOBEC3A	TargetNet
ar-Curcumene	Q9HC16	APOBEC3G	TargetNet
ar-Curcumene	P51452	DUSP3	TargetNet
ar-Curcumene	Q9HC97	GPR35	TargetNet
ar-Curcumene	P23141	CES1	TargetNet
ar-Curcumene	P14174	MIF	TargetNet
ar-Curcumene	P50579	METAP2	TargetNet
ar-Curcumene	P30305	CDC25B	TargetNet
ar-Curcumene	P08173	CHRM4	TargetNet
ar-Curcumene	P35228	NOS2	TargetNet
ar-Curcumene	P35398	RORA	TargetNet
ar-Curcumene	P04054	PLA2G1B	TargetNet
ar-Curcumene	P23219	PTGS1	TargetNet
ar-Curcumene	P23219	PTGS1	TargetNet
ar-Curcumene	O60725	ICMT	TargetNet
ar-Curcumene	O43353	RIPK2	TargetNet
ar-Curcumene	Q01959	SLC6A3	TargetNet
ar-Curcumene	P23975	SLC6A2	TargetNet

ar-Curcumene	P31645	SLC6A4	TargetNet
ar-Curcumene	O95136	S1PR2	TargetNet
ar-Curcumene	P05093	CYP17A1	TargetNet
ar-Curcumene	Q9NR96	TLR9	TargetNet
ar-Curcumene	Q96RJ0	TAAR1	TargetNet
ar-Curcumene	Q04206	RELA	TargetNet
ar-Curcumene	Q00975	CACNA1B	TargetNet
ar-Curcumene	O95180	CACNA1H	TargetNet
Borneol	P04278	SHBG	SEA
Borneol	P07510	CHRNG	SEA
Borneol	P11230	CHRNB1	SEA
Borneol	Q07001	CHRND	SEA
Borneol	P15309	ACPP	SwissTargetPrediction
Borneol	P29275	ADORA2B	SwissTargetPrediction
Borneol	P35348	ADRA1A	SwissTargetPrediction
Borneol	P18825	ADRA2C	SwissTargetPrediction
Borneol	O60218	AKR1B10	SwissTargetPrediction
Borneol	P09917	ALOX5	SwissTargetPrediction
Borneol	P10275	AR	SwissTargetPrediction
Borneol	P06276	BCHE	SwissTargetPrediction
Borneol	P25440	BRD2	SwissTargetPrediction
Borneol	Q15059	BRD3	SwissTargetPrediction
Borneol	O60885	BRD4	SwissTargetPrediction
Borneol	P00915	CA1	SwissTargetPrediction
Borneol	P00918	CA2	SwissTargetPrediction
Borneol	P22748	CA4	SwissTargetPrediction
Borneol	P30304	CDC25A	SwissTargetPrediction
Borneol	P30305	CDC25B	SwissTargetPrediction
Borneol	P11229	CHRM1	SwissTargetPrediction
Borneol	P20309	CHRM3	SwissTargetPrediction
Borneol	P08173	CHRM4	SwissTargetPrediction
Borneol	Q9HAZ1	CLK4	SwissTargetPrediction
Borneol	P15538	CYP11B1	SwissTargetPrediction
Borneol	P19099	CYP11B2	SwissTargetPrediction
Borneol	P11511	CYP19A1	SwissTargetPrediction
Borneol	Q16850	CYP51A1	SwissTargetPrediction
Borneol	Q02127	DHODH	SwissTargetPrediction
Borneol	P27487	DPP4	SwissTargetPrediction
Borneol	P14416	DRD2	SwissTargetPrediction
Borneol	Q9BQI3	EIF2AK1	SwissTargetPrediction
Borneol	P03372	ESR1	SwissTargetPrediction
Borneol	Q92731	ESR2	SwissTargetPrediction
Borneol	P12104	FABP2	SwissTargetPrediction

Borneol	P05413	FABP3	SwissTargetPrediction
Borneol	P15090	FABP4	SwissTargetPrediction
Borneol	Q01469	FABP5	SwissTargetPrediction
Borneol	O14842	FFAR1	SwissTargetPrediction
Borneol	P11413	G6PD	SwissTargetPrediction
Borneol	Q9UBS5	GABBR1	SwissTargetPrediction
Borneol	P47869	GABRA2	SwissTargetPrediction
Borneol	P47870	GABRB2	SwissTargetPrediction
Borneol	P18507	GABRG2	SwissTargetPrediction
Borneol	Q8TDU6	GPBAR1	SwissTargetPrediction
Borneol	P09601	HMOX1	SwissTargetPrediction
Borneol	P28845	HSD11B1	SwissTargetPrediction
Borneol	P37059	HSD17B2	SwissTargetPrediction
Borneol	P37058	HSD17B3	SwissTargetPrediction
Borneol	P28335	HTR2C	SwissTargetPrediction
Borneol	O60725	ICMT	SwissTargetPrediction
Borneol	P14902	IDO1	SwissTargetPrediction
Borneol	P12268	IMPDH2	SwissTargetPrediction
Borneol	P23458	JAK1	SwissTargetPrediction
Borneol	O60674	JAK2	SwissTargetPrediction
Borneol	P22460	KCNA5	SwissTargetPrediction
Borneol	P38571	LIPA	SwissTargetPrediction
Borneol	Q5S007	LRRK2	SwissTargetPrediction
Borneol	P09960	LTA4H	SwissTargetPrediction
Borneol	P45983	MAPK8	SwissTargetPrediction
Borneol	P45452	MMP13	SwissTargetPrediction
Borneol	P48039	MTNR1A	SwissTargetPrediction
Borneol	P49286	MTNR1B	SwissTargetPrediction
Borneol	Q9UHC9	NPC1L1	SwissTargetPrediction
Borneol	P16083	NQO2	SwissTargetPrediction
Borneol	Q13133	NR1H3	SwissTargetPrediction
Borneol	Q96RI1	NR1H4	SwissTargetPrediction
Borneol	Q14994	NR1I3	SwissTargetPrediction
Borneol	P04150	NR3C1	SwissTargetPrediction
Borneol	P08235	NR3C2	SwissTargetPrediction
Borneol	P22736	NR4A1	SwissTargetPrediction
Borneol	P41143	OPRD1	SwissTargetPrediction
Borneol	P41145	OPRK1	SwissTargetPrediction
Borneol	P35372	OPRM1	SwissTargetPrediction
Borneol	Q99572	P2RX7	SwissTargetPrediction
Borneol	P11940	PABPC1	SwissTargetPrediction
Borneol	P06401	PGR	SwissTargetPrediction
Borneol	P09884	POLA1	SwissTargetPrediction

Borneol	P06746	POLB	SwissTargetPrediction
Borneol	Q07869	PPARA	SwissTargetPrediction
Borneol	Q03181	PPARD	SwissTargetPrediction
Borneol	O60678	PRMT3	SwissTargetPrediction
Borneol	P25105	PTAFR	SwissTargetPrediction
Borneol	P43116	PTGER2	SwissTargetPrediction
Borneol	P43088	PTGFR	SwissTargetPrediction
Borneol	P35354	PTGS2	SwissTargetPrediction
Borneol	Q14524	SCN5A	SwissTargetPrediction
Borneol	Q15858	SCN9A	SwissTargetPrediction
Borneol	P08185	SERPINA6	SwissTargetPrediction
Borneol	P04278	SHBG	SwissTargetPrediction
Borneol	Q15465	SHH	SwissTargetPrediction
Borneol	Q99720	SIGMAR1	SwissTargetPrediction
Borneol	Q16572	SLC18A3	SwissTargetPrediction
Borneol	Q01959	SLC6A3	SwissTargetPrediction
Borneol	Q9NYA1	SPHK1	SwissTargetPrediction
Borneol	Q9P289	STK26	SwissTargetPrediction
Borneol	Q13188	STK3	SwissTargetPrediction
Borneol	Q9H2K2	TNKS2	SwissTargetPrediction
Borneol	Q7Z2W7	TRPM8	SwissTargetPrediction
Borneol	Q8NER1	TRPV1	SwissTargetPrediction
Borneol	Q8NET8	TRPV3	SwissTargetPrediction
Borneol	P33981	TTK	SwissTargetPrediction
Borneol	P04818	TYMS	SwissTargetPrediction
Borneol	P16662	UGT2B7	SwissTargetPrediction
Borneol	Q9UKP6	UTS2R	SwissTargetPrediction
Borneol	P11473	VDR	SwissTargetPrediction
Borneol	P31213	SRD5A2	TargetNet
Borneol	P28566	HTR1E	TargetNet
Borneol	P47898	HTR5A	TargetNet
Borneol	P22303	ACHE	TargetNet
Borneol	P22303	ACHE	TargetNet
Borneol	P22303	ACHE	TargetNet
Borneol	P18089	ADRA2B	TargetNet
Borneol	P18825	ADRA2C	TargetNet
Borneol	P18825	ADRA2C	TargetNet
Borneol	P21397	MAOA	TargetNet
Borneol	P10275	AR	TargetNet
Borneol	P10275	AR	TargetNet
Borneol	P16050	ALOX15	TargetNet
Borneol	P11511	CYP19A1	TargetNet
Borneol	P35869	AHR	TargetNet

Borneol	Q16548	BCL2A1	TargetNet
Borneol	P34913	EPHX2	TargetNet
Borneol	P21554	CNR1	TargetNet
Borneol	P21554	CNR1	TargetNet
Borneol	P34972	CNR2	TargetNet
Borneol	P34972	CNR2	TargetNet
Borneol	P34972	CNR2	TargetNet
Borneol	P00915	CA1	TargetNet
Borneol	O43570	CA12	TargetNet
Borneol	Q9ULX7	CA14	TargetNet
Borneol	P00918	CA2	TargetNet
Borneol	P22748	CA4	TargetNet
Borneol	P35218	CA5A	TargetNet
Borneol	Q9Y2D0	CA5B	TargetNet
Borneol	P23280	CA6	TargetNet
Borneol	P43166	CA7	TargetNet
Borneol	Q16790	CA9	TargetNet
Borneol	Q494W8	CHRFAM7A	TargetNet
Borneol	O00748	CES2	TargetNet
Borneol	P28845	HSD11B1	TargetNet
Borneol	P28845	HSD11B1	TargetNet
Borneol	P80365	HSD11B2	TargetNet
Borneol	P24468	NR2F2	TargetNet
Borneol	P05177	CYP1A2	TargetNet
Borneol	P33261	CYP2C19	TargetNet
Borneol	P21728	DRD1	TargetNet
Borneol	P21918	DRD5	TargetNet
Borneol	Q9UHL4	DPP7	TargetNet
Borneol	P27487	DPP4	TargetNet
Borneol	Q6V1X1	DPP8	TargetNet
Borneol	Q86TI2	DPP9	TargetNet
Borneol	P26358	DNMT1	TargetNet
Borneol	P31941	APOBEC3A	TargetNet
Borneol	Q9HC16	APOBEC3G	TargetNet
Borneol	P51452	DUSP3	TargetNet
Borneol	P03372	ESR1	TargetNet
Borneol	Q92731	ESR2	TargetNet
Borneol	P14324	FDPS	TargetNet
Borneol	Q8TDU6	GPBAR1	TargetNet
Borneol	Q9HC97	GPR35	TargetNet
Borneol	Q05469	LIPE	TargetNet
Borneol	P23141	CES1	TargetNet
Borneol	P14174	MIF	TargetNet

Borneol	Q99685	MGLL	TargetNet
Borneol	P30305	CDC25B	TargetNet
Borneol	P11229	CHRM1	TargetNet
Borneol	P11229	CHRM1	TargetNet
Borneol	P08172	CHRM2	TargetNet
Borneol	P08172	CHRM2	TargetNet
Borneol	P20309	CHRM3	TargetNet
Borneol	P08173	CHRM4	TargetNet
Borneol	P43681	CHRNA4	TargetNet
Borneol	P43681	CHRNA4	TargetNet
Borneol	P36544	CHRNA7	TargetNet
Borneol	P36544	CHRNA7	TargetNet
Borneol	P29475	NOS1	TargetNet
Borneol	P29475	NOS1	TargetNet
Borneol	P29474	NOS3	TargetNet
Borneol	P35228	NOS2	TargetNet
Borneol	P35228	NOS2	TargetNet
Borneol	P35398	RORA	TargetNet
Borneol	Q00G26	PLIN5	TargetNet
Borneol	P11086	PNMT	TargetNet
Borneol	P23219	PTGS1	TargetNet
Borneol	P23219	PTGS1	TargetNet
Borneol	P35354	PTGS2	TargetNet
Borneol	O43353	RIPK2	TargetNet
Borneol	P10276	RARA	TargetNet
Borneol	P10826	RARB	TargetNet
Borneol	P13631	RARG	TargetNet
Borneol	Q99720	SIGMAR1	TargetNet
Borneol	Q99720	SIGMAR1	TargetNet
Borneol	Q99720	SIGMAR1	TargetNet
Borneol	Q01959	SLC6A3	TargetNet
Borneol	Q01959	SLC6A3	TargetNet
Borneol	P23975	SLC6A2	TargetNet
Borneol	O95136	S1PR2	TargetNet
Borneol	Q9H228	S1PR5	TargetNet
Borneol	P05093	CYP17A1	TargetNet
Borneol	P08842	STS	TargetNet
Borneol	Q9NR96	TLR9	TargetNet
Bornyl acetate	O94759	TRPM2	SEA
Bornyl acetate	P59538	TAS2R31	SEA
Bornyl acetate	Q96IY4	CPB2	SEA
Bornyl acetate	P22303	ACHE	SwissTargetPrediction
Bornyl acetate	P07327	ADH1A	SwissTargetPrediction

Bornyl acetate	P30542	ADORA1	SwissTargetPrediction
Bornyl acetate	P29274	ADORA2A	SwissTargetPrediction
Bornyl acetate	P35348	ADRA1A	SwissTargetPrediction
Bornyl acetate	P35368	ADRA1B	SwissTargetPrediction
Bornyl acetate	P25100	ADRA1D	SwissTargetPrediction
Bornyl acetate	P08913	ADRA2A	SwissTargetPrediction
Bornyl acetate	P18089	ADRA2B	SwissTargetPrediction
Bornyl acetate	P18825	ADRA2C	SwissTargetPrediction
Bornyl acetate	P00352	ALDH1A1	SwissTargetPrediction
Bornyl acetate	P05091	ALDH2	SwissTargetPrediction
Bornyl acetate	P30838	ALDH3A1	SwissTargetPrediction
Bornyl acetate	P00915	CA1	SwissTargetPrediction
Bornyl acetate	P00918	CA2	SwissTargetPrediction
Bornyl acetate	P51681	CCR5	SwissTargetPrediction
Bornyl acetate	P30304	CDC25A	SwissTargetPrediction
Bornyl acetate	P50750	CDK9	SwissTargetPrediction
Bornyl acetate	P50750	CDK9	SwissTargetPrediction
Bornyl acetate	P19835	CEL	SwissTargetPrediction
Bornyl acetate	P23141	CES1	SwissTargetPrediction
Bornyl acetate	P32297	CHRNA3	SwissTargetPrediction
Bornyl acetate	P43681	CHRNA4	SwissTargetPrediction
Bornyl acetate	P43681	CHRNA4	SwissTargetPrediction
Bornyl acetate	P17787	CHRNB2	SwissTargetPrediction
Bornyl acetate	P30926	CHRNB4	SwissTargetPrediction
Bornyl acetate	P17538	CTRB1	SwissTargetPrediction
Bornyl acetate	Q99895	CTRC	SwissTargetPrediction
Bornyl acetate	P53634	CTSC	SwissTargetPrediction
Bornyl acetate	P43235	CTSK	SwissTargetPrediction
Bornyl acetate	P25774	CTSS	SwissTargetPrediction
Bornyl acetate	O60911	CTSV	SwissTargetPrediction
Bornyl acetate	P15538	CYP11B1	SwissTargetPrediction
Bornyl acetate	P19099	CYP11B2	SwissTargetPrediction
Bornyl acetate	P11511	CYP19A1	SwissTargetPrediction
Bornyl acetate	P11509	CYP2A6	SwissTargetPrediction
Bornyl acetate	P00374	DHFR	SwissTargetPrediction
Bornyl acetate	P21917	DRD4	SwissTargetPrediction
Bornyl acetate	P00533	EGFR	SwissTargetPrediction
Bornyl acetate	P08246	ELANE	SwissTargetPrediction
Bornyl acetate	P07099	EPHX1	SwissTargetPrediction
Bornyl acetate	P00734	F2	SwissTargetPrediction
Bornyl acetate	P36888	FLT3	SwissTargetPrediction
Bornyl acetate	P49354	FNTA	SwissTargetPrediction
Bornyl acetate	P14867	GABRA1	SwissTargetPrediction

Bornyl acetate	P47869	GABRA2	SwissTargetPrediction
Bornyl acetate	P34903	GABRA3	SwissTargetPrediction
Bornyl acetate	P31644	GABRA5	SwissTargetPrediction
Bornyl acetate	P47870	GABRB2	SwissTargetPrediction
Bornyl acetate	P28472	GABRB3	SwissTargetPrediction
Bornyl acetate	P28472	GABRB3	SwissTargetPrediction
Bornyl acetate	P28472	GABRB3	SwissTargetPrediction
Bornyl acetate	P18507	GABRG2	SwissTargetPrediction
Bornyl acetate	P18507	GABRG2	SwissTargetPrediction
Bornyl acetate	P18507	GABRG2	SwissTargetPrediction
Bornyl acetate	P18507	GABRG2	SwissTargetPrediction
Bornyl acetate	Q14833	GRM4	SwissTargetPrediction
Bornyl acetate	P41594	GRM5	SwissTargetPrediction
Bornyl acetate	P49841	GSK3B	SwissTargetPrediction
Bornyl acetate	P04035	HMGCR	SwissTargetPrediction
Bornyl acetate	P09601	HMOX1	SwissTargetPrediction
Bornyl acetate	P28845	HSD11B1	SwissTargetPrediction
Bornyl acetate	P37058	HSD17B3	SwissTargetPrediction
Bornyl acetate	P08908	HTR1A	SwissTargetPrediction
Bornyl acetate	P42858	HTT	SwissTargetPrediction
Bornyl acetate	O75874	IDH1	SwissTargetPrediction
Bornyl acetate	P14902	IDO1	SwissTargetPrediction
Bornyl acetate	Q12809	KCNH2	SwissTargetPrediction
Bornyl acetate	O95069	KCNK2	SwissTargetPrediction
Bornyl acetate	Q92952	KCNN1	SwissTargetPrediction
Bornyl acetate	Q9H2S1	KCNN2	SwissTargetPrediction
Bornyl acetate	Q9UGI6	KCNN3	SwissTargetPrediction
Bornyl acetate	P06239	LCK	SwissTargetPrediction
Bornyl acetate	Q05469	LIPE	SwissTargetPrediction
Bornyl acetate	Q5S007	LRRK2	SwissTargetPrediction
Bornyl acetate	P27338	MAOB	SwissTargetPrediction
Bornyl acetate	Q16539	MAPK14	SwissTargetPrediction
Bornyl acetate	Q99705	MCHR1	SwissTargetPrediction
Bornyl acetate	P08581	MET	SwissTargetPrediction
Bornyl acetate	P05164	MPO	SwissTargetPrediction
Bornyl acetate	P29475	NOS1	SwissTargetPrediction
Bornyl acetate	P29474	NOS3	SwissTargetPrediction
Bornyl acetate	O75469	NR1I2	SwissTargetPrediction
Bornyl acetate	P36639	NUDT1	SwissTargetPrediction
Bornyl acetate	Q99572	P2RX7	SwissTargetPrediction
Bornyl acetate	P11940	PABPC1	SwissTargetPrediction
Bornyl acetate	P09874	PARP1	SwissTargetPrediction
Bornyl acetate	Q9Y233	PDE10A	SwissTargetPrediction

Bornyl acetate	P53609	PGGT1B	SwissTargetPrediction
Bornyl acetate	O00329	PIK3CD	SwissTargetPrediction
Bornyl acetate	P27986	PIK3R1	SwissTargetPrediction
Bornyl acetate	O60733	PLA2G6	SwissTargetPrediction
Bornyl acetate	P09884	POLA1	SwissTargetPrediction
Bornyl acetate	P17252	PRKCA	SwissTargetPrediction
Bornyl acetate	P78527	PRKDC	SwissTargetPrediction
Bornyl acetate	P07477	PRSS1	SwissTargetPrediction
Bornyl acetate	P23219	PTGS1	SwissTargetPrediction
Bornyl acetate	P18031	PTPN1	SwissTargetPrediction
Bornyl acetate	Q16769	QPCT	SwissTargetPrediction
Bornyl acetate	Q15465	SHH	SwissTargetPrediction
Bornyl acetate	P23975	SLC6A2	SwissTargetPrediction
Bornyl acetate	P40763	STAT3	SwissTargetPrediction
Bornyl acetate	Q96RJ0	TAAR1	SwissTargetPrediction
Bornyl acetate	P24557	TBXAS1	SwissTargetPrediction
Bornyl acetate	P36897	TGFBR1	SwissTargetPrediction
Bornyl acetate	Q9NR97	TLR8	SwissTargetPrediction
Bornyl acetate	Q9H2K2	TNKS2	SwissTargetPrediction
Bornyl acetate	O75762	TRPA1	SwissTargetPrediction
Bornyl acetate	Q7Z2W7	TRPM8	SwissTargetPrediction
Bornyl acetate	P04818	TYMS	SwissTargetPrediction
Bornyl acetate	P16662	UGT2B7	SwissTargetPrediction
Bornyl acetate	P11473	VDR	SwissTargetPrediction
Bornyl acetate	O14980	XPO1	SwissTargetPrediction
Bornyl acetate	P31213	SRD5A2	TargetNet
Bornyl acetate	P28566	HTR1E	TargetNet
Bornyl acetate	P22303	ACHE	TargetNet
Bornyl acetate	P22303	ACHE	TargetNet
Bornyl acetate	P18089	ADRA2B	TargetNet
Bornyl acetate	P18825	ADRA2C	TargetNet
Bornyl acetate	P10275	AR	TargetNet
Bornyl acetate	P10275	AR	TargetNet
Bornyl acetate	P16050	ALOX15	TargetNet
Bornyl acetate	P11511	CYP19A1	TargetNet
Bornyl acetate	P35869	AHR	TargetNet
Bornyl acetate	Q16548	BCL2A1	TargetNet
Bornyl acetate	P34913	EPHX2	TargetNet
Bornyl acetate	P34972	CNR2	TargetNet
Bornyl acetate	P34972	CNR2	TargetNet
Bornyl acetate	P00915	CA1	TargetNet
Bornyl acetate	O43570	CA12	TargetNet
Bornyl acetate	Q9ULX7	CA14	TargetNet

Bornyl acetate	P00918	CA2	TargetNet
Bornyl acetate	P22748	CA4	TargetNet
Bornyl acetate	P35218	CA5A	TargetNet
Bornyl acetate	Q9Y2D0	CA5B	TargetNet
Bornyl acetate	P23280	CA6	TargetNet
Bornyl acetate	P43166	CA7	TargetNet
Bornyl acetate	Q16790	CA9	TargetNet
Bornyl acetate	Q9GZU7	CTDSP1	TargetNet
Bornyl acetate	Q494W8	CHRFAM7A	TargetNet
Bornyl acetate	O00748	CES2	TargetNet
Bornyl acetate	P28845	HSD11B1	TargetNet
Bornyl acetate	P28845	HSD11B1	TargetNet
Bornyl acetate	P80365	HSD11B2	TargetNet
Bornyl acetate	P24468	NR2F2	TargetNet
Bornyl acetate	P33261	CYP2C19	TargetNet
Bornyl acetate	P21728	DRD1	TargetNet
Bornyl acetate	P26358	DNMT1	TargetNet
Bornyl acetate	P31941	APOBEC3A	TargetNet
Bornyl acetate	Q9HC16	APOBEC3G	TargetNet
Bornyl acetate	Q9HC97	GPR35	TargetNet
Bornyl acetate	P23141	CES1	TargetNet
Bornyl acetate	P14174	MIF	TargetNet
Bornyl acetate	Q99685	MGLL	TargetNet
Bornyl acetate	P30305	CDC25B	TargetNet
Bornyl acetate	P11229	CHRM1	TargetNet
Bornyl acetate	P11229	CHRM1	TargetNet
Bornyl acetate	P08172	CHRM2	TargetNet
Bornyl acetate	P08172	CHRM2	TargetNet
Bornyl acetate	P20309	CHRM3	TargetNet
Bornyl acetate	P20309	CHRM3	TargetNet
Bornyl acetate	P08173	CHRM4	TargetNet
Bornyl acetate	P08912	CHRM5	TargetNet
Bornyl acetate	P43681	CHRNA4	TargetNet
Bornyl acetate	P43681	CHRNA4	TargetNet
Bornyl acetate	P36544	CHRNA7	TargetNet
Bornyl acetate	P36544	CHRNA7	TargetNet
Bornyl acetate	P29475	NOS1	TargetNet
Bornyl acetate	P29474	NOS3	TargetNet
Bornyl acetate	P35228	NOS2	TargetNet
Bornyl acetate	P35398	RORA	TargetNet
Bornyl acetate	P23219	PTGS1	TargetNet
Bornyl acetate	P35354	PTGS2	TargetNet
Bornyl acetate	O43353	RIPK2	TargetNet

Bornyl acetate	P10276	RARA	TargetNet
Bornyl acetate	P10826	RARB	TargetNet
Bornyl acetate	P13631	RARG	TargetNet
Bornyl acetate	Q99720	SIGMAR1	TargetNet
Bornyl acetate	Q01959	SLC6A3	TargetNet
Bornyl acetate	O95136	S1PR2	TargetNet
Bornyl acetate	P05093	CYP17A1	TargetNet
Bornyl acetate	P08842	STS	TargetNet
Bornyl acetate	Q9NR96	TLR9	TargetNet
Camphene	P22303	ACHE	SwissTargetPrediction
Camphene	O60218	AKR1B10	SwissTargetPrediction
Camphene	P10275	AR	SwissTargetPrediction
Camphene	P30304	CDC25A	SwissTargetPrediction
Camphene	P30305	CDC25B	SwissTargetPrediction
Camphene	P08172	CHRM2	SwissTargetPrediction
Camphene	P34972	CNR2	SwissTargetPrediction
Camphene	P07339	CTSD	SwissTargetPrediction
Camphene	P05093	CYP17A1	SwissTargetPrediction
Camphene	P11511	CYP19A1	SwissTargetPrediction
Camphene	P33261	CYP2C19	SwissTargetPrediction
Camphene	Q16850	CYP51A1	SwissTargetPrediction
Camphene	P03372	ESR1	SwissTargetPrediction
Camphene	Q92731	ESR2	SwissTargetPrediction
Camphene	O00519	FAAH	SwissTargetPrediction
Camphene	P04035	HMGCR	SwissTargetPrediction
Camphene	P28845	HSD11B1	SwissTargetPrediction
Camphene	Q9UHC9	NPC1L1	SwissTargetPrediction
Camphene	Q13133	NR1H3	SwissTargetPrediction
Camphene	Q96RI1	NR1H4	SwissTargetPrediction
Camphene	Q14994	NR1I3	SwissTargetPrediction
Camphene	P06746	POLB	SwissTargetPrediction
Camphene	Q07869	PPARA	SwissTargetPrediction
Camphene	O14684	PTGES	SwissTargetPrediction
Camphene	P23219	PTGS1	SwissTargetPrediction
Camphene	P18031	PTPN1	SwissTargetPrediction
Camphene	P51449	RORC	SwissTargetPrediction
Camphene	Q9UBE0	SAE1	SwissTargetPrediction
Camphene	P04278	SHBG	SwissTargetPrediction
Camphene	Q9GZV3	SLC5A7	SwissTargetPrediction
Camphene	P23975	SLC6A2	SwissTargetPrediction
Camphene	P31645	SLC6A4	SwissTargetPrediction
Camphene	Q12772	SREBF2	SwissTargetPrediction
Camphene	Q8NER1	TRPV1	SwissTargetPrediction

Camphene	Q9UBT2	UBA2	SwissTargetPrediction
Camphene	P16662	UGT2B7	SwissTargetPrediction
Camphene	P18405	SRD5A1	TargetNet
Camphene	P31213	SRD5A2	TargetNet
Camphene	P28566	HTR1E	TargetNet
Camphene	P28223	HTR2A	TargetNet
Camphene	P28335	HTR2C	TargetNet
Camphene	P47898	HTR5A	TargetNet
Camphene	P22303	ACHE	TargetNet
Camphene	P22303	ACHE	TargetNet
Camphene	P05186	ALPL	TargetNet
Camphene	P08913	ADRA2A	TargetNet
Camphene	P18089	ADRA2B	TargetNet
Camphene	P18825	ADRA2C	TargetNet
Camphene	P18825	ADRA2C	TargetNet
Camphene	P10275	AR	TargetNet
Camphene	P10275	AR	TargetNet
Camphene	P16050	ALOX15	TargetNet
Camphene	P11511	CYP19A1	TargetNet
Camphene	P35869	AHR	TargetNet
Camphene	Q16548	BCL2A1	TargetNet
Camphene	P22748	CA4	TargetNet
Camphene	P35218	CA5A	TargetNet
Camphene	Q9Y2D0	CA5B	TargetNet
Camphene	P23280	CA6	TargetNet
Camphene	P43166	CA7	TargetNet
Camphene	P55211	CASP9	TargetNet
Camphene	Q494W8	CHRFAM7A	TargetNet
Camphene	O00748	CES2	TargetNet
Camphene	P28845	HSD11B1	TargetNet
Camphene	P28845	HSD11B1	TargetNet
Camphene	P24468	NR2F2	TargetNet
Camphene	P05177	CYP1A2	TargetNet
Camphene	P21728	DRD1	TargetNet
Camphene	P21728	DRD1	TargetNet
Camphene	P21918	DRD5	TargetNet
Camphene	P31941	APOBEC3A	TargetNet
Camphene	Q9HC16	APOBEC3G	TargetNet
Camphene	Q92731	ESR2	TargetNet
Camphene	P04150	NR3C1	TargetNet
Camphene	P42262	GRIA2	TargetNet
Camphene	Q9HC97	GPR35	TargetNet
Camphene	Q9H3N8	HRH4	TargetNet

Camphene	P23141	CES1	TargetNet
Camphene	P14174	MIF	TargetNet
Camphene	Q14416	GRM2	TargetNet
Camphene	Q99685	MGLL	TargetNet
Camphene	P11229	CHRM1	TargetNet
Camphene	P11229	CHRM1	TargetNet
Camphene	P08172	CHRM2	TargetNet
Camphene	P08172	CHRM2	TargetNet
Camphene	P20309	CHRM3	TargetNet
Camphene	P08173	CHRM4	TargetNet
Camphene	P08912	CHRM5	TargetNet
Camphene	P43681	CHRNA4	TargetNet
Camphene	P43681	CHRNA4	TargetNet
Camphene	P36544	CHRNA7	TargetNet
Camphene	P36544	CHRNA7	TargetNet
Camphene	P29475	NOS1	TargetNet
Camphene	P29474	NOS3	TargetNet
Camphene	P35228	NOS2	TargetNet
Camphene	P11086	PNMT	TargetNet
Camphene	P06401	PGR	TargetNet
Camphene	P23219	PTGS1	TargetNet
Camphene	O43353	RIPK2	TargetNet
Camphene	P10276	RARA	TargetNet
Camphene	P10826	RARB	TargetNet
Camphene	P13631	RARG	TargetNet
Camphene	Q99720	SIGMAR1	TargetNet
Camphene	Q99720	SIGMAR1	TargetNet
Camphene	Q99720	SIGMAR1	TargetNet
Camphene	Q01959	SLC6A3	TargetNet
Camphene	Q01959	SLC6A3	TargetNet
Camphene	P23975	SLC6A2	TargetNet
Camphene	P31645	SLC6A4	TargetNet
Camphene	P31645	SLC6A4	TargetNet
Camphene	O95136	S1PR2	TargetNet
Camphene	P05093	CYP17A1	TargetNet
Camphene	P37058	HSD17B3	TargetNet
Camphene	Q9NR96	TLR9	TargetNet
Camphene	Q04206	RELA	TargetNet
Geranial	O95749	GGPS1	SEA
Geranial	P48449	LSS	SEA
Geranial	P49354	FNTA	SEA
Geranial	P49356	FNTB	SEA
Geranial	Q14534	SQLE	SEA

Geranial	Q9UNQ0	ABCG2	SwissTargetPrediction
Geranial	P22303	ACHE	SwissTargetPrediction
Geranial	P07327	ADH1A	SwissTargetPrediction
Geranial	P00325	ADH1B	SwissTargetPrediction
Geranial	P00326	ADH1C	SwissTargetPrediction
Geranial	P08319	ADH4	SwissTargetPrediction
Geranial	P40394	ADH7	SwissTargetPrediction
Geranial	P31749	AKT1	SwissTargetPrediction
Geranial	P00352	ALDH1A1	SwissTargetPrediction
Geranial	Q16853	AOC3	SwissTargetPrediction
Geranial	Q96BI3	APH1A	SwissTargetPrediction
Geranial	Q8WW43	APH1B	SwissTargetPrediction
Geranial	P00915	CA1	SwissTargetPrediction
Geranial	O43570	CA12	SwissTargetPrediction
Geranial	Q8N1Q1	CA13	SwissTargetPrediction
Geranial	Q9ULX7	CA14	SwissTargetPrediction
Geranial	P00918	CA2	SwissTargetPrediction
Geranial	P07451	CA3	SwissTargetPrediction
Geranial	Q9Y2D0	CA5B	SwissTargetPrediction
Geranial	P43166	CA7	SwissTargetPrediction
Geranial	Q16790	CA9	SwissTargetPrediction
Geranial	P24385	CCND1	SwissTargetPrediction
Geranial	P30304	CDC25A	SwissTargetPrediction
Geranial	O00311	CDC7	SwissTargetPrediction
Geranial	P11802	CDK4	SwissTargetPrediction
Geranial	P19835	CEL	SwissTargetPrediction
Geranial	Q02224	CENPE	SwissTargetPrediction
Geranial	P11229	CHRM1	SwissTargetPrediction
Geranial	P08172	CHRM2	SwissTargetPrediction
Geranial	P20309	CHRM3	SwissTargetPrediction
Geranial	P08173	CHRM4	SwissTargetPrediction
Geranial	P08912	CHRM5	SwissTargetPrediction
Geranial	P43681	CHRNA4	SwissTargetPrediction
Geranial	P17787	CHRNB2	SwissTargetPrediction
Geranial	P21554	CNR1	SwissTargetPrediction
Geranial	P43235	CTSK	SwissTargetPrediction
Geranial	P15538	CYP11B1	SwissTargetPrediction
Geranial	P19099	CYP11B2	SwissTargetPrediction
Geranial	P11511	CYP19A1	SwissTargetPrediction
Geranial	P11509	CYP2A6	SwissTargetPrediction
Geranial	P33261	CYP2C19	SwissTargetPrediction
Geranial	Q9UBU7	DBF4	SwissTargetPrediction
Geranial	P21728	DRD1	SwissTargetPrediction

Geranial	P14416	DRD2	SwissTargetPrediction
Geranial	P21917	DRD4	SwissTargetPrediction
Geranial	Q92630	DYRK2	SwissTargetPrediction
Geranial	P07099	EPHX1	SwissTargetPrediction
Geranial	P34913	EPHX2	SwissTargetPrediction
Geranial	P03372	ESR1	SwissTargetPrediction
Geranial	Q92731	ESR2	SwissTargetPrediction
Geranial	P00488	F13A1	SwissTargetPrediction
Geranial	P05413	FABP3	SwissTargetPrediction
Geranial	P15090	FABP4	SwissTargetPrediction
Geranial	P14867	GABRA1	SwissTargetPrediction
Geranial	P47870	GABRB2	SwissTargetPrediction
Geranial	P18507	GABRG2	SwissTargetPrediction
Geranial	P08151	GLI1	SwissTargetPrediction
Geranial	P10070	GLI2	SwissTargetPrediction
Geranial	P41594	GRM5	SwissTargetPrediction
Geranial	P49841	GSK3B	SwissTargetPrediction
Geranial	P00390	GSR	SwissTargetPrediction
Geranial	Q8TF76	HASPIN	SwissTargetPrediction
Geranial	P50747	HLCS	SwissTargetPrediction
Geranial	P09601	HMOX1	SwissTargetPrediction
Geranial	P0DMV8	HSPA1A	SwissTargetPrediction
Geranial	P28335	HTR2C	SwissTargetPrediction
Geranial	P05362	ICAM1	SwissTargetPrediction
Geranial	O14920	IKBKB	SwissTargetPrediction
Geranial	P12268	IMPDH2	SwissTargetPrediction
Geranial	P23458	JAK1	SwissTargetPrediction
Geranial	O60674	JAK2	SwissTargetPrediction
Geranial	Q05469	LIPE	SwissTargetPrediction
Geranial	P21397	MAOA	SwissTargetPrediction
Geranial	P27338	MAOB	SwissTargetPrediction
Geranial	Q07820	MCL1	SwissTargetPrediction
Geranial	Q99685	MGLL	SwissTargetPrediction
Geranial	P05164	MPO	SwissTargetPrediction
Geranial	P48039	MTNR1A	SwissTargetPrediction
Geranial	P49286	MTNR1B	SwissTargetPrediction
Geranial	Q02083	NAAA	SwissTargetPrediction
Geranial	Q92542	NCSTN	SwissTargetPrediction
Geranial	Q6P988	NOTUM	SwissTargetPrediction
Geranial	P16083	NQO2	SwissTargetPrediction
Geranial	P22736	NR4A1	SwissTargetPrediction
Geranial	Q99572	P2RX7	SwissTargetPrediction
Geranial	P11940	PABPC1	SwissTargetPrediction

Geranial	P09874	PARP1	SwissTargetPrediction
Geranial	Q9UGN5	PARP2	SwissTargetPrediction
Geranial	Q08499	PDE4D	SwissTargetPrediction
Geranial	P06401	PGR	SwissTargetPrediction
Geranial	P37231	PPARG	SwissTargetPrediction
Geranial	P49768	PSEN1	SwissTargetPrediction
Geranial	P49810	PSEN2	SwissTargetPrediction
Geranial	Q9NZ42	PSENEN	SwissTargetPrediction
Geranial	P28074	PSMB5	SwissTargetPrediction
Geranial	P23219	PTGS1	SwissTargetPrediction
Geranial	P35354	PTGS2	SwissTargetPrediction
Geranial	Q14289	PTK2B	SwissTargetPrediction
Geranial	P17706	PTPN2	SwissTargetPrediction
Geranial	P08575	PTPRC	SwissTargetPrediction
Geranial	P10586	PTPRF	SwissTargetPrediction
Geranial	P16581	SELE	SwissTargetPrediction
Geranial	Q8IXJ6	SIRT2	SwissTargetPrediction
Geranial	P18405	SRD5A1	SwissTargetPrediction
Geranial	O14746	TERT	SwissTargetPrediction
Geranial	P21980	TGM2	SwissTargetPrediction
Geranial	P07202	TPO	SwissTargetPrediction
Geranial	O75762	TRPA1	SwissTargetPrediction
Geranial	P02766	TTR	SwissTargetPrediction
Geranial	P19971	TYMP	SwissTargetPrediction
Geranial	P28566	HTR1E	TargetNet
Geranial	P28335	HTR2C	TargetNet
Geranial	P22303	ACHE	TargetNet
Geranial	P22303	ACHE	TargetNet
Geranial	P15121	AKR1B1	TargetNet
Geranial	P18089	ADRA2B	TargetNet
Geranial	P18825	ADRA2C	TargetNet
Geranial	P18825	ADRA2C	TargetNet
Geranial	P21397	MAOA	TargetNet
Geranial	P27338	MAOB	TargetNet
Geranial	P16050	ALOX15	TargetNet
Geranial	P09917	ALOX5	TargetNet
Geranial	Q16548	BCL2A1	TargetNet
Geranial	P00915	CA1	TargetNet
Geranial	O43570	CA12	TargetNet
Geranial	Q8N1Q1	CA13	TargetNet
Geranial	Q9ULX7	CA14	TargetNet
Geranial	P00918	CA2	TargetNet
Geranial	P22748	CA4	TargetNet

Geranial	P35218	CA5A	TargetNet
Geranial	Q9Y2D0	CA5B	TargetNet
Geranial	P23280	CA6	TargetNet
Geranial	P43166	CA7	TargetNet
Geranial	Q16790	CA9	TargetNet
Geranial	Q9GZU7	CTDSP1	TargetNet
Geranial	P55211	CASP9	TargetNet
Geranial	O00748	CES2	TargetNet
Geranial	P21918	DRD5	TargetNet
Geranial	P26358	DNMT1	TargetNet
Geranial	P31941	APOBEC3A	TargetNet
Geranial	Q9HC16	APOBEC3G	TargetNet
Geranial	P51452	DUSP3	TargetNet
Geranial	P14324	FDPS	TargetNet
Geranial	Q04609	FOLH1	TargetNet
Geranial	Q9HC97	GPR35	TargetNet
Geranial	P25021	HRH2	TargetNet
Geranial	P56524	HDAC4	TargetNet
Geranial	P23141	CES1	TargetNet
Geranial	P14174	MIF	TargetNet
Geranial	Q99685	MGLL	TargetNet
Geranial	P30305	CDC25B	TargetNet
Geranial	P11229	CHRM1	TargetNet
Geranial	P11229	CHRM1	TargetNet
Geranial	P08172	CHRM2	TargetNet
Geranial	P08172	CHRM2	TargetNet
Geranial	P20309	CHRM3	TargetNet
Geranial	P08173	CHRM4	TargetNet
Geranial	P08912	CHRM5	TargetNet
Geranial	P43681	CHRNA4	TargetNet
Geranial	P29475	NOS1	TargetNet
Geranial	P29474	NOS3	TargetNet
Geranial	P35228	NOS2	TargetNet
Geranial	P35228	NOS2	TargetNet
Geranial	P35398	RORA	TargetNet
Geranial	O60240	PLIN1	TargetNet
Geranial	Q00G26	PLIN5	TargetNet
Geranial	P23219	PTGS1	TargetNet
Geranial	P23219	PTGS1	TargetNet
Geranial	P35354	PTGS2	TargetNet
Geranial	O43353	RIPK2	TargetNet
Geranial	P28702	RXRB	TargetNet
Geranial	Q99720	SIGMAR1	TargetNet

Geranial	O95136	S1PR2	TargetNet
Geranial	Q9NR96	TLR9	TargetNet
Geranial	Q04206	RELA	TargetNet
α-Farnesene	O95749	GGPS1	SEA
α-Farnesene	P48449	LSS	SEA
α-Farnesene	P49354	FNTA	SEA
α-Farnesene	P49356	FNTB	SEA
α-Farnesene	Q14534	SQLE	SEA
α-Farnesene	P22303	ACHE	TargetNet
α-Farnesene	P22303	ACHE	TargetNet
α-Farnesene	P05186	ALPL	TargetNet
α-Farnesene	P18825	ADRA2C	TargetNet
α-Farnesene	P16050	ALOX15	TargetNet
α-Farnesene	P09917	ALOX5	TargetNet
α-Farnesene	Q16548	BCL2A1	TargetNet
α-Farnesene	O43570	CA12	TargetNet
α-Farnesene	P22748	CA4	TargetNet
α-Farnesene	Q9Y2D0	CA5B	TargetNet
α-Farnesene	P23280	CA6	TargetNet
α-Farnesene	P43166	CA7	TargetNet
α-Farnesene	Q16790	CA9	TargetNet
α-Farnesene	P55211	CASP9	TargetNet
α-Farnesene	O00748	CES2	TargetNet
α-Farnesene	P24468	NR2F2	TargetNet
α-Farnesene	P26358	DNMT1	TargetNet
α-Farnesene	P31941	APOBEC3A	TargetNet
α-Farnesene	Q9HC16	APOBEC3G	TargetNet
α-Farnesene	P51452	DUSP3	TargetNet
α-Farnesene	P14324	FDPS	TargetNet
α-Farnesene	Q9HC97	GPR35	TargetNet
α-Farnesene	P56524	HDAC4	TargetNet
α-Farnesene	P23141	CES1	TargetNet
α-Farnesene	Q99685	MGLL	TargetNet
α-Farnesene	P30305	CDC25B	TargetNet
α-Farnesene	P11229	CHRM1	TargetNet
α-Farnesene	P08172	CHRM2	TargetNet
α-Farnesene	P08173	CHRM4	TargetNet
α-Farnesene	P29475	NOS1	TargetNet
α-Farnesene	P29474	NOS3	TargetNet
α-Farnesene	P35228	NOS2	TargetNet
α-Farnesene	P35398	RORA	TargetNet
α-Farnesene	O60240	PLIN1	TargetNet
α-Farnesene	P04054	PLA2G1B	TargetNet

α-Farnesene	P23219	PTGS1	TargetNet
α-Farnesene	P35354	PTGS2	TargetNet
α-Farnesene	O43353	RIPK2	TargetNet
α-Farnesene	O95136	S1PR2	TargetNet
α-Farnesene	Q9NR96	TLR9	TargetNet
α-Farnesene	Q04206	RELA	TargetNet
α-Pinene	P49682	CXCR3	SEA
α-Pinene	Q9NUW8	TDP1	SEA
α-Pinene	P22303	ACHE	SwissTargetPrediction
α-Pinene	P24666	ACP1	SwissTargetPrediction
α-Pinene	P30542	ADORA1	SwissTargetPrediction
α-Pinene	P29274	ADORA2A	SwissTargetPrediction
α-Pinene	P0DMS8	ADORA3	SwissTargetPrediction
α-Pinene	P18089	ADRA2B	SwissTargetPrediction
α-Pinene	O60218	AKR1B10	SwissTargetPrediction
α-Pinene	P09917	ALOX5	SwissTargetPrediction
α-Pinene	P10275	AR	SwissTargetPrediction
α-Pinene	P56817	BACE1	SwissTargetPrediction
α-Pinene	P06276	BCHE	SwissTargetPrediction
α-Pinene	P51681	CCR5	SwissTargetPrediction
α-Pinene	P60033	CD81	SwissTargetPrediction
α-Pinene	P30304	CDC25A	SwissTargetPrediction
α-Pinene	P30305	CDC25B	SwissTargetPrediction
α-Pinene	P23141	CES1	SwissTargetPrediction
α-Pinene	O00748	CES2	SwissTargetPrediction
α-Pinene	P08172	CHRM2	SwissTargetPrediction
α-Pinene	P21554	CNR1	SwissTargetPrediction
α-Pinene	P34972	CNR2	SwissTargetPrediction
α-Pinene	P07339	CTSD	SwissTargetPrediction
α-Pinene	P05093	CYP17A1	SwissTargetPrediction
α-Pinene	P11511	CYP19A1	SwissTargetPrediction
α-Pinene	P33261	CYP2C19	SwissTargetPrediction
α-Pinene	Q16850	CYP51A1	SwissTargetPrediction
α-Pinene	P03372	ESR1	SwissTargetPrediction
α-Pinene	Q92731	ESR2	SwissTargetPrediction
α-Pinene	O00519	FAAH	SwissTargetPrediction
α-Pinene	P07148	FABP1	SwissTargetPrediction
α-Pinene	P05413	FABP3	SwissTargetPrediction
α-Pinene	P15090	FABP4	SwissTargetPrediction
α-Pinene	Q01469	FABP5	SwissTargetPrediction
α-Pinene	O14842	FFAR1	SwissTargetPrediction
α-Pinene	P49354	FNTA	SwissTargetPrediction
α-Pinene	P49356	FNTB	SwissTargetPrediction

α-Pinene	P11413	G6PD	SwissTargetPrediction
α-Pinene	P04035	HMGCR	SwissTargetPrediction
α-Pinene	P28845	HSD11B1	SwissTargetPrediction
α-Pinene	P80365	HSD11B2	SwissTargetPrediction
α-Pinene	P37058	HSD17B3	SwissTargetPrediction
α-Pinene	P41595	HTR2B	SwissTargetPrediction
α-Pinene	P48449	LSS	SwissTargetPrediction
α-Pinene	P28482	MAPK1	SwissTargetPrediction
α-Pinene	Q16539	MAPK14	SwissTargetPrediction
α-Pinene	P27361	MAPK3	SwissTargetPrediction
α-Pinene	P35228	NOS2	SwissTargetPrediction
α-Pinene	Q9UHC9	NPC1L1	SwissTargetPrediction
α-Pinene	P55055	NR1H2	SwissTargetPrediction
α-Pinene	Q13133	NR1H3	SwissTargetPrediction
α-Pinene	O75469	NR1I2	SwissTargetPrediction
α-Pinene	Q14994	NR1I3	SwissTargetPrediction
α-Pinene	P04150	NR3C1	SwissTargetPrediction
α-Pinene	P08235	NR3C2	SwissTargetPrediction
α-Pinene	Q08499	PDE4D	SwissTargetPrediction
α-Pinene	P06401	PGR	SwissTargetPrediction
α-Pinene	P04054	PLA2G1B	SwissTargetPrediction
α-Pinene	P47712	PLA2G4A	SwissTargetPrediction
α-Pinene	P06746	POLB	SwissTargetPrediction
α-Pinene	Q07869	PPARA	SwissTargetPrediction
α-Pinene	Q03181	PPARD	SwissTargetPrediction
α-Pinene	P37231	PPARG	SwissTargetPrediction
α-Pinene	P48147	PREP	SwissTargetPrediction
α-Pinene	P24723	PRKCH	SwissTargetPrediction
α-Pinene	P34995	PTGER1	SwissTargetPrediction
α-Pinene	P43116	PTGER2	SwissTargetPrediction
α-Pinene	O14684	PTGES	SwissTargetPrediction
α-Pinene	P23219	PTGS1	SwissTargetPrediction
α-Pinene	P18031	PTPN1	SwissTargetPrediction
α-Pinene	Q06124	PTPN11	SwissTargetPrediction
α-Pinene	P17706	PTPN2	SwissTargetPrediction
α-Pinene	P29350	PTPN6	SwissTargetPrediction
α-Pinene	P10586	PTPRF	SwissTargetPrediction
α-Pinene	P10276	RARA	SwissTargetPrediction
α-Pinene	P10826	RARB	SwissTargetPrediction
α-Pinene	P13631	RARG	SwissTargetPrediction
α-Pinene	P02753	RBP4	SwissTargetPrediction
α-Pinene	P35398	RORA	SwissTargetPrediction
α-Pinene	Q92753	RORB	SwissTargetPrediction

α-Pinene	P51449	RORC	SwissTargetPrediction
α-Pinene	P19793	RXRA	SwissTargetPrediction
α-Pinene	P28702	RXRB	SwissTargetPrediction
α-Pinene	P48443	RXRG	SwissTargetPrediction
α-Pinene	O00767	SCD	SwissTargetPrediction
α-Pinene	P08185	SERPINA6	SwissTargetPrediction
α-Pinene	P04278	SHBG	SwissTargetPrediction
α-Pinene	Q99720	SIGMAR1	SwissTargetPrediction
α-Pinene	Q9GZV3	SLC5A7	SwissTargetPrediction
α-Pinene	P23975	SLC6A2	SwissTargetPrediction
α-Pinene	Q01959	SLC6A3	SwissTargetPrediction
α-Pinene	P31645	SLC6A4	SwissTargetPrediction
α-Pinene	Q9NYA1	SPHK1	SwissTargetPrediction
α-Pinene	Q9NRA0	SPHK2	SwissTargetPrediction
α-Pinene	Q14534	SQLE	SwissTargetPrediction
α-Pinene	P18405	SRD5A1	SwissTargetPrediction
α-Pinene	P31213	SRD5A2	SwissTargetPrediction
α-Pinene	Q12772	SREBF2	SwissTargetPrediction
α-Pinene	O14746	TERT	SwissTargetPrediction
α-Pinene	P11387	TOP1	SwissTargetPrediction
α-Pinene	O75762	TRPA1	SwissTargetPrediction
α-Pinene	Q8NER1	TRPV1	SwissTargetPrediction
α-Pinene	P16662	UGT2B7	SwissTargetPrediction
α-Pinene	P28566	HTR1E	TargetNet
α-Pinene	P28223	HTR2A	TargetNet
α-Pinene	P28335	HTR2C	TargetNet
α-Pinene	P28335	HTR2C	TargetNet
α-Pinene	P47898	HTR5A	TargetNet
α-Pinene	P22303	ACHE	TargetNet
α-Pinene	P05186	ALPL	TargetNet
α-Pinene	P08913	ADRA2A	TargetNet
α-Pinene	P18089	ADRA2B	TargetNet
α-Pinene	P18825	ADRA2C	TargetNet
α-Pinene	P18825	ADRA2C	TargetNet
α-Pinene	P10275	AR	TargetNet
α-Pinene	P10275	AR	TargetNet
α-Pinene	P16050	ALOX15	TargetNet
α-Pinene	P09917	ALOX5	TargetNet
α-Pinene	P11511	CYP19A1	TargetNet
α-Pinene	P35869	AHR	TargetNet
α-Pinene	Q16548	BCL2A1	TargetNet
α-Pinene	P34972	CNR2	TargetNet
α-Pinene	P34972	CNR2	TargetNet

α-Pinene	P00915	CA1	TargetNet
α-Pinene	O43570	CA12	TargetNet
α-Pinene	Q8N1Q1	CA13	TargetNet
α-Pinene	Q9ULX7	CA14	TargetNet
α-Pinene	P22748	CA4	TargetNet
α-Pinene	P35218	CA5A	TargetNet
α-Pinene	Q9Y2D0	CA5B	TargetNet
α-Pinene	P23280	CA6	TargetNet
α-Pinene	P43166	CA7	TargetNet
α-Pinene	Q16790	CA9	TargetNet
α-Pinene	P55211	CASP9	TargetNet
α-Pinene	O00748	CES2	TargetNet
α-Pinene	P28845	HSD11B1	TargetNet
α-Pinene	P24468	NR2F2	TargetNet
α-Pinene	P49682	CXCR3	TargetNet
α-Pinene	P05177	CYP1A2	TargetNet
α-Pinene	P33261	CYP2C19	TargetNet
α-Pinene	P11712	CYP2C9	TargetNet
α-Pinene	P21728	DRD1	TargetNet
α-Pinene	P21728	DRD1	TargetNet
α-Pinene	P21918	DRD5	TargetNet
α-Pinene	P31941	APOBEC3A	TargetNet
α-Pinene	Q9HC16	APOBEC3G	TargetNet
α-Pinene	P49759	CLK1	TargetNet
α-Pinene	P51452	DUSP3	TargetNet
α-Pinene	P03372	ESR1	TargetNet
α-Pinene	Q92731	ESR2	TargetNet
α-Pinene	P42262	GRIA2	TargetNet
α-Pinene	Q9HC97	GPR35	TargetNet
α-Pinene	Q8TDS4	HCAR2	TargetNet
α-Pinene	P23141	CES1	TargetNet
α-Pinene	P14174	MIF	TargetNet
α-Pinene	Q14416	GRM2	TargetNet
α-Pinene	Q99685	MGLL	TargetNet
α-Pinene	P30305	CDC25B	TargetNet
α-Pinene	P11229	CHRM1	TargetNet
α-Pinene	P11229	CHRM1	TargetNet
α-Pinene	P08172	CHRM2	TargetNet
α-Pinene	P20309	CHRM3	TargetNet
α-Pinene	P08173	CHRM4	TargetNet
α-Pinene	P08912	CHRM5	TargetNet
α-Pinene	P43681	CHRNA4	TargetNet
α-Pinene	P43681	CHRNA4	TargetNet

α-Pinene	P36544	CHRNA7	TargetNet
α-Pinene	P29475	NOS1	TargetNet
α-Pinene	P29474	NOS3	TargetNet
α-Pinene	P35228	NOS2	TargetNet
α-Pinene	P35228	NOS2	TargetNet
α-Pinene	P35398	RORA	TargetNet
α-Pinene	P11086	PNMT	TargetNet
α-Pinene	Q9Y5X4	NR2E3	TargetNet
α-Pinene	P23219	PTGS1	TargetNet
α-Pinene	P23219	PTGS1	TargetNet
α-Pinene	P35354	PTGS2	TargetNet
α-Pinene	P35354	PTGS2	TargetNet
α-Pinene	O43353	RIPK2	TargetNet
α-Pinene	P10276	RARA	TargetNet
α-Pinene	P10826	RARB	TargetNet
α-Pinene	P13631	RARG	TargetNet
α-Pinene	Q99720	SIGMAR1	TargetNet
α-Pinene	Q01959	SLC6A3	TargetNet
α-Pinene	Q01959	SLC6A3	TargetNet
α-Pinene	P23975	SLC6A2	TargetNet
α-Pinene	P31645	SLC6A4	TargetNet
α-Pinene	P31645	SLC6A4	TargetNet
α-Pinene	O95136	S1PR2	TargetNet
α-Pinene	P05093	CYP17A1	TargetNet
α-Pinene	Q9NR96	TLR9	TargetNet
α-Pinene	Q96RJ0	TAAR1	TargetNet
α-Pinene	Q04206	RELA	TargetNet
α-Terpineol	P22303	ACHE	SwissTargetPrediction
α-Terpineol	P24666	ACP1	SwissTargetPrediction
α-Terpineol	P35348	ADRA1A	SwissTargetPrediction
α-Terpineol	P18825	ADRA2C	SwissTargetPrediction
α-Terpineol	O60218	AKR1B10	SwissTargetPrediction
α-Terpineol	P10275	AR	SwissTargetPrediction
α-Terpineol	P54707	ATP12A	SwissTargetPrediction
α-Terpineol	P06276	BCHE	SwissTargetPrediction
α-Terpineol	P25440	BRD2	SwissTargetPrediction
α-Terpineol	Q15059	BRD3	SwissTargetPrediction
α-Terpineol	O60885	BRD4	SwissTargetPrediction
α-Terpineol	P00915	CA1	SwissTargetPrediction
α-Terpineol	P00918	CA2	SwissTargetPrediction
α-Terpineol	P22748	CA4	SwissTargetPrediction
α-Terpineol	P60033	CD81	SwissTargetPrediction
α-Terpineol	O00748	CES2	SwissTargetPrediction

α-Terpineol	P08172	CHRM2	SwissTargetPrediction
α-Terpineol	P08173	CHRM4	SwissTargetPrediction
α-Terpineol	P43235	CTSK	SwissTargetPrediction
α-Terpineol	P25774	CTSS	SwissTargetPrediction
α-Terpineol	P15538	CYP11B1	SwissTargetPrediction
α-Terpineol	P19099	CYP11B2	SwissTargetPrediction
α-Terpineol	P05093	CYP17A1	SwissTargetPrediction
α-Terpineol	P11511	CYP19A1	SwissTargetPrediction
α-Terpineol	P33261	CYP2C19	SwissTargetPrediction
α-Terpineol	Q16850	CYP51A1	SwissTargetPrediction
α-Terpineol	Q9UBM7	DHCR7	SwissTargetPrediction
α-Terpineol	P14416	DRD2	SwissTargetPrediction
α-Terpineol	P00533	EGFR	SwissTargetPrediction
α-Terpineol	Q9BQI3	EIF2AK1	SwissTargetPrediction
α-Terpineol	P03372	ESR1	SwissTargetPrediction
α-Terpineol	Q92731	ESR2	SwissTargetPrediction
α-Terpineol	P07148	FABP1	SwissTargetPrediction
α-Terpineol	P05413	FABP3	SwissTargetPrediction
α-Terpineol	P15090	FABP4	SwissTargetPrediction
α-Terpineol	Q01469	FABP5	SwissTargetPrediction
α-Terpineol	O14842	FFAR1	SwissTargetPrediction
α-Terpineol	P49354	FNTA	SwissTargetPrediction
α-Terpineol	P49356	FNTB	SwissTargetPrediction
α-Terpineol	P11413	G6PD	SwissTargetPrediction
α-Terpineol	P04035	HMGCR	SwissTargetPrediction
α-Terpineol	P09601	HMOX1	SwissTargetPrediction
α-Terpineol	P28845	HSD11B1	SwissTargetPrediction
α-Terpineol	P28335	HTR2C	SwissTargetPrediction
α-Terpineol	P14902	IDO1	SwissTargetPrediction
α-Terpineol	P23458	JAK1	SwissTargetPrediction
α-Terpineol	O60674	JAK2	SwissTargetPrediction
α-Terpineol	P22460	KCNA5	SwissTargetPrediction
α-Terpineol	P38571	LIPA	SwissTargetPrediction
α-Terpineol	Q5S007	LRRK2	SwissTargetPrediction
α-Terpineol	P48449	LSS	SwissTargetPrediction
α-Terpineol	P09960	LTA4H	SwissTargetPrediction
α-Terpineol	Q15722	LTB4R	SwissTargetPrediction
α-Terpineol	P28482	MAPK1	SwissTargetPrediction
α-Terpineol	P48039	MTNR1A	SwissTargetPrediction
α-Terpineol	P49286	MTNR1B	SwissTargetPrediction
α-Terpineol	Q9UHC9	NPC1L1	SwissTargetPrediction
α-Terpineol	P55055	NR1H2	SwissTargetPrediction
α-Terpineol	Q13133	NR1H3	SwissTargetPrediction

α-Terpineol	Q14994	NR1I3	SwissTargetPrediction
α-Terpineol	P04150	NR3C1	SwissTargetPrediction
α-Terpineol	P08235	NR3C2	SwissTargetPrediction
α-Terpineol	P41143	OPRD1	SwissTargetPrediction
α-Terpineol	P41145	OPRK1	SwissTargetPrediction
α-Terpineol	P35372	OPRM1	SwissTargetPrediction
α-Terpineol	P09874	PARP1	SwissTargetPrediction
α-Terpineol	Q08499	PDE4D	SwissTargetPrediction
α-Terpineol	P06401	PGR	SwissTargetPrediction
α-Terpineol	P04054	PLA2G1B	SwissTargetPrediction
α-Terpineol	Q07869	PPARA	SwissTargetPrediction
α-Terpineol	Q03181	PPARD	SwissTargetPrediction
α-Terpineol	P25105	PTAFR	SwissTargetPrediction
α-Terpineol	P23219	PTGS1	SwissTargetPrediction
α-Terpineol	P35354	PTGS2	SwissTargetPrediction
α-Terpineol	P18031	PTPN1	SwissTargetPrediction
α-Terpineol	Q06124	PTPN11	SwissTargetPrediction
α-Terpineol	P17706	PTPN2	SwissTargetPrediction
α-Terpineol	P29350	PTPN6	SwissTargetPrediction
α-Terpineol	P10586	PTPRF	SwissTargetPrediction
α-Terpineol	P35398	RORA	SwissTargetPrediction
α-Terpineol	P51449	RORC	SwissTargetPrediction
α-Terpineol	O00767	SCD	SwissTargetPrediction
α-Terpineol	Q14524	SCN5A	SwissTargetPrediction
α-Terpineol	Q15858	SCN9A	SwissTargetPrediction
α-Terpineol	P04278	SHBG	SwissTargetPrediction
α-Terpineol	Q99720	SIGMAR1	SwissTargetPrediction
α-Terpineol	P23975	SLC6A2	SwissTargetPrediction
α-Terpineol	Q01959	SLC6A3	SwissTargetPrediction
α-Terpineol	P31645	SLC6A4	SwissTargetPrediction
α-Terpineol	Q9NYA1	SPHK1	SwissTargetPrediction
α-Terpineol	Q9NRA0	SPHK2	SwissTargetPrediction
α-Terpineol	Q14534	SQLE	SwissTargetPrediction
α-Terpineol	Q12772	SREBF2	SwissTargetPrediction
α-Terpineol	Q9P289	STK26	SwissTargetPrediction
α-Terpineol	Q13188	STK3	SwissTargetPrediction
α-Terpineol	P63316	TNNC1	SwissTargetPrediction
α-Terpineol	P19429	TNNI3	SwissTargetPrediction
α-Terpineol	P45379	TNNT2	SwissTargetPrediction
α-Terpineol	P11387	TOP1	SwissTargetPrediction
α-Terpineol	Q7Z2W7	TRPM8	SwissTargetPrediction
α-Terpineol	Q8NET8	TRPV3	SwissTargetPrediction
α-Terpineol	P04818	TYMS	SwissTargetPrediction

α-Terpineol	Q9UKP6	UTS2R	SwissTargetPrediction
α-Terpineol	P28566	HTR1E	TargetNet
α-Terpineol	P28335	HTR2C	TargetNet
α-Terpineol	P46098	HTR3A	TargetNet
α-Terpineol	P22303	ACHE	TargetNet
α-Terpineol	P08913	ADRA2A	TargetNet
α-Terpineol	P18089	ADRA2B	TargetNet
α-Terpineol	P18825	ADRA2C	TargetNet
α-Terpineol	P18825	ADRA2C	TargetNet
α-Terpineol	P05067	APP	TargetNet
α-Terpineol	P10275	AR	TargetNet
α-Terpineol	P10275	AR	TargetNet
α-Terpineol	P16050	ALOX15	TargetNet
α-Terpineol	P09917	ALOX5	TargetNet
α-Terpineol	P35869	AHR	TargetNet
α-Terpineol	Q16548	BCL2A1	TargetNet
α-Terpineol	P21554	CNR1	TargetNet
α-Terpineol	P34972	CNR2	TargetNet
α-Terpineol	P34972	CNR2	TargetNet
α-Terpineol	O43570	CA12	TargetNet
α-Terpineol	Q9ULX7	CA14	TargetNet
α-Terpineol	P35218	CA5A	TargetNet
α-Terpineol	Q9Y2D0	CA5B	TargetNet
α-Terpineol	P23280	CA6	TargetNet
α-Terpineol	P43166	CA7	TargetNet
α-Terpineol	O00748	CES2	TargetNet
α-Terpineol	P28845	HSD11B1	TargetNet
α-Terpineol	P24468	NR2F2	TargetNet
α-Terpineol	P05177	CYP1A2	TargetNet
α-Terpineol	P33261	CYP2C19	TargetNet
α-Terpineol	P10635	CYP2D6	TargetNet
α-Terpineol	P21728	DRD1	TargetNet
α-Terpineol	P31941	APOBEC3A	TargetNet
α-Terpineol	Q9HC16	APOBEC3G	TargetNet
α-Terpineol	P03372	ESR1	TargetNet
α-Terpineol	Q92731	ESR2	TargetNet
α-Terpineol	P14324	FDPS	TargetNet
α-Terpineol	Q9HC97	GPR35	TargetNet
α-Terpineol	Q05469	LIPE	TargetNet
α-Terpineol	P23141	CES1	TargetNet
α-Terpineol	P14174	MIF	TargetNet
α-Terpineol	P08235	NR3C2	TargetNet
α-Terpineol	P11229	CHRM1	TargetNet

α-Terpineol	P11229	CHRM1	TargetNet
α-Terpineol	P08172	CHRM2	TargetNet
α-Terpineol	P08172	CHRM2	TargetNet
α-Terpineol	P20309	CHRM3	TargetNet
α-Terpineol	P08173	CHRM4	TargetNet
α-Terpineol	P08912	CHRM5	TargetNet
α-Terpineol	P36544	CHRNA7	TargetNet
α-Terpineol	P29475	NOS1	TargetNet
α-Terpineol	P29474	NOS3	TargetNet
α-Terpineol	P35228	NOS2	TargetNet
α-Terpineol	P35228	NOS2	TargetNet
α-Terpineol	P35398	RORA	TargetNet
α-Terpineol	Q13133	NR1H3	TargetNet
α-Terpineol	P06401	PGR	TargetNet
α-Terpineol	P23219	PTGS1	TargetNet
α-Terpineol	O43353	RIPK2	TargetNet
α-Terpineol	P10276	RARA	TargetNet
α-Terpineol	Q99720	SIGMAR1	TargetNet
α-Terpineol	Q01959	SLC6A3	TargetNet
α-Terpineol	Q01959	SLC6A3	TargetNet
α-Terpineol	P23975	SLC6A2	TargetNet
α-Terpineol	P05093	CYP17A1	TargetNet
α-Terpineol	Q9NR96	TLR9	TargetNet
α-Terpineol	Q96RJ0	TAAR1	TargetNet
α-Terpineol	Q04206	RELA	TargetNet
α-Zingiberene	P02511	CRYAB	SEA
α-Zingiberene	P21757	MSR1	SEA
α-Zingiberene	P28566	HTR1E	TargetNet
α-Zingiberene	P18825	ADRA2C	TargetNet
α-Zingiberene	P16050	ALOX15	TargetNet
α-Zingiberene	Q16548	BCL2A1	TargetNet
α-Zingiberene	P34972	CNR2	TargetNet
α-Zingiberene	O00748	CES2	TargetNet
α-Zingiberene	P26358	DNMT1	TargetNet
α-Zingiberene	P31941	APOBEC3A	TargetNet
α-Zingiberene	Q9HC16	APOBEC3G	TargetNet
α-Zingiberene	P30305	CDC25B	TargetNet
α-Zingiberene	P11229	CHRM1	TargetNet
α-Zingiberene	P08172	CHRM2	TargetNet
α-Zingiberene	P08173	CHRM4	TargetNet
α-Zingiberene	P35228	NOS2	TargetNet
α-Zingiberene	P23219	PTGS1	TargetNet
α-Zingiberene	O43353	RIPK2	TargetNet

α-Zingiberene	O95136	S1PR2	TargetNet
α-Zingiberene	P05093	CYP17A1	TargetNet
α-Zingiberene	Q9NR96	TLR9	TargetNet
β-Bisabolene	P28566	HTR1E	TargetNet
β-Bisabolene	P22303	ACHE	TargetNet
β-Bisabolene	P08913	ADRA2A	TargetNet
β-Bisabolene	P18089	ADRA2B	TargetNet
β-Bisabolene	P18825	ADRA2C	TargetNet
β-Bisabolene	P18825	ADRA2C	TargetNet
β-Bisabolene	P21397	MAOA	TargetNet
β-Bisabolene	P16050	ALOX15	TargetNet
β-Bisabolene	Q16548	BCL2A1	TargetNet
β-Bisabolene	P34972	CNR2	TargetNet
β-Bisabolene	P34972	CNR2	TargetNet
β-Bisabolene	P43166	CA7	TargetNet
β-Bisabolene	O00748	CES2	TargetNet
β-Bisabolene	P26358	DNMT1	TargetNet
β-Bisabolene	P31941	APOBEC3A	TargetNet
β-Bisabolene	Q9HC16	APOBEC3G	TargetNet
β-Bisabolene	Q92731	ESR2	TargetNet
β-Bisabolene	Q9HC97	GPR35	TargetNet
β-Bisabolene	P30305	CDC25B	TargetNet
β-Bisabolene	P11229	CHRM1	TargetNet
β-Bisabolene	P08172	CHRM2	TargetNet
β-Bisabolene	P08173	CHRM4	TargetNet
β-Bisabolene	P29474	NOS3	TargetNet
β-Bisabolene	P35228	NOS2	TargetNet
β-Bisabolene	Q00G26	PLIN5	TargetNet
β-Bisabolene	O43353	RIPK2	TargetNet
β-Bisabolene	P10276	RARA	TargetNet
β-Bisabolene	P13631	RARG	TargetNet
β-Bisabolene	P19793	RXRA	TargetNet
β-Bisabolene	P28702	RXRB	TargetNet
β-Bisabolene	P48443	RXRG	TargetNet
β-Bisabolene	Q99720	SIGMAR1	TargetNet
β-Bisabolene	Q99720	SIGMAR1	TargetNet
β-Bisabolene	Q01959	SLC6A3	TargetNet
β-Bisabolene	Q9NR96	TLR9	TargetNet
β-Elemene	P35869	AHR	SwissTargetPrediction
β-Elemene	P34972	CNR2	SwissTargetPrediction
β-Elemene	P49682	CXCR3	SwissTargetPrediction
β-Elemene	P11511	CYP19A1	SwissTargetPrediction
β-Elemene	P05177	CYP1A2	SwissTargetPrediction

β-Elemene	P28845	HSD11B1	SwissTargetPrediction
β-Elemene	P28223	HTR2A	SwissTargetPrediction
β-Elemene	P27338	MAOB	SwissTargetPrediction
β-Elemene	Q99685	MGLL	SwissTargetPrediction
β-Elemene	Q13133	NR1H3	SwissTargetPrediction
β-Elemene	Q14994	NR1I3	SwissTargetPrediction
β-Elemene	Q07869	PPARA	SwissTargetPrediction
β-Elemene	P23219	PTGS1	SwissTargetPrediction
β-Elemene	P18031	PTPN1	SwissTargetPrediction
β-Elemene	P04278	SHBG	SwissTargetPrediction
β-Elemene	P16662	UGT2B7	SwissTargetPrediction
β-Elemene	P28566	HTR1E	TargetNet
β-Elemene	P47898	HTR5A	TargetNet
β-Elemene	P22303	ACHE	TargetNet
β-Elemene	P22303	ACHE	TargetNet
β-Elemene	P08913	ADRA2A	TargetNet
β-Elemene	P18089	ADRA2B	TargetNet
β-Elemene	P18825	ADRA2C	TargetNet
β-Elemene	P18825	ADRA2C	TargetNet
β-Elemene	P10275	AR	TargetNet
β-Elemene	P10275	AR	TargetNet
β-Elemene	P16050	ALOX15	TargetNet
β-Elemene	Q16548	BCL2A1	TargetNet
β-Elemene	P34972	CNR2	TargetNet
β-Elemene	Q9ULX7	CA14	TargetNet
β-Elemene	P22748	CA4	TargetNet
β-Elemene	P35218	CA5A	TargetNet
β-Elemene	Q9Y2D0	CA5B	TargetNet
β-Elemene	P23280	CA6	TargetNet
β-Elemene	P43166	CA7	TargetNet
β-Elemene	P55211	CASP9	TargetNet
β-Elemene	O00748	CES2	TargetNet
β-Elemene	P28845	HSD11B1	TargetNet
β-Elemene	P28845	HSD11B1	TargetNet
β-Elemene	P24468	NR2F2	TargetNet
β-Elemene	P21728	DRD1	TargetNet
β-Elemene	P21918	DRD5	TargetNet
β-Elemene	Q9UHL4	DPP7	TargetNet
β-Elemene	P27487	DPP4	TargetNet
β-Elemene	Q6V1X1	DPP8	TargetNet
β-Elemene	P31941	APOBEC3A	TargetNet
β-Elemene	Q9HC16	APOBEC3G	TargetNet
β-Elemene	P51452	DUSP3	TargetNet

β-Elemene	P03372	ESR1	TargetNet
β-Elemene	Q92731	ESR2	TargetNet
β-Elemene	Q9HC97	GPR35	TargetNet
β-Elemene	P30305	CDC25B	TargetNet
β-Elemene	P11229	CHRM1	TargetNet
β-Elemene	P11229	CHRM1	TargetNet
β-Elemene	P08172	CHRM2	TargetNet
β-Elemene	P08172	CHRM2	TargetNet
β-Elemene	P20309	CHRM3	TargetNet
β-Elemene	P08173	CHRM4	TargetNet
β-Elemene	P43681	CHRNA4	TargetNet
β-Elemene	P43681	CHRNA4	TargetNet
β-Elemene	P36544	CHRNA7	TargetNet
β-Elemene	P29474	NOS3	TargetNet
β-Elemene	P35228	NOS2	TargetNet
β-Elemene	O43353	RIPK2	TargetNet
β-Elemene	Q99720	SIGMAR1	TargetNet
β-Elemene	Q99720	SIGMAR1	TargetNet
β-Elemene	Q01959	SLC6A3	TargetNet
β-Elemene	Q01959	SLC6A3	TargetNet
β-Elemene	P23975	SLC6A2	TargetNet
β-Elemene	O95136	S1PR2	TargetNet
β-Elemene	P05093	CYP17A1	TargetNet
β-Elemene	P37058	HSD17B3	TargetNet
β-Elemene	Q9NR96	TLR9	TargetNet
β-Phellandrene	P22303	ACHE	SwissTargetPrediction
β-Phellandrene	P30542	ADORA1	SwissTargetPrediction
β-Phellandrene	P29274	ADORA2A	SwissTargetPrediction
β-Phellandrene	P0DMS8	ADORA3	SwissTargetPrediction
β-Phellandrene	P18089	ADRA2B	SwissTargetPrediction
β-Phellandrene	P10275	AR	SwissTargetPrediction
β-Phellandrene	P06276	BCHE	SwissTargetPrediction
β-Phellandrene	P30304	CDC25A	SwissTargetPrediction
β-Phellandrene	P08172	CHRM2	SwissTargetPrediction
β-Phellandrene	P21554	CNR1	SwissTargetPrediction
β-Phellandrene	P34972	CNR2	SwissTargetPrediction
β-Phellandrene	P07339	CTSD	SwissTargetPrediction
β-Phellandrene	P05093	CYP17A1	SwissTargetPrediction
β-Phellandrene	P11511	CYP19A1	SwissTargetPrediction
β-Phellandrene	P33261	CYP2C19	SwissTargetPrediction
β-Phellandrene	Q16850	CYP51A1	SwissTargetPrediction
β-Phellandrene	P03372	ESR1	SwissTargetPrediction
β-Phellandrene	Q92731	ESR2	SwissTargetPrediction

β-Phellandrene	O00519	FAAH	SwissTargetPrediction
β-Phellandrene	P07148	FABP1	SwissTargetPrediction
β-Phellandrene	P05413	FABP3	SwissTargetPrediction
β-Phellandrene	P15090	FABP4	SwissTargetPrediction
β-Phellandrene	Q01469	FABP5	SwissTargetPrediction
β-Phellandrene	P23415	GLRA1	SwissTargetPrediction
β-Phellandrene	P04035	HMGCR	SwissTargetPrediction
β-Phellandrene	P37058	HSD17B3	SwissTargetPrediction
β-Phellandrene	P41595	HTR2B	SwissTargetPrediction
β-Phellandrene	P48449	LSS	SwissTargetPrediction
β-Phellandrene	P28482	MAPK1	SwissTargetPrediction
β-Phellandrene	Q16539	MAPK14	SwissTargetPrediction
β-Phellandrene	Q9UHC9	NPC1L1	SwissTargetPrediction
β-Phellandrene	Q13133	NR1H3	SwissTargetPrediction
β-Phellandrene	O75469	NR1I2	SwissTargetPrediction
β-Phellandrene	Q14994	NR1I3	SwissTargetPrediction
β-Phellandrene	P04150	NR3C1	SwissTargetPrediction
β-Phellandrene	P08235	NR3C2	SwissTargetPrediction
β-Phellandrene	P06401	PGR	SwissTargetPrediction
β-Phellandrene	P06746	POLB	SwissTargetPrediction
β-Phellandrene	Q07869	PPARA	SwissTargetPrediction
β-Phellandrene	Q03181	PPARD	SwissTargetPrediction
β-Phellandrene	P37231	PPARG	SwissTargetPrediction
β-Phellandrene	P23219	PTGS1	SwissTargetPrediction
β-Phellandrene	P18031	PTPN1	SwissTargetPrediction
β-Phellandrene	P17706	PTPN2	SwissTargetPrediction
β-Phellandrene	P29350	PTPN6	SwissTargetPrediction
β-Phellandrene	P10276	RARA	SwissTargetPrediction
β-Phellandrene	P10826	RARB	SwissTargetPrediction
β-Phellandrene	P13631	RARG	SwissTargetPrediction
β-Phellandrene	P02753	RBP4	SwissTargetPrediction
β-Phellandrene	P35398	RORA	SwissTargetPrediction
β-Phellandrene	Q92753	RORB	SwissTargetPrediction
β-Phellandrene	P51449	RORC	SwissTargetPrediction
β-Phellandrene	P19793	RXRA	SwissTargetPrediction
β-Phellandrene	P28702	RXRB	SwissTargetPrediction
β-Phellandrene	P48443	RXRG	SwissTargetPrediction
β-Phellandrene	P08185	SERPINA6	SwissTargetPrediction
β-Phellandrene	P04278	SHBG	SwissTargetPrediction
β-Phellandrene	Q99720	SIGMAR1	SwissTargetPrediction
β-Phellandrene	P23975	SLC6A2	SwissTargetPrediction
β-Phellandrene	P31645	SLC6A4	SwissTargetPrediction
β-Phellandrene	Q14534	SQLE	SwissTargetPrediction

β-Phellandrene	P18405	SRD5A1	SwissTargetPrediction
β-Phellandrene	P31213	SRD5A2	SwissTargetPrediction
β-Phellandrene	Q12772	SREBF2	SwissTargetPrediction
β-Phellandrene	O14746	TERT	SwissTargetPrediction
β-Phellandrene	P01375	TNF	SwissTargetPrediction
β-Phellandrene	P11388	TOP2A	SwissTargetPrediction
β-Phellandrene	Q8NER1	TRPV1	SwissTargetPrediction
β-Phellandrene	P16662	UGT2B7	SwissTargetPrediction
β-Phellandrene	P11473	VDR	SwissTargetPrediction
β-Phellandrene	P28566	HTR1E	TargetNet
β-Phellandrene	P28223	HTR2A	TargetNet
β-Phellandrene	P28223	HTR2A	TargetNet
β-Phellandrene	P28335	HTR2C	TargetNet
β-Phellandrene	P28335	HTR2C	TargetNet
β-Phellandrene	P47898	HTR5A	TargetNet
β-Phellandrene	P08913	ADRA2A	TargetNet
β-Phellandrene	P18089	ADRA2B	TargetNet
β-Phellandrene	P18825	ADRA2C	TargetNet
β-Phellandrene	P18825	ADRA2C	TargetNet
β-Phellandrene	P21397	MAOA	TargetNet
β-Phellandrene	P15144	ANPEP	TargetNet
β-Phellandrene	P10275	AR	TargetNet
β-Phellandrene	P09917	ALOX5	TargetNet
β-Phellandrene	P11511	CYP19A1	TargetNet
β-Phellandrene	Q16548	BCL2A1	TargetNet
β-Phellandrene	P34972	CNR2	TargetNet
β-Phellandrene	O43570	CA12	TargetNet
β-Phellandrene	Q9ULX7	CA14	TargetNet
β-Phellandrene	P22748	CA4	TargetNet
β-Phellandrene	P35218	CA5A	TargetNet
β-Phellandrene	Q9Y2D0	CA5B	TargetNet
β-Phellandrene	P23280	CA6	TargetNet
β-Phellandrene	P43166	CA7	TargetNet
β-Phellandrene	Q16790	CA9	TargetNet
β-Phellandrene	P55211	CASP9	TargetNet
β-Phellandrene	O00748	CES2	TargetNet
β-Phellandrene	P24468	NR2F2	TargetNet
β-Phellandrene	P21728	DRD1	TargetNet
β-Phellandrene	P21728	DRD1	TargetNet
β-Phellandrene	P31941	APOBEC3A	TargetNet
β-Phellandrene	Q9HC16	APOBEC3G	TargetNet
β-Phellandrene	P51452	DUSP3	TargetNet
β-Phellandrene	P42262	GRIA2	TargetNet

β-Phellandrene	P23141	CES1	TargetNet
β-Phellandrene	P14174	MIF	TargetNet
β-Phellandrene	Q99685	MGLL	TargetNet
β-Phellandrene	P30305	CDC25B	TargetNet
β-Phellandrene	P11229	CHRM1	TargetNet
β-Phellandrene	P11229	CHRM1	TargetNet
β-Phellandrene	P08172	CHRM2	TargetNet
β-Phellandrene	P08173	CHRM4	TargetNet
β-Phellandrene	P43681	CHRNA4	TargetNet
β-Phellandrene	P29475	NOS1	TargetNet
β-Phellandrene	P29474	NOS3	TargetNet
β-Phellandrene	P35228	NOS2	TargetNet
β-Phellandrene	P35398	RORA	TargetNet
β-Phellandrene	P06401	PGR	TargetNet
β-Phellandrene	P23219	PTGS1	TargetNet
β-Phellandrene	O43353	RIPK2	TargetNet
β-Phellandrene	Q99720	SIGMAR1	TargetNet
β-Phellandrene	Q01959	SLC6A3	TargetNet
β-Phellandrene	Q01959	SLC6A3	TargetNet
β-Phellandrene	P23975	SLC6A2	TargetNet
β-Phellandrene	O95136	S1PR2	TargetNet
β-Phellandrene	P05093	CYP17A1	TargetNet
β-Phellandrene	Q9NR96	TLR9	TargetNet
β-Phellandrene	Q96RJ0	TAAR1	TargetNet
β-Phellandrene	Q04206	RELA	TargetNet
β-Pinene	P22303	ACHE	SwissTargetPrediction
β-Pinene	P10275	AR	SwissTargetPrediction
β-Pinene	P08172	CHRM2	SwissTargetPrediction
β-Pinene	P34972	CNR2	SwissTargetPrediction
β-Pinene	P11511	CYP19A1	SwissTargetPrediction
β-Pinene	P33261	CYP2C19	SwissTargetPrediction
β-Pinene	P03372	ESR1	SwissTargetPrediction
β-Pinene	O00519	FAAH	SwissTargetPrediction
β-Pinene	P28845	HSD11B1	SwissTargetPrediction
β-Pinene	Q13133	NR1H3	SwissTargetPrediction
β-Pinene	Q14994	NR1I3	SwissTargetPrediction
β-Pinene	Q07869	PPARA	SwissTargetPrediction
β-Pinene	P23219	PTGS1	SwissTargetPrediction
β-Pinene	P18031	PTPN1	SwissTargetPrediction
β-Pinene	P04278	SHBG	SwissTargetPrediction
β-Pinene	Q9GZV3	SLC5A7	SwissTargetPrediction
β-Pinene	P23975	SLC6A2	SwissTargetPrediction
β-Pinene	P31645	SLC6A4	SwissTargetPrediction

β-Pinene	Q8NER1	TRPV1	SwissTargetPrediction
β-Pinene	P16662	UGT2B7	SwissTargetPrediction
β-Pinene	P18405	SRD5A1	TargetNet
β-Pinene	P31213	SRD5A2	TargetNet
β-Pinene	P28566	HTR1E	TargetNet
β-Pinene	P28335	HTR2C	TargetNet
β-Pinene	P28335	HTR2C	TargetNet
β-Pinene	P47898	HTR5A	TargetNet
β-Pinene	P22303	ACHE	TargetNet
β-Pinene	P22303	ACHE	TargetNet
β-Pinene	P08913	ADRA2A	TargetNet
β-Pinene	P18089	ADRA2B	TargetNet
β-Pinene	P18825	ADRA2C	TargetNet
β-Pinene	P18825	ADRA2C	TargetNet
β-Pinene	P21397	MAOA	TargetNet
β-Pinene	P21397	MAOA	TargetNet
β-Pinene	P27338	MAOB	TargetNet
β-Pinene	P05067	APP	TargetNet
β-Pinene	P10275	AR	TargetNet
β-Pinene	P10275	AR	TargetNet
β-Pinene	P09917	ALOX5	TargetNet
β-Pinene	P11511	CYP19A1	TargetNet
β-Pinene	P35869	AHR	TargetNet
β-Pinene	Q16548	BCL2A1	TargetNet
β-Pinene	P34972	CNR2	TargetNet
β-Pinene	P34972	CNR2	TargetNet
β-Pinene	P00915	CA1	TargetNet
β-Pinene	O43570	CA12	TargetNet
β-Pinene	Q8N1Q1	CA13	TargetNet
β-Pinene	Q9ULX7	CA14	TargetNet
β-Pinene	P00918	CA2	TargetNet
β-Pinene	P22748	CA4	TargetNet
β-Pinene	P35218	CA5A	TargetNet
β-Pinene	Q9Y2D0	CA5B	TargetNet
β-Pinene	P23280	CA6	TargetNet
β-Pinene	P43166	CA7	TargetNet
β-Pinene	Q16790	CA9	TargetNet
β-Pinene	P68400	CSNK2A1	TargetNet
β-Pinene	P55211	CASP9	TargetNet
β-Pinene	Q14432	PDE3A	TargetNet
β-Pinene	Q494W8	CHRFAM7A	TargetNet
β-Pinene	O00748	CES2	TargetNet
β-Pinene	P28845	HSD11B1	TargetNet

β-Pinene	P28845	HSD11B1	TargetNet
β-Pinene	P24468	NR2F2	TargetNet
β-Pinene	P05177	CYP1A2	TargetNet
β-Pinene	P21728	DRD1	TargetNet
β-Pinene	P21728	DRD1	TargetNet
β-Pinene	P21918	DRD5	TargetNet
β-Pinene	P31941	APOBEC3A	TargetNet
β-Pinene	Q9HC16	APOBEC3G	TargetNet
β-Pinene	P51452	DUSP3	TargetNet
β-Pinene	Q92731	ESR2	TargetNet
β-Pinene	P42262	GRIA2	TargetNet
β-Pinene	Q9HC97	GPR35	TargetNet
β-Pinene	P23141	CES1	TargetNet
β-Pinene	P14174	MIF	TargetNet
β-Pinene	Q14416	GRM2	TargetNet
β-Pinene	Q99685	MGLL	TargetNet
β-Pinene	P30305	CDC25B	TargetNet
β-Pinene	P11229	CHRM1	TargetNet
β-Pinene	P11229	CHRM1	TargetNet
β-Pinene	P08172	CHRM2	TargetNet
β-Pinene	P08172	CHRM2	TargetNet
β-Pinene	P20309	CHRM3	TargetNet
β-Pinene	P08173	CHRM4	TargetNet
β-Pinene	P08912	CHRM5	TargetNet
β-Pinene	P43681	CHRNA4	TargetNet
β-Pinene	P43681	CHRNA4	TargetNet
β-Pinene	P36544	CHRNA7	TargetNet
β-Pinene	P36544	CHRNA7	TargetNet
β-Pinene	P29475	NOS1	TargetNet
β-Pinene	P29474	NOS3	TargetNet
β-Pinene	P35228	NOS2	TargetNet
β-Pinene	P35228	NOS2	TargetNet
β-Pinene	P35398	RORA	TargetNet
β-Pinene	O60240	PLIN1	TargetNet
β-Pinene	Q00G26	PLIN5	TargetNet
β-Pinene	P11086	PNMT	TargetNet
β-Pinene	P06401	PGR	TargetNet
β-Pinene	P23219	PTGS1	TargetNet
β-Pinene	P35354	PTGS2	TargetNet
β-Pinene	O43353	RIPK2	TargetNet
β-Pinene	P10276	RARA	TargetNet
β-Pinene	P10826	RARB	TargetNet
β-Pinene	P13631	RARG	TargetNet

β-Pinene	Q99720	SIGMAR1	TargetNet
β-Pinene	Q99720	SIGMAR1	TargetNet
β-Pinene	Q01959	SLC6A3	TargetNet
β-Pinene	Q01959	SLC6A3	TargetNet
β-Pinene	P23975	SLC6A2	TargetNet
β-Pinene	P31645	SLC6A4	TargetNet
β-Pinene	O95136	S1PR2	TargetNet
β-Pinene	P05093	CYP17A1	TargetNet
β-Pinene	P08842	STS	TargetNet
β-Pinene	P37058	HSD17B3	TargetNet
β-Pinene	Q9NR96	TLR9	TargetNet
β-Pinene	Q04206	RELA	TargetNet
β-Sesquiphellandrene	P02511	CRYAB	SEA
β-Sesquiphellandrene	P21757	MSR1	SEA
β-Sesquiphellandrene	P08913	ADRA2A	TargetNet
β-Sesquiphellandrene	P18089	ADRA2B	TargetNet
β-Sesquiphellandrene	P18825	ADRA2C	TargetNet
β-Sesquiphellandrene	P18825	ADRA2C	TargetNet
β-Sesquiphellandrene	P26358	DNMT1	TargetNet
β-Sesquiphellandrene	P31941	APOBEC3A	TargetNet
β-Sesquiphellandrene	Q9HC16	APOBEC3G	TargetNet
β-Sesquiphellandrene	P30305	CDC25B	TargetNet
β-Sesquiphellandrene	P11229	CHRM1	TargetNet
β-Sesquiphellandrene	P08172	CHRM2	TargetNet
β-Sesquiphellandrene	P08173	CHRM4	TargetNet
β-Sesquiphellandrene	P43681	CHRNA4	TargetNet
β-Sesquiphellandrene	P29474	NOS3	TargetNet
β-Sesquiphellandrene	P35228	NOS2	TargetNet
β-Sesquiphellandrene	P06401	PGR	TargetNet
β-Sesquiphellandrene	O43353	RIPK2	TargetNet
β-Sesquiphellandrene	Q9NR96	TLR9	TargetNet
β-Zingiberene	P02511	CRYAB	SEA
β-Zingiberene	P21757	MSR1	SEA
β-Zingiberene	P08913	ADRA2A	TargetNet
β-Zingiberene	P18089	ADRA2B	TargetNet
β-Zingiberene	P18825	ADRA2C	TargetNet
β-Zingiberene	P18825	ADRA2C	TargetNet
β-Zingiberene	P26358	DNMT1	TargetNet
β-Zingiberene	P31941	APOBEC3A	TargetNet
β-Zingiberene	Q9HC16	APOBEC3G	TargetNet
β-Zingiberene	P30305	CDC25B	TargetNet
β-Zingiberene	P11229	CHRM1	TargetNet
β-Zingiberene	P08172	CHRM2	TargetNet

β-Zingiberene	P08173	CHRM4	TargetNet
β-Zingiberene	P43681	CHRNA4	TargetNet
β-Zingiberene	P29474	NOS3	TargetNet
β-Zingiberene	P35228	NOS2	TargetNet
β-Zingiberene	P06401	PGR	TargetNet
β-Zingiberene	O43353	RIPK2	TargetNet
β-Zingiberene	Q9NR96	TLR9	TargetNet
γ-Selinene	P22303	ACHE	SwissTargetPrediction
γ-Selinene	P30542	ADORA1	SwissTargetPrediction
γ-Selinene	P29274	ADORA2A	SwissTargetPrediction
γ-Selinene	P0DMS8	ADORA3	SwissTargetPrediction
γ-Selinene	P10275	AR	SwissTargetPrediction
γ-Selinene	P54707	ATP12A	SwissTargetPrediction
γ-Selinene	P06276	BCHE	SwissTargetPrediction
γ-Selinene	P51681	CCR5	SwissTargetPrediction
γ-Selinene	P30304	CDC25A	SwissTargetPrediction
γ-Selinene	P30305	CDC25B	SwissTargetPrediction
γ-Selinene	P23141	CES1	SwissTargetPrediction
γ-Selinene	O00748	CES2	SwissTargetPrediction
γ-Selinene	P08172	CHRM2	SwissTargetPrediction
γ-Selinene	P21554	CNR1	SwissTargetPrediction
γ-Selinene	P34972	CNR2	SwissTargetPrediction
γ-Selinene	P07339	CTSD	SwissTargetPrediction
γ-Selinene	P49682	CXCR3	SwissTargetPrediction
γ-Selinene	P05093	CYP17A1	SwissTargetPrediction
γ-Selinene	P11511	CYP19A1	SwissTargetPrediction
γ-Selinene	P33261	CYP2C19	SwissTargetPrediction
γ-Selinene	Q16850	CYP51A1	SwissTargetPrediction
γ-Selinene	P03372	ESR1	SwissTargetPrediction
γ-Selinene	Q92731	ESR2	SwissTargetPrediction
γ-Selinene	O00519	FAAH	SwissTargetPrediction
γ-Selinene	P07148	FABP1	SwissTargetPrediction
γ-Selinene	P05413	FABP3	SwissTargetPrediction
γ-Selinene	P15090	FABP4	SwissTargetPrediction
γ-Selinene	Q01469	FABP5	SwissTargetPrediction
γ-Selinene	P08151	GLI1	SwissTargetPrediction
γ-Selinene	P10070	GLI2	SwissTargetPrediction
γ-Selinene	P04035	HMGCR	SwissTargetPrediction
γ-Selinene	P28845	HSD11B1	SwissTargetPrediction
γ-Selinene	P37058	HSD17B3	SwissTargetPrediction
γ-Selinene	P28223	HTR2A	SwissTargetPrediction
γ-Selinene	P27338	MAOB	SwissTargetPrediction
γ-Selinene	Q99685	MGLL	SwissTargetPrediction

γ-Selinene	Q9UHC9	NPC1L1	SwissTargetPrediction
γ-Selinene	Q13133	NR1H3	SwissTargetPrediction
γ-Selinene	O75469	NR1I2	SwissTargetPrediction
γ-Selinene	Q14994	NR1I3	SwissTargetPrediction
γ-Selinene	P04150	NR3C1	SwissTargetPrediction
γ-Selinene	P08235	NR3C2	SwissTargetPrediction
γ-Selinene	P06401	PGR	SwissTargetPrediction
γ-Selinene	Q07869	PPARA	SwissTargetPrediction
γ-Selinene	Q03181	PPARD	SwissTargetPrediction
γ-Selinene	P37231	PPARG	SwissTargetPrediction
γ-Selinene	P48147	PREP	SwissTargetPrediction
γ-Selinene	O14684	PTGES	SwissTargetPrediction
γ-Selinene	P23219	PTGS1	SwissTargetPrediction
γ-Selinene	P35354	PTGS2	SwissTargetPrediction
γ-Selinene	P18031	PTPN1	SwissTargetPrediction
γ-Selinene	P17706	PTPN2	SwissTargetPrediction
γ-Selinene	P29350	PTPN6	SwissTargetPrediction
γ-Selinene	P51449	RORC	SwissTargetPrediction
γ-Selinene	O00767	SCD	SwissTargetPrediction
γ-Selinene	P08185	SERPINA6	SwissTargetPrediction
γ-Selinene	P04278	SHBG	SwissTargetPrediction
γ-Selinene	Q99720	SIGMAR1	SwissTargetPrediction
γ-Selinene	P23975	SLC6A2	SwissTargetPrediction
γ-Selinene	P31645	SLC6A4	SwissTargetPrediction
γ-Selinene	Q14534	SQLE	SwissTargetPrediction
γ-Selinene	P18405	SRD5A1	SwissTargetPrediction
γ-Selinene	P31213	SRD5A2	SwissTargetPrediction
γ-Selinene	Q12772	SREBF2	SwissTargetPrediction
γ-Selinene	O14746	TERT	SwissTargetPrediction
γ-Selinene	P11387	TOP1	SwissTargetPrediction
γ-Selinene	O75762	TRPA1	SwissTargetPrediction
γ-Selinene	Q8NER1	TRPV1	SwissTargetPrediction
γ-Selinene	P16662	UGT2B7	SwissTargetPrediction
γ-Selinene	P11473	VDR	SwissTargetPrediction
γ-Selinene	P28566	HTR1E	TargetNet
γ-Selinene	P28335	HTR2C	TargetNet
γ-Selinene	P47898	HTR5A	TargetNet
γ-Selinene	P22303	ACHE	TargetNet
γ-Selinene	P08913	ADRA2A	TargetNet
γ-Selinene	P18089	ADRA2B	TargetNet
γ-Selinene	P18825	ADRA2C	TargetNet
γ-Selinene	P18825	ADRA2C	TargetNet
γ-Selinene	P21397	MAOA	TargetNet

γ-Selinene	P27338	MAOB	TargetNet
γ-Selinene	P10275	AR	TargetNet
γ-Selinene	P35869	AHR	TargetNet
γ-Selinene	Q16548	BCL2A1	TargetNet
γ-Selinene	P35218	CA5A	TargetNet
γ-Selinene	Q9Y2D0	CA5B	TargetNet
γ-Selinene	P23280	CA6	TargetNet
γ-Selinene	P43166	CA7	TargetNet
γ-Selinene	Q9GZU7	CTDSP1	TargetNet
γ-Selinene	O00748	CES2	TargetNet
γ-Selinene	P24468	NR2F2	TargetNet
γ-Selinene	P10635	CYP2D6	TargetNet
γ-Selinene	P21728	DRD1	TargetNet
γ-Selinene	P21918	DRD5	TargetNet
γ-Selinene	P31941	APOBEC3A	TargetNet
γ-Selinene	Q9HC16	APOBEC3G	TargetNet
γ-Selinene	P51452	DUSP3	TargetNet
γ-Selinene	Q9HC97	GPR35	TargetNet
γ-Selinene	Q9Y5N1	HRH3	TargetNet
γ-Selinene	P41145	OPRK1	TargetNet
γ-Selinene	P14174	MIF	TargetNet
γ-Selinene	Q99685	MGLL	TargetNet
γ-Selinene	P08173	CHRM4	TargetNet
γ-Selinene	P35372	OPRM1	TargetNet
γ-Selinene	P43681	CHRNA4	TargetNet
γ-Selinene	P43681	CHRNA4	TargetNet
γ-Selinene	P29474	NOS3	TargetNet
γ-Selinene	P35228	NOS2	TargetNet
γ-Selinene	P35398	RORA	TargetNet
γ-Selinene	P06401	PGR	TargetNet
γ-Selinene	P10276	RARA	TargetNet
γ-Selinene	P13631	RARG	TargetNet
γ-Selinene	Q01959	SLC6A3	TargetNet
γ-Selinene	P23975	SLC6A2	TargetNet
γ-Selinene	P05093	CYP17A1	TargetNet
γ-Selinene	Q9NR96	TLR9	TargetNet
γ-Selinene	Q96RJ0	TAAR1	TargetNet
γ-Selinene	Q04206	RELA	TargetNet
Isoborneol	P04278	SHBG	SEA
Isoborneol	P07510	CHRNG	SEA
Isoborneol	P11230	CHRNB1	SEA
Isoborneol	Q07001	CHRND	SEA
Isoborneol	P15309	ACPP	SwissTargetPrediction

Isoborneol	P29275	ADORA2B	SwissTargetPrediction
Isoborneol	P35348	ADRA1A	SwissTargetPrediction
Isoborneol	P18825	ADRA2C	SwissTargetPrediction
Isoborneol	O60218	AKR1B10	SwissTargetPrediction
Isoborneol	P09917	ALOX5	SwissTargetPrediction
Isoborneol	P10275	AR	SwissTargetPrediction
Isoborneol	P06276	BCHE	SwissTargetPrediction
Isoborneol	P25440	BRD2	SwissTargetPrediction
Isoborneol	Q15059	BRD3	SwissTargetPrediction
Isoborneol	O60885	BRD4	SwissTargetPrediction
Isoborneol	P00915	CA1	SwissTargetPrediction
Isoborneol	P00918	CA2	SwissTargetPrediction
Isoborneol	P22748	CA4	SwissTargetPrediction
Isoborneol	P30304	CDC25A	SwissTargetPrediction
Isoborneol	P30305	CDC25B	SwissTargetPrediction
Isoborneol	P11229	CHRM1	SwissTargetPrediction
Isoborneol	P20309	CHRM3	SwissTargetPrediction
Isoborneol	P08173	CHRM4	SwissTargetPrediction
Isoborneol	P15538	CYP11B1	SwissTargetPrediction
Isoborneol	P19099	CYP11B2	SwissTargetPrediction
Isoborneol	P11511	CYP19A1	SwissTargetPrediction
Isoborneol	Q16850	CYP51A1	SwissTargetPrediction
Isoborneol	Q02127	DHODH	SwissTargetPrediction
Isoborneol	P27487	DPP4	SwissTargetPrediction
Isoborneol	P14416	DRD2	SwissTargetPrediction
Isoborneol	Q9BQI3	EIF2AK1	SwissTargetPrediction
Isoborneol	P03372	ESR1	SwissTargetPrediction
Isoborneol	Q92731	ESR2	SwissTargetPrediction
Isoborneol	P12104	FABP2	SwissTargetPrediction
Isoborneol	P05413	FABP3	SwissTargetPrediction
Isoborneol	P15090	FABP4	SwissTargetPrediction
Isoborneol	Q01469	FABP5	SwissTargetPrediction
Isoborneol	O14842	FFAR1	SwissTargetPrediction
Isoborneol	P11413	G6PD	SwissTargetPrediction
Isoborneol	Q9UBS5	GABBR1	SwissTargetPrediction
Isoborneol	P47869	GABRA2	SwissTargetPrediction
Isoborneol	P47870	GABRB2	SwissTargetPrediction
Isoborneol	P18507	GABRG2	SwissTargetPrediction
Isoborneol	Q8TDU6	GPBAR1	SwissTargetPrediction
Isoborneol	P09601	HMOX1	SwissTargetPrediction
Isoborneol	P28845	HSD11B1	SwissTargetPrediction
Isoborneol	P37059	HSD17B2	SwissTargetPrediction
Isoborneol	P37058	HSD17B3	SwissTargetPrediction

Isoborneol	P28335	HTR2C	SwissTargetPrediction
Isoborneol	O60725	ICMT	SwissTargetPrediction
Isoborneol	P14902	IDO1	SwissTargetPrediction
Isoborneol	P12268	IMPDH2	SwissTargetPrediction
Isoborneol	P23458	JAK1	SwissTargetPrediction
Isoborneol	O60674	JAK2	SwissTargetPrediction
Isoborneol	P22460	KCNA5	SwissTargetPrediction
Isoborneol	P38571	LIPA	SwissTargetPrediction
Isoborneol	Q5S007	LRRK2	SwissTargetPrediction
Isoborneol	P09960	LTA4H	SwissTargetPrediction
Isoborneol	P45983	MAPK8	SwissTargetPrediction
Isoborneol	P45452	MMP13	SwissTargetPrediction
Isoborneol	P48039	MTNR1A	SwissTargetPrediction
Isoborneol	P49286	MTNR1B	SwissTargetPrediction
Isoborneol	Q9UHC9	NPC1L1	SwissTargetPrediction
Isoborneol	P16083	NQO2	SwissTargetPrediction
Isoborneol	Q13133	NR1H3	SwissTargetPrediction
Isoborneol	Q96RI1	NR1H4	SwissTargetPrediction
Isoborneol	Q14994	NR1I3	SwissTargetPrediction
Isoborneol	P04150	NR3C1	SwissTargetPrediction
Isoborneol	P08235	NR3C2	SwissTargetPrediction
Isoborneol	P22736	NR4A1	SwissTargetPrediction
Isoborneol	P41143	OPRD1	SwissTargetPrediction
Isoborneol	P41145	OPRK1	SwissTargetPrediction
Isoborneol	P35372	OPRM1	SwissTargetPrediction
Isoborneol	Q99572	P2RX7	SwissTargetPrediction
Isoborneol	P11940	PABPC1	SwissTargetPrediction
Isoborneol	P06401	PGR	SwissTargetPrediction
Isoborneol	P09884	POLA1	SwissTargetPrediction
Isoborneol	P06746	POLB	SwissTargetPrediction
Isoborneol	Q07869	PPARA	SwissTargetPrediction
Isoborneol	Q03181	PPARD	SwissTargetPrediction
Isoborneol	O60678	PRMT3	SwissTargetPrediction
Isoborneol	P25105	PTAFR	SwissTargetPrediction
Isoborneol	P43116	PTGER2	SwissTargetPrediction
Isoborneol	P43088	PTGFR	SwissTargetPrediction
Isoborneol	P35354	PTGS2	SwissTargetPrediction
Isoborneol	Q14524	SCN5A	SwissTargetPrediction
Isoborneol	Q15858	SCN9A	SwissTargetPrediction
Isoborneol	P08185	SERPINA6	SwissTargetPrediction
Isoborneol	P04278	SHBG	SwissTargetPrediction
Isoborneol	Q15465	SHH	SwissTargetPrediction
Isoborneol	Q99720	SIGMAR1	SwissTargetPrediction

Isoborneol	Q16572	SLC18A3	SwissTargetPrediction
Isoborneol	Q01959	SLC6A3	SwissTargetPrediction
Isoborneol	Q9NYA1	SPHK1	SwissTargetPrediction
Isoborneol	Q9P289	STK26	SwissTargetPrediction
Isoborneol	Q13188	STK3	SwissTargetPrediction
Isoborneol	P24557	TBXAS1	SwissTargetPrediction
Isoborneol	Q9H2K2	TNKS2	SwissTargetPrediction
Isoborneol	Q7Z2W7	TRPM8	SwissTargetPrediction
Isoborneol	Q8NER1	TRPV1	SwissTargetPrediction
Isoborneol	Q8NET8	TRPV3	SwissTargetPrediction
Isoborneol	P33981	TTK	SwissTargetPrediction
Isoborneol	P04818	TYMS	SwissTargetPrediction
Isoborneol	P16662	UGT2B7	SwissTargetPrediction
Isoborneol	Q9UKP6	UTS2R	SwissTargetPrediction
Isoborneol	P11473	VDR	SwissTargetPrediction
Isoborneol	P31213	SRD5A2	TargetNet
Isoborneol	P28566	HTR1E	TargetNet
Isoborneol	P47898	HTR5A	TargetNet
Isoborneol	P22303	ACHE	TargetNet
Isoborneol	P22303	ACHE	TargetNet
Isoborneol	P22303	ACHE	TargetNet
Isoborneol	P18089	ADRA2B	TargetNet
Isoborneol	P18825	ADRA2C	TargetNet
Isoborneol	P18825	ADRA2C	TargetNet
Isoborneol	P21397	MAOA	TargetNet
Isoborneol	P10275	AR	TargetNet
Isoborneol	P10275	AR	TargetNet
Isoborneol	P16050	ALOX15	TargetNet
Isoborneol	P11511	CYP19A1	TargetNet
Isoborneol	P35869	AHR	TargetNet
Isoborneol	Q16548	BCL2A1	TargetNet
Isoborneol	P34913	EPHX2	TargetNet
Isoborneol	P21554	CNR1	TargetNet
Isoborneol	P21554	CNR1	TargetNet
Isoborneol	P34972	CNR2	TargetNet
Isoborneol	P34972	CNR2	TargetNet
Isoborneol	P34972	CNR2	TargetNet
Isoborneol	P00915	CA1	TargetNet
Isoborneol	O43570	CA12	TargetNet
Isoborneol	Q9ULX7	CA14	TargetNet
Isoborneol	P00918	CA2	TargetNet
Isoborneol	P22748	CA4	TargetNet
Isoborneol	P35218	CA5A	TargetNet

Isoborneol	Q9Y2D0	CA5B	TargetNet
Isoborneol	P23280	CA6	TargetNet
Isoborneol	P43166	CA7	TargetNet
Isoborneol	Q16790	CA9	TargetNet
Isoborneol	Q494W8	CHRFAM7A	TargetNet
Isoborneol	O00748	CES2	TargetNet
Isoborneol	P28845	HSD11B1	TargetNet
Isoborneol	P28845	HSD11B1	TargetNet
Isoborneol	P80365	HSD11B2	TargetNet
Isoborneol	P24468	NR2F2	TargetNet
Isoborneol	P05177	CYP1A2	TargetNet
Isoborneol	P33261	CYP2C19	TargetNet
Isoborneol	P21728	DRD1	TargetNet
Isoborneol	P21918	DRD5	TargetNet
Isoborneol	Q9UHL4	DPP7	TargetNet
Isoborneol	P27487	DPP4	TargetNet
Isoborneol	Q6V1X1	DPP8	TargetNet
Isoborneol	Q86TI2	DPP9	TargetNet
Isoborneol	P26358	DNMT1	TargetNet
Isoborneol	P31941	APOBEC3A	TargetNet
Isoborneol	Q9HC16	APOBEC3G	TargetNet
Isoborneol	P51452	DUSP3	TargetNet
Isoborneol	P03372	ESR1	TargetNet
Isoborneol	Q92731	ESR2	TargetNet
Isoborneol	P14324	FDPS	TargetNet
Isoborneol	Q8TDU6	GPBAR1	TargetNet
Isoborneol	Q9HC97	GPR35	TargetNet
Isoborneol	Q05469	LIPE	TargetNet
Isoborneol	P23141	CES1	TargetNet
Isoborneol	P14174	MIF	TargetNet
Isoborneol	Q99685	MGLL	TargetNet
Isoborneol	P30305	CDC25B	TargetNet
Isoborneol	P11229	CHRM1	TargetNet
Isoborneol	P11229	CHRM1	TargetNet
Isoborneol	P08172	CHRM2	TargetNet
Isoborneol	P08172	CHRM2	TargetNet
Isoborneol	P20309	CHRM3	TargetNet
Isoborneol	P08173	CHRM4	TargetNet
Isoborneol	P43681	CHRNA4	TargetNet
Isoborneol	P43681	CHRNA4	TargetNet
Isoborneol	P36544	CHRNA7	TargetNet
Isoborneol	P36544	CHRNA7	TargetNet
Isoborneol	P29475	NOS1	TargetNet

Isoborneol	P29475	NOS1	TargetNet
Isoborneol	P29474	NOS3	TargetNet
Isoborneol	P35228	NOS2	TargetNet
Isoborneol	P35228	NOS2	TargetNet
Isoborneol	P35398	RORA	TargetNet
Isoborneol	Q00G26	PLIN5	TargetNet
Isoborneol	P11086	PNMT	TargetNet
Isoborneol	P23219	PTGS1	TargetNet
Isoborneol	P23219	PTGS1	TargetNet
Isoborneol	P35354	PTGS2	TargetNet
Isoborneol	O43353	RIPK2	TargetNet
Isoborneol	P10276	RARA	TargetNet
Isoborneol	P10826	RARB	TargetNet
Isoborneol	P13631	RARG	TargetNet
Isoborneol	Q99720	SIGMAR1	TargetNet
Isoborneol	Q99720	SIGMAR1	TargetNet
Isoborneol	Q99720	SIGMAR1	TargetNet
Isoborneol	Q01959	SLC6A3	TargetNet
Isoborneol	Q01959	SLC6A3	TargetNet
Isoborneol	P23975	SLC6A2	TargetNet
Isoborneol	O95136	S1PR2	TargetNet
Isoborneol	Q9H228	S1PR5	TargetNet
Isoborneol	P05093	CYP17A1	TargetNet
Isoborneol	P08842	STS	TargetNet
Isoborneol	Q9NR96	TLR9	TargetNet
Limonene	P22303	ACHE	SwissTargetPrediction
Limonene	P24666	ACP1	SwissTargetPrediction
Limonene	P30542	ADORA1	SwissTargetPrediction
Limonene	P29274	ADORA2A	SwissTargetPrediction
Limonene	P0DMS8	ADORA3	SwissTargetPrediction
Limonene	O60218	AKR1B10	SwissTargetPrediction
Limonene	P09917	ALOX5	SwissTargetPrediction
Limonene	P10275	AR	SwissTargetPrediction
Limonene	P06276	BCHE	SwissTargetPrediction
Limonene	P51681	CCR5	SwissTargetPrediction
Limonene	P60033	CD81	SwissTargetPrediction
Limonene	P30304	CDC25A	SwissTargetPrediction
Limonene	P30305	CDC25B	SwissTargetPrediction
Limonene	P23141	CES1	SwissTargetPrediction
Limonene	O00748	CES2	SwissTargetPrediction
Limonene	P08172	CHRM2	SwissTargetPrediction
Limonene	P21554	CNR1	SwissTargetPrediction
Limonene	P34972	CNR2	SwissTargetPrediction

Limonene	P07339	CTSD	SwissTargetPrediction
Limonene	P05093	CYP17A1	SwissTargetPrediction
Limonene	P11511	CYP19A1	SwissTargetPrediction
Limonene	P33261	CYP2C19	SwissTargetPrediction
Limonene	Q16850	CYP51A1	SwissTargetPrediction
Limonene	P03372	ESR1	SwissTargetPrediction
Limonene	Q92731	ESR2	SwissTargetPrediction
Limonene	O00519	FAAH	SwissTargetPrediction
Limonene	P07148	FABP1	SwissTargetPrediction
Limonene	P05413	FABP3	SwissTargetPrediction
Limonene	P15090	FABP4	SwissTargetPrediction
Limonene	Q01469	FABP5	SwissTargetPrediction
Limonene	P37268	FDFT1	SwissTargetPrediction
Limonene	O14842	FFAR1	SwissTargetPrediction
Limonene	P49354	FNTA	SwissTargetPrediction
Limonene	P49356	FNTB	SwissTargetPrediction
Limonene	P11413	G6PD	SwissTargetPrediction
Limonene	P08151	GLI1	SwissTargetPrediction
Limonene	P10070	GLI2	SwissTargetPrediction
Limonene	P04035	HMGCR	SwissTargetPrediction
Limonene	P28845	HSD11B1	SwissTargetPrediction
Limonene	P80365	HSD11B2	SwissTargetPrediction
Limonene	P37058	HSD17B3	SwissTargetPrediction
Limonene	P48449	LSS	SwissTargetPrediction
Limonene	P27361	MAPK3	SwissTargetPrediction
Limonene	Q9UHC9	NPC1L1	SwissTargetPrediction
Limonene	Q13133	NR1H3	SwissTargetPrediction
Limonene	O75469	NR1I2	SwissTargetPrediction
Limonene	Q14994	NR1I3	SwissTargetPrediction
Limonene	P04150	NR3C1	SwissTargetPrediction
Limonene	P08235	NR3C2	SwissTargetPrediction
Limonene	Q08499	PDE4D	SwissTargetPrediction
Limonene	P06401	PGR	SwissTargetPrediction
Limonene	P04054	PLA2G1B	SwissTargetPrediction
Limonene	P06746	POLB	SwissTargetPrediction
Limonene	Q07869	PPARA	SwissTargetPrediction
Limonene	Q03181	PPARD	SwissTargetPrediction
Limonene	P37231	PPARG	SwissTargetPrediction
Limonene	P48147	PREP	SwissTargetPrediction
Limonene	P24723	PRKCH	SwissTargetPrediction
Limonene	P34995	PTGER1	SwissTargetPrediction
Limonene	P43116	PTGER2	SwissTargetPrediction
Limonene	O14684	PTGES	SwissTargetPrediction

Limonene	P23219	PTGS1	SwissTargetPrediction
Limonene	P18031	PTPN1	SwissTargetPrediction
Limonene	Q06124	PTPN11	SwissTargetPrediction
Limonene	P17706	PTPN2	SwissTargetPrediction
Limonene	P29350	PTPN6	SwissTargetPrediction
Limonene	P10586	PTPRF	SwissTargetPrediction
Limonene	P10276	RARA	SwissTargetPrediction
Limonene	P10826	RARB	SwissTargetPrediction
Limonene	P13631	RARG	SwissTargetPrediction
Limonene	P02753	RBP4	SwissTargetPrediction
Limonene	P35398	RORA	SwissTargetPrediction
Limonene	P51449	RORC	SwissTargetPrediction
Limonene	P19793	RXRA	SwissTargetPrediction
Limonene	P48443	RXRG	SwissTargetPrediction
Limonene	O00767	SCD	SwissTargetPrediction
Limonene	P08185	SERPINA6	SwissTargetPrediction
Limonene	P04278	SHBG	SwissTargetPrediction
Limonene	Q99720	SIGMAR1	SwissTargetPrediction
Limonene	Q9GZV3	SLC5A7	SwissTargetPrediction
Limonene	P23975	SLC6A2	SwissTargetPrediction
Limonene	Q01959	SLC6A3	SwissTargetPrediction
Limonene	P31645	SLC6A4	SwissTargetPrediction
Limonene	Q14534	SQLE	SwissTargetPrediction
Limonene	P18405	SRD5A1	SwissTargetPrediction
Limonene	P31213	SRD5A2	SwissTargetPrediction
Limonene	Q12772	SREBF2	SwissTargetPrediction
Limonene	O14746	TERT	SwissTargetPrediction
Limonene	P11387	TOP1	SwissTargetPrediction
Limonene	O75762	TRPA1	SwissTargetPrediction
Limonene	Q8NER1	TRPV1	SwissTargetPrediction
Limonene	P16662	UGT2B7	SwissTargetPrediction
Limonene	P11473	VDR	SwissTargetPrediction
Limonene	P28566	HTR1E	TargetNet
Limonene	P28335	HTR2C	TargetNet
Limonene	P28335	HTR2C	TargetNet
Limonene	P47898	HTR5A	TargetNet
Limonene	P22303	ACHE	TargetNet
Limonene	P08913	ADRA2A	TargetNet
Limonene	P18089	ADRA2B	TargetNet
Limonene	P18825	ADRA2C	TargetNet
Limonene	P18825	ADRA2C	TargetNet
Limonene	P21397	MAOA	TargetNet
Limonene	P21397	MAOA	TargetNet

Limonene	P27338	MAOB	TargetNet
Limonene	P10275	AR	TargetNet
Limonene	P10275	AR	TargetNet
Limonene	P09917	ALOX5	TargetNet
Limonene	P35869	AHR	TargetNet
Limonene	Q16548	BCL2A1	TargetNet
Limonene	P21554	CNR1	TargetNet
Limonene	P34972	CNR2	TargetNet
Limonene	P34972	CNR2	TargetNet
Limonene	Q8N1Q1	CA13	TargetNet
Limonene	Q9ULX7	CA14	TargetNet
Limonene	P35218	CA5A	TargetNet
Limonene	Q9Y2D0	CA5B	TargetNet
Limonene	P23280	CA6	TargetNet
Limonene	P43166	CA7	TargetNet
Limonene	Q9GZU7	CTDSP1	TargetNet
Limonene	P55211	CASP9	TargetNet
Limonene	O00748	CES2	TargetNet
Limonene	P24468	NR2F2	TargetNet
Limonene	P05177	CYP1A2	TargetNet
Limonene	P10635	CYP2D6	TargetNet
Limonene	P21728	DRD1	TargetNet
Limonene	P21728	DRD1	TargetNet
Limonene	P21918	DRD5	TargetNet
Limonene	P26358	DNMT1	TargetNet
Limonene	P31941	APOBEC3A	TargetNet
Limonene	Q9HC16	APOBEC3G	TargetNet
Limonene	P51452	DUSP3	TargetNet
Limonene	P03372	ESR1	TargetNet
Limonene	Q92731	ESR2	TargetNet
Limonene	Q9HC97	GPR35	TargetNet
Limonene	P23141	CES1	TargetNet
Limonene	P14174	MIF	TargetNet
Limonene	P30305	CDC25B	TargetNet
Limonene	P11229	CHRM1	TargetNet
Limonene	P11229	CHRM1	TargetNet
Limonene	P08172	CHRM2	TargetNet
Limonene	P08172	CHRM2	TargetNet
Limonene	P20309	CHRM3	TargetNet
Limonene	P08173	CHRM4	TargetNet
Limonene	P43681	CHRNA4	TargetNet
Limonene	P43681	CHRNA4	TargetNet
Limonene	P29475	NOS1	TargetNet

Limonene	P29474	NOS3	TargetNet
Limonene	P35228	NOS2	TargetNet
Limonene	P35228	NOS2	TargetNet
Limonene	P35398	RORA	TargetNet
Limonene	P06401	PGR	TargetNet
Limonene	P23219	PTGS1	TargetNet
Limonene	P23219	PTGS1	TargetNet
Limonene	P35354	PTGS2	TargetNet
Limonene	O43353	RIPK2	TargetNet
Limonene	P10276	RARA	TargetNet
Limonene	P10826	RARB	TargetNet
Limonene	P13631	RARG	TargetNet
Limonene	P48443	RXRG	TargetNet
Limonene	Q99720	SIGMAR1	TargetNet
Limonene	Q99720	SIGMAR1	TargetNet
Limonene	Q99720	SIGMAR1	TargetNet
Limonene	Q01959	SLC6A3	TargetNet
Limonene	Q01959	SLC6A3	TargetNet
Limonene	P23975	SLC6A2	TargetNet
Limonene	P31645	SLC6A4	TargetNet
Limonene	O95136	S1PR2	TargetNet
Limonene	P05093	CYP17A1	TargetNet
Limonene	Q9NR96	TLR9	TargetNet
Limonene	Q96RJ0	TAAR1	TargetNet
Limonene	Q04206	RELA	TargetNet
Limonene	O95180	CACNA1H	TargetNet
Linalool	O95749	GGPS1	SEA
Linalool	Q14534	SQLE	SEA
Linalool	O60706	ABCC9	SwissTargetPrediction
Linalool	P15309	ACPP	SwissTargetPrediction
Linalool	P29274	ADORA2A	SwissTargetPrediction
Linalool	P29275	ADORA2B	SwissTargetPrediction
Linalool	P35348	ADRA1A	SwissTargetPrediction
Linalool	P18825	ADRA2C	SwissTargetPrediction
Linalool	P15121	AKR1B1	SwissTargetPrediction
Linalool	P42330	AKR1C3	SwissTargetPrediction
Linalool	P09917	ALOX5	SwissTargetPrediction
Linalool	Q96BI3	APH1A	SwissTargetPrediction
Linalool	Q8WW43	APH1B	SwissTargetPrediction
Linalool	P10275	AR	SwissTargetPrediction
Linalool	P25440	BRD2	SwissTargetPrediction
Linalool	Q15059	BRD3	SwissTargetPrediction
Linalool	O60885	BRD4	SwissTargetPrediction

Linalool	P00915	CA1	SwissTargetPrediction
Linalool	P00918	CA2	SwissTargetPrediction
Linalool	P22748	CA4	SwissTargetPrediction
Linalool	P14635	CCNB1	SwissTargetPrediction
Linalool	O95067	CCNB2	SwissTargetPrediction
Linalool	Q8WWL7	CCNB3	SwissTargetPrediction
Linalool	P24864	CCNE1	SwissTargetPrediction
Linalool	O96020	CCNE2	SwissTargetPrediction
Linalool	P06493	CDK1	SwissTargetPrediction
Linalool	P06493	CDK1	SwissTargetPrediction
Linalool	P24941	CDK2	SwissTargetPrediction
Linalool	P23141	CES1	SwissTargetPrediction
Linalool	P08172	CHRM2	SwissTargetPrediction
Linalool	P08173	CHRM4	SwissTargetPrediction
Linalool	Q9HAZ1	CLK4	SwissTargetPrediction
Linalool	P43235	CTSK	SwissTargetPrediction
Linalool	P25774	CTSS	SwissTargetPrediction
Linalool	P11511	CYP19A1	SwissTargetPrediction
Linalool	Q02127	DHODH	SwissTargetPrediction
Linalool	P14416	DRD2	SwissTargetPrediction
Linalool	P00533	EGFR	SwissTargetPrediction
Linalool	Q9BQI3	EIF2AK1	SwissTargetPrediction
Linalool	Q15056	EIF4H	SwissTargetPrediction
Linalool	P54764	EPHA4	SwissTargetPrediction
Linalool	P34913	EPHX2	SwissTargetPrediction
Linalool	P11362	FGFR1	SwissTargetPrediction
Linalool	P49841	GSK3B	SwissTargetPrediction
Linalool	O43613	HCRTR1	SwissTargetPrediction
Linalool	O43614	HCRTR2	SwissTargetPrediction
Linalool	P09601	HMOX1	SwissTargetPrediction
Linalool	Q9Y5N1	HRH3	SwissTargetPrediction
Linalool	Q9H3N8	HRH4	SwissTargetPrediction
Linalool	P37059	HSD17B2	SwissTargetPrediction
Linalool	P28335	HTR2C	SwissTargetPrediction
Linalool	O60725	ICMT	SwissTargetPrediction
Linalool	P14902	IDO1	SwissTargetPrediction
Linalool	P08069	IGF1R	SwissTargetPrediction
Linalool	P12268	IMPDH2	SwissTargetPrediction
Linalool	P06213	INSR	SwissTargetPrediction
Linalool	P23458	JAK1	SwissTargetPrediction
Linalool	O60674	JAK2	SwissTargetPrediction
Linalool	P52333	JAK3	SwissTargetPrediction
Linalool	P22460	KCNA5	SwissTargetPrediction

Linalool	P15382	KCNE1	SwissTargetPrediction
Linalool	P51787	KCNQ1	SwissTargetPrediction
Linalool	P35968	KDR	SwissTargetPrediction
Linalool	P38571	LIPA	SwissTargetPrediction
Linalool	Q5S007	LRRK2	SwissTargetPrediction
Linalool	P09960	LTA4H	SwissTargetPrediction
Linalool	P21397	MAOA	SwissTargetPrediction
Linalool	P28482	MAPK1	SwissTargetPrediction
Linalool	P53779	MAPK10	SwissTargetPrediction
Linalool	P45983	MAPK8	SwissTargetPrediction
Linalool	Q00987	MDM2	SwissTargetPrediction
Linalool	P48039	MTNR1A	SwissTargetPrediction
Linalool	P49286	MTNR1B	SwissTargetPrediction
Linalool	Q92542	NCSTN	SwissTargetPrediction
Linalool	P16083	NQO2	SwissTargetPrediction
Linalool	P04150	NR3C1	SwissTargetPrediction
Linalool	P08235	NR3C2	SwissTargetPrediction
Linalool	P04629	NTRK1	SwissTargetPrediction
Linalool	P41143	OPRD1	SwissTargetPrediction
Linalool	P41145	OPRK1	SwissTargetPrediction
Linalool	P35372	OPRM1	SwissTargetPrediction
Linalool	Q99572	P2RX7	SwissTargetPrediction
Linalool	P11940	PABPC1	SwissTargetPrediction
Linalool	P09874	PARP1	SwissTargetPrediction
Linalool	O15055	PER2	SwissTargetPrediction
Linalool	P06401	PGR	SwissTargetPrediction
Linalool	P11309	PIM1	SwissTargetPrediction
Linalool	Q86V86	PIM3	SwissTargetPrediction
Linalool	P48147	PREP	SwissTargetPrediction
Linalool	P78527	PRKDC	SwissTargetPrediction
Linalool	P49768	PSEN1	SwissTargetPrediction
Linalool	P49810	PSEN2	SwissTargetPrediction
Linalool	Q9NZ42	PSENEN	SwissTargetPrediction
Linalool	P25105	PTAFR	SwissTargetPrediction
Linalool	P35354	PTGS2	SwissTargetPrediction
Linalool	Q05397	PTK2	SwissTargetPrediction
Linalool	P06737	PYGL	SwissTargetPrediction
Linalool	Q14524	SCN5A	SwissTargetPrediction
Linalool	Q15858	SCN9A	SwissTargetPrediction
Linalool	Q99720	SIGMAR1	SwissTargetPrediction
Linalool	Q01959	SLC6A3	SwissTargetPrediction
Linalool	Q14534	SQLE	SwissTargetPrediction
Linalool	Q9P289	STK26	SwissTargetPrediction

Linalool	Q13188	STK3	SwissTargetPrediction
Linalool	Q9H2K2	TNKS2	SwissTargetPrediction
Linalool	P63316	TNNC1	SwissTargetPrediction
Linalool	P19429	TNNI3	SwissTargetPrediction
Linalool	P45379	TNNT2	SwissTargetPrediction
Linalool	Q7Z2W7	TRPM8	SwissTargetPrediction
Linalool	Q8NER1	TRPV1	SwissTargetPrediction
Linalool	Q8NET8	TRPV3	SwissTargetPrediction
Linalool	P30536	TSPO	SwissTargetPrediction
Linalool	P29597	TYK2	SwissTargetPrediction
Linalool	P04818	TYMS	SwissTargetPrediction
Linalool	Q9UKP6	UTS2R	SwissTargetPrediction
Linalool	P28566	HTR1E	TargetNet
Linalool	P22303	ACHE	TargetNet
Linalool	P05186	ALPL	TargetNet
Linalool	P08913	ADRA2A	TargetNet
Linalool	P18089	ADRA2B	TargetNet
Linalool	P18825	ADRA2C	TargetNet
Linalool	P18825	ADRA2C	TargetNet
Linalool	P10275	AR	TargetNet
Linalool	P10275	AR	TargetNet
Linalool	P16050	ALOX15	TargetNet
Linalool	P09917	ALOX5	TargetNet
Linalool	P35869	AHR	TargetNet
Linalool	Q16548	BCL2A1	TargetNet
Linalool	P00915	CA1	TargetNet
Linalool	O43570	CA12	TargetNet
Linalool	Q8N1Q1	CA13	TargetNet
Linalool	Q9ULX7	CA14	TargetNet
Linalool	P00918	CA2	TargetNet
Linalool	P22748	CA4	TargetNet
Linalool	P35218	CA5A	TargetNet
Linalool	Q9Y2D0	CA5B	TargetNet
Linalool	P23280	CA6	TargetNet
Linalool	P43166	CA7	TargetNet
Linalool	Q16790	CA9	TargetNet
Linalool	Q9GZU7	CTDSP1	TargetNet
Linalool	P68400	CSNK2A1	TargetNet
Linalool	P55211	CASP9	TargetNet
Linalool	O00748	CES2	TargetNet
Linalool	P26358	DNMT1	TargetNet
Linalool	P31941	APOBEC3A	TargetNet
Linalool	Q9HC16	APOBEC3G	TargetNet

Linalool	P51452	DUSP3	TargetNet
Linalool	Q13627	DYRK1A	TargetNet
Linalool	P14324	FDPS	TargetNet
Linalool	Q04609	FOLH1	TargetNet
Linalool	Q9HC97	GPR35	TargetNet
Linalool	P56524	HDAC4	TargetNet
Linalool	P23141	CES1	TargetNet
Linalool	P14174	MIF	TargetNet
Linalool	P50579	METAP2	TargetNet
Linalool	Q99685	MGLL	TargetNet
Linalool	P30305	CDC25B	TargetNet
Linalool	P11229	CHRM1	TargetNet
Linalool	P11229	CHRM1	TargetNet
Linalool	P08172	CHRM2	TargetNet
Linalool	P08172	CHRM2	TargetNet
Linalool	P08173	CHRM4	TargetNet
Linalool	P43681	CHRNA4	TargetNet
Linalool	P29475	NOS1	TargetNet
Linalool	P29475	NOS1	TargetNet
Linalool	P29474	NOS3	TargetNet
Linalool	P35228	NOS2	TargetNet
Linalool	O60240	PLIN1	TargetNet
Linalool	Q00G26	PLIN5	TargetNet
Linalool	P04054	PLA2G1B	TargetNet
Linalool	P23219	PTGS1	TargetNet
Linalool	P23219	PTGS1	TargetNet
Linalool	P35354	PTGS2	TargetNet
Linalool	O43353	RIPK2	TargetNet
Linalool	Q99720	SIGMAR1	TargetNet
Linalool	O95136	S1PR2	TargetNet
Linalool	O95977	S1PR4	TargetNet
Linalool	P05093	CYP17A1	TargetNet
Linalool	P37058	HSD17B3	TargetNet
Linalool	Q9NR96	TLR9	TargetNet
Linalool	Q04206	RELA	TargetNet
Myrcene	O95749	GGPS1	SEA
Myrcene	P49356	FNTB	SEA
Myrcene	Q14534	SQLE	SEA
Myrcene	P30542	ADORA1	SwissTargetPrediction
Myrcene	P29274	ADORA2A	SwissTargetPrediction
Myrcene	P0DMS8	ADORA3	SwissTargetPrediction
Myrcene	P43681	CHRNA4	SwissTargetPrediction
Myrcene	P17787	CHRNB2	SwissTargetPrediction

Myrcene	P34972	CNR2	SwissTargetPrediction
Myrcene	P49682	CXCR3	SwissTargetPrediction
Myrcene	P11511	CYP19A1	SwissTargetPrediction
Myrcene	P05177	CYP1A2	SwissTargetPrediction
Myrcene	O00519	FAAH	SwissTargetPrediction
Myrcene	P37268	FDFT1	SwissTargetPrediction
Myrcene	P08151	GLI1	SwissTargetPrediction
Myrcene	P10070	GLI2	SwissTargetPrediction
Myrcene	P28223	HTR2A	SwissTargetPrediction
Myrcene	P27338	MAOB	SwissTargetPrediction
Myrcene	Q99685	MGLL	SwissTargetPrediction
Myrcene	Q13133	NR1H3	SwissTargetPrediction
Myrcene	Q07869	PPARA	SwissTargetPrediction
Myrcene	P04278	SHBG	SwissTargetPrediction
Myrcene	Q14534	SQLE	SwissTargetPrediction
Myrcene	Q8NER1	TRPV1	SwissTargetPrediction
Myrcene	P28566	HTR1E	TargetNet
Myrcene	P47898	HTR5A	TargetNet
Myrcene	P22303	ACHE	TargetNet
Myrcene	P22303	ACHE	TargetNet
Myrcene	P05186	ALPL	TargetNet
Myrcene	P18089	ADRA2B	TargetNet
Myrcene	P18825	ADRA2C	TargetNet
Myrcene	P21397	MAOA	TargetNet
Myrcene	P27338	MAOB	TargetNet
Myrcene	P15144	ANPEP	TargetNet
Myrcene	P10275	AR	TargetNet
Myrcene	P16050	ALOX15	TargetNet
Myrcene	P09917	ALOX5	TargetNet
Myrcene	P11511	CYP19A1	TargetNet
Myrcene	Q16548	BCL2A1	TargetNet
Myrcene	P34972	CNR2	TargetNet
Myrcene	O43570	CA12	TargetNet
Myrcene	Q8N1Q1	CA13	TargetNet
Myrcene	Q9ULX7	CA14	TargetNet
Myrcene	P22748	CA4	TargetNet
Myrcene	P35218	CA5A	TargetNet
Myrcene	Q9Y2D0	CA5B	TargetNet
Myrcene	P23280	CA6	TargetNet
Myrcene	P43166	CA7	TargetNet
Myrcene	Q16790	CA9	TargetNet
Myrcene	Q9GZU7	CTDSP1	TargetNet
Myrcene	P68400	CSNK2A1	TargetNet

Myrcene	P55211	CASP9	TargetNet
Myrcene	O00748	CES2	TargetNet
Myrcene	P24468	NR2F2	TargetNet
Myrcene	P05177	CYP1A2	TargetNet
Myrcene	P21918	DRD5	TargetNet
Myrcene	P26358	DNMT1	TargetNet
Myrcene	P31941	APOBEC3A	TargetNet
Myrcene	Q9HC16	APOBEC3G	TargetNet
Myrcene	P49759	CLK1	TargetNet
Myrcene	P51452	DUSP3	TargetNet
Myrcene	Q13627	DYRK1A	TargetNet
Myrcene	P03372	ESR1	TargetNet
Myrcene	Q92731	ESR2	TargetNet
Myrcene	P14324	FDPS	TargetNet
Myrcene	O00519	FAAH	TargetNet
Myrcene	O60755	GALR3	TargetNet
Myrcene	Q04609	FOLH1	TargetNet
Myrcene	Q9HC97	GPR35	TargetNet
Myrcene	P56524	HDAC4	TargetNet
Myrcene	P23141	CES1	TargetNet
Myrcene	P14174	MIF	TargetNet
Myrcene	P50579	METAP2	TargetNet
Myrcene	Q99685	MGLL	TargetNet
Myrcene	P30305	CDC25B	TargetNet
Myrcene	P11229	CHRM1	TargetNet
Myrcene	P11229	CHRM1	TargetNet
Myrcene	P08172	CHRM2	TargetNet
Myrcene	P08172	CHRM2	TargetNet
Myrcene	P20309	CHRM3	TargetNet
Myrcene	P08173	CHRM4	TargetNet
Myrcene	P08912	CHRM5	TargetNet
Myrcene	P43681	CHRNA4	TargetNet
Myrcene	P29475	NOS1	TargetNet
Myrcene	P29475	NOS1	TargetNet
Myrcene	P29474	NOS3	TargetNet
Myrcene	P35228	NOS2	TargetNet
Myrcene	P35398	RORA	TargetNet
Myrcene	O60240	PLIN1	TargetNet
Myrcene	Q00G26	PLIN5	TargetNet
Myrcene	P11086	PNMT	TargetNet
Myrcene	P04054	PLA2G1B	TargetNet
Myrcene	P23219	PTGS1	TargetNet
Myrcene	P23219	PTGS1	TargetNet

Myrcene	P35354	PTGS2	TargetNet
Myrcene	O43353	RIPK2	TargetNet
Myrcene	P10276	RARA	TargetNet
Myrcene	Q99720	SIGMAR1	TargetNet
Myrcene	O95136	S1PR2	TargetNet
Myrcene	P05093	CYP17A1	TargetNet
Myrcene	Q9NR96	TLR9	TargetNet
Myrcene	Q04206	RELA	TargetNet
Neral	O95749	GGPS1	SEA
Neral	P48449	LSS	SEA
Neral	P49354	FNTA	SEA
Neral	P49356	FNTB	SEA
Neral	Q14534	SQLE	SEA
Neral	Q9UNQ0	ABCG2	SwissTargetPrediction
Neral	P12821	ACE	SwissTargetPrediction
Neral	P22303	ACHE	SwissTargetPrediction
Neral	P07327	ADH1A	SwissTargetPrediction
Neral	P00325	ADH1B	SwissTargetPrediction
Neral	P00326	ADH1C	SwissTargetPrediction
Neral	P08319	ADH4	SwissTargetPrediction
Neral	P40394	ADH7	SwissTargetPrediction
Neral	P35869	AHR	SwissTargetPrediction
Neral	P00352	ALDH1A1	SwissTargetPrediction
Neral	P18054	ALOX12	SwissTargetPrediction
Neral	P16050	ALOX15	SwissTargetPrediction
Neral	Q96BI3	APH1A	SwissTargetPrediction
Neral	Q8WW43	APH1B	SwissTargetPrediction
Neral	P00915	CA1	SwissTargetPrediction
Neral	O43570	CA12	SwissTargetPrediction
Neral	P00918	CA2	SwissTargetPrediction
Neral	P35218	CA5A	SwissTargetPrediction
Neral	Q00975	CACNA1B	SwissTargetPrediction
Neral	P24385	CCND1	SwissTargetPrediction
Neral	O00311	CDC7	SwissTargetPrediction
Neral	P11802	CDK4	SwissTargetPrediction
Neral	O14757	CHEK1	SwissTargetPrediction
Neral	P11229	CHRM1	SwissTargetPrediction
Neral	P08172	CHRM2	SwissTargetPrediction
Neral	P20309	CHRM3	SwissTargetPrediction
Neral	P08173	CHRM4	SwissTargetPrediction
Neral	P08912	CHRM5	SwissTargetPrediction
Neral	P43681	CHRNA4	SwissTargetPrediction
Neral	P17787	CHRNB2	SwissTargetPrediction

Neral	P07858	CTSB	SwissTargetPrediction
Neral	P43235	CTSK	SwissTargetPrediction
Neral	P07711	CTSL	SwissTargetPrediction
Neral	P15538	CYP11B1	SwissTargetPrediction
Neral	P19099	CYP11B2	SwissTargetPrediction
Neral	P11511	CYP19A1	SwissTargetPrediction
Neral	P11509	CYP2A6	SwissTargetPrediction
Neral	P33261	CYP2C19	SwissTargetPrediction
Neral	Q9UBU7	DBF4	SwissTargetPrediction
Neral	P14416	DRD2	SwissTargetPrediction
Neral	P21917	DRD4	SwissTargetPrediction
Neral	P34913	EPHX2	SwissTargetPrediction
Neral	P00734	F2	SwissTargetPrediction
Neral	P05413	FABP3	SwissTargetPrediction
Neral	P15090	FABP4	SwissTargetPrediction
Neral	P14867	GABRA1	SwissTargetPrediction
Neral	P47869	GABRA2	SwissTargetPrediction
Neral	P34903	GABRA3	SwissTargetPrediction
Neral	P31644	GABRA5	SwissTargetPrediction
Neral	Q16445	GABRA6	SwissTargetPrediction
Neral	P47870	GABRB2	SwissTargetPrediction
Neral	P28472	GABRB3	SwissTargetPrediction
Neral	P28472	GABRB3	SwissTargetPrediction
Neral	P28472	GABRB3	SwissTargetPrediction
Neral	P28472	GABRB3	SwissTargetPrediction
Neral	P18507	GABRG2	SwissTargetPrediction
Neral	P18507	GABRG2	SwissTargetPrediction
Neral	P18507	GABRG2	SwissTargetPrediction
Neral	P18507	GABRG2	SwissTargetPrediction
Neral	P18507	GABRG2	SwissTargetPrediction
Neral	P08151	GLI1	SwissTargetPrediction
Neral	P10070	GLI2	SwissTargetPrediction
Neral	Q13255	GRM1	SwissTargetPrediction
Neral	P41594	GRM5	SwissTargetPrediction
Neral	P00390	GSR	SwissTargetPrediction
Neral	Q13547	HDAC1	SwissTargetPrediction
Neral	O15379	HDAC3	SwissTargetPrediction
Neral	Q9UBN7	HDAC6	SwissTargetPrediction
Neral	Q9BY41	HDAC8	SwissTargetPrediction
Neral	P50747	HLCS	SwissTargetPrediction
Neral	P41595	HTR2B	SwissTargetPrediction
Neral	P34969	HTR7	SwissTargetPrediction
Neral	P14902	IDO1	SwissTargetPrediction

Neral	O14920	IKBKB	SwissTargetPrediction
Neral	P12268	IMPDH2	SwissTargetPrediction
Neral	P23458	JAK1	SwissTargetPrediction
Neral	O60674	JAK2	SwissTargetPrediction
Neral	Q92831	KAT2B	SwissTargetPrediction
Neral	P21397	MAOA	SwissTargetPrediction
Neral	P27338	MAOB	SwissTargetPrediction
Neral	Q16539	MAPK14	SwissTargetPrediction
Neral	Q07820	MCL1	SwissTargetPrediction
Neral	Q99685	MGLL	SwissTargetPrediction
Neral	P03956	MMP1	SwissTargetPrediction
Neral	P45452	MMP13	SwissTargetPrediction
Neral	P22894	MMP8	SwissTargetPrediction
Neral	P05164	MPO	SwissTargetPrediction
Neral	P48039	MTNR1A	SwissTargetPrediction
Neral	P49286	MTNR1B	SwissTargetPrediction
Neral	Q92542	NCSTN	SwissTargetPrediction
Neral	P15559	NQO1	SwissTargetPrediction
Neral	P16083	NQO2	SwissTargetPrediction
Neral	Q99572	P2RX7	SwissTargetPrediction
Neral	P11940	PABPC1	SwissTargetPrediction
Neral	P09874	PARP1	SwissTargetPrediction
Neral	Q9UGN5	PARP2	SwissTargetPrediction
Neral	Q13946	PDE7A	SwissTargetPrediction
Neral	Q13526	PIN1	SwissTargetPrediction
Neral	P37231	PPARG	SwissTargetPrediction
Neral	P48147	PREP	SwissTargetPrediction
Neral	P07477	PRSS1	SwissTargetPrediction
Neral	P49768	PSEN1	SwissTargetPrediction
Neral	P49810	PSEN2	SwissTargetPrediction
Neral	Q9NZ42	PSENEN	SwissTargetPrediction
Neral	P28074	PSMB5	SwissTargetPrediction
Neral	P35354	PTGS2	SwissTargetPrediction
Neral	P08575	PTPRC	SwissTargetPrediction
Neral	P49798	RGS4	SwissTargetPrediction
Neral	Q8IXJ6	SIRT2	SwissTargetPrediction
Neral	Q9GZV3	SLC5A7	SwissTargetPrediction
Neral	P23975	SLC6A2	SwissTargetPrediction
Neral	P31645	SLC6A4	SwissTargetPrediction
Neral	P35610	SOAT1	SwissTargetPrediction
Neral	P18405	SRD5A1	SwissTargetPrediction
Neral	P48775	TDO2	SwissTargetPrediction
Neral	O75762	TRPA1	SwissTargetPrediction

Neral	P02766	TTR	SwissTargetPrediction
Neral	P04818	TYMS	SwissTargetPrediction
Neral	P28566	HTR1E	TargetNet
Neral	P28335	HTR2C	TargetNet
Neral	P22303	ACHE	TargetNet
Neral	P22303	ACHE	TargetNet
Neral	P15121	AKR1B1	TargetNet
Neral	P18089	ADRA2B	TargetNet
Neral	P18825	ADRA2C	TargetNet
Neral	P18825	ADRA2C	TargetNet
Neral	P21397	MAOA	TargetNet
Neral	P27338	MAOB	TargetNet
Neral	P16050	ALOX15	TargetNet
Neral	P09917	ALOX5	TargetNet
Neral	Q16548	BCL2A1	TargetNet
Neral	P00915	CA1	TargetNet
Neral	O43570	CA12	TargetNet
Neral	Q8N1Q1	CA13	TargetNet
Neral	Q9ULX7	CA14	TargetNet
Neral	P00918	CA2	TargetNet
Neral	P22748	CA4	TargetNet
Neral	P35218	CA5A	TargetNet
Neral	Q9Y2D0	CA5B	TargetNet
Neral	P23280	CA6	TargetNet
Neral	P43166	CA7	TargetNet
Neral	Q16790	CA9	TargetNet
Neral	Q9GZU7	CTDSP1	TargetNet
Neral	P55211	CASP9	TargetNet
Neral	O00748	CES2	TargetNet
Neral	P21918	DRD5	TargetNet
Neral	P26358	DNMT1	TargetNet
Neral	P31941	APOBEC3A	TargetNet
Neral	Q9HC16	APOBEC3G	TargetNet
Neral	P51452	DUSP3	TargetNet
Neral	P14324	FDPS	TargetNet
Neral	Q04609	FOLH1	TargetNet
Neral	Q9HC97	GPR35	TargetNet
Neral	P25021	HRH2	TargetNet
Neral	P56524	HDAC4	TargetNet
Neral	P23141	CES1	TargetNet
Neral	P14174	MIF	TargetNet
Neral	Q99685	MGLL	TargetNet
Neral	P30305	CDC25B	TargetNet

Neral	P11229	CHRM1	TargetNet
Neral	P11229	CHRM1	TargetNet
Neral	P08172	CHRM2	TargetNet
Neral	P08172	CHRM2	TargetNet
Neral	P20309	CHRM3	TargetNet
Neral	P08173	CHRM4	TargetNet
Neral	P08912	CHRM5	TargetNet
Neral	P43681	CHRNA4	TargetNet
Neral	P29475	NOS1	TargetNet
Neral	P29474	NOS3	TargetNet
Neral	P35228	NOS2	TargetNet
Neral	P35228	NOS2	TargetNet
Neral	P35398	RORA	TargetNet
Neral	O60240	PLIN1	TargetNet
Neral	Q00G26	PLIN5	TargetNet
Neral	P23219	PTGS1	TargetNet
Neral	P23219	PTGS1	TargetNet
Neral	P35354	PTGS2	TargetNet
Neral	O43353	RIPK2	TargetNet
Neral	P28702	RXRB	TargetNet
Neral	Q99720	SIGMAR1	TargetNet
Neral	O95136	S1PR2	TargetNet
Neral	Q9NR96	TLR9	TargetNet
Neral	Q04206	RELA	TargetNet
Nerolidol	O95749	GGPS1	SEA
Nerolidol	P48449	LSS	SEA
Nerolidol	P49354	FNTA	SEA
Nerolidol	P49356	FNTB	SEA
Nerolidol	Q14534	SQLE	SEA
Nerolidol	O60706	ABCC9	SwissTargetPrediction
Nerolidol	Q9BV23	ABHD6	SwissTargetPrediction
Nerolidol	P30542	ADORA1	SwissTargetPrediction
Nerolidol	P29274	ADORA2A	SwissTargetPrediction
Nerolidol	P0DMS8	ADORA3	SwissTargetPrediction
Nerolidol	P15121	AKR1B1	SwissTargetPrediction
Nerolidol	P42330	AKR1C3	SwissTargetPrediction
Nerolidol	Q96BI3	APH1A	SwissTargetPrediction
Nerolidol	Q8WW43	APH1B	SwissTargetPrediction
Nerolidol	P05067	APP	SwissTargetPrediction
Nerolidol	P37288	AVPR1A	SwissTargetPrediction
Nerolidol	P30518	AVPR2	SwissTargetPrediction
Nerolidol	P56817	BACE1	SwissTargetPrediction
Nerolidol	P32247	BRS3	SwissTargetPrediction

Nerolidol	P21730	C5AR1	SwissTargetPrediction
Nerolidol	P41180	CASR	SwissTargetPrediction
Nerolidol	P32246	CCR1	SwissTargetPrediction
Nerolidol	P51686	CCR9	SwissTargetPrediction
Nerolidol	P30304	CDC25A	SwissTargetPrediction
Nerolidol	P11229	CHRM1	SwissTargetPrediction
Nerolidol	P20309	CHRM3	SwissTargetPrediction
Nerolidol	P36544	CHRNA7	SwissTargetPrediction
Nerolidol	P07333	CSF1R	SwissTargetPrediction
Nerolidol	P15538	CYP11B1	SwissTargetPrediction
Nerolidol	P19099	CYP11B2	SwissTargetPrediction
Nerolidol	P11712	CYP2C9	SwissTargetPrediction
Nerolidol	P08684	CYP3A4	SwissTargetPrediction
Nerolidol	P00533	EGFR	SwissTargetPrediction
Nerolidol	Q9BQI3	EIF2AK1	SwissTargetPrediction
Nerolidol	P34913	EPHX2	SwissTargetPrediction
Nerolidol	P25116	F2R	SwissTargetPrediction
Nerolidol	P37268	FDFT1	SwissTargetPrediction
Nerolidol	P62942	FKBP1A	SwissTargetPrediction
Nerolidol	P49354	FNTA	SwissTargetPrediction
Nerolidol	P49354	FNTA	SwissTargetPrediction
Nerolidol	P49356	FNTB	SwissTargetPrediction
Nerolidol	Q9UBS5	GABBR1	SwissTargetPrediction
Nerolidol	O75899	GABBR2	SwissTargetPrediction
Nerolidol	P47871	GCGR	SwissTargetPrediction
Nerolidol	P35557	GCK	SwissTargetPrediction
Nerolidol	P15104	GLUL	SwissTargetPrediction
Nerolidol	P42262	GRIA2	SwissTargetPrediction
Nerolidol	Q14416	GRM2	SwissTargetPrediction
Nerolidol	P41594	GRM5	SwissTargetPrediction
Nerolidol	P49841	GSK3B	SwissTargetPrediction
Nerolidol	Q16665	HIF1A	SwissTargetPrediction
Nerolidol	Q9Y5N1	HRH3	SwissTargetPrediction
Nerolidol	P37059	HSD17B2	SwissTargetPrediction
Nerolidol	P37058	HSD17B3	SwissTargetPrediction
Nerolidol	P28223	HTR2A	SwissTargetPrediction
Nerolidol	P50406	HTR6	SwissTargetPrediction
Nerolidol	O60725	ICMT	SwissTargetPrediction
Nerolidol	P14902	IDO1	SwissTargetPrediction
Nerolidol	P40189	IL6ST	SwissTargetPrediction
Nerolidol	P52333	JAK3	SwissTargetPrediction
Nerolidol	P22001	KCNA3	SwissTargetPrediction
Nerolidol	P22460	KCNA5	SwissTargetPrediction

Nerolidol	Q12809	KCNH2	SwissTargetPrediction
Nerolidol	P35968	KDR	SwissTargetPrediction
Nerolidol	P10721	KIT	SwissTargetPrediction
Nerolidol	Q05469	LIPE	SwissTargetPrediction
Nerolidol	Q5S007	LRRK2	SwissTargetPrediction
Nerolidol	O75608	LYPLA1	SwissTargetPrediction
Nerolidol	O95372	LYPLA2	SwissTargetPrediction
Nerolidol	Q16584	MAP3K11	SwissTargetPrediction
Nerolidol	Q16539	MAPK14	SwissTargetPrediction
Nerolidol	P45983	MAPK8	SwissTargetPrediction
Nerolidol	Q00987	MDM2	SwissTargetPrediction
Nerolidol	P08581	MET	SwissTargetPrediction
Nerolidol	Q92542	NCSTN	SwissTargetPrediction
Nerolidol	P49146	NPY2R	SwissTargetPrediction
Nerolidol	Q15761	NPY5R	SwissTargetPrediction
Nerolidol	P55055	NR1H2	SwissTargetPrediction
Nerolidol	Q13133	NR1H3	SwissTargetPrediction
Nerolidol	O75469	NR1I2	SwissTargetPrediction
Nerolidol	P04150	NR3C1	SwissTargetPrediction
Nerolidol	P41146	OPRL1	SwissTargetPrediction
Nerolidol	P30559	OXTR	SwissTargetPrediction
Nerolidol	Q99572	P2RX7	SwissTargetPrediction
Nerolidol	P11940	PABPC1	SwissTargetPrediction
Nerolidol	P09874	PARP1	SwissTargetPrediction
Nerolidol	Q9Y233	PDE10A	SwissTargetPrediction
Nerolidol	O00408	PDE2A	SwissTargetPrediction
Nerolidol	O15055	PER2	SwissTargetPrediction
Nerolidol	P53609	PGGT1B	SwissTargetPrediction
Nerolidol	P53609	PGGT1B	SwissTargetPrediction
Nerolidol	P06401	PGR	SwissTargetPrediction
Nerolidol	P14618	PKM	SwissTargetPrediction
Nerolidol	P42785	PRCP	SwissTargetPrediction
Nerolidol	P17252	PRKCA	SwissTargetPrediction
Nerolidol	Q05655	PRKCD	SwissTargetPrediction
Nerolidol	P49768	PSEN1	SwissTargetPrediction
Nerolidol	P49810	PSEN2	SwissTargetPrediction
Nerolidol	Q9NZ42	PSENEN	SwissTargetPrediction
Nerolidol	P35354	PTGS2	SwissTargetPrediction
Nerolidol	P18031	PTPN1	SwissTargetPrediction
Nerolidol	P06737	PYGL	SwissTargetPrediction
Nerolidol	Q15858	SCN9A	SwissTargetPrediction
Nerolidol	Q99720	SIGMAR1	SwissTargetPrediction
Nerolidol	Q12908	SLC10A2	SwissTargetPrediction

Nerolidol	Q01959	SLC6A3	SwissTargetPrediction
Nerolidol	Q14534	SQLE	SwissTargetPrediction
Nerolidol	P25103	TACR1	SwissTargetPrediction
Nerolidol	P10827	THRA	SwissTargetPrediction
Nerolidol	Q07912	TNK2	SwissTargetPrediction
Nerolidol	O75762	TRPA1	SwissTargetPrediction
Nerolidol	Q8NER1	TRPV1	SwissTargetPrediction
Nerolidol	Q8NG68	TTL	SwissTargetPrediction
Nerolidol	P22303	ACHE	TargetNet
Nerolidol	P05186	ALPL	TargetNet
Nerolidol	P18089	ADRA2B	TargetNet
Nerolidol	P18825	ADRA2C	TargetNet
Nerolidol	P16050	ALOX15	TargetNet
Nerolidol	P09917	ALOX5	TargetNet
Nerolidol	P00915	CA1	TargetNet
Nerolidol	O43570	CA12	TargetNet
Nerolidol	Q8N1Q1	CA13	TargetNet
Nerolidol	Q9ULX7	CA14	TargetNet
Nerolidol	P22748	CA4	TargetNet
Nerolidol	P35218	CA5A	TargetNet
Nerolidol	Q9Y2D0	CA5B	TargetNet
Nerolidol	P23280	CA6	TargetNet
Nerolidol	P43166	CA7	TargetNet
Nerolidol	Q16790	CA9	TargetNet
Nerolidol	P55211	CASP9	TargetNet
Nerolidol	O00748	CES2	TargetNet
Nerolidol	P26358	DNMT1	TargetNet
Nerolidol	P31941	APOBEC3A	TargetNet
Nerolidol	Q9HC16	APOBEC3G	TargetNet
Nerolidol	P14324	FDPS	TargetNet
Nerolidol	Q9HC97	GPR35	TargetNet
Nerolidol	P41235	HNF4A	TargetNet
Nerolidol	P23141	CES1	TargetNet
Nerolidol	P30305	CDC25B	TargetNet
Nerolidol	P11229	CHRM1	TargetNet
Nerolidol	P08173	CHRM4	TargetNet
Nerolidol	P29475	NOS1	TargetNet
Nerolidol	P29474	NOS3	TargetNet
Nerolidol	P35228	NOS2	TargetNet
Nerolidol	P35398	RORA	TargetNet
Nerolidol	O60240	PLIN1	TargetNet
Nerolidol	Q00G26	PLIN5	TargetNet
Nerolidol	P23219	PTGS1	TargetNet

Nerolidol	P23219	PTGS1	TargetNet
Nerolidol	O43353	RIPK2	TargetNet
Nerolidol	O95136	S1PR2	TargetNet
Nerolidol	Q9NR96	TLR9	TargetNet
Nerolidol	Q04206	RELA	TargetNet
p-Cymene	O60930	RNASEH1	SEA
p-Cymene	O75751	SLC22A3	SEA
p-Cymene	P14679	TYR	SEA
p-Cymene	P41180	CASR	SEA
p-Cymene	Q9C000	NLRP1	SEA
p-Cymene	P22303	ACHE	SwissTargetPrediction
p-Cymene	P0DMS8	ADORA3	SwissTargetPrediction
p-Cymene	P35348	ADRA1A	SwissTargetPrediction
p-Cymene	P08913	ADRA2A	SwissTargetPrediction
p-Cymene	P18089	ADRA2B	SwissTargetPrediction
p-Cymene	P18825	ADRA2C	SwissTargetPrediction
p-Cymene	P13945	ADRB3	SwissTargetPrediction
p-Cymene	P35869	AHR	SwissTargetPrediction
p-Cymene	P02768	ALB	SwissTargetPrediction
p-Cymene	P09917	ALOX5	SwissTargetPrediction
p-Cymene	P15144	ANPEP	SwissTargetPrediction
p-Cymene	Q16853	AOC3	SwissTargetPrediction
p-Cymene	P10275	AR	SwissTargetPrediction
p-Cymene	P00918	CA2	SwissTargetPrediction
p-Cymene	P22748	CA4	SwissTargetPrediction
p-Cymene	P54289	CACNA2D1	SwissTargetPrediction
p-Cymene	P23141	CES1	SwissTargetPrediction
p-Cymene	O00748	CES2	SwissTargetPrediction
p-Cymene	P11229	CHRM1	SwissTargetPrediction
p-Cymene	P08172	CHRM2	SwissTargetPrediction
p-Cymene	P20309	CHRM3	SwissTargetPrediction
p-Cymene	P21554	CNR1	SwissTargetPrediction
p-Cymene	P34972	CNR2	SwissTargetPrediction
p-Cymene	P10145	CXCL8	SwissTargetPrediction
p-Cymene	P11511	CYP19A1	SwissTargetPrediction
p-Cymene	P05177	CYP1A2	SwissTargetPrediction
p-Cymene	P11509	CYP2A6	SwissTargetPrediction
p-Cymene	P33261	CYP2C19	SwissTargetPrediction
p-Cymene	P11712	CYP2C9	SwissTargetPrediction
p-Cymene	P10635	CYP2D6	SwissTargetPrediction
p-Cymene	P08684	CYP3A4	SwissTargetPrediction
p-Cymene	P03372	ESR1	SwissTargetPrediction
p-Cymene	Q92731	ESR2	SwissTargetPrediction

p-Cymene	P62508	ESRRG	SwissTargetPrediction
p-Cymene	P00734	F2	SwissTargetPrediction
p-Cymene	O00519	FAAH	SwissTargetPrediction
p-Cymene	P05413	FABP3	SwissTargetPrediction
p-Cymene	P15090	FABP4	SwissTargetPrediction
p-Cymene	P14867	GABRA1	SwissTargetPrediction
p-Cymene	P14867	GABRA1	SwissTargetPrediction
p-Cymene	P47870	GABRB2	SwissTargetPrediction
p-Cymene	P28472	GABRB3	SwissTargetPrediction
p-Cymene	P18507	GABRG2	SwissTargetPrediction
p-Cymene	P18507	GABRG2	SwissTargetPrediction
p-Cymene	P49841	GSK3B	SwissTargetPrediction
p-Cymene	Q13547	HDAC1	SwissTargetPrediction
p-Cymene	P35367	HRH1	SwissTargetPrediction
p-Cymene	Q9Y5N1	HRH3	SwissTargetPrediction
p-Cymene	P14061	HSD17B1	SwissTargetPrediction
p-Cymene	P28222	HTR1B	SwissTargetPrediction
p-Cymene	P28221	HTR1D	SwissTargetPrediction
p-Cymene	P28223	HTR2A	SwissTargetPrediction
p-Cymene	P41595	HTR2B	SwissTargetPrediction
p-Cymene	P28335	HTR2C	SwissTargetPrediction
p-Cymene	P46098	HTR3A	SwissTargetPrediction
p-Cymene	P50406	HTR6	SwissTargetPrediction
p-Cymene	P14902	IDO1	SwissTargetPrediction
p-Cymene	P08069	IGF1R	SwissTargetPrediction
p-Cymene	Q12809	KCNH2	SwissTargetPrediction
p-Cymene	O15554	KCNN4	SwissTargetPrediction
p-Cymene	O60341	KDM1A	SwissTargetPrediction
p-Cymene	P52732	KIF11	SwissTargetPrediction
p-Cymene	P21397	MAOA	SwissTargetPrediction
p-Cymene	P27338	MAOB	SwissTargetPrediction
p-Cymene	P29475	NOS1	SwissTargetPrediction
p-Cymene	P00749	PLAU	SwissTargetPrediction
p-Cymene	P11086	PNMT	SwissTargetPrediction
p-Cymene	Q07869	PPARA	SwissTargetPrediction
p-Cymene	P23219	PTGS1	SwissTargetPrediction
p-Cymene	P35354	PTGS2	SwissTargetPrediction
p-Cymene	P21453	S1PR1	SwissTargetPrediction
p-Cymene	Q99500	S1PR3	SwissTargetPrediction
p-Cymene	O95977	S1PR4	SwissTargetPrediction
p-Cymene	Q9H228	S1PR5	SwissTargetPrediction
p-Cymene	P04278	SHBG	SwissTargetPrediction
p-Cymene	Q99720	SIGMAR1	SwissTargetPrediction

p-Cymene	Q05940	SLC18A2	SwissTargetPrediction
p-Cymene	Q4U2R8	SLC22A6	SwissTargetPrediction
p-Cymene	P23975	SLC6A2	SwissTargetPrediction
p-Cymene	Q01959	SLC6A3	SwissTargetPrediction
p-Cymene	P31645	SLC6A4	SwissTargetPrediction
p-Cymene	Q9NRA0	SPHK2	SwissTargetPrediction
p-Cymene	P31213	SRD5A2	SwissTargetPrediction
p-Cymene	Q96RJ0	TAAR1	SwissTargetPrediction
p-Cymene	P21452	TACR2	SwissTargetPrediction
p-Cymene	O75762	TRPA1	SwissTargetPrediction
p-Cymene	P14679	TYR	SwissTargetPrediction
p-Cymene	P28566	HTR1E	TargetNet
p-Cymene	P28223	HTR2A	TargetNet
p-Cymene	P28335	HTR2C	TargetNet
p-Cymene	P28335	HTR2C	TargetNet
p-Cymene	P47898	HTR5A	TargetNet
p-Cymene	P42330	AKR1C3	TargetNet
p-Cymene	P15121	AKR1B1	TargetNet
p-Cymene	P15121	AKR1B1	TargetNet
p-Cymene	P05186	ALPL	TargetNet
p-Cymene	P08913	ADRA2A	TargetNet
p-Cymene	P18089	ADRA2B	TargetNet
p-Cymene	P18825	ADRA2C	TargetNet
p-Cymene	P18825	ADRA2C	TargetNet
p-Cymene	P21397	MAOA	TargetNet
p-Cymene	P21397	MAOA	TargetNet
p-Cymene	P27338	MAOB	TargetNet
p-Cymene	P27338	MAOB	TargetNet
p-Cymene	P15144	ANPEP	TargetNet
p-Cymene	P05067	APP	TargetNet
p-Cymene	P10275	AR	TargetNet
p-Cymene	P16050	ALOX15	TargetNet
p-Cymene	P09917	ALOX5	TargetNet
p-Cymene	P09917	ALOX5	TargetNet
p-Cymene	P11511	CYP19A1	TargetNet
p-Cymene	P35869	AHR	TargetNet
p-Cymene	Q16548	BCL2A1	TargetNet
p-Cymene	P21554	CNR1	TargetNet
p-Cymene	P34972	CNR2	TargetNet
p-Cymene	P34972	CNR2	TargetNet
p-Cymene	P00915	CA1	TargetNet
p-Cymene	O43570	CA12	TargetNet
p-Cymene	Q8N1Q1	CA13	TargetNet

p-Cymene	Q9ULX7	CA14	TargetNet
p-Cymene	P00918	CA2	TargetNet
p-Cymene	P22748	CA4	TargetNet
p-Cymene	P35218	CA5A	TargetNet
p-Cymene	Q9Y2D0	CA5B	TargetNet
p-Cymene	P23280	CA6	TargetNet
p-Cymene	P43166	CA7	TargetNet
p-Cymene	Q16790	CA9	TargetNet
p-Cymene	Q9GZU7	CTDSP1	TargetNet
p-Cymene	P55211	CASP9	TargetNet
p-Cymene	O00748	CES2	TargetNet
p-Cymene	P28845	HSD11B1	TargetNet
p-Cymene	P34998	CRHR1	TargetNet
p-Cymene	P24468	NR2F2	TargetNet
p-Cymene	P15538	CYP11B1	TargetNet
p-Cymene	P19099	CYP11B2	TargetNet
p-Cymene	P05177	CYP1A2	TargetNet
p-Cymene	P10635	CYP2D6	TargetNet
p-Cymene	P21918	DRD5	TargetNet
p-Cymene	P26358	DNMT1	TargetNet
p-Cymene	P31941	APOBEC3A	TargetNet
p-Cymene	Q9HC16	APOBEC3G	TargetNet
p-Cymene	P51452	DUSP3	TargetNet
p-Cymene	P03372	ESR1	TargetNet
p-Cymene	Q92731	ESR2	TargetNet
p-Cymene	P41180	CASR	TargetNet
p-Cymene	P14324	FDPS	TargetNet
p-Cymene	Q8TDU6	GPBAR1	TargetNet
p-Cymene	Q9HC97	GPR35	TargetNet
p-Cymene	P41235	HNF4A	TargetNet
p-Cymene	Q9Y5N1	HRH3	TargetNet
p-Cymene	Q9H3N8	HRH4	TargetNet
p-Cymene	Q05469	LIPE	TargetNet
p-Cymene	Q8TDS4	HCAR2	TargetNet
p-Cymene	Q07820	MCL1	TargetNet
p-Cymene	P05362	ICAM1	TargetNet
p-Cymene	P52732	KIF11	TargetNet
p-Cymene	P23141	CES1	TargetNet
p-Cymene	P14174	MIF	TargetNet
p-Cymene	Q14416	GRM2	TargetNet
p-Cymene	Q99685	MGLL	TargetNet
p-Cymene	P30305	CDC25B	TargetNet
p-Cymene	P11229	CHRM1	TargetNet

p-Cymene	P08172	CHRM2	TargetNet
p-Cymene	P08173	CHRM4	TargetNet
p-Cymene	P43681	CHRNA4	TargetNet
p-Cymene	P43681	CHRNA4	TargetNet
p-Cymene	P48145	NPBWR1	TargetNet
p-Cymene	P29475	NOS1	TargetNet
p-Cymene	P29475	NOS1	TargetNet
p-Cymene	P29474	NOS3	TargetNet
p-Cymene	P35228	NOS2	TargetNet
p-Cymene	P35228	NOS2	TargetNet
p-Cymene	P35398	RORA	TargetNet
p-Cymene	O60240	PLIN1	TargetNet
p-Cymene	Q00G26	PLIN5	TargetNet
p-Cymene	P04054	PLA2G1B	TargetNet
p-Cymene	Q9Y5X4	NR2E3	TargetNet
p-Cymene	O14684	PTGES	TargetNet
p-Cymene	P23219	PTGS1	TargetNet
p-Cymene	P23219	PTGS1	TargetNet
p-Cymene	P35354	PTGS2	TargetNet
p-Cymene	P35354	PTGS2	TargetNet
p-Cymene	P35354	PTGS2	TargetNet
p-Cymene	P63000	RAC1	TargetNet
p-Cymene	P63000	RAC1	TargetNet
p-Cymene	O43353	RIPK2	TargetNet
p-Cymene	Q01959	SLC6A3	TargetNet
p-Cymene	Q01959	SLC6A3	TargetNet
p-Cymene	P23975	SLC6A2	TargetNet
p-Cymene	P31645	SLC6A4	TargetNet
p-Cymene	P31645	SLC6A4	TargetNet
p-Cymene	O95136	S1PR2	TargetNet
p-Cymene	P05093	CYP17A1	TargetNet
p-Cymene	Q9NR96	TLR9	TargetNet
p-Cymene	Q96RJ0	TAAR1	TargetNet
p-Cymene	Q96RJ0	TAAR1	TargetNet
p-Cymene	Q04206	RELA	TargetNet
p-Cymene	P35236	PTPN7	TargetNet
p-Cymene	Q00975	CACNA1B	TargetNet
p-Cymene	O95180	CACNA1H	TargetNet
Zerumbone	P08151	GLI1	SEA
Zerumbone	P10070	GLI2	SEA
Zerumbone	P12821	ACE	SwissTargetPrediction
Zerumbone	P07327	ADH1A	SwissTargetPrediction
Zerumbone	P00325	ADH1B	SwissTargetPrediction

Zerumbone	P00326	ADH1C	SwissTargetPrediction
Zerumbone	P08319	ADH4	SwissTargetPrediction
Zerumbone	P35348	ADRA1A	SwissTargetPrediction
Zerumbone	P25100	ADRA1D	SwissTargetPrediction
Zerumbone	P08913	ADRA2A	SwissTargetPrediction
Zerumbone	P18089	ADRA2B	SwissTargetPrediction
Zerumbone	P18825	ADRA2C	SwissTargetPrediction
Zerumbone	P35869	AHR	SwissTargetPrediction
Zerumbone	P42330	AKR1C3	SwissTargetPrediction
Zerumbone	P31749	AKT1	SwissTargetPrediction
Zerumbone	P09917	ALOX5	SwissTargetPrediction
Zerumbone	Q96BI3	APH1A	SwissTargetPrediction
Zerumbone	Q8WW43	APH1B	SwissTargetPrediction
Zerumbone	O60885	BRD4	SwissTargetPrediction
Zerumbone	P00915	CA1	SwissTargetPrediction
Zerumbone	P00918	CA2	SwissTargetPrediction
Zerumbone	Q00975	CACNA1B	SwissTargetPrediction
Zerumbone	P11229	CHRM1	SwissTargetPrediction
Zerumbone	P08172	CHRM2	SwissTargetPrediction
Zerumbone	P20309	CHRM3	SwissTargetPrediction
Zerumbone	P08173	CHRM4	SwissTargetPrediction
Zerumbone	P08912	CHRM5	SwissTargetPrediction
Zerumbone	P32297	CHRNA3	SwissTargetPrediction
Zerumbone	P43681	CHRNA4	SwissTargetPrediction
Zerumbone	P17787	CHRNB2	SwissTargetPrediction
Zerumbone	P30926	CHRNB4	SwissTargetPrediction
Zerumbone	P07858	CTSB	SwissTargetPrediction
Zerumbone	P43235	CTSK	SwissTargetPrediction
Zerumbone	P07711	CTSL	SwissTargetPrediction
Zerumbone	P25774	CTSS	SwissTargetPrediction
Zerumbone	P15538	CYP11B1	SwissTargetPrediction
Zerumbone	P19099	CYP11B2	SwissTargetPrediction
Zerumbone	P11511	CYP19A1	SwissTargetPrediction
Zerumbone	Q9Y6K1	DNMT3A	SwissTargetPrediction
Zerumbone	P21728	DRD1	SwissTargetPrediction
Zerumbone	P14416	DRD2	SwissTargetPrediction
Zerumbone	P35462	DRD3	SwissTargetPrediction
Zerumbone	P21917	DRD4	SwissTargetPrediction
Zerumbone	P07099	EPHX1	SwissTargetPrediction
Zerumbone	P34913	EPHX2	SwissTargetPrediction
Zerumbone	P07148	FABP1	SwissTargetPrediction
Zerumbone	P14867	GABRA1	SwissTargetPrediction
Zerumbone	P47869	GABRA2	SwissTargetPrediction

Zerumbone	P34903	GABRA3	SwissTargetPrediction
Zerumbone	P31644	GABRA5	SwissTargetPrediction
Zerumbone	Q16445	GABRA6	SwissTargetPrediction
Zerumbone	P47870	GABRB2	SwissTargetPrediction
Zerumbone	P28472	GABRB3	SwissTargetPrediction
Zerumbone	P28472	GABRB3	SwissTargetPrediction
Zerumbone	P28472	GABRB3	SwissTargetPrediction
Zerumbone	P28472	GABRB3	SwissTargetPrediction
Zerumbone	P18507	GABRG2	SwissTargetPrediction
Zerumbone	P18507	GABRG2	SwissTargetPrediction
Zerumbone	P18507	GABRG2	SwissTargetPrediction
Zerumbone	P18507	GABRG2	SwissTargetPrediction
Zerumbone	P18507	GABRG2	SwissTargetPrediction
Zerumbone	P08151	GLI1	SwissTargetPrediction
Zerumbone	P10070	GLI2	SwissTargetPrediction
Zerumbone	Q13255	GRM1	SwissTargetPrediction
Zerumbone	P41594	GRM5	SwissTargetPrediction
Zerumbone	O43613	HCRTR1	SwissTargetPrediction
Zerumbone	O43614	HCRTR2	SwissTargetPrediction
Zerumbone	P35367	HRH1	SwissTargetPrediction
Zerumbone	P25021	HRH2	SwissTargetPrediction
Zerumbone	P37059	HSD17B2	SwissTargetPrediction
Zerumbone	P08908	HTR1A	SwissTargetPrediction
Zerumbone	P28222	HTR1B	SwissTargetPrediction
Zerumbone	P28221	HTR1D	SwissTargetPrediction
Zerumbone	P28223	HTR2A	SwissTargetPrediction
Zerumbone	P41595	HTR2B	SwissTargetPrediction
Zerumbone	P28335	HTR2C	SwissTargetPrediction
Zerumbone	P50406	HTR6	SwissTargetPrediction
Zerumbone	P34969	HTR7	SwissTargetPrediction
Zerumbone	P14902	IDO1	SwissTargetPrediction
Zerumbone	P12268	IMPDH2	SwissTargetPrediction
Zerumbone	P21397	MAOA	SwissTargetPrediction
Zerumbone	P27338	MAOB	SwissTargetPrediction
Zerumbone	Q16539	MAPK14	SwissTargetPrediction
Zerumbone	P32245	MC4R	SwissTargetPrediction
Zerumbone	P03956	MMP1	SwissTargetPrediction
Zerumbone	P45452	MMP13	SwissTargetPrediction
Zerumbone	P05164	MPO	SwissTargetPrediction
Zerumbone	P48039	MTNR1A	SwissTargetPrediction
Zerumbone	P49286	MTNR1B	SwissTargetPrediction
Zerumbone	Q92542	NCSTN	SwissTargetPrediction
Zerumbone	P16083	NQO2	SwissTargetPrediction

Zerumbone	O75469	NR1I2	SwissTargetPrediction
Zerumbone	P08235	NR3C2	SwissTargetPrediction
Zerumbone	Q99572	P2RX7	SwissTargetPrediction
Zerumbone	P11940	PABPC1	SwissTargetPrediction
Zerumbone	P09874	PARP1	SwissTargetPrediction
Zerumbone	P53350	PLK1	SwissTargetPrediction
Zerumbone	Q07869	PPARA	SwissTargetPrediction
Zerumbone	Q03181	PPARD	SwissTargetPrediction
Zerumbone	P48147	PREP	SwissTargetPrediction
Zerumbone	P49768	PSEN1	SwissTargetPrediction
Zerumbone	P49810	PSEN2	SwissTargetPrediction
Zerumbone	Q9NZ42	PSENEN	SwissTargetPrediction
Zerumbone	P35354	PTGS2	SwissTargetPrediction
Zerumbone	P17706	PTPN2	SwissTargetPrediction
Zerumbone	P08185	SERPINA6	SwissTargetPrediction
Zerumbone	P04278	SHBG	SwissTargetPrediction
Zerumbone	Q8IXJ6	SIRT2	SwissTargetPrediction
Zerumbone	P23975	SLC6A2	SwissTargetPrediction
Zerumbone	P31645	SLC6A4	SwissTargetPrediction
Zerumbone	P18405	SRD5A1	SwissTargetPrediction
Zerumbone	P31213	SRD5A2	SwissTargetPrediction
Zerumbone	P48775	TDO2	SwissTargetPrediction
Zerumbone	Q9H2K2	TNKS2	SwissTargetPrediction
Zerumbone	O75762	TRPA1	SwissTargetPrediction
Zerumbone	Q8NER1	TRPV1	SwissTargetPrediction
Zerumbone	P30536	TSPO	SwissTargetPrediction
Zerumbone	P04818	TYMS	SwissTargetPrediction
Zerumbone	Q9UKP6	UTS2R	SwissTargetPrediction
Zerumbone	P28566	HTR1E	TargetNet
Zerumbone	P28335	HTR2C	TargetNet
Zerumbone	P22303	ACHE	TargetNet
Zerumbone	P22303	ACHE	TargetNet
Zerumbone	P18089	ADRA2B	TargetNet
Zerumbone	P18825	ADRA2C	TargetNet
Zerumbone	P10275	AR	TargetNet
Zerumbone	P10275	AR	TargetNet
Zerumbone	P11511	CYP19A1	TargetNet
Zerumbone	P35869	AHR	TargetNet
Zerumbone	Q16548	BCL2A1	TargetNet
Zerumbone	Q8N1Q1	CA13	TargetNet
Zerumbone	Q9ULX7	CA14	TargetNet
Zerumbone	Q9Y2D0	CA5B	TargetNet
Zerumbone	P23280	CA6	TargetNet

Zerumbone	P43166	CA7	TargetNet
Zerumbone	Q9GZU7	CTDSP1	TargetNet
Zerumbone	P55211	CASP9	TargetNet
Zerumbone	O00748	CES2	TargetNet
Zerumbone	P26358	DNMT1	TargetNet
Zerumbone	P31941	APOBEC3A	TargetNet
Zerumbone	Q9HC16	APOBEC3G	TargetNet
Zerumbone	P51452	DUSP3	TargetNet
Zerumbone	P23141	CES1	TargetNet
Zerumbone	P14174	MIF	TargetNet
Zerumbone	Q99685	MGLL	TargetNet
Zerumbone	P30305	CDC25B	TargetNet
Zerumbone	P11229	CHRM1	TargetNet
Zerumbone	P08173	CHRM4	TargetNet
Zerumbone	P29474	NOS3	TargetNet
Zerumbone	P35228	NOS2	TargetNet
Zerumbone	P35398	RORA	TargetNet
Zerumbone	O60240	PLIN1	TargetNet
Zerumbone	Q00G26	PLIN5	TargetNet
Zerumbone	P06401	PGR	TargetNet
Zerumbone	P23219	PTGS1	TargetNet
Zerumbone	O43353	RIPK2	TargetNet
Zerumbone	P10276	RARA	TargetNet
Zerumbone	P10826	RARB	TargetNet
Zerumbone	P13631	RARG	TargetNet
Zerumbone	P48443	RXRG	TargetNet
Zerumbone	P37058	HSD17B3	TargetNet
Zerumbone	Q9NR96	TLR9	TargetNet
Zerumbone	Q04206	RELA	TargetNet
Zingiberenol	P02511	CRYAB	SEA
Zingiberenol	P21757	MSR1	SEA
Zingiberenol	Q9BV23	ABHD6	SwissTargetPrediction
Zingiberenol	P22303	ACHE	SwissTargetPrediction
Zingiberenol	P00813	ADA	SwissTargetPrediction
Zingiberenol	P42330	AKR1C3	SwissTargetPrediction
Zingiberenol	Q96BI3	APH1A	SwissTargetPrediction
Zingiberenol	Q8WW43	APH1B	SwissTargetPrediction
Zingiberenol	P05067	APP	SwissTargetPrediction
Zingiberenol	P10275	AR	SwissTargetPrediction
Zingiberenol	P37288	AVPR1A	SwissTargetPrediction
Zingiberenol	P06276	BCHE	SwissTargetPrediction
Zingiberenol	P32247	BRS3	SwissTargetPrediction
Zingiberenol	P21730	C5AR1	SwissTargetPrediction

Zingiberenol	P32246	CCR1	SwissTargetPrediction
Zingiberenol	P30304	CDC25A	SwissTargetPrediction
Zingiberenol	P30305	CDC25B	SwissTargetPrediction
Zingiberenol	P11229	CHRM1	SwissTargetPrediction
Zingiberenol	P08172	CHRM2	SwissTargetPrediction
Zingiberenol	P20309	CHRM3	SwissTargetPrediction
Zingiberenol	P08173	CHRM4	SwissTargetPrediction
Zingiberenol	P08912	CHRM5	SwissTargetPrediction
Zingiberenol	P21554	CNR1	SwissTargetPrediction
Zingiberenol	P07333	CSF1R	SwissTargetPrediction
Zingiberenol	P15538	CYP11B1	SwissTargetPrediction
Zingiberenol	P19099	CYP11B2	SwissTargetPrediction
Zingiberenol	P05093	CYP17A1	SwissTargetPrediction
Zingiberenol	P11511	CYP19A1	SwissTargetPrediction
Zingiberenol	P33261	CYP2C19	SwissTargetPrediction
Zingiberenol	Q16850	CYP51A1	SwissTargetPrediction
Zingiberenol	P34913	EPHX2	SwissTargetPrediction
Zingiberenol	P03372	ESR1	SwissTargetPrediction
Zingiberenol	Q92731	ESR2	SwissTargetPrediction
Zingiberenol	P25116	F2R	SwissTargetPrediction
Zingiberenol	P07148	FABP1	SwissTargetPrediction
Zingiberenol	P05413	FABP3	SwissTargetPrediction
Zingiberenol	P15090	FABP4	SwissTargetPrediction
Zingiberenol	Q01469	FABP5	SwissTargetPrediction
Zingiberenol	P49354	FNTA	SwissTargetPrediction
Zingiberenol	P49356	FNTB	SwissTargetPrediction
Zingiberenol	P47871	GCGR	SwissTargetPrediction
Zingiberenol	Q14416	GRM2	SwissTargetPrediction
Zingiberenol	P41594	GRM5	SwissTargetPrediction
Zingiberenol	Q16665	HIF1A	SwissTargetPrediction
Zingiberenol	P04035	HMGCR	SwissTargetPrediction
Zingiberenol	P28845	HSD11B1	SwissTargetPrediction
Zingiberenol	P37058	HSD17B3	SwissTargetPrediction
Zingiberenol	P40189	IL6ST	SwissTargetPrediction
Zingiberenol	P23458	JAK1	SwissTargetPrediction
Zingiberenol	O60674	JAK2	SwissTargetPrediction
Zingiberenol	P52333	JAK3	SwissTargetPrediction
Zingiberenol	P22001	KCNA3	SwissTargetPrediction
Zingiberenol	P22460	KCNA5	SwissTargetPrediction
Zingiberenol	Q12809	KCNH2	SwissTargetPrediction
Zingiberenol	P35968	KDR	SwissTargetPrediction
Zingiberenol	P10721	KIT	SwissTargetPrediction
Zingiberenol	Q15722	LTB4R	SwissTargetPrediction

Zingiberenol	Q16584	MAP3K11	SwissTargetPrediction
Zingiberenol	P45983	MAPK8	SwissTargetPrediction
Zingiberenol	Q92542	NCSTN	SwissTargetPrediction
Zingiberenol	Q9UHC9	NPC1L1	SwissTargetPrediction
Zingiberenol	Q15761	NPY5R	SwissTargetPrediction
Zingiberenol	Q13133	NR1H3	SwissTargetPrediction
Zingiberenol	Q14994	NR1I3	SwissTargetPrediction
Zingiberenol	P04150	NR3C1	SwissTargetPrediction
Zingiberenol	Q99572	P2RX7	SwissTargetPrediction
Zingiberenol	P11940	PABPC1	SwissTargetPrediction
Zingiberenol	O00408	PDE2A	SwissTargetPrediction
Zingiberenol	O15055	PER2	SwissTargetPrediction
Zingiberenol	P11309	PIM1	SwissTargetPrediction
Zingiberenol	Q86V86	PIM3	SwissTargetPrediction
Zingiberenol	P06746	POLB	SwissTargetPrediction
Zingiberenol	Q07869	PPARA	SwissTargetPrediction
Zingiberenol	Q03181	PPARD	SwissTargetPrediction
Zingiberenol	P37231	PPARG	SwissTargetPrediction
Zingiberenol	P05771	PRKCB	SwissTargetPrediction
Zingiberenol	Q05655	PRKCD	SwissTargetPrediction
Zingiberenol	Q02156	PRKCE	SwissTargetPrediction
Zingiberenol	P05129	PRKCG	SwissTargetPrediction
Zingiberenol	Q04759	PRKCQ	SwissTargetPrediction
Zingiberenol	P49768	PSEN1	SwissTargetPrediction
Zingiberenol	P49810	PSEN2	SwissTargetPrediction
Zingiberenol	Q9NZ42	PSENEN	SwissTargetPrediction
Zingiberenol	Q13258	PTGDR	SwissTargetPrediction
Zingiberenol	P43088	PTGFR	SwissTargetPrediction
Zingiberenol	P18031	PTPN1	SwissTargetPrediction
Zingiberenol	P17706	PTPN2	SwissTargetPrediction
Zingiberenol	P29350	PTPN6	SwissTargetPrediction
Zingiberenol	P06737	PYGL	SwissTargetPrediction
Zingiberenol	O95267	RASGRP1	SwissTargetPrediction
Zingiberenol	P35398	RORA	SwissTargetPrediction
Zingiberenol	P51449	RORC	SwissTargetPrediction
Zingiberenol	Q15858	SCN9A	SwissTargetPrediction
Zingiberenol	P08185	SERPINA6	SwissTargetPrediction
Zingiberenol	P04278	SHBG	SwissTargetPrediction
Zingiberenol	Q16572	SLC18A3	SwissTargetPrediction
Zingiberenol	P23975	SLC6A2	SwissTargetPrediction
Zingiberenol	P31645	SLC6A4	SwissTargetPrediction
Zingiberenol	Q9NYA1	SPHK1	SwissTargetPrediction
Zingiberenol	Q9NRA0	SPHK2	SwissTargetPrediction

Zingiberenol	Q14534	SQLE	SwissTargetPrediction
Zingiberenol	P31213	SRD5A2	SwissTargetPrediction
Zingiberenol	Q12772	SREBF2	SwissTargetPrediction
Zingiberenol	O14746	TERT	SwissTargetPrediction
Zingiberenol	P10827	THRA	SwissTargetPrediction
Zingiberenol	O75762	TRPA1	SwissTargetPrediction
Zingiberenol	P29597	TYK2	SwissTargetPrediction
Zingiberenol	P16662	UGT2B7	SwissTargetPrediction
Zingiberenol	P22303	ACHE	TargetNet
Zingiberenol	P08913	ADRA2A	TargetNet
Zingiberenol	P18089	ADRA2B	TargetNet
Zingiberenol	P18825	ADRA2C	TargetNet
Zingiberenol	P18825	ADRA2C	TargetNet
Zingiberenol	P31941	APOBEC3A	TargetNet
Zingiberenol	Q99685	MGLL	TargetNet
Zingiberenol	P30305	CDC25B	TargetNet
Zingiberenol	P11229	CHRM1	TargetNet
Zingiberenol	P08172	CHRM2	TargetNet
Zingiberenol	P20309	CHRM3	TargetNet
Zingiberenol	P08173	CHRM4	TargetNet
Zingiberenol	P43681	CHRNA4	TargetNet
Zingiberenol	P35228	NOS2	TargetNet
Zingiberenol	Q99720	SIGMAR1	TargetNet
Zingiberenol	Q01959	SLC6A3	TargetNet
Zingiberenol	Q9NR96	TLR9	TargetNet

Table S3: Human protein-protein interactions of 696 predicted targets from STRING database							
node1	node2	node1_string_id	node2_string_id	coexpression	entally_determined_int	database_annotated	combined_score
ABCB1	EP300	9606.ENSP00000478255	9606.ENSP00000263253	0	0.076	0.9	0.903
ABCB1	MAPK8	9606.ENSP00000478255	9606.ENSP00000378974	0	0	0.9	0.9
ABCC9	ATP12A	9606.ENSP00000261200	9606.ENSP00000218548	0	0.063	0.54	0.55
ABL1	CCND1	9606.ENSP00000361423	9606.ENSP00000227507	0.07	0	0.9	0.903
ABL1	MAPK14	9606.ENSP00000361423	9606.ENSP00000229795	0.062	0.148	0.6	0.652
ABL1	CDK4	9606.ENSP00000361423	9606.ENSP00000257904	0.062	0.072	0.9	0.905
ABL1	MDM2	9606.ENSP00000361423	9606.ENSP00000258149	0	0.68	0.9	0.966
ABL1	PDGFRB	9606.ENSP00000361423	9606.ENSP00000261799	0.089	0.331	0.9	0.933
ABL1	CCNE1	9606.ENSP00000361423	9606.ENSP00000262643	0.058	0.139	0.9	0.911
ABL1	CDK2	9606.ENSP00000361423	9606.ENSP00000266970	0.085	0.102	0.9	0.91
ABL1	ERBB2	9606.ENSP00000361423	9606.ENSP00000269571	0	0.667	0	0.667
ABL1	CCNA2	9606.ENSP00000361423	9606.ENSP00000274026	0	0.103	0.9	0.906
ABL1	EGFR	9606.ENSP00000361423	9606.ENSP00000275493	0.069	0.838	0	0.843
ABL1	EPHA4	9606.ENSP00000361423	9606.ENSP00000281821	0.062	0.113	0.8	0.819
ABL1	PPM1B	9606.ENSP00000361423	9606.ENSP00000282412	0.065	0.723	0	0.73
ABL1	YWHAG	9606.ENSP00000361423	9606.ENSP00000306330	0.065	0.717	0	0.724
ABL1	TERT	9606.ENSP00000361423	9606.ENSP00000309572	0.049	0.213	0.9	0.918
ABL1	EPHB3	9606.ENSP00000361423	9606.ENSP00000332118	0.062	0.057	0.8	0.807
ABL1	HSP90AA1	9606.ENSP00000361423	9606.ENSP00000335153	0	0.829	0	0.829
ABL1	EPHA3	9606.ENSP00000361423	9606.ENSP00000337451	0	0.282	0.8	0.85
ABL1	LCK	9606.ENSP00000361423	9606.ENSP00000337825	0	0	0.9	0.9
ABL1	MAPT	9606.ENSP00000361423	9606.ENSP00000340820	0.053	0.541	0	0.547
ABL1	JAK1	9606.ENSP00000361423	9606.ENSP00000343204	0	0.462	0	0.461
ABL1	ATR	9606.ENSP00000361423	9606.ENSP00000343741	0.064	0.496	0.9	0.948
ABL1	RAC1	9606.ENSP00000361423	9606.ENSP00000348461	0	0.14	0.9	0.91
ABL1	EPHB4	9606.ENSP00000361423	9606.ENSP00000350896	0.069	0.057	0.8	0.809
ABL1	RAD52	9606.ENSP00000361423	9606.ENSP00000351284	0	0.234	0.9	0.92
ABL1	JUN	9606.ENSP00000361423	9606.ENSP00000360266	0.062	0.683	0	0.69
ABL1	PTPN1	9606.ENSP00000361423	9606.ENSP00000360683	0.062	0.426	0.9	0.941
ABL1	PTPN6	9606.ENSP00000361423	9606.ENSP00000391592	0.063	0.482	0	0.494
ABL1	YES1	9606.ENSP00000361423	9606.ENSP00000462468	0	0	0.9	0.9
ABL1	FGR	9606.ENSP00000361423	9606.ENSP00000363117	0	0	0.9	0.9
ABL1	LYN	9606.ENSP00000361423	9606.ENSP00000428924	0	0	0.9	0.9
ABL1	MAPK8	9606.ENSP00000361423	9606.ENSP00000378974	0	0.101	0.9	0.906
ABL1	PIK3R1	9606.ENSP00000361423	9606.ENSP00000428056	0	0.883	0	0.883
ABL1	HDAC1	9606.ENSP00000361423	9606.ENSP00000362649	0	0.078	0.9	0.903
ABL1	CDK5	9606.ENSP00000361423	9606.ENSP00000419782	0	0.225	0.9	0.919
ABL1	HCK	9606.ENSP00000361423	9606.ENSP00000444986	0	0.486	0.9	0.946
ABL1	NTRK1	9606.ENSP00000361423	9606.ENSP00000431418	0	0.486	0.9	0.946
ABL1	SRC	9606.ENSP00000361423	9606.ENSP00000362680	0.067	0.675	0.9	0.967
ACHE	APP	9606.ENSP00000303211	9606.ENSP00000284981	0.092	0.801	0	0.811
ACP1	INSR	9606.ENSP00000272067	9606.ENSP00000303830	0	0.47	0	0.47

ACPP	ERBB2	9606.ENSP00000323036	9606.ENSP00000269571	0	0.463	0	0.463
ACVR1	BMP4	9606.ENSP00000263640	9606.ENSP00000245451	0	0.662	0.8	0.929
ACVR1	TGFBR1	9606.ENSP00000263640	9606.ENSP00000364133	0	0.569	0.54	0.793
ACVR1	FKBP1A	9606.ENSP00000263640	9606.ENSP00000383003	0	0.849	0.9	0.984
ADA	PRMT3	9606.ENSP00000361965	9606.ENSP00000331879	0.5	0	0	0.499
ADA	DPP4	9606.ENSP00000361965	9606.ENSP00000353731	0.064	0.749	0	0.755
ADA	PNP	9606.ENSP00000361965	9606.ENSP00000354532	0.102	0	0.9	0.906
ADA	ADORA1	9606.ENSP00000361965	9606.ENSP00000356205	0	0.478	0	0.478
ADAM10	LCK	9606.ENSP00000260408	9606.ENSP00000337825	0	0.486	0	0.485
ADAM10	EPHA4	9606.ENSP00000260408	9606.ENSP00000281821	0.061	0	0.6	0.608
ADAM10	CD81	9606.ENSP00000260408	9606.ENSP00000263645	0	0.696	0	0.696
ADAM10	ADAM17	9606.ENSP00000260408	9606.ENSP00000309968	0.062	0	0.8	0.804
ADAM10	EPHA3	9606.ENSP00000260408	9606.ENSP00000337451	0.061	0.462	0.6	0.78
ADAM10	APP	9606.ENSP00000260408	9606.ENSP00000284981	0.062	0.395	0.9	0.938
ADAM17	MAPK14	9606.ENSP00000309968	9606.ENSP00000229795	0	0	0.9	0.9
ADAM17	ERBB2	9606.ENSP00000309968	9606.ENSP00000269571	0.049	0	0.9	0.9
ADAM17	SHH	9606.ENSP00000309968	9606.ENSP00000297261	0	0	0.9	0.9
ADAM17	PRKCB	9606.ENSP00000309968	9606.ENSP00000305355	0	0	0.9	0.9
ADAM17	PRKCA	9606.ENSP00000309968	9606.ENSP00000408695	0	0	0.9	0.9
ADAM17	TNF	9606.ENSP00000309968	9606.ENSP00000398698	0.062	0.213	0.9	0.919
ADH1A	ADH7	9606.ENSP00000209668	9606.ENSP00000420269	0.064	0	0.6	0.609
ADH1A	ADH4	9606.ENSP00000209668	9606.ENSP00000265512	0.065	0	0.6	0.61
ADH1A	UGT2B7	9606.ENSP00000209668	9606.ENSP00000304811	0.098	0	0.65	0.67
ADH1A	MAOB	9606.ENSP00000209668	9606.ENSP00000367309	0.065	0	0.65	0.658
ADH1A	MAOA	9606.ENSP00000209668	9606.ENSP00000340684	0.097	0	0.65	0.67
ADH1A	COMT	9606.ENSP00000209668	9606.ENSP00000354511	0.062	0	0.65	0.657
ADH1A	HPGDS	9606.ENSP00000209668	9606.ENSP00000295256	0.101	0.166	0.65	0.714
ADH1A	ADH1C	9606.ENSP00000209668	9606.ENSP00000426083	0.182	0	0.8	0.829
ADH1A	ALDH3A1	9606.ENSP00000209668	9606.ENSP00000411821	0.062	0	0.65	0.657
ADH1A	ALDH1A1	9606.ENSP00000209668	9606.ENSP00000297785	0.064	0	0.65	0.658
ADH1A	ALDH2	9606.ENSP00000209668	9606.ENSP00000261733	0.135	0	0.65	0.684
ADH1A	ADH1B	9606.ENSP00000209668	9606.ENSP00000306606	0.828	0	0.8	0.964
ADH1B	ALDH2	9606.ENSP00000306606	9606.ENSP00000261733	0.14	0	0.9	0.91
ADH1B	ADH4	9606.ENSP00000306606	9606.ENSP00000265512	0.065	0	0.6	0.61
ADH1B	HPGDS	9606.ENSP00000306606	9606.ENSP00000295256	0.1	0.166	0.65	0.714
ADH1B	ALDH1A1	9606.ENSP00000306606	9606.ENSP00000297785	0.087	0	0.65	0.666
ADH1B	CYP2A6	9606.ENSP00000306606	9606.ENSP00000301141	0.085	0	0.9	0.904
ADH1B	UGT2B7	9606.ENSP00000306606	9606.ENSP00000304811	0.064	0	0.65	0.658
ADH1B	ADH7	9606.ENSP00000306606	9606.ENSP00000420269	0.064	0	0.6	0.609
ADH1B	MAOB	9606.ENSP00000306606	9606.ENSP00000367309	0.104	0	0.65	0.673
ADH1B	ALDH3A1	9606.ENSP00000306606	9606.ENSP00000411821	0.062	0	0.65	0.657
ADH1B	ADH1C	9606.ENSP00000306606	9606.ENSP00000426083	0.179	0.66	0.8	0.939
ADH1B	MAOA	9606.ENSP00000306606	9606.ENSP00000340684	0.104	0	0.9	0.906
ADH1B	COMT	9606.ENSP00000306606	9606.ENSP00000354511	0.062	0	0.9	0.902

ADH1C	ALDH2	9606.ENSP00000426083	9606.ENSP00000261733	0.087	0	0.65	0.666
ADH1C	ADH4	9606.ENSP00000426083	9606.ENSP00000265512	0.065	0	0.6	0.61
ADH1C	HPGDS	9606.ENSP00000426083	9606.ENSP00000295256	0	0.166	0.65	0.695
ADH1C	ALDH1A1	9606.ENSP00000426083	9606.ENSP00000297785	0.098	0	0.65	0.67
ADH1C	UGT2B7	9606.ENSP00000426083	9606.ENSP00000304811	0.119	0	0.65	0.678
ADH1C	MAOA	9606.ENSP00000426083	9606.ENSP00000340684	0.103	0	0.65	0.672
ADH1C	COMT	9606.ENSP00000426083	9606.ENSP00000354511	0.062	0	0.65	0.657
ADH1C	MAOB	9606.ENSP00000426083	9606.ENSP00000367309	0.093	0	0.65	0.669
ADH1C	ALDH3A1	9606.ENSP00000426083	9606.ENSP00000411821	0.062	0	0.65	0.657
ADH1C	ADH7	9606.ENSP00000426083	9606.ENSP00000420269	0.064	0	0.6	0.609
ADH4	ALDH2	9606.ENSP00000265512	9606.ENSP00000261733	0.063	0	0.65	0.658
ADH4	ADH7	9606.ENSP00000265512	9606.ENSP00000420269	0.064	0	0.6	0.609
ADH4	MAOB	9606.ENSP00000265512	9606.ENSP00000367309	0.063	0	0.65	0.658
ADH4	UGT2B7	9606.ENSP00000265512	9606.ENSP00000304811	0.186	0	0.65	0.702
ADH4	HPGDS	9606.ENSP00000265512	9606.ENSP00000295256	0	0.166	0.65	0.695
ADH4	ALDH3A1	9606.ENSP00000265512	9606.ENSP00000411821	0.062	0	0.65	0.657
ADH4	ALDH1A1	9606.ENSP00000265512	9606.ENSP00000297785	0.063	0	0.65	0.658
ADH4	MAOA	9606.ENSP00000265512	9606.ENSP00000340684	0.062	0	0.9	0.902
ADH4	COMT	9606.ENSP00000265512	9606.ENSP00000354511	0.062	0	0.9	0.902
ADH7	ALDH2	9606.ENSP00000420269	9606.ENSP00000261733	0.063	0	0.65	0.658
ADH7	HPGDS	9606.ENSP00000420269	9606.ENSP00000295256	0	0.166	0.65	0.695
ADH7	ALDH1A1	9606.ENSP00000420269	9606.ENSP00000297785	0.064	0	0.65	0.658
ADH7	UGT2B7	9606.ENSP00000420269	9606.ENSP00000304811	0.064	0	0.65	0.658
ADH7	MAOA	9606.ENSP00000420269	9606.ENSP00000340684	0.062	0	0.65	0.657
ADH7	COMT	9606.ENSP00000420269	9606.ENSP00000354511	0.062	0	0.65	0.657
ADH7	MAOB	9606.ENSP00000420269	9606.ENSP00000367309	0.063	0	0.65	0.658
ADH7	ALDH3A1	9606.ENSP00000420269	9606.ENSP00000411821	0.063	0	0.65	0.658
ADORA1	OPRD1	9606.ENSP00000356205	9606.ENSP00000234961	0.062	0	0.8	0.804
ADORA1	ADORA2B	9606.ENSP00000356205	9606.ENSP00000304501	0.065	0.219	0.8	0.841
ADORA1	ADORA2A	9606.ENSP00000356205	9606.ENSP00000336630	0.062	0.474	0.8	0.892
ADORA1	GNAI1	9606.ENSP00000356205	9606.ENSP00000343027	0.089	0.151	0.8	0.831
ADORA1	GNAI3	9606.ENSP00000356205	9606.ENSP00000358867	0.062	0.151	0.8	0.826
ADORA2A	ADORA2B	9606.ENSP00000336630	9606.ENSP00000304501	0	0.324	0.8	0.859
ADORA2A	CNR1	9606.ENSP00000336630	9606.ENSP00000358513	0	0.754	0	0.754
ADORA2A	DRD2	9606.ENSP00000336630	9606.ENSP00000354859	0.063	0.787	0	0.792
ADRA1A	ADRA1B	9606.ENSP00000369960	9606.ENSP00000306662	0.062	0.676	0.8	0.933
ADRA1A	ADRA1D	9606.ENSP00000369960	9606.ENSP00000368766	0.062	0.213	0.8	0.839
ADRA1A	TBXA2R	9606.ENSP00000369960	9606.ENSP00000393333	0	0	0.5	0.499
ADRA1A	BTK	9606.ENSP00000369960	9606.ENSP00000483570	0	0.056	0.65	0.655
ADRA1B	HTR2C	9606.ENSP00000306662	9606.ENSP00000276198	0	0	0.9	0.9
ADRA1B	CCR5	9606.ENSP00000306662	9606.ENSP00000292303	0	0	0.9	0.9
ADRA1B	PTGER1	9606.ENSP00000306662	9606.ENSP00000292513	0	0	0.9	0.9
ADRA1B	CHRM1	9606.ENSP00000306662	9606.ENSP00000306490	0.076	0	0.9	0.903
ADRA1B	BTK	9606.ENSP00000306662	9606.ENSP00000483570	0	0.056	0.65	0.655

ADRA1B	TBXA2R	9606.ENSP00000306662	9606.ENSP00000393333	0.062	0	0.5	0.51
ADRA1B	ADRA1D	9606.ENSP00000306662	9606.ENSP00000368766	0.111	0.27	0.8	0.858
ADRA1B	F2	9606.ENSP00000306662	9606.ENSP00000308541	0.066	0.097	0.9	0.908
ADRA1B	CXCL12	9606.ENSP00000306662	9606.ENSP00000379140	0	0.076	0.9	0.903
ADRA1B	TGM2	9606.ENSP00000306662	9606.ENSP00000355330	0.077	0	0.9	0.903
ADRA1D	BTK	9606.ENSP00000368766	9606.ENSP00000483570	0	0.056	0.65	0.655
ADRA1D	TBXA2R	9606.ENSP00000368766	9606.ENSP00000393333	0	0	0.5	0.499
ADRA2A	GNAO1	9606.ENSP00000280155	9606.ENSP00000262494	0.086	0.549	0	0.57
ADRA2A	PRKCQ	9606.ENSP00000280155	9606.ENSP00000263125	0	0	0.65	0.65
ADRA2A	PRKCG	9606.ENSP00000280155	9606.ENSP00000263431	0.062	0.056	0.65	0.663
ADRA2A	ADRA2C	9606.ENSP00000280155	9606.ENSP00000386069	0.062	0.312	0.3	0.508
ADRA2A	PRKCB	9606.ENSP00000280155	9606.ENSP00000305355	0.062	0.056	0.65	0.663
ADRA2A	PRKCE	9606.ENSP00000280155	9606.ENSP00000306124	0	0	0.65	0.65
ADRA2A	PRKCA	9606.ENSP00000280155	9606.ENSP00000408695	0.062	0.056	0.65	0.663
ADRA2A	PRKCH	9606.ENSP00000280155	9606.ENSP00000329127	0	0	0.65	0.65
ADRA2A	PRKCD	9606.ENSP00000280155	9606.ENSP00000378217	0	0.213	0.65	0.712
ADRA2A	GNAI1	9606.ENSP00000280155	9606.ENSP00000343027	0	0.303	0.6	0.709
ADRA2B	PRKCQ	9606.ENSP00000480573	9606.ENSP00000263125	0	0	0.65	0.65
ADRA2B	PRKCG	9606.ENSP00000480573	9606.ENSP00000263431	0.062	0.056	0.65	0.663
ADRA2B	PRKCB	9606.ENSP00000480573	9606.ENSP00000305355	0.062	0.056	0.65	0.663
ADRA2B	PRKCE	9606.ENSP00000480573	9606.ENSP00000306124	0	0	0.65	0.65
ADRA2B	PRKCH	9606.ENSP00000480573	9606.ENSP00000329127	0	0	0.65	0.65
ADRA2B	PRKCD	9606.ENSP00000480573	9606.ENSP00000378217	0	0	0.65	0.65
ADRA2B	PRKCA	9606.ENSP00000480573	9606.ENSP00000408695	0.062	0.056	0.65	0.663
ADRA2C	PRKCQ	9606.ENSP00000386069	9606.ENSP00000263125	0	0	0.65	0.65
ADRA2C	PRKCG	9606.ENSP00000386069	9606.ENSP00000263431	0.062	0.056	0.65	0.663
ADRA2C	PRKCB	9606.ENSP00000386069	9606.ENSP00000305355	0.062	0.056	0.65	0.663
ADRA2C	PRKCE	9606.ENSP00000386069	9606.ENSP00000306124	0	0	0.65	0.65
ADRA2C	PRKCH	9606.ENSP00000386069	9606.ENSP00000329127	0	0	0.65	0.65
ADRA2C	GNAI1	9606.ENSP00000386069	9606.ENSP00000343027	0.062	0.151	0.6	0.653
ADRA2C	PRKCD	9606.ENSP00000386069	9606.ENSP00000378217	0	0	0.65	0.65
ADRA2C	PRKCA	9606.ENSP00000386069	9606.ENSP00000408695	0.062	0.056	0.65	0.663
ADRB3	GNAI1	9606.ENSP00000343782	9606.ENSP00000343027	0	0.151	0.8	0.822
ADRB3	GNAI3	9606.ENSP00000343782	9606.ENSP00000358867	0	0.151	0.8	0.822
AHR	EP300	9606.ENSP00000242057	9606.ENSP00000263253	0.062	0.655	0	0.662
AHR	NCOR2	9606.ENSP00000242057	9606.ENSP00000384018	0	0.486	0	0.485
AHR	AR	9606.ENSP00000242057	9606.ENSP00000363822	0	0.47	0	0.47
AHR	RELA	9606.ENSP00000242057	9606.ENSP00000384273	0	0.486	0	0.485
AHR	ESR1	9606.ENSP00000242057	9606.ENSP00000405330	0	0.873	0	0.873
AHR	HSP90AA1	9606.ENSP00000242057	9606.ENSP00000335153	0	0.885	0.9	0.988
AKR1B1	GCK	9606.ENSP00000285930	9606.ENSP00000223366	0.066	0.056	0.8	0.808
AKR1B1	ALDH2	9606.ENSP00000285930	9606.ENSP00000261733	0.109	0.102	0.65	0.695
AKR1B1	MGLL	9606.ENSP00000285930	9606.ENSP00000265052	0.063	0	0.9	0.902
AKR1B1	QDPR	9606.ENSP00000285930	9606.ENSP00000281243	0.062	0.248	0.9	0.923

AKR1B1	FABP5	9606.ENSP00000285930	9606.ENSP00000297258	0.064	0.684	0	0.691
AKR1B1	GLO1	9606.ENSP00000285930	9606.ENSP00000362463	0.167	0.346	0	0.432
AKR1B1	GLB1	9606.ENSP00000285930	9606.ENSP00000306920	0.052	0	0.9	0.901
AKR1B1	AKR1C3	9606.ENSP00000285930	9606.ENSP00000369927	0	0	0.9	0.9
AKR1B1	AKR1B10	9606.ENSP00000285930	9606.ENSP00000352584	0.099	0	0.9	0.906
AKR1B1	DHFR	9606.ENSP00000285930	9606.ENSP00000396308	0	0	0.9	0.9
AKR1B10	GCK	9606.ENSP00000352584	9606.ENSP00000223366	0.066	0.056	0.8	0.808
AKR1B10	ALDH2	9606.ENSP00000352584	9606.ENSP00000261733	0.109	0.102	0.65	0.695
AKR1B10	MGLL	9606.ENSP00000352584	9606.ENSP00000265052	0.063	0	0.9	0.902
AKR1B10	QDPR	9606.ENSP00000352584	9606.ENSP00000281243	0.062	0.248	0.9	0.923
AKR1B10	GLB1	9606.ENSP00000352584	9606.ENSP00000306920	0.052	0	0.9	0.901
AKR1B10	DHFR	9606.ENSP00000352584	9606.ENSP00000396308	0	0	0.9	0.9
AKR1B10	AKR1C3	9606.ENSP00000352584	9606.ENSP00000369927	0.102	0	0.9	0.906
AKR1C3	HSD17B2	9606.ENSP00000369927	9606.ENSP00000199936	0.062	0	0.8	0.804
AKR1C3	STS	9606.ENSP00000369927	9606.ENSP00000217961	0	0	0.9	0.9
AKR1C3	SRD5A1	9606.ENSP00000369927	9606.ENSP00000274192	0	0	0.9	0.9
AKR1C3	CYP11B1	9606.ENSP00000369927	9606.ENSP00000292427	0.062	0	0.9	0.902
AKR1C3	HPGDS	9606.ENSP00000369927	9606.ENSP00000295256	0	0.14	0.9	0.91
AKR1C3	UGT2B7	9606.ENSP00000369927	9606.ENSP00000304811	0.107	0	0.65	0.674
AKR1C3	CYP11B2	9606.ENSP00000369927	9606.ENSP00000325822	0.062	0	0.9	0.902
AKR1C3	CYP3A4	9606.ENSP00000369927	9606.ENSP00000337915	0.062	0	0.9	0.902
AKR1C3	CYP1A2	9606.ENSP00000369927	9606.ENSP00000342007	0.062	0	0.9	0.902
AKR1C3	CYP17A1	9606.ENSP00000369927	9606.ENSP00000358903	0.062	0	0.9	0.902
AKR1C3	HSD17B3	9606.ENSP00000369927	9606.ENSP00000364412	0.053	0	0.8	0.802
AKR1C3	HSD17B1	9606.ENSP00000369927	9606.ENSP00000466799	0.062	0.104	0.8	0.817
AKR1C3	CYP19A1	9606.ENSP00000369927	9606.ENSP00000379683	0.062	0	0.9	0.902
AKR1C3	SRD5A2	9606.ENSP00000369927	9606.ENSP00000477587	0.052	0	0.9	0.901
AKT1	MAPK1	9606.ENSP00000451828	9606.ENSP00000215832	0.068	0.148	0.8	0.827
AKT1	AURKA	9606.ENSP00000451828	9606.ENSP00000216911	0	0.242	0.9	0.921
AKT1	GSK3A	9606.ENSP00000451828	9606.ENSP00000222330	0.081	0.9	0.9	0.99
AKT1	RPS6KB1	9606.ENSP00000451828	9606.ENSP00000225577	0.183	0.764	0.9	0.979
AKT1	NFKB1	9606.ENSP00000451828	9606.ENSP00000226574	0.066	0.176	0.9	0.916
AKT1	MAPK14	9606.ENSP00000451828	9606.ENSP00000229795	0.083	0.83	0.6	0.932
AKT1	LNPEP	9606.ENSP00000451828	9606.ENSP00000231368	0.064	0	0.9	0.902
AKT1	RAF1	9606.ENSP00000451828	9606.ENSP00000251849	0	0.88	0.9	0.987
AKT1	RARA	9606.ENSP00000451828	9606.ENSP00000254066	0.088	0.505	0.9	0.95
AKT1	MDM2	9606.ENSP00000451828	9606.ENSP00000258149	0	0.898	0.9	0.989
AKT1	PDGFRB	9606.ENSP00000451828	9606.ENSP00000261799	0.062	0.122	0.9	0.91
AKT1	GNAO1	9606.ENSP00000451828	9606.ENSP00000262494	0.053	0.159	0.9	0.913
AKT1	MAPK3	9606.ENSP00000451828	9606.ENSP00000263025	0.066	0.148	0.8	0.826
AKT1	EP300	9606.ENSP00000451828	9606.ENSP00000263253	0	0.885	0.9	0.988
AKT1	AKT3	9606.ENSP00000451828	9606.ENSP00000263826	0	0.13	0.8	0.818
AKT1	PIK3CA	9606.ENSP00000451828	9606.ENSP00000263967	0.064	0.88	0.9	0.987
AKT1	STAT3	9606.ENSP00000451828	9606.ENSP00000264657	0	0.14	0.9	0.91

AKT1	EGFR	9606.ENSP00000451828	9606.ENSP00000275493	0	0.65	0	0.65
AKT1	PDE3B	9606.ENSP00000451828	9606.ENSP00000282096	0	0.236	0.8	0.84
AKT1	BRAF	9606.ENSP00000451828	9606.ENSP00000288602	0	0.744	0	0.744
AKT1	PIK3CB	9606.ENSP00000451828	9606.ENSP00000289153	0.064	0.395	0.9	0.938
AKT1	NOS3	9606.ENSP00000451828	9606.ENSP00000297494	0.049	0.879	0.9	0.987
AKT1	HDAC3	9606.ENSP00000451828	9606.ENSP00000302967	0	0.505	0	0.505
AKT1	INSR	9606.ENSP00000451828	9606.ENSP00000303830	0.052	0.182	0.9	0.915
AKT1	PRKCB	9606.ENSP00000451828	9606.ENSP00000305355	0	0.456	0	0.456
AKT1	S1PR1	9606.ENSP00000451828	9606.ENSP00000305416	0	0.226	0.9	0.919
AKT1	YWHAG	9606.ENSP00000451828	9606.ENSP00000306330	0.074	0.364	0.9	0.936
AKT1	TERT	9606.ENSP00000451828	9606.ENSP00000309572	0.055	0.723	0.9	0.971
AKT1	PRKDC	9606.ENSP00000451828	9606.ENSP00000313420	0.062	0.701	0.9	0.969
AKT1	EDNRA	9606.ENSP00000451828	9606.ENSP00000315011	0	0.056	0.9	0.901
AKT1	GSK3B	9606.ENSP00000451828	9606.ENSP00000324806	0.049	0.934	0.9	0.993
AKT1	NOS2	9606.ENSP00000451828	9606.ENSP00000327251	0.063	0.077	0.9	0.905
AKT1	CASP9	9606.ENSP00000451828	9606.ENSP00000330237	0	0.231	0.9	0.919
AKT1	RARB	9606.ENSP00000451828	9606.ENSP00000332296	0	0.077	0.9	0.903
AKT1	HSP90AA1	9606.ENSP00000451828	9606.ENSP00000335153	0.062	0.946	0.9	0.994
AKT1	LCK	9606.ENSP00000451828	9606.ENSP00000337825	0.049	0	0.9	0.9
AKT1	MAPT	9606.ENSP00000451828	9606.ENSP00000340820	0	0.709	0	0.709
AKT1	PTPN11	9606.ENSP00000451828	9606.ENSP00000340944	0.09	0.138	0.9	0.914
AKT1	GNAI1	9606.ENSP00000451828	9606.ENSP00000343027	0	0.159	0.9	0.912
AKT1	JAK1	9606.ENSP00000451828	9606.ENSP00000343204	0.062	0	0.9	0.902
AKT1	PDPK1	9606.ENSP00000451828	9606.ENSP00000344220	0.066	0.936	0.9	0.993
AKT1	RAC1	9606.ENSP00000451828	9606.ENSP00000348461	0.063	0.345	0.9	0.933
AKT1	VCP	9606.ENSP00000451828	9606.ENSP00000351777	0.073	0.676	0	0.687
AKT1	PIK3CG	9606.ENSP00000451828	9606.ENSP00000352121	0.063	0.184	0.9	0.916
AKT1	RXRG	9606.ENSP00000451828	9606.ENSP00000352900	0	0.077	0.9	0.903
AKT1	MTOR	9606.ENSP00000451828	9606.ENSP00000354558	0.081	0.904	0.9	0.99
AKT1	MAPKAPK2	9606.ENSP00000451828	9606.ENSP00000356070	0.089	0.736	0	0.749
AKT1	MCL1	9606.ENSP00000451828	9606.ENSP00000358022	0	0	0.9	0.9
AKT1	GNAI3	9606.ENSP00000451828	9606.ENSP00000358867	0	0.159	0.9	0.912
AKT1	JUN	9606.ENSP00000451828	9606.ENSP00000360266	0	0.057	0.8	0.803
AKT1	PTPN1	9606.ENSP00000451828	9606.ENSP00000360683	0.091	0.284	0.9	0.929
AKT1	SRC	9606.ENSP00000451828	9606.ENSP00000362680	0.098	0.683	0.9	0.968
AKT1	RXRB	9606.ENSP00000451828	9606.ENSP00000363817	0.07	0.077	0.9	0.906
AKT1	AR	9606.ENSP00000451828	9606.ENSP00000363822	0	0.87	0	0.87
AKT1	S1PR3	9606.ENSP00000451828	9606.ENSP00000365006	0	0	0.9	0.9
AKT1	PIK3CD	9606.ENSP00000451828	9606.ENSP00000366563	0.064	0.204	0.6	0.675
AKT1	PRKCZ	9606.ENSP00000451828	9606.ENSP00000367830	0.064	0.865	0.9	0.986
AKT1	TNK2	9606.ENSP00000451828	9606.ENSP00000371341	0.085	0.498	0	0.521
AKT1	PDK1	9606.ENSP00000451828	9606.ENSP00000376352	0.065	0.549	0	0.56
AKT1	NR2F2	9606.ENSP00000451828	9606.ENSP00000377721	0	0.497	0	0.497
AKT1	NCOR2	9606.ENSP00000451828	9606.ENSP00000384018	0.089	0.282	0.9	0.929

AKT1	RARG	9606.ENSP00000451828	9606.ENSP00000388510	0.065	0.077	0.9	0.906
AKT1	GLI2	9606.ENSP00000451828	9606.ENSP00000390436	0.055	0.067	0.9	0.904
AKT1	JAK3	9606.ENSP00000451828	9606.ENSP00000391676	0.049	0	0.9	0.9
AKT1	ESR1	9606.ENSP00000451828	9606.ENSP00000405330	0.055	0.297	0.9	0.927
AKT1	RXRA	9606.ENSP00000451828	9606.ENSP00000419692	0.082	0.077	0.9	0.907
AKT1	PIK3R1	9606.ENSP00000451828	9606.ENSP00000428056	0.062	0.7	0.9	0.969
AKT1	LYN	9606.ENSP00000451828	9606.ENSP00000428924	0.049	0	0.65	0.652
AKT1	STK3	9606.ENSP00000451828	9606.ENSP00000429744	0.065	0.494	0	0.506
AKT1	IKBKB	9606.ENSP00000451828	9606.ENSP00000430684	0	0.784	0.9	0.977
AKT1	HIF1A	9606.ENSP00000451828	9606.ENSP00000437955	0	0.261	0.9	0.923
AKT1	NR4A1	9606.ENSP00000451828	9606.ENSP00000440864	0	0.282	0.9	0.925
AKT1	YES1	9606.ENSP00000451828	9606.ENSP00000462468	0.064	0.059	0.65	0.664
AKT1	NOS1	9606.ENSP00000451828	9606.ENSP00000477999	0.049	0.077	0.8	0.809
AKT1	IKBKG	9606.ENSP00000451828	9606.ENSP00000483825	0.076	0.452	0.9	0.944
AKT3	MAPK1	9606.ENSP00000263826	9606.ENSP00000215832	0.066	0.148	0.8	0.826
AKT3	GSK3A	9606.ENSP00000263826	9606.ENSP00000222330	0.051	0.181	0.9	0.915
AKT3	RPS6KB1	9606.ENSP00000263826	9606.ENSP00000225577	0.183	0.154	0.8	0.849
AKT3	MAPK14	9606.ENSP00000263826	9606.ENSP00000229795	0.065	0.148	0.6	0.653
AKT3	RAF1	9606.ENSP00000263826	9606.ENSP00000251849	0	0.057	0.8	0.803
AKT3	MDM2	9606.ENSP00000263826	9606.ENSP00000258149	0	0.056	0.9	0.901
AKT3	MAPK3	9606.ENSP00000263826	9606.ENSP00000263025	0.066	0.148	0.8	0.826
AKT3	YES1	9606.ENSP00000263826	9606.ENSP00000462468	0.064	0.059	0.65	0.664
AKT3	LCK	9606.ENSP00000263826	9606.ENSP00000337825	0.049	0	0.65	0.652
AKT3	LYN	9606.ENSP00000263826	9606.ENSP00000428924	0.049	0	0.65	0.652
AKT3	RAC1	9606.ENSP00000263826	9606.ENSP00000348461	0.063	0.14	0.65	0.693
AKT3	SRC	9606.ENSP00000263826	9606.ENSP00000362680	0.064	0.059	0.65	0.664
AKT3	NOS1	9606.ENSP00000263826	9606.ENSP00000477999	0.049	0.077	0.8	0.809
AKT3	PDE3B	9606.ENSP00000263826	9606.ENSP00000282096	0	0.07	0.8	0.806
AKT3	NOS2	9606.ENSP00000263826	9606.ENSP00000327251	0.065	0.077	0.8	0.812
AKT3	IKBKB	9606.ENSP00000263826	9606.ENSP00000430684	0	0	0.8	0.8
AKT3	IKBKG	9606.ENSP00000263826	9606.ENSP00000483825	0	0.059	0.8	0.803
AKT3	PIK3CD	9606.ENSP00000263826	9606.ENSP00000366563	0.064	0.204	0.6	0.675
AKT3	NOS3	9606.ENSP00000263826	9606.ENSP00000297494	0.049	0.077	0.8	0.809
AKT3	JUN	9606.ENSP00000263826	9606.ENSP00000360266	0	0.057	0.8	0.803
AKT3	PIK3CB	9606.ENSP00000263826	9606.ENSP00000289153	0.064	0.204	0.6	0.675
AKT3	PIK3R1	9606.ENSP00000263826	9606.ENSP00000428056	0.062	0.102	0.65	0.679
AKT3	NR4A1	9606.ENSP00000263826	9606.ENSP00000440864	0	0.057	0.9	0.901
AKT3	HSP90AA1	9606.ENSP00000263826	9606.ENSP00000335153	0.062	0.647	0.65	0.874
AKT3	PIK3CG	9606.ENSP00000263826	9606.ENSP00000352121	0.063	0.184	0.8	0.833
AKT3	PIK3CA	9606.ENSP00000263826	9606.ENSP00000263967	0.064	0.204	0.65	0.716
AKT3	PRKCZ	9606.ENSP00000263826	9606.ENSP00000367830	0.064	0.674	0.8	0.933
AKT3	CASP9	9606.ENSP00000263826	9606.ENSP00000330237	0	0.063	0.9	0.902
AKT3	PDPK1	9606.ENSP00000263826	9606.ENSP00000344220	0.062	0.463	0.9	0.945
AKT3	MTOR	9606.ENSP00000263826	9606.ENSP00000354558	0.062	0.104	0.9	0.908

AKT3	GSK3B	9606.ENSP00000263826	9606.ENSP00000324806	0	0.563	0.9	0.954
ALB	TTR	9606.ENSP00000295897	9606.ENSP00000237014	0.297	0.348	0	0.522
ALB	FABP1	9606.ENSP00000295897	9606.ENSP00000295834	0.59	0	0	0.59
ALB	RXRA	9606.ENSP00000295897	9606.ENSP00000419692	0.062	0.058	0.65	0.663
ALB	RBP4	9606.ENSP00000295897	9606.ENSP00000360522	0.377	0.465	0	0.653
ALB	NR1H4	9606.ENSP00000295897	9606.ENSP00000447149	0.234	0.058	0.65	0.725
ALB	F2	9606.ENSP00000295897	9606.ENSP00000308541	0.624	0.073	0	0.637
ALB	SERPINA6	9606.ENSP00000295897	9606.ENSP00000342850	0.379	0.151	0	0.451
ALDH1A1	CYP2C9	9606.ENSP00000297785	9606.ENSP00000260682	0.063	0.061	0.65	0.665
ALDH1A1	UGT2B7	9606.ENSP00000297785	9606.ENSP00000304811	0.082	0	0.65	0.664
ALDH1A1	CYP2A6	9606.ENSP00000297785	9606.ENSP00000301141	0.063	0.061	0.9	0.904
ALDH1A1	CYP1A2	9606.ENSP00000297785	9606.ENSP00000342007	0	0.061	0.9	0.902
ALDH1A1	CYP3A4	9606.ENSP00000297785	9606.ENSP00000337915	0.065	0.061	0.9	0.904
ALDH2	GLUL	9606.ENSP00000261733	9606.ENSP00000307900	0.08	0	0.8	0.808
ALDH2	MAOB	9606.ENSP00000261733	9606.ENSP00000367309	0.083	0	0.9	0.904
ALDH2	COMT	9606.ENSP00000261733	9606.ENSP00000354511	0.062	0	0.9	0.902
ALDH2	AOC3	9606.ENSP00000261733	9606.ENSP00000312326	0.139	0.165	0.9	0.921
ALDH2	MAOA	9606.ENSP00000261733	9606.ENSP00000340684	0.064	0	0.9	0.902
ALDH3A1	HPGDS	9606.ENSP00000411821	9606.ENSP00000295256	0	0.104	0.65	0.672
ALDH3A1	AOC3	9606.ENSP00000411821	9606.ENSP00000312326	0.065	0.08	0.9	0.906
ALDH3A1	MAOA	9606.ENSP00000411821	9606.ENSP00000340684	0.064	0	0.9	0.902
ALDH3A1	COMT	9606.ENSP00000411821	9606.ENSP00000354511	0	0	0.9	0.9
ALDH3A1	MAOB	9606.ENSP00000411821	9606.ENSP00000367309	0	0	0.9	0.9
ALK	PIK3CA	9606.ENSP00000373700	9606.ENSP00000263967	0.062	0.104	0.8	0.817
ALK	ERBB2	9606.ENSP00000373700	9606.ENSP00000269571	0.049	0.415	0	0.42
ALK	KIT	9606.ENSP00000373700	9606.ENSP00000288135	0.062	0.392	0	0.405
ALK	PIK3CB	9606.ENSP00000373700	9606.ENSP00000289153	0.062	0.317	0.8	0.86
ALK	JAK1	9606.ENSP00000373700	9606.ENSP00000343204	0.064	0	0.8	0.804
ALK	RET	9606.ENSP00000373700	9606.ENSP00000347942	0.062	0.391	0	0.404
ALK	PIK3CD	9606.ENSP00000373700	9606.ENSP00000366563	0.062	0.104	0.8	0.817
ALK	JAK2	9606.ENSP00000373700	9606.ENSP00000371067	0.064	0.27	0.8	0.851
ALK	PIK3R1	9606.ENSP00000373700	9606.ENSP00000428056	0	0.316	0.6	0.714
ALK	JAK3	9606.ENSP00000373700	9606.ENSP00000391676	0.064	0.486	0.8	0.895
ALOX12	PLA2G2C	9606.ENSP00000251535	9606.ENSP00000247992	0	0	0.65	0.65
ALOX12	PLA2G5	9606.ENSP00000251535	9606.ENSP00000364249	0	0	0.65	0.65
ALOX12	PLA2G4B	9606.ENSP00000251535	9606.ENSP00000396045	0	0	0.65	0.65
ALOX12	PLA2G6	9606.ENSP00000251535	9606.ENSP00000333142	0	0	0.65	0.65
ALOX12	PLA2G10	9606.ENSP00000251535	9606.ENSP00000393847	0	0	0.65	0.65
ALOX12	PLA2G1B	9606.ENSP00000251535	9606.ENSP00000312286	0	0	0.65	0.65
ALOX12	PLA2G2A	9606.ENSP00000251535	9606.ENSP00000383364	0.062	0.27	0.65	0.739
ALOX12	PLA2G4A	9606.ENSP00000251535	9606.ENSP00000356436	0	0.27	0.65	0.733
ALOX12	ALOX15	9606.ENSP00000251535	9606.ENSP00000458832	0.062	0	0.9	0.902
ALOX12	CYP2C19	9606.ENSP00000251535	9606.ENSP00000360372	0	0	0.9	0.9
ALOX12	ALOX5	9606.ENSP00000251535	9606.ENSP00000363512	0.063	0	0.9	0.902

ALOX12	CYP2C9	9606.ENSP00000251535	9606.ENSP00000260682	0	0	0.9	0.9
ALOX12	PTGS2	9606.ENSP00000251535	9606.ENSP00000356438	0.062	0	0.9	0.902
ALOX12	PTGS1	9606.ENSP00000251535	9606.ENSP00000354612	0.107	0	0.9	0.906
ALOX15	PLA2G2C	9606.ENSP00000458832	9606.ENSP00000247992	0	0	0.65	0.65
ALOX15	CYP2C9	9606.ENSP00000458832	9606.ENSP00000260682	0	0	0.9	0.9
ALOX15	PLA2G1B	9606.ENSP00000458832	9606.ENSP00000312286	0	0	0.65	0.65
ALOX15	PLA2G6	9606.ENSP00000458832	9606.ENSP00000333142	0	0	0.65	0.65
ALOX15	CYP3A4	9606.ENSP00000458832	9606.ENSP00000337915	0	0	0.9	0.9
ALOX15	CYP1A2	9606.ENSP00000458832	9606.ENSP00000342007	0	0	0.9	0.9
ALOX15	PTGS1	9606.ENSP00000458832	9606.ENSP00000354612	0.062	0	0.9	0.902
ALOX15	PLA2G4A	9606.ENSP00000458832	9606.ENSP00000356436	0	0	0.65	0.65
ALOX15	PTGS2	9606.ENSP00000458832	9606.ENSP00000356438	0.062	0	0.9	0.902
ALOX15	CYP2C19	9606.ENSP00000458832	9606.ENSP00000360372	0	0	0.9	0.9
ALOX15	ALOX5	9606.ENSP00000458832	9606.ENSP00000363512	0.062	0	0.9	0.902
ALOX15	PLA2G5	9606.ENSP00000458832	9606.ENSP00000364249	0	0	0.65	0.65
ALOX15	PLA2G2A	9606.ENSP00000458832	9606.ENSP00000383364	0	0	0.65	0.65
ALOX15	PLA2G10	9606.ENSP00000458832	9606.ENSP00000393847	0	0	0.65	0.65
ALOX15	PLA2G4B	9606.ENSP00000458832	9606.ENSP00000396045	0	0	0.65	0.65
ALOX5	LTA4H	9606.ENSP00000363512	9606.ENSP00000228740	0	0	0.9	0.9
ALOX5	PLA2G2C	9606.ENSP00000363512	9606.ENSP00000247992	0	0	0.65	0.65
ALOX5	CYP2C9	9606.ENSP00000363512	9606.ENSP00000260682	0	0	0.9	0.9
ALOX5	PLA2G1B	9606.ENSP00000363512	9606.ENSP00000312286	0	0	0.65	0.65
ALOX5	PLA2G6	9606.ENSP00000363512	9606.ENSP00000333142	0	0	0.65	0.65
ALOX5	PTGS1	9606.ENSP00000363512	9606.ENSP00000354612	0.096	0	0.9	0.905
ALOX5	MAPKAPK2	9606.ENSP00000363512	9606.ENSP00000356070	0.062	0.679	0.9	0.967
ALOX5	PLA2G4A	9606.ENSP00000363512	9606.ENSP00000356436	0.062	0	0.65	0.657
ALOX5	PTGS2	9606.ENSP00000363512	9606.ENSP00000356438	0.081	0	0.9	0.904
ALOX5	CYP2C19	9606.ENSP00000363512	9606.ENSP00000360372	0	0	0.9	0.9
ALOX5	FGR	9606.ENSP00000363512	9606.ENSP00000363117	0.404	0.27	0	0.546
ALOX5	ITGB2	9606.ENSP00000363512	9606.ENSP00000380948	0.548	0	0	0.548
ALOX5	HCK	9606.ENSP00000363512	9606.ENSP00000444986	0.535	0.27	0	0.646
ALOX5	PLA2G4B	9606.ENSP00000363512	9606.ENSP00000396045	0	0	0.65	0.65
ALOX5	PLA2G5	9606.ENSP00000363512	9606.ENSP00000364249	0	0	0.65	0.65
ALOX5	PLA2G10	9606.ENSP00000363512	9606.ENSP00000393847	0	0	0.65	0.65
ALOX5	PLA2G2A	9606.ENSP00000363512	9606.ENSP00000383364	0.062	0	0.65	0.657
ANPEP	LAP3	9606.ENSP00000300060	9606.ENSP00000226299	0.083	0.15	0.8	0.83
AOC3	GLO1	9606.ENSP00000312326	9606.ENSP00000362463	0	0	0.8	0.8
AOC3	COMT	9606.ENSP00000312326	9606.ENSP00000354511	0	0	0.9	0.9
AOC3	MAOA	9606.ENSP00000312326	9606.ENSP00000340684	0.082	0	0.8	0.808
AOC3	MAOB	9606.ENSP00000312326	9606.ENSP00000367309	0.089	0	0.8	0.81
APH1A	APH1B	9606.ENSP00000358105	9606.ENSP00000261879	0	0	0.9	0.9
APH1A	APP	9606.ENSP00000358105	9606.ENSP00000284981	0	0.8	0.9	0.979
APH1A	NCSTN	9606.ENSP00000358105	9606.ENSP00000294785	0.088	0.985	0.9	0.998
APH1A	PSEN1	9606.ENSP00000358105	9606.ENSP00000326366	0.066	0.982	0.9	0.998

APH1A	EPHB3	9606.ENSP00000358105	9606.ENSP00000332118	0.052	0	0.9	0.901
APH1A	EPHB4	9606.ENSP00000358105	9606.ENSP00000350896	0.062	0	0.9	0.902
APH1A	PSEN2	9606.ENSP00000358105	9606.ENSP00000355747	0.066	0.786	0.8	0.956
APH1A	PSENEN	9606.ENSP00000358105	9606.ENSP00000468411	0.088	0.984	0.9	0.998
APH1B	EPHB4	9606.ENSP00000261879	9606.ENSP00000350896	0.051	0	0.9	0.901
APH1B	EPHB3	9606.ENSP00000261879	9606.ENSP00000332118	0.052	0	0.9	0.901
APH1B	APP	9606.ENSP00000261879	9606.ENSP00000284981	0.052	0.044	0.9	0.901
APH1B	PSEN2	9606.ENSP00000261879	9606.ENSP00000355747	0.066	0.661	0.8	0.931
APH1B	PSEN1	9606.ENSP00000261879	9606.ENSP00000326366	0.066	0.646	0.9	0.964
APH1B	PSENEN	9606.ENSP00000261879	9606.ENSP00000468411	0.088	0.519	0.9	0.952
APH1B	NCSTN	9606.ENSP00000261879	9606.ENSP00000294785	0.065	0.646	0.9	0.964
APOBEC3A	NDUFS7	9606.ENSP00000384359	9606.ENSP00000233627	0.241	0.343	0	0.48
APOBEC3A	NDUFA2	9606.ENSP00000384359	9606.ENSP00000252102	0.082	0.529	0	0.549
APOBEC3A	NDUFA10	9606.ENSP00000384359	9606.ENSP00000252711	0.184	0.533	0	0.602
APOBEC3A	NDUFB5	9606.ENSP00000384359	9606.ENSP00000259037	0.167	0.346	0	0.431
APOBEC3A	NDUFS3	9606.ENSP00000384359	9606.ENSP00000263774	0.264	0.254	0	0.427
APOBEC3A	NDUFA9	9606.ENSP00000384359	9606.ENSP00000266544	0.156	0.53	0	0.586
APOBEC3A	NDUFB10	9606.ENSP00000384359	9606.ENSP00000268668	0.246	0.524	0	0.625
APOBEC3A	NDUFS6	9606.ENSP00000384359	9606.ENSP00000274137	0.144	0.531	0	0.581
APOBEC3A	NDUFB11	9606.ENSP00000384359	9606.ENSP00000276062	0.189	0.528	0	0.6
APOBEC3A	NDUFB9	9606.ENSP00000384359	9606.ENSP00000276689	0.241	0.404	0	0.528
APOBEC3A	NDUFC2	9606.ENSP00000384359	9606.ENSP00000281031	0.166	0.372	0	0.453
APOBEC3A	NDUFA7	9606.ENSP00000384359	9606.ENSP00000301457	0.153	0.388	0	0.459
APOBEC3A	NDUFS8	9606.ENSP00000384359	9606.ENSP00000315774	0.344	0.375	0	0.572
APOBEC3A	NDUFV2	9606.ENSP00000384359	9606.ENSP00000327268	0.166	0.346	0	0.431
APOBEC3A	NDUFA12	9606.ENSP00000384359	9606.ENSP00000330737	0.167	0.533	0	0.594
APOBEC3A	NDUFS2	9606.ENSP00000384359	9606.ENSP00000356972	0.167	0.533	0	0.594
APOBEC3A	NDUFS5	9606.ENSP00000384359	9606.ENSP00000362060	0.098	0.528	0	0.556
APOBEC3A	NDUFA8	9606.ENSP00000384359	9606.ENSP00000362873	0.184	0.318	0	0.419
APOBEC3A	NDUFB6	9606.ENSP00000384359	9606.ENSP00000369176	0.145	0.346	0	0.416
APOBEC3A	NDUFS1	9606.ENSP00000384359	9606.ENSP00000392709	0.183	0.369	0	0.462
APOBEC3A	NDUFA13	9606.ENSP00000384359	9606.ENSP00000423673	0.188	0.367	0	0.464
APOBEC3A	NDUFA6	9606.ENSP00000384359	9606.ENSP00000418842	0.204	0.533	0	0.612
APOBEC3A	NDUFAB1	9606.ENSP00000384359	9606.ENSP00000458770	0.235	0.533	0	0.627
APOBEC3G	NDUFS7	9606.ENSP00000385057	9606.ENSP00000233627	0.247	0.343	0	0.484
APOBEC3G	NDUFA2	9606.ENSP00000385057	9606.ENSP00000252102	0.089	0.529	0	0.552
APOBEC3G	NDUFA10	9606.ENSP00000385057	9606.ENSP00000252711	0.184	0.533	0	0.602
APOBEC3G	NDUFB5	9606.ENSP00000385057	9606.ENSP00000259037	0.167	0.346	0	0.431
APOBEC3G	NDUFS3	9606.ENSP00000385057	9606.ENSP00000263774	0.269	0.254	0	0.431
APOBEC3G	NDUFA9	9606.ENSP00000385057	9606.ENSP00000266544	0.16	0.53	0	0.588
APOBEC3G	NDUFB10	9606.ENSP00000385057	9606.ENSP00000268668	0.25	0.524	0	0.628
APOBEC3G	NDUFS6	9606.ENSP00000385057	9606.ENSP00000274137	0.144	0.531	0	0.581
APOBEC3G	NDUFB11	9606.ENSP00000385057	9606.ENSP00000276062	0.194	0.528	0	0.603
APOBEC3G	NDUFB9	9606.ENSP00000385057	9606.ENSP00000276689	0.244	0.404	0	0.53

APOBEC3G	NDUFC2	9606.ENSP00000385057	9606.ENSP00000281031	0.181	0.372	0	0.464
APOBEC3G	NDUFA7	9606.ENSP00000385057	9606.ENSP00000301457	0.153	0.388	0	0.459
APOBEC3G	NDUFS8	9606.ENSP00000385057	9606.ENSP00000315774	0.348	0.375	0	0.575
APOBEC3G	NDUFV2	9606.ENSP00000385057	9606.ENSP00000327268	0.171	0.346	0	0.434
APOBEC3G	NDUFA12	9606.ENSP00000385057	9606.ENSP00000330737	0.173	0.533	0	0.597
APOBEC3G	NDUFS2	9606.ENSP00000385057	9606.ENSP00000356972	0.167	0.533	0	0.594
APOBEC3G	NDUFS5	9606.ENSP00000385057	9606.ENSP00000362060	0.11	0.528	0	0.562
APOBEC3G	NDUFA8	9606.ENSP00000385057	9606.ENSP00000362873	0.2	0.318	0	0.431
APOBEC3G	NDUFB6	9606.ENSP00000385057	9606.ENSP00000369176	0.159	0.346	0	0.426
APOBEC3G	NDUFS1	9606.ENSP00000385057	9606.ENSP00000392709	0.183	0.369	0	0.462
APOBEC3G	NDUFA13	9606.ENSP00000385057	9606.ENSP00000423673	0.198	0.367	0	0.47
APOBEC3G	NDUFA6	9606.ENSP00000385057	9606.ENSP00000418842	0.204	0.533	0	0.612
APOBEC3G	NDUFAB1	9606.ENSP00000385057	9606.ENSP00000458770	0.238	0.533	0	0.629
APP	CETP	9606.ENSP00000284981	9606.ENSP00000200676	0	0	0.72	0.72
APP	MAPK1	9606.ENSP00000284981	9606.ENSP00000215832	0.063	0.086	0.6	0.627
APP	GSK3A	9606.ENSP00000284981	9606.ENSP00000222330	0.058	0.626	0	0.633
APP	NFKB1	9606.ENSP00000284981	9606.ENSP00000226574	0	0.057	0.9	0.901
APP	CTSD	9606.ENSP00000284981	9606.ENSP00000236671	0.087	0.502	0	0.526
APP	TTR	9606.ENSP00000284981	9606.ENSP00000237014	0.065	0.756	0.5	0.876
APP	MSR1	9606.ENSP00000284981	9606.ENSP00000262101	0	0	0.54	0.54
APP	MAPK3	9606.ENSP00000284981	9606.ENSP00000263025	0.063	0.086	0.6	0.627
APP	KDR	9606.ENSP00000284981	9606.ENSP00000263923	0.076	0.488	0	0.507
APP	PLA2G7	9606.ENSP00000284981	9606.ENSP00000274793	0.062	0	0.72	0.726
APP	EGFR	9606.ENSP00000284981	9606.ENSP00000275493	0.126	0.642	0	0.673
APP	TGM2	9606.ENSP00000284981	9606.ENSP00000355330	0.052	0.431	0	0.437
APP	LIPC	9606.ENSP00000284981	9606.ENSP00000299022	0	0	0.54	0.54
APP	LPL	9606.ENSP00000284981	9606.ENSP00000309757	0.061	0	0.54	0.549
APP	PRSS1	9606.ENSP00000284981	9606.ENSP00000308720	0	0.651	0	0.651
APP	VCP	9606.ENSP00000284981	9606.ENSP00000351777	0	0.403	0	0.402
APP	F2	9606.ENSP00000284981	9606.ENSP00000308541	0	0.162	0.5	0.563
APP	CAPN1	9606.ENSP00000284981	9606.ENSP00000431984	0.058	0.042	0.65	0.656
APP	CHRNA7	9606.ENSP00000284981	9606.ENSP00000407546	0	0.545	0	0.545
APP	GRIN1	9606.ENSP00000284981	9606.ENSP00000360608	0.113	0.595	0	0.625
APP	HSP90AA1	9606.ENSP00000284981	9606.ENSP00000335153	0.053	0.494	0	0.5
APP	GRIN2B	9606.ENSP00000284981	9606.ENSP00000477455	0.062	0.486	0	0.497
APP	CRYAB	9606.ENSP00000284981	9606.ENSP00000433560	0.101	0.873	0	0.88
APP	GSK3B	9606.ENSP00000284981	9606.ENSP00000324806	0.06	0.698	0	0.704
APP	RELA	9606.ENSP00000284981	9606.ENSP00000384273	0	0	0.9	0.9
APP	CASP8	9606.ENSP00000284981	9606.ENSP00000351273	0	0.2	0.9	0.916
APP	MAPK10	9606.ENSP00000284981	9606.ENSP00000352157	0.089	0	0.9	0.905
APP	MAPK8	9606.ENSP00000284981	9606.ENSP00000378974	0.052	0.27	0.9	0.924
APP	MAPT	9606.ENSP00000284981	9606.ENSP00000340820	0.065	0.746	0	0.752
APP	PSEN2	9606.ENSP00000284981	9606.ENSP00000355747	0.048	0.478	0	0.482
APP	PSENEN	9606.ENSP00000284981	9606.ENSP00000468411	0	0.801	0.9	0.979

APP	BACE1	9606.ENSP00000284981	9606.ENSP00000318585	0.088	0.88	0.9	0.988
APP	PSEN1	9606.ENSP00000284981	9606.ENSP00000326366	0.048	0.974	0.9	0.997
APP	NCSTN	9606.ENSP00000284981	9606.ENSP00000294785	0.062	0.896	0.9	0.989
AR	HDAC7	9606.ENSP00000363822	9606.ENSP00000080059	0	0.298	0.9	0.926
AR	MAPK1	9606.ENSP00000363822	9606.ENSP00000215832	0	0.497	0	0.497
AR	CCND1	9606.ENSP00000363822	9606.ENSP00000227507	0.061	0.839	0.9	0.983
AR	MAPK14	9606.ENSP00000363822	9606.ENSP00000229795	0	0.104	0.9	0.906
AR	NR3C1	9606.ENSP00000363822	9606.ENSP00000231509	0	0.213	0.9	0.917
AR	CDK7	9606.ENSP00000363822	9606.ENSP00000256443	0	0.486	0	0.485
AR	VHL	9606.ENSP00000363822	9606.ENSP00000256474	0	0.494	0	0.494
AR	MDM2	9606.ENSP00000363822	9606.ENSP00000258149	0	0.898	0	0.898
AR	GNAO1	9606.ENSP00000363822	9606.ENSP00000262494	0	0.056	0.9	0.901
AR	EP300	9606.ENSP00000363822	9606.ENSP00000263253	0	0.881	0.9	0.987
AR	BRD4	9606.ENSP00000363822	9606.ENSP00000263377	0	0.644	0	0.644
AR	KAT2B	9606.ENSP00000363822	9606.ENSP00000263754	0	0.518	0.9	0.949
AR	PIK3CA	9606.ENSP00000363822	9606.ENSP00000263967	0	0.061	0.9	0.902
AR	HDAC4	9606.ENSP00000363822	9606.ENSP00000264606	0	0.505	0	0.505
AR	STAT3	9606.ENSP00000363822	9606.ENSP00000264657	0	0.68	0	0.68
AR	SMARCA2	9606.ENSP00000363822	9606.ENSP00000265773	0.062	0.505	0	0.516
AR	NCOR1	9606.ENSP00000363822	9606.ENSP00000268712	0	0.9	0	0.9
AR	EGFR	9606.ENSP00000363822	9606.ENSP00000275493	0.063	0.698	0	0.705
AR	HDAC3	9606.ENSP00000363822	9606.ENSP00000302967	0	0.505	0.65	0.819
AR	BRD3	9606.ENSP00000363822	9606.ENSP00000305918	0	0.518	0	0.518
AR	PRKDC	9606.ENSP00000363822	9606.ENSP00000313420	0	0.496	0	0.496
AR	GAK	9606.ENSP00000363822	9606.ENSP00000314499	0	0.485	0	0.485
AR	EZH2	9606.ENSP00000363822	9606.ENSP00000320147	0	0.692	0	0.692
AR	GSK3B	9606.ENSP00000363822	9606.ENSP00000324806	0	0.482	0.9	0.946
AR	PGR	9606.ENSP00000363822	9606.ENSP00000325120	0.069	0	0.6	0.611
AR	HSP90AA1	9606.ENSP00000363822	9606.ENSP00000335153	0	0.884	0.9	0.987
AR	GNAI1	9606.ENSP00000363822	9606.ENSP00000343027	0	0.056	0.9	0.901
AR	NR3C2	9606.ENSP00000363822	9606.ENSP00000350815	0.062	0.213	0.6	0.678
AR	CASP8	9606.ENSP00000363822	9606.ENSP00000351273	0	0.345	0.9	0.931
AR	GNAI3	9606.ENSP00000363822	9606.ENSP00000358867	0	0.056	0.9	0.901
AR	JUN	9606.ENSP00000363822	9606.ENSP00000360266	0	0.499	0	0.499
AR	KDM4A	9606.ENSP00000363822	9606.ENSP00000361473	0.049	0.4	0	0.405
AR	HDAC1	9606.ENSP00000363822	9606.ENSP00000362649	0	0.762	0	0.762
AR	SRC	9606.ENSP00000363822	9606.ENSP00000362680	0	0.839	0.9	0.983
AR	BRD2	9606.ENSP00000363822	9606.ENSP00000378704	0	0.518	0	0.518
AR	RELA	9606.ENSP00000363822	9606.ENSP00000384273	0	0.489	0	0.489
AR	HIF1A	9606.ENSP00000363822	9606.ENSP00000437955	0	0.521	0	0.521
AR	SMARCA4	9606.ENSP00000363822	9606.ENSP00000395654	0	0.687	0	0.687
AR	HSPA1A	9606.ENSP00000363822	9606.ENSP00000364802	0	0.498	0.65	0.817
AR	MAPK8	9606.ENSP00000363822	9606.ENSP00000378974	0	0	0.9	0.9
AR	KDM1A	9606.ENSP00000363822	9606.ENSP00000383042	0	0.705	0.6	0.876

AR	PIK3R1	9606.ENSP00000363822	9606.ENSP00000428056	0	0.154	0.9	0.911
AR	NCOR2	9606.ENSP00000363822	9606.ENSP00000384018	0	0.894	0	0.894
ASAH1	SPHK2	9606.ENSP00000371152	9606.ENSP00000245222	0	0	0.9	0.9
ASAH1	GLB1	9606.ENSP00000371152	9606.ENSP00000306920	0.075	0.383	0	0.405
ASAH1	SPHK1	9606.ENSP00000371152	9606.ENSP00000313681	0	0	0.9	0.9
ASAH1	GBA	9606.ENSP00000371152	9606.ENSP00000314508	0.065	0	0.9	0.902
ASAH1	DPP7	9606.ENSP00000371152	9606.ENSP00000360635	0.066	0.391	0	0.406
ASAH1	GBA2	9606.ENSP00000371152	9606.ENSP00000367343	0	0	0.9	0.9
ATP12A	ATP4A	9606.ENSP00000218548	9606.ENSP00000262623	0	0	0.8	0.8
ATR	MDM2	9606.ENSP00000343741	9606.ENSP00000258149	0.062	0	0.9	0.902
ATR	EP300	9606.ENSP00000343741	9606.ENSP00000263253	0.071	0.393	0	0.412
ATR	DBF4	9606.ENSP00000343741	9606.ENSP00000265728	0.117	0.236	0.9	0.926
ATR	CDK2	9606.ENSP00000343741	9606.ENSP00000266970	0.063	0.234	0.9	0.922
ATR	CCNA2	9606.ENSP00000343741	9606.ENSP00000274026	0.063	0.14	0.9	0.912
ATR	CCNB2	9606.ENSP00000343741	9606.ENSP00000288207	0.063	0.527	0	0.538
ATR	CDC25A	9606.ENSP00000343741	9606.ENSP00000303706	0.063	0.347	0.65	0.767
ATR	PLEC	9606.ENSP00000343741	9606.ENSP00000323856	0.387	0.139	0	0.449
ATR	HSP90AA1	9606.ENSP00000343741	9606.ENSP00000335153	0	0.449	0	0.449
ATR	PCNA	9606.ENSP00000343741	9606.ENSP00000368458	0.063	0.728	0	0.734
ATR	CDC7	9606.ENSP00000343741	9606.ENSP00000393139	0.082	0.369	0.9	0.937
ATR	RAD52	9606.ENSP00000343741	9606.ENSP00000351284	0.062	0.749	0.6	0.897
ATR	CHEK1	9606.ENSP00000343741	9606.ENSP00000388648	0.088	0.991	0.9	0.999
AURKA	DUT	9606.ENSP00000216911	9606.ENSP00000370376	0.554	0	0	0.554
AURKA	CDK2	9606.ENSP00000216911	9606.ENSP00000266970	0.298	0.326	0	0.507
AURKA	NFKB1	9606.ENSP00000216911	9606.ENSP00000226574	0.063	0.439	0	0.452
AURKA	CCNA1	9606.ENSP00000216911	9606.ENSP00000255465	0.45	0.104	0	0.486
AURKA	EZH2	9606.ENSP00000216911	9606.ENSP00000320147	0.345	0.14	0	0.412
AURKA	CCNE2	9606.ENSP00000216911	9606.ENSP00000429089	0.276	0.317	0	0.485
AURKA	PPP1CC	9606.ENSP00000216911	9606.ENSP00000341779	0.091	0.546	0	0.57
AURKA	EP300	9606.ENSP00000216911	9606.ENSP00000263253	0	0	0.65	0.65
AURKA	CCNE1	9606.ENSP00000216911	9606.ENSP00000262643	0.315	0.237	0	0.455
AURKA	PCNA	9606.ENSP00000216911	9606.ENSP00000368458	0.757	0	0	0.757
AURKA	CDC25A	9606.ENSP00000216911	9606.ENSP00000303706	0.558	0	0	0.558
AURKA	VHL	9606.ENSP00000216911	9606.ENSP00000256474	0	0.486	0	0.485
AURKA	CHEK1	9606.ENSP00000216911	9606.ENSP00000388648	0.754	0.128	0	0.776
AURKA	CCNB3	9606.ENSP00000216911	9606.ENSP00000365210	0.768	0.113	0	0.785
AURKA	FEN1	9606.ENSP00000216911	9606.ENSP00000305480	0.856	0	0	0.856
AURKA	TYMS	9606.ENSP00000216911	9606.ENSP00000315644	0.859	0	0	0.859
AURKA	MELK	9606.ENSP00000216911	9606.ENSP00000298048	0.917	0	0	0.917
AURKA	TTK	9606.ENSP00000216911	9606.ENSP00000358813	0.913	0.108	0	0.919
AURKA	CENPE	9606.ENSP00000216911	9606.ENSP00000265148	0.887	0.146	0	0.9
AURKA	CDC25B	9606.ENSP00000216911	9606.ENSP00000245960	0.143	0.213	0.9	0.926
AURKA	TOP2A	9606.ENSP00000216911	9606.ENSP00000411532	0.918	0.093	0	0.923
AURKA	GSK3B	9606.ENSP00000216911	9606.ENSP00000324806	0	0.689	0.9	0.967

AURKA	CDK1	9606.ENSP00000216911	9606.ENSP00000378699	0.965	0.326	0	0.975
AURKA	CCNA2	9606.ENSP00000216911	9606.ENSP00000274026	0.964	0.104	0	0.967
AURKA	CCNB1	9606.ENSP00000216911	9606.ENSP00000256442	0.926	0.165	0	0.935
AURKA	AURKB	9606.ENSP00000216911	9606.ENSP00000313950	0.925	0.308	0.9	0.994
AURKA	KIF11	9606.ENSP00000216911	9606.ENSP00000260731	0.946	0.417	0	0.967
AURKA	CCNB2	9606.ENSP00000216911	9606.ENSP00000288207	0.972	0.364	0	0.982
AURKA	PLK1	9606.ENSP00000216911	9606.ENSP00000300093	0.946	0.786	0.9	0.998
AURKB	CCNA1	9606.ENSP00000313950	9606.ENSP00000255465	0.45	0.104	0	0.486
AURKB	CCNB1	9606.ENSP00000313950	9606.ENSP00000256442	0.941	0.165	0	0.948
AURKB	KIF11	9606.ENSP00000313950	9606.ENSP00000260731	0.959	0.29	0	0.97
AURKB	CENPE	9606.ENSP00000313950	9606.ENSP00000265148	0.935	0.146	0	0.942
AURKB	CDK2	9606.ENSP00000313950	9606.ENSP00000266970	0.419	0.115	0	0.464
AURKB	CCNA2	9606.ENSP00000313950	9606.ENSP00000274026	0.967	0.104	0	0.969
AURKB	CCNB2	9606.ENSP00000313950	9606.ENSP00000288207	0.962	0.165	0	0.967
AURKB	MELK	9606.ENSP00000313950	9606.ENSP00000298048	0.872	0	0	0.872
AURKB	PLK1	9606.ENSP00000313950	9606.ENSP00000300093	0.941	0.371	0.6	0.984
AURKB	CDC25A	9606.ENSP00000313950	9606.ENSP00000303706	0.586	0.27	0	0.685
AURKB	FEN1	9606.ENSP00000313950	9606.ENSP00000305480	0.869	0	0	0.869
AURKB	EZH2	9606.ENSP00000313950	9606.ENSP00000320147	0.366	0.14	0	0.431
AURKB	PCNA	9606.ENSP00000313950	9606.ENSP00000368458	0.737	0	0	0.737
AURKB	CHEK1	9606.ENSP00000313950	9606.ENSP00000388648	0.72	0.128	0	0.745
AURKB	DUT	9606.ENSP00000313950	9606.ENSP00000370376	0.825	0	0	0.825
AURKB	CCNB3	9606.ENSP00000313950	9606.ENSP00000365210	0.768	0.113	0	0.785
AURKB	HSP90AA1	9606.ENSP00000313950	9606.ENSP00000335153	0.065	0.676	0	0.684
AURKB	TYMS	9606.ENSP00000313950	9606.ENSP00000315644	0.914	0	0	0.914
AURKB	TTK	9606.ENSP00000313950	9606.ENSP00000358813	0.934	0.334	0	0.954
AURKB	PPP1CC	9606.ENSP00000313950	9606.ENSP00000341779	0.086	0.519	0.9	0.952
AURKB	CDK1	9606.ENSP00000313950	9606.ENSP00000378699	0.959	0.326	0	0.971
AURKB	TOP2A	9606.ENSP00000313950	9606.ENSP00000411532	0.947	0.309	0	0.962
AVPR1A	OXTR	9606.ENSP00000299178	9606.ENSP00000324270	0	0	0.5	0.499
AVPR1A	CXCL8	9606.ENSP00000299178	9606.ENSP00000306512	0	0	0.6	0.6
AVPR1A	F2	9606.ENSP00000299178	9606.ENSP00000308541	0.063	0.097	0.6	0.631
AVPR1A	AVPR2	9606.ENSP00000299178	9606.ENSP00000351805	0	0	0.8	0.8
AVPR2	TACR1	9606.ENSP00000351805	9606.ENSP00000303522	0	0.06	0.5	0.509
AVPR2	CXCL8	9606.ENSP00000351805	9606.ENSP00000306512	0	0	0.6	0.6
AVPR2	F2	9606.ENSP00000351805	9606.ENSP00000308541	0	0.097	0.6	0.623
AVPR2	CHRM2	9606.ENSP00000351805	9606.ENSP00000399745	0	0	0.5	0.499
BCL2A1	NFKB1	9606.ENSP00000267953	9606.ENSP00000226574	0.136	0.056	0.9	0.911
BCL2A1	PTPRC	9606.ENSP00000267953	9606.ENSP00000411355	0.425	0	0	0.425
BCL2A1	ITGB2	9606.ENSP00000267953	9606.ENSP00000380948	0.588	0	0	0.588
BCL2A1	MCL1	9606.ENSP00000267953	9606.ENSP00000358022	0.062	0	0.9	0.902
BCL2A1	RELA	9606.ENSP00000267953	9606.ENSP00000384273	0	0	0.9	0.9
BMP1	MMP7	9606.ENSP00000305714	9606.ENSP00000260227	0	0	0.5	0.499
BMP1	MMP13	9606.ENSP00000305714	9606.ENSP00000260302	0	0	0.5	0.499

BMP1	MMP3	9606.ENSP00000305714	9606.ENSP00000299855	0.065	0	0.5	0.512
BMP1	MMP12	9606.ENSP00000305714	9606.ENSP00000458585	0	0	0.5	0.499
BMP1	CTSL	9606.ENSP00000305714	9606.ENSP00000345344	0.069	0	0.5	0.514
BRAF	MAPK1	9606.ENSP00000288602	9606.ENSP00000215832	0.066	0.835	0.9	0.983
BRAF	PIN1	9606.ENSP00000288602	9606.ENSP00000247970	0	0.138	0.9	0.91
BRAF	RAF1	9606.ENSP00000288602	9606.ENSP00000251849	0	0.934	0.9	0.993
BRAF	MAPK3	9606.ENSP00000288602	9606.ENSP00000263025	0.066	0.757	0.9	0.975
BRAF	PRKCQ	9606.ENSP00000288602	9606.ENSP00000263125	0.066	0.117	0.6	0.641
BRAF	PRKCG	9606.ENSP00000288602	9606.ENSP00000263431	0.06	0.061	0.8	0.808
BRAF	JAK2	9606.ENSP00000288602	9606.ENSP00000371067	0.053	0.184	0.3	0.411
BRAF	CAMK2A	9606.ENSP00000288602	9606.ENSP00000381412	0.062	0.057	0.5	0.519
BRAF	HSPA1A	9606.ENSP00000288602	9606.ENSP00000364802	0	0.52	0	0.52
BRAF	SRC	9606.ENSP00000288602	9606.ENSP00000362680	0.062	0.427	0.3	0.591
BRAF	PRKCD	9606.ENSP00000288602	9606.ENSP00000378217	0.066	0.117	0.6	0.641
BRAF	PRKCH	9606.ENSP00000288602	9606.ENSP00000329127	0.066	0.117	0.6	0.641
BRAF	NTRK1	9606.ENSP00000288602	9606.ENSP00000431418	0.062	0.176	0.6	0.663
BRAF	GSK3B	9606.ENSP00000288602	9606.ENSP00000324806	0.062	0	0.8	0.804
BRAF	PRKCE	9606.ENSP00000288602	9606.ENSP00000306124	0.066	0.526	0.6	0.807
BRAF	HSP90AA1	9606.ENSP00000288602	9606.ENSP00000335153	0	0.724	0	0.724
BRAF	MAP3K11	9606.ENSP00000288602	9606.ENSP00000309597	0	0	0.9	0.9
BRAF	PRKCB	9606.ENSP00000288602	9606.ENSP00000305355	0.062	0.061	0.9	0.904
BRAF	PPP1CC	9606.ENSP00000288602	9606.ENSP00000341779	0	0.117	0.9	0.907
BRAF	PRKCA	9606.ENSP00000288602	9606.ENSP00000408695	0	0.285	0.9	0.925
BRAF	YWHAG	9606.ENSP00000288602	9606.ENSP00000306330	0	0.77	0.9	0.976
BRAF	MAP2K1	9606.ENSP00000288602	9606.ENSP00000302486	0.083	0.993	0.9	0.999
BRD2	CSNK2A1	9606.ENSP00000378704	9606.ENSP00000217244	0.065	0.673	0	0.681
BRD2	CCND1	9606.ENSP00000378704	9606.ENSP00000227507	0.062	0	0.6	0.608
BRD2	EP300	9606.ENSP00000378704	9606.ENSP00000263253	0.112	0	0.6	0.629
BRD2	BRD4	9606.ENSP00000378704	9606.ENSP00000263377	0.062	0.479	0	0.49
BRD2	HDAC4	9606.ENSP00000378704	9606.ENSP00000264606	0.062	0.171	0.6	0.661
BRD2	BRD3	9606.ENSP00000378704	9606.ENSP00000305918	0	0.824	0	0.824
BRD3	BRD4	9606.ENSP00000305918	9606.ENSP00000263377	0.105	0.811	0	0.823
BRD4	VHL	9606.ENSP00000263377	9606.ENSP00000256474	0	0.8	0	0.8
BRD4	CCNT1	9606.ENSP00000263377	9606.ENSP00000261900	0.138	0.879	0	0.891
BRD4	EP300	9606.ENSP00000263377	9606.ENSP00000263253	0.243	0.862	0	0.891
BRD4	SMARCA4	9606.ENSP00000263377	9606.ENSP00000395654	0.185	0.4	0	0.491
BRD4	CDK9	9606.ENSP00000263377	9606.ENSP00000362361	0.081	0.893	0	0.897
BRD4	RELA	9606.ENSP00000263377	9606.ENSP00000384273	0.083	0.956	0	0.958
BTK	PIK3CA	9606.ENSP00000483570	9606.ENSP00000263967	0.062	0.104	0.9	0.908
BTK	TXK	9606.ENSP00000483570	9606.ENSP00000264316	0.076	0.398	0.3	0.576
BTK	KIT	9606.ENSP00000483570	9606.ENSP00000288135	0	0.468	0	0.468
BTK	PIK3CB	9606.ENSP00000483570	9606.ENSP00000289153	0.062	0.104	0.9	0.908
BTK	PRKCB	9606.ENSP00000483570	9606.ENSP00000305355	0.195	0.473	0	0.557
BTK	FOS	9606.ENSP00000483570	9606.ENSP00000306245	0	0.064	0.9	0.902

BTK	TLR8	9606.ENSP00000483570	9606.ENSP00000312082	0.349	0.27	0	0.505
BTK	LCK	9606.ENSP00000483570	9606.ENSP00000337825	0.113	0	0.9	0.907
BTK	RAC1	9606.ENSP00000483570	9606.ENSP00000348461	0.093	0.14	0.9	0.915
BTK	JUN	9606.ENSP00000483570	9606.ENSP00000360266	0	0.057	0.9	0.901
BTK	SRC	9606.ENSP00000483570	9606.ENSP00000362680	0.062	0.347	0.9	0.933
BTK	FGR	9606.ENSP00000483570	9606.ENSP00000363117	0.367	0	0.9	0.934
BTK	SYK	9606.ENSP00000483570	9606.ENSP00000364907	0.305	0.462	0.9	0.959
BTK	PIK3CD	9606.ENSP00000483570	9606.ENSP00000366563	0.117	0.104	0.9	0.914
BTK	JAK2	9606.ENSP00000483570	9606.ENSP00000371067	0.1	0	0.9	0.906
BTK	MAPK8	9606.ENSP00000483570	9606.ENSP00000378974	0.062	0.143	0.9	0.912
BTK	ITGB2	9606.ENSP00000483570	9606.ENSP00000380948	0.384	0.07	0	0.403
BTK	PTPN6	9606.ENSP00000483570	9606.ENSP00000391592	0.344	0.078	0.9	0.934
BTK	TBXA2R	9606.ENSP00000483570	9606.ENSP00000393333	0	0.056	0.65	0.655
BTK	ITK	9606.ENSP00000483570	9606.ENSP00000398655	0	0.213	0.9	0.917
BTK	PTPRC	9606.ENSP00000483570	9606.ENSP00000411355	0.44	0.078	0	0.462
BTK	PIK3R1	9606.ENSP00000483570	9606.ENSP00000428056	0	0.102	0.9	0.906
BTK	LYN	9606.ENSP00000483570	9606.ENSP00000428924	0.17	0.462	0.9	0.951
BTK	IKBKB	9606.ENSP00000483570	9606.ENSP00000430684	0	0	0.8	0.8
BTK	HCK	9606.ENSP00000483570	9606.ENSP00000444986	0.36	0.27	0.9	0.949
BTK	YES1	9606.ENSP00000483570	9606.ENSP00000462468	0.066	0.347	0.9	0.933
BTK	IKBKG	9606.ENSP00000483570	9606.ENSP00000483825	0	0.059	0.8	0.803
C5AR1	MPEG1	9606.ENSP00000347197	9606.ENSP00000354335	0.464	0	0	0.464
C5AR1	ITGB2	9606.ENSP00000347197	9606.ENSP00000380948	0.422	0	0	0.422
CA1	CA2	9606.ENSP00000430656	9606.ENSP00000285379	0	0	0.6	0.6
CA1	CA3	9606.ENSP00000430656	9606.ENSP00000285381	0.062	0	0.6	0.608
CA1	CA13	9606.ENSP00000430656	9606.ENSP00000318912	0	0	0.6	0.6
CA1	CA7	9606.ENSP00000430656	9606.ENSP00000345659	0	0	0.6	0.6
CA12	CA14	9606.ENSP00000178638	9606.ENSP00000358107	0.062	0	0.6	0.608
CA12	CA9	9606.ENSP00000178638	9606.ENSP00000367608	0.07	0	0.6	0.612
CA12	CA4	9606.ENSP00000178638	9606.ENSP00000300900	0.062	0	0.6	0.608
CA13	CA2	9606.ENSP00000318912	9606.ENSP00000285379	0	0	0.6	0.6
CA13	CA3	9606.ENSP00000318912	9606.ENSP00000285381	0.062	0	0.6	0.608
CA13	CA7	9606.ENSP00000318912	9606.ENSP00000345659	0.064	0	0.6	0.609
CA14	CA4	9606.ENSP00000358107	9606.ENSP00000300900	0.055	0	0.6	0.605
CA14	CA9	9606.ENSP00000358107	9606.ENSP00000367608	0	0	0.6	0.6
CA2	CA7	9606.ENSP00000285379	9606.ENSP00000345659	0.064	0	0.6	0.609
CA2	CA3	9606.ENSP00000285379	9606.ENSP00000285381	0.089	0	0.6	0.62
CA3	CA7	9606.ENSP00000285381	9606.ENSP00000345659	0.062	0	0.6	0.608
CA4	CA9	9606.ENSP00000300900	9606.ENSP00000367608	0	0	0.6	0.6
CA5A	CA5B	9606.ENSP00000309649	9606.ENSP00000314099	0.109	0	0.6	0.628
CA9	EP300	9606.ENSP00000367608	9606.ENSP00000263253	0	0	0.9	0.9
CA9	EPAS1	9606.ENSP00000367608	9606.ENSP00000263734	0.069	0	0.9	0.902
CA9	HIF1A	9606.ENSP00000367608	9606.ENSP00000437955	0	0.459	0.9	0.943
CACNA1B	GNAO1	9606.ENSP00000360423	9606.ENSP00000262494	0.215	0.289	0	0.418

CACNA1B	RASGRP1	9606.ENSP00000360423	9606.ENSP00000310244	0.062	0	0.6	0.608
CACNA1B	PDE4B	9606.ENSP00000360423	9606.ENSP00000332116	0.079	0	0.54	0.558
CACNA1B	CACNA1H	9606.ENSP00000360423	9606.ENSP00000334198	0.106	0.056	0.72	0.743
CACNA1B	GNAI1	9606.ENSP00000360423	9606.ENSP00000343027	0.089	0.067	0.6	0.63
CACNA1B	PDE4D	9606.ENSP00000360423	9606.ENSP00000345502	0.079	0	0.54	0.558
CACNA1B	CACNA2D1	9606.ENSP00000360423	9606.ENSP00000349320	0.142	0.346	0.72	0.829
CACNA1B	GNAI3	9606.ENSP00000360423	9606.ENSP00000358867	0.062	0.067	0.6	0.619
CACNA1B	SCN5A	9606.ENSP00000360423	9606.ENSP00000410257	0.06	0.402	0	0.413
CACNA1B	FKBP1A	9606.ENSP00000360423	9606.ENSP00000383003	0.062	0	0.72	0.726
CACNA1H	RASGRP1	9606.ENSP00000334198	9606.ENSP00000310244	0.062	0	0.6	0.608
CACNA1H	PDE4B	9606.ENSP00000334198	9606.ENSP00000332116	0.066	0	0.54	0.551
CACNA1H	PDE4D	9606.ENSP00000334198	9606.ENSP00000345502	0.066	0	0.54	0.551
CACNA1H	SCN5A	9606.ENSP00000334198	9606.ENSP00000410257	0.064	0.056	0.54	0.558
CACNA1H	SCN9A	9606.ENSP00000334198	9606.ENSP00000386306	0.064	0.056	0.54	0.558
CACNA1H	FKBP1A	9606.ENSP00000334198	9606.ENSP00000383003	0	0	0.72	0.72
CACNA1H	NOS1	9606.ENSP00000334198	9606.ENSP00000477999	0.062	0.059	0.8	0.808
CACNA1H	ESR2	9606.ENSP00000334198	9606.ENSP00000343925	0	0	0.8	0.8
CACNA1H	CACNA2D1	9606.ENSP00000334198	9606.ENSP00000349320	0.088	0.211	0.72	0.781
CACNA2D1	RASGRP1	9606.ENSP00000349320	9606.ENSP00000310244	0.062	0	0.6	0.608
CACNA2D1	OXTR	9606.ENSP00000349320	9606.ENSP00000324270	0	0	0.6	0.6
CACNA2D1	PDE4B	9606.ENSP00000349320	9606.ENSP00000332116	0.062	0	0.54	0.55
CACNA2D1	PPP1CC	9606.ENSP00000349320	9606.ENSP00000341779	0	0	0.6	0.6
CACNA2D1	PDE4D	9606.ENSP00000349320	9606.ENSP00000345502	0.062	0	0.54	0.55
CACNA2D1	CAMK2A	9606.ENSP00000349320	9606.ENSP00000381412	0.107	0	0.6	0.627
CACNA2D1	FKBP1A	9606.ENSP00000349320	9606.ENSP00000383003	0	0	0.72	0.72
CALCRL	GABBR2	9606.ENSP00000386972	9606.ENSP00000259455	0	0	0.72	0.72
CALCRL	GRM1	9606.ENSP00000386972	9606.ENSP00000354896	0.064	0	0.9	0.902
CALCRL	GABBR1	9606.ENSP00000386972	9606.ENSP00000366233	0	0	0.72	0.72
CAMK2A	HDAC5	9606.ENSP00000381412	9606.ENSP00000225983	0.059	0.27	0.9	0.925
CAMK2A	RAF1	9606.ENSP00000381412	9606.ENSP00000251849	0	0.057	0.5	0.508
CAMK2A	MDM2	9606.ENSP00000381412	9606.ENSP00000258149	0	0	0.9	0.9
CAMK2A	GNAO1	9606.ENSP00000381412	9606.ENSP00000262494	0.224	0.272	0	0.411
CAMK2A	EP300	9606.ENSP00000381412	9606.ENSP00000263253	0.063	0	0.8	0.804
CAMK2A	HDAC4	9606.ENSP00000381412	9606.ENSP00000264606	0.059	0	0.9	0.901
CAMK2A	SLC6A3	9606.ENSP00000381412	9606.ENSP00000270349	0	0.27	0.8	0.847
CAMK2A	PDE3B	9606.ENSP00000381412	9606.ENSP00000282096	0	0	0.6	0.6
CAMK2A	HTR5A	9606.ENSP00000381412	9606.ENSP00000287907	0.508	0.057	0	0.516
CAMK2A	GRIA2	9606.ENSP00000381412	9606.ENSP00000296526	0.371	0.138	0.9	0.941
CAMK2A	NOS3	9606.ENSP00000381412	9606.ENSP00000297494	0.052	0.27	0.8	0.849
CAMK2A	MAP2K1	9606.ENSP00000381412	9606.ENSP00000302486	0.062	0	0.5	0.51
CAMK2A	GRM5	9606.ENSP00000381412	9606.ENSP00000306138	0.372	0.151	0.8	0.884
CAMK2A	CHRM1	9606.ENSP00000381412	9606.ENSP00000306490	0.351	0.148	0	0.423
CAMK2A	PDE4B	9606.ENSP00000381412	9606.ENSP00000332116	0.065	0	0.6	0.61
CAMK2A	MAPT	9606.ENSP00000381412	9606.ENSP00000340820	0.228	0.69	0.9	0.974

CAMK2A	PPP1CC	9606.ENSP00000381412	9606.ENSP00000341779	0	0.07	0.8	0.806
CAMK2A	JAK1	9606.ENSP00000381412	9606.ENSP00000343204	0	0.057	0.9	0.901
CAMK2A	PDE4D	9606.ENSP00000381412	9606.ENSP00000345502	0.065	0	0.6	0.61
CAMK2A	PDE3A	9606.ENSP00000381412	9606.ENSP00000351957	0	0	0.6	0.6
CAMK2A	GRM1	9606.ENSP00000381412	9606.ENSP00000354896	0.316	0.151	0.8	0.873
CAMK2A	GRIN1	9606.ENSP00000381412	9606.ENSP00000360608	0.378	0.631	0.5	0.875
CAMK2A	SRC	9606.ENSP00000381412	9606.ENSP00000362680	0.062	0.057	0.5	0.519
CAMK2A	JAK2	9606.ENSP00000381412	9606.ENSP00000371067	0	0.057	0.9	0.901
CAMK2A	PRKCD	9606.ENSP00000381412	9606.ENSP00000378217	0.064	0	0.5	0.511
CAMK2A	SCN5A	9606.ENSP00000381412	9606.ENSP00000410257	0.062	0.105	0.6	0.634
CAMK2A	PDE10A	9606.ENSP00000381412	9606.ENSP00000438284	0.063	0	0.6	0.609
CAMK2A	TRPV1	9606.ENSP00000381412	9606.ENSP00000459962	0	0	0.8	0.8
CAMK2A	NOS1	9606.ENSP00000381412	9606.ENSP00000477999	0.096	0.213	0.8	0.845
CAMK2A	GRIA1	9606.ENSP00000381412	9606.ENSP00000428994	0.272	0.343	0.9	0.948
CAMK2A	GRIN2B	9606.ENSP00000381412	9606.ENSP00000477455	0.231	0.991	0.5	0.996
CAPN1	PIK3CA	9606.ENSP00000431984	9606.ENSP00000263967	0.062	0	0.9	0.902
CAPN1	CDK5R1	9606.ENSP00000431984	9606.ENSP00000318486	0.057	0.213	0.9	0.919
CAPN1	F2R	9606.ENSP00000431984	9606.ENSP00000321326	0	0.6	0	0.6
CAPN1	PTK2	9606.ENSP00000431984	9606.ENSP00000341189	0.062	0	0.9	0.902
CAPN1	RAC1	9606.ENSP00000431984	9606.ENSP00000348461	0.046	0.058	0.9	0.902
CAPN1	PSEN2	9606.ENSP00000431984	9606.ENSP00000355747	0.062	0.462	0	0.473
CAPN1	PTPN1	9606.ENSP00000431984	9606.ENSP00000360683	0	0.487	0.9	0.946
CAPN1	SRC	9606.ENSP00000431984	9606.ENSP00000362680	0.076	0.057	0.9	0.905
CAPN1	CDK5	9606.ENSP00000431984	9606.ENSP00000419782	0.049	0	0.65	0.652
CAPN1	PIK3R1	9606.ENSP00000431984	9606.ENSP00000428056	0	0	0.9	0.9
CAPN1	GRIN2B	9606.ENSP00000431984	9606.ENSP00000477455	0	0.608	0	0.608
CASP8	TNFRSF1A	9606.ENSP00000351273	9606.ENSP00000162749	0.063	0.476	0.9	0.946
CASP8	MAPK1	9606.ENSP00000351273	9606.ENSP00000215832	0	0.282	0.9	0.925
CASP8	RIPK2	9606.ENSP00000351273	9606.ENSP00000220751	0.062	0.231	0.9	0.921
CASP8	NFKB1	9606.ENSP00000351273	9606.ENSP00000226574	0.062	0.09	0.9	0.907
CASP8	MDM2	9606.ENSP00000351273	9606.ENSP00000258149	0.466	0	0	0.465
CASP8	MAPK3	9606.ENSP00000351273	9606.ENSP00000263025	0	0.282	0.9	0.925
CASP8	PLEC	9606.ENSP00000351273	9606.ENSP00000323856	0	0.284	0.9	0.925
CASP8	CASP9	9606.ENSP00000351273	9606.ENSP00000330237	0.053	0.488	0	0.494
CASP8	RELA	9606.ENSP00000351273	9606.ENSP00000384273	0.062	0	0.9	0.902
CASP8	IKBKB	9606.ENSP00000351273	9606.ENSP00000430684	0.062	0.317	0.9	0.93
CASP8	SRC	9606.ENSP00000351273	9606.ENSP00000362680	0	0.497	0	0.497
CASP8	TNF	9606.ENSP00000351273	9606.ENSP00000398698	0.062	0.282	0.9	0.926
CASP8	IKBKG	9606.ENSP00000351273	9606.ENSP00000483825	0	0.213	0.9	0.917
CASP9	MAPK1	9606.ENSP00000330237	9606.ENSP00000215832	0	0.282	0.9	0.925
CASP9	PARP2	9606.ENSP00000330237	9606.ENSP00000250416	0	0.097	0.8	0.811
CASP9	MAPK3	9606.ENSP00000330237	9606.ENSP00000263025	0	0.226	0.9	0.919
CASP9	NLRP1	9606.ENSP00000330237	9606.ENSP00000478516	0	0.486	0	0.485
CASP9	PDPK1	9606.ENSP00000330237	9606.ENSP00000344220	0	0.078	0.65	0.663

CASP9	PARP1	9606.ENSP00000330237	9606.ENSP00000355759	0	0.097	0.8	0.811
CASR	GABBR2	9606.ENSP00000420194	9606.ENSP00000259455	0	0.359	0.6	0.732
CASR	PRKCB	9606.ENSP00000420194	9606.ENSP00000305355	0.062	0.472	0	0.484
CASR	GRM5	9606.ENSP00000420194	9606.ENSP00000306138	0	0.84	0.5	0.916
CASR	TAS1R1	9606.ENSP00000420194	9606.ENSP00000331867	0	0	0.6	0.6
CASR	GNAI1	9606.ENSP00000420194	9606.ENSP00000343027	0	0	0.8	0.8
CASR	GRM1	9606.ENSP00000420194	9606.ENSP00000354896	0	0.955	0.5	0.976
CASR	GNAI3	9606.ENSP00000420194	9606.ENSP00000358867	0	0	0.8	0.8
CASR	GABBR1	9606.ENSP00000420194	9606.ENSP00000366233	0	0.359	0.6	0.732
CASR	TAS2R31	9606.ENSP00000420194	9606.ENSP00000375093	0	0	0.6	0.6
CASR	GRM2	9606.ENSP00000420194	9606.ENSP00000378492	0	0	0.6	0.6
CASR	HIF1A	9606.ENSP00000420194	9606.ENSP00000437955	0	0.486	0	0.485
CASR	GRM4	9606.ENSP00000420194	9606.ENSP00000440556	0	0	0.6	0.6
CCKBR	PTPN11	9606.ENSP00000335544	9606.ENSP00000340944	0	0.417	0	0.416
CCNA1	PYGL	9606.ENSP00000255465	9606.ENSP00000216392	0.109	0.533	0	0.566
CCNA1	CCND1	9606.ENSP00000255465	9606.ENSP00000227507	0.062	0.098	0.9	0.908
CCNA1	CDC25B	9606.ENSP00000255465	9606.ENSP00000245960	0.084	0.16	0.9	0.916
CCNA1	PTK2	9606.ENSP00000255465	9606.ENSP00000341189	0	0.444	0	0.444
CCNA1	MELK	9606.ENSP00000255465	9606.ENSP00000298048	0.509	0.155	0	0.567
CCNA1	PCNA	9606.ENSP00000255465	9606.ENSP00000368458	0.243	0	0.36	0.494
CCNA1	CENPE	9606.ENSP00000255465	9606.ENSP00000265148	0.644	0.151	0	0.684
CCNA1	TTK	9606.ENSP00000255465	9606.ENSP00000358813	0.653	0.067	0	0.662
CCNA1	PLK1	9606.ENSP00000255465	9606.ENSP00000300093	0.632	0.082	0	0.647
CCNA1	KIF11	9606.ENSP00000255465	9606.ENSP00000260731	0.755	0.139	0	0.78
CCNA1	CCNB3	9606.ENSP00000255465	9606.ENSP00000365210	0.385	0.181	0.54	0.748
CCNA1	CCNB2	9606.ENSP00000255465	9606.ENSP00000288207	0.518	0.181	0.54	0.802
CCNA1	CDK9	9606.ENSP00000255465	9606.ENSP00000362361	0.062	0	0.9	0.902
CCNA1	CCNT1	9606.ENSP00000255465	9606.ENSP00000261900	0.064	0.077	0.9	0.906
CCNA1	TERT	9606.ENSP00000255465	9606.ENSP00000309572	0.123	0.061	0.9	0.91
CCNA1	CCNA2	9606.ENSP00000255465	9606.ENSP00000274026	0.066	0.288	0.9	0.927
CCNA1	MDM2	9606.ENSP00000255465	9606.ENSP00000258149	0.062	0.131	0.9	0.911
CCNA1	CDK7	9606.ENSP00000255465	9606.ENSP00000256443	0.055	0.136	0.9	0.911
CCNA1	CCNH	9606.ENSP00000255465	9606.ENSP00000256897	0.067	0.077	0.9	0.906
CCNA1	CCNE2	9606.ENSP00000255465	9606.ENSP00000429089	0.192	0.245	0.9	0.933
CCNA1	CCNE1	9606.ENSP00000255465	9606.ENSP00000262643	0.192	0.245	0.9	0.933
CCNA1	WEE1	9606.ENSP00000255465	9606.ENSP00000402084	0.167	0.216	0.9	0.928
CCNA1	CCNB1	9606.ENSP00000255465	9606.ENSP00000256442	0.551	0.181	0.9	0.96
CCNA1	CDK4	9606.ENSP00000255465	9606.ENSP00000257904	0.11	0.23	0.9	0.925
CCNA1	CDC25A	9606.ENSP00000255465	9606.ENSP00000303706	0.084	0.392	0.9	0.939
CCNA1	CDK2	9606.ENSP00000255465	9606.ENSP00000266970	0.144	0.932	0.9	0.993
CCNA1	CDK1	9606.ENSP00000255465	9606.ENSP00000378699	0.764	0.867	0.9	0.996
CCNA2	PYGL	9606.ENSP00000274026	9606.ENSP00000216392	0.109	0.533	0	0.566
CCNA2	CCND1	9606.ENSP00000274026	9606.ENSP00000227507	0.085	0.098	0.72	0.748
CCNA2	CDC25B	9606.ENSP00000274026	9606.ENSP00000245960	0.121	0.16	0.9	0.919

CCNA2	CCNB1	9606.ENSP00000274026	9606.ENSP00000256442	0.969	0.54	0.72	0.995
CCNA2	CDK7	9606.ENSP00000274026	9606.ENSP00000256443	0.081	0.711	0.9	0.971
CCNA2	CCNH	9606.ENSP00000274026	9606.ENSP00000256897	0.067	0.077	0.9	0.906
CCNA2	CDK4	9606.ENSP00000274026	9606.ENSP00000257904	0.183	0.587	0.9	0.963
CCNA2	MDM2	9606.ENSP00000274026	9606.ENSP00000258149	0.062	0.131	0.9	0.911
CCNA2	KIF11	9606.ENSP00000274026	9606.ENSP00000260731	0.976	0.139	0	0.978
CCNA2	CCNT1	9606.ENSP00000274026	9606.ENSP00000261900	0.081	0.077	0.9	0.907
CCNA2	CCNE1	9606.ENSP00000274026	9606.ENSP00000262643	0.393	0.245	0.9	0.95
CCNA2	EP300	9606.ENSP00000274026	9606.ENSP00000263253	0.063	0.699	0	0.706
CCNA2	CENPE	9606.ENSP00000274026	9606.ENSP00000265148	0.963	0.151	0	0.967
CCNA2	DBF4	9606.ENSP00000274026	9606.ENSP00000265728	0.367	0.102	0	0.407
CCNA2	CDK2	9606.ENSP00000274026	9606.ENSP00000266970	0.401	0.999	0.9	0.999
CCNA2	DUT	9606.ENSP00000274026	9606.ENSP00000370376	0.536	0	0	0.536
CCNA2	POLA1	9606.ENSP00000274026	9606.ENSP00000368349	0.286	0.339	0	0.508
CCNA2	DHFR	9606.ENSP00000274026	9606.ENSP00000396308	0.431	0	0	0.431
CCNA2	EZH2	9606.ENSP00000274026	9606.ENSP00000320147	0.606	0.064	0	0.616
CCNA2	CDC7	9606.ENSP00000274026	9606.ENSP00000393139	0.572	0.078	0	0.589
CCNA2	CCNB3	9606.ENSP00000274026	9606.ENSP00000365210	0.521	0.181	0.54	0.803
CCNA2	TYMS	9606.ENSP00000274026	9606.ENSP00000315644	0.837	0	0	0.837
CCNA2	FEN1	9606.ENSP00000274026	9606.ENSP00000305480	0.817	0.379	0	0.881
CCNA2	TERT	9606.ENSP00000274026	9606.ENSP00000309572	0.2	0.061	0.9	0.918
CCNA2	CHEK1	9606.ENSP00000274026	9606.ENSP00000388648	0.852	0.058	0	0.854
CCNA2	CDK9	9606.ENSP00000274026	9606.ENSP00000362361	0.062	0	0.9	0.902
CCNA2	MELK	9606.ENSP00000274026	9606.ENSP00000298048	0.93	0.155	0	0.939
CCNA2	HDAC1	9606.ENSP00000274026	9606.ENSP00000362649	0.112	0.298	0.9	0.932
CCNA2	CCNE2	9606.ENSP00000274026	9606.ENSP00000429089	0.426	0.245	0.9	0.952
CCNA2	PCNA	9606.ENSP00000274026	9606.ENSP00000368458	0.678	0.803	0.36	0.955
CCNA2	WEE1	9606.ENSP00000274026	9606.ENSP00000402084	0.213	0.216	0.9	0.932
CCNA2	TOP2A	9606.ENSP00000274026	9606.ENSP00000411532	0.967	0.056	0	0.967
CCNA2	TTK	9606.ENSP00000274026	9606.ENSP00000358813	0.979	0.067	0	0.979
CCNA2	CCNB2	9606.ENSP00000274026	9606.ENSP00000288207	0.968	0.181	0.54	0.987
CCNA2	PLK1	9606.ENSP00000274026	9606.ENSP00000300093	0.976	0.082	0	0.977
CCNA2	CDC25A	9606.ENSP00000274026	9606.ENSP00000303706	0.579	0.196	0.9	0.963
CCNA2	CDK1	9606.ENSP00000274026	9606.ENSP00000378699	0.985	0.929	0.9	0.999
CCNB1	CCND1	9606.ENSP00000256442	9606.ENSP00000227507	0.097	0.078	0.9	0.909
CCNB1	CDC25B	9606.ENSP00000256442	9606.ENSP00000245960	0.47	0.196	0.9	0.953
CCNB1	PIN1	9606.ENSP00000256442	9606.ENSP00000247970	0.048	0.229	0.9	0.92
CCNB1	DBF4	9606.ENSP00000256442	9606.ENSP00000265728	0.346	0.125	0	0.403
CCNB1	DNMT1	9606.ENSP00000256442	9606.ENSP00000352516	0.218	0.27	0	0.404
CCNB1	PRKDC	9606.ENSP00000256442	9606.ENSP00000313420	0.139	0.345	0	0.412
CCNB1	EZH2	9606.ENSP00000256442	9606.ENSP00000320147	0.443	0.065	0	0.458
CCNB1	POLA1	9606.ENSP00000256442	9606.ENSP00000368349	0.304	0.339	0	0.52
CCNB1	DUT	9606.ENSP00000256442	9606.ENSP00000370376	0.692	0	0	0.692
CCNB1	YWHAG	9606.ENSP00000256442	9606.ENSP00000306330	0.051	0.15	0.65	0.693

CCNB1	CDC7	9606.ENSP00000256442	9606.ENSP00000393139	0.417	0.102	0	0.454
CCNB1	FEN1	9606.ENSP00000256442	9606.ENSP00000305480	0.741	0.141	0	0.768
CCNB1	CDK9	9606.ENSP00000256442	9606.ENSP00000362361	0.066	0	0.72	0.727
CCNB1	CCNT1	9606.ENSP00000256442	9606.ENSP00000261900	0.096	0.077	0.72	0.746
CCNB1	TYMS	9606.ENSP00000256442	9606.ENSP00000315644	0.725	0	0	0.725
CCNB1	CCNE2	9606.ENSP00000256442	9606.ENSP00000429089	0.357	0	0.72	0.812
CCNB1	SHH	9606.ENSP00000256442	9606.ENSP00000297261	0	0.07	0.9	0.903
CCNB1	CDK8	9606.ENSP00000256442	9606.ENSP00000370938	0.062	0.136	0.9	0.911
CCNB1	CENPE	9606.ENSP00000256442	9606.ENSP00000265148	0.829	0.139	0	0.846
CCNB1	CCNE1	9606.ENSP00000256442	9606.ENSP00000262643	0.359	0	0.9	0.933
CCNB1	TTK	9606.ENSP00000256442	9606.ENSP00000358813	0.875	0.103	0	0.883
CCNB1	CHEK1	9606.ENSP00000256442	9606.ENSP00000388648	0.74	0.06	0	0.745
CCNB1	MELK	9606.ENSP00000256442	9606.ENSP00000298048	0.921	0.183	0	0.933
CCNB1	CCNH	9606.ENSP00000256442	9606.ENSP00000256897	0.068	0.108	0.9	0.909
CCNB1	PCNA	9606.ENSP00000256442	9606.ENSP00000368458	0.806	0.685	0.36	0.957
CCNB1	CCNB3	9606.ENSP00000256442	9606.ENSP00000365210	0.812	0.367	0.8	0.974
CCNB1	CDK5	9606.ENSP00000256442	9606.ENSP00000419782	0	0.683	0.9	0.967
CCNB1	CDK7	9606.ENSP00000256442	9606.ENSP00000256443	0.116	0.512	0.9	0.953
CCNB1	KIF11	9606.ENSP00000256442	9606.ENSP00000260731	0.935	0.248	0	0.949
CCNB1	XPO1	9606.ENSP00000256442	9606.ENSP00000384863	0.128	0.112	0.9	0.915
CCNB1	TOP2A	9606.ENSP00000256442	9606.ENSP00000411532	0.964	0.056	0	0.964
CCNB1	WEE1	9606.ENSP00000256442	9606.ENSP00000402084	0.213	0.216	0.9	0.932
CCNB1	CDK4	9606.ENSP00000256442	9606.ENSP00000257904	0.213	0.23	0.9	0.934
CCNB1	CCNB2	9606.ENSP00000256442	9606.ENSP00000288207	0.955	0.754	0.9	0.998
CCNB1	PLK1	9606.ENSP00000256442	9606.ENSP00000300093	0.936	0.719	0.8	0.996
CCNB1	CDK2	9606.ENSP00000256442	9606.ENSP00000266970	0.421	0.982	0.9	0.998
CCNB1	CDC25A	9606.ENSP00000256442	9606.ENSP00000303706	0.729	0.715	0.9	0.991
CCNB1	CDK1	9606.ENSP00000256442	9606.ENSP00000378699	0.968	0.999	0.9	0.999
CCNB2	CCND1	9606.ENSP00000288207	9606.ENSP00000227507	0.096	0.078	0.54	0.583
CCNB2	CDC25B	9606.ENSP00000288207	9606.ENSP00000245960	0.471	0.16	0.9	0.951
CCNB2	CDK7	9606.ENSP00000288207	9606.ENSP00000256443	0.097	0.344	0.9	0.935
CCNB2	CCNH	9606.ENSP00000288207	9606.ENSP00000256897	0.065	0.108	0.54	0.582
CCNB2	CDK4	9606.ENSP00000288207	9606.ENSP00000257904	0.199	0.23	0.9	0.932
CCNB2	KIF11	9606.ENSP00000288207	9606.ENSP00000260731	0.964	0.248	0	0.972
CCNB2	CCNT1	9606.ENSP00000288207	9606.ENSP00000261900	0.062	0.077	0.54	0.566
CCNB2	CCNE1	9606.ENSP00000288207	9606.ENSP00000262643	0.364	0	0.54	0.695
CCNB2	CENPE	9606.ENSP00000288207	9606.ENSP00000265148	0.871	0.139	0	0.884
CCNB2	CDK2	9606.ENSP00000288207	9606.ENSP00000266970	0.448	0.917	0.9	0.995
CCNB2	CDK9	9606.ENSP00000288207	9606.ENSP00000362361	0.064	0	0.54	0.551
CCNB2	DUT	9606.ENSP00000288207	9606.ENSP00000370376	0.694	0	0	0.694
CCNB2	CDC7	9606.ENSP00000288207	9606.ENSP00000393139	0.367	0.102	0	0.408
CCNB2	EZH2	9606.ENSP00000288207	9606.ENSP00000320147	0.707	0.064	0	0.714
CCNB2	CCNE2	9606.ENSP00000288207	9606.ENSP00000429089	0.345	0.27	0.54	0.76
CCNB2	FEN1	9606.ENSP00000288207	9606.ENSP00000305480	0.766	0.141	0	0.79

CCNB2	PCNA	9606.ENSP00000288207	9606.ENSP00000368458	0.729	0	0.36	0.819
CCNB2	TYMS	9606.ENSP00000288207	9606.ENSP00000315644	0.833	0	0	0.833
CCNB2	CDK8	9606.ENSP00000288207	9606.ENSP00000370938	0.062	0.136	0.9	0.911
CCNB2	CDK5	9606.ENSP00000288207	9606.ENSP00000419782	0	0.064	0.9	0.902
CCNB2	CHEK1	9606.ENSP00000288207	9606.ENSP00000388648	0.815	0.06	0	0.819
CCNB2	MELK	9606.ENSP00000288207	9606.ENSP00000298048	0.918	0.183	0	0.93
CCNB2	TTK	9606.ENSP00000288207	9606.ENSP00000358813	0.935	0.103	0	0.94
CCNB2	CCNB3	9606.ENSP00000288207	9606.ENSP00000365210	0.812	0.367	0.8	0.974
CCNB2	CDC25A	9606.ENSP00000288207	9606.ENSP00000303706	0.729	0.16	0.8	0.95
CCNB2	TOP2A	9606.ENSP00000288207	9606.ENSP00000411532	0.969	0.056	0	0.969
CCNB2	PLK1	9606.ENSP00000288207	9606.ENSP00000300093	0.937	0.15	0.8	0.988
CCNB2	CDK1	9606.ENSP00000288207	9606.ENSP00000378699	0.993	0.971	0.9	0.999
CCNB3	CCND1	9606.ENSP00000365210	9606.ENSP00000227507	0.062	0.078	0.54	0.567
CCNB3	CDK7	9606.ENSP00000365210	9606.ENSP00000256443	0.062	0.136	0.54	0.594
CCNB3	CCNH	9606.ENSP00000365210	9606.ENSP00000256897	0.062	0.082	0.54	0.569
CCNB3	CDK4	9606.ENSP00000365210	9606.ENSP00000257904	0.11	0.23	0.54	0.657
CCNB3	KIF11	9606.ENSP00000365210	9606.ENSP00000260731	0.862	0.18	0	0.882
CCNB3	CCNT1	9606.ENSP00000365210	9606.ENSP00000261900	0.062	0.077	0.54	0.566
CCNB3	CCNE1	9606.ENSP00000365210	9606.ENSP00000262643	0.185	0	0.54	0.609
CCNB3	CENPE	9606.ENSP00000365210	9606.ENSP00000265148	0.366	0.139	0	0.43
CCNB3	CDK2	9606.ENSP00000365210	9606.ENSP00000266970	0.264	0.502	0.54	0.817
CCNB3	PLK1	9606.ENSP00000365210	9606.ENSP00000300093	0.917	0.082	0.8	0.983
CCNB3	CDC25A	9606.ENSP00000365210	9606.ENSP00000303706	0.145	0.16	0.8	0.843
CCNB3	TTK	9606.ENSP00000365210	9606.ENSP00000358813	0.518	0.077	0	0.536
CCNB3	CDK9	9606.ENSP00000365210	9606.ENSP00000362361	0.064	0	0.54	0.551
CCNB3	DUT	9606.ENSP00000365210	9606.ENSP00000370376	0.446	0	0	0.446
CCNB3	CCNE2	9606.ENSP00000365210	9606.ENSP00000429089	0.185	0	0.54	0.609
CCNB3	PCNA	9606.ENSP00000365210	9606.ENSP00000368458	0.882	0	0.36	0.921
CCNB3	CDK1	9606.ENSP00000365210	9606.ENSP00000378699	0.917	0.789	0.8	0.996
CCNC	FABP4	9606.ENSP00000428982	9606.ENSP00000256104	0	0	0.9	0.9
CCNC	CCNT1	9606.ENSP00000428982	9606.ENSP00000261900	0.068	0.056	0.6	0.617
CCNC	EP300	9606.ENSP00000428982	9606.ENSP00000263253	0.062	0.102	0.6	0.633
CCNC	KAT2B	9606.ENSP00000428982	9606.ENSP00000263754	0.084	0.113	0.6	0.646
CCNC	PPARG	9606.ENSP00000428982	9606.ENSP00000287820	0	0.104	0.6	0.626
CCNC	PLIN1	9606.ENSP00000428982	9606.ENSP00000300055	0	0	0.9	0.9
CCNC	LPL	9606.ENSP00000428982	9606.ENSP00000309757	0	0	0.9	0.9
CCNC	CDK9	9606.ENSP00000428982	9606.ENSP00000362361	0.062	0.26	0.6	0.698
CCNC	CDK8	9606.ENSP00000428982	9606.ENSP00000370938	0.164	0.995	0.8	0.999
CCNC	RXRA	9606.ENSP00000428982	9606.ENSP00000419692	0	0.17	0.6	0.653
CCND1	CRYAB	9606.ENSP00000227507	9606.ENSP00000433560	0	0.502	0	0.502
CCND1	HDAC2	9606.ENSP00000227507	9606.ENSP00000430432	0.062	0.421	0	0.434
CCND1	SMARCA2	9606.ENSP00000227507	9606.ENSP00000265773	0.058	0.057	0.65	0.661
CCND1	PBRM1	9606.ENSP00000227507	9606.ENSP00000378307	0.062	0	0.65	0.657
CCND1	KAT2B	9606.ENSP00000227507	9606.ENSP00000263754	0	0.486	0	0.485

CCND1	DNMT3A	9606.ENSP00000227507	9606.ENSP00000264709	0	0	0.65	0.65
CCND1	CCNT1	9606.ENSP00000227507	9606.ENSP00000261900	0	0.077	0.72	0.73
CCND1	CCNE2	9606.ENSP00000227507	9606.ENSP00000429089	0.063	0.067	0.72	0.733
CCND1	SMARCA4	9606.ENSP00000227507	9606.ENSP00000395654	0.062	0.057	0.65	0.663
CCND1	PGR	9606.ENSP00000227507	9606.ENSP00000325120	0	0.501	0	0.501
CCND1	CDK9	9606.ENSP00000227507	9606.ENSP00000362361	0	0	0.72	0.72
CCND1	RORC	9606.ENSP00000227507	9606.ENSP00000327025	0	0	0.9	0.9
CCND1	HDAC3	9606.ENSP00000227507	9606.ENSP00000302967	0.062	0.76	0	0.765
CCND1	PSMB8	9606.ENSP00000227507	9606.ENSP00000364016	0	0	0.9	0.9
CCND1	HDAC4	9606.ENSP00000227507	9606.ENSP00000264606	0.062	0.056	0.8	0.807
CCND1	RORA	9606.ENSP00000227507	9606.ENSP00000261523	0	0	0.9	0.9
CCND1	PSMB5	9606.ENSP00000227507	9606.ENSP00000355325	0.067	0	0.9	0.902
CCND1	PIM1	9606.ENSP00000227507	9606.ENSP00000362608	0	0	0.9	0.9
CCND1	LYN	9606.ENSP00000227507	9606.ENSP00000428924	0	0	0.9	0.9
CCND1	CCNE1	9606.ENSP00000227507	9606.ENSP00000262643	0.063	0.067	0.9	0.904
CCND1	CDK7	9606.ENSP00000227507	9606.ENSP00000256443	0.062	0.103	0.9	0.908
CCND1	CCNH	9606.ENSP00000227507	9606.ENSP00000256897	0.062	0.147	0.9	0.913
CCND1	CDK5	9606.ENSP00000227507	9606.ENSP00000419782	0.052	0.401	0	0.408
CCND1	JAK2	9606.ENSP00000227507	9606.ENSP00000371067	0	0	0.9	0.9
CCND1	HIF1A	9606.ENSP00000227507	9606.ENSP00000437955	0	0	0.9	0.9
CCND1	CDC25A	9606.ENSP00000227507	9606.ENSP00000303706	0.086	0	0.9	0.904
CCND1	RELA	9606.ENSP00000227507	9606.ENSP00000384273	0	0.486	0.9	0.946
CCND1	JUN	9606.ENSP00000227507	9606.ENSP00000360266	0	0.27	0.9	0.923
CCND1	SRC	9606.ENSP00000227507	9606.ENSP00000362680	0.068	0.27	0.9	0.926
CCND1	PCNA	9606.ENSP00000227507	9606.ENSP00000368458	0.064	0.783	0.9	0.977
CCND1	XPO1	9606.ENSP00000227507	9606.ENSP00000384863	0.052	0.409	0	0.415
CCND1	FOS	9606.ENSP00000227507	9606.ENSP00000306245	0	0.494	0.9	0.947
CCND1	HDAC1	9606.ENSP00000227507	9606.ENSP00000362649	0.062	0.619	0.9	0.961
CCND1	CDK1	9606.ENSP00000227507	9606.ENSP00000378699	0.096	0.804	0.72	0.946
CCND1	GSK3B	9606.ENSP00000227507	9606.ENSP00000324806	0	0.6	0.9	0.958
CCND1	EP300	9606.ENSP00000227507	9606.ENSP00000263253	0.062	0.835	0.9	0.983
CCND1	STAT3	9606.ENSP00000227507	9606.ENSP00000264657	0.049	0.708	0.9	0.969
CCND1	CDK2	9606.ENSP00000227507	9606.ENSP00000266970	0.087	0.898	0.8	0.979
CCND1	CDK4	9606.ENSP00000227507	9606.ENSP00000257904	0.08	0.998	0.9	0.999
CCND1	ESR1	9606.ENSP00000227507	9606.ENSP00000405330	0	0.867	0.9	0.986
CCNE1	CDK7	9606.ENSP00000262643	9606.ENSP00000256443	0.082	0.342	0.9	0.934
CCNE1	CCNH	9606.ENSP00000262643	9606.ENSP00000256897	0.082	0.077	0.9	0.907
CCNE1	CDK4	9606.ENSP00000262643	9606.ENSP00000257904	0.142	0.551	0.9	0.958
CCNE1	CCNT1	9606.ENSP00000262643	9606.ENSP00000261900	0.062	0.077	0.9	0.905
CCNE1	GBA	9606.ENSP00000262643	9606.ENSP00000314508	0	0	0.5	0.499
CCNE1	SMARCA2	9606.ENSP00000262643	9606.ENSP00000265773	0.062	0.497	0	0.508
CCNE1	HDAC3	9606.ENSP00000262643	9606.ENSP00000302967	0.088	0.138	0.5	0.572
CCNE1	STAT3	9606.ENSP00000262643	9606.ENSP00000264657	0	0	0.5	0.499
CCNE1	SMARCA4	9606.ENSP00000262643	9606.ENSP00000395654	0.062	0.646	0	0.654

CCNE1	GSK3B	9606.ENSP00000262643	9606.ENSP00000324806	0	0.695	0	0.695
CCNE1	PTGDR	9606.ENSP00000262643	9606.ENSP00000303424	0	0	0.9	0.9
CCNE1	CCNE2	9606.ENSP00000262643	9606.ENSP00000429089	0.124	0	0.9	0.908
CCNE1	HDAC1	9606.ENSP00000262643	9606.ENSP00000362649	0.089	0.182	0.9	0.919
CCNE1	CDK9	9606.ENSP00000262643	9606.ENSP00000362361	0.064	0	0.9	0.902
CCNE1	CDK5	9606.ENSP00000262643	9606.ENSP00000419782	0	0.689	0	0.689
CCNE1	WEE1	9606.ENSP00000262643	9606.ENSP00000402084	0.146	0.216	0.9	0.927
CCNE1	PCNA	9606.ENSP00000262643	9606.ENSP00000368458	0.267	0.814	0.9	0.985
CCNE1	CDC25A	9606.ENSP00000262643	9606.ENSP00000303706	0.418	0.593	0.9	0.974
CCNE1	CDK1	9606.ENSP00000262643	9606.ENSP00000378699	0.306	0.92	0.9	0.993
CCNE1	CDK2	9606.ENSP00000262643	9606.ENSP00000266970	0.161	0.999	0.9	0.999
CCNE2	CDK7	9606.ENSP00000429089	9606.ENSP00000256443	0.063	0.136	0.9	0.911
CCNE2	CCNH	9606.ENSP00000429089	9606.ENSP00000256897	0.082	0.077	0.9	0.907
CCNE2	CDK4	9606.ENSP00000429089	9606.ENSP00000257904	0.11	0.23	0.9	0.925
CCNE2	KIF11	9606.ENSP00000429089	9606.ENSP00000260731	0.585	0.139	0	0.627
CCNE2	CCNT1	9606.ENSP00000429089	9606.ENSP00000261900	0.062	0.077	0.9	0.905
CCNE2	STAT3	9606.ENSP00000429089	9606.ENSP00000264657	0	0	0.5	0.499
CCNE2	CDK2	9606.ENSP00000429089	9606.ENSP00000266970	0.199	0.968	0.9	0.997
CCNE2	MELK	9606.ENSP00000429089	9606.ENSP00000298048	0.359	0.155	0	0.435
CCNE2	HDAC3	9606.ENSP00000429089	9606.ENSP00000302967	0.065	0.138	0.5	0.561
CCNE2	CDC25A	9606.ENSP00000429089	9606.ENSP00000303706	0.305	0.241	0.9	0.942
CCNE2	FEN1	9606.ENSP00000429089	9606.ENSP00000305480	0.352	0.141	0	0.42
CCNE2	GBA	9606.ENSP00000429089	9606.ENSP00000314508	0	0	0.5	0.499
CCNE2	CDK9	9606.ENSP00000429089	9606.ENSP00000362361	0.064	0	0.9	0.902
CCNE2	PCNA	9606.ENSP00000429089	9606.ENSP00000368458	0.313	0.877	0.36	0.941
CCNE2	CDK1	9606.ENSP00000429089	9606.ENSP00000378699	0.599	0.534	0.9	0.979
CCNE2	WEE1	9606.ENSP00000429089	9606.ENSP00000402084	0.146	0.216	0.9	0.927
CCNH	PARP2	9606.ENSP00000256897	9606.ENSP00000250416	0.11	0	0.6	0.628
CCNH	RARA	9606.ENSP00000256897	9606.ENSP00000254066	0	0.317	0.9	0.928
CCNH	CDK7	9606.ENSP00000256897	9606.ENSP00000256443	0.195	0.999	0.9	0.999
CCNH	PARP1	9606.ENSP00000256897	9606.ENSP00000355759	0.11	0	0.6	0.628
CCNH	EP300	9606.ENSP00000256897	9606.ENSP00000263253	0.062	0.102	0.6	0.633
CCNH	CDK8	9606.ENSP00000256897	9606.ENSP00000370938	0.063	0.499	0	0.511
CCNH	RXRA	9606.ENSP00000256897	9606.ENSP00000419692	0.046	0.103	0.9	0.906
CCNH	RARG	9606.ENSP00000256897	9606.ENSP00000388510	0	0.103	0.9	0.906
CCNH	ERCC5	9606.ENSP00000256897	9606.ENSP00000347978	0.095	0.733	0.6	0.894
CCNH	CDK4	9606.ENSP00000256897	9606.ENSP00000257904	0.063	0.181	0.9	0.916
CCNH	CCNT1	9606.ENSP00000256897	9606.ENSP00000261900	0.085	0	0.9	0.904
CCNH	CDK1	9606.ENSP00000256897	9606.ENSP00000378699	0.066	0.181	0.9	0.916
CCNH	CDK9	9606.ENSP00000256897	9606.ENSP00000362361	0.078	0.436	0.9	0.943
CCNH	CDK2	9606.ENSP00000256897	9606.ENSP00000266970	0.066	0.889	0.9	0.988
CCNT1	CDK7	9606.ENSP00000261900	9606.ENSP00000256443	0.083	0.344	0.9	0.934
CCNT1	CDK4	9606.ENSP00000261900	9606.ENSP00000257904	0.052	0.191	0.9	0.916
CCNT1	CDK8	9606.ENSP00000261900	9606.ENSP00000370938	0.066	0.499	0.6	0.796

CCNT1	ESR1	9606.ENSP00000261900	9606.ENSP00000405330	0	0.34	0.6	0.724
CCNT1	CDK1	9606.ENSP00000261900	9606.ENSP00000378699	0.083	0.191	0.9	0.919
CCNT1	CDK2	9606.ENSP00000261900	9606.ENSP00000266970	0.083	0.191	0.9	0.919
CCNT1	CDK9	9606.ENSP00000261900	9606.ENSP00000362361	0.139	0.999	0.9	0.999
CCR1	STAT3	9606.ENSP00000296140	9606.ENSP00000264657	0	0.225	0.65	0.717
CCR1	CCR2	9606.ENSP00000296140	9606.ENSP00000292301	0.334	0	0.3	0.513
CCR1	CCR5	9606.ENSP00000296140	9606.ENSP00000292303	0.335	0	0.8	0.861
CCR1	MPEG1	9606.ENSP00000296140	9606.ENSP00000354335	0.406	0	0	0.406
CCR1	HCK	9606.ENSP00000296140	9606.ENSP00000444986	0.417	0.056	0	0.426
CCR1	ITGB2	9606.ENSP00000296140	9606.ENSP00000380948	0.431	0	0	0.431
CCR1	GNAI3	9606.ENSP00000296140	9606.ENSP00000358867	0	0.151	0.6	0.645
CCR1	GNAI1	9606.ENSP00000296140	9606.ENSP00000343027	0	0.151	0.6	0.645
CCR1	JAK2	9606.ENSP00000296140	9606.ENSP00000371067	0.07	0.056	0.6	0.618
CCR1	JAK3	9606.ENSP00000296140	9606.ENSP00000391676	0.087	0.056	0.6	0.625
CCR1	CXCL8	9606.ENSP00000296140	9606.ENSP00000306512	0.118	0	0.6	0.632
CCR1	CXCL12	9606.ENSP00000296140	9606.ENSP00000379140	0	0.076	0.6	0.614
CCR1	PTAFR	9606.ENSP00000296140	9606.ENSP00000362965	0.277	0	0.9	0.924
CCR2	STAT3	9606.ENSP00000292301	9606.ENSP00000264657	0	0.056	0.65	0.655
CCR2	GNAI3	9606.ENSP00000292301	9606.ENSP00000358867	0	0.151	0.6	0.645
CCR2	GNAI1	9606.ENSP00000292301	9606.ENSP00000343027	0	0.151	0.6	0.645
CCR2	JAK3	9606.ENSP00000292301	9606.ENSP00000391676	0.107	0.056	0.6	0.633
CCR2	CCR5	9606.ENSP00000292301	9606.ENSP00000292303	0.328	0.213	0.6	0.769
CCR2	JAK2	9606.ENSP00000292301	9606.ENSP00000371067	0.065	0.225	0.6	0.684
CCR2	CXCL8	9606.ENSP00000292301	9606.ENSP00000306512	0.08	0	0.6	0.616
CCR2	CXCL12	9606.ENSP00000292301	9606.ENSP00000379140	0	0.076	0.6	0.614
CCR2	PTAFR	9606.ENSP00000292301	9606.ENSP00000362965	0.231	0	0.9	0.919
CCR5	STAT3	9606.ENSP00000292303	9606.ENSP00000264657	0	0.47	0.65	0.806
CCR5	HTR2C	9606.ENSP00000292303	9606.ENSP00000276198	0	0	0.9	0.9
CCR5	CSF1R	9606.ENSP00000292303	9606.ENSP00000286301	0.568	0	0	0.568
CCR5	MPEG1	9606.ENSP00000292303	9606.ENSP00000354335	0.434	0	0	0.434
CCR5	TLR8	9606.ENSP00000292303	9606.ENSP00000312082	0.426	0	0	0.426
CCR5	ITGB2	9606.ENSP00000292303	9606.ENSP00000380948	0.534	0	0	0.534
CCR5	JAK3	9606.ENSP00000292303	9606.ENSP00000391676	0.116	0.056	0.6	0.637
CCR5	JAK2	9606.ENSP00000292303	9606.ENSP00000371067	0.065	0.47	0.6	0.784
CCR5	GNAI3	9606.ENSP00000292303	9606.ENSP00000358867	0	0.303	0.8	0.854
CCR5	GNAI1	9606.ENSP00000292303	9606.ENSP00000343027	0	0.303	0.8	0.854
CCR5	CHRM1	9606.ENSP00000292303	9606.ENSP00000306490	0	0	0.9	0.9
CCR5	PTGER1	9606.ENSP00000292303	9606.ENSP00000292513	0	0	0.9	0.9
CCR5	F2	9606.ENSP00000292303	9606.ENSP00000308541	0	0.097	0.9	0.905
CCR5	CXCL8	9606.ENSP00000292303	9606.ENSP00000306512	0.086	0	0.6	0.618
CCR5	PTAFR	9606.ENSP00000292303	9606.ENSP00000362965	0.278	0	0.9	0.924
CCR5	JUN	9606.ENSP00000292303	9606.ENSP00000360266	0	0	0.9	0.9
CCR5	CXCL12	9606.ENSP00000292303	9606.ENSP00000379140	0	0.076	0.9	0.903
CCR9	CXCL8	9606.ENSP00000350256	9606.ENSP00000306512	0	0	0.6	0.6

CCR9	GNAI1	9606.ENSP00000350256	9606.ENSP00000343027	0	0.151	0.6	0.645
CCR9	JAK2	9606.ENSP00000350256	9606.ENSP00000371067	0	0.056	0.6	0.606
CCR9	GNAI3	9606.ENSP00000350256	9606.ENSP00000358867	0	0.151	0.6	0.645
CCR9	JAK3	9606.ENSP00000350256	9606.ENSP00000391676	0	0.056	0.6	0.606
CCR9	CXCL12	9606.ENSP00000350256	9606.ENSP00000379140	0	0.076	0.6	0.614
CD38	NAMPT	9606.ENSP00000226279	9606.ENSP00000222553	0	0	0.9	0.9
CD38	LCK	9606.ENSP00000226279	9606.ENSP00000337825	0.089	0.478	0	0.504
CD38	PRKCG	9606.ENSP00000226279	9606.ENSP00000263431	0	0	0.8	0.8
CD38	PRKCA	9606.ENSP00000226279	9606.ENSP00000408695	0	0	0.8	0.8
CD38	SIRT2	9606.ENSP00000226279	9606.ENSP00000249396	0	0	0.65	0.65
CD38	PRKCB	9606.ENSP00000226279	9606.ENSP00000305355	0.1	0	0.8	0.812
CD38	PNP	9606.ENSP00000226279	9606.ENSP00000354532	0	0	0.9	0.9
CDC25A	MAPK14	9606.ENSP00000303706	9606.ENSP00000229795	0.064	0.484	0	0.497
CDC25A	CDC25B	9606.ENSP00000303706	9606.ENSP00000245960	0	0	0.9	0.9
CDC25A	RAF1	9606.ENSP00000303706	9606.ENSP00000251849	0.062	0.87	0	0.873
CDC25A	CDK4	9606.ENSP00000303706	9606.ENSP00000257904	0.132	0.319	0.9	0.935
CDC25A	KIF11	9606.ENSP00000303706	9606.ENSP00000260731	0.571	0.102	0	0.598
CDC25A	EP300	9606.ENSP00000303706	9606.ENSP00000263253	0.049	0	0.9	0.9
CDC25A	CENPE	9606.ENSP00000303706	9606.ENSP00000265148	0.527	0.152	0	0.582
CDC25A	CDK2	9606.ENSP00000303706	9606.ENSP00000266970	0.221	0.785	0.9	0.981
CDC25A	MELK	9606.ENSP00000303706	9606.ENSP00000298048	0.573	0.151	0	0.622
CDC25A	PLK1	9606.ENSP00000303706	9606.ENSP00000300093	0.395	0.244	0.8	0.9
CDC25A	PKM	9606.ENSP00000303706	9606.ENSP00000320171	0.053	0.42	0	0.427
CDC25A	YWHAG	9606.ENSP00000303706	9606.ENSP00000306330	0.062	0.496	0	0.507
CDC25A	FEN1	9606.ENSP00000303706	9606.ENSP00000305480	0.548	0	0	0.548
CDC25A	TTK	9606.ENSP00000303706	9606.ENSP00000358813	0.549	0	0	0.549
CDC25A	TOP2A	9606.ENSP00000303706	9606.ENSP00000411532	0.544	0	0	0.544
CDC25A	PIM1	9606.ENSP00000303706	9606.ENSP00000362608	0.065	0.548	0	0.559
CDC25A	PSMB8	9606.ENSP00000303706	9606.ENSP00000364016	0	0	0.9	0.9
CDC25A	PSMB5	9606.ENSP00000303706	9606.ENSP00000355325	0.071	0	0.9	0.903
CDC25A	CDK5R1	9606.ENSP00000303706	9606.ENSP00000318486	0	0	0.9	0.9
CDC25A	CDK5	9606.ENSP00000303706	9606.ENSP00000419782	0	0.334	0.9	0.93
CDC25A	WEE1	9606.ENSP00000303706	9606.ENSP00000402084	0.131	0.528	0.65	0.843
CDC25A	CDK1	9606.ENSP00000303706	9606.ENSP00000378699	0.572	0.521	0.9	0.977
CDC25A	CHEK1	9606.ENSP00000303706	9606.ENSP00000388648	0.418	0.965	0.9	0.997
CDC25B	MAPK14	9606.ENSP00000245960	9606.ENSP00000229795	0.064	0.62	0.9	0.961
CDC25B	MELK	9606.ENSP00000245960	9606.ENSP00000298048	0.09	0.467	0	0.494
CDC25B	WEE1	9606.ENSP00000245960	9606.ENSP00000402084	0.064	0.528	0	0.539
CDC25B	CDK5R1	9606.ENSP00000245960	9606.ENSP00000318486	0	0	0.9	0.9
CDC25B	MAPKAPK2	9606.ENSP00000245960	9606.ENSP00000356070	0	0.27	0.9	0.923
CDC25B	CDK5	9606.ENSP00000245960	9606.ENSP00000419782	0	0.5	0.9	0.947
CDC25B	PLK1	9606.ENSP00000245960	9606.ENSP00000300093	0.186	0.244	0.8	0.866
CDC25B	YWHAG	9606.ENSP00000245960	9606.ENSP00000306330	0.062	0.799	0.9	0.979
CDC25B	CHEK1	9606.ENSP00000245960	9606.ENSP00000388648	0.065	0.805	0.8	0.96

CDC25B	CDK1	9606.ENSP00000245960	9606.ENSP00000378699	0.128	0.787	0.9	0.979
CDC25B	CDK2	9606.ENSP00000245960	9606.ENSP00000266970	0.117	0.789	0.9	0.979
CDC7	POLK	9606.ENSP00000393139	9606.ENSP00000241436	0.744	0.207	0	0.788
CDC7	CDK4	9606.ENSP00000393139	9606.ENSP00000257904	0.087	0.745	0	0.757
CDC7	KIF11	9606.ENSP00000393139	9606.ENSP00000260731	0.608	0	0	0.608
CDC7	DBF4	9606.ENSP00000393139	9606.ENSP00000265728	0.357	0.978	0.8	0.996
CDC7	CDK2	9606.ENSP00000393139	9606.ENSP00000266970	0.159	0.604	0.6	0.855
CDC7	TTK	9606.ENSP00000393139	9606.ENSP00000358813	0.608	0	0	0.608
CDC7	POLH	9606.ENSP00000393139	9606.ENSP00000361310	0.146	0.348	0	0.419
CDC7	POLA1	9606.ENSP00000393139	9606.ENSP00000368349	0.248	0.142	0.9	0.929
CDC7	PCNA	9606.ENSP00000393139	9606.ENSP00000368458	0.346	0.251	0	0.489
CDC7	CDK1	9606.ENSP00000393139	9606.ENSP00000378699	0.596	0.261	0	0.688
CDC7	CHEK1	9606.ENSP00000393139	9606.ENSP00000388648	0.403	0.262	0.65	0.832
CDK1	CSNK2A1	9606.ENSP00000378699	9606.ENSP00000217244	0.099	0.479	0	0.511
CDK1	PIN1	9606.ENSP00000378699	9606.ENSP00000247970	0.062	0.243	0.9	0.922
CDK1	CDK7	9606.ENSP00000378699	9606.ENSP00000256443	0.124	0.767	0.9	0.977
CDK1	CDK4	9606.ENSP00000378699	9606.ENSP00000257904	0.213	0.119	0.9	0.924
CDK1	MDM2	9606.ENSP00000378699	9606.ENSP00000258149	0	0	0.9	0.9
CDK1	KIF11	9606.ENSP00000378699	9606.ENSP00000260731	0.987	0.559	0	0.994
CDK1	CENPE	9606.ENSP00000378699	9606.ENSP00000265148	0.97	0.162	0	0.974
CDK1	DBF4	9606.ENSP00000378699	9606.ENSP00000265728	0.501	0.104	0	0.534
CDK1	CDK2	9606.ENSP00000378699	9606.ENSP00000266970	0.68	0.617	0.9	0.986
CDK1	SAE1	9606.ENSP00000378699	9606.ENSP00000270225	0.549	0.078	0	0.566
CDK1	EGFR	9606.ENSP00000378699	9606.ENSP00000275493	0	0.78	0	0.78
CDK1	SHH	9606.ENSP00000378699	9606.ENSP00000297261	0	0.078	0.9	0.903
CDK1	MELK	9606.ENSP00000378699	9606.ENSP00000298048	0.982	0.078	0	0.982
CDK1	PLK1	9606.ENSP00000378699	9606.ENSP00000300093	0.963	0.23	0.8	0.993
CDK1	MAP2K1	9606.ENSP00000378699	9606.ENSP00000302486	0.065	0.103	0.9	0.908
CDK1	FEN1	9606.ENSP00000378699	9606.ENSP00000305480	0.85	0.351	0	0.899
CDK1	PRKCE	9606.ENSP00000378699	9606.ENSP00000306124	0.049	0.073	0.9	0.904
CDK1	TYMS	9606.ENSP00000378699	9606.ENSP00000315644	0.977	0	0	0.977
CDK1	EZH2	9606.ENSP00000378699	9606.ENSP00000320147	0.684	0.876	0	0.959
CDK1	HSP90AA1	9606.ENSP00000378699	9606.ENSP00000335153	0.087	0.428	0	0.456
CDK1	MAPT	9606.ENSP00000378699	9606.ENSP00000340820	0	0.675	0	0.675
CDK1	TTK	9606.ENSP00000378699	9606.ENSP00000358813	0.99	0.13	0	0.991
CDK1	CDK9	9606.ENSP00000378699	9606.ENSP00000362361	0.054	0	0.9	0.901
CDK1	HDAC1	9606.ENSP00000378699	9606.ENSP00000362649	0.134	0.104	0.9	0.915
CDK1	SRC	9606.ENSP00000378699	9606.ENSP00000362680	0.062	0.285	0.9	0.927
CDK1	POLA1	9606.ENSP00000378699	9606.ENSP00000368349	0.463	0.349	0	0.636
CDK1	PCNA	9606.ENSP00000378699	9606.ENSP00000368458	0.982	0.629	0.36	0.995
CDK1	DUT	9606.ENSP00000378699	9606.ENSP00000370376	0.933	0.213	0	0.945
CDK1	CDK8	9606.ENSP00000378699	9606.ENSP00000370938	0.063	0.165	0.9	0.914
CDK1	DHFR	9606.ENSP00000378699	9606.ENSP00000396308	0.566	0	0	0.566
CDK1	LYN	9606.ENSP00000378699	9606.ENSP00000428924	0.062	0.861	0	0.864

CDK1	RXRA	9606.ENSP00000378699	9606.ENSP00000419692	0	0.057	0.9	0.901
CDK1	CDK5	9606.ENSP00000378699	9606.ENSP00000419782	0	0.068	0.9	0.902
CDK1	CHEK1	9606.ENSP00000378699	9606.ENSP00000388648	0.855	0.483	0	0.922
CDK1	XPO1	9606.ENSP00000378699	9606.ENSP00000384863	0.157	0.058	0.9	0.913
CDK1	TOP2A	9606.ENSP00000378699	9606.ENSP00000411532	0.988	0.225	0	0.99
CDK1	WEE1	9606.ENSP00000378699	9606.ENSP00000402084	0.19	0.782	0.9	0.98
CDK2	MAPK1	9606.ENSP00000266970	9606.ENSP00000215832	0.049	0.235	0.9	0.92
CDK2	CDK7	9606.ENSP00000266970	9606.ENSP00000256443	0.067	0.92	0.9	0.991
CDK2	CDK4	9606.ENSP00000266970	9606.ENSP00000257904	0.119	0.076	0.9	0.911
CDK2	MDM2	9606.ENSP00000266970	9606.ENSP00000258149	0.051	0	0.9	0.901
CDK2	KIF11	9606.ENSP00000266970	9606.ENSP00000260731	0.393	0.213	0	0.501
CDK2	MAPK3	9606.ENSP00000266970	9606.ENSP00000263025	0.049	0.235	0.9	0.92
CDK2	EP300	9606.ENSP00000266970	9606.ENSP00000263253	0.062	0.757	0	0.762
CDK2	DBF4	9606.ENSP00000266970	9606.ENSP00000265728	0.119	0.317	0.6	0.738
CDK2	SMARCA2	9606.ENSP00000266970	9606.ENSP00000265773	0.087	0.119	0.9	0.912
CDK2	DUT	9606.ENSP00000266970	9606.ENSP00000370376	0.479	0	0	0.479
CDK2	LRRK2	9606.ENSP00000266970	9606.ENSP00000298910	0.065	0.536	0	0.547
CDK2	POLL	9606.ENSP00000266970	9606.ENSP00000359181	0.058	0.463	0	0.472
CDK2	PLK1	9606.ENSP00000266970	9606.ENSP00000300093	0.283	0.23	0	0.424
CDK2	TTK	9606.ENSP00000266970	9606.ENSP00000358813	0.346	0.337	0	0.548
CDK2	LYN	9606.ENSP00000266970	9606.ENSP00000428924	0.062	0.473	0	0.484
CDK2	CDK5R1	9606.ENSP00000266970	9606.ENSP00000318486	0.062	0.459	0	0.47
CDK2	FEN1	9606.ENSP00000266970	9606.ENSP00000305480	0.324	0.351	0	0.543
CDK2	EZH2	9606.ENSP00000266970	9606.ENSP00000320147	0.199	0.282	0	0.401
CDK2	PGR	9606.ENSP00000266970	9606.ENSP00000325120	0	0.494	0	0.494
CDK2	TYMS	9606.ENSP00000266970	9606.ENSP00000315644	0.686	0	0	0.686
CDK2	CHEK1	9606.ENSP00000266970	9606.ENSP00000388648	0.321	0.36	0.65	0.834
CDK2	CDK5	9606.ENSP00000266970	9606.ENSP00000419782	0	0.068	0.9	0.902
CDK2	PTGDR	9606.ENSP00000266970	9606.ENSP00000303424	0	0	0.9	0.9
CDK2	CDK9	9606.ENSP00000266970	9606.ENSP00000362361	0.072	0	0.9	0.903
CDK2	CDK8	9606.ENSP00000266970	9606.ENSP00000370938	0.062	0.165	0.9	0.914
CDK2	WEE1	9606.ENSP00000266970	9606.ENSP00000402084	0.113	0.465	0.8	0.897
CDK2	TERT	9606.ENSP00000266970	9606.ENSP00000309572	0.076	0	0.9	0.903
CDK2	HDAC1	9606.ENSP00000266970	9606.ENSP00000362649	0.133	0.104	0.9	0.915
CDK2	POLA1	9606.ENSP00000266970	9606.ENSP00000368349	0.226	0.543	0.9	0.961
CDK2	PCNA	9606.ENSP00000266970	9606.ENSP00000368458	0.684	0.992	0.9	0.999
CDK4	GLI1	9606.ENSP00000257904	9606.ENSP00000228682	0.054	0.448	0	0.455
CDK4	FABP4	9606.ENSP00000257904	9606.ENSP00000256104	0	0	0.9	0.9
CDK4	CDK7	9606.ENSP00000257904	9606.ENSP00000256443	0.063	0.47	0.9	0.946
CDK4	JAK3	9606.ENSP00000257904	9606.ENSP00000391676	0.062	0.434	0	0.447
CDK4	PLK1	9606.ENSP00000257904	9606.ENSP00000300093	0.175	0.374	0	0.461
CDK4	PBRM1	9606.ENSP00000257904	9606.ENSP00000378307	0.062	0.064	0.65	0.665
CDK4	SMARCA2	9606.ENSP00000257904	9606.ENSP00000265773	0.082	0.119	0.65	0.692
CDK4	DNMT3A	9606.ENSP00000257904	9606.ENSP00000264709	0	0	0.65	0.65

CDK4	SMARCA4	9606.ENSP00000257904	9606.ENSP00000395654	0.115	0.119	0.65	0.703
CDK4	CDK9	9606.ENSP00000257904	9606.ENSP00000362361	0.062	0	0.9	0.902
CDK4	CDK5	9606.ENSP00000257904	9606.ENSP00000419782	0.061	0.119	0.9	0.91
CDK4	CDK8	9606.ENSP00000257904	9606.ENSP00000370938	0.062	0.165	0.9	0.914
CDK4	GSK3B	9606.ENSP00000257904	9606.ENSP00000324806	0.064	0.061	0.9	0.904
CDK4	JAK2	9606.ENSP00000257904	9606.ENSP00000371067	0.062	0.133	0.9	0.911
CDK4	LYN	9606.ENSP00000257904	9606.ENSP00000428924	0.062	0.149	0.9	0.913
CDK4	SRC	9606.ENSP00000257904	9606.ENSP00000362680	0.062	0.061	0.9	0.904
CDK4	HDAC1	9606.ENSP00000257904	9606.ENSP00000362649	0.097	0.243	0.9	0.925
CDK4	HSP90AA1	9606.ENSP00000257904	9606.ENSP00000335153	0.062	0.889	0	0.891
CDK4	PCNA	9606.ENSP00000257904	9606.ENSP00000368458	0.301	0.98	0.9	0.998
CDK5	NR3C1	9606.ENSP00000419782	9606.ENSP00000231509	0.046	0	0.9	0.9
CDK5	CDK7	9606.ENSP00000419782	9606.ENSP00000256443	0.105	0.27	0.9	0.929
CDK5	EPHA4	9606.ENSP00000419782	9606.ENSP00000281821	0	0.056	0.9	0.901
CDK5	PPARG	9606.ENSP00000419782	9606.ENSP00000287820	0.046	0.675	0	0.676
CDK5	LRRK2	9606.ENSP00000419782	9606.ENSP00000298910	0	0.501	0	0.501
CDK5	MAP2K1	9606.ENSP00000419782	9606.ENSP00000302486	0.062	0.213	0.9	0.919
CDK5	CDK5R1	9606.ENSP00000419782	9606.ENSP00000318486	0.062	0.995	0.9	0.999
CDK5	GSK3B	9606.ENSP00000419782	9606.ENSP00000324806	0.062	0.47	0.9	0.945
CDK5	EPHA3	9606.ENSP00000419782	9606.ENSP00000337451	0	0.056	0.9	0.901
CDK5	MAPT	9606.ENSP00000419782	9606.ENSP00000340820	0.062	0.905	0.9	0.99
CDK5	RAC1	9606.ENSP00000419782	9606.ENSP00000348461	0	0	0.9	0.9
CDK5	JUN	9606.ENSP00000419782	9606.ENSP00000360266	0	0	0.9	0.9
CDK5	DNM1	9606.ENSP00000419782	9606.ENSP00000362014	0.062	0.477	0	0.488
CDK5	PCNA	9606.ENSP00000419782	9606.ENSP00000368458	0	0.98	0	0.98
CDK5	CDK8	9606.ENSP00000419782	9606.ENSP00000370938	0	0	0.9	0.9
CDK5R1	NR3C1	9606.ENSP00000318486	9606.ENSP00000231509	0.062	0	0.9	0.902
CDK5R1	MAP2K1	9606.ENSP00000318486	9606.ENSP00000302486	0	0	0.9	0.9
CDK5R1	CDK9	9606.ENSP00000318486	9606.ENSP00000362361	0	0.462	0	0.461
CDK5R1	MAPT	9606.ENSP00000318486	9606.ENSP00000340820	0.136	0	0.8	0.819
CDK5R1	JUN	9606.ENSP00000318486	9606.ENSP00000360266	0	0	0.9	0.9
CDK5R1	RAC1	9606.ENSP00000318486	9606.ENSP00000348461	0	0	0.9	0.9
CDK5R1	GSK3B	9606.ENSP00000318486	9606.ENSP00000324806	0.062	0.37	0.9	0.935
CDK7	PARP2	9606.ENSP00000256443	9606.ENSP00000250416	0.063	0	0.6	0.609
CDK7	RARA	9606.ENSP00000256443	9606.ENSP00000254066	0	0.213	0.9	0.917
CDK7	PARP1	9606.ENSP00000256443	9606.ENSP00000355759	0.065	0	0.6	0.61
CDK7	EP300	9606.ENSP00000256443	9606.ENSP00000263253	0.062	0	0.6	0.608
CDK7	ESR1	9606.ENSP00000256443	9606.ENSP00000405330	0	0.462	0	0.461
CDK7	RARG	9606.ENSP00000256443	9606.ENSP00000388510	0	0	0.9	0.9
CDK7	CDK8	9606.ENSP00000256443	9606.ENSP00000370938	0.072	0.149	0.9	0.914
CDK7	ERCC5	9606.ENSP00000256443	9606.ENSP00000347978	0.062	0.682	0.6	0.87
CDK7	CDK9	9606.ENSP00000256443	9606.ENSP00000362361	0.096	0.684	0.9	0.969
CDK8	FABP4	9606.ENSP00000370938	9606.ENSP00000256104	0	0	0.9	0.9
CDK8	EP300	9606.ENSP00000370938	9606.ENSP00000263253	0.064	0.062	0.6	0.618

CDK8	KAT2B	9606.ENSP00000370938	9606.ENSP00000263754	0.108	0.435	0.6	0.78
CDK8	PPARG	9606.ENSP00000370938	9606.ENSP00000287820	0	0.138	0.6	0.64
CDK8	PLIN1	9606.ENSP00000370938	9606.ENSP00000300055	0	0	0.9	0.9
CDK8	LPL	9606.ENSP00000370938	9606.ENSP00000309757	0	0	0.9	0.9
CDK8	PARP1	9606.ENSP00000370938	9606.ENSP00000355759	0	0.486	0	0.485
CDK8	CDK9	9606.ENSP00000370938	9606.ENSP00000362361	0.054	0.486	0.6	0.788
CDK8	RXRA	9606.ENSP00000370938	9606.ENSP00000419692	0	0.159	0.6	0.649
CDK9	PRKDC	9606.ENSP00000362361	9606.ENSP00000313420	0.072	0.482	0	0.498
CDK9	PLEC	9606.ENSP00000362361	9606.ENSP00000323856	0	0.47	0	0.47
CDK9	HSP90AA1	9606.ENSP00000362361	9606.ENSP00000335153	0	0.707	0	0.707
CDK9	ESR1	9606.ENSP00000362361	9606.ENSP00000405330	0	0	0.6	0.6
CDK9	HSPA1A	9606.ENSP00000362361	9606.ENSP00000364802	0	0.69	0	0.69
CEL	STS	9606.ENSP00000361151	9606.ENSP00000217961	0	0	0.8	0.8
CEL	LIPE	9606.ENSP00000361151	9606.ENSP00000244289	0.125	0	0.9	0.908
CEL	LIPG	9606.ENSP00000361151	9606.ENSP00000261292	0	0	0.8	0.8
CEL	MGLL	9606.ENSP00000361151	9606.ENSP00000265052	0	0	0.9	0.9
CEL	LIPC	9606.ENSP00000361151	9606.ENSP00000299022	0.062	0	0.8	0.804
CEL	LPL	9606.ENSP00000361151	9606.ENSP00000309757	0	0	0.9	0.9
CEL	LIPA	9606.ENSP00000361151	9606.ENSP00000337354	0.062	0	0.8	0.804
CEL	DHCR7	9606.ENSP00000361151	9606.ENSP00000347717	0	0	0.9	0.9
CEL	CTRB1	9606.ENSP00000361151	9606.ENSP00000354294	0.66	0.056	0	0.666
CEL	CTRC	9606.ENSP00000361151	9606.ENSP00000365116	0.479	0.056	0	0.487
CENPE	KIF11	9606.ENSP00000265148	9606.ENSP00000260731	0.969	0.369	0.5	0.989
CENPE	FEN1	9606.ENSP00000265148	9606.ENSP00000305480	0.412	0	0	0.412
CENPE	TYMS	9606.ENSP00000265148	9606.ENSP00000315644	0.556	0	0	0.556
CENPE	CHEK1	9606.ENSP00000265148	9606.ENSP00000388648	0.689	0	0	0.689
CENPE	MELK	9606.ENSP00000265148	9606.ENSP00000298048	0.842	0.056	0	0.844
CENPE	TOP2A	9606.ENSP00000265148	9606.ENSP00000411532	0.959	0	0	0.959
CENPE	TTK	9606.ENSP00000265148	9606.ENSP00000358813	0.983	0.15	0	0.985
CENPE	PLK1	9606.ENSP00000265148	9606.ENSP00000300093	0.915	0.15	0.9	0.992
CES1	MPO	9606.ENSP00000353720	9606.ENSP00000225275	0	0.064	0.9	0.902
CES1	UGT2B7	9606.ENSP00000353720	9606.ENSP00000304811	0.071	0	0.65	0.66
CES1	CES2	9606.ENSP00000353720	9606.ENSP00000317842	0.062	0	0.8	0.804
CES1	CYP3A4	9606.ENSP00000353720	9606.ENSP00000337915	0.062	0.06	0.9	0.904
CES1	RBP4	9606.ENSP00000353720	9606.ENSP00000360522	0.146	0	0.9	0.911
CES2	UGT2B7	9606.ENSP00000317842	9606.ENSP00000304811	0.064	0	0.65	0.658
CES2	RBP4	9606.ENSP00000317842	9606.ENSP00000360522	0.062	0	0.9	0.902
CES2	CYP3A4	9606.ENSP00000317842	9606.ENSP00000337915	0.053	0.06	0.9	0.903
CETP	MSR1	9606.ENSP00000200676	9606.ENSP00000262101	0.062	0	0.54	0.55
CETP	PLA2G7	9606.ENSP00000200676	9606.ENSP00000274793	0	0	0.72	0.72
CETP	RXRB	9606.ENSP00000200676	9606.ENSP00000363817	0	0	0.9	0.9
CETP	RXRA	9606.ENSP00000200676	9606.ENSP00000419692	0	0	0.9	0.9
CETP	LPL	9606.ENSP00000200676	9606.ENSP00000309757	0.065	0.213	0.54	0.631
CETP	NR1H2	9606.ENSP00000200676	9606.ENSP00000253727	0	0	0.9	0.9

CETP	LIPC	9606.ENSP00000200676	9606.ENSP00000299022	0.09	0	0.54	0.563
CETP	NR1H3	9606.ENSP00000200676	9606.ENSP00000477707	0	0	0.9	0.9
CHEK1	CSNK2A1	9606.ENSP00000388648	9606.ENSP00000217244	0.062	0.499	0	0.51
CHEK1	MDM2	9606.ENSP00000388648	9606.ENSP00000258149	0	0	0.9	0.9
CHEK1	KIF11	9606.ENSP00000388648	9606.ENSP00000260731	0.821	0	0	0.821
CHEK1	DBF4	9606.ENSP00000388648	9606.ENSP00000265728	0.27	0.231	0.65	0.786
CHEK1	KIT	9606.ENSP00000388648	9606.ENSP00000288135	0	0.057	0.6	0.606
CHEK1	MELK	9606.ENSP00000388648	9606.ENSP00000298048	0.84	0.059	0	0.843
CHEK1	PLK1	9606.ENSP00000388648	9606.ENSP00000300093	0.59	0	0	0.59
CHEK1	FEN1	9606.ENSP00000388648	9606.ENSP00000305480	0.778	0.144	0	0.802
CHEK1	YWHAG	9606.ENSP00000388648	9606.ENSP00000306330	0	0.647	0	0.647
CHEK1	PRKDC	9606.ENSP00000388648	9606.ENSP00000313420	0.1	0.736	0	0.752
CHEK1	TYMS	9606.ENSP00000388648	9606.ENSP00000315644	0.81	0.241	0	0.85
CHEK1	EZH2	9606.ENSP00000388648	9606.ENSP00000320147	0.557	0	0	0.557
CHEK1	HSP90AA1	9606.ENSP00000388648	9606.ENSP00000335153	0.062	0.655	0	0.662
CHEK1	DNMT1	9606.ENSP00000388648	9606.ENSP00000352516	0.16	0.486	0	0.55
CHEK1	MTOR	9606.ENSP00000388648	9606.ENSP00000354558	0	0.513	0	0.513
CHEK1	TTK	9606.ENSP00000388648	9606.ENSP00000358813	0.805	0	0	0.805
CHEK1	POLA1	9606.ENSP00000388648	9606.ENSP00000368349	0.321	0.642	0	0.746
CHEK1	PCNA	9606.ENSP00000388648	9606.ENSP00000368458	0.584	0.548	0	0.804
CHEK1	TOP2A	9606.ENSP00000388648	9606.ENSP00000411532	0.803	0.205	0	0.836
CHEK1	WEE1	9606.ENSP00000388648	9606.ENSP00000402084	0.131	0.391	0.9	0.942
CHRFAM7A	CHRND	9606.ENSP00000299847	9606.ENSP00000258385	0.063	0.161	0.54	0.606
CHRFAM7A	CHRNB4	9606.ENSP00000299847	9606.ENSP00000261751	0.066	0.324	0.54	0.684
CHRFAM7A	CHRNG	9606.ENSP00000299847	9606.ENSP00000374145	0.063	0.161	0.54	0.606
CHRFAM7A	CHRNB1	9606.ENSP00000299847	9606.ENSP00000304290	0.063	0.161	0.54	0.606
CHRFAM7A	CHRNA7	9606.ENSP00000299847	9606.ENSP00000407546	0	0.27	0.54	0.649
CHRFAM7A	CHRNA4	9606.ENSP00000299847	9606.ENSP00000359285	0.066	0.213	0.54	0.632
CHRFAM7A	CHRNA3	9606.ENSP00000299847	9606.ENSP00000315602	0.066	0.213	0.54	0.632
CHRFAM7A	CHRNB2	9606.ENSP00000299847	9606.ENSP00000357461	0.066	0.368	0.54	0.704
CHRM1	CHRM3	9606.ENSP00000306490	9606.ENSP00000255380	0.119	0	0.8	0.816
CHRM1	HTR2C	9606.ENSP00000306490	9606.ENSP00000276198	0.159	0	0.9	0.912
CHRM1	HTR5A	9606.ENSP00000306490	9606.ENSP00000287907	0.487	0	0	0.487
CHRM1	PTGER1	9606.ENSP00000306490	9606.ENSP00000292513	0	0	0.9	0.9
CHRM1	GRM5	9606.ENSP00000306490	9606.ENSP00000306138	0.352	0	0.8	0.864
CHRM1	GRIN1	9606.ENSP00000306490	9606.ENSP00000360608	0.533	0	0	0.533
CHRM1	PIK3CG	9606.ENSP00000306490	9606.ENSP00000352121	0	0	0.6	0.6
CHRM1	GNAI3	9606.ENSP00000306490	9606.ENSP00000358867	0	0	0.6	0.6
CHRM1	GNAI1	9606.ENSP00000306490	9606.ENSP00000343027	0.061	0	0.6	0.608
CHRM1	LPAR4	9606.ENSP00000306490	9606.ENSP00000408205	0	0	0.8	0.8
CHRM1	LPAR2	9606.ENSP00000306490	9606.ENSP00000443256	0	0	0.8	0.8
CHRM1	LPAR3	9606.ENSP00000306490	9606.ENSP00000395389	0	0	0.8	0.8
CHRM1	F2R	9606.ENSP00000306490	9606.ENSP00000321326	0	0	0.8	0.8
CHRM1	CHRM5	9606.ENSP00000306490	9606.ENSP00000372750	0.062	0	0.8	0.804

CHRM1	CHRM2	9606.ENSP00000306490	9606.ENSP00000399745	0.059	0	0.8	0.803
CHRM1	LPAR1	9606.ENSP00000306490	9606.ENSP00000363553	0	0	0.8	0.8
CHRM1	LPAR6	9606.ENSP00000306490	9606.ENSP00000367691	0	0	0.8	0.8
CHRM1	F2	9606.ENSP00000306490	9606.ENSP00000308541	0	0	0.9	0.9
CHRM1	JUN	9606.ENSP00000306490	9606.ENSP00000360266	0	0	0.9	0.9
CHRM1	CXCL12	9606.ENSP00000306490	9606.ENSP00000379140	0	0	0.9	0.9
CHRM1	PTK2	9606.ENSP00000306490	9606.ENSP00000341189	0	0	0.9	0.9
CHRM1	SRC	9606.ENSP00000306490	9606.ENSP00000362680	0	0	0.9	0.9
CHRM2	F2R	9606.ENSP00000399745	9606.ENSP00000321326	0	0	0.8	0.8
CHRM2	GNAI1	9606.ENSP00000399745	9606.ENSP00000343027	0.049	0	0.6	0.603
CHRM2	PIK3CG	9606.ENSP00000399745	9606.ENSP00000352121	0	0	0.6	0.6
CHRM2	GNAI3	9606.ENSP00000399745	9606.ENSP00000358867	0.049	0	0.6	0.603
CHRM2	LPAR1	9606.ENSP00000399745	9606.ENSP00000363553	0.076	0	0.8	0.807
CHRM2	LPAR6	9606.ENSP00000399745	9606.ENSP00000367691	0	0	0.8	0.8
CHRM2	LPAR3	9606.ENSP00000399745	9606.ENSP00000395389	0	0	0.8	0.8
CHRM2	LPAR4	9606.ENSP00000399745	9606.ENSP00000408205	0	0	0.8	0.8
CHRM2	LPAR2	9606.ENSP00000399745	9606.ENSP00000443256	0	0	0.8	0.8
CHRM3	GRM5	9606.ENSP00000255380	9606.ENSP00000306138	0.108	0	0.8	0.814
CHRM3	CHRM5	9606.ENSP00000255380	9606.ENSP00000372750	0.062	0.312	0.8	0.859
CHRM4	GNAI1	9606.ENSP00000409378	9606.ENSP00000343027	0	0	0.6	0.6
CHRM4	GNAI3	9606.ENSP00000409378	9606.ENSP00000358867	0	0	0.6	0.6
CHRM5	GRM5	9606.ENSP00000372750	9606.ENSP00000306138	0.062	0	0.8	0.804
CHRNA3	CHRND	9606.ENSP00000315602	9606.ENSP00000258385	0	0	0.6	0.6
CHRNA3	CHRNB4	9606.ENSP00000315602	9606.ENSP00000261751	0.063	0.382	0.9	0.937
CHRNA3	CHRNB1	9606.ENSP00000315602	9606.ENSP00000304290	0	0	0.72	0.72
CHRNA3	CHRNG	9606.ENSP00000315602	9606.ENSP00000374145	0.062	0	0.6	0.608
CHRNA3	JAK2	9606.ENSP00000315602	9606.ENSP00000371067	0.062	0.056	0.8	0.807
CHRNA3	CHRNA4	9606.ENSP00000315602	9606.ENSP00000359285	0.062	0	0.9	0.902
CHRNA3	CHRNA7	9606.ENSP00000315602	9606.ENSP00000407546	0.066	0.213	0.9	0.92
CHRNA3	CHRNB2	9606.ENSP00000315602	9606.ENSP00000357461	0.062	0.427	0.9	0.941
CHRNA4	CHRND	9606.ENSP00000359285	9606.ENSP00000258385	0	0	0.6	0.6
CHRNA4	CHRNB4	9606.ENSP00000359285	9606.ENSP00000261751	0.056	0.361	0.9	0.934
CHRNA4	CHRNB1	9606.ENSP00000359285	9606.ENSP00000304290	0	0	0.72	0.72
CHRNA4	CHRNB2	9606.ENSP00000359285	9606.ENSP00000357461	0.214	0.796	0.9	0.982
CHRNA4	CHRNG	9606.ENSP00000359285	9606.ENSP00000374145	0.062	0	0.6	0.608
CHRNA4	JAK2	9606.ENSP00000359285	9606.ENSP00000371067	0.062	0.056	0.8	0.807
CHRNA4	CHRNA7	9606.ENSP00000359285	9606.ENSP00000407546	0.066	0.213	0.9	0.92
CHRNA7	CHRND	9606.ENSP00000407546	9606.ENSP00000258385	0.063	0.161	0.54	0.606
CHRNA7	CHRNB4	9606.ENSP00000407546	9606.ENSP00000261751	0.088	0.324	0.9	0.932
CHRNA7	CHRNB1	9606.ENSP00000407546	9606.ENSP00000304290	0.063	0.161	0.72	0.76
CHRNA7	CHRNB2	9606.ENSP00000407546	9606.ENSP00000357461	0.066	0.368	0.9	0.935
CHRNA7	CHRNG	9606.ENSP00000407546	9606.ENSP00000374145	0.063	0.161	0.54	0.606
CHRNB1	CHRND	9606.ENSP00000304290	9606.ENSP00000258385	0.063	0	0.54	0.55
CHRNB1	CHRNB4	9606.ENSP00000304290	9606.ENSP00000261751	0	0	0.72	0.72

CHRNB1	CHRNB2	9606.ENSP00000304290	9606.ENSP00000357461	0	0	0.72	0.72
CHRNB1	PTK2	9606.ENSP00000304290	9606.ENSP00000341189	0.051	0	0.9	0.901
CHRNB1	PTK2B	9606.ENSP00000304290	9606.ENSP00000380638	0.051	0	0.9	0.901
CHRNB1	SRC	9606.ENSP00000304290	9606.ENSP00000362680	0	0.056	0.9	0.901
CHRNB1	CHRNG	9606.ENSP00000304290	9606.ENSP00000374145	0	0	0.9	0.9
CHRNB2	CHRND	9606.ENSP00000357461	9606.ENSP00000258385	0	0	0.6	0.6
CHRNB2	CHRNB4	9606.ENSP00000357461	9606.ENSP00000261751	0.062	0.176	0.9	0.915
CHRNB2	CHRNG	9606.ENSP00000357461	9606.ENSP00000374145	0.063	0	0.6	0.609
CHRNB2	JAK2	9606.ENSP00000357461	9606.ENSP00000371067	0.062	0.056	0.8	0.807
CHRNB4	CHRND	9606.ENSP00000261751	9606.ENSP00000258385	0	0	0.6	0.6
CHRNB4	CHRNG	9606.ENSP00000261751	9606.ENSP00000374145	0.063	0	0.6	0.609
CHRNB4	JAK2	9606.ENSP00000261751	9606.ENSP00000371067	0.062	0.056	0.8	0.807
CHRND	CHRNG	9606.ENSP00000258385	9606.ENSP00000374145	0.476	0	0.54	0.749
CHRNG	PTK2	9606.ENSP00000374145	9606.ENSP00000341189	0.051	0	0.9	0.901
CHRNG	SRC	9606.ENSP00000374145	9606.ENSP00000362680	0	0.056	0.9	0.901
CHRNG	PTK2B	9606.ENSP00000374145	9606.ENSP00000380638	0.051	0	0.9	0.901
CLK1	CLK4	9606.ENSP00000394734	9606.ENSP00000316948	0.956	0	0.8	0.99
CLK1	CLK2	9606.ENSP00000394734	9606.ENSP00000357345	0	0.677	0	0.677
CLK1	PTPN1	9606.ENSP00000394734	9606.ENSP00000360683	0.082	0.497	0	0.518
CLK1	CLK3	9606.ENSP00000394734	9606.ENSP00000378505	0.062	0.397	0	0.41
CLK2	PTPN1	9606.ENSP00000357345	9606.ENSP00000360683	0.082	0.497	0	0.518
CLK2	CLK3	9606.ENSP00000357345	9606.ENSP00000378505	0	0.882	0	0.882
CLK3	NCOR2	9606.ENSP00000378505	9606.ENSP00000384018	0.076	0.392	0	0.414
CNR1	MTNR1B	9606.ENSP00000358513	9606.ENSP00000257068	0	0	0.6	0.6
CNR1	MTNR1A	9606.ENSP00000358513	9606.ENSP00000302811	0	0	0.6	0.6
CNR1	GNAI1	9606.ENSP00000358513	9606.ENSP00000343027	0.062	0.935	0.8	0.986
CNR1	GPR18	9606.ENSP00000358513	9606.ENSP00000343428	0	0	0.6	0.6
CNR1	DRD2	9606.ENSP00000358513	9606.ENSP00000354859	0.089	0.322	0.8	0.865
CNR1	HCAR2	9606.ENSP00000358513	9606.ENSP00000375066	0	0	0.6	0.6
CNR1	GNAI3	9606.ENSP00000358513	9606.ENSP00000358867	0.062	0.353	0.8	0.868
CNR1	CNR2	9606.ENSP00000358513	9606.ENSP00000363596	0	0.634	0.8	0.923
CNR2	MTNR1B	9606.ENSP00000363596	9606.ENSP00000257068	0	0	0.6	0.6
CNR2	MTNR1A	9606.ENSP00000363596	9606.ENSP00000302811	0	0	0.6	0.6
CNR2	GNAI1	9606.ENSP00000363596	9606.ENSP00000343027	0	0.822	0	0.822
CNR2	GPR18	9606.ENSP00000363596	9606.ENSP00000343428	0.107	0.322	0.6	0.737
CNR2	HCAR2	9606.ENSP00000363596	9606.ENSP00000375066	0	0	0.6	0.6
COMT	PNMT	9606.ENSP00000354511	9606.ENSP00000269582	0	0	0.9	0.9
COMT	UGT2B7	9606.ENSP00000354511	9606.ENSP00000304811	0.062	0.134	0.65	0.69
COMT	MAOA	9606.ENSP00000354511	9606.ENSP00000340684	0	0	0.9	0.9
COMT	MAOB	9606.ENSP00000354511	9606.ENSP00000367309	0	0	0.9	0.9
CPB2	GYS1	9606.ENSP00000181383	9606.ENSP00000317904	0.48	0	0	0.48
CPB2	FFAR4	9606.ENSP00000181383	9606.ENSP00000360538	0.509	0	0	0.509
CPB2	FABP1	9606.ENSP00000181383	9606.ENSP00000295834	0.608	0	0	0.608
CPB2	NR1H4	9606.ENSP00000181383	9606.ENSP00000447149	0.683	0	0	0.683

CPB2	TTR	9606.ENSP00000181383	9606.ENSP00000237014	0.662	0	0	0.662
CPB2	CTRC	9606.ENSP00000181383	9606.ENSP00000365116	0.124	0.344	0	0.4
CPB2	F2R	9606.ENSP00000181383	9606.ENSP00000321326	0	0	0.9	0.9
CPB2	F2	9606.ENSP00000181383	9606.ENSP00000308541	0.602	0.495	0.9	0.978
CPT1A	RORA	9606.ENSP00000265641	9606.ENSP00000261523	0	0	0.9	0.9
CPT1A	EP300	9606.ENSP00000265641	9606.ENSP00000263253	0	0	0.9	0.9
CPT1A	NDUFV1	9606.ENSP00000265641	9606.ENSP00000322450	0.049	0.401	0	0.405
CPT1A	RELA	9606.ENSP00000265641	9606.ENSP00000384273	0	0	0.9	0.9
CPT1A	JUN	9606.ENSP00000265641	9606.ENSP00000360266	0	0	0.9	0.9
CPT1A	RXRA	9606.ENSP00000265641	9606.ENSP00000419692	0.062	0.057	0.9	0.903
CPT1A	NR1H3	9606.ENSP00000265641	9606.ENSP00000477707	0.062	0.057	0.9	0.903
CPT1A	LPL	9606.ENSP00000265641	9606.ENSP00000309757	0.049	0	0.9	0.9
CPT1A	FABP1	9606.ENSP00000265641	9606.ENSP00000295834	0.062	0	0.9	0.902
CPT1A	PPARA	9606.ENSP00000265641	9606.ENSP00000385523	0.062	0.057	0.9	0.903
CRHR1	GNAO1	9606.ENSP00000381333	9606.ENSP00000262494	0.249	0.366	0	0.504
CRHR1	HTR5A	9606.ENSP00000381333	9606.ENSP00000287907	0.508	0	0	0.508
CRHR1	GNAI1	9606.ENSP00000381333	9606.ENSP00000343027	0.089	0.366	0.8	0.874
CRHR1	GNAI3	9606.ENSP00000381333	9606.ENSP00000358867	0.062	0.168	0.8	0.83
CRHR1	GRIN1	9606.ENSP00000381333	9606.ENSP00000360608	0.402	0	0	0.402
CRYAB	HSP90AA1	9606.ENSP00000433560	9606.ENSP00000335153	0.065	0.143	0.9	0.912
CRYAB	HSPA1A	9606.ENSP00000433560	9606.ENSP00000364802	0.087	0.141	0.9	0.914
CSF1R	F13A1	9606.ENSP00000286301	9606.ENSP00000264870	0.588	0	0	0.588
CSF1R	YES1	9606.ENSP00000286301	9606.ENSP00000462468	0	0.52	0	0.52
CSF1R	FGR	9606.ENSP00000286301	9606.ENSP00000363117	0.556	0.122	0	0.594
CSF1R	HCK	9606.ENSP00000286301	9606.ENSP00000444986	0.568	0.122	0	0.604
CSF1R	MPEG1	9606.ENSP00000286301	9606.ENSP00000354335	0.671	0	0	0.671
CSF1R	SRC	9606.ENSP00000286301	9606.ENSP00000362680	0	0.767	0	0.767
CSF1R	ITGB2	9606.ENSP00000286301	9606.ENSP00000380948	0.775	0.077	0	0.784
CSF1R	CTSS	9606.ENSP00000286301	9606.ENSP00000357981	0.713	0.078	0	0.724
CSF1R	PTPRC	9606.ENSP00000286301	9606.ENSP00000411355	0.556	0.06	0	0.565
CSF1R	PTPN1	9606.ENSP00000286301	9606.ENSP00000360683	0	0.06	0.9	0.901
CSF1R	PTPN2	9606.ENSP00000286301	9606.ENSP00000311857	0	0.06	0.9	0.901
CSF1R	PIK3R1	9606.ENSP00000286301	9606.ENSP00000428056	0.076	0.84	0	0.846
CSNK2A1	PSEN2	9606.ENSP00000217244	9606.ENSP00000355747	0	0.463	0	0.463
CSNK2A1	PTPN1	9606.ENSP00000217244	9606.ENSP00000360683	0.083	0.472	0	0.495
CSNK2A1	KDM1A	9606.ENSP00000217244	9606.ENSP00000383042	0.065	0.486	0	0.498
CSNK2A1	VHL	9606.ENSP00000217244	9606.ENSP00000256474	0	0.486	0	0.485
CSNK2A1	HDAC3	9606.ENSP00000217244	9606.ENSP00000302967	0.093	0.549	0	0.574
CSNK2A1	MDM2	9606.ENSP00000217244	9606.ENSP00000258149	0.062	0.488	0	0.499
CSNK2A1	WEE1	9606.ENSP00000217244	9606.ENSP00000402084	0.064	0.47	0	0.483
CSNK2A1	PIN1	9606.ENSP00000217244	9606.ENSP00000247970	0.241	0.27	0	0.422
CSNK2A1	MME	9606.ENSP00000217244	9606.ENSP00000418525	0	0.487	0	0.487
CSNK2A1	TOP2A	9606.ENSP00000217244	9606.ENSP00000411532	0.085	0.807	0	0.816
CSNK2A1	HSP90AA1	9606.ENSP00000217244	9606.ENSP00000335153	0.211	0.763	0	0.805

CSNK2A1	HDAC1	9606.ENSP00000217244	9606.ENSP00000362649	0.129	0.834	0.72	0.956
CSNK2A1	JUN	9606.ENSP00000217244	9606.ENSP00000360266	0	0.674	0.9	0.966
CSNK2A1	HDAC2	9606.ENSP00000217244	9606.ENSP00000430432	0.123	0.888	0.72	0.97
CTRB1	MMP3	9606.ENSP00000354294	9606.ENSP00000299855	0	0	0.9	0.9
CTRB1	PRSS1	9606.ENSP00000354294	9606.ENSP00000308720	0.208	0.265	0.5	0.683
CTRB1	MMP9	9606.ENSP00000354294	9606.ENSP00000361405	0	0	0.9	0.9
CTRC	PRSS1	9606.ENSP00000365116	9606.ENSP00000308720	0.228	0	0.9	0.919
CTSB	CTSD	9606.ENSP00000345672	9606.ENSP00000236671	0.225	0.677	0.5	0.864
CTSB	SPHK1	9606.ENSP00000345672	9606.ENSP00000313681	0.077	0.463	0	0.483
CTSB	NLRP3	9606.ENSP00000345672	9606.ENSP00000337383	0.062	0	0.8	0.804
CTSB	CTSL	9606.ENSP00000345672	9606.ENSP00000345344	0.244	0.157	0.8	0.861
CTSB	CTSV	9606.ENSP00000345672	9606.ENSP00000445052	0.155	0.157	0.3	0.457
CTSB	MMP9	9606.ENSP00000345672	9606.ENSP00000361405	0.069	0.27	0.3	0.482
CTSB	CTSS	9606.ENSP00000345672	9606.ENSP00000357981	0.104	0	0.8	0.813
CTSC	GRIA1	9606.ENSP00000227266	9606.ENSP00000428994	0	0	0.6	0.6
CTSD	LDHA	9606.ENSP00000236671	9606.ENSP00000445175	0.063	0.401	0	0.414
CTSD	CTSK	9606.ENSP00000236671	9606.ENSP00000271651	0.086	0.219	0.5	0.612
CTSD	TPO	9606.ENSP00000236671	9606.ENSP00000318820	0.089	0	0.9	0.905
CTSD	EP300	9606.ENSP00000236671	9606.ENSP00000263253	0.063	0.057	0.9	0.903
CTSD	CTSL	9606.ENSP00000236671	9606.ENSP00000345344	0.136	0.219	0.5	0.633
CTSD	ESR1	9606.ENSP00000236671	9606.ENSP00000405330	0.088	0.056	0.9	0.906
CTSK	CTSS	9606.ENSP00000271651	9606.ENSP00000357981	0.246	0	0.3	0.449
CTSK	CTSV	9606.ENSP00000271651	9606.ENSP00000445052	0	0.645	0	0.645
CTSK	CTSL	9606.ENSP00000271651	9606.ENSP00000345344	0.065	0.603	0.5	0.798
CTSL	MMP7	9606.ENSP00000345344	9606.ENSP00000260227	0	0	0.5	0.499
CTSL	MMP13	9606.ENSP00000345344	9606.ENSP00000260302	0	0	0.5	0.499
CTSL	MMP3	9606.ENSP00000345344	9606.ENSP00000299855	0.076	0	0.5	0.518
CTSL	RXRA	9606.ENSP00000345344	9606.ENSP00000419692	0	0.471	0	0.471
CTSL	MMP12	9606.ENSP00000345344	9606.ENSP00000458585	0.065	0	0.5	0.512
CTSL	MMP9	9606.ENSP00000345344	9606.ENSP00000361405	0.062	0	0.5	0.51
CTSL	CTSS	9606.ENSP00000345344	9606.ENSP00000357981	0.063	0	0.8	0.804
CTSL	CTSV	9606.ENSP00000345344	9606.ENSP00000445052	0	0.644	0.5	0.814
CTSS	TLR2	9606.ENSP00000357981	9606.ENSP00000260010	0.412	0	0	0.412
CTSS	TLR8	9606.ENSP00000357981	9606.ENSP00000312082	0.531	0	0	0.531
CTSS	MPEG1	9606.ENSP00000357981	9606.ENSP00000354335	0.748	0	0	0.748
CTSS	HCK	9606.ENSP00000357981	9606.ENSP00000444986	0.43	0.059	0	0.441
CTSS	PTPRC	9606.ENSP00000357981	9606.ENSP00000411355	0.706	0	0	0.706
CTSS	ITGB2	9606.ENSP00000357981	9606.ENSP00000380948	0.751	0	0	0.751
CX3CR1	CXCL8	9606.ENSP00000351059	9606.ENSP00000306512	0	0	0.6	0.6
CX3CR1	GNAI1	9606.ENSP00000351059	9606.ENSP00000343027	0	0	0.6	0.6
CX3CR1	GNAI3	9606.ENSP00000351059	9606.ENSP00000358867	0	0	0.6	0.6
CX3CR1	JAK3	9606.ENSP00000351059	9606.ENSP00000391676	0.05	0	0.6	0.603
CX3CR1	JAK2	9606.ENSP00000351059	9606.ENSP00000371067	0.061	0	0.6	0.608
CX3CR1	CXCL12	9606.ENSP00000351059	9606.ENSP00000379140	0	0	0.6	0.6

CXCL12	MAPK1	9606.ENSP00000379140	9606.ENSP00000215832	0	0	0.9	0.9
CXCL12	RAF1	9606.ENSP00000379140	9606.ENSP00000251849	0	0	0.9	0.9
CXCL12	GNAO1	9606.ENSP00000379140	9606.ENSP00000262494	0	0	0.9	0.9
CXCL12	MAPK3	9606.ENSP00000379140	9606.ENSP00000263025	0	0	0.9	0.9
CXCL12	PIK3CA	9606.ENSP00000379140	9606.ENSP00000263967	0	0	0.9	0.9
CXCL12	STAT3	9606.ENSP00000379140	9606.ENSP00000264657	0	0	0.9	0.9
CXCL12	HTR2C	9606.ENSP00000379140	9606.ENSP00000276198	0	0.076	0.9	0.903
CXCL12	PIK3CB	9606.ENSP00000379140	9606.ENSP00000289153	0	0	0.9	0.9
CXCL12	PTGER1	9606.ENSP00000379140	9606.ENSP00000292513	0	0.076	0.9	0.903
CXCL12	MAP2K1	9606.ENSP00000379140	9606.ENSP00000302486	0	0	0.9	0.9
CXCL12	F2	9606.ENSP00000379140	9606.ENSP00000308541	0	0	0.9	0.9
CXCL12	CXCR2	9606.ENSP00000379140	9606.ENSP00000319635	0	0.076	0.6	0.614
CXCL12	LCK	9606.ENSP00000379140	9606.ENSP00000337825	0	0	0.9	0.9
CXCL12	PTPN11	9606.ENSP00000379140	9606.ENSP00000340944	0	0	0.9	0.9
CXCL12	PTK2	9606.ENSP00000379140	9606.ENSP00000341189	0	0	0.9	0.9
CXCL12	GNAI1	9606.ENSP00000379140	9606.ENSP00000343027	0	0	0.9	0.9
CXCL12	RAC1	9606.ENSP00000379140	9606.ENSP00000348461	0	0	0.9	0.9
CXCL12	PIK3CG	9606.ENSP00000379140	9606.ENSP00000352121	0	0	0.9	0.9
CXCL12	DPP4	9606.ENSP00000379140	9606.ENSP00000353731	0	0.679	0	0.679
CXCL12	GNAI3	9606.ENSP00000379140	9606.ENSP00000358867	0	0	0.9	0.9
CXCL12	DNM1	9606.ENSP00000379140	9606.ENSP00000362014	0	0	0.9	0.9
CXCL12	SRC	9606.ENSP00000379140	9606.ENSP00000362680	0	0	0.9	0.9
CXCL12	CXCR3	9606.ENSP00000379140	9606.ENSP00000362795	0	0.076	0.6	0.614
CXCL12	FGR	9606.ENSP00000379140	9606.ENSP00000363117	0	0	0.9	0.9
CXCL12	JAK2	9606.ENSP00000379140	9606.ENSP00000371067	0	0	0.9	0.9
CXCL12	PTPN6	9606.ENSP00000379140	9606.ENSP00000391592	0	0	0.9	0.9
CXCL12	PIK3R1	9606.ENSP00000379140	9606.ENSP00000428056	0	0	0.9	0.9
CXCL12	HCK	9606.ENSP00000379140	9606.ENSP00000444986	0	0	0.9	0.9
CXCL12	PPARA	9606.ENSP00000379140	9606.ENSP00000385523	0	0	0.9	0.9
CXCL12	ESR1	9606.ENSP00000379140	9606.ENSP00000405330	0	0	0.9	0.9
CXCL12	PTPRC	9606.ENSP00000379140	9606.ENSP00000411355	0	0	0.9	0.9
CXCL8	NFKB1	9606.ENSP00000306512	9606.ENSP00000226574	0.118	0	0.9	0.908
CXCL8	MAPK14	9606.ENSP00000306512	9606.ENSP00000229795	0	0	0.9	0.9
CXCL8	EP300	9606.ENSP00000306512	9606.ENSP00000263253	0	0.27	0.9	0.923
CXCL8	STAT3	9606.ENSP00000306512	9606.ENSP00000264657	0.053	0	0.65	0.654
CXCL8	GRM5	9606.ENSP00000306512	9606.ENSP00000306138	0	0	0.6	0.6
CXCL8	GRM4	9606.ENSP00000306512	9606.ENSP00000440556	0	0	0.6	0.6
CXCL8	GRM2	9606.ENSP00000306512	9606.ENSP00000378492	0	0	0.6	0.6
CXCL8	LPAR6	9606.ENSP00000306512	9606.ENSP00000367691	0	0	0.6	0.6
CXCL8	GRM1	9606.ENSP00000306512	9606.ENSP00000354896	0	0	0.6	0.6
CXCL8	LPAR4	9606.ENSP00000306512	9606.ENSP00000408205	0	0	0.6	0.6
CXCL8	PTGFR	9606.ENSP00000306512	9606.ENSP00000359793	0	0	0.6	0.6
CXCL8	GNAI1	9606.ENSP00000306512	9606.ENSP00000343027	0	0.8	0	0.8
CXCL8	F2R	9606.ENSP00000306512	9606.ENSP00000321326	0	0	0.6	0.6

CXCL8	CXCR3	9606.ENSP00000306512	9606.ENSP00000362795	0	0	0.6	0.6
CXCL8	DNM1	9606.ENSP00000306512	9606.ENSP00000362014	0.062	0	0.9	0.902
CXCL8	FGR	9606.ENSP00000306512	9606.ENSP00000363117	0.076	0	0.9	0.903
CXCL8	LPAR2	9606.ENSP00000306512	9606.ENSP00000443256	0	0	0.9	0.9
CXCL8	LPAR3	9606.ENSP00000306512	9606.ENSP00000395389	0	0	0.9	0.9
CXCL8	HCK	9606.ENSP00000306512	9606.ENSP00000444986	0.117	0	0.9	0.907
CXCL8	LPAR1	9606.ENSP00000306512	9606.ENSP00000363553	0	0	0.9	0.9
CXCL8	LYN	9606.ENSP00000306512	9606.ENSP00000428924	0.076	0	0.9	0.903
CXCL8	PTGS2	9606.ENSP00000306512	9606.ENSP00000356438	0.753	0	0	0.753
CXCL8	JUN	9606.ENSP00000306512	9606.ENSP00000360266	0.095	0.27	0.8	0.856
CXCL8	RELA	9606.ENSP00000306512	9606.ENSP00000384273	0.065	0.753	0.9	0.974
CXCL8	TNF	9606.ENSP00000306512	9606.ENSP00000398698	0.158	0	0.9	0.912
CXCL8	CXCR2	9606.ENSP00000306512	9606.ENSP00000319635	0.065	0.974	0.9	0.997
CXCR2	F2	9606.ENSP00000319635	9606.ENSP00000308541	0	0.097	0.6	0.623
CXCR2	JAK3	9606.ENSP00000319635	9606.ENSP00000391676	0.077	0.056	0.6	0.621
CXCR2	GNAI3	9606.ENSP00000319635	9606.ENSP00000358867	0	0.303	0.6	0.709
CXCR2	JAK2	9606.ENSP00000319635	9606.ENSP00000371067	0	0.056	0.6	0.606
CXCR2	DNM1	9606.ENSP00000319635	9606.ENSP00000362014	0	0.056	0.9	0.901
CXCR2	GNAI1	9606.ENSP00000319635	9606.ENSP00000343027	0	0.822	0.6	0.926
CXCR3	GNAO1	9606.ENSP00000362795	9606.ENSP00000262494	0	0.151	0.9	0.911
CXCR3	GNAI1	9606.ENSP00000362795	9606.ENSP00000343027	0	0.151	0.9	0.911
CXCR3	GNAI3	9606.ENSP00000362795	9606.ENSP00000358867	0	0.151	0.9	0.911
CXCR3	DNM1	9606.ENSP00000362795	9606.ENSP00000362014	0	0.056	0.9	0.901
CXCR3	JAK2	9606.ENSP00000362795	9606.ENSP00000371067	0	0.056	0.6	0.606
CXCR3	JAK3	9606.ENSP00000362795	9606.ENSP00000391676	0.093	0.056	0.6	0.627
CYP11B1	HSD17B2	9606.ENSP00000292427	9606.ENSP00000199936	0	0.058	0.9	0.901
CYP11B1	SRD5A1	9606.ENSP00000292427	9606.ENSP00000274192	0	0	0.9	0.9
CYP11B1	CYP11B2	9606.ENSP00000292427	9606.ENSP00000325822	0.181	0.213	0.9	0.929
CYP11B1	CYP19A1	9606.ENSP00000292427	9606.ENSP00000379683	0.063	0.134	0.9	0.911
CYP11B1	SRD5A2	9606.ENSP00000292427	9606.ENSP00000477587	0	0	0.9	0.9
CYP11B1	CYP17A1	9606.ENSP00000292427	9606.ENSP00000358903	0.062	0.114	0.9	0.909
CYP11B1	HSD11B1	9606.ENSP00000292427	9606.ENSP00000355995	0	0.056	0.9	0.901
CYP11B1	HSD17B3	9606.ENSP00000292427	9606.ENSP00000364412	0	0	0.9	0.9
CYP11B1	HSD11B2	9606.ENSP00000292427	9606.ENSP00000316786	0	0.058	0.9	0.901
CYP11B2	HSD17B2	9606.ENSP00000325822	9606.ENSP00000199936	0	0.058	0.9	0.901
CYP11B2	SRD5A1	9606.ENSP00000325822	9606.ENSP00000274192	0	0	0.9	0.9
CYP11B2	HSD11B2	9606.ENSP00000325822	9606.ENSP00000316786	0	0.058	0.9	0.901
CYP11B2	SRD5A2	9606.ENSP00000325822	9606.ENSP00000477587	0	0	0.9	0.9
CYP11B2	CYP19A1	9606.ENSP00000325822	9606.ENSP00000379683	0.063	0.134	0.9	0.911
CYP11B2	HSD17B3	9606.ENSP00000325822	9606.ENSP00000364412	0	0	0.9	0.9
CYP11B2	HSD11B1	9606.ENSP00000325822	9606.ENSP00000355995	0	0.056	0.9	0.901
CYP11B2	CYP17A1	9606.ENSP00000325822	9606.ENSP00000358903	0.062	0.114	0.9	0.909
CYP17A1	HSD17B2	9606.ENSP00000358903	9606.ENSP00000199936	0.066	0.058	0.9	0.904
CYP17A1	STS	9606.ENSP00000358903	9606.ENSP00000217961	0.063	0	0.9	0.902

CYP17A1	SRD5A1	9606.ENSP00000358903	9606.ENSP00000274192	0.062	0	0.9	0.902
CYP17A1	CYP3A4	9606.ENSP00000358903	9606.ENSP00000337915	0.063	0.077	0.9	0.905
CYP17A1	CYP1A2	9606.ENSP00000358903	9606.ENSP00000342007	0.062	0	0.9	0.902
CYP17A1	CYP19A1	9606.ENSP00000358903	9606.ENSP00000379683	0	0.09	0.9	0.905
CYP17A1	SRD5A2	9606.ENSP00000358903	9606.ENSP00000477587	0.062	0	0.9	0.902
CYP17A1	HSD17B3	9606.ENSP00000358903	9606.ENSP00000364412	0.062	0	0.9	0.902
CYP19A1	HSD17B2	9606.ENSP00000379683	9606.ENSP00000199936	0	0.072	0.9	0.903
CYP19A1	SRD5A1	9606.ENSP00000379683	9606.ENSP00000274192	0	0	0.9	0.9
CYP19A1	CYP2A6	9606.ENSP00000379683	9606.ENSP00000301141	0.052	0.09	0.9	0.906
CYP19A1	UGT2B7	9606.ENSP00000379683	9606.ENSP00000304811	0	0.058	0.65	0.656
CYP19A1	CYP3A4	9606.ENSP00000379683	9606.ENSP00000337915	0	0.139	0.9	0.91
CYP19A1	HSD17B3	9606.ENSP00000379683	9606.ENSP00000364412	0.052	0	0.9	0.901
CYP19A1	SRD5A2	9606.ENSP00000379683	9606.ENSP00000477587	0.049	0	0.9	0.9
CYP19A1	HSD17B1	9606.ENSP00000379683	9606.ENSP00000466799	0.064	0.056	0.9	0.903
CYP1A2	STS	9606.ENSP00000342007	9606.ENSP00000217961	0	0	0.9	0.9
CYP1A2	PLA2G2C	9606.ENSP00000342007	9606.ENSP00000247992	0.069	0	0.65	0.66
CYP1A2	CYP2C9	9606.ENSP00000342007	9606.ENSP00000260682	0.077	0	0.9	0.903
CYP1A2	HPGDS	9606.ENSP00000342007	9606.ENSP00000295256	0	0.057	0.65	0.655
CYP1A2	CYP2A6	9606.ENSP00000342007	9606.ENSP00000301141	0.106	0	0.9	0.906
CYP1A2	UGT2B7	9606.ENSP00000342007	9606.ENSP00000304811	0.062	0.058	0.65	0.663
CYP1A2	PLA2G1B	9606.ENSP00000342007	9606.ENSP00000312286	0	0	0.65	0.65
CYP1A2	PLA2G6	9606.ENSP00000342007	9606.ENSP00000333142	0	0	0.65	0.65
CYP1A2	CYP3A4	9606.ENSP00000342007	9606.ENSP00000337915	0.139	0.077	0.9	0.913
CYP1A2	PLA2G10	9606.ENSP00000342007	9606.ENSP00000393847	0	0	0.65	0.65
CYP1A2	PLA2G4B	9606.ENSP00000342007	9606.ENSP00000396045	0	0	0.65	0.65
CYP1A2	PLA2G5	9606.ENSP00000342007	9606.ENSP00000364249	0	0	0.65	0.65
CYP1A2	PLA2G4A	9606.ENSP00000342007	9606.ENSP00000356436	0	0	0.65	0.65
CYP1A2	PLA2G2A	9606.ENSP00000342007	9606.ENSP00000383364	0	0	0.65	0.65
CYP1A2	IDO1	9606.ENSP00000342007	9606.ENSP00000430950	0	0	0.9	0.9
CYP1A2	CYP2C19	9606.ENSP00000342007	9606.ENSP00000360372	0	0	0.9	0.9
CYP1A2	EPHX1	9606.ENSP00000342007	9606.ENSP00000480004	0	0.091	0.9	0.905
CYP1A2	NR1I3	9606.ENSP00000342007	9606.ENSP00000356959	0.076	0.073	0.9	0.906
CYP2A6	CYP2C9	9606.ENSP00000301141	9606.ENSP00000260682	0.108	0.111	0.9	0.913
CYP2A6	HPGDS	9606.ENSP00000301141	9606.ENSP00000295256	0	0.057	0.65	0.655
CYP2A6	UGT2B7	9606.ENSP00000301141	9606.ENSP00000304811	0.084	0.058	0.65	0.671
CYP2A6	HSD17B1	9606.ENSP00000301141	9606.ENSP00000466799	0	0.066	0.9	0.902
CYP2A6	TYMP	9606.ENSP00000301141	9606.ENSP00000379038	0	0	0.9	0.9
CYP2A6	HSD11B1	9606.ENSP00000301141	9606.ENSP00000355995	0.112	0.101	0.9	0.913
CYP2A6	CYP2D6	9606.ENSP00000301141	9606.ENSP00000353820	0.107	0	0.9	0.906
CYP2A6	EPHX1	9606.ENSP00000301141	9606.ENSP00000480004	0.062	0.104	0.9	0.908
CYP2A6	CYP3A4	9606.ENSP00000301141	9606.ENSP00000337915	0.14	0.142	0.9	0.919
CYP2C19	PLA2G2C	9606.ENSP00000360372	9606.ENSP00000247992	0	0	0.65	0.65
CYP2C19	CYP2C9	9606.ENSP00000360372	9606.ENSP00000260682	0.322	0.229	0.9	0.943
CYP2C19	HPGDS	9606.ENSP00000360372	9606.ENSP00000295256	0	0.057	0.65	0.655

CYP2C19	PLA2G1B	9606.ENSP00000360372	9606.ENSP00000312286	0.052	0	0.65	0.654
CYP2C19	PLA2G6	9606.ENSP00000360372	9606.ENSP00000333142	0	0	0.65	0.65
CYP2C19	CYP3A4	9606.ENSP00000360372	9606.ENSP00000337915	0.178	0.142	0.9	0.923
CYP2C19	MAOA	9606.ENSP00000360372	9606.ENSP00000340684	0.063	0	0.9	0.902
CYP2C19	CYP2D6	9606.ENSP00000360372	9606.ENSP00000353820	0.051	0	0.9	0.901
CYP2C19	PTGS1	9606.ENSP00000360372	9606.ENSP00000354612	0.053	0	0.9	0.901
CYP2C19	PLA2G4A	9606.ENSP00000360372	9606.ENSP00000356436	0	0	0.65	0.65
CYP2C19	PTGS2	9606.ENSP00000360372	9606.ENSP00000356438	0.053	0	0.9	0.901
CYP2C19	PLA2G10	9606.ENSP00000360372	9606.ENSP00000393847	0	0	0.65	0.65
CYP2C19	PLA2G4B	9606.ENSP00000360372	9606.ENSP00000396045	0	0	0.65	0.65
CYP2C19	PLA2G2A	9606.ENSP00000360372	9606.ENSP00000383364	0.069	0	0.65	0.66
CYP2C19	PLA2G5	9606.ENSP00000360372	9606.ENSP00000364249	0	0	0.65	0.65
CYP2C19	MAOB	9606.ENSP00000360372	9606.ENSP00000367309	0.063	0	0.9	0.902
CYP2C19	EPHX1	9606.ENSP00000360372	9606.ENSP00000480004	0.054	0.104	0.9	0.907
CYP2C9	PLA2G2C	9606.ENSP00000260682	9606.ENSP00000247992	0	0	0.65	0.65
CYP2C9	PLA2G10	9606.ENSP00000260682	9606.ENSP00000393847	0	0	0.65	0.65
CYP2C9	PLA2G4B	9606.ENSP00000260682	9606.ENSP00000396045	0	0	0.65	0.65
CYP2C9	PLA2G6	9606.ENSP00000260682	9606.ENSP00000333142	0	0	0.65	0.65
CYP2C9	PLA2G5	9606.ENSP00000260682	9606.ENSP00000364249	0	0	0.65	0.65
CYP2C9	PLA2G4A	9606.ENSP00000260682	9606.ENSP00000356436	0	0	0.65	0.65
CYP2C9	PLA2G2A	9606.ENSP00000260682	9606.ENSP00000383364	0.063	0	0.65	0.658
CYP2C9	PLA2G1B	9606.ENSP00000260682	9606.ENSP00000312286	0.052	0	0.65	0.654
CYP2C9	HPGDS	9606.ENSP00000260682	9606.ENSP00000295256	0	0.057	0.65	0.655
CYP2C9	UGT2B7	9606.ENSP00000260682	9606.ENSP00000304811	0.1	0.058	0.65	0.677
CYP2C9	PTGS1	9606.ENSP00000260682	9606.ENSP00000354612	0.053	0	0.9	0.901
CYP2C9	PTGS2	9606.ENSP00000260682	9606.ENSP00000356438	0.053	0	0.9	0.901
CYP2C9	EPHX2	9606.ENSP00000260682	9606.ENSP00000430269	0.049	0.069	0.9	0.903
CYP2C9	EPHX1	9606.ENSP00000260682	9606.ENSP00000480004	0.054	0.104	0.9	0.907
CYP2C9	CYP3A4	9606.ENSP00000260682	9606.ENSP00000337915	0.247	0.346	0.9	0.946
CYP2D6	UGT2B7	9606.ENSP00000353820	9606.ENSP00000304811	0.068	0.058	0.65	0.665
CYP2D6	CYP3A4	9606.ENSP00000353820	9606.ENSP00000337915	0.09	0.11	0.9	0.911
CYP2D6	MAOA	9606.ENSP00000353820	9606.ENSP00000340684	0.063	0	0.9	0.902
CYP2D6	HSD11B1	9606.ENSP00000353820	9606.ENSP00000355995	0.062	0.173	0.9	0.915
CYP2D6	MAOB	9606.ENSP00000353820	9606.ENSP00000367309	0.063	0	0.9	0.902
CYP3A4	HSD17B2	9606.ENSP00000337915	9606.ENSP00000199936	0.07	0.058	0.9	0.904
CYP3A4	STS	9606.ENSP00000337915	9606.ENSP00000217961	0.049	0	0.9	0.9
CYP3A4	PLA2G2C	9606.ENSP00000337915	9606.ENSP00000247992	0	0	0.65	0.65
CYP3A4	HPGDS	9606.ENSP00000337915	9606.ENSP00000295256	0	0.056	0.65	0.655
CYP3A4	UGT2B7	9606.ENSP00000337915	9606.ENSP00000304811	0.144	0.688	0.65	0.898
CYP3A4	PLA2G1B	9606.ENSP00000337915	9606.ENSP00000312286	0.063	0	0.65	0.658
CYP3A4	PLA2G6	9606.ENSP00000337915	9606.ENSP00000333142	0	0	0.65	0.65
CYP3A4	PLA2G5	9606.ENSP00000337915	9606.ENSP00000364249	0	0	0.65	0.65
CYP3A4	PLA2G10	9606.ENSP00000337915	9606.ENSP00000393847	0	0	0.65	0.65
CYP3A4	PLA2G4B	9606.ENSP00000337915	9606.ENSP00000396045	0	0	0.65	0.65

CYP3A4	PLA2G2A	9606.ENSP00000337915	9606.ENSP00000383364	0.069	0	0.65	0.66
CYP3A4	PLA2G4A	9606.ENSP00000337915	9606.ENSP00000356436	0	0	0.65	0.65
CYP3A4	MAOB	9606.ENSP00000337915	9606.ENSP00000367309	0.064	0	0.9	0.902
CYP3A4	HSD17B1	9606.ENSP00000337915	9606.ENSP00000466799	0.063	0.056	0.9	0.903
CYP3A4	HSD11B1	9606.ENSP00000337915	9606.ENSP00000355995	0.111	0.056	0.9	0.908
CYP3A4	MAOA	9606.ENSP00000337915	9606.ENSP00000340684	0.064	0	0.9	0.902
CYP3A4	EPHX1	9606.ENSP00000337915	9606.ENSP00000480004	0.062	0.069	0.9	0.905
CYP51A1	DHCR7	9606.ENSP00000003100	9606.ENSP00000347717	0.543	0.297	0	0.665
CYP51A1	SIGMAR1	9606.ENSP00000003100	9606.ENSP00000277010	0.108	0.781	0	0.796
CYP51A1	FDPS	9606.ENSP00000003100	9606.ENSP00000349078	0.681	0	0	0.681
CYP51A1	SREBF2	9606.ENSP00000003100	9606.ENSP00000354476	0.064	0	0.9	0.902
CYP51A1	HMGCR	9606.ENSP00000003100	9606.ENSP00000287936	0.862	0	0	0.862
CYP51A1	SQLE	9606.ENSP00000003100	9606.ENSP00000265896	0.928	0.168	0	0.937
CYP51A1	FDFT1	9606.ENSP00000003100	9606.ENSP00000480828	0.809	0.781	0	0.956
CYP51A1	LSS	9606.ENSP00000003100	9606.ENSP00000380837	0.823	0.781	0.9	0.995
DBF4	SPTLC2	9606.ENSP00000265728	9606.ENSP00000216484	0.422	0	0	0.422
DBF4	TTK	9606.ENSP00000265728	9606.ENSP00000358813	0.582	0	0	0.582
DBF4	PLK1	9606.ENSP00000265728	9606.ENSP00000300093	0.202	0.527	0	0.606
DBF4	POLA1	9606.ENSP00000265728	9606.ENSP00000368349	0.132	0.104	0.9	0.915
DDR1	FLT4	9606.ENSP00000427552	9606.ENSP00000261937	0	0.4	0	0.4
DDR1	TXK	9606.ENSP00000427552	9606.ENSP00000264316	0	0.4	0	0.4
DDR1	PTPN11	9606.ENSP00000427552	9606.ENSP00000340944	0.062	0.486	0	0.497
DDR1	EPHB4	9606.ENSP00000427552	9606.ENSP00000350896	0.062	0.392	0	0.405
DDR2	PDGFRB	9606.ENSP00000356899	9606.ENSP00000261799	0.341	0.15	0	0.416
DDR2	SRC	9606.ENSP00000356899	9606.ENSP00000362680	0	0.473	0	0.473
DHCR7	STS	9606.ENSP00000347717	9606.ENSP00000217961	0	0	0.8	0.8
DHCR7	SQLE	9606.ENSP00000347717	9606.ENSP00000265896	0.478	0.498	0	0.726
DHCR7	SIGMAR1	9606.ENSP00000347717	9606.ENSP00000277010	0.214	0.641	0	0.706
DHCR7	LIPA	9606.ENSP00000347717	9606.ENSP00000337354	0.062	0.061	0.9	0.904
DHCR7	SOAT1	9606.ENSP00000347717	9606.ENSP00000356591	0	0.076	0.9	0.903
DHCR7	SREBF2	9606.ENSP00000347717	9606.ENSP00000354476	0.125	0	0.9	0.908
DHCR7	LSS	9606.ENSP00000347717	9606.ENSP00000380837	0.845	0	0	0.845
DHFR	KIF11	9606.ENSP00000396308	9606.ENSP00000260731	0.42	0	0	0.42
DHFR	QDPR	9606.ENSP00000396308	9606.ENSP00000281243	0.062	0	0.9	0.902
DHFR	TYMS	9606.ENSP00000396308	9606.ENSP00000315644	0.467	0.081	0.9	0.946
DHFR	POLA1	9606.ENSP00000396308	9606.ENSP00000368349	0.536	0	0	0.536
DHFR	PCNA	9606.ENSP00000396308	9606.ENSP00000368458	0.458	0	0	0.458
DHODH	NDUFS8	9606.ENSP00000219240	9606.ENSP00000315774	0	0.645	0	0.645
DNM1	MAPK1	9606.ENSP00000362014	9606.ENSP00000215832	0.062	0	0.9	0.902
DNM1	RAF1	9606.ENSP00000362014	9606.ENSP00000251849	0.062	0.059	0.9	0.904
DNM1	MAPK3	9606.ENSP00000362014	9606.ENSP00000263025	0.062	0	0.9	0.902
DNM1	GABRA6	9606.ENSP00000362014	9606.ENSP00000274545	0.192	0	0.9	0.915
DNM1	EGFR	9606.ENSP00000362014	9606.ENSP00000275493	0.052	0	0.9	0.901
DNM1	LRRK2	9606.ENSP00000362014	9606.ENSP00000298910	0.077	0.495	0	0.514

DNM1	MAP2K1	9606.ENSP00000362014	9606.ENSP00000302486	0.058	0	0.9	0.901
DNM1	F2	9606.ENSP00000362014	9606.ENSP00000308541	0	0	0.9	0.9
DNM1	GAK	9606.ENSP00000362014	9606.ENSP00000314499	0.138	0.603	0	0.643
DNM1	F2R	9606.ENSP00000362014	9606.ENSP00000321326	0	0	0.9	0.9
DNM1	EPHB3	9606.ENSP00000362014	9606.ENSP00000332118	0.062	0.059	0.6	0.616
DNM1	GABRA5	9606.ENSP00000362014	9606.ENSP00000335592	0.159	0	0.9	0.912
DNM1	EPHB4	9606.ENSP00000362014	9606.ENSP00000350896	0.062	0.059	0.6	0.616
DNM1	GABRA3	9606.ENSP00000362014	9606.ENSP00000359337	0.1	0	0.9	0.906
DNM1	GRIN1	9606.ENSP00000362014	9606.ENSP00000360608	0.263	0.403	0	0.542
DNM1	GRIN2B	9606.ENSP00000362014	9606.ENSP00000477455	0.13	0.368	0	0.426
DNM1	DYRK1A	9606.ENSP00000362014	9606.ENSP00000381932	0.063	0.559	0	0.57
DNM1	TBXA2R	9606.ENSP00000362014	9606.ENSP00000393333	0	0.056	0.9	0.901
DNM1	FGR	9606.ENSP00000362014	9606.ENSP00000363117	0	0.059	0.9	0.901
DNM1	HCK	9606.ENSP00000362014	9606.ENSP00000444986	0	0.059	0.9	0.901
DNM1	GABRA2	9606.ENSP00000362014	9606.ENSP00000421828	0.137	0	0.9	0.91
DNM1	NTRK1	9606.ENSP00000362014	9606.ENSP00000431418	0	0.08	0.9	0.904
DNM1	GABRA1	9606.ENSP00000362014	9606.ENSP00000393097	0.2	0	0.9	0.916
DNM1	SRC	9606.ENSP00000362014	9606.ENSP00000362680	0.064	0.703	0.9	0.969
DNMT1	STAT3	9606.ENSP00000352516	9606.ENSP00000264657	0	0.494	0	0.494
DNMT1	DNMT3A	9606.ENSP00000352516	9606.ENSP00000264709	0.049	0.696	0.9	0.968
DNMT1	EZH2	9606.ENSP00000352516	9606.ENSP00000320147	0.179	0.848	0.6	0.946
DNMT1	PARP1	9606.ENSP00000352516	9606.ENSP00000355759	0.231	0.501	0	0.6
DNMT1	KDM1A	9606.ENSP00000352516	9606.ENSP00000383042	0.121	0.675	0	0.702
DNMT1	PCNA	9606.ENSP00000352516	9606.ENSP00000368458	0.355	0.975	0	0.983
DNMT1	HDAC2	9606.ENSP00000352516	9606.ENSP00000430432	0.062	0.846	0.8	0.968
DNMT1	HDAC1	9606.ENSP00000352516	9606.ENSP00000362649	0.188	0.903	0.9	0.991
DNMT3A	PBRM1	9606.ENSP00000264709	9606.ENSP00000378307	0.067	0	0.65	0.659
DNMT3A	SMARCA2	9606.ENSP00000264709	9606.ENSP00000265773	0	0.056	0.65	0.655
DNMT3A	SMARCA4	9606.ENSP00000264709	9606.ENSP00000395654	0.058	0.056	0.65	0.661
DNMT3A	HDAC1	9606.ENSP00000264709	9606.ENSP00000362649	0.062	0.495	0	0.506
DNMT3A	EZH2	9606.ENSP00000264709	9606.ENSP00000320147	0	0.501	0.6	0.792
DPP4	FAP	9606.ENSP00000353731	9606.ENSP00000188790	0.076	0.462	0	0.481
DPP4	PRCP	9606.ENSP00000353731	9606.ENSP00000377055	0.062	0	0.8	0.804
DPP4	MME	9606.ENSP00000353731	9606.ENSP00000418525	0.076	0	0.8	0.807
DPP9	SAE1	9606.ENSP00000262960	9606.ENSP00000270225	0.062	0.458	0	0.47
DRD1	HTR5A	9606.ENSP00000377353	9606.ENSP00000287907	0.519	0	0	0.519
DRD1	HRH3	9606.ENSP00000377353	9606.ENSP00000342560	0.184	0.312	0	0.414
DRD1	GRIN1	9606.ENSP00000377353	9606.ENSP00000360608	0.241	0.467	0	0.578
DRD2	SLC6A3	9606.ENSP00000354859	9606.ENSP00000270349	0.062	0.428	0.8	0.883
DRD2	GNAI1	9606.ENSP00000354859	9606.ENSP00000343027	0.055	0.39	0.8	0.874
DRD2	GNAI3	9606.ENSP00000354859	9606.ENSP00000358867	0.055	0.303	0.8	0.856
DRD3	DRD4	9606.ENSP00000373169	9606.ENSP00000176183	0	0	0.8	0.8
DRD3	GNAI1	9606.ENSP00000373169	9606.ENSP00000343027	0	0.303	0.6	0.709
DRD3	GNAI3	9606.ENSP00000373169	9606.ENSP00000358867	0	0.151	0.6	0.645

DRD4	GNAI3	9606.ENSP00000176183	9606.ENSP00000358867	0	0.151	0.6	0.645
DRD4	GNAI1	9606.ENSP00000176183	9606.ENSP00000343027	0	0.39	0.6	0.745
DRD5	HTR5A	9606.ENSP00000306129	9606.ENSP00000287907	0.452	0	0	0.452
DUSP3	MAPK1	9606.ENSP00000226004	9606.ENSP00000215832	0.045	0.595	0.9	0.958
DUSP3	MAPK10	9606.ENSP00000226004	9606.ENSP00000352157	0.06	0.154	0.6	0.654
DUSP3	MAPK14	9606.ENSP00000226004	9606.ENSP00000229795	0.048	0.244	0.6	0.686
DUSP3	MAPK8	9606.ENSP00000226004	9606.ENSP00000378974	0	0.154	0.6	0.647
DUSP3	PTPN7	9606.ENSP00000226004	9606.ENSP00000309116	0.052	0.07	0.8	0.808
DUSP3	MAPK3	9606.ENSP00000226004	9606.ENSP00000263025	0	0.595	0.9	0.957
DUT	MAPK1	9606.ENSP00000370376	9606.ENSP00000215832	0	0	0.9	0.9
DUT	MAPK3	9606.ENSP00000370376	9606.ENSP00000263025	0.063	0	0.9	0.902
DUT	SAE1	9606.ENSP00000370376	9606.ENSP00000270225	0.842	0	0	0.842
DUT	PLK1	9606.ENSP00000370376	9606.ENSP00000300093	0.653	0	0	0.653
DUT	FEN1	9606.ENSP00000370376	9606.ENSP00000305480	0.777	0	0	0.777
DUT	TYMS	9606.ENSP00000370376	9606.ENSP00000315644	0.874	0.285	0.9	0.99
DUT	HSP90AA1	9606.ENSP00000370376	9606.ENSP00000335153	0.062	0	0.9	0.902
DUT	HSPA1A	9606.ENSP00000370376	9606.ENSP00000364802	0.063	0	0.9	0.902
DUT	POLA1	9606.ENSP00000370376	9606.ENSP00000368349	0.8	0.07	0	0.807
DUT	PCNA	9606.ENSP00000370376	9606.ENSP00000368458	0.942	0	0	0.942
DUT	PPARA	9606.ENSP00000370376	9606.ENSP00000385523	0.062	0.296	0.9	0.928
DYRK1A	FNTB	9606.ENSP00000381932	9606.ENSP00000246166	0.064	0.778	0	0.783
DYRK1A	EP300	9606.ENSP00000381932	9606.ENSP00000263253	0.086	0.539	0	0.56
DYRK1A	FNTA	9606.ENSP00000381932	9606.ENSP00000303423	0.052	0.708	0	0.712
DYRK1A	MAPT	9606.ENSP00000381932	9606.ENSP00000340820	0	0.488	0	0.487
DYRK1A	TGM2	9606.ENSP00000381932	9606.ENSP00000355330	0	0.486	0	0.485
DYRK2	MDM2	9606.ENSP00000342105	9606.ENSP00000258149	0	0.282	0.9	0.925
EDNRA	EGFR	9606.ENSP00000315011	9606.ENSP00000275493	0.161	0.061	0.9	0.914
EDNRA	F2	9606.ENSP00000315011	9606.ENSP00000308541	0	0.097	0.8	0.811
EDNRA	GNAI1	9606.ENSP00000315011	9606.ENSP00000343027	0.069	0.151	0.6	0.656
EDNRA	MCL1	9606.ENSP00000315011	9606.ENSP00000358022	0	0.684	0	0.684
EDNRA	GNAI3	9606.ENSP00000315011	9606.ENSP00000358867	0	0.151	0.6	0.645
EDNRA	RAC1	9606.ENSP00000315011	9606.ENSP00000348461	0	0.056	0.9	0.901
EDNRA	MAPK8	9606.ENSP00000315011	9606.ENSP00000378974	0	0.058	0.9	0.901
EDNRA	JAK2	9606.ENSP00000315011	9606.ENSP00000371067	0	0.056	0.9	0.901
EGFR	HDAC7	9606.ENSP00000275493	9606.ENSP00000080059	0	0.642	0	0.642
EGFR	TNFRSF1A	9606.ENSP00000275493	9606.ENSP00000162749	0.062	0.642	0	0.649
EGFR	MAPK1	9606.ENSP00000275493	9606.ENSP00000215832	0	0.44	0.9	0.941
EGFR	MAPK14	9606.ENSP00000275493	9606.ENSP00000229795	0	0.736	0	0.736
EGFR	NR3C1	9606.ENSP00000275493	9606.ENSP00000231509	0	0.664	0	0.664
EGFR	RAF1	9606.ENSP00000275493	9606.ENSP00000251849	0	0.687	0	0.687
EGFR	PDGFRA	9606.ENSP00000275493	9606.ENSP00000257290	0.11	0.68	0.8	0.938
EGFR	MDM2	9606.ENSP00000275493	9606.ENSP00000258149	0	0	0.6	0.6
EGFR	TLR2	9606.ENSP00000275493	9606.ENSP00000260010	0	0.71	0	0.71
EGFR	PDGFRB	9606.ENSP00000275493	9606.ENSP00000261799	0.105	0.541	0.8	0.91

EGFR	MAPK3	9606.ENSP00000275493	9606.ENSP00000263025	0	0.413	0.9	0.938
EGFR	PIK3CA	9606.ENSP00000275493	9606.ENSP00000263967	0	0.741	0.9	0.973
EGFR	STAT3	9606.ENSP00000275493	9606.ENSP00000264657	0	0.931	0.9	0.992
EGFR	IGF1R	9606.ENSP00000275493	9606.ENSP00000268035	0	0.665	0	0.665
EGFR	ERBB2	9606.ENSP00000275493	9606.ENSP00000269571	0.077	0.982	0.9	0.998
EGFR	SYK	9606.ENSP00000275493	9606.ENSP00000364907	0	0.403	0	0.403
EGFR	EPHB4	9606.ENSP00000275493	9606.ENSP00000350896	0.091	0.377	0	0.409
EGFR	PTPRF	9606.ENSP00000275493	9606.ENSP00000353030	0.213	0.355	0	0.471
EGFR	NR2F2	9606.ENSP00000275493	9606.ENSP00000377721	0.332	0.26	0	0.484
EGFR	INSR	9606.ENSP00000275493	9606.ENSP00000303830	0.062	0.107	0.5	0.544
EGFR	PIM1	9606.ENSP00000275493	9606.ENSP00000362608	0.048	0.642	0	0.644
EGFR	ITK	9606.ENSP00000275493	9606.ENSP00000398655	0	0.666	0	0.666
EGFR	PRKCE	9606.ENSP00000275493	9606.ENSP00000306124	0	0.613	0	0.613
EGFR	TYK2	9606.ENSP00000275493	9606.ENSP00000431885	0	0.182	0.6	0.658
EGFR	RGS4	9606.ENSP00000275493	9606.ENSP00000397181	0.075	0.691	0	0.701
EGFR	LRRK2	9606.ENSP00000275493	9606.ENSP00000298910	0	0.176	0.6	0.656
EGFR	PRKCZ	9606.ENSP00000275493	9606.ENSP00000367830	0	0.653	0	0.653
EGFR	ITGB2	9606.ENSP00000275493	9606.ENSP00000380948	0.051	0.649	0	0.653
EGFR	MAPK8	9606.ENSP00000275493	9606.ENSP00000378974	0	0.642	0	0.642
EGFR	MAPT	9606.ENSP00000275493	9606.ENSP00000340820	0.063	0.642	0	0.65
EGFR	IKBKG	9606.ENSP00000275493	9606.ENSP00000483825	0	0.642	0	0.642
EGFR	HSPA1A	9606.ENSP00000275493	9606.ENSP00000364802	0	0.677	0	0.677
EGFR	RET	9606.ENSP00000275493	9606.ENSP00000347942	0.063	0.723	0	0.729
EGFR	MMP3	9606.ENSP00000275493	9606.ENSP00000299855	0.076	0	0.65	0.662
EGFR	JAK3	9606.ENSP00000275493	9606.ENSP00000391676	0	0.166	0.8	0.826
EGFR	PIK3CD	9606.ENSP00000275493	9606.ENSP00000366563	0	0.058	0.8	0.803
EGFR	HDAC6	9606.ENSP00000275493	9606.ENSP00000334061	0	0.743	0	0.743
EGFR	LPAR2	9606.ENSP00000275493	9606.ENSP00000443256	0.049	0.061	0.9	0.902
EGFR	TNK2	9606.ENSP00000275493	9606.ENSP00000371341	0	0.878	0	0.878
EGFR	LCK	9606.ENSP00000275493	9606.ENSP00000337825	0	0.231	0.9	0.919
EGFR	PRKCB	9606.ENSP00000275493	9606.ENSP00000305355	0.049	0.282	0.9	0.925
EGFR	GNAI1	9606.ENSP00000275493	9606.ENSP00000343027	0.069	0.281	0.9	0.927
EGFR	LPAR1	9606.ENSP00000275493	9606.ENSP00000363553	0.111	0.061	0.9	0.909
EGFR	PIK3C2B	9606.ENSP00000275493	9606.ENSP00000356155	0	0.782	0	0.782
EGFR	LPAR3	9606.ENSP00000275493	9606.ENSP00000395389	0	0.061	0.9	0.902
EGFR	RAC1	9606.ENSP00000275493	9606.ENSP00000348461	0	0.115	0.9	0.907
EGFR	MET	9606.ENSP00000275493	9606.ENSP00000317272	0.23	0.887	0	0.909
EGFR	JAK1	9606.ENSP00000275493	9606.ENSP00000343204	0	0.32	0.9	0.929
EGFR	GNAI3	9606.ENSP00000275493	9606.ENSP00000358867	0	0.494	0.9	0.947
EGFR	PLAU	9606.ENSP00000275493	9606.ENSP00000361850	0.088	0	0.9	0.904
EGFR	PTPN6	9606.ENSP00000275493	9606.ENSP00000391592	0	0.79	0	0.79
EGFR	PIK3CB	9606.ENSP00000275493	9606.ENSP00000289153	0	0.326	0.9	0.929
EGFR	FGR	9606.ENSP00000275493	9606.ENSP00000363117	0	0.65	0.9	0.963
EGFR	PTK2	9606.ENSP00000275493	9606.ENSP00000341189	0.108	0.79	0.8	0.959

EGFR	JAK2	9606.ENSP00000275493	9606.ENSP00000371067	0	0.807	0.8	0.959
EGFR	HIF1A	9606.ENSP00000275493	9606.ENSP00000437955	0	0	0.9	0.9
EGFR	YES1	9606.ENSP00000275493	9606.ENSP00000462468	0.098	0.698	0.9	0.97
EGFR	FOS	9606.ENSP00000275493	9606.ENSP00000306245	0	0.406	0.9	0.938
EGFR	MTOR	9606.ENSP00000275493	9606.ENSP00000354558	0.062	0.197	0.9	0.918
EGFR	PTK2B	9606.ENSP00000275493	9606.ENSP00000380638	0.064	0.718	0.9	0.971
EGFR	PRKCA	9606.ENSP00000275493	9606.ENSP00000408695	0.049	0.751	0.9	0.974
EGFR	LYN	9606.ENSP00000275493	9606.ENSP00000428924	0	0.773	0.9	0.976
EGFR	PTPN2	9606.ENSP00000275493	9606.ENSP00000311857	0	0.714	0.9	0.97
EGFR	HCK	9606.ENSP00000275493	9606.ENSP00000444986	0	0.833	0.9	0.982
EGFR	SRC	9606.ENSP00000275493	9606.ENSP00000362680	0	0.931	0.9	0.992
EGFR	ESR1	9606.ENSP00000275493	9606.ENSP00000405330	0	0.696	0	0.696
EGFR	PIK3R1	9606.ENSP00000275493	9606.ENSP00000428056	0	0.893	0.9	0.988
EGFR	PTPN1	9606.ENSP00000275493	9606.ENSP00000360683	0	0.893	0.9	0.988
EGFR	PTPN11	9606.ENSP00000275493	9606.ENSP00000340944	0	0.941	0.9	0.993
EGFR	HSP90AA1	9606.ENSP00000275493	9606.ENSP00000335153	0	0.884	0.9	0.987
EGLN1	PYGL	9606.ENSP00000355601	9606.ENSP00000216392	0	0.4	0	0.4
EGLN1	VHL	9606.ENSP00000355601	9606.ENSP00000256474	0	0.685	0.6	0.868
EGLN1	PRKCG	9606.ENSP00000355601	9606.ENSP00000263431	0	0	0.8	0.8
EGLN1	EPAS1	9606.ENSP00000355601	9606.ENSP00000263734	0.065	0.906	0.9	0.99
EGLN1	PRKCB	9606.ENSP00000355601	9606.ENSP00000305355	0	0	0.8	0.8
EGLN1	HSP90AA1	9606.ENSP00000355601	9606.ENSP00000335153	0	0.486	0	0.485
EGLN1	PRKCA	9606.ENSP00000355601	9606.ENSP00000408695	0	0	0.8	0.8
EGLN1	HIF1A	9606.ENSP00000355601	9606.ENSP00000437955	0	0.935	0.9	0.993
EIF2AK1	IKBKG	9606.ENSP00000199389	9606.ENSP00000483825	0	0	0.6	0.6
EIF2AK1	IKBKB	9606.ENSP00000199389	9606.ENSP00000430684	0.062	0	0.6	0.608
EIF2AK1	HSP90AA1	9606.ENSP00000199389	9606.ENSP00000335153	0.061	0.411	0	0.423
EIF2AK1	NFE2L2	9606.ENSP00000199389	9606.ENSP00000380252	0	0	0.8	0.8
EIF4H	G6PD	9606.ENSP00000265753	9606.ENSP00000377192	0.086	0.426	0	0.454
ELANE	MPO	9606.ENSP00000466090	9606.ENSP00000225275	0.784	0.141	0	0.807
ELANE	MMP8	9606.ENSP00000466090	9606.ENSP00000236826	0.176	0	0.9	0.914
ELANE	MMP7	9606.ENSP00000466090	9606.ENSP00000260227	0	0	0.9	0.9
ELANE	F2	9606.ENSP00000466090	9606.ENSP00000308541	0.068	0	0.5	0.514
ELANE	MMP1	9606.ENSP00000466090	9606.ENSP00000322788	0.052	0	0.9	0.901
EP300	HDAC7	9606.ENSP00000263253	9606.ENSP00000080059	0.062	0.368	0.9	0.935
EP300	MAPK1	9606.ENSP00000263253	9606.ENSP00000215832	0	0.526	0	0.526
EP300	HDAC10	9606.ENSP00000263253	9606.ENSP00000216271	0.062	0.171	0.65	0.704
EP300	HDAC5	9606.ENSP00000263253	9606.ENSP00000225983	0.062	0.171	0.65	0.704
EP300	NFKB1	9606.ENSP00000263253	9606.ENSP00000226574	0	0.873	0.9	0.986
EP300	MAPK14	9606.ENSP00000263253	9606.ENSP00000229795	0	0.525	0.9	0.95
EP300	NR3C1	9606.ENSP00000263253	9606.ENSP00000231509	0.062	0.695	0.9	0.968
EP300	NR1D1	9606.ENSP00000263253	9606.ENSP00000246672	0.062	0	0.9	0.902
EP300	SIRT2	9606.ENSP00000263253	9606.ENSP00000249396	0.062	0.708	0	0.714
EP300	NR1H2	9606.ENSP00000263253	9606.ENSP00000253727	0.062	0.214	0.6	0.679

EP300	RARA	9606.ENSP00000263253	9606.ENSP00000254066	0	0.518	0.9	0.949
EP300	FABP4	9606.ENSP00000263253	9606.ENSP00000256104	0.062	0.082	0.9	0.906
EP300	VHL	9606.ENSP00000263253	9606.ENSP00000256474	0	0.835	0	0.835
EP300	MDM2	9606.ENSP00000263253	9606.ENSP00000258149	0	0.874	0.9	0.986
EP300	RORA	9606.ENSP00000263253	9606.ENSP00000261523	0	0.298	0.8	0.853
EP300	SPHK1	9606.ENSP00000263253	9606.ENSP00000313681	0.063	0.495	0	0.506
EP300	MTOR	9606.ENSP00000263253	9606.ENSP00000354558	0.065	0.393	0	0.408
EP300	RXRB	9606.ENSP00000263253	9606.ENSP00000363817	0	0.102	0.6	0.625
EP300	PRKDC	9606.ENSP00000263253	9606.ENSP00000313420	0.063	0.494	0	0.506
EP300	RXRG	9606.ENSP00000263253	9606.ENSP00000352900	0	0.102	0.6	0.625
EP300	SMARCA2	9606.ENSP00000263253	9606.ENSP00000265773	0.095	0.368	0	0.404
EP300	PTGS2	9606.ENSP00000263253	9606.ENSP00000356438	0	0.497	0	0.497
EP300	PRKCD	9606.ENSP00000263253	9606.ENSP00000378217	0	0.463	0	0.463
EP300	THRB	9606.ENSP00000263253	9606.ENSP00000379904	0	0.102	0.6	0.625
EP300	PRKCA	9606.ENSP00000263253	9606.ENSP00000408695	0.064	0.487	0	0.499
EP300	MAPT	9606.ENSP00000263253	9606.ENSP00000340820	0	0.675	0	0.675
EP300	THRA	9606.ENSP00000263253	9606.ENSP00000264637	0	0.316	0.6	0.714
EP300	PLK1	9606.ENSP00000263253	9606.ENSP00000300093	0.067	0.144	0.65	0.696
EP300	PPARD	9606.ENSP00000263253	9606.ENSP00000310928	0	0.701	0	0.701
EP300	PGR	9606.ENSP00000263253	9606.ENSP00000325120	0	0.316	0.6	0.714
EP300	ERCC5	9606.ENSP00000263253	9606.ENSP00000347978	0.063	0.057	0.8	0.807
EP300	FEN1	9606.ENSP00000263253	9606.ENSP00000305480	0	0.68	0	0.68
EP300	ESR2	9606.ENSP00000263253	9606.ENSP00000343925	0	0.673	0	0.673
EP300	HDAC8	9606.ENSP00000263253	9606.ENSP00000362674	0.063	0.171	0.65	0.704
EP300	HDAC11	9606.ENSP00000263253	9606.ENSP00000295757	0.062	0.171	0.65	0.704
EP300	HDAC4	9606.ENSP00000263253	9606.ENSP00000264606	0.062	0.171	0.65	0.704
EP300	PARP1	9606.ENSP00000263253	9606.ENSP00000355759	0	0.756	0	0.756
EP300	PLIN1	9606.ENSP00000263253	9606.ENSP00000300055	0	0	0.9	0.9
EP300	FABP1	9606.ENSP00000263253	9606.ENSP00000295834	0	0.082	0.9	0.904
EP300	LPL	9606.ENSP00000263253	9606.ENSP00000309757	0	0	0.9	0.9
EP300	PTGDR	9606.ENSP00000263253	9606.ENSP00000303424	0.049	0	0.9	0.9
EP300	HSPA1A	9606.ENSP00000263253	9606.ENSP00000364802	0	0.056	0.9	0.901
EP300	HDAC9	9606.ENSP00000263253	9606.ENSP00000408617	0.062	0.171	0.65	0.704
EP300	RARG	9606.ENSP00000263253	9606.ENSP00000388510	0	0.182	0.9	0.914
EP300	NOS3	9606.ENSP00000263253	9606.ENSP00000297494	0.049	0	0.9	0.9
EP300	PCNA	9606.ENSP00000263253	9606.ENSP00000368458	0.062	0.831	0.6	0.931
EP300	RARB	9606.ENSP00000263253	9606.ENSP00000332296	0	0.182	0.9	0.914
EP300	LDHA	9606.ENSP00000263253	9606.ENSP00000445175	0	0.282	0.9	0.925
EP300	MAPK8	9606.ENSP00000263253	9606.ENSP00000378974	0.062	0.27	0.9	0.925
EP300	RXRA	9606.ENSP00000263253	9606.ENSP00000419692	0	0.316	0.9	0.928
EP300	NR1H3	9606.ENSP00000263253	9606.ENSP00000477707	0.062	0.401	0.9	0.938
EP300	TNF	9606.ENSP00000263253	9606.ENSP00000398698	0	0.27	0.9	0.923
EP300	NCOR2	9606.ENSP00000263253	9606.ENSP00000384018	0.166	0.378	0.9	0.943
EP300	VDR	9606.ENSP00000263253	9606.ENSP00000447173	0	0.651	0.9	0.963

EP300	EZH2	9606.ENSP00000263253	9606.ENSP00000320147	0.064	0.378	0.9	0.936
EP300	HDAC6	9606.ENSP00000263253	9606.ENSP00000334061	0.062	0.797	0.65	0.927
EP300	NCOR1	9606.ENSP00000263253	9606.ENSP00000268712	0.425	0.5	0.9	0.968
EP300	HDAC3	9606.ENSP00000263253	9606.ENSP00000302967	0.064	0.722	0.9	0.971
EP300	EPAS1	9606.ENSP00000263253	9606.ENSP00000263734	0.062	0.778	0.9	0.977
EP300	STAT6	9606.ENSP00000263253	9606.ENSP00000300134	0.062	0.684	0.6	0.871
EP300	HDAC2	9606.ENSP00000263253	9606.ENSP00000430432	0.064	0.289	0.9	0.927
EP300	NR4A1	9606.ENSP00000263253	9606.ENSP00000440864	0	0.623	0.9	0.96
EP300	FOS	9606.ENSP00000263253	9606.ENSP00000306245	0.049	0.849	0.9	0.984
EP300	PPARA	9606.ENSP00000263253	9606.ENSP00000385523	0	0.7	0.9	0.968
EP300	KAT2B	9606.ENSP00000263253	9606.ENSP00000263754	0.062	0.883	0.9	0.988
EP300	HDAC1	9606.ENSP00000263253	9606.ENSP00000362649	0.064	0.9	0.9	0.989
EP300	RELA	9606.ENSP00000263253	9606.ENSP00000384273	0	0.922	0.9	0.991
EP300	JUN	9606.ENSP00000263253	9606.ENSP00000360266	0	0.91	0.9	0.99
EP300	PPARG	9606.ENSP00000263253	9606.ENSP00000287820	0	0.878	0.9	0.987
EP300	ESR1	9606.ENSP00000263253	9606.ENSP00000405330	0	0.929	0.9	0.992
EP300	STAT3	9606.ENSP00000263253	9606.ENSP00000264657	0.062	0.872	0.9	0.986
EP300	HIF1A	9606.ENSP00000263253	9606.ENSP00000437955	0.053	0.998	0.9	0.999
EPAS1	NR3C1	9606.ENSP00000263734	9606.ENSP00000231509	0.07	0.323	0.6	0.726
EPAS1	VHL	9606.ENSP00000263734	9606.ENSP00000256474	0	0.993	0.9	0.999
EPAS1	JUN	9606.ENSP00000263734	9606.ENSP00000360266	0.076	0.497	0	0.515
EPAS1	HIF1A	9606.ENSP00000263734	9606.ENSP00000437955	0	0.309	0.8	0.855
EPAS1	IKBKG	9606.ENSP00000263734	9606.ENSP00000483825	0	0.651	0	0.651
EPHA3	EPHA4	9606.ENSP00000337451	9606.ENSP00000281821	0.062	0	0.9	0.902
EPHA3	EPHB3	9606.ENSP00000337451	9606.ENSP00000332118	0	0	0.6	0.6
EPHA3	EPHB4	9606.ENSP00000337451	9606.ENSP00000350896	0	0.199	0.6	0.665
EPHA3	FGR	9606.ENSP00000337451	9606.ENSP00000363117	0.062	0	0.9	0.902
EPHA3	HCK	9606.ENSP00000337451	9606.ENSP00000444986	0.062	0	0.9	0.902
EPHA3	LCK	9606.ENSP00000337451	9606.ENSP00000337825	0.062	0	0.9	0.902
EPHA3	LYN	9606.ENSP00000337451	9606.ENSP00000428924	0.062	0	0.9	0.902
EPHA3	YES1	9606.ENSP00000337451	9606.ENSP00000462468	0.073	0.07	0.9	0.906
EPHA3	SRC	9606.ENSP00000337451	9606.ENSP00000362680	0.073	0.292	0.9	0.928
EPHA4	EPHB3	9606.ENSP00000281821	9606.ENSP00000332118	0.062	0	0.6	0.608
EPHA4	EPHB4	9606.ENSP00000281821	9606.ENSP00000350896	0	0.199	0.6	0.665
EPHA4	FGFR1	9606.ENSP00000281821	9606.ENSP00000393312	0	0.529	0	0.529
EPHA4	FGR	9606.ENSP00000281821	9606.ENSP00000363117	0.062	0	0.9	0.902
EPHA4	HCK	9606.ENSP00000281821	9606.ENSP00000444986	0.062	0	0.9	0.902
EPHA4	LCK	9606.ENSP00000281821	9606.ENSP00000337825	0.062	0	0.9	0.902
EPHA4	YES1	9606.ENSP00000281821	9606.ENSP00000462468	0.073	0.078	0.9	0.907
EPHA4	LYN	9606.ENSP00000281821	9606.ENSP00000428924	0.062	0	0.9	0.902
EPHA4	SRC	9606.ENSP00000281821	9606.ENSP00000362680	0.073	0.315	0.9	0.931
EPHB3	NCSTN	9606.ENSP00000332118	9606.ENSP00000294785	0.062	0.056	0.9	0.903
EPHB3	PSEN1	9606.ENSP00000332118	9606.ENSP00000326366	0	0.058	0.9	0.901
EPHB3	EPHB4	9606.ENSP00000332118	9606.ENSP00000350896	0.062	0.389	0.3	0.563

EPHB3	SRC	9606.ENSP00000332118	9606.ENSP00000362680	0.1	0.07	0.6	0.635
EPHB3	PSENEN	9606.ENSP00000332118	9606.ENSP00000468411	0	0	0.9	0.9
EPHB3	ROCK1	9606.ENSP00000332118	9606.ENSP00000382697	0.064	0	0.9	0.902
EPHB3	PSEN2	9606.ENSP00000332118	9606.ENSP00000355747	0	0.058	0.9	0.901
EPHB3	MMP9	9606.ENSP00000332118	9606.ENSP00000361405	0	0	0.9	0.9
EPHB3	RAC1	9606.ENSP00000332118	9606.ENSP00000348461	0	0.14	0.9	0.91
EPHB4	PIK3CA	9606.ENSP00000350896	9606.ENSP00000263967	0.062	0.104	0.9	0.908
EPHB4	NCSTN	9606.ENSP00000350896	9606.ENSP00000294785	0.062	0.056	0.9	0.903
EPHB4	PSEN1	9606.ENSP00000350896	9606.ENSP00000326366	0	0.058	0.9	0.901
EPHB4	RAC1	9606.ENSP00000350896	9606.ENSP00000348461	0	0.14	0.9	0.91
EPHB4	SRC	9606.ENSP00000350896	9606.ENSP00000362680	0.073	0.069	0.6	0.624
EPHB4	PSEN2	9606.ENSP00000350896	9606.ENSP00000355747	0	0.058	0.9	0.901
EPHB4	PSENEN	9606.ENSP00000350896	9606.ENSP00000468411	0	0	0.9	0.9
EPHB4	PIK3R1	9606.ENSP00000350896	9606.ENSP00000428056	0	0.102	0.9	0.906
EPHB4	MMP9	9606.ENSP00000350896	9606.ENSP00000361405	0	0	0.9	0.9
EPHX1	HPGDS	9606.ENSP00000480004	9606.ENSP00000295256	0.063	0	0.65	0.658
EPHX2	SLC27A2	9606.ENSP00000430269	9606.ENSP00000267842	0.082	0.136	0.9	0.913
EPHX2	PAOX	9606.ENSP00000430269	9606.ENSP00000278060	0	0	0.9	0.9
EPHX2	NOS2	9606.ENSP00000430269	9606.ENSP00000327251	0	0	0.9	0.9
EPHX2	IDH1	9606.ENSP00000430269	9606.ENSP00000390265	0.063	0	0.9	0.902
ERBB2	PDGFRA	9606.ENSP00000269571	9606.ENSP00000257290	0.062	0.477	0	0.489
ERBB2	PDGFRB	9606.ENSP00000269571	9606.ENSP00000261799	0.063	0.553	0	0.563
ERBB2	FLT4	9606.ENSP00000269571	9606.ENSP00000261937	0.062	0.456	0	0.468
ERBB2	PIK3CA	9606.ENSP00000269571	9606.ENSP00000263967	0	0.12	0.9	0.908
ERBB2	TXK	9606.ENSP00000269571	9606.ENSP00000264316	0	0.485	0	0.485
ERBB2	STAT3	9606.ENSP00000269571	9606.ENSP00000264657	0	0.743	0	0.743
ERBB2	IGF1R	9606.ENSP00000269571	9606.ENSP00000268035	0	0.534	0	0.534
ERBB2	ITK	9606.ENSP00000269571	9606.ENSP00000398655	0	0.444	0	0.444
ERBB2	FGR	9606.ENSP00000269571	9606.ENSP00000363117	0	0.444	0	0.444
ERBB2	SYK	9606.ENSP00000269571	9606.ENSP00000364907	0	0.447	0	0.447
ERBB2	NTRK1	9606.ENSP00000269571	9606.ENSP00000431418	0.091	0.377	0	0.409
ERBB2	PTK2B	9606.ENSP00000269571	9606.ENSP00000380638	0.064	0.674	0	0.682
ERBB2	ROCK2	9606.ENSP00000269571	9606.ENSP00000317985	0	0.107	0.65	0.674
ERBB2	ROCK1	9606.ENSP00000269571	9606.ENSP00000382697	0	0.107	0.65	0.674
ERBB2	JAK3	9606.ENSP00000269571	9606.ENSP00000391676	0	0.107	0.8	0.813
ERBB2	PIK3CD	9606.ENSP00000269571	9606.ENSP00000366563	0	0.058	0.8	0.803
ERBB2	JAK1	9606.ENSP00000269571	9606.ENSP00000343204	0	0.302	0.8	0.854
ERBB2	YES1	9606.ENSP00000269571	9606.ENSP00000462468	0.079	0.341	0.9	0.934
ERBB2	MAPK8	9606.ENSP00000269571	9606.ENSP00000378974	0	0	0.9	0.9
ERBB2	MAP2K1	9606.ENSP00000269571	9606.ENSP00000302486	0	0.285	0.9	0.925
ERBB2	JAK2	9606.ENSP00000269571	9606.ENSP00000371067	0	0.32	0.9	0.929
ERBB2	PIK3CB	9606.ENSP00000269571	9606.ENSP00000289153	0	0.12	0.9	0.908
ERBB2	PTK2	9606.ENSP00000269571	9606.ENSP00000341189	0.072	0.798	0.8	0.959
ERBB2	SRC	9606.ENSP00000269571	9606.ENSP00000362680	0.062	0.897	0.8	0.979

ERBB2	PIK3R1	9606.ENSP00000269571	9606.ENSP00000428056	0	0.884	0.9	0.987
ERBB2	PTPN11	9606.ENSP00000269571	9606.ENSP00000340944	0	0.939	0.9	0.993
ERBB2	HSP90AA1	9606.ENSP00000269571	9606.ENSP00000335153	0	0.907	0.9	0.99
ERCC5	POLK	9606.ENSP00000347978	9606.ENSP00000241436	0.075	0.077	0.6	0.628
ERCC5	PARP2	9606.ENSP00000347978	9606.ENSP00000250416	0.109	0	0.6	0.628
ERCC5	PRKDC	9606.ENSP00000347978	9606.ENSP00000313420	0.079	0.157	0.9	0.915
ERCC5	PARP1	9606.ENSP00000347978	9606.ENSP00000355759	0.109	0	0.6	0.628
ERCC5	KDM1A	9606.ENSP00000347978	9606.ENSP00000383042	0.065	0	0.9	0.902
ERCC5	PCNA	9606.ENSP00000347978	9606.ENSP00000368458	0.234	0.646	0.6	0.882
ERN1	PSEN1	9606.ENSP00000401445	9606.ENSP00000326366	0	0.282	0.8	0.85
ERN1	PSEN2	9606.ENSP00000401445	9606.ENSP00000355747	0	0.058	0.8	0.803
ESR1	MAPK1	9606.ENSP00000405330	9606.ENSP00000215832	0	0.699	0.9	0.968
ESR1	RPS6KB1	9606.ENSP00000405330	9606.ENSP00000225577	0	0.068	0.8	0.805
ESR1	NFKB1	9606.ENSP00000405330	9606.ENSP00000226574	0	0.494	0	0.494
ESR1	MAPK14	9606.ENSP00000405330	9606.ENSP00000229795	0	0.264	0.9	0.923
ESR1	NR3C1	9606.ENSP00000405330	9606.ENSP00000231509	0	0	0.9	0.9
ESR1	MDM2	9606.ENSP00000405330	9606.ENSP00000258149	0	0.757	0	0.757
ESR1	GNAO1	9606.ENSP00000405330	9606.ENSP00000262494	0	0.056	0.9	0.901
ESR1	MAPK3	9606.ENSP00000405330	9606.ENSP00000263025	0	0.264	0.9	0.923
ESR1	KAT2B	9606.ENSP00000405330	9606.ENSP00000263754	0	0.102	0.6	0.625
ESR1	PIK3CA	9606.ENSP00000405330	9606.ENSP00000263967	0	0.496	0.9	0.947
ESR1	HDAC4	9606.ENSP00000405330	9606.ENSP00000264606	0	0.505	0.9	0.948
ESR1	STAT3	9606.ENSP00000405330	9606.ENSP00000264657	0	0.507	0	0.507
ESR1	IGF1R	9606.ENSP00000405330	9606.ENSP00000268035	0	0.774	0.9	0.976
ESR1	NCOR1	9606.ENSP00000405330	9606.ENSP00000268712	0	0.797	0.9	0.978
ESR1	NOS3	9606.ENSP00000405330	9606.ENSP00000297494	0	0.345	0.9	0.931
ESR1	HDAC3	9606.ENSP00000405330	9606.ENSP00000302967	0	0.709	0	0.709
ESR1	FOS	9606.ENSP00000405330	9606.ENSP00000306245	0	0.422	0.9	0.939
ESR1	TERT	9606.ENSP00000405330	9606.ENSP00000309572	0	0	0.9	0.9
ESR1	PGR	9606.ENSP00000405330	9606.ENSP00000325120	0.102	0.825	0.6	0.931
ESR1	HSP90AA1	9606.ENSP00000405330	9606.ENSP00000335153	0	0.887	0.6	0.953
ESR1	PTK2	9606.ENSP00000405330	9606.ENSP00000341189	0	0	0.6	0.6
ESR1	GNAI1	9606.ENSP00000405330	9606.ENSP00000343027	0	0.478	0.9	0.945
ESR1	ESR2	9606.ENSP00000405330	9606.ENSP00000343925	0	0.873	0.9	0.986
ESR1	MAPK10	9606.ENSP00000405330	9606.ENSP00000352157	0	0	0.8	0.8
ESR1	GNAI3	9606.ENSP00000405330	9606.ENSP00000358867	0	0.284	0.9	0.925
ESR1	JUN	9606.ENSP00000405330	9606.ENSP00000360266	0	0.684	0.9	0.967
ESR1	HDAC1	9606.ENSP00000405330	9606.ENSP00000362649	0	0.876	0.9	0.987
ESR1	SRC	9606.ENSP00000405330	9606.ENSP00000362680	0.05	0.921	0.9	0.991
ESR1	S1PR3	9606.ENSP00000405330	9606.ENSP00000365006	0	0	0.65	0.65
ESR1	PCNA	9606.ENSP00000405330	9606.ENSP00000368458	0.049	0.079	0.9	0.904
ESR1	RPS6KA3	9606.ENSP00000405330	9606.ENSP00000368884	0	0.47	0	0.47
ESR1	NR2F2	9606.ENSP00000405330	9606.ENSP00000377721	0	0.94	0	0.94
ESR1	MAPK8	9606.ENSP00000405330	9606.ENSP00000378974	0	0	0.8	0.8

ESR1	KDM1A	9606.ENSP00000405330	9606.ENSP00000383042	0	0.337	0.6	0.723
ESR1	NCOR2	9606.ENSP00000405330	9606.ENSP00000384018	0	0.798	0.9	0.978
ESR1	RELA	9606.ENSP00000405330	9606.ENSP00000384273	0	0.757	0	0.757
ESR1	SMARCA4	9606.ENSP00000405330	9606.ENSP00000395654	0	0.519	0	0.519
ESR1	NR1H4	9606.ENSP00000405330	9606.ENSP00000447149	0.062	0.486	0	0.497
ESR1	HIF1A	9606.ENSP00000405330	9606.ENSP00000437955	0	0.587	0	0.587
ESR1	PIK3R1	9606.ENSP00000405330	9606.ENSP00000428056	0.063	0.745	0.9	0.974
ESR2	MAPK1	9606.ENSP00000343925	9606.ENSP00000215832	0	0.317	0.8	0.857
ESR2	RPS6KB1	9606.ENSP00000343925	9606.ENSP00000225577	0	0.068	0.8	0.805
ESR2	MAPK14	9606.ENSP00000343925	9606.ENSP00000229795	0.062	0.104	0.8	0.817
ESR2	MAPK3	9606.ENSP00000343925	9606.ENSP00000263025	0	0.104	0.8	0.813
ESR2	PIK3CA	9606.ENSP00000343925	9606.ENSP00000263967	0	0.061	0.5	0.51
ESR2	NCOR1	9606.ENSP00000343925	9606.ENSP00000268712	0.063	0.35	0.8	0.867
ESR2	NOS3	9606.ENSP00000343925	9606.ENSP00000297494	0	0.067	0.9	0.902
ESR2	FOS	9606.ENSP00000343925	9606.ENSP00000306245	0	0.182	0.8	0.829
ESR2	HSP90AA1	9606.ENSP00000343925	9606.ENSP00000335153	0	0.285	0.6	0.702
ESR2	PTK2	9606.ENSP00000343925	9606.ENSP00000341189	0	0	0.65	0.65
ESR2	GNAI1	9606.ENSP00000343925	9606.ENSP00000343027	0	0.056	0.6	0.606
ESR2	GNAI3	9606.ENSP00000343925	9606.ENSP00000358867	0	0.056	0.6	0.606
ESR2	PIK3R1	9606.ENSP00000343925	9606.ENSP00000428056	0	0.07	0.5	0.515
ESR2	MAPK10	9606.ENSP00000343925	9606.ENSP00000352157	0.062	0	0.8	0.804
ESR2	MAPK8	9606.ENSP00000343925	9606.ENSP00000378974	0.062	0	0.8	0.804
ESR2	SMARCA4	9606.ENSP00000343925	9606.ENSP00000395654	0	0.078	0.9	0.903
ESR2	NCOR2	9606.ENSP00000343925	9606.ENSP00000384018	0.062	0.575	0	0.584
ESR2	JUN	9606.ENSP00000343925	9606.ENSP00000360266	0	0.066	0.8	0.805
ESR2	SRC	9606.ENSP00000343925	9606.ENSP00000362680	0	0.749	0.8	0.947
ESRRG	HIF1A	9606.ENSP00000355904	9606.ENSP00000437955	0	0.4	0	0.4
ESRRG	NCOR2	9606.ENSP00000355904	9606.ENSP00000384018	0	0.709	0	0.709
EZH2	HDAC7	9606.ENSP00000320147	9606.ENSP00000080059	0.062	0.17	0.65	0.703
EZH2	HDAC5	9606.ENSP00000320147	9606.ENSP00000225983	0.062	0.171	0.65	0.704
EZH2	RARA	9606.ENSP00000320147	9606.ENSP00000254066	0	0.309	0.6	0.712
EZH2	KIF11	9606.ENSP00000320147	9606.ENSP00000260731	0.583	0.057	0	0.59
EZH2	NCOR1	9606.ENSP00000320147	9606.ENSP00000268712	0.076	0.218	0.6	0.685
EZH2	PPARG	9606.ENSP00000320147	9606.ENSP00000287820	0	0.309	0.65	0.748
EZH2	MELK	9606.ENSP00000320147	9606.ENSP00000298048	0.789	0.065	0	0.794
EZH2	HDAC3	9606.ENSP00000320147	9606.ENSP00000302967	0.062	0.661	0.6	0.861
EZH2	RARG	9606.ENSP00000320147	9606.ENSP00000388510	0	0.093	0.6	0.621
EZH2	RARB	9606.ENSP00000320147	9606.ENSP00000332296	0	0.093	0.6	0.621
EZH2	TTK	9606.ENSP00000320147	9606.ENSP00000358813	0.671	0	0	0.671
EZH2	RXRA	9606.ENSP00000320147	9606.ENSP00000419692	0	0.093	0.6	0.621
EZH2	JUN	9606.ENSP00000320147	9606.ENSP00000360266	0	0	0.65	0.65
EZH2	TOP2A	9606.ENSP00000320147	9606.ENSP00000411532	0.7	0.043	0	0.7
EZH2	SMARCA4	9606.ENSP00000320147	9606.ENSP00000395654	0.087	0.372	0	0.403
EZH2	NCOR2	9606.ENSP00000320147	9606.ENSP00000384018	0.076	0.154	0.9	0.915

EZH2	KDM1A	9606.ENSP00000320147	9606.ENSP00000383042	0.1	0.335	0.65	0.772
EZH2	HDAC2	9606.ENSP00000320147	9606.ENSP00000430432	0.097	0.896	0.9	0.989
EZH2	HDAC1	9606.ENSP00000320147	9606.ENSP00000362649	0.097	0.939	0.9	0.994
F13A1	F2	9606.ENSP00000264870	9606.ENSP00000308541	0	0.213	0.9	0.917
F2	MAPK1	9606.ENSP00000308541	9606.ENSP00000215832	0	0.056	0.9	0.901
F2	RAF1	9606.ENSP00000308541	9606.ENSP00000251849	0	0.056	0.9	0.901
F2	MAPK3	9606.ENSP00000308541	9606.ENSP00000263025	0	0.056	0.9	0.901
F2	TYR	9606.ENSP00000308541	9606.ENSP00000263321	0.527	0	0	0.527
F2	HTR2C	9606.ENSP00000308541	9606.ENSP00000276198	0	0.097	0.9	0.905
F2	PTGER1	9606.ENSP00000308541	9606.ENSP00000292513	0	0.097	0.9	0.905
F2	FABP1	9606.ENSP00000308541	9606.ENSP00000295834	0.587	0	0	0.587
F2	MAP2K1	9606.ENSP00000308541	9606.ENSP00000302486	0	0.147	0.9	0.911
F2	GRM5	9606.ENSP00000308541	9606.ENSP00000306138	0	0	0.6	0.6
F2	SERPINA6	9606.ENSP00000308541	9606.ENSP00000342850	0.366	0.12	0	0.418
F2	GRM1	9606.ENSP00000308541	9606.ENSP00000354896	0	0	0.6	0.6
F2	GRM2	9606.ENSP00000308541	9606.ENSP00000378492	0	0	0.6	0.6
F2	GRM4	9606.ENSP00000308541	9606.ENSP00000440556	0	0	0.6	0.6
F2	PTGFR	9606.ENSP00000308541	9606.ENSP00000359793	0	0.097	0.6	0.623
F2	LPAR6	9606.ENSP00000308541	9606.ENSP00000367691	0	0	0.8	0.8
F2	LPAR4	9606.ENSP00000308541	9606.ENSP00000408205	0	0	0.8	0.8
F2	LPAR3	9606.ENSP00000308541	9606.ENSP00000395389	0	0.097	0.8	0.811
F2	LPAR2	9606.ENSP00000308541	9606.ENSP00000443256	0.062	0.097	0.8	0.815
F2	LPAR1	9606.ENSP00000308541	9606.ENSP00000363553	0	0.097	0.8	0.811
F2	HCK	9606.ENSP00000308541	9606.ENSP00000444986	0	0.056	0.9	0.901
F2	FGR	9606.ENSP00000308541	9606.ENSP00000363117	0	0.056	0.9	0.901
F2	GNAI1	9606.ENSP00000308541	9606.ENSP00000343027	0	0	0.9	0.9
F2	PPARA	9606.ENSP00000308541	9606.ENSP00000385523	0.064	0	0.9	0.902
F2	PLAU	9606.ENSP00000308541	9606.ENSP00000361850	0	0.213	0.9	0.917
F2	SRC	9606.ENSP00000308541	9606.ENSP00000362680	0	0.056	0.9	0.901
F2	F2R	9606.ENSP00000308541	9606.ENSP00000321326	0	0.932	0.9	0.992
F2R	MAPK1	9606.ENSP00000321326	9606.ENSP00000215832	0	0	0.6	0.6
F2R	GNAO1	9606.ENSP00000321326	9606.ENSP00000262494	0	0	0.9	0.9
F2R	MAPK3	9606.ENSP00000321326	9606.ENSP00000263025	0	0	0.6	0.6
F2R	PRSS1	9606.ENSP00000321326	9606.ENSP00000308720	0	0	0.6	0.6
F2R	PIK3CG	9606.ENSP00000321326	9606.ENSP00000352121	0	0	0.6	0.6
F2R	SRC	9606.ENSP00000321326	9606.ENSP00000362680	0	0	0.6	0.6
F2R	LPAR6	9606.ENSP00000321326	9606.ENSP00000367691	0	0	0.8	0.8
F2R	LPAR4	9606.ENSP00000321326	9606.ENSP00000408205	0	0	0.8	0.8
F2R	LPAR1	9606.ENSP00000321326	9606.ENSP00000363553	0	0	0.8	0.8
F2R	LPAR3	9606.ENSP00000321326	9606.ENSP00000395389	0	0	0.8	0.8
F2R	LPAR2	9606.ENSP00000321326	9606.ENSP00000443256	0	0	0.8	0.8
F2R	MMP1	9606.ENSP00000321326	9606.ENSP00000322788	0.077	0.393	0	0.415
F2R	GNAI3	9606.ENSP00000321326	9606.ENSP00000358867	0	0	0.9	0.9
F2R	GNAI1	9606.ENSP00000321326	9606.ENSP00000343027	0	0	0.9	0.9

FABP1	LIPE	9606.ENSP00000295834	9606.ENSP00000244289	0	0.056	0.65	0.655
FABP1	FABP4	9606.ENSP00000295834	9606.ENSP00000256104	0.062	0	0.65	0.657
FABP1	NCOR1	9606.ENSP00000295834	9606.ENSP00000268712	0	0	0.65	0.65
FABP1	FABP2	9606.ENSP00000295834	9606.ENSP00000274024	0.481	0	0	0.481
FABP1	HMGCR	9606.ENSP00000295834	9606.ENSP00000287936	0	0	0.65	0.65
FABP1	HNF4A	9606.ENSP00000295834	9606.ENSP00000312987	0.328	0.175	0	0.422
FABP1	HDAC3	9606.ENSP00000295834	9606.ENSP00000302967	0	0	0.65	0.65
FABP1	NCOR2	9606.ENSP00000295834	9606.ENSP00000384018	0	0	0.65	0.65
FABP1	FDFT1	9606.ENSP00000295834	9606.ENSP00000480828	0	0	0.65	0.65
FABP1	NR1H4	9606.ENSP00000295834	9606.ENSP00000447149	0.661	0.064	0	0.669
FABP1	RELA	9606.ENSP00000295834	9606.ENSP00000384273	0	0	0.9	0.9
FABP1	JUN	9606.ENSP00000295834	9606.ENSP00000360266	0	0	0.9	0.9
FABP1	RXRA	9606.ENSP00000295834	9606.ENSP00000419692	0.062	0.064	0.9	0.904
FABP1	NR1H3	9606.ENSP00000295834	9606.ENSP00000477707	0.064	0.064	0.9	0.904
FABP1	LPL	9606.ENSP00000295834	9606.ENSP00000309757	0.055	0	0.9	0.901
FABP1	PPARA	9606.ENSP00000295834	9606.ENSP00000385523	0.065	0.285	0.9	0.927
FABP2	LIPE	9606.ENSP00000274024	9606.ENSP00000244289	0	0.056	0.65	0.655
FABP2	FABP4	9606.ENSP00000274024	9606.ENSP00000256104	0	0	0.65	0.65
FABP3	LIPE	9606.ENSP00000362817	9606.ENSP00000244289	0	0.056	0.65	0.655
FABP3	FABP4	9606.ENSP00000362817	9606.ENSP00000256104	0.062	0	0.65	0.657
FABP3	FKBP1A	9606.ENSP00000362817	9606.ENSP00000383003	0.062	0.718	0	0.724
FABP4	LIPE	9606.ENSP00000256104	9606.ENSP00000244289	0.062	0.705	0.6	0.879
FABP4	FABP5	9606.ENSP00000256104	9606.ENSP00000297258	0.068	0	0.65	0.659
FABP4	RXRA	9606.ENSP00000256104	9606.ENSP00000419692	0.062	0.064	0.9	0.904
FABP4	PPARG	9606.ENSP00000256104	9606.ENSP00000287820	0.088	0.064	0.9	0.907
FABP5	LIPE	9606.ENSP00000297258	9606.ENSP00000244289	0.052	0.056	0.65	0.659
FABP5	PIN1	9606.ENSP00000297258	9606.ENSP00000247970	0	0.644	0	0.644
FABP5	GLO1	9606.ENSP00000297258	9606.ENSP00000362463	0.063	0.484	0	0.496
FABP5	RXRA	9606.ENSP00000297258	9606.ENSP00000419692	0	0.064	0.6	0.609
FABP5	FKBP1A	9606.ENSP00000297258	9606.ENSP00000383003	0.062	0.788	0	0.792
FABP5	PPARD	9606.ENSP00000297258	9606.ENSP00000310928	0	0.064	0.6	0.609
FDFT1	FNTB	9606.ENSP00000480828	9606.ENSP00000246166	0.062	0	0.8	0.804
FDFT1	SQLE	9606.ENSP00000480828	9606.ENSP00000265896	0.569	0	0.9	0.955
FDFT1	GGPS1	9606.ENSP00000480828	9606.ENSP00000282841	0.112	0.151	0.9	0.918
FDFT1	FNTA	9606.ENSP00000480828	9606.ENSP00000303423	0.062	0	0.8	0.804
FDFT1	FDPS	9606.ENSP00000480828	9606.ENSP00000349078	0.534	0.151	0.9	0.957
FDFT1	SREBF2	9606.ENSP00000480828	9606.ENSP00000354476	0.085	0.068	0.9	0.907
FDFT1	LSS	9606.ENSP00000480828	9606.ENSP00000380837	0.639	0	0	0.639
FDFT1	PPARA	9606.ENSP00000480828	9606.ENSP00000385523	0	0	0.9	0.9
FDFT1	RXRA	9606.ENSP00000480828	9606.ENSP00000419692	0	0	0.9	0.9
FDPS	FNTB	9606.ENSP00000349078	9606.ENSP00000246166	0.062	0.122	0.9	0.91
FDPS	SQLE	9606.ENSP00000349078	9606.ENSP00000265896	0.471	0.066	0	0.485
FDPS	GGPS1	9606.ENSP00000349078	9606.ENSP00000282841	0.054	0.379	0.9	0.936
FDPS	FNTA	9606.ENSP00000349078	9606.ENSP00000303423	0.062	0	0.9	0.902

FDPS	IDH1	9606.ENSP00000349078	9606.ENSP00000390265	0.125	0.485	0	0.53
FDPS	SREBF2	9606.ENSP00000349078	9606.ENSP00000354476	0.064	0	0.9	0.902
FEN1	PARP2	9606.ENSP00000305480	9606.ENSP00000250416	0.22	0.209	0.6	0.731
FEN1	KIF11	9606.ENSP00000305480	9606.ENSP00000260731	0.77	0	0	0.77
FEN1	POLB	9606.ENSP00000305480	9606.ENSP00000265421	0.049	0.105	0.8	0.814
FEN1	MELK	9606.ENSP00000305480	9606.ENSP00000298048	0.802	0	0	0.802
FEN1	PLK1	9606.ENSP00000305480	9606.ENSP00000300093	0.532	0.062	0	0.543
FEN1	TTK	9606.ENSP00000305480	9606.ENSP00000358813	0.669	0	0	0.669
FEN1	TYMS	9606.ENSP00000305480	9606.ENSP00000315644	0.839	0.056	0	0.842
FEN1	TOP2A	9606.ENSP00000305480	9606.ENSP00000411532	0.787	0.056	0	0.791
FEN1	RAD52	9606.ENSP00000305480	9606.ENSP00000351284	0.083	0.564	0	0.583
FEN1	PARP1	9606.ENSP00000305480	9606.ENSP00000355759	0.249	0.209	0.6	0.741
FEN1	POLA1	9606.ENSP00000305480	9606.ENSP00000368349	0.592	0.224	0.9	0.965
FEN1	PCNA	9606.ENSP00000305480	9606.ENSP00000368458	0.964	0.995	0.6	0.999
FGFR1	MAPK1	9606.ENSP00000393312	9606.ENSP00000215832	0	0.081	0.9	0.904
FGFR1	FLT3	9606.ENSP00000393312	9606.ENSP00000241453	0	0	0.5	0.499
FGFR1	MAPK3	9606.ENSP00000393312	9606.ENSP00000263025	0	0.081	0.9	0.904
FGFR1	KDR	9606.ENSP00000393312	9606.ENSP00000263923	0.07	0.399	0	0.417
FGFR1	PIK3CA	9606.ENSP00000393312	9606.ENSP00000263967	0	0.097	0.8	0.811
FGFR1	FLT1	9606.ENSP00000393312	9606.ENSP00000282397	0.068	0.394	0	0.411
FGFR1	PIK3CB	9606.ENSP00000393312	9606.ENSP00000289153	0	0.097	0.8	0.811
FGFR1	HTR1A	9606.ENSP00000393312	9606.ENSP00000316244	0	0.631	0	0.631
FGFR1	PKM	9606.ENSP00000393312	9606.ENSP00000320171	0.051	0.42	0	0.426
FGFR1	HSP90AA1	9606.ENSP00000393312	9606.ENSP00000335153	0	0.442	0	0.442
FGFR1	PTPN11	9606.ENSP00000393312	9606.ENSP00000340944	0.063	0.109	0.5	0.546
FGFR1	PTPN1	9606.ENSP00000393312	9606.ENSP00000360683	0	0.659	0	0.659
FGFR1	PIK3CD	9606.ENSP00000393312	9606.ENSP00000366563	0	0.097	0.8	0.811
FGFR1	NTRK1	9606.ENSP00000393312	9606.ENSP00000431418	0.062	0.391	0	0.404
FGFR1	PIK3R1	9606.ENSP00000393312	9606.ENSP00000428056	0	0.807	0.8	0.959
FGR	MAPK14	9606.ENSP00000363117	9606.ENSP00000229795	0	0.148	0.9	0.911
FGR	FLT3	9606.ENSP00000363117	9606.ENSP00000241453	0.159	0.331	0	0.413
FGR	S1PR4	9606.ENSP00000363117	9606.ENSP00000246115	0.558	0	0	0.558
FGR	TLR2	9606.ENSP00000363117	9606.ENSP00000260010	0.558	0.089	0	0.58
FGR	PDGFRB	9606.ENSP00000363117	9606.ENSP00000261799	0	0.122	0.9	0.908
FGR	PIK3CA	9606.ENSP00000363117	9606.ENSP00000263967	0.062	0.104	0.6	0.634
FGR	STAT3	9606.ENSP00000363117	9606.ENSP00000264657	0.062	0.384	0.9	0.937
FGR	PIK3CB	9606.ENSP00000363117	9606.ENSP00000289153	0.062	0.104	0.6	0.634
FGR	S1PR1	9606.ENSP00000363117	9606.ENSP00000305416	0.112	0.069	0.9	0.91
FGR	TLR8	9606.ENSP00000363117	9606.ENSP00000312082	0.533	0.089	0	0.556
FGR	HSP90AA1	9606.ENSP00000363117	9606.ENSP00000335153	0	0.538	0	0.538
FGR	LCK	9606.ENSP00000363117	9606.ENSP00000337825	0.069	0	0.9	0.902
FGR	PTK2	9606.ENSP00000363117	9606.ENSP00000341189	0	0.298	0.9	0.926
FGR	GNAI1	9606.ENSP00000363117	9606.ENSP00000343027	0	0.159	0.6	0.649
FGR	PIK3CG	9606.ENSP00000363117	9606.ENSP00000352121	0.177	0.104	0.6	0.679

FGR	MPEG1	9606.ENSP00000363117	9606.ENSP00000354335	0.456	0	0	0.456
FGR	PLA2G4A	9606.ENSP00000363117	9606.ENSP00000356436	0.062	0.112	0.65	0.683
FGR	GNAI3	9606.ENSP00000363117	9606.ENSP00000358867	0	0.159	0.6	0.649
FGR	SRC	9606.ENSP00000363117	9606.ENSP00000362680	0	0.213	0.9	0.917
FGR	IKBKG	9606.ENSP00000363117	9606.ENSP00000483825	0	0.452	0	0.452
FGR	PIK3R1	9606.ENSP00000363117	9606.ENSP00000428056	0.061	0.102	0.6	0.633
FGR	PIK3CD	9606.ENSP00000363117	9606.ENSP00000366563	0.153	0.104	0.6	0.67
FGR	ITGB2	9606.ENSP00000363117	9606.ENSP00000380948	0.676	0.147	0	0.712
FGR	ITK	9606.ENSP00000363117	9606.ENSP00000398655	0.078	0	0.9	0.903
FGR	PRKCD	9606.ENSP00000363117	9606.ENSP00000378217	0.108	0.117	0.9	0.914
FGR	PTK2B	9606.ENSP00000363117	9606.ENSP00000380638	0.152	0.078	0.9	0.914
FGR	LYN	9606.ENSP00000363117	9606.ENSP00000428924	0.187	0.241	0.9	0.932
FGR	YES1	9606.ENSP00000363117	9606.ENSP00000462468	0	0.465	0.9	0.944
FGR	SYK	9606.ENSP00000363117	9606.ENSP00000364907	0.228	0.515	0.9	0.959
FGR	HCK	9606.ENSP00000363117	9606.ENSP00000444986	0.712	0	0.9	0.97
FKBP1A	RPS6KB1	9606.ENSP00000383003	9606.ENSP00000225577	0	0	0.9	0.9
FKBP1A	PIN1	9606.ENSP00000383003	9606.ENSP00000247970	0.065	0.711	0	0.719
FKBP1A	PDE4B	9606.ENSP00000383003	9606.ENSP00000332116	0	0	0.54	0.54
FKBP1A	PDE4D	9606.ENSP00000383003	9606.ENSP00000345502	0	0	0.54	0.54
FKBP1A	MTOR	9606.ENSP00000383003	9606.ENSP00000354558	0	0.995	0.9	0.999
FKBP1A	TGFBR1	9606.ENSP00000383003	9606.ENSP00000364133	0	0.973	0.9	0.997
FKBP1A	PRKCZ	9606.ENSP00000383003	9606.ENSP00000367830	0	0.058	0.6	0.607
FLT1	FLT4	9606.ENSP00000282397	9606.ENSP00000261937	0.062	0.829	0.9	0.982
FLT1	KDR	9606.ENSP00000282397	9606.ENSP00000263923	0.162	0.98	0.9	0.998
FLT1	PIK3CA	9606.ENSP00000282397	9606.ENSP00000263967	0	0.306	0.9	0.927
FLT1	TXK	9606.ENSP00000282397	9606.ENSP00000264316	0	0.4	0	0.4
FLT1	KIT	9606.ENSP00000282397	9606.ENSP00000288135	0.077	0.394	0	0.417
FLT1	HCK	9606.ENSP00000282397	9606.ENSP00000444986	0	0.511	0	0.511
FLT1	PIK3CD	9606.ENSP00000282397	9606.ENSP00000366563	0.062	0.306	0.6	0.716
FLT1	PIK3CB	9606.ENSP00000282397	9606.ENSP00000289153	0.049	0.306	0.6	0.712
FLT1	SRC	9606.ENSP00000282397	9606.ENSP00000362680	0.062	0.154	0.9	0.913
FLT1	PTK2	9606.ENSP00000282397	9606.ENSP00000341189	0	0.71	0.9	0.969
FLT1	PIK3R1	9606.ENSP00000282397	9606.ENSP00000428056	0	0.822	0.9	0.981
FLT1	PTPN11	9606.ENSP00000282397	9606.ENSP00000340944	0.063	0.878	0.9	0.987
FLT3	LCK	9606.ENSP00000241453	9606.ENSP00000337825	0	0.201	0.5	0.583
FLT3	HCK	9606.ENSP00000241453	9606.ENSP00000444986	0.153	0.331	0.6	0.753
FLT3	SYK	9606.ENSP00000241453	9606.ENSP00000364907	0.098	0.428	0.6	0.775
FLT3	PIK3CB	9606.ENSP00000241453	9606.ENSP00000289153	0	0.097	0.8	0.811
FLT3	PIK3CD	9606.ENSP00000241453	9606.ENSP00000366563	0.065	0.097	0.8	0.816
FLT3	PIK3CA	9606.ENSP00000241453	9606.ENSP00000263967	0	0.097	0.8	0.811
FLT3	STAT3	9606.ENSP00000241453	9606.ENSP00000264657	0	0.111	0.8	0.814
FLT3	PIK3R1	9606.ENSP00000241453	9606.ENSP00000428056	0.065	0.376	0.8	0.873
FLT3	PTPN11	9606.ENSP00000241453	9606.ENSP00000340944	0.063	0.424	0.5	0.706
FLT4	TXK	9606.ENSP00000261937	9606.ENSP00000264316	0	0.4	0	0.4

FLT4	KIT	9606.ENSP00000261937	9606.ENSP00000288135	0.052	0.395	0	0.401
FLT4	NTRK1	9606.ENSP00000261937	9606.ENSP00000431418	0	0.4	0	0.4
FLT4	PIK3CB	9606.ENSP00000261937	9606.ENSP00000289153	0	0.306	0.6	0.71
FLT4	PIK3CD	9606.ENSP00000261937	9606.ENSP00000366563	0	0.306	0.6	0.71
FLT4	SRC	9606.ENSP00000261937	9606.ENSP00000362680	0.062	0.154	0.9	0.913
FLT4	PTK2	9606.ENSP00000261937	9606.ENSP00000341189	0	0.281	0.9	0.925
FLT4	PIK3R1	9606.ENSP00000261937	9606.ENSP00000428056	0	0.355	0.9	0.932
FLT4	PIK3CA	9606.ENSP00000261937	9606.ENSP00000263967	0	0.306	0.9	0.927
FLT4	KDR	9606.ENSP00000261937	9606.ENSP00000263923	0.094	0.875	0.9	0.987
FNTA	FNTB	9606.ENSP00000303423	9606.ENSP00000246166	0.087	0.996	0.9	0.999
FNTA	GGPS1	9606.ENSP00000303423	9606.ENSP00000282841	0.122	0	0.9	0.908
FNTA	HDAC6	9606.ENSP00000303423	9606.ENSP00000334061	0.062	0.501	0	0.512
FNTA	PGGT1B	9606.ENSP00000303423	9606.ENSP00000404676	0.105	0.97	0.9	0.997
FNTB	HDAC6	9606.ENSP00000246166	9606.ENSP00000334061	0.052	0.429	0	0.435
FNTB	GGPS1	9606.ENSP00000246166	9606.ENSP00000282841	0.065	0.07	0.9	0.905
FOS	MAPK1	9606.ENSP00000306245	9606.ENSP00000215832	0	0.578	0.9	0.956
FOS	HMOX1	9606.ENSP00000306245	9606.ENSP00000216117	0	0	0.9	0.9
FOS	RPS6KB1	9606.ENSP00000306245	9606.ENSP00000225577	0.062	0.102	0.8	0.816
FOS	MAPK14	9606.ENSP00000306245	9606.ENSP00000229795	0	0.248	0.9	0.921
FOS	NR3C1	9606.ENSP00000306245	9606.ENSP00000231509	0.054	0.225	0.9	0.92
FOS	RORA	9606.ENSP00000306245	9606.ENSP00000261523	0	0.077	0.9	0.903
FOS	MAPK3	9606.ENSP00000306245	9606.ENSP00000263025	0	0.427	0.9	0.94
FOS	PRKCG	9606.ENSP00000306245	9606.ENSP00000263431	0	0	0.8	0.8
FOS	STAT3	9606.ENSP00000306245	9606.ENSP00000264657	0	0.416	0.9	0.939
FOS	PPARG	9606.ENSP00000306245	9606.ENSP00000287820	0	0.082	0.9	0.904
FOS	PRKCB	9606.ENSP00000306245	9606.ENSP00000305355	0	0	0.8	0.8
FOS	NCOR2	9606.ENSP00000306245	9606.ENSP00000384018	0	0.43	0	0.43
FOS	NR4A1	9606.ENSP00000306245	9606.ENSP00000440864	0.403	0.146	0	0.468
FOS	PRKCA	9606.ENSP00000306245	9606.ENSP00000408695	0	0	0.8	0.8
FOS	LPAR2	9606.ENSP00000306245	9606.ENSP00000443256	0	0	0.9	0.9
FOS	PIM1	9606.ENSP00000306245	9606.ENSP00000362608	0.062	0.078	0.9	0.905
FOS	LPAR1	9606.ENSP00000306245	9606.ENSP00000363553	0	0	0.9	0.9
FOS	MAPK10	9606.ENSP00000306245	9606.ENSP00000352157	0	0.243	0.8	0.842
FOS	RORC	9606.ENSP00000306245	9606.ENSP00000327025	0	0.077	0.9	0.903
FOS	LYN	9606.ENSP00000306245	9606.ENSP00000428924	0	0.064	0.9	0.902
FOS	PTK2	9606.ENSP00000306245	9606.ENSP00000341189	0	0	0.9	0.9
FOS	SRC	9606.ENSP00000306245	9606.ENSP00000362680	0	0.064	0.8	0.804
FOS	SYK	9606.ENSP00000306245	9606.ENSP00000364907	0	0.064	0.9	0.902
FOS	LPAR3	9606.ENSP00000306245	9606.ENSP00000395389	0	0	0.9	0.9
FOS	NFE2L2	9606.ENSP00000306245	9606.ENSP00000380252	0	0.056	0.9	0.901
FOS	TNF	9606.ENSP00000306245	9606.ENSP00000398698	0	0	0.9	0.9
FOS	HIF1A	9606.ENSP00000306245	9606.ENSP00000437955	0	0.405	0.9	0.938
FOS	MAPK8	9606.ENSP00000306245	9606.ENSP00000378974	0	0.243	0.9	0.921
FOS	RELA	9606.ENSP00000306245	9606.ENSP00000384273	0	0.659	0.9	0.964

FOS	JUN	9606.ENSP00000306245	9606.ENSP00000360266	0.656	0.985	0.9	0.999
G6PD	GCK	9606.ENSP00000377192	9606.ENSP00000223366	0.065	0	0.8	0.805
G6PD	PKM	9606.ENSP00000377192	9606.ENSP00000320171	0.144	0	0.8	0.821
G6PD	TGM2	9606.ENSP00000377192	9606.ENSP00000355330	0	0.484	0	0.484
G6PD	LDHA	9606.ENSP00000377192	9606.ENSP00000445175	0.065	0	0.8	0.805
GABBR1	GABBR2	9606.ENSP00000366233	9606.ENSP00000259455	0.088	0.989	0.8	0.998
GABBR1	TAS1R1	9606.ENSP00000366233	9606.ENSP00000331867	0	0.133	0.6	0.638
GABBR1	GNAI1	9606.ENSP00000366233	9606.ENSP00000343027	0.062	0	0.8	0.804
GABBR1	GRM1	9606.ENSP00000366233	9606.ENSP00000354896	0.11	0.636	0.72	0.901
GABBR1	GNAI3	9606.ENSP00000366233	9606.ENSP00000358867	0.062	0	0.8	0.804
GABBR1	TAS2R31	9606.ENSP00000366233	9606.ENSP00000375093	0	0	0.6	0.6
GABBR1	GRM4	9606.ENSP00000366233	9606.ENSP00000440556	0.098	0.158	0.6	0.669
GABBR1	GRM2	9606.ENSP00000366233	9606.ENSP00000378492	0.109	0.158	0.6	0.674
GABBR2	GRM5	9606.ENSP00000259455	9606.ENSP00000306138	0.293	0.255	0	0.451
GABBR2	TAS2R31	9606.ENSP00000259455	9606.ENSP00000375093	0	0	0.6	0.6
GABBR2	TAS1R1	9606.ENSP00000259455	9606.ENSP00000331867	0	0.133	0.6	0.638
GABBR2	GRM2	9606.ENSP00000259455	9606.ENSP00000378492	0.087	0.158	0.6	0.665
GABBR2	GRM4	9606.ENSP00000259455	9606.ENSP00000440556	0.159	0.158	0.6	0.692
GABBR2	GABRA6	9606.ENSP00000259455	9606.ENSP00000274545	0.228	0.073	0.54	0.642
GABBR2	GABRA3	9606.ENSP00000259455	9606.ENSP00000359337	0.113	0.073	0.54	0.589
GABBR2	GABRA5	9606.ENSP00000259455	9606.ENSP00000335592	0.214	0.073	0.54	0.635
GABBR2	GNAI3	9606.ENSP00000259455	9606.ENSP00000358867	0.062	0	0.8	0.804
GABBR2	GNAI1	9606.ENSP00000259455	9606.ENSP00000343027	0.08	0	0.8	0.808
GABBR2	GRM1	9606.ENSP00000259455	9606.ENSP00000354896	0.164	0.369	0.72	0.839
GABBR2	GABRA2	9606.ENSP00000259455	9606.ENSP00000421828	0.121	0.073	0.72	0.752
GABBR2	GABRA1	9606.ENSP00000259455	9606.ENSP00000393097	0.231	0.073	0.72	0.783
GABBR2	GABRB3	9606.ENSP00000259455	9606.ENSP00000308725	0.131	0.06	0.72	0.751
GABBR2	GABRG2	9606.ENSP00000259455	9606.ENSP00000410732	0.232	0.139	0.72	0.798
GABBR2	GABRB2	9606.ENSP00000259455	9606.ENSP00000274547	0.201	0.06	0.72	0.771
GABRA1	PRKCG	9606.ENSP00000393097	9606.ENSP00000263431	0.314	0.27	0	0.478
GABRA1	GABRA6	9606.ENSP00000393097	9606.ENSP00000274545	0.476	0.151	0.9	0.951
GABRA1	GABRB2	9606.ENSP00000393097	9606.ENSP00000274547	0.348	0.833	0.72	0.966
GABRA1	HTR5A	9606.ENSP00000393097	9606.ENSP00000287907	0.532	0	0	0.532
GABRA1	GABRB3	9606.ENSP00000393097	9606.ENSP00000308725	0.172	0.892	0.9	0.99
GABRA1	GABRA5	9606.ENSP00000393097	9606.ENSP00000335592	0.232	0.182	0.9	0.931
GABRA1	GABRA3	9606.ENSP00000393097	9606.ENSP00000359337	0.144	0.182	0.9	0.923
GABRA1	GLRA1	9606.ENSP00000393097	9606.ENSP00000411593	0.062	0	0.54	0.55
GABRA1	GABRA2	9606.ENSP00000393097	9606.ENSP00000421828	0.197	0	0.9	0.916
GABRA1	GABRG2	9606.ENSP00000393097	9606.ENSP00000410732	0.546	0.897	0.72	0.985
GABRA2	GABRA6	9606.ENSP00000421828	9606.ENSP00000274545	0.159	0	0.9	0.912
GABRA2	GABRB2	9606.ENSP00000421828	9606.ENSP00000274547	0.176	0.196	0.72	0.798
GABRA2	GABRB3	9606.ENSP00000421828	9606.ENSP00000308725	0.116	0.196	0.72	0.783
GABRA2	GABRA5	9606.ENSP00000421828	9606.ENSP00000335592	0.124	0	0.9	0.908
GABRA2	GABRA3	9606.ENSP00000421828	9606.ENSP00000359337	0.14	0	0.9	0.91

GABRA2	GABRG2	9606.ENSP00000421828	9606.ENSP00000410732	0.224	0.412	0.72	0.861
GABRA2	GLRA1	9606.ENSP00000421828	9606.ENSP00000411593	0.063	0	0.54	0.55
GABRA3	GABRA6	9606.ENSP00000359337	9606.ENSP00000274545	0.109	0	0.9	0.907
GABRA3	GABRB2	9606.ENSP00000359337	9606.ENSP00000274547	0.117	0.196	0.6	0.691
GABRA3	GABRB3	9606.ENSP00000359337	9606.ENSP00000308725	0.118	0.196	0.6	0.691
GABRA3	GABRA5	9606.ENSP00000359337	9606.ENSP00000335592	0.13	0.323	0.9	0.936
GABRA3	GLRA1	9606.ENSP00000359337	9606.ENSP00000411593	0.062	0	0.54	0.55
GABRA3	GABRG2	9606.ENSP00000359337	9606.ENSP00000410732	0.196	0.181	0.6	0.713
GABRA5	GABRA6	9606.ENSP00000335592	9606.ENSP00000274545	0.183	0.151	0.9	0.924
GABRA5	GABRB2	9606.ENSP00000335592	9606.ENSP00000274547	0.231	0.196	0.6	0.731
GABRA5	GABRB3	9606.ENSP00000335592	9606.ENSP00000308725	0.231	0.196	0.6	0.731
GABRA5	GLRA1	9606.ENSP00000335592	9606.ENSP00000411593	0.062	0	0.54	0.55
GABRA5	GABRG2	9606.ENSP00000335592	9606.ENSP00000410732	0.335	0.181	0.6	0.763
GABRA6	GRIN1	9606.ENSP00000274545	9606.ENSP00000360608	0.426	0	0	0.426
GABRA6	HTR5A	9606.ENSP00000274545	9606.ENSP00000287907	0.557	0	0	0.557
GABRA6	GLRA1	9606.ENSP00000274545	9606.ENSP00000411593	0.064	0	0.54	0.551
GABRA6	GABRB3	9606.ENSP00000274545	9606.ENSP00000308725	0.105	0.311	0.6	0.732
GABRA6	GABRG2	9606.ENSP00000274545	9606.ENSP00000410732	0.434	0.181	0.6	0.798
GABRA6	GABRB2	9606.ENSP00000274545	9606.ENSP00000274547	0.458	0.196	0.6	0.81
GABRB2	GLRA1	9606.ENSP00000274547	9606.ENSP00000411593	0.079	0	0.54	0.558
GABRB2	HTR5A	9606.ENSP00000274547	9606.ENSP00000287907	0.552	0	0	0.552
GABRB2	GABRB3	9606.ENSP00000274547	9606.ENSP00000308725	0.157	0.179	0.8	0.849
GABRB2	GABRG2	9606.ENSP00000274547	9606.ENSP00000410732	0.426	0.393	0.72	0.894
GABRB3	GLRA1	9606.ENSP00000308725	9606.ENSP00000411593	0.079	0	0.54	0.558
GABRB3	GABRG2	9606.ENSP00000308725	9606.ENSP00000410732	0.175	0.681	0.72	0.919
GABRG2	HTR5A	9606.ENSP00000410732	9606.ENSP00000287907	0.533	0	0	0.533
GABRG2	GRIA2	9606.ENSP00000410732	9606.ENSP00000296526	0.465	0	0	0.465
GABRG2	GRM5	9606.ENSP00000410732	9606.ENSP00000306138	0.404	0	0	0.404
GABRG2	GRIN1	9606.ENSP00000410732	9606.ENSP00000360608	0.448	0	0	0.448
GABRG2	GLRA1	9606.ENSP00000410732	9606.ENSP00000411593	0.063	0	0.54	0.55
GAK	LRRK2	9606.ENSP00000314499	9606.ENSP00000298910	0.109	0.679	0	0.702
GAK	GBA2	9606.ENSP00000314499	9606.ENSP00000367343	0.066	0.61	0	0.62
GBA	GLB1	9606.ENSP00000314508	9606.ENSP00000306920	0.102	0.389	0.9	0.94
GBA	GBA2	9606.ENSP00000314508	9606.ENSP00000367343	0	0	0.8	0.8
GBA2	GLB1	9606.ENSP00000367343	9606.ENSP00000306920	0	0	0.9	0.9
GCGR	GNAI1	9606.ENSP00000383558	9606.ENSP00000343027	0	0.826	0	0.826
GCK	GLB1	9606.ENSP00000223366	9606.ENSP00000306920	0	0	0.9	0.9
GCK	HDAC2	9606.ENSP00000223366	9606.ENSP00000430432	0.082	0	0.9	0.904
GCK	HDAC1	9606.ENSP00000223366	9606.ENSP00000362649	0.082	0	0.9	0.904
GGPS1	SREBF2	9606.ENSP00000282841	9606.ENSP00000354476	0	0	0.9	0.9
GLI1	GNAI3	9606.ENSP00000228682	9606.ENSP00000358867	0	0.056	0.9	0.901
GLI1	PSMB5	9606.ENSP00000228682	9606.ENSP00000355325	0	0.065	0.9	0.902
GLI1	PSMB8	9606.ENSP00000228682	9606.ENSP00000364016	0	0.065	0.9	0.902
GLI1	GNAO1	9606.ENSP00000228682	9606.ENSP00000262494	0	0.056	0.9	0.901

GLI1	GNAI1	9606.ENSP00000228682	9606.ENSP00000343027	0	0.281	0.9	0.925
GLI1	XPO1	9606.ENSP00000228682	9606.ENSP00000384863	0.062	0.131	0.9	0.911
GLI1	GLI2	9606.ENSP00000228682	9606.ENSP00000390436	0.064	0.389	0.9	0.937
GLI1	HDAC2	9606.ENSP00000228682	9606.ENSP00000430432	0	0.358	0.9	0.933
GLI1	HDAC1	9606.ENSP00000228682	9606.ENSP00000362649	0	0.358	0.9	0.933
GLI2	SHH	9606.ENSP00000390436	9606.ENSP00000297261	0.062	0.258	0.9	0.924
GLI2	MAP2K1	9606.ENSP00000390436	9606.ENSP00000302486	0.055	0.057	0.9	0.903
GLI2	GSK3B	9606.ENSP00000390436	9606.ENSP00000324806	0.063	0.147	0.9	0.913
GLI2	PSMB5	9606.ENSP00000390436	9606.ENSP00000355325	0	0.065	0.9	0.902
GLI2	PSMB8	9606.ENSP00000390436	9606.ENSP00000364016	0	0.065	0.9	0.902
GLI2	PRKCD	9606.ENSP00000390436	9606.ENSP00000378217	0.063	0.057	0.9	0.903
GLO1	NDUFB10	9606.ENSP00000362463	9606.ENSP00000268668	0.82	0	0	0.82
GLO1	MAOA	9606.ENSP00000362463	9606.ENSP00000340684	0	0	0.8	0.8
GLO1	MAOB	9606.ENSP00000362463	9606.ENSP00000367309	0	0	0.8	0.8
GNAI1	MAPK1	9606.ENSP00000343027	9606.ENSP00000215832	0.078	0.254	0.9	0.925
GNAI1	MAPK14	9606.ENSP00000343027	9606.ENSP00000229795	0.078	0.247	0.9	0.924
GNAI1	OPRD1	9606.ENSP00000343027	9606.ENSP00000234961	0	0.303	0.8	0.854
GNAI1	PTGER2	9606.ENSP00000343027	9606.ENSP00000245457	0	0.151	0.6	0.645
GNAI1	S1PR4	9606.ENSP00000343027	9606.ENSP00000246115	0	0.213	0.9	0.917
GNAI1	MTNR1B	9606.ENSP00000343027	9606.ENSP00000257068	0	0.274	0.6	0.697
GNAI1	GNAO1	9606.ENSP00000343027	9606.ENSP00000262494	0.085	0.68	0.9	0.968
GNAI1	MAPK3	9606.ENSP00000343027	9606.ENSP00000263025	0.078	0.32	0.9	0.931
GNAI1	PRKCG	9606.ENSP00000343027	9606.ENSP00000263431	0.071	0.078	0.6	0.627
GNAI1	PIK3CA	9606.ENSP00000343027	9606.ENSP00000263967	0.056	0.06	0.9	0.903
GNAI1	IGF1R	9606.ENSP00000343027	9606.ENSP00000268035	0.049	0.359	0.8	0.867
GNAI1	MMP16	9606.ENSP00000343027	9606.ENSP00000286614	0.093	0	0.6	0.621
GNAI1	HTR5A	9606.ENSP00000343027	9606.ENSP00000287907	0.075	0.151	0.6	0.658
GNAI1	PIK3CB	9606.ENSP00000343027	9606.ENSP00000289153	0	0.06	0.6	0.607
GNAI1	PTGER1	9606.ENSP00000343027	9606.ENSP00000292513	0	0.151	0.6	0.645
GNAI1	NOS3	9606.ENSP00000343027	9606.ENSP00000297494	0.062	0.163	0.9	0.914
GNAI1	LRRK2	9606.ENSP00000343027	9606.ENSP00000298910	0.063	0.525	0	0.535
GNAI1	MTNR1A	9606.ENSP00000343027	9606.ENSP00000302811	0.053	0.274	0.6	0.7
GNAI1	PRKCB	9606.ENSP00000343027	9606.ENSP00000305355	0	0.078	0.6	0.615
GNAI1	S1PR1	9606.ENSP00000343027	9606.ENSP00000305416	0	0.27	0.9	0.923
GNAI1	PRKCE	9606.ENSP00000343027	9606.ENSP00000306124	0.062	0.069	0.6	0.62
GNAI1	HTR1E	9606.ENSP00000343027	9606.ENSP00000307766	0	0.151	0.6	0.645
GNAI1	MMP14	9606.ENSP00000343027	9606.ENSP00000308208	0.062	0	0.6	0.608
GNAI1	HTR1A	9606.ENSP00000343027	9606.ENSP00000316244	0.062	0.151	0.6	0.653
GNAI1	OXTR	9606.ENSP00000343027	9606.ENSP00000324270	0	0.151	0.8	0.822
GNAI1	S1PR5	9606.ENSP00000343027	9606.ENSP00000328472	0	0.213	0.9	0.917
GNAI1	LCK	9606.ENSP00000343027	9606.ENSP00000337825	0	0.159	0.9	0.912
GNAI1	PTPN11	9606.ENSP00000343027	9606.ENSP00000340944	0.062	0.068	0.9	0.904
GNAI1	PTK2	9606.ENSP00000343027	9606.ENSP00000341189	0.089	0.056	0.9	0.906
GNAI1	PIK3CD	9606.ENSP00000343027	9606.ENSP00000366563	0	0.06	0.6	0.607

GNAI1	HTR1D	9606.ENSP00000343027	9606.ENSP00000363748	0	0.151	0.6	0.645
GNAI1	HCK	9606.ENSP00000343027	9606.ENSP00000444986	0	0.159	0.6	0.649
GNAI1	PLA2G4A	9606.ENSP00000343027	9606.ENSP00000356436	0.062	0.146	0.6	0.651
GNAI1	HCAR2	9606.ENSP00000343027	9606.ENSP00000375066	0	0.151	0.6	0.645
GNAI1	MAPK10	9606.ENSP00000343027	9606.ENSP00000352157	0.096	0	0.6	0.623
GNAI1	PLA2G4B	9606.ENSP00000343027	9606.ENSP00000396045	0.062	0.162	0.6	0.658
GNAI1	LYN	9606.ENSP00000343027	9606.ENSP00000428924	0.053	0.159	0.6	0.653
GNAI1	HTR1B	9606.ENSP00000343027	9606.ENSP00000358963	0.072	0.151	0.6	0.657
GNAI1	PRKCA	9606.ENSP00000343027	9606.ENSP00000408695	0	0.078	0.6	0.615
GNAI1	GRM2	9606.ENSP00000343027	9606.ENSP00000378492	0.052	0	0.8	0.802
GNAI1	GRM1	9606.ENSP00000343027	9606.ENSP00000354896	0.074	0	0.8	0.807
GNAI1	PIK3CG	9606.ENSP00000343027	9606.ENSP00000352121	0	0.06	0.8	0.803
GNAI1	OPRM1	9606.ENSP00000343027	9606.ENSP00000394624	0	0.303	0.8	0.854
GNAI1	MAPK8	9606.ENSP00000343027	9606.ENSP00000378974	0	0	0.9	0.9
GNAI1	PIK3R1	9606.ENSP00000343027	9606.ENSP00000428056	0	0	0.9	0.9
GNAI1	PTK2B	9606.ENSP00000343027	9606.ENSP00000380638	0.062	0.056	0.9	0.903
GNAI1	SRC	9606.ENSP00000343027	9606.ENSP00000362680	0.062	0.159	0.9	0.914
GNAI1	S1PR3	9606.ENSP00000343027	9606.ENSP00000365006	0	0.213	0.9	0.917
GNAI1	S1PR2	9606.ENSP00000343027	9606.ENSP00000466933	0.054	0.213	0.9	0.919
GNAI1	RAC1	9606.ENSP00000343027	9606.ENSP00000348461	0	0.352	0.9	0.932
GNAI1	LPAR1	9606.ENSP00000343027	9606.ENSP00000363553	0.076	0.151	0.9	0.914
GNAI1	GNAI3	9606.ENSP00000343027	9606.ENSP00000358867	0	0.87	0.9	0.986
GNAI1	RGS4	9606.ENSP00000343027	9606.ENSP00000397181	0.121	0.944	0.6	0.978
GNAI3	MAPK1	9606.ENSP00000358867	9606.ENSP00000215832	0.078	0.254	0.9	0.925
GNAI3	MAPK14	9606.ENSP00000358867	9606.ENSP00000229795	0.078	0.247	0.9	0.924
GNAI3	OPRD1	9606.ENSP00000358867	9606.ENSP00000234961	0	0.151	0.8	0.822
GNAI3	PTGER2	9606.ENSP00000358867	9606.ENSP00000245457	0	0.151	0.6	0.645
GNAI3	S1PR4	9606.ENSP00000358867	9606.ENSP00000246115	0	0	0.9	0.9
GNAI3	MTNR1B	9606.ENSP00000358867	9606.ENSP00000257068	0	0.274	0.6	0.697
GNAI3	GNAO1	9606.ENSP00000358867	9606.ENSP00000262494	0.062	0.468	0.9	0.945
GNAI3	MAPK3	9606.ENSP00000358867	9606.ENSP00000263025	0.078	0.32	0.9	0.931
GNAI3	PRKCG	9606.ENSP00000358867	9606.ENSP00000263431	0.048	0.078	0.6	0.618
GNAI3	PIK3CA	9606.ENSP00000358867	9606.ENSP00000263967	0.062	0.145	0.6	0.651
GNAI3	IGF1R	9606.ENSP00000358867	9606.ENSP00000268035	0.049	0.159	0.8	0.826
GNAI3	MMP16	9606.ENSP00000358867	9606.ENSP00000286614	0.062	0	0.6	0.608
GNAI3	HTR5A	9606.ENSP00000358867	9606.ENSP00000287907	0	0.151	0.6	0.645
GNAI3	PIK3CB	9606.ENSP00000358867	9606.ENSP00000289153	0	0.06	0.6	0.607
GNAI3	PTGER1	9606.ENSP00000358867	9606.ENSP00000292513	0	0.151	0.6	0.645
GNAI3	NOS3	9606.ENSP00000358867	9606.ENSP00000297494	0.062	0.163	0.9	0.914
GNAI3	LRRK2	9606.ENSP00000358867	9606.ENSP00000298910	0.063	0.638	0	0.646
GNAI3	MTNR1A	9606.ENSP00000358867	9606.ENSP00000302811	0.053	0.508	0.6	0.797
GNAI3	PRKCB	9606.ENSP00000358867	9606.ENSP00000305355	0	0.078	0.6	0.615
GNAI3	S1PR1	9606.ENSP00000358867	9606.ENSP00000305416	0	0.27	0.9	0.923
GNAI3	PRKCE	9606.ENSP00000358867	9606.ENSP00000306124	0.062	0.069	0.6	0.62

GNAI3	HTR1E	9606.ENSP00000358867	9606.ENSP00000307766	0	0.151	0.6	0.645
GNAI3	MMP14	9606.ENSP00000358867	9606.ENSP00000308208	0.062	0	0.6	0.608
GNAI3	HTR1A	9606.ENSP00000358867	9606.ENSP00000316244	0.062	0.303	0.6	0.715
GNAI3	OXTR	9606.ENSP00000358867	9606.ENSP00000324270	0	0.151	0.8	0.822
GNAI3	S1PR5	9606.ENSP00000358867	9606.ENSP00000328472	0	0	0.9	0.9
GNAI3	LCK	9606.ENSP00000358867	9606.ENSP00000337825	0	0.159	0.9	0.912
GNAI3	PTPN11	9606.ENSP00000358867	9606.ENSP00000340944	0.097	0.068	0.9	0.908
GNAI3	PTK2	9606.ENSP00000358867	9606.ENSP00000341189	0.062	0.056	0.9	0.903
GNAI3	RAC1	9606.ENSP00000358867	9606.ENSP00000348461	0	0.352	0.9	0.932
GNAI3	PIK3CG	9606.ENSP00000358867	9606.ENSP00000352121	0	0.06	0.8	0.803
GNAI3	MAPK10	9606.ENSP00000358867	9606.ENSP00000352157	0.05	0	0.6	0.603
GNAI3	GRM1	9606.ENSP00000358867	9606.ENSP00000354896	0.051	0	0.8	0.802
GNAI3	PLA2G4A	9606.ENSP00000358867	9606.ENSP00000356436	0.062	0.146	0.6	0.651
GNAI3	PIK3CD	9606.ENSP00000358867	9606.ENSP00000366563	0	0.06	0.6	0.607
GNAI3	PIK3R1	9606.ENSP00000358867	9606.ENSP00000428056	0	0	0.6	0.6
GNAI3	PRKCA	9606.ENSP00000358867	9606.ENSP00000408695	0	0.078	0.6	0.615
GNAI3	HTR1D	9606.ENSP00000358867	9606.ENSP00000363748	0	0.151	0.6	0.645
GNAI3	HCK	9606.ENSP00000358867	9606.ENSP00000444986	0	0.159	0.6	0.649
GNAI3	HCAR2	9606.ENSP00000358867	9606.ENSP00000375066	0	0.151	0.6	0.645
GNAI3	PLA2G4B	9606.ENSP00000358867	9606.ENSP00000396045	0.062	0.162	0.6	0.658
GNAI3	HTR1B	9606.ENSP00000358867	9606.ENSP00000358963	0.049	0.151	0.6	0.648
GNAI3	LYN	9606.ENSP00000358867	9606.ENSP00000428924	0.053	0.159	0.6	0.653
GNAI3	RGS4	9606.ENSP00000358867	9606.ENSP00000397181	0.066	0.211	0.6	0.679
GNAI3	GRM2	9606.ENSP00000358867	9606.ENSP00000378492	0.052	0	0.8	0.802
GNAI3	OPRM1	9606.ENSP00000358867	9606.ENSP00000394624	0	0.151	0.8	0.822
GNAI3	S1PR3	9606.ENSP00000358867	9606.ENSP00000365006	0	0	0.9	0.9
GNAI3	S1PR2	9606.ENSP00000358867	9606.ENSP00000466933	0.054	0	0.9	0.901
GNAI3	MAPK8	9606.ENSP00000358867	9606.ENSP00000378974	0	0	0.9	0.9
GNAI3	SRC	9606.ENSP00000358867	9606.ENSP00000362680	0.062	0.159	0.9	0.914
GNAI3	LPAR1	9606.ENSP00000358867	9606.ENSP00000363553	0	0.303	0.9	0.927
GNAO1	MAPK1	9606.ENSP00000262494	9606.ENSP00000215832	0.078	0.247	0.9	0.924
GNAO1	MAPK14	9606.ENSP00000262494	9606.ENSP00000229795	0.078	0.247	0.9	0.924
GNAO1	OPRD1	9606.ENSP00000262494	9606.ENSP00000234961	0.062	0.523	0	0.534
GNAO1	S1PR4	9606.ENSP00000262494	9606.ENSP00000246115	0	0	0.9	0.9
GNAO1	LRRK2	9606.ENSP00000262494	9606.ENSP00000298910	0.063	0.525	0	0.535
GNAO1	OPRM1	9606.ENSP00000262494	9606.ENSP00000394624	0	0.353	0.6	0.73
GNAO1	HTR1B	9606.ENSP00000262494	9606.ENSP00000358963	0.085	0.776	0	0.786
GNAO1	PTK2	9606.ENSP00000262494	9606.ENSP00000341189	0.062	0.056	0.9	0.903
GNAO1	S1PR2	9606.ENSP00000262494	9606.ENSP00000466933	0	0	0.9	0.9
GNAO1	MAPK8	9606.ENSP00000262494	9606.ENSP00000378974	0.051	0	0.9	0.901
GNAO1	LCK	9606.ENSP00000262494	9606.ENSP00000337825	0	0.159	0.9	0.912
GNAO1	S1PR3	9606.ENSP00000262494	9606.ENSP00000365006	0	0	0.9	0.9
GNAO1	NOS3	9606.ENSP00000262494	9606.ENSP00000297494	0.062	0.163	0.9	0.914
GNAO1	LPAR1	9606.ENSP00000262494	9606.ENSP00000363553	0	0.151	0.9	0.911

GNAO1	S1PR1	9606.ENSP00000262494	9606.ENSP00000305416	0	0	0.9	0.9
GNAO1	SRC	9606.ENSP00000262494	9606.ENSP00000362680	0.062	0.159	0.9	0.914
GNAO1	RAC1	9606.ENSP00000262494	9606.ENSP00000348461	0	0.352	0.9	0.932
GNAO1	MAPK3	9606.ENSP00000262494	9606.ENSP00000263025	0.078	0.247	0.9	0.924
GNAO1	S1PR5	9606.ENSP00000262494	9606.ENSP00000328472	0.07	0.213	0.9	0.92
GNAO1	RGS4	9606.ENSP00000262494	9606.ENSP00000397181	0.122	0.577	0	0.613
GPR174	P2RY10	9606.ENSP00000276077	9606.ENSP00000171757	0.691	0	0	0.691
GPR174	P2RX7	9606.ENSP00000276077	9606.ENSP00000330696	0.512	0	0	0.512
GPR174	PTPRC	9606.ENSP00000276077	9606.ENSP00000411355	0.5	0	0	0.5
GPR174	ITGB2	9606.ENSP00000276077	9606.ENSP00000380948	0.568	0	0	0.568
GPR174	ITK	9606.ENSP00000276077	9606.ENSP00000398655	0.676	0	0	0.676
GPR18	MTNR1B	9606.ENSP00000343428	9606.ENSP00000257068	0	0	0.6	0.6
GPR18	MTNR1A	9606.ENSP00000343428	9606.ENSP00000302811	0	0	0.6	0.6
GPR18	HCAR2	9606.ENSP00000343428	9606.ENSP00000375066	0.062	0	0.6	0.608
GRIA1	MDM2	9606.ENSP00000428994	9606.ENSP00000258149	0	0	0.9	0.9
GRIA1	PRKCG	9606.ENSP00000428994	9606.ENSP00000263431	0.159	0.067	0.9	0.914
GRIA1	GRIA2	9606.ENSP00000428994	9606.ENSP00000296526	0.359	0.988	0.8	0.998
GRIA1	PRKCB	9606.ENSP00000428994	9606.ENSP00000305355	0.065	0.067	0.9	0.905
GRIA1	PPP1CC	9606.ENSP00000428994	9606.ENSP00000341779	0	0.069	0.6	0.611
GRIA1	HTR3A	9606.ENSP00000428994	9606.ENSP00000347754	0.064	0.057	0.72	0.731
GRIA1	GRIN1	9606.ENSP00000428994	9606.ENSP00000360608	0.212	0.182	0.72	0.803
GRIA1	PTK2B	9606.ENSP00000428994	9606.ENSP00000380638	0.062	0.07	0.54	0.563
GRIA1	PRKCA	9606.ENSP00000428994	9606.ENSP00000408695	0.063	0.477	0.9	0.946
GRIA1	LYN	9606.ENSP00000428994	9606.ENSP00000428924	0	0.06	0.8	0.803
GRIA1	GRIN2B	9606.ENSP00000428994	9606.ENSP00000477455	0.218	0.143	0.72	0.796
GRIA2	PRKCG	9606.ENSP00000296526	9606.ENSP00000263431	0.231	0.067	0.9	0.922
GRIA2	HTR5A	9606.ENSP00000296526	9606.ENSP00000287907	0.479	0.056	0	0.487
GRIA2	PPP1CC	9606.ENSP00000296526	9606.ENSP00000341779	0	0.069	0.6	0.611
GRIA2	PTK2B	9606.ENSP00000296526	9606.ENSP00000380638	0.062	0.07	0.54	0.563
GRIA2	MAPK10	9606.ENSP00000296526	9606.ENSP00000352157	0.348	0.153	0	0.424
GRIA2	HTR3A	9606.ENSP00000296526	9606.ENSP00000347754	0.062	0.057	0.72	0.73
GRIA2	GRM5	9606.ENSP00000296526	9606.ENSP00000306138	0.41	0.076	0	0.431
GRIA2	LYN	9606.ENSP00000296526	9606.ENSP00000428924	0	0.06	0.8	0.803
GRIA2	HDAC1	9606.ENSP00000296526	9606.ENSP00000362649	0	0	0.9	0.9
GRIA2	HDAC2	9606.ENSP00000296526	9606.ENSP00000430432	0	0	0.9	0.9
GRIA2	PRKCB	9606.ENSP00000296526	9606.ENSP00000305355	0.087	0.067	0.9	0.907
GRIA2	GRIN2B	9606.ENSP00000296526	9606.ENSP00000477455	0.284	0.102	0.9	0.93
GRIA2	GRIN1	9606.ENSP00000296526	9606.ENSP00000360608	0.377	0.168	0.9	0.943
GRIA2	PRKCA	9606.ENSP00000296526	9606.ENSP00000408695	0.062	0.234	0.9	0.921
GRIN1	P2RX3	9606.ENSP00000360608	9606.ENSP00000263314	0	0	0.8	0.8
GRIN1	PRKCG	9606.ENSP00000360608	9606.ENSP00000263431	0.347	0.403	0	0.594
GRIN1	HTR5A	9606.ENSP00000360608	9606.ENSP00000287907	0.535	0.078	0	0.553
GRIN1	PRKCB	9606.ENSP00000360608	9606.ENSP00000305355	0.089	0.403	0	0.433
GRIN1	PRKCE	9606.ENSP00000360608	9606.ENSP00000306124	0.107	0.403	0	0.444

GRIN1	GRM5	9606.ENSP00000360608	9606.ENSP00000306138	0.428	0.25	0	0.553
GRIN1	P2RX7	9606.ENSP00000360608	9606.ENSP00000330696	0	0	0.8	0.8
GRIN1	MAPT	9606.ENSP00000360608	9606.ENSP00000340820	0.298	0.204	0	0.417
GRIN1	PPP1CC	9606.ENSP00000360608	9606.ENSP00000341779	0.062	0.145	0.6	0.651
GRIN1	HRH3	9606.ENSP00000360608	9606.ENSP00000342560	0.377	0.078	0	0.401
GRIN1	HTR3A	9606.ENSP00000360608	9606.ENSP00000347754	0.077	0	0.72	0.73
GRIN1	RAC1	9606.ENSP00000360608	9606.ENSP00000348461	0	0.213	0.9	0.917
GRIN1	GRM1	9606.ENSP00000360608	9606.ENSP00000354896	0.32	0.368	0	0.552
GRIN1	PLA2G4A	9606.ENSP00000360608	9606.ENSP00000356436	0	0.532	0	0.532
GRIN1	RPS6KA3	9606.ENSP00000360608	9606.ENSP00000368884	0	0.425	0	0.425
GRIN1	LYN	9606.ENSP00000360608	9606.ENSP00000428924	0	0.119	0.6	0.632
GRIN1	GRM4	9606.ENSP00000360608	9606.ENSP00000440556	0.306	0.172	0	0.401
GRIN1	PTK2B	9606.ENSP00000360608	9606.ENSP00000380638	0.087	0.368	0.54	0.711
GRIN1	YES1	9606.ENSP00000360608	9606.ENSP00000462468	0.062	0.33	0.6	0.726
GRIN1	SRC	9606.ENSP00000360608	9606.ENSP00000362680	0.062	0.386	0.6	0.749
GRIN1	NOS1	9606.ENSP00000360608	9606.ENSP00000477999	0.063	0.394	0.8	0.876
GRIN1	GRIN2B	9606.ENSP00000360608	9606.ENSP00000477455	0.269	0.992	0.9	0.999
GRIN2B	PRKCG	9606.ENSP00000477455	9606.ENSP00000263431	0.162	0.436	0	0.507
GRIN2B	PRKCB	9606.ENSP00000477455	9606.ENSP00000305355	0.086	0.436	0	0.462
GRIN2B	GRM5	9606.ENSP00000477455	9606.ENSP00000306138	0.316	0.177	0	0.413
GRIN2B	PTPN11	9606.ENSP00000477455	9606.ENSP00000340944	0	0.444	0	0.444
GRIN2B	PPP1CC	9606.ENSP00000477455	9606.ENSP00000341779	0	0.137	0.8	0.82
GRIN2B	HTR3A	9606.ENSP00000477455	9606.ENSP00000347754	0.064	0.057	0.72	0.731
GRIN2B	RAC1	9606.ENSP00000477455	9606.ENSP00000348461	0	0.152	0.9	0.911
GRIN2B	GRM1	9606.ENSP00000477455	9606.ENSP00000354896	0.195	0.368	0	0.469
GRIN2B	SRC	9606.ENSP00000477455	9606.ENSP00000362680	0.062	0.436	0.6	0.769
GRIN2B	PTK2B	9606.ENSP00000477455	9606.ENSP00000380638	0.076	0.368	0.54	0.707
GRIN2B	PRKCA	9606.ENSP00000477455	9606.ENSP00000408695	0	0.527	0	0.527
GRIN2B	LYN	9606.ENSP00000477455	9606.ENSP00000428924	0	0.06	0.6	0.607
GRIN2B	YES1	9606.ENSP00000477455	9606.ENSP00000462468	0.062	0.244	0.6	0.691
GRIN2B	NOS1	9606.ENSP00000477455	9606.ENSP00000477999	0.096	0.393	0.8	0.88
GRM1	RORA	9606.ENSP00000354896	9606.ENSP00000261523	0.062	0	0.8	0.804
GRM1	GRM5	9606.ENSP00000354896	9606.ENSP00000306138	0.347	0.787	0.8	0.969
GRM1	GRM2	9606.ENSP00000354896	9606.ENSP00000378492	0.122	0	0.5	0.542
GRM1	GRM4	9606.ENSP00000354896	9606.ENSP00000440556	0.183	0	0.8	0.829
GRM2	GRM5	9606.ENSP00000378492	9606.ENSP00000306138	0.114	0	0.5	0.538
GRM2	TAS1R1	9606.ENSP00000378492	9606.ENSP00000331867	0	0	0.6	0.6
GRM2	TAS2R31	9606.ENSP00000378492	9606.ENSP00000375093	0	0	0.6	0.6
GRM2	HTR2A	9606.ENSP00000378492	9606.ENSP00000437737	0.075	0.392	0	0.414
GRM4	GRM5	9606.ENSP00000440556	9606.ENSP00000306138	0.164	0	0.5	0.564
GRM4	TAS1R1	9606.ENSP00000440556	9606.ENSP00000331867	0	0	0.6	0.6
GRM4	TAS2R31	9606.ENSP00000440556	9606.ENSP00000375093	0	0	0.6	0.6
GRM5	PRKCG	9606.ENSP00000306138	9606.ENSP00000263431	0.346	0.227	0	0.473
GRM5	HTR5A	9606.ENSP00000306138	9606.ENSP00000287907	0.533	0.056	0	0.54

GRM5	PRKCA	9606.ENSP00000306138	9606.ENSP00000408695	0.062	0.476	0	0.488
GRM5	PRKCD	9606.ENSP00000306138	9606.ENSP00000378217	0	0.606	0	0.606
GRM5	PRKCZ	9606.ENSP00000306138	9606.ENSP00000367830	0.097	0.612	0	0.634
GSK3A	PRKCG	9606.ENSP00000222330	9606.ENSP00000263431	0	0.474	0	0.474
GSK3A	PRKCB	9606.ENSP00000222330	9606.ENSP00000305355	0	0.474	0	0.474
GSK3A	PRKCA	9606.ENSP00000222330	9606.ENSP00000408695	0	0.474	0	0.474
GSK3A	PRKCD	9606.ENSP00000222330	9606.ENSP00000378217	0.063	0.505	0	0.516
GSK3A	YWHAG	9606.ENSP00000222330	9606.ENSP00000306330	0.08	0.481	0	0.502
GSK3A	MCL1	9606.ENSP00000222330	9606.ENSP00000358022	0	0.473	0	0.473
GSK3A	PDPK1	9606.ENSP00000222330	9606.ENSP00000344220	0.181	0.145	0.65	0.733
GSK3A	GSK3B	9606.ENSP00000222330	9606.ENSP00000324806	0.1	0.335	0.9	0.934
GSK3A	WNT3A	9606.ENSP00000222330	9606.ENSP00000284523	0.049	0.153	0.9	0.912
GSK3A	MAPT	9606.ENSP00000222330	9606.ENSP00000340820	0.049	0.942	0	0.942
GSK3B	MAPK1	9606.ENSP00000324806	9606.ENSP00000215832	0.288	0.351	0	0.519
GSK3B	RPS6KB1	9606.ENSP00000324806	9606.ENSP00000225577	0.064	0.091	0.9	0.907
GSK3B	NR3C1	9606.ENSP00000324806	9606.ENSP00000231509	0	0.077	0.9	0.903
GSK3B	PIN1	9606.ENSP00000324806	9606.ENSP00000247970	0.061	0.06	0.9	0.904
GSK3B	RAF1	9606.ENSP00000324806	9606.ENSP00000251849	0	0	0.8	0.8
GSK3B	WNT3A	9606.ENSP00000324806	9606.ENSP00000284523	0.049	0.153	0.9	0.912
GSK3B	LRRK2	9606.ENSP00000324806	9606.ENSP00000298910	0.062	0.532	0.72	0.866
GSK3B	MAP2K1	9606.ENSP00000324806	9606.ENSP00000302486	0.063	0.178	0.8	0.832
GSK3B	PRKCB	9606.ENSP00000324806	9606.ENSP00000305355	0.062	0.608	0	0.616
GSK3B	GYS1	9606.ENSP00000324806	9606.ENSP00000317904	0.114	0.783	0.9	0.979
GSK3B	NFE2L2	9606.ENSP00000324806	9606.ENSP00000380252	0	0.413	0	0.413
GSK3B	MCL1	9606.ENSP00000324806	9606.ENSP00000358022	0	0.476	0	0.476
GSK3B	IKBKG	9606.ENSP00000324806	9606.ENSP00000483825	0.048	0.683	0	0.686
GSK3B	PDPK1	9606.ENSP00000324806	9606.ENSP00000344220	0.128	0.145	0.65	0.716
GSK3B	HSP90AA1	9606.ENSP00000324806	9606.ENSP00000335153	0	0.64	0	0.64
GSK3B	PTK2B	9606.ENSP00000324806	9606.ENSP00000380638	0	0.078	0.9	0.903
GSK3B	PPP1CC	9606.ENSP00000324806	9606.ENSP00000341779	0.063	0.435	0.9	0.942
GSK3B	PRKCZ	9606.ENSP00000324806	9606.ENSP00000367830	0.062	0.653	0.9	0.964
GSK3B	JUN	9606.ENSP00000324806	9606.ENSP00000360266	0	0.691	0.9	0.967
GSK3B	PSEN1	9606.ENSP00000324806	9606.ENSP00000326366	0.089	0.79	0.8	0.958
GSK3B	MAPT	9606.ENSP00000324806	9606.ENSP00000340820	0.062	0.994	0.9	0.999
GSR	NDUFS1	9606.ENSP00000221130	9606.ENSP00000392709	0.344	0.175	0	0.435
GSR	NAMPT	9606.ENSP00000221130	9606.ENSP00000222553	0.063	0.484	0	0.496
GSR	HPGDS	9606.ENSP00000221130	9606.ENSP00000295256	0.064	0	0.65	0.658
GYS1	PYGL	9606.ENSP00000317904	9606.ENSP00000216392	0.512	0.104	0	0.544
GYS1	PASK	9606.ENSP00000317904	9606.ENSP00000351475	0.087	0.426	0	0.453
GYS1	PPP1CC	9606.ENSP00000317904	9606.ENSP00000341779	0.052	0.81	0.6	0.921
HCAR2	MTNR1B	9606.ENSP00000375066	9606.ENSP00000257068	0	0	0.6	0.6
HCAR2	MTNR1A	9606.ENSP00000375066	9606.ENSP00000302811	0	0	0.6	0.6
HCK	MAPK14	9606.ENSP00000444986	9606.ENSP00000229795	0	0.148	0.9	0.911
HCK	S1PR4	9606.ENSP00000444986	9606.ENSP00000246115	0.524	0	0	0.524

HCK	TLR2	9606.ENSP00000444986	9606.ENSP00000260010	0.576	0.089	0	0.597
HCK	PDGFRB	9606.ENSP00000444986	9606.ENSP00000261799	0	0.122	0.9	0.908
HCK	PIK3CA	9606.ENSP00000444986	9606.ENSP00000263967	0.062	0.104	0.6	0.634
HCK	STAT3	9606.ENSP00000444986	9606.ENSP00000264657	0	0.517	0.9	0.949
HCK	PIK3CB	9606.ENSP00000444986	9606.ENSP00000289153	0.062	0.87	0.6	0.947
HCK	S1PR1	9606.ENSP00000444986	9606.ENSP00000305416	0.078	0.069	0.9	0.906
HCK	TLR8	9606.ENSP00000444986	9606.ENSP00000312082	0.539	0.089	0	0.562
HCK	LCK	9606.ENSP00000444986	9606.ENSP00000337825	0.065	0	0.9	0.902
HCK	PTPN11	9606.ENSP00000444986	9606.ENSP00000340944	0.062	0.078	0.9	0.905
HCK	PTK2	9606.ENSP00000444986	9606.ENSP00000341189	0.055	0.307	0.9	0.928
HCK	JAK1	9606.ENSP00000444986	9606.ENSP00000343204	0	0	0.9	0.9
HCK	PIK3CG	9606.ENSP00000444986	9606.ENSP00000352121	0.159	0.104	0.6	0.672
HCK	MPEG1	9606.ENSP00000444986	9606.ENSP00000354335	0.456	0	0	0.456
HCK	SRC	9606.ENSP00000444986	9606.ENSP00000362680	0	0.27	0.9	0.923
HCK	SYK	9606.ENSP00000444986	9606.ENSP00000364907	0.302	0	0.9	0.927
HCK	PIK3CD	9606.ENSP00000444986	9606.ENSP00000366563	0.121	0.104	0.6	0.657
HCK	IL6ST	9606.ENSP00000444986	9606.ENSP00000370698	0	0.281	0.9	0.925
HCK	TNK2	9606.ENSP00000444986	9606.ENSP00000371341	0	0.498	0	0.498
HCK	PRKCD	9606.ENSP00000444986	9606.ENSP00000378217	0.109	0.117	0.9	0.914
HCK	PTK2B	9606.ENSP00000444986	9606.ENSP00000380638	0.117	0.078	0.9	0.911
HCK	ITGB2	9606.ENSP00000444986	9606.ENSP00000380948	0.708	0.147	0.9	0.972
HCK	ITK	9606.ENSP00000444986	9606.ENSP00000398655	0.081	0	0.9	0.904
HCK	PTPRC	9606.ENSP00000444986	9606.ENSP00000411355	0.384	0.078	0.8	0.876
HCK	PIK3R1	9606.ENSP00000444986	9606.ENSP00000428056	0	0.316	0.6	0.714
HCK	LYN	9606.ENSP00000444986	9606.ENSP00000428924	0.287	0.131	0.9	0.932
HCK	YES1	9606.ENSP00000444986	9606.ENSP00000462468	0	0	0.9	0.9
HCRTR1	HCRTR2	9606.ENSP00000384387	9606.ENSP00000477548	0.062	0	0.8	0.804
HDAC1	HDAC7	9606.ENSP00000362649	9606.ENSP00000080059	0.08	0.518	0.5	0.759
HDAC1	HDAC5	9606.ENSP00000362649	9606.ENSP00000225983	0.062	0.518	0.5	0.754
HDAC1	NFKB1	9606.ENSP00000362649	9606.ENSP00000226574	0.074	0.894	0	0.897
HDAC1	NR3C1	9606.ENSP00000362649	9606.ENSP00000231509	0	0.859	0	0.859
HDAC1	RARA	9606.ENSP00000362649	9606.ENSP00000254066	0	0.298	0.9	0.926
HDAC1	VHL	9606.ENSP00000362649	9606.ENSP00000256474	0	0.486	0	0.485
HDAC1	MDM2	9606.ENSP00000362649	9606.ENSP00000258149	0	0.675	0	0.675
HDAC1	KAT2B	9606.ENSP00000362649	9606.ENSP00000263754	0.095	0.289	0.65	0.755
HDAC1	THRA	9606.ENSP00000362649	9606.ENSP00000264637	0	0.078	0.6	0.615
HDAC1	STAT3	9606.ENSP00000362649	9606.ENSP00000264657	0.048	0.845	0	0.846
HDAC1	SMARCA2	9606.ENSP00000362649	9606.ENSP00000265773	0.125	0.718	0	0.743
HDAC1	NCOR1	9606.ENSP00000362649	9606.ENSP00000268712	0.106	0.535	0.9	0.954
HDAC1	PPARG	9606.ENSP00000362649	9606.ENSP00000287820	0	0.505	0.65	0.819
HDAC1	HDAC3	9606.ENSP00000362649	9606.ENSP00000302967	0.117	0.322	0.9	0.935
HDAC1	PTGDR	9606.ENSP00000362649	9606.ENSP00000303424	0	0	0.9	0.9
HDAC1	TERT	9606.ENSP00000362649	9606.ENSP00000309572	0.066	0	0.9	0.902
HDAC1	PPARD	9606.ENSP00000362649	9606.ENSP00000310928	0	0.505	0	0.505

HDAC1	RARB	9606.ENSP00000362649	9606.ENSP00000332296	0	0.078	0.9	0.903
HDAC1	PPP1CC	9606.ENSP00000362649	9606.ENSP00000341779	0.054	0.7	0	0.704
HDAC1	RXRG	9606.ENSP00000362649	9606.ENSP00000352900	0	0.078	0.9	0.903
HDAC1	PARP1	9606.ENSP00000362649	9606.ENSP00000355759	0.125	0.682	0	0.709
HDAC1	JUN	9606.ENSP00000362649	9606.ENSP00000360266	0	0.476	0.65	0.808
HDAC1	PCNA	9606.ENSP00000362649	9606.ENSP00000368458	0.186	0.486	0	0.564
HDAC1	THRB	9606.ENSP00000362649	9606.ENSP00000379904	0	0.078	0.6	0.615
HDAC1	TOP2A	9606.ENSP00000362649	9606.ENSP00000411532	0.154	0.486	0	0.546
HDAC1	RARG	9606.ENSP00000362649	9606.ENSP00000388510	0.062	0.078	0.9	0.905
HDAC1	RXRB	9606.ENSP00000362649	9606.ENSP00000363817	0.053	0.078	0.9	0.905
HDAC1	OPRM1	9606.ENSP00000362649	9606.ENSP00000394624	0	0	0.9	0.9
HDAC1	RXRA	9606.ENSP00000362649	9606.ENSP00000419692	0.145	0.078	0.9	0.914
HDAC1	HDAC9	9606.ENSP00000362649	9606.ENSP00000408617	0.062	0.911	0	0.912
HDAC1	XPO1	9606.ENSP00000362649	9606.ENSP00000384863	0.177	0.182	0.9	0.926
HDAC1	HIF1A	9606.ENSP00000362649	9606.ENSP00000437955	0.05	0.759	0	0.761
HDAC1	RELA	9606.ENSP00000362649	9606.ENSP00000384273	0.107	0.894	0	0.901
HDAC1	KDM1A	9606.ENSP00000362649	9606.ENSP00000383042	0.123	0.969	0.5	0.985
HDAC1	SMARCA4	9606.ENSP00000362649	9606.ENSP00000395654	0.305	0.879	0.65	0.968
HDAC1	NCOR2	9606.ENSP00000362649	9606.ENSP00000384018	0.064	0.793	0.9	0.978
HDAC1	HDAC2	9606.ENSP00000362649	9606.ENSP00000430432	0.111	0.992	0.9	0.999
HDAC10	HDAC3	9606.ENSP00000216271	9606.ENSP00000302967	0.062	0.518	0	0.529
HDAC10	HDAC2	9606.ENSP00000216271	9606.ENSP00000430432	0.062	0.518	0	0.529
HDAC10	NCOR1	9606.ENSP00000216271	9606.ENSP00000268712	0.065	0.251	0.6	0.695
HDAC10	KAT2B	9606.ENSP00000216271	9606.ENSP00000263754	0.062	0.257	0.65	0.734
HDAC10	NCOR2	9606.ENSP00000216271	9606.ENSP00000384018	0.11	0.598	0.6	0.844
HDAC11	KAT2B	9606.ENSP00000295757	9606.ENSP00000263754	0.062	0.171	0.65	0.704
HDAC11	NCOR1	9606.ENSP00000295757	9606.ENSP00000268712	0.064	0.251	0.6	0.695
HDAC11	KDM1A	9606.ENSP00000295757	9606.ENSP00000383042	0.056	0.428	0	0.437
HDAC11	NCOR2	9606.ENSP00000295757	9606.ENSP00000384018	0.082	0.251	0.6	0.701
HDAC11	HDAC6	9606.ENSP00000295757	9606.ENSP00000334061	0	0.345	0.9	0.931
HDAC2	HDAC7	9606.ENSP00000430432	9606.ENSP00000080059	0.062	0.541	0.5	0.766
HDAC2	HDAC5	9606.ENSP00000430432	9606.ENSP00000225983	0.062	0.368	0.9	0.935
HDAC2	NFKB1	9606.ENSP00000430432	9606.ENSP00000226574	0.066	0.4	0.9	0.939
HDAC2	NR3C1	9606.ENSP00000430432	9606.ENSP00000231509	0	0.298	0.9	0.926
HDAC2	RARA	9606.ENSP00000430432	9606.ENSP00000254066	0	0.505	0	0.505
HDAC2	VHL	9606.ENSP00000430432	9606.ENSP00000256474	0	0.626	0	0.626
HDAC2	KAT2B	9606.ENSP00000430432	9606.ENSP00000263754	0.095	0.289	0.65	0.755
HDAC2	THRA	9606.ENSP00000430432	9606.ENSP00000264637	0	0.243	0.6	0.684
HDAC2	STAT3	9606.ENSP00000430432	9606.ENSP00000264657	0.048	0.655	0	0.657
HDAC2	SMARCA2	9606.ENSP00000430432	9606.ENSP00000265773	0.125	0.716	0	0.741
HDAC2	NCOR1	9606.ENSP00000430432	9606.ENSP00000268712	0.094	0.39	0.9	0.939
HDAC2	PPARG	9606.ENSP00000430432	9606.ENSP00000287820	0	0.078	0.65	0.663
HDAC2	HDAC3	9606.ENSP00000430432	9606.ENSP00000302967	0.072	0.175	0.8	0.833
HDAC2	PPARD	9606.ENSP00000430432	9606.ENSP00000310928	0	0.646	0	0.646

HDAC2	RXRG	9606.ENSP00000430432	9606.ENSP00000352900	0	0.078	0.6	0.615
HDAC2	PARP1	9606.ENSP00000430432	9606.ENSP00000355759	0.09	0.501	0	0.527
HDAC2	JUN	9606.ENSP00000430432	9606.ENSP00000360266	0	0.273	0.65	0.734
HDAC2	RXRB	9606.ENSP00000430432	9606.ENSP00000363817	0.062	0.078	0.6	0.623
HDAC2	THRB	9606.ENSP00000430432	9606.ENSP00000379904	0	0.243	0.6	0.684
HDAC2	KDM1A	9606.ENSP00000430432	9606.ENSP00000383042	0.085	0.963	0.5	0.981
HDAC2	NCOR2	9606.ENSP00000430432	9606.ENSP00000384018	0.064	0.783	0.9	0.977
HDAC2	RELA	9606.ENSP00000430432	9606.ENSP00000384273	0.045	0.883	0.9	0.987
HDAC2	OPRM1	9606.ENSP00000430432	9606.ENSP00000394624	0	0	0.9	0.9
HDAC2	SMARCA4	9606.ENSP00000430432	9606.ENSP00000395654	0.176	0.887	0.8	0.979
HDAC2	RXRA	9606.ENSP00000430432	9606.ENSP00000419692	0.071	0.078	0.6	0.627
HDAC2	HIF1A	9606.ENSP00000430432	9606.ENSP00000437955	0.062	0.641	0	0.649
HDAC3	HDAC7	9606.ENSP00000302967	9606.ENSP00000080059	0.062	0.661	0.9	0.965
HDAC3	TNFRSF1A	9606.ENSP00000302967	9606.ENSP00000162749	0	0	0.9	0.9
HDAC3	HDAC5	9606.ENSP00000302967	9606.ENSP00000225983	0.062	0.917	0.3	0.941
HDAC3	NFKB1	9606.ENSP00000302967	9606.ENSP00000226574	0.062	0.565	0.9	0.955
HDAC3	NR1D1	9606.ENSP00000302967	9606.ENSP00000246672	0	0.27	0.6	0.695
HDAC3	NR1H2	9606.ENSP00000302967	9606.ENSP00000253727	0	0.078	0.6	0.615
HDAC3	RARA	9606.ENSP00000302967	9606.ENSP00000254066	0	0.298	0.9	0.926
HDAC3	RORA	9606.ENSP00000302967	9606.ENSP00000261523	0	0	0.65	0.65
HDAC3	KAT2B	9606.ENSP00000302967	9606.ENSP00000263754	0.095	0.458	0.65	0.813
HDAC3	HDAC4	9606.ENSP00000302967	9606.ENSP00000264606	0.062	0.916	0.9	0.991
HDAC3	THRA	9606.ENSP00000302967	9606.ENSP00000264637	0	0.327	0.6	0.719
HDAC3	STAT3	9606.ENSP00000302967	9606.ENSP00000264657	0.048	0.845	0.9	0.984
HDAC3	NCOR1	9606.ENSP00000302967	9606.ENSP00000268712	0.103	0.968	0.8	0.993
HDAC3	PPARG	9606.ENSP00000302967	9606.ENSP00000287820	0	0.841	0.9	0.983
HDAC3	PPARD	9606.ENSP00000302967	9606.ENSP00000310928	0	0.505	0	0.505
HDAC3	HIF1A	9606.ENSP00000302967	9606.ENSP00000437955	0	0.499	0	0.499
HDAC3	NR1H3	9606.ENSP00000302967	9606.ENSP00000477707	0	0.078	0.6	0.615
HDAC3	THRB	9606.ENSP00000302967	9606.ENSP00000379904	0	0.128	0.6	0.636
HDAC3	XPO1	9606.ENSP00000302967	9606.ENSP00000384863	0.09	0.576	0	0.598
HDAC3	SRC	9606.ENSP00000302967	9606.ENSP00000362680	0.063	0.505	0	0.517
HDAC3	PPARA	9606.ENSP00000302967	9606.ENSP00000385523	0	0.078	0.6	0.615
HDAC3	SMARCA4	9606.ENSP00000302967	9606.ENSP00000395654	0.107	0.577	0	0.606
HDAC3	HDAC9	9606.ENSP00000302967	9606.ENSP00000408617	0.062	0.787	0	0.792
HDAC3	RARG	9606.ENSP00000302967	9606.ENSP00000388510	0	0.078	0.9	0.903
HDAC3	RXRB	9606.ENSP00000302967	9606.ENSP00000363817	0.062	0.078	0.9	0.905
HDAC3	RARB	9606.ENSP00000302967	9606.ENSP00000332296	0	0.078	0.9	0.903
HDAC3	TNF	9606.ENSP00000302967	9606.ENSP00000398698	0	0.067	0.9	0.902
HDAC3	RXRG	9606.ENSP00000302967	9606.ENSP00000352900	0	0.078	0.9	0.903
HDAC3	RXRA	9606.ENSP00000302967	9606.ENSP00000419692	0.047	0.298	0.9	0.927
HDAC3	JUN	9606.ENSP00000302967	9606.ENSP00000360266	0	0.376	0.65	0.772
HDAC3	KDM1A	9606.ENSP00000302967	9606.ENSP00000383042	0.062	0.807	0.5	0.901
HDAC3	RELA	9606.ENSP00000302967	9606.ENSP00000384273	0.042	0.874	0.9	0.986

HDAC3	NCOR2	9606.ENSP00000302967	9606.ENSP00000384018	0.064	0.987	0.9	0.998
HDAC4	HDAC5	9606.ENSP00000264606	9606.ENSP00000225983	0.062	0.486	0.8	0.895
HDAC4	NR1H2	9606.ENSP00000264606	9606.ENSP00000253727	0.052	0.298	0.9	0.927
HDAC4	KAT2B	9606.ENSP00000264606	9606.ENSP00000263754	0.062	0.257	0.65	0.734
HDAC4	PPARG	9606.ENSP00000264606	9606.ENSP00000287820	0	0.505	0	0.505
HDAC4	XPO1	9606.ENSP00000264606	9606.ENSP00000384863	0.067	0.056	0.9	0.904
HDAC4	NR1H3	9606.ENSP00000264606	9606.ENSP00000477707	0.052	0.298	0.9	0.927
HDAC4	YWHAG	9606.ENSP00000264606	9606.ENSP00000306330	0.059	0.953	0	0.953
HDAC4	HIF1A	9606.ENSP00000264606	9606.ENSP00000437955	0	0.767	0	0.767
HDAC4	NCOR1	9606.ENSP00000264606	9606.ENSP00000268712	0.065	0.904	0.6	0.961
HDAC4	NCOR2	9606.ENSP00000264606	9606.ENSP00000384018	0.065	0.949	0.9	0.994
HDAC5	HDAC7	9606.ENSP00000225983	9606.ENSP00000080059	0.063	0.27	0.3	0.479
HDAC5	VCP	9606.ENSP00000225983	9606.ENSP00000351777	0.062	0.426	0	0.439
HDAC5	PPARG	9606.ENSP00000225983	9606.ENSP00000287820	0	0.078	0.65	0.663
HDAC5	JUN	9606.ENSP00000225983	9606.ENSP00000360266	0.049	0.117	0.65	0.68
HDAC5	YWHAG	9606.ENSP00000225983	9606.ENSP00000306330	0.059	0.772	0	0.776
HDAC5	KAT2B	9606.ENSP00000225983	9606.ENSP00000263754	0.062	0.257	0.65	0.734
HDAC5	KDM1A	9606.ENSP00000225983	9606.ENSP00000383042	0.062	0.428	0.5	0.708
HDAC5	NCOR2	9606.ENSP00000225983	9606.ENSP00000384018	0.088	0.748	0.9	0.975
HDAC5	NCOR1	9606.ENSP00000225983	9606.ENSP00000268712	0.065	0.807	0.6	0.921
HDAC6	MAPK1	9606.ENSP00000334061	9606.ENSP00000215832	0	0.495	0	0.495
HDAC6	NR3C1	9606.ENSP00000334061	9606.ENSP00000231509	0	0.298	0.9	0.926
HDAC6	SIRT2	9606.ENSP00000334061	9606.ENSP00000249396	0.052	0.562	0	0.567
HDAC6	MAPK3	9606.ENSP00000334061	9606.ENSP00000263025	0	0.495	0	0.495
HDAC6	KAT2B	9606.ENSP00000334061	9606.ENSP00000263754	0.062	0.257	0.65	0.734
HDAC6	NCOR1	9606.ENSP00000334061	9606.ENSP00000268712	0.065	0.251	0.6	0.695
HDAC6	LRRK2	9606.ENSP00000334061	9606.ENSP00000298910	0.065	0.519	0	0.531
HDAC6	PKM	9606.ENSP00000334061	9606.ENSP00000320171	0.052	0.675	0	0.678
HDAC6	PBRM1	9606.ENSP00000334061	9606.ENSP00000378307	0.062	0.406	0	0.419
HDAC6	PRKCA	9606.ENSP00000334061	9606.ENSP00000408695	0	0.486	0	0.485
HDAC6	KDM1A	9606.ENSP00000334061	9606.ENSP00000383042	0.062	0.428	0	0.44
HDAC6	NCOR2	9606.ENSP00000334061	9606.ENSP00000384018	0.065	0.251	0.6	0.695
HDAC6	MAPT	9606.ENSP00000334061	9606.ENSP00000340820	0	0.684	0	0.684
HDAC6	VCP	9606.ENSP00000334061	9606.ENSP00000351777	0.062	0.639	0.8	0.926
HDAC6	HSP90AA1	9606.ENSP00000334061	9606.ENSP00000335153	0.053	0.885	0.9	0.988
HDAC7	YWHAG	9606.ENSP00000080059	9606.ENSP00000306330	0.059	0.543	0	0.551
HDAC7	PPARG	9606.ENSP00000080059	9606.ENSP00000287820	0	0.078	0.65	0.663
HDAC7	KDM1A	9606.ENSP00000080059	9606.ENSP00000383042	0.062	0.249	0.5	0.617
HDAC7	JUN	9606.ENSP00000080059	9606.ENSP00000360266	0	0.117	0.65	0.677
HDAC7	KAT2B	9606.ENSP00000080059	9606.ENSP00000263754	0.062	0.257	0.65	0.734
HDAC7	NCOR1	9606.ENSP00000080059	9606.ENSP00000268712	0.072	0.598	0.6	0.838
HDAC7	HIF1A	9606.ENSP00000080059	9606.ENSP00000437955	0	0.501	0.9	0.948
HDAC7	NCOR2	9606.ENSP00000080059	9606.ENSP00000384018	0.099	0.894	0.6	0.958
HDAC8	KAT2B	9606.ENSP00000362674	9606.ENSP00000263754	0.095	0.213	0.65	0.728

HDAC8	NCOR1	9606.ENSP00000362674	9606.ENSP00000268712	0.064	0.251	0.6	0.695
HDAC8	NCOR2	9606.ENSP00000362674	9606.ENSP00000384018	0.064	0.251	0.6	0.695
HDAC9	KAT2B	9606.ENSP00000408617	9606.ENSP00000263754	0.062	0.257	0.65	0.734
HDAC9	NCOR1	9606.ENSP00000408617	9606.ENSP00000268712	0.065	0.579	0.6	0.829
HDAC9	NCOR2	9606.ENSP00000408617	9606.ENSP00000384018	0.065	0.251	0.6	0.695
HIF1A	MAPK1	9606.ENSP00000437955	9606.ENSP00000215832	0	0.675	0.8	0.932
HIF1A	NR3C1	9606.ENSP00000437955	9606.ENSP00000231509	0	0.108	0.65	0.674
HIF1A	VHL	9606.ENSP00000437955	9606.ENSP00000256474	0	0.998	0.9	0.999
HIF1A	MDM2	9606.ENSP00000437955	9606.ENSP00000258149	0.062	0.873	0.9	0.987
HIF1A	RORA	9606.ENSP00000437955	9606.ENSP00000261523	0	0.283	0.9	0.925
HIF1A	MAPK3	9606.ENSP00000437955	9606.ENSP00000263025	0	0.213	0.8	0.835
HIF1A	KAT2B	9606.ENSP00000437955	9606.ENSP00000263754	0.062	0.841	0	0.844
HIF1A	STAT3	9606.ENSP00000437955	9606.ENSP00000264657	0.062	0.839	0.9	0.983
HIF1A	NOS3	9606.ENSP00000437955	9606.ENSP00000297494	0	0	0.9	0.9
HIF1A	LRRK2	9606.ENSP00000437955	9606.ENSP00000298910	0	0.298	0.9	0.926
HIF1A	HNF4A	9606.ENSP00000437955	9606.ENSP00000312987	0.062	0.514	0	0.525
HIF1A	RORC	9606.ENSP00000437955	9606.ENSP00000327025	0	0.182	0.9	0.914
HIF1A	HSP90AA1	9606.ENSP00000437955	9606.ENSP00000335153	0	0.918	0.9	0.991
HIF1A	JUN	9606.ENSP00000437955	9606.ENSP00000360266	0	0.676	0.9	0.966
HIF1A	PIM1	9606.ENSP00000437955	9606.ENSP00000362608	0	0	0.9	0.9
HIF1A	SRC	9606.ENSP00000437955	9606.ENSP00000362680	0.062	0.685	0	0.692
HIF1A	LDHA	9606.ENSP00000437955	9606.ENSP00000445175	0.056	0.513	0.9	0.95
HMGCR	SQLE	9606.ENSP00000287936	9606.ENSP00000265896	0.912	0	0	0.912
HMGCR	VCP	9606.ENSP00000287936	9606.ENSP00000351777	0.062	0.519	0	0.53
HMGCR	RXRA	9606.ENSP00000287936	9606.ENSP00000419692	0.062	0	0.9	0.902
HMGCR	PPARA	9606.ENSP00000287936	9606.ENSP00000385523	0.062	0	0.9	0.902
HMGCR	SREBF2	9606.ENSP00000287936	9606.ENSP00000354476	0.085	0	0.9	0.904
HMOX1	MAPK14	9606.ENSP00000216117	9606.ENSP00000229795	0	0	0.9	0.9
HMOX1	JUN	9606.ENSP00000216117	9606.ENSP00000360266	0	0	0.9	0.9
HMOX1	NFE2L2	9606.ENSP00000216117	9606.ENSP00000380252	0	0	0.9	0.9
HNF4A	NR1H4	9606.ENSP00000312987	9606.ENSP00000447149	0.496	0.149	0	0.553
HNF4A	NR1I2	9606.ENSP00000312987	9606.ENSP00000336528	0.127	0.543	0	0.584
HNF4A	SREBF2	9606.ENSP00000312987	9606.ENSP00000354476	0	0.495	0	0.495
HPGDS	PTGS1	9606.ENSP00000295256	9606.ENSP00000354612	0.069	0	0.9	0.902
HPGDS	PTGES	9606.ENSP00000295256	9606.ENSP00000342385	0	0	0.9	0.9
HPGDS	PTGS2	9606.ENSP00000295256	9606.ENSP00000356438	0	0	0.9	0.9
HRH3	HTR5A	9606.ENSP00000342560	9606.ENSP00000287907	0.472	0	0	0.472
HSD11B1	PPARG	9606.ENSP00000355995	9606.ENSP00000287820	0.062	0.057	0.9	0.903
HSD11B1	UGT2B7	9606.ENSP00000355995	9606.ENSP00000304811	0.108	0.059	0.65	0.68
HSD11B1	HSD11B2	9606.ENSP00000355995	9606.ENSP00000316786	0	0	0.8	0.8
HSD11B1	RXRA	9606.ENSP00000355995	9606.ENSP00000419692	0.062	0.057	0.9	0.903
HSD17B1	HSD17B2	9606.ENSP00000466799	9606.ENSP00000199936	0	0	0.8	0.8
HSD17B1	UGT2B7	9606.ENSP00000466799	9606.ENSP00000304811	0.062	0.059	0.65	0.664
HSD17B2	UGT2B7	9606.ENSP00000199936	9606.ENSP00000304811	0.082	0	0.65	0.664

HSD17B2	SRD5A2	9606.ENSP00000199936	9606.ENSP00000477587	0.063	0	0.9	0.902
HSD17B2	SRD5A1	9606.ENSP00000199936	9606.ENSP00000274192	0.062	0	0.9	0.902
HSD17B2	HSD17B3	9606.ENSP00000199936	9606.ENSP00000364412	0	0	0.8	0.8
HSD17B3	SRD5A1	9606.ENSP00000364412	9606.ENSP00000274192	0.049	0	0.9	0.9
HSD17B3	UGT2B7	9606.ENSP00000364412	9606.ENSP00000304811	0	0	0.65	0.65
HSD17B3	SRD5A2	9606.ENSP00000364412	9606.ENSP00000477587	0.049	0	0.9	0.9
HSP90AA1	MAPK1	9606.ENSP00000335153	9606.ENSP00000215832	0.062	0.365	0.9	0.935
HSP90AA1	RIPK2	9606.ENSP00000335153	9606.ENSP00000220751	0	0.228	0.8	0.839
HSP90AA1	RPS6KB1	9606.ENSP00000335153	9606.ENSP00000225577	0.076	0.247	0.9	0.924
HSP90AA1	NR3C1	9606.ENSP00000335153	9606.ENSP00000231509	0	0.923	0.9	0.992
HSP90AA1	RAF1	9606.ENSP00000335153	9606.ENSP00000251849	0	0.942	0	0.942
HSP90AA1	VHL	9606.ENSP00000335153	9606.ENSP00000256474	0	0.439	0	0.439
HSP90AA1	MDM2	9606.ENSP00000335153	9606.ENSP00000258149	0	0.675	0	0.675
HSP90AA1	KCNH2	9606.ENSP00000335153	9606.ENSP00000262186	0	0.835	0	0.835
HSP90AA1	MAPK3	9606.ENSP00000335153	9606.ENSP00000263025	0.054	0.299	0.9	0.927
HSP90AA1	KDR	9606.ENSP00000335153	9606.ENSP00000263923	0	0.488	0.9	0.946
HSP90AA1	PIK3CA	9606.ENSP00000335153	9606.ENSP00000263967	0	0.092	0.6	0.621
HSP90AA1	STAT3	9606.ENSP00000335153	9606.ENSP00000264657	0	0.27	0.9	0.923
HSP90AA1	NOS3	9606.ENSP00000335153	9606.ENSP00000297494	0.062	0.76	0.9	0.975
HSP90AA1	LRRK2	9606.ENSP00000335153	9606.ENSP00000298910	0	0.919	0	0.919
HSP90AA1	PRKCE	9606.ENSP00000335153	9606.ENSP00000306124	0	0.486	0	0.486
HSP90AA1	YWHAG	9606.ENSP00000335153	9606.ENSP00000306330	0.118	0.358	0	0.409
HSP90AA1	TERT	9606.ENSP00000335153	9606.ENSP00000309572	0	0.883	0.9	0.987
HSP90AA1	MAP3K11	9606.ENSP00000335153	9606.ENSP00000309597	0	0.55	0	0.55
HSP90AA1	MET	9606.ENSP00000335153	9606.ENSP00000317272	0	0.55	0	0.55
HSP90AA1	ROCK2	9606.ENSP00000335153	9606.ENSP00000317985	0	0.161	0.9	0.912
HSP90AA1	PGR	9606.ENSP00000335153	9606.ENSP00000325120	0	0.344	0.6	0.726
HSP90AA1	NOS2	9606.ENSP00000335153	9606.ENSP00000327251	0.062	0.299	0.9	0.928
HSP90AA1	PIK3R1	9606.ENSP00000335153	9606.ENSP00000428056	0	0	0.6	0.6
HSP90AA1	PIM1	9606.ENSP00000335153	9606.ENSP00000362608	0	0.494	0	0.494
HSP90AA1	NTRK1	9606.ENSP00000335153	9606.ENSP00000431418	0	0.493	0	0.493
HSP90AA1	NR1I3	9606.ENSP00000335153	9606.ENSP00000356959	0	0.64	0	0.64
HSP90AA1	LIMK1	9606.ENSP00000335153	9606.ENSP00000336740	0.051	0.237	0.6	0.685
HSP90AA1	TNK2	9606.ENSP00000335153	9606.ENSP00000371341	0	0.772	0	0.772
HSP90AA1	PRKCD	9606.ENSP00000335153	9606.ENSP00000378217	0	0.476	0	0.476
HSP90AA1	LCK	9606.ENSP00000335153	9606.ENSP00000337825	0.048	0.714	0	0.716
HSP90AA1	RAC1	9606.ENSP00000335153	9606.ENSP00000348461	0	0.056	0.9	0.901
HSP90AA1	WEE1	9606.ENSP00000335153	9606.ENSP00000402084	0	0.5	0	0.5
HSP90AA1	NR3C2	9606.ENSP00000335153	9606.ENSP00000350815	0	0.335	0.6	0.722
HSP90AA1	MAPT	9606.ENSP00000335153	9606.ENSP00000340820	0	0.759	0	0.759
HSP90AA1	PTK2B	9606.ENSP00000335153	9606.ENSP00000380638	0	0.129	0.9	0.909
HSP90AA1	PRKCA	9606.ENSP00000335153	9606.ENSP00000408695	0	0.288	0.9	0.925
HSP90AA1	VCP	9606.ENSP00000335153	9606.ENSP00000351777	0.157	0.453	0.6	0.799
HSP90AA1	MCL1	9606.ENSP00000335153	9606.ENSP00000358022	0	0.128	0.9	0.909

HSP90AA1	ROCK1	9606.ENSP00000335153	9606.ENSP00000382697	0.062	0.161	0.9	0.914
HSP90AA1	PTK2	9606.ENSP00000335153	9606.ENSP00000341189	0	0.129	0.9	0.909
HSP90AA1	HTT	9606.ENSP00000335153	9606.ENSP00000347184	0	0.426	0	0.426
HSP90AA1	MTOR	9606.ENSP00000335153	9606.ENSP00000354558	0	0.092	0.9	0.905
HSP90AA1	LYN	9606.ENSP00000335153	9606.ENSP00000428924	0	0.539	0	0.539
HSP90AA1	PDPK1	9606.ENSP00000335153	9606.ENSP00000344220	0.086	0.213	0.9	0.921
HSP90AA1	TGFBR1	9606.ENSP00000335153	9606.ENSP00000364133	0	0.715	0	0.715
HSP90AA1	PPARA	9606.ENSP00000335153	9606.ENSP00000385523	0	0.51	0.9	0.949
HSP90AA1	IKBKG	9606.ENSP00000335153	9606.ENSP00000483825	0	0.681	0	0.681
HSP90AA1	IKBKB	9606.ENSP00000335153	9606.ENSP00000430684	0	0.723	0	0.723
HSP90AA1	NLRP3	9606.ENSP00000335153	9606.ENSP00000337383	0	0.27	0.8	0.847
HSP90AA1	HSPA1A	9606.ENSP00000335153	9606.ENSP00000364802	0.317	0.871	0.9	0.99
HSP90AA1	SRC	9606.ENSP00000335153	9606.ENSP00000362680	0	0.821	0.9	0.981
HSPA1A	MAPK1	9606.ENSP00000364802	9606.ENSP00000215832	0	0.104	0.9	0.906
HSPA1A	NR3C1	9606.ENSP00000364802	9606.ENSP00000231509	0	0.288	0.65	0.74
HSPA1A	MAPK3	9606.ENSP00000364802	9606.ENSP00000263025	0	0.187	0.9	0.915
HSPA1A	PABPC1	9606.ENSP00000364802	9606.ENSP00000313007	0.11	0.081	0.8	0.822
HSPA1A	PGR	9606.ENSP00000364802	9606.ENSP00000325120	0	0.288	0.65	0.74
HSPA1A	MAPT	9606.ENSP00000364802	9606.ENSP00000340820	0	0.521	0	0.521
HSPA1A	NR3C2	9606.ENSP00000364802	9606.ENSP00000350815	0	0.065	0.65	0.658
HSPA1A	VCP	9606.ENSP00000364802	9606.ENSP00000351777	0.101	0.416	0	0.452
HSPA1A	PRKCA	9606.ENSP00000364802	9606.ENSP00000408695	0	0.435	0	0.435
HSPA1A	IKBKG	9606.ENSP00000364802	9606.ENSP00000483825	0.065	0.455	0	0.468
HSPA1A	JAK2	9606.ENSP00000364802	9606.ENSP00000371067	0	0.146	0.9	0.91
HSPA1A	PPARA	9606.ENSP00000364802	9606.ENSP00000385523	0	0.065	0.9	0.902
HTR1A	HTR2A	9606.ENSP00000316244	9606.ENSP00000437737	0.108	0.357	0	0.402
HTR1A	HTR1B	9606.ENSP00000316244	9606.ENSP00000358963	0.097	0.491	0	0.52
HTR1B	HTR1D	9606.ENSP00000358963	9606.ENSP00000363748	0	0.27	0.3	0.467
HTR1E	HTR5A	9606.ENSP00000307766	9606.ENSP00000287907	0.395	0	0.3	0.558
HTR2A	HTR2B	9606.ENSP00000437737	9606.ENSP00000258400	0	0.312	0.8	0.856
HTR2A	HTR2C	9606.ENSP00000437737	9606.ENSP00000276198	0.085	0.621	0.8	0.924
HTR2B	HTR2C	9606.ENSP00000258400	9606.ENSP00000276198	0	0.312	0.8	0.856
HTR2C	PTGER1	9606.ENSP00000276198	9606.ENSP00000292513	0	0	0.9	0.9
HTR3A	PTK2B	9606.ENSP00000347754	9606.ENSP00000380638	0.062	0	0.54	0.55
HTR5A	MCHR1	9606.ENSP00000287907	9606.ENSP00000249016	0.654	0	0	0.654
HTR6	HTR7	9606.ENSP00000289753	9606.ENSP00000337949	0.062	0	0.8	0.804
HTT	NCOR1	9606.ENSP00000347184	9606.ENSP00000268712	0	0.469	0	0.469
HTT	PSMB5	9606.ENSP00000347184	9606.ENSP00000355325	0	0	0.6	0.6
HTT	PIK3R1	9606.ENSP00000347184	9606.ENSP00000428056	0	0.468	0	0.468
HTT	IKBKG	9606.ENSP00000347184	9606.ENSP00000483825	0.062	0.494	0	0.505
HTT	MAPK8	9606.ENSP00000347184	9606.ENSP00000378974	0	0	0.8	0.8
HTT	MAPK10	9606.ENSP00000347184	9606.ENSP00000352157	0.062	0	0.8	0.804
HTT	VCP	9606.ENSP00000347184	9606.ENSP00000351777	0.062	0.888	0	0.891
ICAM1	STAT3	9606.ENSP00000264832	9606.ENSP00000264657	0.085	0	0.9	0.904

ICAM1	RELA	9606.ENSP00000264832	9606.ENSP00000384273	0.065	0.681	0	0.689
ICAM1	S1PR1	9606.ENSP00000264832	9606.ENSP00000305416	0.062	0	0.9	0.902
ICAM1	ITGAL	9606.ENSP00000264832	9606.ENSP00000349252	0.091	0.972	0.9	0.997
ICAM1	ITGB2	9606.ENSP00000264832	9606.ENSP00000380948	0.113	0.835	0.9	0.984
IDH1	SLC27A2	9606.ENSP00000390265	9606.ENSP00000267842	0	0	0.9	0.9
IDH1	PAOX	9606.ENSP00000390265	9606.ENSP00000278060	0	0	0.9	0.9
IDH1	NOS2	9606.ENSP00000390265	9606.ENSP00000327251	0	0	0.9	0.9
IDO1	MAOA	9606.ENSP00000430950	9606.ENSP00000340684	0	0	0.9	0.9
IDO1	MAOB	9606.ENSP00000430950	9606.ENSP00000367309	0	0	0.9	0.9
IDO1	TDO2	9606.ENSP00000430950	9606.ENSP00000444788	0	0	0.8	0.8
IGF1R	MAPK1	9606.ENSP00000268035	9606.ENSP00000215832	0	0.148	0.9	0.911
IGF1R	RPS6KB1	9606.ENSP00000268035	9606.ENSP00000225577	0.056	0.119	0.9	0.909
IGF1R	RAF1	9606.ENSP00000268035	9606.ENSP00000251849	0	0	0.9	0.9
IGF1R	MDM2	9606.ENSP00000268035	9606.ENSP00000258149	0	0.88	0.6	0.95
IGF1R	MAPK3	9606.ENSP00000268035	9606.ENSP00000263025	0	0.148	0.9	0.911
IGF1R	PIK3CA	9606.ENSP00000268035	9606.ENSP00000263967	0.063	0.146	0.9	0.912
IGF1R	STAT3	9606.ENSP00000268035	9606.ENSP00000264657	0	0.707	0	0.707
IGF1R	PCNA	9606.ENSP00000268035	9606.ENSP00000368458	0.062	0.4	0	0.413
IGF1R	S1PR3	9606.ENSP00000268035	9606.ENSP00000365006	0	0	0.65	0.65
IGF1R	JAK1	9606.ENSP00000268035	9606.ENSP00000343204	0	0.282	0.8	0.85
IGF1R	PTK2	9606.ENSP00000268035	9606.ENSP00000341189	0	0.298	0.8	0.853
IGF1R	PIK3CD	9606.ENSP00000268035	9606.ENSP00000366563	0.063	0.202	0.8	0.837
IGF1R	PIK3CB	9606.ENSP00000268035	9606.ENSP00000289153	0.063	0.146	0.8	0.825
IGF1R	JAK2	9606.ENSP00000268035	9606.ENSP00000371067	0	0.471	0.8	0.889
IGF1R	PRKCD	9606.ENSP00000268035	9606.ENSP00000378217	0.063	0.275	0.9	0.926
IGF1R	SRC	9606.ENSP00000268035	9606.ENSP00000362680	0	0.47	0.9	0.944
IGF1R	INSR	9606.ENSP00000268035	9606.ENSP00000303830	0.062	0.854	0.8	0.97
IGF1R	PIK3R1	9606.ENSP00000268035	9606.ENSP00000428056	0.049	0.805	0.9	0.979
IGF1R	PTPN1	9606.ENSP00000268035	9606.ENSP00000360683	0.062	0.957	0.9	0.995
IGF1R	PTPN11	9606.ENSP00000268035	9606.ENSP00000340944	0.062	0.712	0.9	0.97
IKBKB	TNFRSF1A	9606.ENSP00000430684	9606.ENSP00000162749	0.062	0.896	0.9	0.989
IKBKB	RIPK2	9606.ENSP00000430684	9606.ENSP00000220751	0	0	0.9	0.9
IKBKB	NFKB1	9606.ENSP00000430684	9606.ENSP00000226574	0.062	0.963	0.9	0.996
IKBKB	TLR2	9606.ENSP00000430684	9606.ENSP00000260010	0	0.089	0.9	0.905
IKBKB	PRKCQ	9606.ENSP00000430684	9606.ENSP00000263125	0.063	0.848	0.9	0.984
IKBKB	PRKCB	9606.ENSP00000430684	9606.ENSP00000305355	0	0.688	0.9	0.967
IKBKB	PRKCE	9606.ENSP00000430684	9606.ENSP00000306124	0.063	0.327	0.9	0.931
IKBKB	PDPK1	9606.ENSP00000430684	9606.ENSP00000344220	0.053	0.145	0.9	0.911
IKBKB	MTOR	9606.ENSP00000430684	9606.ENSP00000354558	0.062	0.519	0	0.53
IKBKB	TLR1	9606.ENSP00000430684	9606.ENSP00000354932	0	0.089	0.9	0.905
IKBKB	SRC	9606.ENSP00000430684	9606.ENSP00000362680	0.062	0.836	0	0.839
IKBKB	PRKCZ	9606.ENSP00000430684	9606.ENSP00000367830	0	0.213	0.9	0.917
IKBKB	RELA	9606.ENSP00000430684	9606.ENSP00000384273	0.063	0.881	0.9	0.987
IKBKB	TNF	9606.ENSP00000430684	9606.ENSP00000398698	0.062	0.696	0.9	0.969

IKBKB	PRKCA	9606.ENSP00000430684	9606.ENSP00000408695	0	0.285	0.9	0.925
IKBKB	NTRK1	9606.ENSP00000430684	9606.ENSP00000431418	0	0	0.9	0.9
IKBKB	IKBKG	9606.ENSP00000430684	9606.ENSP00000483825	0.062	0.998	0.9	0.999
IKBKG	TNFRSF1A	9606.ENSP00000483825	9606.ENSP00000162749	0	0.879	0.9	0.987
IKBKG	RIPK2	9606.ENSP00000483825	9606.ENSP00000220751	0.059	0.495	0.9	0.948
IKBKG	NFKB1	9606.ENSP00000483825	9606.ENSP00000226574	0.062	0.532	0.9	0.952
IKBKG	TLR2	9606.ENSP00000483825	9606.ENSP00000260010	0	0.058	0.9	0.901
IKBKG	PRKCQ	9606.ENSP00000483825	9606.ENSP00000263125	0	0.27	0.9	0.923
IKBKG	PRKCB	9606.ENSP00000483825	9606.ENSP00000305355	0	0.178	0.9	0.914
IKBKG	PRKCE	9606.ENSP00000483825	9606.ENSP00000306124	0	0.27	0.9	0.923
IKBKG	PDPK1	9606.ENSP00000483825	9606.ENSP00000344220	0	0	0.9	0.9
IKBKG	TLR1	9606.ENSP00000483825	9606.ENSP00000354932	0	0.058	0.9	0.901
IKBKG	SRC	9606.ENSP00000483825	9606.ENSP00000362680	0.07	0.495	0	0.51
IKBKG	PRKCZ	9606.ENSP00000483825	9606.ENSP00000367830	0	0.059	0.9	0.901
IKBKG	RELA	9606.ENSP00000483825	9606.ENSP00000384273	0.101	0.27	0.9	0.928
IKBKG	TNF	9606.ENSP00000483825	9606.ENSP00000398698	0	0.892	0.9	0.988
IKBKG	PRKCA	9606.ENSP00000483825	9606.ENSP00000408695	0	0	0.9	0.9
IKBKG	NTRK1	9606.ENSP00000483825	9606.ENSP00000431418	0	0.059	0.9	0.901
IL6ST	MAPK1	9606.ENSP00000370698	9606.ENSP00000215832	0	0	0.9	0.9
IL6ST	MAPK3	9606.ENSP00000370698	9606.ENSP00000263025	0	0	0.9	0.9
IL6ST	PIK3CA	9606.ENSP00000370698	9606.ENSP00000263967	0.08	0	0.6	0.616
IL6ST	STAT3	9606.ENSP00000370698	9606.ENSP00000264657	0.064	0.317	0.9	0.93
IL6ST	PIK3CB	9606.ENSP00000370698	9606.ENSP00000289153	0.052	0	0.6	0.604
IL6ST	MAP2K1	9606.ENSP00000370698	9606.ENSP00000302486	0	0	0.9	0.9
IL6ST	PTPN11	9606.ENSP00000370698	9606.ENSP00000340944	0.062	0.878	0.9	0.987
IL6ST	JAK1	9606.ENSP00000370698	9606.ENSP00000343204	0.09	0.879	0.9	0.988
IL6ST	PIK3CD	9606.ENSP00000370698	9606.ENSP00000366563	0.052	0	0.6	0.604
IL6ST	PIK3R1	9606.ENSP00000370698	9606.ENSP00000428056	0.063	0.27	0.6	0.702
IL6ST	JAK3	9606.ENSP00000370698	9606.ENSP00000391676	0.062	0.376	0.8	0.872
IL6ST	TYK2	9606.ENSP00000370698	9606.ENSP00000431885	0	0.281	0.9	0.925
IL6ST	JAK2	9606.ENSP00000370698	9606.ENSP00000371067	0.062	0.814	0.9	0.981
INSR	RPS6KB1	9606.ENSP00000303830	9606.ENSP00000225577	0.056	0.119	0.9	0.909
INSR	PIK3CA	9606.ENSP00000303830	9606.ENSP00000263967	0.063	0.146	0.9	0.912
INSR	PIK3CB	9606.ENSP00000303830	9606.ENSP00000289153	0.063	0.146	0.8	0.825
INSR	JAK1	9606.ENSP00000303830	9606.ENSP00000343204	0	0.471	0	0.471
INSR	LCK	9606.ENSP00000303830	9606.ENSP00000337825	0	0	0.5	0.499
INSR	PRKCD	9606.ENSP00000303830	9606.ENSP00000378217	0.063	0.526	0	0.537
INSR	JAK2	9606.ENSP00000303830	9606.ENSP00000371067	0	0.499	0	0.499
INSR	SRC	9606.ENSP00000303830	9606.ENSP00000362680	0	0.302	0.5	0.636
INSR	PTPN6	9606.ENSP00000303830	9606.ENSP00000391592	0.062	0.509	0	0.52
INSR	PRKCB	9606.ENSP00000303830	9606.ENSP00000305355	0.048	0.061	0.8	0.805
INSR	PRKCE	9606.ENSP00000303830	9606.ENSP00000306124	0.063	0.117	0.8	0.82
INSR	PIK3CD	9606.ENSP00000303830	9606.ENSP00000366563	0.063	0.146	0.8	0.825
INSR	PTPRF	9606.ENSP00000303830	9606.ENSP00000353030	0.062	0.353	0.8	0.868

INSR	PPP1CC	9606.ENSP00000303830	9606.ENSP00000341779	0	0.117	0.9	0.907
INSR	PRKCZ	9606.ENSP00000303830	9606.ENSP00000367830	0	0	0.9	0.9
INSR	PDPK1	9606.ENSP00000303830	9606.ENSP00000344220	0.062	0.145	0.9	0.912
INSR	PTPN2	9606.ENSP00000303830	9606.ENSP00000311857	0.062	0.571	0.9	0.956
INSR	PTPN11	9606.ENSP00000303830	9606.ENSP00000340944	0.062	0.866	0.9	0.986
INSR	PIK3R1	9606.ENSP00000303830	9606.ENSP00000428056	0.062	0.895	0.9	0.989
INSR	PTPN1	9606.ENSP00000303830	9606.ENSP00000360683	0.062	0.985	0.9	0.998
ITGAL	PTK2	9606.ENSP00000349252	9606.ENSP00000341189	0.062	0	0.6	0.608
ITGAL	LYN	9606.ENSP00000349252	9606.ENSP00000428924	0.118	0.075	0.36	0.432
ITGAL	SRC	9606.ENSP00000349252	9606.ENSP00000362680	0	0.075	0.6	0.614
ITGAL	PTK2B	9606.ENSP00000349252	9606.ENSP00000380638	0.107	0	0.8	0.813
ITGAL	PTPRC	9606.ENSP00000349252	9606.ENSP00000411355	0.648	0.313	0	0.748
ITGAL	ITGB2	9606.ENSP00000349252	9606.ENSP00000380948	0.418	0.87	0.9	0.991
ITGB2	NFKB1	9606.ENSP00000380948	9606.ENSP00000226574	0.098	0.056	0.9	0.907
ITGB2	S1PR4	9606.ENSP00000380948	9606.ENSP00000246115	0.555	0	0	0.555
ITGB2	TLR2	9606.ENSP00000380948	9606.ENSP00000260010	0.552	0.07	0	0.565
ITGB2	PIK3CA	9606.ENSP00000380948	9606.ENSP00000263967	0.079	0	0.6	0.615
ITGB2	PIK3CB	9606.ENSP00000380948	9606.ENSP00000289153	0.079	0	0.6	0.615
ITGB2	PTK2	9606.ENSP00000380948	9606.ENSP00000341189	0.102	0.378	0.6	0.757
ITGB2	MPEG1	9606.ENSP00000380948	9606.ENSP00000354335	0.643	0	0	0.643
ITGB2	MMP9	9606.ENSP00000380948	9606.ENSP00000361405	0.29	0.069	0.9	0.928
ITGB2	PLAU	9606.ENSP00000380948	9606.ENSP00000361850	0.063	0	0.9	0.902
ITGB2	SRC	9606.ENSP00000380948	9606.ENSP00000362680	0.063	0.07	0.6	0.62
ITGB2	SYK	9606.ENSP00000380948	9606.ENSP00000364907	0.34	0.501	0	0.657
ITGB2	PIK3CD	9606.ENSP00000380948	9606.ENSP00000366563	0.181	0	0.6	0.658
ITGB2	PTK2B	9606.ENSP00000380948	9606.ENSP00000380638	0.214	0.423	0.8	0.901
ITGB2	PTPN6	9606.ENSP00000380948	9606.ENSP00000391592	0.349	0.139	0	0.416
ITGB2	PIK3R1	9606.ENSP00000380948	9606.ENSP00000428056	0	0	0.6	0.6
ITGB2	LYN	9606.ENSP00000380948	9606.ENSP00000428924	0.205	0.318	0.36	0.623
ITGB2	PTPRC	9606.ENSP00000380948	9606.ENSP00000411355	0.764	0.163	0	0.794
ITK	P2RY10	9606.ENSP00000398655	9606.ENSP00000171757	0.669	0	0	0.669
ITK	PIK3CA	9606.ENSP00000398655	9606.ENSP00000263967	0.062	0.104	0.8	0.817
ITK	TXK	9606.ENSP00000398655	9606.ENSP00000264316	0.225	0	0.8	0.838
ITK	PIK3CB	9606.ENSP00000398655	9606.ENSP00000289153	0.062	0.104	0.8	0.817
ITK	LCK	9606.ENSP00000398655	9606.ENSP00000337825	0.408	0.213	0.9	0.949
ITK	PIK3CG	9606.ENSP00000398655	9606.ENSP00000352121	0.118	0.104	0.8	0.828
ITK	SRC	9606.ENSP00000398655	9606.ENSP00000362680	0	0.27	0.9	0.923
ITK	SYK	9606.ENSP00000398655	9606.ENSP00000364907	0.203	0	0.9	0.916
ITK	PIK3CD	9606.ENSP00000398655	9606.ENSP00000366563	0.089	0.104	0.8	0.822
ITK	YES1	9606.ENSP00000398655	9606.ENSP00000462468	0	0	0.9	0.9
ITK	LYN	9606.ENSP00000398655	9606.ENSP00000428924	0.062	0	0.9	0.902
ITK	PIK3R1	9606.ENSP00000398655	9606.ENSP00000428056	0	0.316	0.8	0.857
JAK1	TNFRSF1A	9606.ENSP00000343204	9606.ENSP00000162749	0	0.497	0	0.497
JAK1	MAPK1	9606.ENSP00000343204	9606.ENSP00000215832	0.168	0.148	0.9	0.922

JAK1	MAPK14	9606.ENSP00000343204	9606.ENSP00000229795	0.063	0.148	0.9	0.913
JAK1	PDGFRA	9606.ENSP00000343204	9606.ENSP00000257290	0	0.279	0.9	0.924
JAK1	MDM2	9606.ENSP00000343204	9606.ENSP00000258149	0.062	0.494	0	0.505
JAK1	PDGFRB	9606.ENSP00000343204	9606.ENSP00000261799	0	0.279	0.8	0.849
JAK1	MAPK3	9606.ENSP00000343204	9606.ENSP00000263025	0.111	0.148	0.9	0.917
JAK1	KDR	9606.ENSP00000343204	9606.ENSP00000263923	0	0.122	0.8	0.816
JAK1	PIK3CA	9606.ENSP00000343204	9606.ENSP00000263967	0.062	0.104	0.9	0.908
JAK1	STAT3	9606.ENSP00000343204	9606.ENSP00000264657	0.097	0.887	0.9	0.988
JAK1	PIK3CB	9606.ENSP00000343204	9606.ENSP00000289153	0.062	0.104	0.6	0.634
JAK1	STAT6	9606.ENSP00000343204	9606.ENSP00000300134	0.062	0.518	0.9	0.95
JAK1	MAP2K1	9606.ENSP00000343204	9606.ENSP00000302486	0.063	0.178	0.9	0.916
JAK1	PRKCB	9606.ENSP00000343204	9606.ENSP00000305355	0	0.061	0.9	0.902
JAK1	PRKCE	9606.ENSP00000343204	9606.ENSP00000306124	0.063	0.117	0.9	0.91
JAK1	MAP3K11	9606.ENSP00000343204	9606.ENSP00000309597	0	0	0.9	0.9
JAK1	PTPN2	9606.ENSP00000343204	9606.ENSP00000311857	0.062	0.3	0.9	0.928
JAK1	MET	9606.ENSP00000343204	9606.ENSP00000317272	0.064	0.074	0.8	0.811
JAK1	LCK	9606.ENSP00000343204	9606.ENSP00000337825	0.052	0	0.9	0.901
JAK1	PTPN11	9606.ENSP00000343204	9606.ENSP00000340944	0.062	0.799	0.9	0.979
JAK1	PIK3CD	9606.ENSP00000343204	9606.ENSP00000366563	0.062	0.104	0.6	0.634
JAK1	PRKCD	9606.ENSP00000343204	9606.ENSP00000378217	0.063	0.117	0.9	0.91
JAK1	MAPK8	9606.ENSP00000343204	9606.ENSP00000378974	0.063	0.143	0.9	0.912
JAK1	PRKCZ	9606.ENSP00000343204	9606.ENSP00000367830	0	0.292	0.9	0.926
JAK1	JAK2	9606.ENSP00000343204	9606.ENSP00000371067	0.085	0.27	0.9	0.927
JAK1	SYK	9606.ENSP00000343204	9606.ENSP00000364907	0	0.213	0.9	0.917
JAK1	PTK2B	9606.ENSP00000343204	9606.ENSP00000380638	0.062	0.298	0.9	0.928
JAK1	TYK2	9606.ENSP00000343204	9606.ENSP00000431885	0.062	0.462	0.9	0.945
JAK1	JAK3	9606.ENSP00000343204	9606.ENSP00000391676	0.062	0.462	0.9	0.945
JAK1	PTPN6	9606.ENSP00000343204	9606.ENSP00000391592	0.062	0.483	0.9	0.947
JAK1	PIK3R1	9606.ENSP00000343204	9606.ENSP00000428056	0.062	0.263	0.9	0.924
JAK1	PTPN1	9606.ENSP00000343204	9606.ENSP00000360683	0.062	0.148	0.9	0.913
JAK2	TNFRSF1A	9606.ENSP00000371067	9606.ENSP00000162749	0.049	0.497	0	0.501
JAK2	MAPK1	9606.ENSP00000371067	9606.ENSP00000215832	0.065	0.148	0.9	0.913
JAK2	NFKB1	9606.ENSP00000371067	9606.ENSP00000226574	0.09	0.12	0.9	0.912
JAK2	SPHK2	9606.ENSP00000371067	9606.ENSP00000245222	0	0.056	0.9	0.901
JAK2	RAF1	9606.ENSP00000371067	9606.ENSP00000251849	0.053	0.542	0.3	0.67
JAK2	PDGFRA	9606.ENSP00000371067	9606.ENSP00000257290	0.062	0.122	0.8	0.82
JAK2	PDGFRB	9606.ENSP00000371067	9606.ENSP00000261799	0	0.279	0.9	0.924
JAK2	MAPK3	9606.ENSP00000371067	9606.ENSP00000263025	0.065	0.148	0.9	0.913
JAK2	KDR	9606.ENSP00000371067	9606.ENSP00000263923	0	0.122	0.8	0.816
JAK2	PIK3CA	9606.ENSP00000371067	9606.ENSP00000263967	0.09	0.104	0.9	0.911
JAK2	STAT3	9606.ENSP00000371067	9606.ENSP00000264657	0.053	0.884	0.9	0.988
JAK2	KIT	9606.ENSP00000371067	9606.ENSP00000288135	0	0.507	0.9	0.948
JAK2	PIK3CB	9606.ENSP00000371067	9606.ENSP00000289153	0.062	0.104	0.9	0.908
JAK2	STAT6	9606.ENSP00000371067	9606.ENSP00000300134	0.058	0.14	0.9	0.911

JAK2	MAP2K1	9606.ENSP00000371067	9606.ENSP00000302486	0	0.178	0.5	0.571
JAK2	MAP3K11	9606.ENSP00000371067	9606.ENSP00000309597	0	0	0.9	0.9
JAK2	PTPN2	9606.ENSP00000371067	9606.ENSP00000311857	0.062	0.152	0.9	0.913
JAK2	MET	9606.ENSP00000371067	9606.ENSP00000317272	0.064	0.074	0.8	0.811
JAK2	NOS2	9606.ENSP00000371067	9606.ENSP00000327251	0	0.077	0.9	0.903
JAK2	LCK	9606.ENSP00000371067	9606.ENSP00000337825	0.055	0	0.9	0.901
JAK2	PTPN11	9606.ENSP00000371067	9606.ENSP00000340944	0.062	0.8	0.9	0.979
JAK2	PTK2	9606.ENSP00000371067	9606.ENSP00000341189	0	0.505	0.9	0.948
JAK2	PIK3CG	9606.ENSP00000371067	9606.ENSP00000352121	0.106	0.104	0.9	0.912
JAK2	PTPN1	9606.ENSP00000371067	9606.ENSP00000360683	0.062	0.856	0.9	0.985
JAK2	SRC	9606.ENSP00000371067	9606.ENSP00000362680	0.062	0.345	0.9	0.933
JAK2	PIK3CD	9606.ENSP00000371067	9606.ENSP00000366563	0.062	0.104	0.8	0.817
JAK2	YES1	9606.ENSP00000371067	9606.ENSP00000462468	0.062	0.302	0.6	0.715
JAK2	LYN	9606.ENSP00000371067	9606.ENSP00000428924	0.062	0.27	0.8	0.851
JAK2	PRKCD	9606.ENSP00000371067	9606.ENSP00000378217	0.063	0.117	0.9	0.91
JAK2	TYK2	9606.ENSP00000371067	9606.ENSP00000431885	0.062	0.27	0.9	0.925
JAK2	RELA	9606.ENSP00000371067	9606.ENSP00000384273	0	0.056	0.9	0.901
JAK2	JAK3	9606.ENSP00000371067	9606.ENSP00000391676	0	0.462	0.9	0.943
JAK2	PIK3R1	9606.ENSP00000371067	9606.ENSP00000428056	0.073	0.752	0.9	0.975
JAK2	PTPN6	9606.ENSP00000371067	9606.ENSP00000391592	0.082	0.505	0.9	0.95
JAK3	MAPK1	9606.ENSP00000391676	9606.ENSP00000215832	0.065	0.148	0.9	0.913
JAK3	MAPK14	9606.ENSP00000391676	9606.ENSP00000229795	0.062	0.148	0.9	0.913
JAK3	PDGFRA	9606.ENSP00000391676	9606.ENSP00000257290	0.049	0.122	0.6	0.636
JAK3	PDGFRB	9606.ENSP00000391676	9606.ENSP00000261799	0	0.122	0.6	0.633
JAK3	MAPK3	9606.ENSP00000391676	9606.ENSP00000263025	0.065	0.148	0.9	0.913
JAK3	PIK3CA	9606.ENSP00000391676	9606.ENSP00000263967	0.062	0.104	0.9	0.908
JAK3	STAT3	9606.ENSP00000391676	9606.ENSP00000264657	0.062	0.517	0.9	0.95
JAK3	PIK3CB	9606.ENSP00000391676	9606.ENSP00000289153	0.062	0.104	0.6	0.634
JAK3	STAT6	9606.ENSP00000391676	9606.ENSP00000300134	0.076	0.345	0.9	0.934
JAK3	MAP2K1	9606.ENSP00000391676	9606.ENSP00000302486	0	0.178	0.9	0.914
JAK3	PRKCB	9606.ENSP00000391676	9606.ENSP00000305355	0.077	0.061	0.9	0.905
JAK3	PRKCE	9606.ENSP00000391676	9606.ENSP00000306124	0.063	0.117	0.9	0.91
JAK3	PTPN2	9606.ENSP00000391676	9606.ENSP00000311857	0.062	0.304	0.9	0.929
JAK3	LCK	9606.ENSP00000391676	9606.ENSP00000337825	0.107	0.213	0.9	0.923
JAK3	PTPN11	9606.ENSP00000391676	9606.ENSP00000340944	0.062	0.183	0.9	0.916
JAK3	SYK	9606.ENSP00000391676	9606.ENSP00000364907	0.087	0	0.9	0.904
JAK3	PIK3CD	9606.ENSP00000391676	9606.ENSP00000366563	0.117	0.104	0.6	0.656
JAK3	PRKCZ	9606.ENSP00000391676	9606.ENSP00000367830	0	0.108	0.9	0.906
JAK3	MAPK8	9606.ENSP00000391676	9606.ENSP00000378974	0.063	0.143	0.9	0.912
JAK3	PTK2B	9606.ENSP00000391676	9606.ENSP00000380638	0.085	0.298	0.9	0.93
JAK3	PTPN6	9606.ENSP00000391676	9606.ENSP00000391592	0.107	0.243	0.9	0.926
JAK3	TYK2	9606.ENSP00000391676	9606.ENSP00000431885	0.062	0.27	0.9	0.925
JAK3	PIK3R1	9606.ENSP00000391676	9606.ENSP00000428056	0	0.263	0.9	0.923
JUN	MAPK1	9606.ENSP00000360266	9606.ENSP00000215832	0	0.867	0.9	0.986

JUN	RPS6KB1	9606.ENSP00000360266	9606.ENSP00000225577	0	0	0.8	0.8
JUN	MAPK14	9606.ENSP00000360266	9606.ENSP00000229795	0	0.4	0.9	0.937
JUN	NR3C1	9606.ENSP00000360266	9606.ENSP00000231509	0	0.493	0.9	0.947
JUN	PIN1	9606.ENSP00000360266	9606.ENSP00000247970	0	0.486	0	0.485
JUN	MAPK3	9606.ENSP00000360266	9606.ENSP00000263025	0	0.5	0.9	0.947
JUN	KAT2B	9606.ENSP00000360266	9606.ENSP00000263754	0.062	0.417	0.9	0.94
JUN	PPARG	9606.ENSP00000360266	9606.ENSP00000287820	0	0.066	0.9	0.902
JUN	NOS3	9606.ENSP00000360266	9606.ENSP00000297494	0	0	0.9	0.9
JUN	LPL	9606.ENSP00000360266	9606.ENSP00000309757	0.063	0	0.9	0.902
JUN	MMP1	9606.ENSP00000360266	9606.ENSP00000322788	0	0.628	0	0.628
JUN	NOS2	9606.ENSP00000360266	9606.ENSP00000327251	0	0	0.9	0.9
JUN	MAPK10	9606.ENSP00000360266	9606.ENSP00000352157	0	0.961	0.9	0.995
JUN	KDM1A	9606.ENSP00000360266	9606.ENSP00000383042	0.066	0.094	0.65	0.677
JUN	MMP9	9606.ENSP00000360266	9606.ENSP00000361405	0	0.486	0	0.485
JUN	LPAR3	9606.ENSP00000360266	9606.ENSP00000395389	0	0	0.9	0.9
JUN	LPAR2	9606.ENSP00000360266	9606.ENSP00000443256	0	0	0.9	0.9
JUN	NR1H3	9606.ENSP00000360266	9606.ENSP00000477707	0	0.066	0.9	0.902
JUN	LPAR1	9606.ENSP00000360266	9606.ENSP00000363553	0	0	0.9	0.9
JUN	LDHA	9606.ENSP00000360266	9606.ENSP00000445175	0	0.129	0.9	0.909
JUN	LYN	9606.ENSP00000360266	9606.ENSP00000428924	0	0.057	0.9	0.901
JUN	SRC	9606.ENSP00000360266	9606.ENSP00000362680	0.065	0.057	0.8	0.808
JUN	SYK	9606.ENSP00000360266	9606.ENSP00000364907	0	0.057	0.9	0.901
JUN	SMARCA4	9606.ENSP00000360266	9606.ENSP00000395654	0	0.329	0.9	0.93
JUN	TNF	9606.ENSP00000360266	9606.ENSP00000398698	0	0	0.9	0.9
JUN	RELA	9606.ENSP00000360266	9606.ENSP00000384273	0.069	0.481	0.9	0.947
JUN	PPARA	9606.ENSP00000360266	9606.ENSP00000385523	0	0.066	0.9	0.902
JUN	MAPK8	9606.ENSP00000360266	9606.ENSP00000378974	0	0.998	0.9	0.999
JUN	NFE2L2	9606.ENSP00000360266	9606.ENSP00000380252	0	0.769	0.9	0.975
KAT2B	MAPK14	9606.ENSP00000263754	9606.ENSP00000229795	0.062	0.36	0.9	0.934
KAT2B	NR3C1	9606.ENSP00000263754	9606.ENSP00000231509	0.083	0.102	0.9	0.91
KAT2B	SIRT2	9606.ENSP00000263754	9606.ENSP00000249396	0.062	0.538	0	0.548
KAT2B	RARA	9606.ENSP00000263754	9606.ENSP00000254066	0	0.695	0.9	0.968
KAT2B	VHL	9606.ENSP00000263754	9606.ENSP00000256474	0	0.486	0	0.485
KAT2B	MDM2	9606.ENSP00000263754	9606.ENSP00000258149	0	0.838	0.9	0.983
KAT2B	PGR	9606.ENSP00000263754	9606.ENSP00000325120	0	0.518	0	0.518
KAT2B	RXRG	9606.ENSP00000263754	9606.ENSP00000352900	0	0.102	0.6	0.625
KAT2B	RXRB	9606.ENSP00000263754	9606.ENSP00000363817	0	0.102	0.6	0.625
KAT2B	THRA	9606.ENSP00000263754	9606.ENSP00000264637	0.07	0.102	0.6	0.636
KAT2B	THRB	9606.ENSP00000263754	9606.ENSP00000379904	0.062	0.102	0.6	0.633
KAT2B	NCOR2	9606.ENSP00000263754	9606.ENSP00000384018	0.111	0.095	0.65	0.693
KAT2B	NCOR1	9606.ENSP00000263754	9606.ENSP00000268712	0.111	0.095	0.65	0.693
KAT2B	SMARCA2	9606.ENSP00000263754	9606.ENSP00000265773	0.17	0.539	0	0.601
KAT2B	RARG	9606.ENSP00000263754	9606.ENSP00000388510	0	0.102	0.9	0.906
KAT2B	RARB	9606.ENSP00000263754	9606.ENSP00000332296	0	0.102	0.9	0.906

KAT2B	RXRA	9606.ENSP00000263754	9606.ENSP00000419692	0.062	0.102	0.9	0.908
KAT2B	PTGDR	9606.ENSP00000263754	9606.ENSP00000303424	0.062	0	0.9	0.902
KAT2B	RELA	9606.ENSP00000263754	9606.ENSP00000384273	0	0.234	0.9	0.92
KAT2B	SMARCA4	9606.ENSP00000263754	9606.ENSP00000395654	0.145	0.395	0.9	0.943
KCNA3	KCNQ1	9606.ENSP00000358784	9606.ENSP00000155840	0.107	0.16	0.5	0.592
KCNA3	KCNA5	9606.ENSP00000358784	9606.ENSP00000252321	0	0.479	0.3	0.619
KCNA3	KCNH2	9606.ENSP00000358784	9606.ENSP00000262186	0.049	0	0.5	0.504
KCNA5	KCNQ1	9606.ENSP00000252321	9606.ENSP00000155840	0.062	0.16	0.5	0.571
KCNA5	SRC	9606.ENSP00000252321	9606.ENSP00000362680	0.062	0.486	0	0.497
KCNA5	KCNH2	9606.ENSP00000252321	9606.ENSP00000262186	0.09	0	0.5	0.525
KCNE1	KCNQ1	9606.ENSP00000337255	9606.ENSP00000155840	0.062	0.756	0.8	0.95
KCNE1	KCNH2	9606.ENSP00000337255	9606.ENSP00000262186	0	0.27	0.6	0.695
KCNH2	KCNQ1	9606.ENSP00000262186	9606.ENSP00000155840	0.054	0	0.5	0.506
KCNK2	KCNK3	9606.ENSP00000394033	9606.ENSP00000306275	0.063	0	0.6	0.609
KCNN1	KCNN4	9606.ENSP00000476519	9606.ENSP00000262888	0	0	0.8	0.8
KCNN1	PRKCG	9606.ENSP00000476519	9606.ENSP00000263431	0.161	0	0.6	0.65
KCNN1	PRKCB	9606.ENSP00000476519	9606.ENSP00000305355	0.073	0	0.6	0.613
KCNN1	PRKCA	9606.ENSP00000476519	9606.ENSP00000408695	0.052	0	0.6	0.604
KCNN1	KCNN2	9606.ENSP00000476519	9606.ENSP00000427120	0.062	0	0.8	0.804
KCNN1	KCNN3	9606.ENSP00000476519	9606.ENSP00000481848	0.066	0	0.8	0.805
KCNN2	KCNN4	9606.ENSP00000427120	9606.ENSP00000262888	0	0	0.8	0.8
KCNN2	PRKCG	9606.ENSP00000427120	9606.ENSP00000263431	0.062	0	0.6	0.608
KCNN2	PRKCB	9606.ENSP00000427120	9606.ENSP00000305355	0.062	0	0.6	0.608
KCNN2	PRKCA	9606.ENSP00000427120	9606.ENSP00000408695	0.052	0	0.6	0.604
KCNN2	KCNN3	9606.ENSP00000427120	9606.ENSP00000481848	0.06	0	0.8	0.803
KCNN3	KCNN4	9606.ENSP00000481848	9606.ENSP00000262888	0	0	0.8	0.8
KCNN3	PRKCG	9606.ENSP00000481848	9606.ENSP00000263431	0.072	0	0.6	0.613
KCNN3	PRKCB	9606.ENSP00000481848	9606.ENSP00000305355	0.062	0	0.6	0.608
KCNN3	PRKCA	9606.ENSP00000481848	9606.ENSP00000408695	0.06	0	0.6	0.607
KCNN4	KCNQ1	9606.ENSP00000262888	9606.ENSP00000155840	0.09	0	0.8	0.81
KCNN4	PRKCB	9606.ENSP00000262888	9606.ENSP00000305355	0	0	0.6	0.6
KCNN4	PRKCA	9606.ENSP00000262888	9606.ENSP00000408695	0	0	0.6	0.6
KCNN4	PRKCG	9606.ENSP00000262888	9606.ENSP00000263431	0	0	0.6	0.6
KDM1A	NR1H2	9606.ENSP00000383042	9606.ENSP00000253727	0	0.083	0.6	0.617
KDM1A	PPARG	9606.ENSP00000383042	9606.ENSP00000287820	0	0.082	0.65	0.664
KDM1A	PRKDC	9606.ENSP00000383042	9606.ENSP00000313420	0.097	0	0.9	0.905
KDM1A	METAP2	9606.ENSP00000383042	9606.ENSP00000325312	0.495	0	0	0.495
KDM1A	KDM4A	9606.ENSP00000383042	9606.ENSP00000361473	0.072	0.169	0.65	0.706
KDM1A	RXRB	9606.ENSP00000383042	9606.ENSP00000363817	0	0.082	0.6	0.617
KDM1A	RXRA	9606.ENSP00000383042	9606.ENSP00000419692	0.054	0.082	0.6	0.622
KDM1A	NR1H3	9606.ENSP00000383042	9606.ENSP00000477707	0	0.083	0.6	0.617
KDM1A	SMARCA4	9606.ENSP00000383042	9606.ENSP00000395654	0.145	0.372	0	0.44
KDM4A	NR1H2	9606.ENSP00000361473	9606.ENSP00000253727	0.049	0.145	0.6	0.646
KDM4A	NCOR1	9606.ENSP00000361473	9606.ENSP00000268712	0.135	0.486	0	0.536

KDM4A	RXRB	9606.ENSP00000361473	9606.ENSP00000363817	0.049	0.077	0.6	0.618
KDM4A	RXRA	9606.ENSP00000361473	9606.ENSP00000419692	0.049	0.077	0.6	0.618
KDM4A	NR1H3	9606.ENSP00000361473	9606.ENSP00000477707	0.049	0.145	0.6	0.646
KDR	MAPK14	9606.ENSP00000263923	9606.ENSP00000229795	0	0.081	0.9	0.904
KDR	MET	9606.ENSP00000263923	9606.ENSP00000317272	0	0.495	0	0.495
KDR	PIK3CD	9606.ENSP00000263923	9606.ENSP00000366563	0	0.306	0.8	0.855
KDR	PIK3CB	9606.ENSP00000263923	9606.ENSP00000289153	0	0.306	0.8	0.855
KDR	PTPN2	9606.ENSP00000263923	9606.ENSP00000311857	0	0.06	0.9	0.901
KDR	PTK2B	9606.ENSP00000263923	9606.ENSP00000380638	0	0.124	0.9	0.908
KDR	RAC1	9606.ENSP00000263923	9606.ENSP00000348461	0	0.101	0.9	0.906
KDR	ROCK1	9606.ENSP00000263923	9606.ENSP00000382697	0.055	0.122	0.9	0.909
KDR	ROCK2	9606.ENSP00000263923	9606.ENSP00000317985	0.055	0.122	0.9	0.909
KDR	PTK2	9606.ENSP00000263923	9606.ENSP00000341189	0.065	0.124	0.9	0.91
KDR	PIK3R1	9606.ENSP00000263923	9606.ENSP00000428056	0.054	0.355	0.9	0.933
KDR	PIK3CA	9606.ENSP00000263923	9606.ENSP00000263967	0.053	0.306	0.9	0.928
KDR	NOS3	9606.ENSP00000263923	9606.ENSP00000297494	0.062	0	0.9	0.902
KDR	PTPN6	9606.ENSP00000263923	9606.ENSP00000391592	0.063	0.843	0.9	0.984
KDR	PTPN11	9606.ENSP00000263923	9606.ENSP00000340944	0.063	0.543	0.9	0.953
KDR	SRC	9606.ENSP00000263923	9606.ENSP00000362680	0.062	0.889	0.9	0.988
KIF11	PCNA	9606.ENSP00000260731	9606.ENSP00000368458	0.736	0.061	0	0.741
KIF11	TYMS	9606.ENSP00000260731	9606.ENSP00000315644	0.897	0	0	0.897
KIF11	POLA1	9606.ENSP00000260731	9606.ENSP00000368349	0.872	0	0	0.872
KIF11	MELK	9606.ENSP00000260731	9606.ENSP00000298048	0.907	0	0	0.907
KIF11	TOP2A	9606.ENSP00000260731	9606.ENSP00000411532	0.968	0	0	0.968
KIF11	PLK1	9606.ENSP00000260731	9606.ENSP00000300093	0.962	0.143	0	0.966
KIF11	TTK	9606.ENSP00000260731	9606.ENSP00000358813	0.98	0.348	0	0.986
KIT	RPS6KB1	9606.ENSP00000288135	9606.ENSP00000225577	0	0.152	0.9	0.911
KIT	PIK3CA	9606.ENSP00000288135	9606.ENSP00000263967	0	0.345	0.9	0.931
KIT	TXK	9606.ENSP00000288135	9606.ENSP00000264316	0	0.426	0	0.426
KIT	STAT3	9606.ENSP00000288135	9606.ENSP00000264657	0	0.111	0.9	0.907
KIT	PTPRO	9606.ENSP00000288135	9606.ENSP00000281171	0	0.284	0.9	0.925
KIT	RAC1	9606.ENSP00000288135	9606.ENSP00000348461	0	0.078	0.5	0.519
KIT	LCK	9606.ENSP00000288135	9606.ENSP00000337825	0	0.429	0.5	0.702
KIT	YES1	9606.ENSP00000288135	9606.ENSP00000462468	0	0.429	0.6	0.762
KIT	PIK3CG	9606.ENSP00000288135	9606.ENSP00000352121	0	0.698	0	0.698
KIT	SRC	9606.ENSP00000288135	9606.ENSP00000362680	0	0.434	0.5	0.705
KIT	PRKCA	9606.ENSP00000288135	9606.ENSP00000408695	0	0.213	0.8	0.835
KIT	PIK3CB	9606.ENSP00000288135	9606.ENSP00000289153	0	0.101	0.8	0.812
KIT	PIK3CD	9606.ENSP00000288135	9606.ENSP00000366563	0	0.101	0.8	0.812
KIT	MAPK8	9606.ENSP00000288135	9606.ENSP00000378974	0	0.127	0.9	0.908
KIT	PIK3C2B	9606.ENSP00000288135	9606.ENSP00000356155	0	0.097	0.9	0.905
KIT	LYN	9606.ENSP00000288135	9606.ENSP00000428924	0.062	0.655	0.9	0.964
KIT	PTPN6	9606.ENSP00000288135	9606.ENSP00000391592	0.063	0.681	0.9	0.967
KIT	PTPN11	9606.ENSP00000288135	9606.ENSP00000340944	0.063	0.631	0.9	0.962

KIT	PIK3R1	9606.ENSP00000288135	9606.ENSP00000428056	0	0.888	0.9	0.988
LAP3	LTA4H	9606.ENSP00000226299	9606.ENSP00000228740	0.083	0.391	0	0.417
LAP3	QDPR	9606.ENSP00000226299	9606.ENSP00000281243	0.062	0.524	0	0.534
LAP3	NDUFS5	9606.ENSP00000226299	9606.ENSP00000362060	0.06	0.71	0	0.715
LCK	MAPK1	9606.ENSP00000337825	9606.ENSP00000215832	0	0.713	0.9	0.97
LCK	NFKB1	9606.ENSP00000337825	9606.ENSP00000226574	0.074	0.12	0.9	0.911
LCK	MAPK14	9606.ENSP00000337825	9606.ENSP00000229795	0	0.148	0.9	0.911
LCK	PDGFRB	9606.ENSP00000337825	9606.ENSP00000261799	0	0.122	0.9	0.908
LCK	MAPK3	9606.ENSP00000337825	9606.ENSP00000263025	0	0.3	0.9	0.927
LCK	PRKCQ	9606.ENSP00000337825	9606.ENSP00000263125	0.139	0.706	0.9	0.972
LCK	PIK3CA	9606.ENSP00000337825	9606.ENSP00000263967	0.062	0.699	0.9	0.969
LCK	STAT3	9606.ENSP00000337825	9606.ENSP00000264657	0	0.71	0.9	0.969
LCK	PIK3CB	9606.ENSP00000337825	9606.ENSP00000289153	0.062	0.104	0.5	0.543
LCK	MAP2K1	9606.ENSP00000337825	9606.ENSP00000302486	0	0.178	0.9	0.914
LCK	PRKCB	9606.ENSP00000337825	9606.ENSP00000305355	0.116	0.061	0.9	0.909
LCK	S1PR1	9606.ENSP00000337825	9606.ENSP00000305416	0	0.069	0.9	0.902
LCK	PRKCE	9606.ENSP00000337825	9606.ENSP00000306124	0.063	0.117	0.9	0.91
LCK	PDPK1	9606.ENSP00000337825	9606.ENSP00000344220	0.06	0.145	0.65	0.694
LCK	MAPT	9606.ENSP00000337825	9606.ENSP00000340820	0	0.541	0	0.541
LCK	SRC	9606.ENSP00000337825	9606.ENSP00000362680	0	0	0.9	0.9
LCK	TYK2	9606.ENSP00000337825	9606.ENSP00000431885	0	0	0.9	0.9
LCK	PRKCZ	9606.ENSP00000337825	9606.ENSP00000367830	0	0.056	0.9	0.901
LCK	YES1	9606.ENSP00000337825	9606.ENSP00000462468	0	0.248	0.9	0.921
LCK	LYN	9606.ENSP00000337825	9606.ENSP00000428924	0.078	0.27	0.9	0.926
LCK	RELA	9606.ENSP00000337825	9606.ENSP00000384273	0.048	0.056	0.9	0.902
LCK	MAPK8	9606.ENSP00000337825	9606.ENSP00000378974	0.062	0.143	0.9	0.912
LCK	RAC1	9606.ENSP00000337825	9606.ENSP00000348461	0.063	0.14	0.9	0.912
LCK	PTK2B	9606.ENSP00000337825	9606.ENSP00000380638	0	0.298	0.9	0.926
LCK	PRKCD	9606.ENSP00000337825	9606.ENSP00000378217	0.063	0.275	0.9	0.926
LCK	PTPN11	9606.ENSP00000337825	9606.ENSP00000340944	0.062	0.243	0.9	0.922
LCK	PTK2	9606.ENSP00000337825	9606.ENSP00000341189	0	0.448	0.9	0.942
LCK	SYK	9606.ENSP00000337825	9606.ENSP00000364907	0.096	0.787	0.9	0.979
LCK	PIK3R1	9606.ENSP00000337825	9606.ENSP00000428056	0	0.698	0.9	0.968
LCK	PTPN6	9606.ENSP00000337825	9606.ENSP00000391592	0.117	0.837	0.9	0.984
LCK	PTPRC	9606.ENSP00000337825	9606.ENSP00000411355	0.195	0.88	0.9	0.989
LDHA	MIF	9606.ENSP00000445175	9606.ENSP00000215754	0.218	0.272	0	0.406
LDHA	LRRK2	9606.ENSP00000445175	9606.ENSP00000298910	0.062	0.486	0	0.497
LDHA	PKM	9606.ENSP00000445175	9606.ENSP00000320171	0.238	0.537	0.65	0.865
LDHA	MAPK10	9606.ENSP00000445175	9606.ENSP00000352157	0	0.769	0	0.769
LDHA	PCNA	9606.ENSP00000445175	9606.ENSP00000368458	0.207	0.291	0	0.413
LIMK1	ROCK2	9606.ENSP00000336740	9606.ENSP00000317985	0	0.322	0.9	0.929
LIMK1	RAC1	9606.ENSP00000336740	9606.ENSP00000348461	0	0.175	0.9	0.913
LIMK1	ROCK1	9606.ENSP00000336740	9606.ENSP00000382697	0	0.443	0.9	0.941
LIPA	STS	9606.ENSP00000337354	9606.ENSP00000217961	0	0	0.8	0.8

LIPC	LIPE	9606.ENSP00000299022	9606.ENSP00000244289	0.062	0	0.9	0.902
LIPC	LIPG	9606.ENSP00000299022	9606.ENSP00000261292	0.062	0	0.8	0.804
LIPC	MSR1	9606.ENSP00000299022	9606.ENSP00000262101	0.084	0	0.54	0.56
LIPC	MGLL	9606.ENSP00000299022	9606.ENSP00000265052	0	0	0.9	0.9
LIPC	PLA2G7	9606.ENSP00000299022	9606.ENSP00000274793	0	0	0.54	0.54
LIPC	LPL	9606.ENSP00000299022	9606.ENSP00000309757	0.064	0	0.9	0.902
LIPE	LIPG	9606.ENSP00000244289	9606.ENSP00000261292	0	0	0.9	0.9
LIPE	PLIN1	9606.ENSP00000244289	9606.ENSP00000300055	0.135	0.621	0.65	0.875
LIPG	LPL	9606.ENSP00000261292	9606.ENSP00000309757	0	0	0.9	0.9
LIPG	MGLL	9606.ENSP00000261292	9606.ENSP00000265052	0	0	0.9	0.9
LNPEP	PRKCZ	9606.ENSP00000231368	9606.ENSP00000367830	0.062	0	0.9	0.902
LNPEP	YWHAG	9606.ENSP00000231368	9606.ENSP00000306330	0.087	0.058	0.9	0.906
LNPEP	TNKS2	9606.ENSP00000231368	9606.ENSP00000360689	0.049	0.818	0	0.82
LPAR1	NFKB1	9606.ENSP00000363553	9606.ENSP00000226574	0	0.062	0.9	0.902
LPAR1	PIK3CB	9606.ENSP00000363553	9606.ENSP00000289153	0	0.056	0.9	0.901
LPAR1	PTK2	9606.ENSP00000363553	9606.ENSP00000341189	0	0	0.9	0.9
LPAR1	PIK3CG	9606.ENSP00000363553	9606.ENSP00000352121	0	0.056	0.6	0.606
LPAR1	RELA	9606.ENSP00000363553	9606.ENSP00000384273	0	0	0.9	0.9
LPAR1	LYN	9606.ENSP00000363553	9606.ENSP00000428924	0	0.056	0.9	0.901
LPAR1	LPAR2	9606.ENSP00000363553	9606.ENSP00000443256	0	0	0.9	0.9
LPAR1	LPAR3	9606.ENSP00000363553	9606.ENSP00000395389	0	0	0.9	0.9
LPAR1	PIK3R1	9606.ENSP00000363553	9606.ENSP00000428056	0	0.056	0.9	0.901
LPAR1	PTK2B	9606.ENSP00000363553	9606.ENSP00000380638	0	0	0.9	0.9
LPAR1	LPAR6	9606.ENSP00000363553	9606.ENSP00000367691	0.062	0	0.8	0.804
LPAR1	LPAR4	9606.ENSP00000363553	9606.ENSP00000408205	0	0	0.8	0.8
LPAR2	NFKB1	9606.ENSP00000443256	9606.ENSP00000226574	0	0.062	0.9	0.902
LPAR2	PIK3CB	9606.ENSP00000443256	9606.ENSP00000289153	0	0.056	0.9	0.901
LPAR2	PTK2	9606.ENSP00000443256	9606.ENSP00000341189	0	0	0.9	0.9
LPAR2	PIK3CG	9606.ENSP00000443256	9606.ENSP00000352121	0	0.056	0.6	0.606
LPAR2	LPAR6	9606.ENSP00000443256	9606.ENSP00000367691	0	0	0.8	0.8
LPAR2	PTK2B	9606.ENSP00000443256	9606.ENSP00000380638	0	0	0.9	0.9
LPAR2	RELA	9606.ENSP00000443256	9606.ENSP00000384273	0	0	0.9	0.9
LPAR2	LPAR3	9606.ENSP00000443256	9606.ENSP00000395389	0	0	0.9	0.9
LPAR2	LPAR4	9606.ENSP00000443256	9606.ENSP00000408205	0	0	0.8	0.8
LPAR2	PIK3R1	9606.ENSP00000443256	9606.ENSP00000428056	0	0.056	0.9	0.901
LPAR2	LYN	9606.ENSP00000443256	9606.ENSP00000428924	0	0.056	0.9	0.901
LPAR3	NFKB1	9606.ENSP00000395389	9606.ENSP00000226574	0	0.062	0.9	0.902
LPAR3	PIK3CB	9606.ENSP00000395389	9606.ENSP00000289153	0	0.056	0.9	0.901
LPAR3	PTK2	9606.ENSP00000395389	9606.ENSP00000341189	0	0	0.9	0.9
LPAR3	PIK3CG	9606.ENSP00000395389	9606.ENSP00000352121	0	0.056	0.6	0.606
LPAR3	LPAR6	9606.ENSP00000395389	9606.ENSP00000367691	0	0	0.8	0.8
LPAR3	RELA	9606.ENSP00000395389	9606.ENSP00000384273	0	0	0.9	0.9
LPAR3	LYN	9606.ENSP00000395389	9606.ENSP00000428924	0	0.056	0.9	0.901
LPAR3	PIK3R1	9606.ENSP00000395389	9606.ENSP00000428056	0	0.056	0.9	0.901

LPAR3	LPAR4	9606.ENSP00000395389	9606.ENSP00000408205	0	0	0.8	0.8
LPAR4	PIK3CG	9606.ENSP00000408205	9606.ENSP00000352121	0	0	0.6	0.6
LPAR4	LPAR6	9606.ENSP00000408205	9606.ENSP00000367691	0	0	0.8	0.8
LPAR4	RPS6KA5	9606.ENSP00000408205	9606.ENSP00000479667	0	0	0.9	0.9
LPAR6	PIK3CG	9606.ENSP00000367691	9606.ENSP00000352121	0.085	0	0.6	0.618
LPL	NR3C1	9606.ENSP00000309757	9606.ENSP00000231509	0	0	0.9	0.9
LPL	MSR1	9606.ENSP00000309757	9606.ENSP00000262101	0.063	0	0.54	0.55
LPL	MGLL	9606.ENSP00000309757	9606.ENSP00000265052	0	0	0.9	0.9
LPL	PLA2G7	9606.ENSP00000309757	9606.ENSP00000274793	0.062	0	0.54	0.55
LPL	PPARG	9606.ENSP00000309757	9606.ENSP00000287820	0.062	0	0.9	0.902
LPL	RELA	9606.ENSP00000309757	9606.ENSP00000384273	0	0	0.9	0.9
LPL	RXRA	9606.ENSP00000309757	9606.ENSP00000419692	0	0	0.9	0.9
LPL	NR1H3	9606.ENSP00000309757	9606.ENSP00000477707	0.062	0	0.9	0.902
LPL	PPARA	9606.ENSP00000309757	9606.ENSP00000385523	0.062	0	0.9	0.902
LRRK2	MAPK1	9606.ENSP00000298910	9606.ENSP00000215832	0	0.433	0	0.433
LRRK2	SPTLC1	9606.ENSP00000298910	9606.ENSP00000262554	0.062	0.494	0	0.505
LRRK2	MAPK3	9606.ENSP00000298910	9606.ENSP00000263025	0	0.433	0	0.433
LRRK2	PTPN1	9606.ENSP00000298910	9606.ENSP00000360683	0.063	0.432	0	0.445
LRRK2	PTPN11	9606.ENSP00000298910	9606.ENSP00000340944	0.063	0.432	0	0.445
LRRK2	MAP2K1	9606.ENSP00000298910	9606.ENSP00000302486	0	0.433	0	0.433
LRRK2	PCNA	9606.ENSP00000298910	9606.ENSP00000368458	0.062	0.497	0	0.508
LRRK2	PLK1	9606.ENSP00000298910	9606.ENSP00000300093	0.051	0.462	0	0.468
LRRK2	PKM	9606.ENSP00000298910	9606.ENSP00000320171	0	0.521	0	0.521
LRRK2	PLEC	9606.ENSP00000298910	9606.ENSP00000323856	0.064	0.578	0	0.588
LRRK2	MTOR	9606.ENSP00000298910	9606.ENSP00000354558	0.161	0.353	0	0.434
LRRK2	RAC1	9606.ENSP00000298910	9606.ENSP00000348461	0.062	0.648	0	0.656
LRRK2	PRKDC	9606.ENSP00000298910	9606.ENSP00000313420	0.161	0.712	0	0.748
LRRK2	MAPT	9606.ENSP00000298910	9606.ENSP00000340820	0	0.709	0	0.709
LRRK2	YWHAG	9606.ENSP00000298910	9606.ENSP00000306330	0.064	0.832	0	0.836
LSS	MGLL	9606.ENSP00000380837	9606.ENSP00000265052	0	0.401	0	0.4
LSS	SQLE	9606.ENSP00000380837	9606.ENSP00000265896	0.825	0	0.9	0.981
LSS	SREBF2	9606.ENSP00000380837	9606.ENSP00000354476	0.142	0	0.9	0.91
LYN	MAPK1	9606.ENSP00000428924	9606.ENSP00000215832	0	0.351	0.9	0.932
LYN	NFKB1	9606.ENSP00000428924	9606.ENSP00000226574	0.087	0.12	0.9	0.912
LYN	MAPK14	9606.ENSP00000428924	9606.ENSP00000229795	0	0.148	0.9	0.911
LYN	RAF1	9606.ENSP00000428924	9606.ENSP00000251849	0	0.149	0.9	0.911
LYN	PDGFRB	9606.ENSP00000428924	9606.ENSP00000261799	0	0.122	0.9	0.908
LYN	MAPK3	9606.ENSP00000428924	9606.ENSP00000263025	0	0.543	0.9	0.952
LYN	PRKCQ	9606.ENSP00000428924	9606.ENSP00000263125	0.063	0.275	0.9	0.926
LYN	PRKCG	9606.ENSP00000428924	9606.ENSP00000263431	0	0.061	0.8	0.804
LYN	PIK3CA	9606.ENSP00000428924	9606.ENSP00000263967	0.062	0.104	0.9	0.908
LYN	TXK	9606.ENSP00000428924	9606.ENSP00000264316	0.065	0	0.6	0.61
LYN	STAT3	9606.ENSP00000428924	9606.ENSP00000264657	0.062	0.539	0.9	0.952
LYN	PIK3CB	9606.ENSP00000428924	9606.ENSP00000289153	0.062	0.104	0.8	0.817

LYN	PRKCB	9606.ENSP00000428924	9606.ENSP00000305355	0.057	0.496	0.8	0.896
LYN	S1PR1	9606.ENSP00000428924	9606.ENSP00000305416	0.062	0.069	0.9	0.905
LYN	SPHK1	9606.ENSP00000428924	9606.ENSP00000313681	0	0.225	0.9	0.919
LYN	PTPN11	9606.ENSP00000428924	9606.ENSP00000340944	0.062	0.078	0.9	0.905
LYN	PTK2	9606.ENSP00000428924	9606.ENSP00000341189	0.062	0.695	0.9	0.968
LYN	RAC1	9606.ENSP00000428924	9606.ENSP00000348461	0	0.14	0.9	0.91
LYN	PIK3CG	9606.ENSP00000428924	9606.ENSP00000352121	0.106	0.519	0.6	0.813
LYN	PLA2G4A	9606.ENSP00000428924	9606.ENSP00000356436	0.062	0.112	0.9	0.909
LYN	SRC	9606.ENSP00000428924	9606.ENSP00000362680	0	0.486	0.9	0.946
LYN	SYK	9606.ENSP00000428924	9606.ENSP00000364907	0.244	0.462	0.9	0.955
LYN	PIK3CD	9606.ENSP00000428924	9606.ENSP00000366563	0.096	0.104	0.8	0.823
LYN	PRKCD	9606.ENSP00000428924	9606.ENSP00000378217	0.092	0.275	0.9	0.928
LYN	MAPK8	9606.ENSP00000428924	9606.ENSP00000378974	0.062	0.143	0.9	0.912
LYN	PTK2B	9606.ENSP00000428924	9606.ENSP00000380638	0.062	0.298	0.9	0.928
LYN	PTPN6	9606.ENSP00000428924	9606.ENSP00000391592	0.107	0.87	0.9	0.987
LYN	PRKCA	9606.ENSP00000428924	9606.ENSP00000408695	0	0.061	0.8	0.804
LYN	PTPRC	9606.ENSP00000428924	9606.ENSP00000411355	0.177	0.482	0.9	0.953
LYN	MME	9606.ENSP00000428924	9606.ENSP00000418525	0	0.486	0	0.485
LYN	PIK3R1	9606.ENSP00000428924	9606.ENSP00000428056	0	0.216	0.9	0.918
LYN	YES1	9606.ENSP00000428924	9606.ENSP00000462468	0	0	0.9	0.9
LYPLA1	PLA2G2C	9606.ENSP00000320043	9606.ENSP00000247992	0	0	0.65	0.65
LYPLA1	NOS3	9606.ENSP00000320043	9606.ENSP00000297494	0	0	0.9	0.9
LYPLA1	PLA2G1B	9606.ENSP00000320043	9606.ENSP00000312286	0	0	0.65	0.65
LYPLA1	MDH1	9606.ENSP00000320043	9606.ENSP00000438144	0.428	0	0	0.428
LYPLA1	PLA2G4B	9606.ENSP00000320043	9606.ENSP00000396045	0	0	0.65	0.65
LYPLA1	PLA2G5	9606.ENSP00000320043	9606.ENSP00000364249	0	0	0.65	0.65
LYPLA1	PLA2G2A	9606.ENSP00000320043	9606.ENSP00000383364	0	0	0.65	0.65
LYPLA1	PLA2G10	9606.ENSP00000320043	9606.ENSP00000393847	0	0	0.65	0.65
LYPLA1	PLA2G6	9606.ENSP00000320043	9606.ENSP00000333142	0	0	0.65	0.65
LYPLA1	PLA2G4A	9606.ENSP00000320043	9606.ENSP00000356436	0	0	0.65	0.65
MAOA	PNMT	9606.ENSP00000340684	9606.ENSP00000269582	0	0	0.9	0.9
MAOA	MAOB	9606.ENSP00000340684	9606.ENSP00000367309	0.18	0.307	0.8	0.876
MAOB	PNMT	9606.ENSP00000367309	9606.ENSP00000269582	0.06	0	0.9	0.901
MAP2K1	MAPK1	9606.ENSP00000302486	9606.ENSP00000215832	0.066	0.974	0.9	0.997
MAP2K1	NFKB1	9606.ENSP00000302486	9606.ENSP00000226574	0.042	0.057	0.65	0.656
MAP2K1	MAPK14	9606.ENSP00000302486	9606.ENSP00000229795	0.066	0.603	0.9	0.959
MAP2K1	RAF1	9606.ENSP00000302486	9606.ENSP00000251849	0.083	0.971	0.9	0.997
MAP2K1	MAPK3	9606.ENSP00000302486	9606.ENSP00000263025	0.066	0.963	0.9	0.996
MAP2K1	PIK3CA	9606.ENSP00000302486	9606.ENSP00000263967	0.062	0.152	0.9	0.913
MAP2K1	STAT3	9606.ENSP00000302486	9606.ENSP00000264657	0	0	0.9	0.9
MAP2K1	PPARG	9606.ENSP00000302486	9606.ENSP00000287820	0	0.501	0	0.501
MAP2K1	SHH	9606.ENSP00000302486	9606.ENSP00000297261	0	0.132	0.9	0.909
MAP2K1	MAPK10	9606.ENSP00000302486	9606.ENSP00000352157	0.063	0	0.8	0.804
MAP2K1	MAPK8	9606.ENSP00000302486	9606.ENSP00000378974	0.063	0	0.8	0.804

MAP2K1	NCOR2	9606.ENSP00000302486	9606.ENSP00000384018	0	0	0.9	0.9
MAP2K1	TYK2	9606.ENSP00000302486	9606.ENSP00000431885	0	0.178	0.9	0.914
MAP2K1	PLA2G4A	9606.ENSP00000302486	9606.ENSP00000356436	0	0	0.9	0.9
MAP2K1	PRKCD	9606.ENSP00000302486	9606.ENSP00000378217	0.064	0.21	0.9	0.919
MAP2K1	YWHAG	9606.ENSP00000302486	9606.ENSP00000306330	0.088	0.233	0.9	0.923
MAP2K1	PIK3R1	9606.ENSP00000302486	9606.ENSP00000428056	0	0	0.9	0.9
MAP2K1	PIK3CG	9606.ENSP00000302486	9606.ENSP00000352121	0.062	0.067	0.9	0.904
MAP2K1	SRC	9606.ENSP00000302486	9606.ENSP00000362680	0.062	0.317	0.9	0.93
MAP2K1	RAC1	9606.ENSP00000302486	9606.ENSP00000348461	0.062	0.284	0.9	0.927
MAP2K1	PTPN11	9606.ENSP00000302486	9606.ENSP00000340944	0.065	0.429	0.9	0.942
MAP3K11	RAF1	9606.ENSP00000309597	9606.ENSP00000251849	0	0	0.9	0.9
MAP3K11	MAPK8	9606.ENSP00000309597	9606.ENSP00000378974	0.062	0.347	0.9	0.933
MAP3K11	RAC1	9606.ENSP00000309597	9606.ENSP00000348461	0	0.366	0.9	0.933
MAPK1	TNFRSF1A	9606.ENSP00000215832	9606.ENSP00000162749	0.051	0.502	0	0.507
MAPK1	SREBF2	9606.ENSP00000215832	9606.ENSP00000354476	0.052	0.503	0	0.509
MAPK1	MAPK10	9606.ENSP00000215832	9606.ENSP00000352157	0.081	0.056	0.5	0.528
MAPK1	PTPN1	9606.ENSP00000215832	9606.ENSP00000360683	0.092	0.382	0	0.415
MAPK1	PTPRF	9606.ENSP00000215832	9606.ENSP00000353030	0.059	0.555	0	0.563
MAPK1	PLA2G2C	9606.ENSP00000215832	9606.ENSP00000247992	0	0	0.6	0.6
MAPK1	PLA2G5	9606.ENSP00000215832	9606.ENSP00000364249	0	0	0.6	0.6
MAPK1	PLA2G6	9606.ENSP00000215832	9606.ENSP00000333142	0	0	0.6	0.6
MAPK1	PLA2G10	9606.ENSP00000215832	9606.ENSP00000393847	0	0	0.6	0.6
MAPK1	PLA2G2A	9606.ENSP00000215832	9606.ENSP00000383364	0	0	0.6	0.6
MAPK1	PLA2G4B	9606.ENSP00000215832	9606.ENSP00000396045	0	0.057	0.6	0.606
MAPK1	RET	9606.ENSP00000215832	9606.ENSP00000347942	0.049	0.616	0	0.619
MAPK1	MCL1	9606.ENSP00000215832	9606.ENSP00000358022	0	0.463	0	0.463
MAPK1	PPARG	9606.ENSP00000215832	9606.ENSP00000287820	0	0.699	0	0.699
MAPK1	SPHK1	9606.ENSP00000215832	9606.ENSP00000313681	0.049	0.057	0.8	0.804
MAPK1	SPHK2	9606.ENSP00000215832	9606.ENSP00000245222	0.062	0.057	0.8	0.807
MAPK1	NOS1	9606.ENSP00000215832	9606.ENSP00000477999	0.049	0	0.8	0.801
MAPK1	THRB	9606.ENSP00000215832	9606.ENSP00000379904	0	0.104	0.8	0.813
MAPK1	ROCK2	9606.ENSP00000215832	9606.ENSP00000317985	0.062	0.148	0.8	0.826
MAPK1	ROCK1	9606.ENSP00000215832	9606.ENSP00000382697	0.062	0.148	0.8	0.826
MAPK1	PRKCG	9606.ENSP00000215832	9606.ENSP00000263431	0.053	0.213	0.8	0.837
MAPK1	PTPN6	9606.ENSP00000215832	9606.ENSP00000391592	0.059	0.185	0.8	0.833
MAPK1	NR4A1	9606.ENSP00000215832	9606.ENSP00000440864	0	0.241	0.8	0.841
MAPK1	S1PR4	9606.ENSP00000215832	9606.ENSP00000246115	0	0	0.9	0.9
MAPK1	S1PR3	9606.ENSP00000215832	9606.ENSP00000365006	0	0	0.9	0.9
MAPK1	S1PR2	9606.ENSP00000215832	9606.ENSP00000466933	0	0	0.9	0.9
MAPK1	S1PR1	9606.ENSP00000215832	9606.ENSP00000305416	0	0	0.9	0.9
MAPK1	MAPT	9606.ENSP00000215832	9606.ENSP00000340820	0	0.362	0.8	0.867
MAPK1	RXRG	9606.ENSP00000215832	9606.ENSP00000352900	0	0.104	0.9	0.906
MAPK1	RXRB	9606.ENSP00000215832	9606.ENSP00000363817	0	0.104	0.9	0.906
MAPK1	RARG	9606.ENSP00000215832	9606.ENSP00000388510	0	0.104	0.9	0.906

MAPK1	RARB	9606.ENSP00000215832	9606.ENSP00000332296	0	0.104	0.9	0.906
MAPK1	PLA2G1B	9606.ENSP00000215832	9606.ENSP00000312286	0	0	0.9	0.9
MAPK1	PIN1	9606.ENSP00000215832	9606.ENSP00000247970	0.055	0	0.9	0.901
MAPK1	NCOR2	9606.ENSP00000215832	9606.ENSP00000384018	0.053	0	0.9	0.901
MAPK1	TYK2	9606.ENSP00000215832	9606.ENSP00000431885	0	0.148	0.9	0.911
MAPK1	YES1	9606.ENSP00000215832	9606.ENSP00000462468	0.062	0.148	0.9	0.913
MAPK1	TERT	9606.ENSP00000215832	9606.ENSP00000309572	0.057	0	0.9	0.901
MAPK1	MET	9606.ENSP00000215832	9606.ENSP00000317272	0.062	0.148	0.9	0.913
MAPK1	NTRK1	9606.ENSP00000215832	9606.ENSP00000431418	0	0.167	0.9	0.913
MAPK1	PDPK1	9606.ENSP00000215832	9606.ENSP00000344220	0.064	0.068	0.9	0.905
MAPK1	PRKCQ	9606.ENSP00000215832	9606.ENSP00000263125	0.062	0.249	0.9	0.923
MAPK1	PTK2	9606.ENSP00000215832	9606.ENSP00000341189	0.063	0.139	0.9	0.912
MAPK1	PRKCH	9606.ENSP00000215832	9606.ENSP00000329127	0.062	0.249	0.9	0.923
MAPK1	MAPK8	9606.ENSP00000215832	9606.ENSP00000378974	0.081	0.225	0.9	0.922
MAPK1	PRKCB	9606.ENSP00000215832	9606.ENSP00000305355	0.053	0.213	0.9	0.918
MAPK1	PRKCZ	9606.ENSP00000215832	9606.ENSP00000367830	0	0.3	0.9	0.927
MAPK1	MAPKAPK2	9606.ENSP00000215832	9606.ENSP00000356070	0.064	0.908	0	0.91
MAPK1	RPS6KA5	9606.ENSP00000215832	9606.ENSP00000479667	0.062	0.319	0.9	0.93
MAPK1	YWHAG	9606.ENSP00000215832	9606.ENSP00000306330	0.132	0.179	0.9	0.922
MAPK1	PRKCA	9606.ENSP00000215832	9606.ENSP00000408695	0.053	0.275	0.9	0.925
MAPK1	PPARA	9606.ENSP00000215832	9606.ENSP00000385523	0	0.264	0.9	0.923
MAPK1	NR3C1	9606.ENSP00000215832	9606.ENSP00000231509	0	0.264	0.9	0.923
MAPK1	RELA	9606.ENSP00000215832	9606.ENSP00000384273	0	0.118	0.9	0.908
MAPK1	PGR	9606.ENSP00000215832	9606.ENSP00000325120	0	0.317	0.9	0.928
MAPK1	NFKB1	9606.ENSP00000215832	9606.ENSP00000226574	0.042	0.134	0.9	0.909
MAPK1	RARA	9606.ENSP00000215832	9606.ENSP00000254066	0	0.436	0.9	0.941
MAPK1	PRKCD	9606.ENSP00000215832	9606.ENSP00000378217	0.062	0.383	0.9	0.937
MAPK1	RAC1	9606.ENSP00000215832	9606.ENSP00000348461	0.062	0.247	0.9	0.923
MAPK1	PRKCE	9606.ENSP00000215832	9606.ENSP00000306124	0.062	0.428	0.9	0.941
MAPK1	RPS6KB1	9606.ENSP00000215832	9606.ENSP00000225577	0.062	0.309	0.9	0.929
MAPK1	RXRA	9606.ENSP00000215832	9606.ENSP00000419692	0	0.498	0.9	0.947
MAPK1	SRC	9606.ENSP00000215832	9606.ENSP00000362680	0.062	0.351	0.9	0.933
MAPK1	PLA2G4A	9606.ENSP00000215832	9606.ENSP00000356436	0	0.487	0.9	0.946
MAPK1	PTPN11	9606.ENSP00000215832	9606.ENSP00000340944	0.099	0.349	0.9	0.936
MAPK1	MAPK14	9606.ENSP00000215832	9606.ENSP00000229795	0.106	0.874	0.8	0.975
MAPK1	MAPK3	9606.ENSP00000215832	9606.ENSP00000263025	0	0.887	0.9	0.988
MAPK1	RAF1	9606.ENSP00000215832	9606.ENSP00000251849	0.066	0.846	0.9	0.984
MAPK1	MKNK2	9606.ENSP00000215832	9606.ENSP00000250896	0.056	0.884	0.9	0.988
MAPK1	RPS6KA3	9606.ENSP00000215832	9606.ENSP00000368884	0.062	0.899	0.9	0.989
MAPK1	STAT3	9606.ENSP00000215832	9606.ENSP00000264657	0.062	0.888	0.9	0.988
MAPK1	PTPN7	9606.ENSP00000215832	9606.ENSP00000309116	0.063	0.899	0.9	0.989
MAPK10	RIPK2	9606.ENSP00000352157	9606.ENSP00000220751	0	0.143	0.8	0.821
MAPK10	NFKB1	9606.ENSP00000352157	9606.ENSP00000226574	0	0.102	0.8	0.812
MAPK10	MAPK14	9606.ENSP00000352157	9606.ENSP00000229795	0.066	0.056	0.6	0.616

MAPK10	NR3C1	9606.ENSP00000352157	9606.ENSP00000231509	0.062	0	0.9	0.902
MAPK10	MAPK3	9606.ENSP00000352157	9606.ENSP00000263025	0.081	0.056	0.5	0.528
MAPK10	PRKCQ	9606.ENSP00000352157	9606.ENSP00000263125	0	0.171	0.8	0.827
MAPK10	PRKCE	9606.ENSP00000352157	9606.ENSP00000306124	0.09	0.171	0.8	0.836
MAPK10	PTPN7	9606.ENSP00000352157	9606.ENSP00000309116	0	0	0.6	0.6
MAPK10	PGR	9606.ENSP00000352157	9606.ENSP00000325120	0.08	0	0.8	0.808
MAPK10	RAC1	9606.ENSP00000352157	9606.ENSP00000348461	0.064	0.243	0.9	0.922
MAPK10	PRKCD	9606.ENSP00000352157	9606.ENSP00000378217	0	0.171	0.8	0.827
MAPK10	RELA	9606.ENSP00000352157	9606.ENSP00000384273	0	0.111	0.8	0.814
MAPK10	SRC	9606.ENSP00000352157	9606.ENSP00000362680	0.063	0.143	0.8	0.825
MAPK10	MAPK8	9606.ENSP00000352157	9606.ENSP00000378974	0.062	0	0.9	0.902
MAPK10	PRKCZ	9606.ENSP00000352157	9606.ENSP00000367830	0.103	0.101	0.9	0.912
MAPK14	RIPK2	9606.ENSP00000229795	9606.ENSP00000220751	0	0.148	0.65	0.689
MAPK14	NFKB1	9606.ENSP00000229795	9606.ENSP00000226574	0.065	0.134	0.9	0.911
MAPK14	MAPK8	9606.ENSP00000229795	9606.ENSP00000378974	0.066	0.47	0.6	0.784
MAPK14	PGR	9606.ENSP00000229795	9606.ENSP00000325120	0	0.104	0.8	0.813
MAPK14	PTK2	9606.ENSP00000229795	9606.ENSP00000341189	0.065	0.1	0.8	0.817
MAPK14	NCOR2	9606.ENSP00000229795	9606.ENSP00000384018	0	0	0.9	0.9
MAPK14	RARB	9606.ENSP00000229795	9606.ENSP00000332296	0	0.104	0.9	0.906
MAPK14	S1PR2	9606.ENSP00000229795	9606.ENSP00000466933	0	0	0.9	0.9
MAPK14	RARG	9606.ENSP00000229795	9606.ENSP00000388510	0	0.104	0.9	0.906
MAPK14	MKNK2	9606.ENSP00000229795	9606.ENSP00000250896	0.062	0.89	0	0.892
MAPK14	RARA	9606.ENSP00000229795	9606.ENSP00000254066	0	0.104	0.9	0.906
MAPK14	PLA2G1B	9606.ENSP00000229795	9606.ENSP00000312286	0	0	0.9	0.9
MAPK14	YES1	9606.ENSP00000229795	9606.ENSP00000462468	0.048	0.148	0.9	0.911
MAPK14	PTK2B	9606.ENSP00000229795	9606.ENSP00000380638	0.065	0.1	0.9	0.908
MAPK14	SYK	9606.ENSP00000229795	9606.ENSP00000364907	0.062	0.148	0.9	0.913
MAPK14	NR3C1	9606.ENSP00000229795	9606.ENSP00000231509	0	0.104	0.9	0.906
MAPK14	SRC	9606.ENSP00000229795	9606.ENSP00000362680	0	0.148	0.9	0.911
MAPK14	RAC1	9606.ENSP00000229795	9606.ENSP00000348461	0.062	0.247	0.9	0.923
MAPK14	PLA2G4A	9606.ENSP00000229795	9606.ENSP00000356436	0	0.213	0.9	0.917
MAPK14	RELA	9606.ENSP00000229795	9606.ENSP00000384273	0	0.128	0.9	0.909
MAPK14	MAPK3	9606.ENSP00000229795	9606.ENSP00000263025	0.048	0.777	0.8	0.953
MAPK14	STAT3	9606.ENSP00000229795	9606.ENSP00000264657	0.062	0.056	0.9	0.903
MAPK14	TNF	9606.ENSP00000229795	9606.ENSP00000398698	0	0	0.9	0.9
MAPK14	PTPN7	9606.ENSP00000229795	9606.ENSP00000309116	0.059	0.791	0.6	0.914
MAPK14	RPS6KA5	9606.ENSP00000229795	9606.ENSP00000479667	0.062	0.777	0.9	0.977
MAPK14	MAPKAPK2	9606.ENSP00000229795	9606.ENSP00000356070	0.11	0.991	0.9	0.999
MAPK3	RPS6KB1	9606.ENSP00000263025	9606.ENSP00000225577	0.062	0.309	0.9	0.929
MAPK3	NFKB1	9606.ENSP00000263025	9606.ENSP00000226574	0	0.134	0.9	0.909
MAPK3	NR3C1	9606.ENSP00000263025	9606.ENSP00000231509	0	0.104	0.9	0.906
MAPK3	SPHK2	9606.ENSP00000263025	9606.ENSP00000245222	0.063	0.057	0.8	0.807
MAPK3	S1PR4	9606.ENSP00000263025	9606.ENSP00000246115	0	0	0.9	0.9
MAPK3	PIN1	9606.ENSP00000263025	9606.ENSP00000247970	0.049	0	0.9	0.9

MAPK3	PLA2G2C	9606.ENSP00000263025	9606.ENSP00000247992	0	0	0.6	0.6
MAPK3	MKNK2	9606.ENSP00000263025	9606.ENSP00000250896	0.056	0.176	0.9	0.915
MAPK3	RAF1	9606.ENSP00000263025	9606.ENSP00000251849	0.066	0.798	0.9	0.979
MAPK3	RARA	9606.ENSP00000263025	9606.ENSP00000254066	0.062	0.104	0.9	0.908
MAPK3	SREBF2	9606.ENSP00000263025	9606.ENSP00000354476	0.062	0.503	0	0.514
MAPK3	PLA2G4B	9606.ENSP00000263025	9606.ENSP00000396045	0	0.057	0.6	0.606
MAPK3	PLA2G5	9606.ENSP00000263025	9606.ENSP00000364249	0	0	0.6	0.6
MAPK3	PLA2G6	9606.ENSP00000263025	9606.ENSP00000333142	0.059	0	0.6	0.607
MAPK3	PLA2G10	9606.ENSP00000263025	9606.ENSP00000393847	0	0	0.6	0.6
MAPK3	PLA2G2A	9606.ENSP00000263025	9606.ENSP00000383364	0	0	0.6	0.6
MAPK3	RET	9606.ENSP00000263025	9606.ENSP00000347942	0.049	0.616	0	0.619
MAPK3	MAPKAPK2	9606.ENSP00000263025	9606.ENSP00000356070	0.064	0.655	0	0.663
MAPK3	THRB	9606.ENSP00000263025	9606.ENSP00000379904	0	0.104	0.8	0.813
MAPK3	ROCK2	9606.ENSP00000263025	9606.ENSP00000317985	0.062	0.148	0.8	0.826
MAPK3	PRKCG	9606.ENSP00000263025	9606.ENSP00000263431	0.053	0.213	0.8	0.837
MAPK3	ROCK1	9606.ENSP00000263025	9606.ENSP00000382697	0.062	0.148	0.8	0.826
MAPK3	NOS1	9606.ENSP00000263025	9606.ENSP00000477999	0.049	0	0.8	0.801
MAPK3	NR4A1	9606.ENSP00000263025	9606.ENSP00000440864	0	0.076	0.8	0.807
MAPK3	PTPN6	9606.ENSP00000263025	9606.ENSP00000391592	0.059	0.185	0.8	0.833
MAPK3	PRKCZ	9606.ENSP00000263025	9606.ENSP00000367830	0	0.3	0.8	0.854
MAPK3	RXRB	9606.ENSP00000263025	9606.ENSP00000363817	0	0.104	0.9	0.906
MAPK3	RXRG	9606.ENSP00000263025	9606.ENSP00000352900	0	0.104	0.9	0.906
MAPK3	RARG	9606.ENSP00000263025	9606.ENSP00000388510	0	0.104	0.9	0.906
MAPK3	SPHK1	9606.ENSP00000263025	9606.ENSP00000313681	0.049	0.057	0.8	0.804
MAPK3	NCOR2	9606.ENSP00000263025	9606.ENSP00000384018	0.053	0	0.9	0.901
MAPK3	RARB	9606.ENSP00000263025	9606.ENSP00000332296	0	0.104	0.9	0.906
MAPK3	TYK2	9606.ENSP00000263025	9606.ENSP00000431885	0	0.148	0.9	0.911
MAPK3	PRKCH	9606.ENSP00000263025	9606.ENSP00000329127	0.074	0.249	0.9	0.924
MAPK3	PLA2G1B	9606.ENSP00000263025	9606.ENSP00000312286	0	0	0.9	0.9
MAPK3	YWHAG	9606.ENSP00000263025	9606.ENSP00000306330	0.073	0.179	0.9	0.917
MAPK3	PRKCQ	9606.ENSP00000263025	9606.ENSP00000263125	0.062	0.249	0.9	0.923
MAPK3	RPS6KA5	9606.ENSP00000263025	9606.ENSP00000479667	0.062	0.175	0.9	0.915
MAPK3	TERT	9606.ENSP00000263025	9606.ENSP00000309572	0.057	0	0.9	0.901
MAPK3	PRKCB	9606.ENSP00000263025	9606.ENSP00000305355	0.053	0.213	0.9	0.918
MAPK3	MAPK8	9606.ENSP00000263025	9606.ENSP00000378974	0.083	0.225	0.9	0.922
MAPK3	MAPT	9606.ENSP00000263025	9606.ENSP00000340820	0	0.517	0.8	0.899
MAPK3	MET	9606.ENSP00000263025	9606.ENSP00000317272	0.062	0.148	0.9	0.913
MAPK3	S1PR1	9606.ENSP00000263025	9606.ENSP00000305416	0	0	0.9	0.9
MAPK3	RXRA	9606.ENSP00000263025	9606.ENSP00000419692	0	0.317	0.9	0.928
MAPK3	S1PR3	9606.ENSP00000263025	9606.ENSP00000365006	0	0	0.9	0.9
MAPK3	PTK2	9606.ENSP00000263025	9606.ENSP00000341189	0.063	0.139	0.9	0.912
MAPK3	NTRK1	9606.ENSP00000263025	9606.ENSP00000431418	0	0.3	0.9	0.927
MAPK3	RELA	9606.ENSP00000263025	9606.ENSP00000384273	0	0	0.9	0.9
MAPK3	PRKCE	9606.ENSP00000263025	9606.ENSP00000306124	0.062	0.383	0.9	0.937

MAPK3	S1PR2	9606.ENSP00000263025	9606.ENSP00000466933	0.077	0	0.9	0.903
MAPK3	PRKCA	9606.ENSP00000263025	9606.ENSP00000408695	0.053	0.369	0.9	0.935
MAPK3	PRKCD	9606.ENSP00000263025	9606.ENSP00000378217	0.062	0.383	0.9	0.937
MAPK3	RAC1	9606.ENSP00000263025	9606.ENSP00000348461	0.062	0.247	0.9	0.923
MAPK3	PLA2G4A	9606.ENSP00000263025	9606.ENSP00000356436	0	0.27	0.9	0.923
MAPK3	PGR	9606.ENSP00000263025	9606.ENSP00000325120	0	0.104	0.9	0.906
MAPK3	PPARA	9606.ENSP00000263025	9606.ENSP00000385523	0	0.264	0.9	0.923
MAPK3	SRC	9606.ENSP00000263025	9606.ENSP00000362680	0.062	0.538	0.9	0.952
MAPK3	STAT3	9606.ENSP00000263025	9606.ENSP00000264657	0.062	0.225	0.9	0.92
MAPK3	PTPN7	9606.ENSP00000263025	9606.ENSP00000309116	0.059	0.803	0.9	0.979
MAPK3	RPS6KA3	9606.ENSP00000263025	9606.ENSP00000368884	0.066	0.86	0.9	0.985
MAPK3	PTPN11	9606.ENSP00000263025	9606.ENSP00000340944	0.067	0.784	0.9	0.978
MAPK8	RIPK2	9606.ENSP00000378974	9606.ENSP00000220751	0.049	0.143	0.8	0.822
MAPK8	RPS6KB1	9606.ENSP00000378974	9606.ENSP00000225577	0.078	0.492	0	0.511
MAPK8	NFKB1	9606.ENSP00000378974	9606.ENSP00000226574	0.068	0.102	0.8	0.817
MAPK8	NR3C1	9606.ENSP00000378974	9606.ENSP00000231509	0.065	0.213	0.9	0.92
MAPK8	RARA	9606.ENSP00000378974	9606.ENSP00000254066	0	0	0.9	0.9
MAPK8	PRKCQ	9606.ENSP00000378974	9606.ENSP00000263125	0	0.171	0.9	0.913
MAPK8	STAT3	9606.ENSP00000378974	9606.ENSP00000264657	0	0.678	0	0.678
MAPK8	PRKCB	9606.ENSP00000378974	9606.ENSP00000305355	0.049	0	0.9	0.9
MAPK8	PRKCE	9606.ENSP00000378974	9606.ENSP00000306124	0.062	0.171	0.8	0.83
MAPK8	PTPN7	9606.ENSP00000378974	9606.ENSP00000309116	0	0	0.6	0.6
MAPK8	MET	9606.ENSP00000378974	9606.ENSP00000317272	0.062	0.101	0.9	0.908
MAPK8	PGR	9606.ENSP00000378974	9606.ENSP00000325120	0.062	0	0.8	0.804
MAPK8	MAPT	9606.ENSP00000378974	9606.ENSP00000340820	0.065	0.488	0.9	0.947
MAPK8	RET	9606.ENSP00000378974	9606.ENSP00000347942	0.062	0.297	0.9	0.928
MAPK8	RAC1	9606.ENSP00000378974	9606.ENSP00000348461	0.064	0.423	0.9	0.941
MAPK8	SRC	9606.ENSP00000378974	9606.ENSP00000362680	0.063	0.143	0.9	0.912
MAPK8	PRKCZ	9606.ENSP00000378974	9606.ENSP00000367830	0.078	0.101	0.9	0.909
MAPK8	PRKCD	9606.ENSP00000378974	9606.ENSP00000378217	0	0.171	0.8	0.827
MAPK8	NR4A1	9606.ENSP00000378974	9606.ENSP00000440864	0	0.488	0	0.487
MAPK8	NFE2L2	9606.ENSP00000378974	9606.ENSP00000380252	0	0.494	0	0.494
MAPK8	PIK3R1	9606.ENSP00000378974	9606.ENSP00000428056	0	0.678	0	0.678
MAPK8	RXRA	9606.ENSP00000378974	9606.ENSP00000419692	0	0	0.9	0.9
MAPK8	RELA	9606.ENSP00000378974	9606.ENSP00000384273	0	0	0.8	0.8
MAPK8	S1PR2	9606.ENSP00000378974	9606.ENSP00000466933	0	0	0.9	0.9
MAPK8	PTK2B	9606.ENSP00000378974	9606.ENSP00000380638	0.085	0	0.9	0.904
MAPKAPK2	SRC	9606.ENSP00000356070	9606.ENSP00000362680	0.111	0.059	0.65	0.681
MAPKAPK2	TNF	9606.ENSP00000356070	9606.ENSP00000398698	0.062	0	0.9	0.902
MAPT	NDUFB4	9606.ENSP00000340820	9606.ENSP00000184266	0	0.182	0.6	0.658
MAPT	NDUFB7	9606.ENSP00000340820	9606.ENSP00000215565	0	0	0.6	0.6
MAPT	TUBB1	9606.ENSP00000340820	9606.ENSP00000217133	0	0.075	0.8	0.807
MAPT	RPS6KB1	9606.ENSP00000340820	9606.ENSP00000225577	0	0.487	0	0.487
MAPT	NDUFS7	9606.ENSP00000340820	9606.ENSP00000233627	0	0.182	0.6	0.658

MAPT	NDUFB3	9606.ENSP00000340820	9606.ENSP00000237889	0	0	0.6	0.6
MAPT	PIN1	9606.ENSP00000340820	9606.ENSP00000247970	0	0.686	0	0.686
MAPT	NDUFA2	9606.ENSP00000340820	9606.ENSP00000252102	0	0	0.6	0.6
MAPT	NDUFA10	9606.ENSP00000340820	9606.ENSP00000252711	0	0	0.6	0.6
MAPT	NDUFB5	9606.ENSP00000340820	9606.ENSP00000259037	0	0	0.6	0.6
MAPT	PRKCG	9606.ENSP00000340820	9606.ENSP00000263431	0.14	0.345	0	0.412
MAPT	NDUFS3	9606.ENSP00000340820	9606.ENSP00000263774	0	0	0.6	0.6
MAPT	NDUFA9	9606.ENSP00000340820	9606.ENSP00000266544	0	0.176	0.6	0.656
MAPT	NDUFB10	9606.ENSP00000340820	9606.ENSP00000268668	0	0	0.6	0.6
MAPT	NDUFS6	9606.ENSP00000340820	9606.ENSP00000274137	0	0	0.6	0.6
MAPT	NDUFB9	9606.ENSP00000340820	9606.ENSP00000276689	0	0	0.6	0.6
MAPT	NDUFC2	9606.ENSP00000340820	9606.ENSP00000281031	0	0	0.6	0.6
MAPT	NDUFS4	9606.ENSP00000340820	9606.ENSP00000296684	0	0	0.6	0.6
MAPT	NDUFB8	9606.ENSP00000340820	9606.ENSP00000299166	0	0	0.6	0.6
MAPT	NDUFA7	9606.ENSP00000340820	9606.ENSP00000301457	0	0.182	0.6	0.658
MAPT	YWHAG	9606.ENSP00000340820	9606.ENSP00000306330	0.062	0.68	0	0.687
MAPT	NDUFS8	9606.ENSP00000340820	9606.ENSP00000315774	0	0	0.6	0.6
MAPT	NDUFV1	9606.ENSP00000340820	9606.ENSP00000322450	0	0.182	0.6	0.658
MAPT	NDUFV2	9606.ENSP00000340820	9606.ENSP00000327268	0	0	0.6	0.6
MAPT	NDUFA12	9606.ENSP00000340820	9606.ENSP00000330737	0	0.172	0.6	0.654
MAPT	NDUFB1	9606.ENSP00000340820	9606.ENSP00000330787	0	0	0.6	0.6
MAPT	NDUFA4	9606.ENSP00000340820	9606.ENSP00000339720	0	0	0.6	0.6
MAPT	RPS6KA3	9606.ENSP00000340820	9606.ENSP00000368884	0	0.463	0	0.463
MAPT	PIK3R1	9606.ENSP00000340820	9606.ENSP00000428056	0	0.486	0	0.485
MAPT	NDUFB2	9606.ENSP00000340820	9606.ENSP00000419087	0	0	0.6	0.6
MAPT	NDUFV3	9606.ENSP00000340820	9606.ENSP00000346196	0	0	0.6	0.6
MAPT	NDUFS1	9606.ENSP00000340820	9606.ENSP00000392709	0	0	0.6	0.6
MAPT	NDUFA11	9606.ENSP00000340820	9606.ENSP00000389160	0	0	0.6	0.6
MAPT	NDUFA8	9606.ENSP00000340820	9606.ENSP00000362873	0	0	0.6	0.6
MAPT	MT-ND4	9606.ENSP00000340820	9606.ENSP00000354961	0	0	0.6	0.6
MAPT	MT-ND4L	9606.ENSP00000340820	9606.ENSP00000354728	0	0	0.6	0.6
MAPT	NDUFA13	9606.ENSP00000340820	9606.ENSP00000423673	0	0	0.6	0.6
MAPT	NDUFS5	9606.ENSP00000340820	9606.ENSP00000362060	0	0	0.6	0.6
MAPT	NDUFC1	9606.ENSP00000340820	9606.ENSP00000441126	0	0	0.6	0.6
MAPT	MT-ND5	9606.ENSP00000340820	9606.ENSP00000354813	0	0	0.6	0.6
MAPT	MT-ND1	9606.ENSP00000340820	9606.ENSP00000354687	0	0	0.6	0.6
MAPT	NDUFS2	9606.ENSP00000340820	9606.ENSP00000356972	0	0	0.6	0.6
MAPT	NDUFB6	9606.ENSP00000340820	9606.ENSP00000369176	0	0	0.6	0.6
MAPT	NDUFAB1	9606.ENSP00000340820	9606.ENSP00000458770	0	0	0.6	0.6
MAPT	NDUFA6	9606.ENSP00000340820	9606.ENSP00000418842	0	0	0.6	0.6
MAPT	MT-ND3	9606.ENSP00000340820	9606.ENSP00000355206	0	0	0.6	0.6
MAPT	NDUFA3	9606.ENSP00000340820	9606.ENSP00000418438	0	0	0.6	0.6
MAPT	NDUFA4L2	9606.ENSP00000340820	9606.ENSP00000377411	0.062	0	0.6	0.608
MAPT	NDUFA5	9606.ENSP00000340820	9606.ENSP00000417142	0	0	0.6	0.6

MAPT	MT-ND6	9606.ENSP00000340820	9606.ENSP00000354665	0	0	0.6	0.6
MAPT	NDUFA1	9606.ENSP00000340820	9606.ENSP00000360492	0	0	0.6	0.6
MAPT	MT-ND2	9606.ENSP00000340820	9606.ENSP00000355046	0	0	0.6	0.6
MAPT	SRC	9606.ENSP00000340820	9606.ENSP00000362680	0	0.773	0	0.773
MCL1	STAT3	9606.ENSP00000358022	9606.ENSP00000264657	0.065	0.069	0.9	0.905
MCL1	NOS2	9606.ENSP00000358022	9606.ENSP00000327251	0	0	0.9	0.9
MCL1	PIK3R1	9606.ENSP00000358022	9606.ENSP00000428056	0	0	0.9	0.9
MDH1	NDUFB5	9606.ENSP00000438144	9606.ENSP00000259037	0.838	0.156	0	0.857
MDH1	NDUFS4	9606.ENSP00000438144	9606.ENSP00000296684	0.445	0	0	0.445
MDH1	NDUFA12	9606.ENSP00000438144	9606.ENSP00000330737	0.667	0	0	0.667
MDH1	NDUFA4	9606.ENSP00000438144	9606.ENSP00000339720	0.337	0.15	0	0.413
MDH1	NDUFA8	9606.ENSP00000438144	9606.ENSP00000362873	0.37	0.153	0	0.444
MDH1	NDUFAB1	9606.ENSP00000438144	9606.ENSP00000458770	0.313	0.256	0	0.467
MDM2	PER2	9606.ENSP00000258149	9606.ENSP00000254657	0	0.486	0	0.485
MDM2	PDGFRA	9606.ENSP00000258149	9606.ENSP00000257290	0	0	0.6	0.6
MDM2	PPARD	9606.ENSP00000258149	9606.ENSP00000310928	0	0.427	0	0.427
MDM2	PCNA	9606.ENSP00000258149	9606.ENSP00000368458	0	0.486	0	0.485
MDM2	PIM1	9606.ENSP00000258149	9606.ENSP00000362608	0	0.487	0	0.487
MDM2	PDPK1	9606.ENSP00000258149	9606.ENSP00000344220	0.062	0	0.65	0.657
MDM2	TERT	9606.ENSP00000258149	9606.ENSP00000309572	0	0.486	0	0.485
MDM2	SRC	9606.ENSP00000258149	9606.ENSP00000362680	0	0.281	0.65	0.737
MDM2	PRKDC	9606.ENSP00000258149	9606.ENSP00000313420	0	0	0.9	0.9
MELK	PCNA	9606.ENSP00000298048	9606.ENSP00000368458	0.663	0	0	0.663
MELK	TYMS	9606.ENSP00000298048	9606.ENSP00000315644	0.821	0	0	0.821
MELK	PLK1	9606.ENSP00000298048	9606.ENSP00000300093	0.839	0.074	0	0.844
MELK	TTK	9606.ENSP00000298048	9606.ENSP00000358813	0.914	0.056	0	0.915
MELK	TOP2A	9606.ENSP00000298048	9606.ENSP00000411532	0.935	0.056	0	0.936
MET	PIK3CA	9606.ENSP00000317272	9606.ENSP00000263967	0.063	0.104	0.9	0.908
MET	STAT3	9606.ENSP00000317272	9606.ENSP00000264657	0.063	0.345	0.6	0.733
MET	PIK3CB	9606.ENSP00000317272	9606.ENSP00000289153	0.063	0.104	0.8	0.817
MET	PTPN2	9606.ENSP00000317272	9606.ENSP00000311857	0.062	0.142	0.9	0.912
MET	RAC1	9606.ENSP00000317272	9606.ENSP00000348461	0.062	0.243	0.5	0.613
MET	PIK3CD	9606.ENSP00000317272	9606.ENSP00000366563	0.063	0.104	0.8	0.817
MET	PTK2	9606.ENSP00000317272	9606.ENSP00000341189	0.075	0.078	0.8	0.814
MET	PIK3R1	9606.ENSP00000317272	9606.ENSP00000428056	0	0.324	0.9	0.929
MET	PTPN1	9606.ENSP00000317272	9606.ENSP00000360683	0.062	0.731	0.9	0.972
MET	SRC	9606.ENSP00000317272	9606.ENSP00000362680	0.064	0.756	0.9	0.975
MET	PTPN11	9606.ENSP00000317272	9606.ENSP00000340944	0.062	0.701	0.9	0.969
METAP2	PAOX	9606.ENSP00000325312	9606.ENSP00000278060	0.482	0	0	0.482
MME	PRCP	9606.ENSP00000418525	9606.ENSP00000377055	0.05	0	0.8	0.801
MMP1	MMP7	9606.ENSP00000322788	9606.ENSP00000260227	0.062	0.213	0.9	0.919
MMP1	MMP13	9606.ENSP00000322788	9606.ENSP00000260302	0.078	0	0.65	0.663
MMP1	STAT3	9606.ENSP00000322788	9606.ENSP00000264657	0	0.273	0.9	0.924
MMP1	MMP3	9606.ENSP00000322788	9606.ENSP00000299855	0.529	0	0.9	0.95

MMP1	PRSS1	9606.ENSP00000322788	9606.ENSP00000308720	0.066	0	0.9	0.902
MMP1	MMP9	9606.ENSP00000322788	9606.ENSP00000361405	0.518	0	0.9	0.949
MMP12	MMP3	9606.ENSP00000458585	9606.ENSP00000299855	0.298	0	0.3	0.487
MMP12	MMP9	9606.ENSP00000458585	9606.ENSP00000361405	0.512	0	0.3	0.643
MMP13	PRSS1	9606.ENSP00000260302	9606.ENSP00000308720	0	0	0.9	0.9
MMP13	MMP16	9606.ENSP00000260302	9606.ENSP00000286614	0	0	0.9	0.9
MMP13	MMP14	9606.ENSP00000260302	9606.ENSP00000308208	0.062	0.213	0.9	0.919
MMP14	MMP16	9606.ENSP00000308208	9606.ENSP00000286614	0	0	0.9	0.9
MMP3	STAT3	9606.ENSP00000299855	9606.ENSP00000264657	0	0.27	0.9	0.923
MMP3	PRSS1	9606.ENSP00000299855	9606.ENSP00000308720	0	0	0.9	0.9
MMP3	PRKCA	9606.ENSP00000299855	9606.ENSP00000408695	0	0	0.9	0.9
MMP3	MMP9	9606.ENSP00000299855	9606.ENSP00000361405	0.518	0	0.8	0.899
MMP7	MMP9	9606.ENSP00000260227	9606.ENSP00000361405	0.12	0.213	0.3	0.473
MMP7	PRSS1	9606.ENSP00000260227	9606.ENSP00000308720	0	0	0.9	0.9
MMP8	MMP9	9606.ENSP00000236826	9606.ENSP00000361405	0.546	0	0	0.546
MMP8	PRSS1	9606.ENSP00000236826	9606.ENSP00000308720	0	0	0.9	0.9
MMP9	STAT3	9606.ENSP00000361405	9606.ENSP00000264657	0	0	0.9	0.9
MMP9	PRSS1	9606.ENSP00000361405	9606.ENSP00000308720	0	0	0.9	0.9
MMP9	SRC	9606.ENSP00000361405	9606.ENSP00000362680	0.089	0	0.9	0.905
MPEG1	TLR8	9606.ENSP00000354335	9606.ENSP00000312082	0.526	0	0	0.526
MPEG1	PTPRC	9606.ENSP00000354335	9606.ENSP00000411355	0.657	0	0	0.657
MPO	PTGS1	9606.ENSP00000225275	9606.ENSP00000354612	0.076	0	0.9	0.903
MSR1	PLA2G7	9606.ENSP00000262101	9606.ENSP00000274793	0.164	0	0.54	0.599
MT-ND1	NDUFB4	9606.ENSP00000354687	9606.ENSP00000184266	0.157	0.929	0.9	0.993
MT-ND1	NDUFB7	9606.ENSP00000354687	9606.ENSP00000215565	0.504	0.929	0.9	0.996
MT-ND1	NDUFS7	9606.ENSP00000354687	9606.ENSP00000233627	0.456	0.953	0.9	0.997
MT-ND1	NDUFB3	9606.ENSP00000354687	9606.ENSP00000237889	0.343	0.868	0.9	0.99
MT-ND1	NDUFA2	9606.ENSP00000354687	9606.ENSP00000252102	0.328	0.993	0.9	0.999
MT-ND1	NDUFA10	9606.ENSP00000354687	9606.ENSP00000252711	0.065	0.901	0.9	0.989
MT-ND1	NDUFB5	9606.ENSP00000354687	9606.ENSP00000259037	0.107	0.929	0.9	0.993
MT-ND1	NDUFAF1	9606.ENSP00000354687	9606.ENSP00000260361	0.064	0.27	0.9	0.925
MT-ND1	NDUFS3	9606.ENSP00000354687	9606.ENSP00000263774	0.691	0.998	0.9	0.999
MT-ND1	NDUFA9	9606.ENSP00000354687	9606.ENSP00000266544	0.202	0.901	0.9	0.991
MT-ND1	NDUFB10	9606.ENSP00000354687	9606.ENSP00000268668	0.18	0.901	0.9	0.991
MT-ND1	NDUFS6	9606.ENSP00000354687	9606.ENSP00000274137	0.182	0.894	0.9	0.99
MT-ND1	NDUFB11	9606.ENSP00000354687	9606.ENSP00000276062	0.105	0.862	0.9	0.986
MT-ND1	NDUFB9	9606.ENSP00000354687	9606.ENSP00000276689	0.247	0.929	0.9	0.994
MT-ND1	NDUFC2	9606.ENSP00000354687	9606.ENSP00000281031	0.329	0.901	0.9	0.992
MT-ND1	NDUFS4	9606.ENSP00000354687	9606.ENSP00000296684	0.303	0.987	0.9	0.999
MT-ND1	NDUFB8	9606.ENSP00000354687	9606.ENSP00000299166	0.302	0.901	0.9	0.992
MT-ND1	NDUFA7	9606.ENSP00000354687	9606.ENSP00000301457	0.114	0.929	0.9	0.993
MT-ND1	NDUFS8	9606.ENSP00000354687	9606.ENSP00000315774	0.704	0.938	0.9	0.998
MT-ND1	NDUFV1	9606.ENSP00000354687	9606.ENSP00000322450	0.68	0.993	0.9	0.999
MT-ND1	NDUFAF3	9606.ENSP00000354687	9606.ENSP00000323076	0.087	0	0.6	0.619

MT-ND1	NDUFV2	9606.ENSP00000354687	9606.ENSP00000327268	0.6	0.977	0.9	0.999
MT-ND1	NDUFA12	9606.ENSP00000354687	9606.ENSP00000330737	0.289	0.987	0.9	0.998
MT-ND1	NDUFB1	9606.ENSP00000354687	9606.ENSP00000330787	0.154	0.842	0.9	0.985
MT-ND1	NDUFA4	9606.ENSP00000354687	9606.ENSP00000339720	0.062	0	0.9	0.902
MT-ND1	NDUFV3	9606.ENSP00000354687	9606.ENSP00000346196	0.06	0.8	0.9	0.979
MT-ND1	MT-ND6	9606.ENSP00000354687	9606.ENSP00000354665	0.577	0.929	0.6	0.987
MT-ND1	NDUFAF4	9606.ENSP00000354687	9606.ENSP00000358272	0.045	0	0.6	0.601
MT-ND1	NDUFC1	9606.ENSP00000354687	9606.ENSP00000441126	0	0.868	0.9	0.986
MT-ND1	NDUFA13	9606.ENSP00000354687	9606.ENSP00000423673	0.422	0.844	0.9	0.99
MT-ND1	NDUFA3	9606.ENSP00000354687	9606.ENSP00000418438	0.322	0.901	0.9	0.992
MT-ND1	NDUFB2	9606.ENSP00000354687	9606.ENSP00000419087	0	0.901	0.9	0.989
MT-ND1	NDUFA5	9606.ENSP00000354687	9606.ENSP00000417142	0.23	0.915	0.9	0.992
MT-ND1	NDUFB6	9606.ENSP00000354687	9606.ENSP00000369176	0.425	0.901	0.9	0.993
MT-ND1	NDUFA11	9606.ENSP00000354687	9606.ENSP00000389160	0.422	0.788	0.9	0.986
MT-ND1	NDUFA8	9606.ENSP00000354687	9606.ENSP00000362873	0.288	0.945	0.9	0.995
MT-ND1	NDUFA1	9606.ENSP00000354687	9606.ENSP00000360492	0.078	0.868	0.9	0.986
MT-ND1	NDUFAB1	9606.ENSP00000354687	9606.ENSP00000458770	0.072	0.987	0.9	0.998
MT-ND1	NDUFS5	9606.ENSP00000354687	9606.ENSP00000362060	0.379	0.993	0.9	0.999
MT-ND1	NDUFS1	9606.ENSP00000354687	9606.ENSP00000392709	0.733	0.977	0.9	0.999
MT-ND1	MT-ND2	9606.ENSP00000354687	9606.ENSP00000355046	0.927	0.929	0.9	0.999
MT-ND1	MT-ND4L	9606.ENSP00000354687	9606.ENSP00000354728	0.753	0.929	0.54	0.991
MT-ND1	NDUFA6	9606.ENSP00000354687	9606.ENSP00000418842	0.154	0.993	0.9	0.999
MT-ND1	MT-ND4	9606.ENSP00000354687	9606.ENSP00000354961	0.985	0.943	0.9	0.999
MT-ND1	NDUFS2	9606.ENSP00000354687	9606.ENSP00000356972	0.671	0.944	0.9	0.998
MT-ND1	MT-ND5	9606.ENSP00000354687	9606.ENSP00000354813	0.947	0.929	0.9	0.999
MT-ND1	MT-ND3	9606.ENSP00000354687	9606.ENSP00000355206	0.825	0.944	0.9	0.998
MT-ND2	NDUFB4	9606.ENSP00000355046	9606.ENSP00000184266	0.082	0.929	0.9	0.992
MT-ND2	NDUFB7	9606.ENSP00000355046	9606.ENSP00000215565	0.183	0.929	0.9	0.993
MT-ND2	NDUFS7	9606.ENSP00000355046	9606.ENSP00000233627	0.226	0.929	0.9	0.994
MT-ND2	NDUFB3	9606.ENSP00000355046	9606.ENSP00000237889	0.161	0.868	0.9	0.987
MT-ND2	NDUFA2	9606.ENSP00000355046	9606.ENSP00000252102	0.181	0.929	0.9	0.993
MT-ND2	NDUFA10	9606.ENSP00000355046	9606.ENSP00000252711	0.056	0.901	0.9	0.989
MT-ND2	NDUFB5	9606.ENSP00000355046	9606.ENSP00000259037	0.069	0.929	0.9	0.992
MT-ND2	NDUFAF1	9606.ENSP00000355046	9606.ENSP00000260361	0.059	0	0.9	0.901
MT-ND2	NDUFS3	9606.ENSP00000355046	9606.ENSP00000263774	0.242	0.96	0.9	0.996
MT-ND2	NDUFA9	9606.ENSP00000355046	9606.ENSP00000266544	0.104	0.901	0.9	0.99
MT-ND2	NDUFB10	9606.ENSP00000355046	9606.ENSP00000268668	0.11	0.901	0.9	0.99
MT-ND2	NDUFS6	9606.ENSP00000355046	9606.ENSP00000274137	0.099	0.902	0.9	0.99
MT-ND2	NDUFB11	9606.ENSP00000355046	9606.ENSP00000276062	0.082	0.877	0.9	0.987
MT-ND2	NDUFB9	9606.ENSP00000355046	9606.ENSP00000276689	0.132	0.929	0.9	0.993
MT-ND2	NDUFC2	9606.ENSP00000355046	9606.ENSP00000281031	0.129	0.901	0.9	0.99
MT-ND2	NDUFS4	9606.ENSP00000355046	9606.ENSP00000296684	0.18	0.868	0.9	0.988
MT-ND2	NDUFB8	9606.ENSP00000355046	9606.ENSP00000299166	0.111	0.901	0.9	0.99
MT-ND2	NDUFA7	9606.ENSP00000355046	9606.ENSP00000301457	0.083	0.929	0.9	0.992

MT-ND2	NDUFS8	9606.ENSP00000355046	9606.ENSP00000315774	0.284	0.918	0.9	0.993
MT-ND2	NDUFV1	9606.ENSP00000355046	9606.ENSP00000322450	0.3	0.929	0.9	0.994
MT-ND2	NDUFAF3	9606.ENSP00000355046	9606.ENSP00000323076	0.124	0	0.6	0.634
MT-ND2	NDUFV2	9606.ENSP00000355046	9606.ENSP00000327268	0.221	0.929	0.9	0.994
MT-ND2	NDUFA12	9606.ENSP00000355046	9606.ENSP00000330737	0.145	0.868	0.9	0.987
MT-ND2	NDUFB1	9606.ENSP00000355046	9606.ENSP00000330787	0.086	0.842	0.9	0.984
MT-ND2	NDUFA4	9606.ENSP00000355046	9606.ENSP00000339720	0	0	0.9	0.9
MT-ND2	NDUFV3	9606.ENSP00000355046	9606.ENSP00000346196	0.046	0.8	0.9	0.979
MT-ND2	MT-ND6	9606.ENSP00000355046	9606.ENSP00000354665	0.743	0.929	0.8	0.996
MT-ND2	MT-ND4L	9606.ENSP00000355046	9606.ENSP00000354728	0.885	0.929	0.54	0.995
MT-ND2	MT-ND5	9606.ENSP00000355046	9606.ENSP00000354813	0.894	0.929	0.9	0.999
MT-ND2	MT-ND4	9606.ENSP00000355046	9606.ENSP00000354961	0.922	0.929	0.9	0.999
MT-ND2	NDUFAF4	9606.ENSP00000355046	9606.ENSP00000358272	0.069	0	0.6	0.611
MT-ND2	NDUFC1	9606.ENSP00000355046	9606.ENSP00000441126	0	0.868	0.9	0.986
MT-ND2	NDUFB2	9606.ENSP00000355046	9606.ENSP00000419087	0	0.901	0.9	0.989
MT-ND2	NDUFA13	9606.ENSP00000355046	9606.ENSP00000423673	0.171	0.86	0.9	0.987
MT-ND2	NDUFA3	9606.ENSP00000355046	9606.ENSP00000418438	0.148	0.901	0.9	0.99
MT-ND2	NDUFB6	9606.ENSP00000355046	9606.ENSP00000369176	0.183	0.901	0.9	0.991
MT-ND2	NDUFA8	9606.ENSP00000355046	9606.ENSP00000362873	0.145	0.929	0.9	0.993
MT-ND2	NDUFA11	9606.ENSP00000355046	9606.ENSP00000389160	0.114	0.901	0.9	0.99
MT-ND2	NDUFS5	9606.ENSP00000355046	9606.ENSP00000362060	0.182	0.929	0.9	0.993
MT-ND2	NDUFA5	9606.ENSP00000355046	9606.ENSP00000417142	0.172	0.916	0.9	0.992
MT-ND2	NDUFA1	9606.ENSP00000355046	9606.ENSP00000360492	0.063	0.868	0.9	0.986
MT-ND2	NDUFS1	9606.ENSP00000355046	9606.ENSP00000392709	0.497	0.929	0.9	0.996
MT-ND2	NDUFAB1	9606.ENSP00000355046	9606.ENSP00000458770	0.074	0.987	0.9	0.998
MT-ND2	NDUFS2	9606.ENSP00000355046	9606.ENSP00000356972	0.264	0.929	0.9	0.994
MT-ND2	MT-ND3	9606.ENSP00000355046	9606.ENSP00000355206	0.598	0.929	0.9	0.996
MT-ND2	NDUFA6	9606.ENSP00000355046	9606.ENSP00000418842	0.1	0.993	0.9	0.999
MT-ND3	NDUFB4	9606.ENSP00000355206	9606.ENSP00000184266	0.063	0.929	0.9	0.992
MT-ND3	NDUFB7	9606.ENSP00000355206	9606.ENSP00000215565	0.139	0.929	0.9	0.993
MT-ND3	NDUFS7	9606.ENSP00000355206	9606.ENSP00000233627	0.756	0.943	0.9	0.998
MT-ND3	NDUFB3	9606.ENSP00000355206	9606.ENSP00000237889	0.062	0.868	0.9	0.986
MT-ND3	NDUFA2	9606.ENSP00000355206	9606.ENSP00000252102	0.062	0.929	0.9	0.992
MT-ND3	NDUFA10	9606.ENSP00000355206	9606.ENSP00000252711	0.062	0.901	0.9	0.989
MT-ND3	NDUFB5	9606.ENSP00000355206	9606.ENSP00000259037	0.062	0.929	0.9	0.992
MT-ND3	NDUFAF1	9606.ENSP00000355206	9606.ENSP00000260361	0.117	0	0.9	0.907
MT-ND3	NDUFS3	9606.ENSP00000355206	9606.ENSP00000263774	0.639	0.944	0.9	0.997
MT-ND3	NDUFA9	9606.ENSP00000355206	9606.ENSP00000266544	0.076	0.987	0.9	0.998
MT-ND3	NDUFB10	9606.ENSP00000355206	9606.ENSP00000268668	0.062	0.901	0.9	0.989
MT-ND3	NDUFS6	9606.ENSP00000355206	9606.ENSP00000274137	0.055	0.868	0.9	0.986
MT-ND3	NDUFB11	9606.ENSP00000355206	9606.ENSP00000276062	0.062	0.877	0.9	0.987
MT-ND3	NDUFB9	9606.ENSP00000355206	9606.ENSP00000276689	0.111	0.929	0.9	0.993
MT-ND3	NDUFC2	9606.ENSP00000355206	9606.ENSP00000281031	0.062	0.901	0.9	0.989
MT-ND3	NDUFS4	9606.ENSP00000355206	9606.ENSP00000296684	0.062	0.868	0.9	0.986

MT-ND3	NDUFB8	9606.ENSP00000355206	9606.ENSP00000299166	0.062	0.901	0.9	0.989
MT-ND3	NDUFA7	9606.ENSP00000355206	9606.ENSP00000301457	0.062	0.929	0.9	0.992
MT-ND3	NDUFS8	9606.ENSP00000355206	9606.ENSP00000315774	0.136	0.939	0.9	0.994
MT-ND3	NDUFV1	9606.ENSP00000355206	9606.ENSP00000322450	0.405	0.935	0.9	0.995
MT-ND3	NDUFAF3	9606.ENSP00000355206	9606.ENSP00000323076	0	0	0.6	0.6
MT-ND3	NDUFV2	9606.ENSP00000355206	9606.ENSP00000327268	0.519	0.935	0.9	0.996
MT-ND3	NDUFA12	9606.ENSP00000355206	9606.ENSP00000330737	0.086	0.863	0.9	0.986
MT-ND3	NDUFB1	9606.ENSP00000355206	9606.ENSP00000330787	0	0.842	0.9	0.983
MT-ND3	NDUFA4	9606.ENSP00000355206	9606.ENSP00000339720	0.062	0	0.9	0.902
MT-ND3	NDUFV3	9606.ENSP00000355206	9606.ENSP00000346196	0	0.8	0.9	0.979
MT-ND3	MT-ND6	9606.ENSP00000355206	9606.ENSP00000354665	0.582	0.929	0.8	0.993
MT-ND3	MT-ND4L	9606.ENSP00000355206	9606.ENSP00000354728	0.596	0.929	0.54	0.985
MT-ND3	MT-ND5	9606.ENSP00000355206	9606.ENSP00000354813	0.5	0.929	0.9	0.996
MT-ND3	MT-ND4	9606.ENSP00000355206	9606.ENSP00000354961	0.74	0.944	0.9	0.998
MT-ND3	NDUFAF4	9606.ENSP00000355206	9606.ENSP00000358272	0	0	0.6	0.6
MT-ND3	NDUFA13	9606.ENSP00000355206	9606.ENSP00000423673	0.062	0.844	0.9	0.984
MT-ND3	NDUFC1	9606.ENSP00000355206	9606.ENSP00000441126	0.062	0.868	0.9	0.986
MT-ND3	NDUFA11	9606.ENSP00000355206	9606.ENSP00000389160	0.062	0.788	0.9	0.978
MT-ND3	NDUFB2	9606.ENSP00000355206	9606.ENSP00000419087	0.062	0.901	0.9	0.989
MT-ND3	NDUFA5	9606.ENSP00000355206	9606.ENSP00000417142	0.062	0.915	0.9	0.991
MT-ND3	NDUFA3	9606.ENSP00000355206	9606.ENSP00000418438	0	0.901	0.9	0.989
MT-ND3	NDUFB6	9606.ENSP00000355206	9606.ENSP00000369176	0.062	0.901	0.9	0.989
MT-ND3	NDUFA8	9606.ENSP00000355206	9606.ENSP00000362873	0.062	0.929	0.9	0.992
MT-ND3	NDUFS5	9606.ENSP00000355206	9606.ENSP00000362060	0	0.929	0.9	0.992
MT-ND3	NDUFA1	9606.ENSP00000355206	9606.ENSP00000360492	0	0.868	0.9	0.986
MT-ND3	NDUFS1	9606.ENSP00000355206	9606.ENSP00000392709	0.183	0.935	0.9	0.994
MT-ND3	NDUFAB1	9606.ENSP00000355206	9606.ENSP00000458770	0.062	0.987	0.9	0.998
MT-ND3	NDUFS2	9606.ENSP00000355206	9606.ENSP00000356972	0.643	0.944	0.9	0.997
MT-ND3	NDUFA6	9606.ENSP00000355206	9606.ENSP00000418842	0.062	0.993	0.9	0.999
MT-ND4	NDUFB4	9606.ENSP00000354961	9606.ENSP00000184266	0.125	0.929	0.9	0.993
MT-ND4	NDUFB7	9606.ENSP00000354961	9606.ENSP00000215565	0.327	0.929	0.9	0.994
MT-ND4	NDUFS7	9606.ENSP00000354961	9606.ENSP00000233627	0.322	0.968	0.9	0.997
MT-ND4	NDUFB3	9606.ENSP00000354961	9606.ENSP00000237889	0.303	0.868	0.9	0.99
MT-ND4	NDUFA2	9606.ENSP00000354961	9606.ENSP00000252102	0.286	0.993	0.9	0.999
MT-ND4	NDUFA10	9606.ENSP00000354961	9606.ENSP00000252711	0.073	0.901	0.9	0.99
MT-ND4	NDUFB5	9606.ENSP00000354961	9606.ENSP00000259037	0.101	0.97	0.9	0.997
MT-ND4	NDUFAF1	9606.ENSP00000354961	9606.ENSP00000260361	0.059	0	0.9	0.901
MT-ND4	NDUFS3	9606.ENSP00000354961	9606.ENSP00000263774	0.35	0.98	0.9	0.998
MT-ND4	NDUFA9	9606.ENSP00000354961	9606.ENSP00000266544	0.198	0.901	0.9	0.991
MT-ND4	NDUFB10	9606.ENSP00000354961	9606.ENSP00000268668	0.148	0.901	0.9	0.99
MT-ND4	NDUFS6	9606.ENSP00000354961	9606.ENSP00000274137	0.183	0.998	0.9	0.999
MT-ND4	NDUFB11	9606.ENSP00000354961	9606.ENSP00000276062	0.085	0.885	0.9	0.988
MT-ND4	NDUFB9	9606.ENSP00000354961	9606.ENSP00000276689	0.229	0.929	0.9	0.994
MT-ND4	NDUFC2	9606.ENSP00000354961	9606.ENSP00000281031	0.271	0.901	0.9	0.992

MT-ND4	NDUFS4	9606.ENSP00000354961	9606.ENSP00000296684	0.289	0.987	0.9	0.999
MT-ND4	NDUFB8	9606.ENSP00000354961	9606.ENSP00000299166	0.236	0.901	0.9	0.991
MT-ND4	NDUFA7	9606.ENSP00000354961	9606.ENSP00000301457	0.155	0.929	0.9	0.993
MT-ND4	NDUFS8	9606.ENSP00000354961	9606.ENSP00000315774	0.379	0.938	0.9	0.995
MT-ND4	NDUFV1	9606.ENSP00000354961	9606.ENSP00000322450	0.262	0.933	0.9	0.994
MT-ND4	NDUFAF3	9606.ENSP00000354961	9606.ENSP00000323076	0.075	0	0.6	0.614
MT-ND4	NDUFV2	9606.ENSP00000354961	9606.ENSP00000327268	0.345	0.933	0.9	0.995
MT-ND4	NDUFA12	9606.ENSP00000354961	9606.ENSP00000330737	0.284	0.863	0.9	0.989
MT-ND4	NDUFB1	9606.ENSP00000354961	9606.ENSP00000330787	0.146	0.842	0.9	0.985
MT-ND4	NDUFA4	9606.ENSP00000354961	9606.ENSP00000339720	0	0	0.9	0.9
MT-ND4	NDUFV3	9606.ENSP00000354961	9606.ENSP00000346196	0.058	0.8	0.9	0.979
MT-ND4	MT-ND6	9606.ENSP00000354961	9606.ENSP00000354665	0.759	0.929	0.6	0.992
MT-ND4	MT-ND4L	9606.ENSP00000354961	9606.ENSP00000354728	0.938	0.929	0.54	0.997
MT-ND4	MT-ND5	9606.ENSP00000354961	9606.ENSP00000354813	0.95	0.929	0.9	0.999
MT-ND4	NDUFAF4	9606.ENSP00000354961	9606.ENSP00000358272	0	0	0.6	0.6
MT-ND4	NDUFC1	9606.ENSP00000354961	9606.ENSP00000441126	0	0.868	0.9	0.986
MT-ND4	NDUFB2	9606.ENSP00000354961	9606.ENSP00000419087	0	0.901	0.9	0.989
MT-ND4	NDUFA3	9606.ENSP00000354961	9606.ENSP00000418438	0.27	0.901	0.9	0.992
MT-ND4	NDUFA13	9606.ENSP00000354961	9606.ENSP00000423673	0.305	0.913	0.9	0.993
MT-ND4	NDUFA5	9606.ENSP00000354961	9606.ENSP00000417142	0.184	0.915	0.9	0.992
MT-ND4	NDUFB6	9606.ENSP00000354961	9606.ENSP00000369176	0.302	0.901	0.9	0.992
MT-ND4	NDUFA1	9606.ENSP00000354961	9606.ENSP00000360492	0	0.868	0.9	0.986
MT-ND4	NDUFA8	9606.ENSP00000354961	9606.ENSP00000362873	0.231	0.975	0.9	0.997
MT-ND4	NDUFA11	9606.ENSP00000354961	9606.ENSP00000389160	0.302	0.957	0.9	0.996
MT-ND4	NDUFS1	9606.ENSP00000354961	9606.ENSP00000392709	0.527	0.933	0.9	0.996
MT-ND4	NDUFA6	9606.ENSP00000354961	9606.ENSP00000418842	0.159	0.993	0.9	0.999
MT-ND4	NDUFS2	9606.ENSP00000354961	9606.ENSP00000356972	0.234	0.944	0.9	0.995
MT-ND4	NDUFS5	9606.ENSP00000354961	9606.ENSP00000362060	0.327	0.993	0.9	0.999
MT-ND4	NDUFAB1	9606.ENSP00000354961	9606.ENSP00000458770	0.072	0.987	0.9	0.998
MT-ND4L	NDUFB4	9606.ENSP00000354728	9606.ENSP00000184266	0	0.929	0.54	0.966
MT-ND4L	NDUFB7	9606.ENSP00000354728	9606.ENSP00000215565	0	0.929	0.54	0.966
MT-ND4L	NDUFS7	9606.ENSP00000354728	9606.ENSP00000233627	0.158	0.929	0.54	0.97
MT-ND4L	NDUFB3	9606.ENSP00000354728	9606.ENSP00000237889	0	0.868	0.54	0.936
MT-ND4L	NDUFA2	9606.ENSP00000354728	9606.ENSP00000252102	0	0.929	0.54	0.966
MT-ND4L	NDUFA10	9606.ENSP00000354728	9606.ENSP00000252711	0	0.901	0.54	0.952
MT-ND4L	NDUFB5	9606.ENSP00000354728	9606.ENSP00000259037	0	0.929	0.54	0.966
MT-ND4L	NDUFAF1	9606.ENSP00000354728	9606.ENSP00000260361	0.062	0	0.54	0.55
MT-ND4L	NDUFS3	9606.ENSP00000354728	9606.ENSP00000263774	0.115	0.929	0.54	0.968
MT-ND4L	NDUFA9	9606.ENSP00000354728	9606.ENSP00000266544	0	0.901	0.54	0.952
MT-ND4L	NDUFB10	9606.ENSP00000354728	9606.ENSP00000268668	0	0.901	0.54	0.952
MT-ND4L	NDUFS6	9606.ENSP00000354728	9606.ENSP00000274137	0.049	0.868	0.54	0.937
MT-ND4L	NDUFB11	9606.ENSP00000354728	9606.ENSP00000276062	0.062	0.877	0.54	0.942
MT-ND4L	NDUFB9	9606.ENSP00000354728	9606.ENSP00000276689	0	0.929	0.54	0.966
MT-ND4L	NDUFC2	9606.ENSP00000354728	9606.ENSP00000281031	0	0.901	0.54	0.952

MT-ND4L	NDUFS4	9606.ENSP00000354728	9606.ENSP00000296684	0	0.868	0.54	0.936
MT-ND4L	NDUFB8	9606.ENSP00000354728	9606.ENSP00000299166	0.062	0.901	0.54	0.953
MT-ND4L	NDUFA7	9606.ENSP00000354728	9606.ENSP00000301457	0	0.929	0.54	0.966
MT-ND4L	NDUFS8	9606.ENSP00000354728	9606.ENSP00000315774	0.125	0.842	0.54	0.931
MT-ND4L	NDUFV1	9606.ENSP00000354728	9606.ENSP00000322450	0	0.929	0.54	0.966
MT-ND4L	NDUFV2	9606.ENSP00000354728	9606.ENSP00000327268	0	0.929	0.54	0.966
MT-ND4L	NDUFA12	9606.ENSP00000354728	9606.ENSP00000330737	0	0.863	0.54	0.934
MT-ND4L	NDUFB1	9606.ENSP00000354728	9606.ENSP00000330787	0	0.842	0.54	0.924
MT-ND4L	NDUFA4	9606.ENSP00000354728	9606.ENSP00000339720	0	0	0.54	0.54
MT-ND4L	NDUFV3	9606.ENSP00000354728	9606.ENSP00000346196	0	0.8	0.54	0.904
MT-ND4L	MT-ND6	9606.ENSP00000354728	9606.ENSP00000354665	0.836	0.929	0	0.988
MT-ND4L	NDUFA11	9606.ENSP00000354728	9606.ENSP00000389160	0	0.788	0.54	0.898
MT-ND4L	NDUFA13	9606.ENSP00000354728	9606.ENSP00000423673	0	0.844	0.54	0.925
MT-ND4L	NDUFC1	9606.ENSP00000354728	9606.ENSP00000441126	0	0.868	0.54	0.936
MT-ND4L	NDUFB2	9606.ENSP00000354728	9606.ENSP00000419087	0	0.901	0.54	0.952
MT-ND4L	NDUFA3	9606.ENSP00000354728	9606.ENSP00000418438	0	0.901	0.54	0.952
MT-ND4L	NDUFA8	9606.ENSP00000354728	9606.ENSP00000362873	0	0.929	0.54	0.966
MT-ND4L	NDUFS5	9606.ENSP00000354728	9606.ENSP00000362060	0	0.929	0.54	0.966
MT-ND4L	NDUFA6	9606.ENSP00000354728	9606.ENSP00000418842	0	0.929	0.54	0.966
MT-ND4L	NDUFS1	9606.ENSP00000354728	9606.ENSP00000392709	0.062	0.929	0.54	0.967
MT-ND4L	NDUFA5	9606.ENSP00000354728	9606.ENSP00000417142	0.062	0.915	0.54	0.96
MT-ND4L	NDUFB6	9606.ENSP00000354728	9606.ENSP00000369176	0	0.901	0.54	0.952
MT-ND4L	NDUFA1	9606.ENSP00000354728	9606.ENSP00000360492	0	0.868	0.54	0.936
MT-ND4L	NDUFS2	9606.ENSP00000354728	9606.ENSP00000356972	0.147	0.929	0.54	0.97
MT-ND4L	NDUFAB1	9606.ENSP00000354728	9606.ENSP00000458770	0.062	0.987	0.54	0.994
MT-ND4L	MT-ND5	9606.ENSP00000354728	9606.ENSP00000354813	0.898	0.929	0.54	0.996
MT-ND5	NDUFB4	9606.ENSP00000354813	9606.ENSP00000184266	0.153	0.929	0.9	0.993
MT-ND5	NDUFB7	9606.ENSP00000354813	9606.ENSP00000215565	0.328	0.929	0.9	0.994
MT-ND5	NDUFS7	9606.ENSP00000354813	9606.ENSP00000233627	0.33	0.945	0.9	0.996
MT-ND5	NDUFB3	9606.ENSP00000354813	9606.ENSP00000237889	0.272	0.868	0.9	0.989
MT-ND5	NDUFA2	9606.ENSP00000354813	9606.ENSP00000252102	0.271	0.929	0.9	0.994
MT-ND5	NDUFA10	9606.ENSP00000354813	9606.ENSP00000252711	0.065	0.901	0.9	0.989
MT-ND5	NDUFB5	9606.ENSP00000354813	9606.ENSP00000259037	0.097	0.972	0.9	0.997
MT-ND5	NDUFAF1	9606.ENSP00000354813	9606.ENSP00000260361	0.053	0	0.9	0.901
MT-ND5	NDUFS3	9606.ENSP00000354813	9606.ENSP00000263774	0.284	0.999	0.9	0.999
MT-ND5	NDUFA9	9606.ENSP00000354813	9606.ENSP00000266544	0.182	0.988	0.9	0.998
MT-ND5	NDUFB10	9606.ENSP00000354813	9606.ENSP00000268668	0.153	0.901	0.9	0.99
MT-ND5	NDUFS6	9606.ENSP00000354813	9606.ENSP00000274137	0.145	0.92	0.9	0.992
MT-ND5	NDUFB11	9606.ENSP00000354813	9606.ENSP00000276062	0.089	0.885	0.9	0.988
MT-ND5	NDUFB9	9606.ENSP00000354813	9606.ENSP00000276689	0.183	0.929	0.9	0.993
MT-ND5	NDUFC2	9606.ENSP00000354813	9606.ENSP00000281031	0.286	0.901	0.9	0.992
MT-ND5	NDUFS4	9606.ENSP00000354813	9606.ENSP00000296684	0.247	0.987	0.9	0.998
MT-ND5	NDUFB8	9606.ENSP00000354813	9606.ENSP00000299166	0.273	0.901	0.9	0.992
MT-ND5	NDUFA7	9606.ENSP00000354813	9606.ENSP00000301457	0.113	0.929	0.9	0.993

MT-ND5	NDUFS8	9606.ENSP00000354813	9606.ENSP00000315774	0.345	0.918	0.9	0.994
MT-ND5	NDUFV1	9606.ENSP00000354813	9606.ENSP00000322450	0.321	0.993	0.9	0.999
MT-ND5	NDUFAF3	9606.ENSP00000354813	9606.ENSP00000323076	0.068	0	0.6	0.611
MT-ND5	NDUFV2	9606.ENSP00000354813	9606.ENSP00000327268	0.275	0.993	0.9	0.999
MT-ND5	NDUFA12	9606.ENSP00000354813	9606.ENSP00000330737	0.188	0.987	0.9	0.998
MT-ND5	NDUFB1	9606.ENSP00000354813	9606.ENSP00000330787	0.115	0.842	0.9	0.984
MT-ND5	NDUFA4	9606.ENSP00000354813	9606.ENSP00000339720	0.062	0	0.9	0.902
MT-ND5	NDUFV3	9606.ENSP00000354813	9606.ENSP00000346196	0.061	0.8	0.9	0.979
MT-ND5	MT-ND6	9606.ENSP00000354813	9606.ENSP00000354665	0.894	0.929	0.6	0.996
MT-ND5	NDUFAF4	9606.ENSP00000354813	9606.ENSP00000358272	0	0	0.6	0.6
MT-ND5	NDUFC1	9606.ENSP00000354813	9606.ENSP00000441126	0	0.868	0.9	0.986
MT-ND5	NDUFA13	9606.ENSP00000354813	9606.ENSP00000423673	0.303	0.86	0.9	0.989
MT-ND5	NDUFB6	9606.ENSP00000354813	9606.ENSP00000369176	0.305	0.901	0.9	0.992
MT-ND5	NDUFA3	9606.ENSP00000354813	9606.ENSP00000418438	0.288	0.901	0.9	0.992
MT-ND5	NDUFA5	9606.ENSP00000354813	9606.ENSP00000417142	0.184	0.916	0.9	0.992
MT-ND5	NDUFB2	9606.ENSP00000354813	9606.ENSP00000419087	0	0.901	0.9	0.989
MT-ND5	NDUFA1	9606.ENSP00000354813	9606.ENSP00000360492	0.062	0.868	0.9	0.986
MT-ND5	NDUFA8	9606.ENSP00000354813	9606.ENSP00000362873	0.23	0.959	0.9	0.996
MT-ND5	NDUFS2	9606.ENSP00000354813	9606.ENSP00000356972	0.275	0.929	0.9	0.994
MT-ND5	NDUFS1	9606.ENSP00000354813	9606.ENSP00000392709	0.511	0.929	0.9	0.996
MT-ND5	NDUFA11	9606.ENSP00000354813	9606.ENSP00000389160	0.307	0.957	0.9	0.996
MT-ND5	NDUFA6	9606.ENSP00000354813	9606.ENSP00000418842	0.113	0.993	0.9	0.999
MT-ND5	NDUFS5	9606.ENSP00000354813	9606.ENSP00000362060	0.289	0.993	0.9	0.999
MT-ND5	NDUFAB1	9606.ENSP00000354813	9606.ENSP00000458770	0.061	0.987	0.9	0.998
MT-ND6	NDUFB4	9606.ENSP00000354665	9606.ENSP00000184266	0.062	0.929	0.6	0.971
MT-ND6	NDUFB7	9606.ENSP00000354665	9606.ENSP00000215565	0	0.929	0.6	0.97
MT-ND6	NDUFS7	9606.ENSP00000354665	9606.ENSP00000233627	0	0.929	0.6	0.97
MT-ND6	NDUFB3	9606.ENSP00000354665	9606.ENSP00000237889	0.062	0.868	0.6	0.946
MT-ND6	NDUFA2	9606.ENSP00000354665	9606.ENSP00000252102	0	0.929	0.6	0.97
MT-ND6	NDUFA10	9606.ENSP00000354665	9606.ENSP00000252711	0	0.901	0.6	0.958
MT-ND6	NDUFB5	9606.ENSP00000354665	9606.ENSP00000259037	0	0.929	0.6	0.97
MT-ND6	NDUFAF1	9606.ENSP00000354665	9606.ENSP00000260361	0	0	0.6	0.6
MT-ND6	NDUFS3	9606.ENSP00000354665	9606.ENSP00000263774	0.062	0.998	0.6	0.999
MT-ND6	NDUFA9	9606.ENSP00000354665	9606.ENSP00000266544	0	0.901	0.6	0.958
MT-ND6	NDUFB10	9606.ENSP00000354665	9606.ENSP00000268668	0.062	0.901	0.6	0.959
MT-ND6	NDUFS6	9606.ENSP00000354665	9606.ENSP00000274137	0.052	0.868	0.6	0.945
MT-ND6	NDUFB11	9606.ENSP00000354665	9606.ENSP00000276062	0.062	0.877	0.6	0.949
MT-ND6	NDUFB9	9606.ENSP00000354665	9606.ENSP00000276689	0.062	0.929	0.6	0.971
MT-ND6	NDUFC2	9606.ENSP00000354665	9606.ENSP00000281031	0	0.901	0.6	0.958
MT-ND6	NDUFS4	9606.ENSP00000354665	9606.ENSP00000296684	0	0.868	0.6	0.945
MT-ND6	NDUFB8	9606.ENSP00000354665	9606.ENSP00000299166	0.062	0.901	0.6	0.959
MT-ND6	NDUFA7	9606.ENSP00000354665	9606.ENSP00000301457	0	0.929	0.6	0.97
MT-ND6	NDUFS8	9606.ENSP00000354665	9606.ENSP00000315774	0	0.842	0.6	0.934
MT-ND6	NDUFV1	9606.ENSP00000354665	9606.ENSP00000322450	0.062	0.929	0.6	0.971

MT-ND6	NDUFAF3	9606.ENSP00000354665	9606.ENSP00000323076	0	0.913	0.6	0.963
MT-ND6	NDUFV2	9606.ENSP00000354665	9606.ENSP00000327268	0.062	0.929	0.6	0.971
MT-ND6	NDUFA12	9606.ENSP00000354665	9606.ENSP00000330737	0.062	0.863	0.6	0.944
MT-ND6	NDUFB1	9606.ENSP00000354665	9606.ENSP00000330787	0	0.842	0.6	0.934
MT-ND6	NDUFV3	9606.ENSP00000354665	9606.ENSP00000346196	0	0.8	0.6	0.916
MT-ND6	NDUFAF4	9606.ENSP00000354665	9606.ENSP00000358272	0	0	0.6	0.6
MT-ND6	NDUFA13	9606.ENSP00000354665	9606.ENSP00000423673	0.062	0.844	0.6	0.936
MT-ND6	NDUFA11	9606.ENSP00000354665	9606.ENSP00000389160	0	0.788	0.6	0.911
MT-ND6	NDUFC1	9606.ENSP00000354665	9606.ENSP00000441126	0	0.868	0.6	0.945
MT-ND6	NDUFB2	9606.ENSP00000354665	9606.ENSP00000419087	0.062	0.901	0.6	0.959
MT-ND6	NDUFA3	9606.ENSP00000354665	9606.ENSP00000418438	0	0.901	0.6	0.958
MT-ND6	NDUFA8	9606.ENSP00000354665	9606.ENSP00000362873	0.062	0.929	0.6	0.971
MT-ND6	NDUFA6	9606.ENSP00000354665	9606.ENSP00000418842	0.062	0.929	0.6	0.971
MT-ND6	NDUFA5	9606.ENSP00000354665	9606.ENSP00000417142	0.062	0.915	0.6	0.965
MT-ND6	NDUFS2	9606.ENSP00000354665	9606.ENSP00000356972	0	0.929	0.6	0.97
MT-ND6	NDUFS1	9606.ENSP00000354665	9606.ENSP00000392709	0	0.929	0.6	0.97
MT-ND6	NDUFB6	9606.ENSP00000354665	9606.ENSP00000369176	0	0.901	0.8	0.979
MT-ND6	NDUFA1	9606.ENSP00000354665	9606.ENSP00000360492	0	0.868	0.6	0.945
MT-ND6	NDUFAB1	9606.ENSP00000354665	9606.ENSP00000458770	0.062	0.987	0.6	0.994
MT-ND6	NDUFS5	9606.ENSP00000354665	9606.ENSP00000362060	0	0.993	0.6	0.997
MTNR1A	MTNR1B	9606.ENSP00000302811	9606.ENSP00000257068	0	0.462	0.8	0.887
MTOR	RPS6KB1	9606.ENSP00000354558	9606.ENSP00000225577	0.062	0.992	0.9	0.999
MTOR	NFKB1	9606.ENSP00000354558	9606.ENSP00000226574	0.054	0.159	0.9	0.913
MTOR	PRKCG	9606.ENSP00000354558	9606.ENSP00000263431	0.049	0	0.8	0.801
MTOR	PIK3CA	9606.ENSP00000354558	9606.ENSP00000263967	0.062	0.347	0.9	0.933
MTOR	STAT3	9606.ENSP00000354558	9606.ENSP00000264657	0.049	0.836	0.9	0.983
MTOR	PPARG	9606.ENSP00000354558	9606.ENSP00000287820	0	0.061	0.8	0.804
MTOR	PIK3CB	9606.ENSP00000354558	9606.ENSP00000289153	0.062	0.347	0.8	0.866
MTOR	PRKCB	9606.ENSP00000354558	9606.ENSP00000305355	0.049	0	0.8	0.801
MTOR	YWHAG	9606.ENSP00000354558	9606.ENSP00000306330	0.061	0.17	0.9	0.915
MTOR	TERT	9606.ENSP00000354558	9606.ENSP00000309572	0	0.33	0.9	0.93
MTOR	PRKDC	9606.ENSP00000354558	9606.ENSP00000313420	0.088	0.457	0	0.483
MTOR	PDK1	9606.ENSP00000354558	9606.ENSP00000376352	0.054	0.494	0	0.502
MTOR	PRKCD	9606.ENSP00000354558	9606.ENSP00000378217	0.094	0.423	0	0.454
MTOR	PIK3CD	9606.ENSP00000354558	9606.ENSP00000366563	0.062	0.347	0.8	0.866
MTOR	RELA	9606.ENSP00000354558	9606.ENSP00000384273	0	0	0.9	0.9
MTOR	PIK3R1	9606.ENSP00000354558	9606.ENSP00000428056	0.062	0.244	0.9	0.922
MTOR	PRKCA	9606.ENSP00000354558	9606.ENSP00000408695	0.049	0.27	0.9	0.924
NAMPT	PNP	9606.ENSP00000222553	9606.ENSP00000354532	0	0	0.9	0.9
NAMPT	SIRT2	9606.ENSP00000222553	9606.ENSP00000249396	0.055	0.101	0.9	0.907
NCOR1	NR3C1	9606.ENSP00000268712	9606.ENSP00000231509	0.049	0.971	0	0.971
NCOR1	NR1D1	9606.ENSP00000268712	9606.ENSP00000246672	0	0.868	0.6	0.945
NCOR1	NR1H2	9606.ENSP00000268712	9606.ENSP00000253727	0.062	0.748	0.6	0.897
NCOR1	RARA	9606.ENSP00000268712	9606.ENSP00000254066	0	0.983	0.6	0.993

NCOR1	RORA	9606.ENSP00000268712	9606.ENSP00000261523	0	0	0.6	0.6
NCOR1	THRA	9606.ENSP00000268712	9606.ENSP00000264637	0	0.899	0.6	0.957
NCOR1	SMARCA2	9606.ENSP00000268712	9606.ENSP00000265773	0.15	0.632	0	0.674
NCOR1	RARB	9606.ENSP00000268712	9606.ENSP00000332296	0	0.208	0.6	0.669
NCOR1	RXRG	9606.ENSP00000268712	9606.ENSP00000352900	0	0.258	0.6	0.69
NCOR1	RARG	9606.ENSP00000268712	9606.ENSP00000388510	0	0.397	0.6	0.748
NCOR1	RXRB	9606.ENSP00000268712	9606.ENSP00000363817	0	0.258	0.6	0.69
NCOR1	PPARD	9606.ENSP00000268712	9606.ENSP00000310928	0	0.862	0	0.862
NCOR1	NR1H4	9606.ENSP00000268712	9606.ENSP00000447149	0	0.873	0	0.873
NCOR1	PGR	9606.ENSP00000268712	9606.ENSP00000325120	0	0.873	0	0.873
NCOR1	RXRA	9606.ENSP00000268712	9606.ENSP00000419692	0.082	0.748	0.6	0.899
NCOR1	NR1H3	9606.ENSP00000268712	9606.ENSP00000477707	0.062	0.758	0.6	0.901
NCOR1	NCOR2	9606.ENSP00000268712	9606.ENSP00000384018	0.09	0.677	0.9	0.968
NCOR1	VDR	9606.ENSP00000268712	9606.ENSP00000447173	0	0.81	0	0.81
NCOR1	THRB	9606.ENSP00000268712	9606.ENSP00000379904	0	0.892	0.6	0.955
NCOR1	PPARA	9606.ENSP00000268712	9606.ENSP00000385523	0	0.895	0.9	0.989
NCOR1	PPARG	9606.ENSP00000268712	9606.ENSP00000287820	0	0.976	0.9	0.997
NCOR2	NFKB1	9606.ENSP00000384018	9606.ENSP00000226574	0.062	0.528	0	0.538
NCOR2	NR3C1	9606.ENSP00000384018	9606.ENSP00000231509	0	0.587	0	0.587
NCOR2	NR1D1	9606.ENSP00000384018	9606.ENSP00000246672	0.076	0.41	0	0.432
NCOR2	PIN1	9606.ENSP00000384018	9606.ENSP00000247970	0	0.494	0	0.494
NCOR2	NR1H2	9606.ENSP00000384018	9606.ENSP00000253727	0.062	0.589	0.6	0.832
NCOR2	RARA	9606.ENSP00000384018	9606.ENSP00000254066	0.086	0.892	0.9	0.989
NCOR2	THRA	9606.ENSP00000384018	9606.ENSP00000264637	0.07	0.732	0.9	0.972
NCOR2	PPARG	9606.ENSP00000384018	9606.ENSP00000287820	0	0.976	0.9	0.997
NCOR2	PPARD	9606.ENSP00000384018	9606.ENSP00000310928	0.089	0.575	0	0.596
NCOR2	PGR	9606.ENSP00000384018	9606.ENSP00000325120	0	0.943	0	0.943
NCOR2	RORC	9606.ENSP00000384018	9606.ENSP00000327025	0	0.8	0	0.8
NCOR2	RARB	9606.ENSP00000384018	9606.ENSP00000332296	0.051	0.413	0.9	0.939
NCOR2	NR1I2	9606.ENSP00000384018	9606.ENSP00000336528	0	0.733	0	0.733
NCOR2	VCP	9606.ENSP00000384018	9606.ENSP00000351777	0.062	0.486	0	0.497
NCOR2	RXRG	9606.ENSP00000384018	9606.ENSP00000352900	0	0.326	0.9	0.929
NCOR2	RXRB	9606.ENSP00000384018	9606.ENSP00000363817	0.065	0.258	0.9	0.924
NCOR2	THRB	9606.ENSP00000384018	9606.ENSP00000379904	0	0.896	0.9	0.989
NCOR2	RELA	9606.ENSP00000384018	9606.ENSP00000384273	0.084	0.675	0	0.689
NCOR2	NR4A1	9606.ENSP00000384018	9606.ENSP00000440864	0.062	0.486	0	0.497
NCOR2	VDR	9606.ENSP00000384018	9606.ENSP00000447173	0	0.587	0	0.587
NCOR2	NR1H3	9606.ENSP00000384018	9606.ENSP00000477707	0.062	0.531	0.6	0.808
NCOR2	RARG	9606.ENSP00000384018	9606.ENSP00000388510	0.084	0.413	0.9	0.941
NCOR2	RXRA	9606.ENSP00000384018	9606.ENSP00000419692	0.083	0.955	0.9	0.995
NCOR2	PPARA	9606.ENSP00000384018	9606.ENSP00000385523	0	0.972	0.9	0.997
NCSTN	PSEN1	9606.ENSP00000294785	9606.ENSP00000326366	0.083	0.989	0.9	0.998
NCSTN	PSEN2	9606.ENSP00000294785	9606.ENSP00000355747	0.063	0.839	0.8	0.967
NCSTN	PSENEN	9606.ENSP00000294785	9606.ENSP00000468411	0.062	0.987	0.9	0.998

NDUFA1	NDUFB4	9606.ENSP00000360492	9606.ENSP00000184266	0.714	0.868	0.9	0.995
NDUFA1	NDUFB7	9606.ENSP00000360492	9606.ENSP00000215565	0.304	0.868	0.9	0.99
NDUFA1	NDUFS7	9606.ENSP00000360492	9606.ENSP00000233627	0.14	0.868	0.9	0.987
NDUFA1	NDUFB3	9606.ENSP00000360492	9606.ENSP00000237889	0.873	0.842	0.9	0.997
NDUFA1	NDUFA2	9606.ENSP00000360492	9606.ENSP00000252102	0.852	0.868	0.9	0.997
NDUFA1	NDUFA10	9606.ENSP00000360492	9606.ENSP00000252711	0.062	0.844	0.9	0.984
NDUFA1	NDUFB5	9606.ENSP00000360492	9606.ENSP00000259037	0.42	0.868	0.9	0.991
NDUFA1	NDUFAF1	9606.ENSP00000360492	9606.ENSP00000260361	0	0	0.9	0.9
NDUFA1	NDUFS3	9606.ENSP00000360492	9606.ENSP00000263774	0.143	0.868	0.9	0.987
NDUFA1	NDUFA9	9606.ENSP00000360492	9606.ENSP00000266544	0.121	0.844	0.9	0.985
NDUFA1	NDUFB10	9606.ENSP00000360492	9606.ENSP00000268668	0.173	0.844	0.9	0.986
NDUFA1	NDUFS6	9606.ENSP00000360492	9606.ENSP00000274137	0.293	0.899	0.9	0.992
NDUFA1	NDUFB11	9606.ENSP00000360492	9606.ENSP00000276062	0.468	0.788	0.9	0.987
NDUFA1	NDUFB9	9606.ENSP00000360492	9606.ENSP00000276689	0.562	0.868	0.9	0.993
NDUFA1	NDUFC2	9606.ENSP00000360492	9606.ENSP00000281031	0.515	0.862	0.9	0.992
NDUFA1	NDUFS4	9606.ENSP00000360492	9606.ENSP00000296684	0.359	0.842	0.9	0.989
NDUFA1	NDUFB8	9606.ENSP00000360492	9606.ENSP00000299166	0.704	0.844	0.9	0.995
NDUFA1	NDUFA7	9606.ENSP00000360492	9606.ENSP00000301457	0.653	0.868	0.9	0.995
NDUFA1	NDUFS8	9606.ENSP00000360492	9606.ENSP00000315774	0.106	0.842	0.9	0.984
NDUFA1	NDUFV1	9606.ENSP00000360492	9606.ENSP00000322450	0.065	0.868	0.9	0.986
NDUFA1	NDUFAF3	9606.ENSP00000360492	9606.ENSP00000323076	0.089	0	0.6	0.62
NDUFA1	NDUFV2	9606.ENSP00000360492	9606.ENSP00000327268	0.201	0.868	0.9	0.988
NDUFA1	NDUFA12	9606.ENSP00000360492	9606.ENSP00000330737	0.691	0.863	0.9	0.995
NDUFA1	NDUFB1	9606.ENSP00000360492	9606.ENSP00000330787	0.399	0.842	0.9	0.989
NDUFA1	NDUFA4	9606.ENSP00000360492	9606.ENSP00000339720	0.575	0	0.9	0.955
NDUFA1	NDUFV3	9606.ENSP00000360492	9606.ENSP00000346196	0.234	0.8	0.9	0.983
NDUFA1	NDUFS2	9606.ENSP00000360492	9606.ENSP00000356972	0.065	0.868	0.9	0.986
NDUFA1	NDUFAF4	9606.ENSP00000360492	9606.ENSP00000358272	0.082	0	0.6	0.617
NDUFA1	NDUFA5	9606.ENSP00000360492	9606.ENSP00000417142	0.282	0.868	0.9	0.989
NDUFA1	NDUFA11	9606.ENSP00000360492	9606.ENSP00000389160	0.472	0.768	0.9	0.986
NDUFA1	NDUFS1	9606.ENSP00000360492	9606.ENSP00000392709	0.062	0.868	0.9	0.986
NDUFA1	NDUFA3	9606.ENSP00000360492	9606.ENSP00000418438	0.397	0.844	0.9	0.989
NDUFA1	NDUFB6	9606.ENSP00000360492	9606.ENSP00000369176	0.463	0.844	0.9	0.99
NDUFA1	NDUFA13	9606.ENSP00000360492	9606.ENSP00000423673	0.358	0.844	0.9	0.989
NDUFA1	NDUFC1	9606.ENSP00000360492	9606.ENSP00000441126	0.555	0.868	0.9	0.993
NDUFA1	NDUFS5	9606.ENSP00000360492	9606.ENSP00000362060	0.764	0.868	0.9	0.996
NDUFA1	NDUFA8	9606.ENSP00000360492	9606.ENSP00000362873	0.206	0.95	0.9	0.995
NDUFA1	NDUFB2	9606.ENSP00000360492	9606.ENSP00000419087	0.796	0.844	0.9	0.996
NDUFA1	NDUFAB1	9606.ENSP00000360492	9606.ENSP00000458770	0.271	0.987	0.9	0.999
NDUFA1	NDUFA6	9606.ENSP00000360492	9606.ENSP00000418842	0.537	0.987	0.9	0.999
NDUFA10	NDUFB4	9606.ENSP00000252711	9606.ENSP00000184266	0.106	0.974	0.9	0.997
NDUFA10	NDUFB7	9606.ENSP00000252711	9606.ENSP00000215565	0.666	0.994	0.9	0.999
NDUFA10	NDUFS7	9606.ENSP00000252711	9606.ENSP00000233627	0.703	0.987	0.9	0.999
NDUFA10	NDUFB3	9606.ENSP00000252711	9606.ENSP00000237889	0.163	0.976	0.9	0.997

NDUFA10	NDUFA2	9606.ENSP00000252711	9606.ENSP00000252102	0.203	0.997	0.9	0.999
NDUFA10	NDUFAF4	9606.ENSP00000252711	9606.ENSP00000358272	0.063	0	0.6	0.609
NDUFA10	NDUFAF3	9606.ENSP00000252711	9606.ENSP00000323076	0.063	0	0.6	0.609
NDUFA10	NDUFAF1	9606.ENSP00000252711	9606.ENSP00000260361	0.149	0	0.9	0.911
NDUFA10	NDUFA4	9606.ENSP00000252711	9606.ENSP00000339720	0.066	0.44	0.9	0.943
NDUFA10	NDUFC1	9606.ENSP00000252711	9606.ENSP00000441126	0.064	0.844	0.9	0.984
NDUFA10	NDUFV3	9606.ENSP00000252711	9606.ENSP00000346196	0.108	0.814	0.9	0.982
NDUFA10	NDUFB1	9606.ENSP00000252711	9606.ENSP00000330787	0.107	0.826	0.9	0.983
NDUFA10	NDUFB11	9606.ENSP00000252711	9606.ENSP00000276062	0.22	0.83	0.9	0.985
NDUFA10	NDUFA13	9606.ENSP00000252711	9606.ENSP00000423673	0.283	0.923	0.9	0.994
NDUFA10	NDUFA3	9606.ENSP00000252711	9606.ENSP00000418438	0.106	0.971	0.9	0.997
NDUFA10	NDUFB2	9606.ENSP00000252711	9606.ENSP00000419087	0.245	0.901	0.9	0.991
NDUFA10	NDUFA12	9606.ENSP00000252711	9606.ENSP00000330737	0.219	0.973	0.9	0.997
NDUFA10	NDUFAB1	9606.ENSP00000252711	9606.ENSP00000458770	0.395	0.987	0.9	0.999
NDUFA10	NDUFB9	9606.ENSP00000252711	9606.ENSP00000276689	0.74	0.986	0.9	0.999
NDUFA10	NDUFS5	9606.ENSP00000252711	9606.ENSP00000362060	0.455	0.985	0.9	0.999
NDUFA10	NDUFV2	9606.ENSP00000252711	9606.ENSP00000327268	0.574	0.994	0.9	0.999
NDUFA10	NDUFC2	9606.ENSP00000252711	9606.ENSP00000281031	0.656	0.953	0.9	0.998
NDUFA10	NDUFS4	9606.ENSP00000252711	9606.ENSP00000296684	0.529	0.987	0.9	0.999
NDUFA10	NDUFS1	9606.ENSP00000252711	9606.ENSP00000392709	0.887	0.987	0.9	0.999
NDUFA10	NDUFS3	9606.ENSP00000252711	9606.ENSP00000263774	0.554	0.962	0.9	0.998
NDUFA10	NDUFS6	9606.ENSP00000252711	9606.ENSP00000274137	0.497	0.987	0.9	0.999
NDUFA10	NDUFA11	9606.ENSP00000252711	9606.ENSP00000389160	0.666	0.942	0.9	0.997
NDUFA10	NDUFB8	9606.ENSP00000252711	9606.ENSP00000299166	0.852	0.901	0.9	0.998
NDUFA10	NDUFA8	9606.ENSP00000252711	9606.ENSP00000362873	0.809	0.987	0.9	0.999
NDUFA10	NDUFS2	9606.ENSP00000252711	9606.ENSP00000356972	0.816	0.992	0.9	0.999
NDUFA10	NDUFA9	9606.ENSP00000252711	9606.ENSP00000266544	0.783	0.997	0.9	0.999
NDUFA10	NDUFS8	9606.ENSP00000252711	9606.ENSP00000315774	0.624	0.965	0.9	0.998
NDUFA10	NDUFA6	9606.ENSP00000252711	9606.ENSP00000418842	0.548	0.995	0.9	0.999
NDUFA10	NDUFA5	9606.ENSP00000252711	9606.ENSP00000417142	0.316	0.988	0.9	0.999
NDUFA10	NDUFB10	9606.ENSP00000252711	9606.ENSP00000268668	0.852	0.987	0.9	0.999
NDUFA10	NDUFB6	9606.ENSP00000252711	9606.ENSP00000369176	0.206	0.985	0.9	0.998
NDUFA10	NDUFB5	9606.ENSP00000252711	9606.ENSP00000259037	0.828	0.972	0.9	0.999
NDUFA10	NDUFV1	9606.ENSP00000252711	9606.ENSP00000322450	0.769	0.99	0.9	0.999
NDUFA10	NDUFA7	9606.ENSP00000252711	9606.ENSP00000301457	0.23	0.969	0.9	0.997
NDUFA11	NDUFB4	9606.ENSP00000389160	9606.ENSP00000184266	0.46	0.957	0.9	0.997
NDUFA11	NDUFB7	9606.ENSP00000389160	9606.ENSP00000215565	0.934	0.788	0.9	0.998
NDUFA11	NDUFS7	9606.ENSP00000389160	9606.ENSP00000233627	0.895	0.957	0.9	0.999
NDUFA11	NDUFB3	9606.ENSP00000389160	9606.ENSP00000237889	0.695	0.94	0.9	0.998
NDUFA11	NDUFA2	9606.ENSP00000389160	9606.ENSP00000252102	0.924	0.997	0.9	0.999
NDUFA11	NDUFB5	9606.ENSP00000389160	9606.ENSP00000259037	0.862	0.957	0.9	0.999
NDUFA11	NDUFAF1	9606.ENSP00000389160	9606.ENSP00000260361	0.086	0	0.9	0.904
NDUFA11	NDUFS3	9606.ENSP00000389160	9606.ENSP00000263774	0.751	0.996	0.9	0.999
NDUFA11	NDUFA9	9606.ENSP00000389160	9606.ENSP00000266544	0.79	0.942	0.9	0.998

NDUFA11	NDUFB10	9606.ENSP00000389160	9606.ENSP00000268668	0.845	0.785	0.9	0.996
NDUFA11	NDUFS6	9606.ENSP00000389160	9606.ENSP00000274137	0.891	0.94	0.9	0.999
NDUFA11	NDUFB11	9606.ENSP00000389160	9606.ENSP00000276062	0.827	0.945	0.9	0.998
NDUFA11	NDUFB9	9606.ENSP00000389160	9606.ENSP00000276689	0.889	0.788	0.9	0.997
NDUFA11	NDUFC2	9606.ENSP00000389160	9606.ENSP00000281031	0.861	0.721	0.9	0.995
NDUFA11	NDUFS4	9606.ENSP00000389160	9606.ENSP00000296684	0.93	0.94	0.9	0.999
NDUFA11	NDUFB8	9606.ENSP00000389160	9606.ENSP00000299166	0.951	0.942	0.9	0.999
NDUFA11	NDUFA7	9606.ENSP00000389160	9606.ENSP00000301457	0.862	0.788	0.9	0.996
NDUFA11	NDUFS8	9606.ENSP00000389160	9606.ENSP00000315774	0.847	0.929	0.9	0.998
NDUFA11	NDUFV1	9606.ENSP00000389160	9606.ENSP00000322450	0.735	0.957	0.9	0.998
NDUFA11	NDUFAF3	9606.ENSP00000389160	9606.ENSP00000323076	0.24	0	0.6	0.683
NDUFA11	NDUFV2	9606.ENSP00000389160	9606.ENSP00000327268	0.867	0.957	0.9	0.999
NDUFA11	NDUFA12	9606.ENSP00000389160	9606.ENSP00000330737	0.897	0.933	0.9	0.999
NDUFA11	NDUFB1	9606.ENSP00000389160	9606.ENSP00000330787	0.32	0.71	0.9	0.978
NDUFA11	NDUFA4	9606.ENSP00000389160	9606.ENSP00000339720	0.138	0	0.9	0.91
NDUFA11	NDUFV3	9606.ENSP00000389160	9606.ENSP00000346196	0.171	0.913	0.9	0.992
NDUFA11	NDUFS2	9606.ENSP00000389160	9606.ENSP00000356972	0.52	0.957	0.9	0.997
NDUFA11	NDUFAF4	9606.ENSP00000389160	9606.ENSP00000358272	0.065	0	0.6	0.61
NDUFA11	NDUFS5	9606.ENSP00000389160	9606.ENSP00000362060	0.818	0.957	0.9	0.999
NDUFA11	NDUFA8	9606.ENSP00000389160	9606.ENSP00000362873	0.877	0.901	0.9	0.998
NDUFA11	NDUFB6	9606.ENSP00000389160	9606.ENSP00000369176	0.892	0.942	0.9	0.999
NDUFA11	NDUFC1	9606.ENSP00000389160	9606.ENSP00000441126	0.384	0.768	0.9	0.984
NDUFA11	NDUFB2	9606.ENSP00000389160	9606.ENSP00000419087	0.819	0.785	0.9	0.995
NDUFA11	NDUFS1	9606.ENSP00000389160	9606.ENSP00000392709	0.27	0.957	0.9	0.996
NDUFA11	NDUFA13	9606.ENSP00000389160	9606.ENSP00000423673	0.856	0.721	0.9	0.995
NDUFA11	NDUFA3	9606.ENSP00000389160	9606.ENSP00000418438	0.89	0.785	0.9	0.997
NDUFA11	NDUFA5	9606.ENSP00000389160	9606.ENSP00000417142	0.868	0.945	0.9	0.999
NDUFA11	NDUFA6	9606.ENSP00000389160	9606.ENSP00000418842	0.85	0.996	0.9	0.999
NDUFA11	NDUFAB1	9606.ENSP00000389160	9606.ENSP00000458770	0.747	0.994	0.9	0.999
NDUFA12	NDUFB4	9606.ENSP00000330737	9606.ENSP00000184266	0.879	0.987	0.9	0.999
NDUFA12	NDUFB7	9606.ENSP00000330737	9606.ENSP00000215565	0.866	0.995	0.9	0.999
NDUFA12	NDUFS7	9606.ENSP00000330737	9606.ENSP00000233627	0.737	0.998	0.9	0.999
NDUFA12	NDUFB3	9606.ENSP00000330737	9606.ENSP00000237889	0.948	0.991	0.9	0.999
NDUFA12	NDUFA2	9606.ENSP00000330737	9606.ENSP00000252102	0.828	0.998	0.9	0.999
NDUFA12	NDUFB5	9606.ENSP00000330737	9606.ENSP00000259037	0.895	0.987	0.9	0.999
NDUFA12	NDUFAF1	9606.ENSP00000330737	9606.ENSP00000260361	0.067	0.913	0.9	0.991
NDUFA12	NDUFS3	9606.ENSP00000330737	9606.ENSP00000263774	0.856	0.998	0.9	0.999
NDUFA12	NDUFA9	9606.ENSP00000330737	9606.ENSP00000266544	0.844	0.999	0.9	0.999
NDUFA12	NDUFB10	9606.ENSP00000330737	9606.ENSP00000268668	0.88	0.998	0.9	0.999
NDUFA12	NDUFS6	9606.ENSP00000330737	9606.ENSP00000274137	0.913	0.998	0.9	0.999
NDUFA12	NDUFB11	9606.ENSP00000330737	9606.ENSP00000276062	0.776	0.997	0.9	0.999
NDUFA12	NDUFB9	9606.ENSP00000330737	9606.ENSP00000276689	0.933	0.998	0.9	0.999
NDUFA12	NDUFC2	9606.ENSP00000330737	9606.ENSP00000281031	0.846	0.95	0.9	0.999
NDUFA12	NDUFAF2	9606.ENSP00000330737	9606.ENSP00000296597	0.225	0	0.8	0.838

NDUFA12	NDUFS4	9606.ENSP00000330737	9606.ENSP00000296684	0.96	0.998	0.9	0.999
NDUFA12	NDUFB8	9606.ENSP00000330737	9606.ENSP00000299166	0.932	0.985	0.9	0.999
NDUFA12	NDUFA7	9606.ENSP00000330737	9606.ENSP00000301457	0.573	0.987	0.9	0.999
NDUFA12	NDUFS8	9606.ENSP00000330737	9606.ENSP00000315774	0.82	0.998	0.9	0.999
NDUFA12	NDUFV1	9606.ENSP00000330737	9606.ENSP00000322450	0.507	0.995	0.9	0.999
NDUFA12	NDUFV2	9606.ENSP00000330737	9606.ENSP00000327268	0.9	0.995	0.9	0.999
NDUFA12	NDUFB2	9606.ENSP00000330737	9606.ENSP00000419087	0.389	0.842	0.9	0.989
NDUFA12	NDUFA3	9606.ENSP00000330737	9606.ENSP00000418438	0.553	0.842	0.9	0.992
NDUFA12	NDUFA4	9606.ENSP00000330737	9606.ENSP00000339720	0.676	0.829	0.9	0.993
NDUFA12	NDUFB1	9606.ENSP00000330737	9606.ENSP00000330787	0.746	0.839	0.9	0.995
NDUFA12	NDUFC1	9606.ENSP00000330737	9606.ENSP00000441126	0.832	0.863	0.9	0.997
NDUFA12	NDUFS2	9606.ENSP00000330737	9606.ENSP00000356972	0.308	0.995	0.9	0.999
NDUFA12	NDUFAB1	9606.ENSP00000330737	9606.ENSP00000458770	0.899	0.987	0.9	0.999
NDUFA12	NDUFS5	9606.ENSP00000330737	9606.ENSP00000362060	0.832	0.995	0.9	0.999
NDUFA12	NDUFA8	9606.ENSP00000330737	9606.ENSP00000362873	0.877	0.99	0.9	0.999
NDUFA12	NDUFA6	9606.ENSP00000330737	9606.ENSP00000418842	0.945	0.998	0.9	0.999
NDUFA12	NDUFV3	9606.ENSP00000330737	9606.ENSP00000346196	0.126	0.982	0.9	0.998
NDUFA12	NDUFA5	9606.ENSP00000330737	9606.ENSP00000417142	0.907	0.995	0.9	0.999
NDUFA12	NDUFB6	9606.ENSP00000330737	9606.ENSP00000369176	0.93	0.995	0.9	0.999
NDUFA12	NDUFS1	9606.ENSP00000330737	9606.ENSP00000392709	0.357	0.998	0.9	0.999
NDUFA12	NDUFA13	9606.ENSP00000330737	9606.ENSP00000423673	0.902	0.999	0.9	0.999
NDUFA13	NDUFB4	9606.ENSP00000423673	9606.ENSP00000184266	0.453	0.844	0.9	0.99
NDUFA13	NDUFB7	9606.ENSP00000423673	9606.ENSP00000215565	0.985	0.955	0.9	0.999
NDUFA13	NDUFS7	9606.ENSP00000423673	9606.ENSP00000233627	0.923	0.999	0.9	0.999
NDUFA13	NDUFB3	9606.ENSP00000423673	9606.ENSP00000237889	0.926	0.985	0.9	0.999
NDUFA13	NDUFA2	9606.ENSP00000423673	9606.ENSP00000252102	0.788	0.994	0.9	0.999
NDUFA13	NDUFB5	9606.ENSP00000423673	9606.ENSP00000259037	0.529	0.914	0.9	0.995
NDUFA13	NDUFAF1	9606.ENSP00000423673	9606.ENSP00000260361	0.075	0.992	0.9	0.999
NDUFA13	NDUFS3	9606.ENSP00000423673	9606.ENSP00000263774	0.841	0.999	0.9	0.999
NDUFA13	STAT3	9606.ENSP00000423673	9606.ENSP00000264657	0	0.576	0	0.576
NDUFA13	NDUFA9	9606.ENSP00000423673	9606.ENSP00000266544	0.835	0.994	0.9	0.999
NDUFA13	NDUFB10	9606.ENSP00000423673	9606.ENSP00000268668	0.845	0.952	0.9	0.999
NDUFA13	NDUFS6	9606.ENSP00000423673	9606.ENSP00000274137	0.799	0.975	0.9	0.999
NDUFA13	NDUFB11	9606.ENSP00000423673	9606.ENSP00000276062	0.866	0.76	0.9	0.996
NDUFA13	NDUFB9	9606.ENSP00000423673	9606.ENSP00000276689	0.969	0.953	0.9	0.999
NDUFA13	NDUFC2	9606.ENSP00000423673	9606.ENSP00000281031	0.82	0.983	0.9	0.999
NDUFA13	NDUFS4	9606.ENSP00000423673	9606.ENSP00000296684	0.848	0.999	0.9	0.999
NDUFA13	NDUFB8	9606.ENSP00000423673	9606.ENSP00000299166	0.857	0.844	0.9	0.997
NDUFA13	NDUFA7	9606.ENSP00000423673	9606.ENSP00000301457	0.761	0.914	0.9	0.997
NDUFA13	NDUFS8	9606.ENSP00000423673	9606.ENSP00000315774	0.957	0.982	0.9	0.999
NDUFA13	NDUFV1	9606.ENSP00000423673	9606.ENSP00000322450	0.848	0.994	0.9	0.999
NDUFA13	NDUFAF3	9606.ENSP00000423673	9606.ENSP00000323076	0.269	0.913	0.6	0.972
NDUFA13	NDUFV2	9606.ENSP00000423673	9606.ENSP00000327268	0.762	0.994	0.9	0.999
NDUFA13	NDUFB1	9606.ENSP00000423673	9606.ENSP00000330787	0.436	0.824	0.9	0.989

NDUFA13	NDUFA4	9606.ENSP00000423673	9606.ENSP00000339720	0.235	0.217	0.9	0.934
NDUFA13	NDUFV3	9606.ENSP00000423673	9606.ENSP00000346196	0.171	0.891	0.9	0.99
NDUFA13	NDUFS2	9606.ENSP00000423673	9606.ENSP00000356972	0.424	0.993	0.9	0.999
NDUFA13	NDUFAF4	9606.ENSP00000423673	9606.ENSP00000358272	0.068	0.992	0.6	0.996
NDUFA13	NDUFS5	9606.ENSP00000423673	9606.ENSP00000362060	0.951	0.99	0.9	0.999
NDUFA13	NDUFA8	9606.ENSP00000423673	9606.ENSP00000362873	0.875	0.955	0.9	0.999
NDUFA13	NDUFB6	9606.ENSP00000423673	9606.ENSP00000369176	0.888	0.952	0.9	0.999
NDUFA13	NDUFS1	9606.ENSP00000423673	9606.ENSP00000392709	0.627	0.994	0.9	0.999
NDUFA13	NDUFA5	9606.ENSP00000423673	9606.ENSP00000417142	0.67	0.994	0.9	0.999
NDUFA13	NDUFA3	9606.ENSP00000423673	9606.ENSP00000418438	0.748	0.915	0.9	0.997
NDUFA13	NDUFA6	9606.ENSP00000423673	9606.ENSP00000418842	0.877	0.994	0.9	0.999
NDUFA13	NDUFB2	9606.ENSP00000423673	9606.ENSP00000419087	0.317	0.844	0.9	0.988
NDUFA13	NDUFC1	9606.ENSP00000423673	9606.ENSP00000441126	0.226	0.844	0.9	0.986
NDUFA13	NDUFAB1	9606.ENSP00000423673	9606.ENSP00000458770	0.652	0.987	0.9	0.999
NDUFA2	NDUFB4	9606.ENSP00000252102	9606.ENSP00000184266	0.772	0.987	0.9	0.999
NDUFA2	NDUFB7	9606.ENSP00000252102	9606.ENSP00000215565	0.948	0.998	0.9	0.999
NDUFA2	NDUFS7	9606.ENSP00000252102	9606.ENSP00000233627	0.757	0.998	0.9	0.999
NDUFA2	NDUFB3	9606.ENSP00000252102	9606.ENSP00000237889	0.889	0.987	0.9	0.999
NDUFA2	NDUFAF3	9606.ENSP00000252102	9606.ENSP00000323076	0.105	0	0.6	0.626
NDUFA2	NDUFAF4	9606.ENSP00000252102	9606.ENSP00000358272	0.07	0	0.6	0.612
NDUFA2	NDUFAF1	9606.ENSP00000252102	9606.ENSP00000260361	0.076	0	0.9	0.903
NDUFA2	NDUFC1	9606.ENSP00000252102	9606.ENSP00000441126	0.719	0.868	0.9	0.995
NDUFA2	NDUFB1	9606.ENSP00000252102	9606.ENSP00000330787	0.683	0.842	0.9	0.994
NDUFA2	NDUFA4	9606.ENSP00000252102	9606.ENSP00000339720	0.338	0.992	0.9	0.999
NDUFA2	NDUFS2	9606.ENSP00000252102	9606.ENSP00000356972	0.373	0.998	0.9	0.999
NDUFA2	NDUFA9	9606.ENSP00000252102	9606.ENSP00000266544	0.378	0.999	0.9	0.999
NDUFA2	NDUFAB1	9606.ENSP00000252102	9606.ENSP00000458770	0.42	0.987	0.9	0.999
NDUFA2	NDUFB9	9606.ENSP00000252102	9606.ENSP00000276689	0.929	0.998	0.9	0.999
NDUFA2	NDUFS5	9606.ENSP00000252102	9606.ENSP00000362060	0.912	0.998	0.9	0.999
NDUFA2	NDUFB8	9606.ENSP00000252102	9606.ENSP00000299166	0.943	0.988	0.9	0.999
NDUFA2	NDUFA8	9606.ENSP00000252102	9606.ENSP00000362873	0.799	0.998	0.9	0.999
NDUFA2	NDUFA3	9606.ENSP00000252102	9606.ENSP00000418438	0.783	0.981	0.9	0.999
NDUFA2	NDUFB2	9606.ENSP00000252102	9606.ENSP00000419087	0.86	0.901	0.9	0.998
NDUFA2	NDUFA6	9606.ENSP00000252102	9606.ENSP00000418842	0.951	0.998	0.9	0.999
NDUFA2	NDUFB11	9606.ENSP00000252102	9606.ENSP00000276062	0.392	0.996	0.9	0.999
NDUFA2	NDUFS8	9606.ENSP00000252102	9606.ENSP00000315774	0.542	0.998	0.9	0.999
NDUFA2	NDUFV3	9606.ENSP00000252102	9606.ENSP00000346196	0.254	0.998	0.9	0.999
NDUFA2	NDUFC2	9606.ENSP00000252102	9606.ENSP00000281031	0.722	0.99	0.9	0.999
NDUFA2	NDUFS4	9606.ENSP00000252102	9606.ENSP00000296684	0.694	0.998	0.9	0.999
NDUFA2	NDUFV2	9606.ENSP00000252102	9606.ENSP00000327268	0.912	0.998	0.9	0.999
NDUFA2	NDUFA5	9606.ENSP00000252102	9606.ENSP00000417142	0.952	0.998	0.9	0.999
NDUFA2	NDUFB10	9606.ENSP00000252102	9606.ENSP00000268668	0.652	0.998	0.9	0.999
NDUFA2	NDUFB6	9606.ENSP00000252102	9606.ENSP00000369176	0.742	0.981	0.9	0.999
NDUFA2	NDUFV1	9606.ENSP00000252102	9606.ENSP00000322450	0.386	0.998	0.9	0.999

NDUFA2	NDUFS3	9606.ENSP00000252102	9606.ENSP00000263774	0.492	0.998	0.9	0.999
NDUFA2	NDUFA7	9606.ENSP00000252102	9606.ENSP00000301457	0.632	0.998	0.9	0.999
NDUFA2	NDUFS6	9606.ENSP00000252102	9606.ENSP00000274137	0.912	0.998	0.9	0.999
NDUFA2	NDUFS1	9606.ENSP00000252102	9606.ENSP00000392709	0.334	0.998	0.9	0.999
NDUFA2	NDUFB5	9606.ENSP00000252102	9606.ENSP00000259037	0.892	0.993	0.9	0.999
NDUFA3	NDUFB4	9606.ENSP00000418438	9606.ENSP00000184266	0.466	0.981	0.9	0.998
NDUFA3	NDUFB7	9606.ENSP00000418438	9606.ENSP00000215565	0.912	0.981	0.9	0.999
NDUFA3	NDUFS7	9606.ENSP00000418438	9606.ENSP00000233627	0.621	0.972	0.9	0.998
NDUFA3	NDUFB3	9606.ENSP00000418438	9606.ENSP00000237889	0.552	0.868	0.9	0.993
NDUFA3	NDUFB5	9606.ENSP00000418438	9606.ENSP00000259037	0.36	0.901	0.9	0.993
NDUFA3	NDUFAF1	9606.ENSP00000418438	9606.ENSP00000260361	0.049	0	0.9	0.9
NDUFA3	NDUFS3	9606.ENSP00000418438	9606.ENSP00000263774	0.267	0.969	0.9	0.997
NDUFA3	NDUFA9	9606.ENSP00000418438	9606.ENSP00000266544	0.394	0.901	0.9	0.993
NDUFA3	NDUFB10	9606.ENSP00000418438	9606.ENSP00000268668	0.399	0.901	0.9	0.993
NDUFA3	NDUFS6	9606.ENSP00000418438	9606.ENSP00000274137	0.401	0.955	0.9	0.997
NDUFA3	NDUFB11	9606.ENSP00000418438	9606.ENSP00000276062	0.249	0.841	0.9	0.987
NDUFA3	NDUFB9	9606.ENSP00000418438	9606.ENSP00000276689	0.525	0.901	0.9	0.994
NDUFA3	NDUFC2	9606.ENSP00000418438	9606.ENSP00000281031	0.532	0.95	0.9	0.997
NDUFA3	NDUFS4	9606.ENSP00000418438	9606.ENSP00000296684	0.579	0.955	0.9	0.997
NDUFA3	NDUFB8	9606.ENSP00000418438	9606.ENSP00000299166	0.737	0.901	0.9	0.997
NDUFA3	NDUFA7	9606.ENSP00000418438	9606.ENSP00000301457	0.726	0.974	0.9	0.999
NDUFA3	NDUFS8	9606.ENSP00000418438	9606.ENSP00000315774	0.459	0.821	0.9	0.989
NDUFA3	NDUFV1	9606.ENSP00000418438	9606.ENSP00000322450	0.311	0.972	0.9	0.997
NDUFA3	NDUFAF3	9606.ENSP00000418438	9606.ENSP00000323076	0.146	0	0.6	0.643
NDUFA3	NDUFV2	9606.ENSP00000418438	9606.ENSP00000327268	0.349	0.974	0.9	0.998
NDUFA3	NDUFB1	9606.ENSP00000418438	9606.ENSP00000330787	0.289	0.823	0.9	0.986
NDUFA3	NDUFA4	9606.ENSP00000418438	9606.ENSP00000339720	0.176	0.385	0.9	0.944
NDUFA3	NDUFV3	9606.ENSP00000418438	9606.ENSP00000346196	0.142	0.816	0.9	0.982
NDUFA3	NDUFS2	9606.ENSP00000418438	9606.ENSP00000356972	0.154	0.974	0.9	0.997
NDUFA3	NDUFAF4	9606.ENSP00000418438	9606.ENSP00000358272	0.062	0	0.6	0.608
NDUFA3	NDUFS5	9606.ENSP00000418438	9606.ENSP00000362060	0.599	0.972	0.9	0.998
NDUFA3	NDUFA8	9606.ENSP00000418438	9606.ENSP00000362873	0.45	0.969	0.9	0.998
NDUFA3	NDUFB6	9606.ENSP00000418438	9606.ENSP00000369176	0.652	0.901	0.9	0.996
NDUFA3	NDUFS1	9606.ENSP00000418438	9606.ENSP00000392709	0.201	0.961	0.9	0.996
NDUFA3	NDUFA5	9606.ENSP00000418438	9606.ENSP00000417142	0.57	0.968	0.9	0.998
NDUFA3	NDUFC1	9606.ENSP00000418438	9606.ENSP00000441126	0.392	0.844	0.9	0.989
NDUFA3	NDUFB2	9606.ENSP00000418438	9606.ENSP00000419087	0.674	0.901	0.9	0.996
NDUFA3	NDUFAB1	9606.ENSP00000418438	9606.ENSP00000458770	0.1	0.987	0.9	0.998
NDUFA3	NDUFA6	9606.ENSP00000418438	9606.ENSP00000418842	0.723	0.99	0.9	0.999
NDUFA4	NDUFB4	9606.ENSP00000339720	9606.ENSP00000184266	0.825	0.504	0.9	0.99
NDUFA4	NDUFB7	9606.ENSP00000339720	9606.ENSP00000215565	0.168	0.456	0.9	0.95
NDUFA4	NDUFS7	9606.ENSP00000339720	9606.ENSP00000233627	0.089	0.452	0.9	0.945
NDUFA4	NDUFB3	9606.ENSP00000339720	9606.ENSP00000237889	0.722	0.275	0.9	0.978
NDUFA4	NDUFB5	9606.ENSP00000339720	9606.ENSP00000259037	0.452	0.275	0.9	0.956

NDUFA4	NDUFAF1	9606.ENSP00000339720	9606.ENSP00000260361	0.068	0	0.9	0.902
NDUFA4	NDUFS3	9606.ENSP00000339720	9606.ENSP00000263774	0.48	0.446	0.9	0.968
NDUFA4	NDUFA9	9606.ENSP00000339720	9606.ENSP00000266544	0.112	0.311	0.9	0.933
NDUFA4	NDUFB10	9606.ENSP00000339720	9606.ENSP00000268668	0.375	0.439	0.9	0.961
NDUFA4	NDUFS6	9606.ENSP00000339720	9606.ENSP00000274137	0.211	0.462	0.9	0.953
NDUFA4	NDUFB11	9606.ENSP00000339720	9606.ENSP00000276062	0.15	0.275	0.9	0.933
NDUFA4	NDUFB9	9606.ENSP00000339720	9606.ENSP00000276689	0.387	0.413	0.9	0.96
NDUFA4	NDUFC2	9606.ENSP00000339720	9606.ENSP00000281031	0.275	0.432	0.9	0.955
NDUFA4	NDUFS4	9606.ENSP00000339720	9606.ENSP00000296684	0.407	0.503	0.9	0.968
NDUFA4	NDUFB8	9606.ENSP00000339720	9606.ENSP00000299166	0.658	0.275	0.9	0.973
NDUFA4	NDUFA7	9606.ENSP00000339720	9606.ENSP00000301457	0.212	0.462	0.9	0.953
NDUFA4	NDUFS8	9606.ENSP00000339720	9606.ENSP00000315774	0.109	0.206	0.9	0.923
NDUFA4	NDUFV1	9606.ENSP00000339720	9606.ENSP00000322450	0.09	0.443	0.9	0.944
NDUFA4	NDUFV2	9606.ENSP00000339720	9606.ENSP00000327268	0.288	0.462	0.9	0.958
NDUFA4	NDUFB1	9606.ENSP00000339720	9606.ENSP00000330787	0.306	0.275	0.9	0.945
NDUFA4	NDUFA4L2	9606.ENSP00000339720	9606.ENSP00000377411	0	0	0.8	0.8
NDUFA4	NDUFV3	9606.ENSP00000339720	9606.ENSP00000346196	0.082	0.385	0.9	0.938
NDUFA4	NDUFS1	9606.ENSP00000339720	9606.ENSP00000392709	0.063	0.438	0.9	0.942
NDUFA4	NDUFA8	9606.ENSP00000339720	9606.ENSP00000362873	0.254	0.448	0.9	0.955
NDUFA4	NDUFB2	9606.ENSP00000339720	9606.ENSP00000419087	0.59	0	0.9	0.957
NDUFA4	NDUFS2	9606.ENSP00000339720	9606.ENSP00000356972	0.108	0.447	0.9	0.946
NDUFA4	NDUFB6	9606.ENSP00000339720	9606.ENSP00000369176	0.549	0	0.9	0.952
NDUFA4	NDUFC1	9606.ENSP00000339720	9606.ENSP00000441126	0.678	0	0.9	0.966
NDUFA4	NDUFA5	9606.ENSP00000339720	9606.ENSP00000417142	0.312	0.449	0.9	0.958
NDUFA4	NDUFAB1	9606.ENSP00000339720	9606.ENSP00000458770	0.614	0.385	0.9	0.974
NDUFA4	NDUFS5	9606.ENSP00000339720	9606.ENSP00000362060	0.755	0.464	0.9	0.985
NDUFA4	NDUFA6	9606.ENSP00000339720	9606.ENSP00000418842	0.637	0.992	0.9	0.999
NDUFA4L2	NDUFA6	9606.ENSP00000377411	9606.ENSP00000418842	0.493	0.078	0	0.512
NDUFA5	NDUFB4	9606.ENSP00000417142	9606.ENSP00000184266	0.702	0.986	0.9	0.999
NDUFA5	NDUFB7	9606.ENSP00000417142	9606.ENSP00000215565	0.946	0.987	0.9	0.999
NDUFA5	NDUFS7	9606.ENSP00000417142	9606.ENSP00000233627	0.875	0.997	0.9	0.999
NDUFA5	NDUFB3	9606.ENSP00000417142	9606.ENSP00000237889	0.906	0.868	0.9	0.998
NDUFA5	NDUFB5	9606.ENSP00000417142	9606.ENSP00000259037	0.929	0.986	0.9	0.999
NDUFA5	NDUFAF1	9606.ENSP00000417142	9606.ENSP00000260361	0.281	0	0.9	0.925
NDUFA5	NDUFS3	9606.ENSP00000417142	9606.ENSP00000263774	0.945	0.998	0.9	0.999
NDUFA5	NDUFA9	9606.ENSP00000417142	9606.ENSP00000266544	0.412	0.997	0.9	0.999
NDUFA5	NDUFB10	9606.ENSP00000417142	9606.ENSP00000268668	0.921	0.994	0.9	0.999
NDUFA5	NDUFS6	9606.ENSP00000417142	9606.ENSP00000274137	0.94	0.987	0.9	0.999
NDUFA5	NDUFB11	9606.ENSP00000417142	9606.ENSP00000276062	0.704	0.831	0.9	0.994
NDUFA5	NDUFB9	9606.ENSP00000417142	9606.ENSP00000276689	0.957	0.995	0.9	0.999
NDUFA5	NDUFC2	9606.ENSP00000417142	9606.ENSP00000281031	0.742	0.954	0.9	0.998
NDUFA5	NDUFS4	9606.ENSP00000417142	9606.ENSP00000296684	0.91	0.987	0.9	0.999
NDUFA5	NDUFB8	9606.ENSP00000417142	9606.ENSP00000299166	0.937	0.874	0.9	0.999
NDUFA5	NDUFA7	9606.ENSP00000417142	9606.ENSP00000301457	0.703	0.98	0.9	0.999

NDUFA5	NDUFS8	9606.ENSP00000417142	9606.ENSP00000315774	0.818	0.987	0.9	0.999
NDUFA5	NDUFV1	9606.ENSP00000417142	9606.ENSP00000322450	0.462	0.987	0.9	0.999
NDUFA5	NDUFAF3	9606.ENSP00000417142	9606.ENSP00000323076	0.106	0	0.6	0.627
NDUFA5	NDUFV2	9606.ENSP00000417142	9606.ENSP00000327268	0.977	0.987	0.9	0.999
NDUFA5	NDUFB1	9606.ENSP00000417142	9606.ENSP00000330787	0.433	0.88	0.9	0.992
NDUFA5	NDUFV3	9606.ENSP00000417142	9606.ENSP00000346196	0.165	0.816	0.9	0.983
NDUFA5	NDUFS2	9606.ENSP00000417142	9606.ENSP00000356972	0.513	0.985	0.9	0.999
NDUFA5	NDUFAF4	9606.ENSP00000417142	9606.ENSP00000358272	0.226	0.294	0.6	0.762
NDUFA5	NDUFS5	9606.ENSP00000417142	9606.ENSP00000362060	0.96	0.995	0.9	0.999
NDUFA5	NDUFA8	9606.ENSP00000417142	9606.ENSP00000362873	0.939	0.988	0.9	0.999
NDUFA5	NDUFB6	9606.ENSP00000417142	9606.ENSP00000369176	0.848	0.976	0.9	0.999
NDUFA5	NDUFS1	9606.ENSP00000417142	9606.ENSP00000392709	0.367	0.988	0.9	0.999
NDUFA5	NDUFB2	9606.ENSP00000417142	9606.ENSP00000419087	0.524	0.874	0.9	0.993
NDUFA5	NDUFA6	9606.ENSP00000417142	9606.ENSP00000418842	0.952	0.995	0.9	0.999
NDUFA5	NDUFAB1	9606.ENSP00000417142	9606.ENSP00000458770	0.887	0.987	0.9	0.999
NDUFA5	NDUFC1	9606.ENSP00000417142	9606.ENSP00000441126	0.828	0.868	0.9	0.997
NDUFA6	NDUFB4	9606.ENSP00000418842	9606.ENSP00000184266	0.751	0.993	0.9	0.999
NDUFA6	NDUFB7	9606.ENSP00000418842	9606.ENSP00000215565	0.939	0.995	0.9	0.999
NDUFA6	NDUFS7	9606.ENSP00000418842	9606.ENSP00000233627	0.773	0.995	0.9	0.999
NDUFA6	NDUFB3	9606.ENSP00000418842	9606.ENSP00000237889	0.876	0.987	0.9	0.999
NDUFA6	NDUFB5	9606.ENSP00000418842	9606.ENSP00000259037	0.932	0.993	0.9	0.999
NDUFA6	NDUFAF1	9606.ENSP00000418842	9606.ENSP00000260361	0.088	0.27	0.9	0.927
NDUFA6	NDUFS3	9606.ENSP00000418842	9606.ENSP00000263774	0.89	0.998	0.9	0.999
NDUFA6	NDUFA9	9606.ENSP00000418842	9606.ENSP00000266544	0.487	0.999	0.9	0.999
NDUFA6	NDUFB10	9606.ENSP00000418842	9606.ENSP00000268668	0.894	0.995	0.9	0.999
NDUFA6	NDUFS6	9606.ENSP00000418842	9606.ENSP00000274137	0.936	0.998	0.9	0.999
NDUFA6	NDUFB11	9606.ENSP00000418842	9606.ENSP00000276062	0.926	0.997	0.9	0.999
NDUFA6	NDUFB9	9606.ENSP00000418842	9606.ENSP00000276689	0.969	0.998	0.9	0.999
NDUFA6	NDUFC2	9606.ENSP00000418842	9606.ENSP00000281031	0.855	0.993	0.9	0.999
NDUFA6	NDUFS4	9606.ENSP00000418842	9606.ENSP00000296684	0.895	0.995	0.9	0.999
NDUFA6	NDUFB8	9606.ENSP00000418842	9606.ENSP00000299166	0.941	0.988	0.9	0.999
NDUFA6	NDUFA7	9606.ENSP00000418842	9606.ENSP00000301457	0.855	0.993	0.9	0.999
NDUFA6	NDUFS8	9606.ENSP00000418842	9606.ENSP00000315774	0.889	0.998	0.9	0.999
NDUFA6	NDUFV1	9606.ENSP00000418842	9606.ENSP00000322450	0.539	0.998	0.9	0.999
NDUFA6	NDUFAF3	9606.ENSP00000418842	9606.ENSP00000323076	0.087	0	0.6	0.619
NDUFA6	NDUFV2	9606.ENSP00000418842	9606.ENSP00000327268	0.918	0.998	0.9	0.999
NDUFA6	NDUFB1	9606.ENSP00000418842	9606.ENSP00000330787	0.492	0.985	0.9	0.999
NDUFA6	NDUFV3	9606.ENSP00000418842	9606.ENSP00000346196	0.179	0.982	0.9	0.998
NDUFA6	NDUFS2	9606.ENSP00000418842	9606.ENSP00000356972	0.288	0.995	0.9	0.999
NDUFA6	NDUFAF4	9606.ENSP00000418842	9606.ENSP00000358272	0.147	0	0.6	0.644
NDUFA6	NDUFS5	9606.ENSP00000418842	9606.ENSP00000362060	0.784	0.998	0.9	0.999
NDUFA6	NDUFA8	9606.ENSP00000418842	9606.ENSP00000362873	0.903	0.995	0.9	0.999
NDUFA6	NDUFB6	9606.ENSP00000418842	9606.ENSP00000369176	0.915	0.994	0.9	0.999
NDUFA6	NDUFS1	9606.ENSP00000418842	9606.ENSP00000392709	0.384	0.998	0.9	0.999

NDUFA6	NDUFC1	9606.ENSP00000418842	9606.ENSP00000441126	0.692	0.868	0.9	0.995
NDUFA6	NDUFAB1	9606.ENSP00000418842	9606.ENSP00000458770	0.885	0.998	0.9	0.999
NDUFA6	NDUFB2	9606.ENSP00000418842	9606.ENSP00000419087	0.869	0.988	0.9	0.999
NDUFA7	NDUFB4	9606.ENSP00000301457	9606.ENSP00000184266	0.411	0.987	0.9	0.999
NDUFA7	NDUFB7	9606.ENSP00000301457	9606.ENSP00000215565	0.912	0.987	0.9	0.999
NDUFA7	NDUFS7	9606.ENSP00000301457	9606.ENSP00000233627	0.865	0.973	0.9	0.999
NDUFA7	NDUFB3	9606.ENSP00000301457	9606.ENSP00000237889	0.556	0.868	0.9	0.993
NDUFA7	NDUFB5	9606.ENSP00000301457	9606.ENSP00000259037	0.344	0.986	0.9	0.999
NDUFA7	NDUFAF1	9606.ENSP00000301457	9606.ENSP00000260361	0.065	0	0.9	0.902
NDUFA7	NDUFS3	9606.ENSP00000301457	9606.ENSP00000263774	0.842	0.998	0.9	0.999
NDUFA7	NDUFA9	9606.ENSP00000301457	9606.ENSP00000266544	0.473	0.988	0.9	0.999
NDUFA7	NDUFB10	9606.ENSP00000301457	9606.ENSP00000268668	0.927	0.984	0.9	0.999
NDUFA7	NDUFS6	9606.ENSP00000301457	9606.ENSP00000274137	0.858	0.998	0.9	0.999
NDUFA7	NDUFB11	9606.ENSP00000301457	9606.ENSP00000276062	0.861	0.875	0.9	0.998
NDUFA7	NDUFB9	9606.ENSP00000301457	9606.ENSP00000276689	0.784	0.987	0.9	0.999
NDUFA7	NDUFC2	9606.ENSP00000301457	9606.ENSP00000281031	0.692	0.955	0.9	0.998
NDUFA7	NDUFS4	9606.ENSP00000301457	9606.ENSP00000296684	0.843	0.988	0.9	0.999
NDUFA7	NDUFB8	9606.ENSP00000301457	9606.ENSP00000299166	0.873	0.901	0.9	0.998
NDUFA7	NDUFAF4	9606.ENSP00000301457	9606.ENSP00000358272	0.064	0	0.6	0.609
NDUFA7	NDUFAF3	9606.ENSP00000301457	9606.ENSP00000323076	0.246	0	0.6	0.685
NDUFA7	NDUFV3	9606.ENSP00000301457	9606.ENSP00000346196	0.177	0.816	0.9	0.983
NDUFA7	NDUFB1	9606.ENSP00000301457	9606.ENSP00000330787	0.274	0.842	0.9	0.987
NDUFA7	NDUFC1	9606.ENSP00000301457	9606.ENSP00000441126	0.388	0.868	0.9	0.991
NDUFA7	NDUFB2	9606.ENSP00000301457	9606.ENSP00000419087	0.234	0.901	0.9	0.991
NDUFA7	NDUFS5	9606.ENSP00000301457	9606.ENSP00000362060	0.674	0.993	0.9	0.999
NDUFA7	NDUFAB1	9606.ENSP00000301457	9606.ENSP00000458770	0.83	0.987	0.9	0.999
NDUFA7	NDUFV2	9606.ENSP00000301457	9606.ENSP00000327268	0.425	0.986	0.9	0.999
NDUFA7	NDUFS1	9606.ENSP00000301457	9606.ENSP00000392709	0.321	0.986	0.9	0.998
NDUFA7	NDUFA8	9606.ENSP00000301457	9606.ENSP00000362873	0.881	0.972	0.9	0.999
NDUFA7	NDUFS2	9606.ENSP00000301457	9606.ENSP00000356972	0.286	0.986	0.9	0.998
NDUFA7	NDUFS8	9606.ENSP00000301457	9606.ENSP00000315774	0.875	0.842	0.9	0.997
NDUFA7	NDUFB6	9606.ENSP00000301457	9606.ENSP00000369176	0.635	0.981	0.9	0.999
NDUFA7	NDUFV1	9606.ENSP00000301457	9606.ENSP00000322450	0.436	0.986	0.9	0.999
NDUFA8	NDUFB4	9606.ENSP00000362873	9606.ENSP00000184266	0.412	0.987	0.9	0.999
NDUFA8	NDUFB7	9606.ENSP00000362873	9606.ENSP00000215565	0.962	0.995	0.9	0.999
NDUFA8	NDUFS7	9606.ENSP00000362873	9606.ENSP00000233627	0.931	0.995	0.9	0.999
NDUFA8	NDUFB3	9606.ENSP00000362873	9606.ENSP00000237889	0.655	0.992	0.9	0.999
NDUFA8	NDUFB5	9606.ENSP00000362873	9606.ENSP00000259037	0.908	0.975	0.9	0.999
NDUFA8	NDUFAF1	9606.ENSP00000362873	9606.ENSP00000260361	0.227	0.482	0.9	0.956
NDUFA8	NDUFS3	9606.ENSP00000362873	9606.ENSP00000263774	0.956	0.994	0.9	0.999
NDUFA8	NDUFA9	9606.ENSP00000362873	9606.ENSP00000266544	0.879	0.995	0.9	0.999
NDUFA8	NDUFB10	9606.ENSP00000362873	9606.ENSP00000268668	0.959	0.988	0.9	0.999
NDUFA8	NDUFS6	9606.ENSP00000362873	9606.ENSP00000274137	0.9	0.994	0.9	0.999
NDUFA8	NDUFB11	9606.ENSP00000362873	9606.ENSP00000276062	0.908	0.877	0.9	0.998

NDUFA8	NDUFB9	9606.ENSP00000362873	9606.ENSP00000276689	0.953	0.992	0.9	0.999
NDUFA8	NDUFC2	9606.ENSP00000362873	9606.ENSP00000281031	0.869	0.981	0.9	0.999
NDUFA8	NDUFS4	9606.ENSP00000362873	9606.ENSP00000296684	0.905	0.992	0.9	0.999
NDUFA8	NDUFB8	9606.ENSP00000362873	9606.ENSP00000299166	0.952	0.934	0.9	0.999
NDUFA8	NDUFS8	9606.ENSP00000362873	9606.ENSP00000315774	0.909	0.995	0.9	0.999
NDUFA8	NDUFV1	9606.ENSP00000362873	9606.ENSP00000322450	0.742	0.994	0.9	0.999
NDUFA8	NDUFAF3	9606.ENSP00000362873	9606.ENSP00000323076	0.198	0.913	0.6	0.969
NDUFA8	NDUFV2	9606.ENSP00000362873	9606.ENSP00000327268	0.931	0.993	0.9	0.999
NDUFA8	NDUFB1	9606.ENSP00000362873	9606.ENSP00000330787	0.315	0.957	0.9	0.996
NDUFA8	NDUFV3	9606.ENSP00000362873	9606.ENSP00000346196	0.169	0.89	0.9	0.99
NDUFA8	NDUFS2	9606.ENSP00000362873	9606.ENSP00000356972	0.918	0.986	0.9	0.999
NDUFA8	NDUFAF4	9606.ENSP00000362873	9606.ENSP00000358272	0.117	0.292	0.6	0.728
NDUFA8	NDUFS5	9606.ENSP00000362873	9606.ENSP00000362060	0.764	0.986	0.9	0.999
NDUFA8	NDUFC1	9606.ENSP00000362873	9606.ENSP00000441126	0.419	0.868	0.9	0.991
NDUFA8	NDUFB2	9606.ENSP00000362873	9606.ENSP00000419087	0.406	0.901	0.9	0.993
NDUFA8	NDUFS1	9606.ENSP00000362873	9606.ENSP00000392709	0.413	0.992	0.9	0.999
NDUFA8	NDUFB6	9606.ENSP00000362873	9606.ENSP00000369176	0.945	0.965	0.9	0.999
NDUFA8	NDUFAB1	9606.ENSP00000362873	9606.ENSP00000458770	0.861	0.987	0.9	0.999
NDUFA9	NDUFB4	9606.ENSP00000266544	9606.ENSP00000184266	0.376	0.988	0.9	0.999
NDUFA9	NDUFB7	9606.ENSP00000266544	9606.ENSP00000215565	0.771	0.995	0.9	0.999
NDUFA9	NDUFS7	9606.ENSP00000266544	9606.ENSP00000233627	0.882	0.999	0.9	0.999
NDUFA9	NDUFB3	9606.ENSP00000266544	9606.ENSP00000237889	0.484	0.987	0.9	0.999
NDUFA9	NDUFB5	9606.ENSP00000266544	9606.ENSP00000259037	0.376	0.99	0.9	0.999
NDUFA9	NDUFAF1	9606.ENSP00000266544	9606.ENSP00000260361	0.115	0.27	0.9	0.929
NDUFA9	NDUFS3	9606.ENSP00000266544	9606.ENSP00000263774	0.942	0.999	0.9	0.999
NDUFA9	NDUFAF3	9606.ENSP00000266544	9606.ENSP00000323076	0.1	0	0.6	0.624
NDUFA9	NDUFAF4	9606.ENSP00000266544	9606.ENSP00000358272	0.115	0	0.6	0.631
NDUFA9	NDUFC1	9606.ENSP00000266544	9606.ENSP00000441126	0.142	0.844	0.9	0.985
NDUFA9	NDUFB2	9606.ENSP00000266544	9606.ENSP00000419087	0.181	0.901	0.9	0.991
NDUFA9	NDUFC2	9606.ENSP00000266544	9606.ENSP00000281031	0.455	0.91	0.9	0.994
NDUFA9	NDUFB1	9606.ENSP00000266544	9606.ENSP00000330787	0.236	0.967	0.9	0.997
NDUFA9	NDUFS6	9606.ENSP00000266544	9606.ENSP00000274137	0.469	0.999	0.9	0.999
NDUFA9	NDUFS1	9606.ENSP00000266544	9606.ENSP00000392709	0.886	0.999	0.9	0.999
NDUFA9	NDUFV3	9606.ENSP00000266544	9606.ENSP00000346196	0.122	0.981	0.9	0.998
NDUFA9	NDUFS4	9606.ENSP00000266544	9606.ENSP00000296684	0.866	0.996	0.9	0.999
NDUFA9	NDUFV2	9606.ENSP00000266544	9606.ENSP00000327268	0.883	0.999	0.9	0.999
NDUFA9	NDUFB11	9606.ENSP00000266544	9606.ENSP00000276062	0.438	0.997	0.9	0.999
NDUFA9	NDUFB9	9606.ENSP00000266544	9606.ENSP00000276689	0.903	0.995	0.9	0.999
NDUFA9	NDUFAB1	9606.ENSP00000266544	9606.ENSP00000458770	0.601	0.998	0.9	0.999
NDUFA9	NDUFS5	9606.ENSP00000266544	9606.ENSP00000362060	0.559	0.998	0.9	0.999
NDUFA9	NDUFV1	9606.ENSP00000266544	9606.ENSP00000322450	0.926	0.999	0.9	0.999
NDUFA9	NDUFB6	9606.ENSP00000266544	9606.ENSP00000369176	0.8	0.94	0.9	0.998
NDUFA9	NDUFB10	9606.ENSP00000266544	9606.ENSP00000268668	0.85	0.998	0.9	0.999
NDUFA9	NDUFS8	9606.ENSP00000266544	9606.ENSP00000315774	0.755	0.999	0.9	0.999

NDUFA9	NDUFS2	9606.ENSP00000266544	9606.ENSP00000356972	0.866	0.999	0.9	0.999
NDUFA9	NDUFB8	9606.ENSP00000266544	9606.ENSP00000299166	0.774	0.998	0.9	0.999
NDUFAB1	NDUFB4	9606.ENSP00000458770	9606.ENSP00000184266	0.436	0.987	0.9	0.999
NDUFAB1	NDUFB7	9606.ENSP00000458770	9606.ENSP00000215565	0.84	0.987	0.9	0.999
NDUFAB1	NDUFS7	9606.ENSP00000458770	9606.ENSP00000233627	0.751	0.987	0.9	0.999
NDUFAB1	NDUFB3	9606.ENSP00000458770	9606.ENSP00000237889	0.706	0.987	0.9	0.999
NDUFAB1	NDUFB5	9606.ENSP00000458770	9606.ENSP00000259037	0.875	0.987	0.9	0.999
NDUFAB1	NDUFAF1	9606.ENSP00000458770	9606.ENSP00000260361	0.11	0	0.9	0.907
NDUFAB1	NDUFS3	9606.ENSP00000458770	9606.ENSP00000263774	0.881	0.988	0.9	0.999
NDUFAB1	NDUFB10	9606.ENSP00000458770	9606.ENSP00000268668	0.929	0.987	0.9	0.999
NDUFAB1	NDUFS6	9606.ENSP00000458770	9606.ENSP00000274137	0.878	0.988	0.9	0.999
NDUFAB1	NDUFB11	9606.ENSP00000458770	9606.ENSP00000276062	0.689	0.941	0.9	0.998
NDUFAB1	NDUFB9	9606.ENSP00000458770	9606.ENSP00000276689	0.895	0.987	0.9	0.999
NDUFAB1	NDUFC2	9606.ENSP00000458770	9606.ENSP00000281031	0.792	0.987	0.9	0.999
NDUFAB1	NDUFS4	9606.ENSP00000458770	9606.ENSP00000296684	0.902	0.987	0.9	0.999
NDUFAB1	NDUFB8	9606.ENSP00000458770	9606.ENSP00000299166	0.852	0.987	0.9	0.999
NDUFAB1	NDUFS8	9606.ENSP00000458770	9606.ENSP00000315774	0.483	0.987	0.9	0.999
NDUFAB1	NDUFV1	9606.ENSP00000458770	9606.ENSP00000322450	0.13	0.987	0.9	0.998
NDUFAB1	NDUFAF3	9606.ENSP00000458770	9606.ENSP00000323076	0.103	0	0.6	0.625
NDUFAB1	NDUFV2	9606.ENSP00000458770	9606.ENSP00000327268	0.741	0.987	0.9	0.999
NDUFAB1	NDUFB1	9606.ENSP00000458770	9606.ENSP00000330787	0.315	0.987	0.9	0.999
NDUFAB1	NDUFV3	9606.ENSP00000458770	9606.ENSP00000346196	0.089	0.987	0.9	0.998
NDUFAB1	NDUFS2	9606.ENSP00000458770	9606.ENSP00000356972	0.377	0.987	0.9	0.999
NDUFAB1	NDUFAF4	9606.ENSP00000458770	9606.ENSP00000358272	0.202	0	0.6	0.667
NDUFAB1	NDUFS5	9606.ENSP00000458770	9606.ENSP00000362060	0.614	0.987	0.9	0.999
NDUFAB1	NDUFB6	9606.ENSP00000458770	9606.ENSP00000369176	0.851	0.987	0.9	0.999
NDUFAB1	NDUFS1	9606.ENSP00000458770	9606.ENSP00000392709	0.269	0.987	0.9	0.999
NDUFAB1	NDUFB2	9606.ENSP00000458770	9606.ENSP00000419087	0.284	0.987	0.9	0.999
NDUFAB1	NDUFC1	9606.ENSP00000458770	9606.ENSP00000441126	0.704	0.987	0.9	0.999
NDUFAF1	NDUFB4	9606.ENSP00000260361	9606.ENSP00000184266	0.064	0	0.9	0.902
NDUFAF1	NDUFB7	9606.ENSP00000260361	9606.ENSP00000215565	0.254	0	0.9	0.922
NDUFAF1	NDUFS7	9606.ENSP00000260361	9606.ENSP00000233627	0.117	0.664	0.9	0.967
NDUFAF1	NDUFB3	9606.ENSP00000260361	9606.ENSP00000237889	0.12	0	0.9	0.908
NDUFAF1	NDUFB5	9606.ENSP00000260361	9606.ENSP00000259037	0.142	0.48	0.9	0.951
NDUFAF1	NDUFAF4	9606.ENSP00000260361	9606.ENSP00000358272	0.069	0.201	0.6	0.676
NDUFAF1	NDUFB1	9606.ENSP00000260361	9606.ENSP00000330787	0.042	0	0.9	0.9
NDUFAF1	NDUFB2	9606.ENSP00000260361	9606.ENSP00000419087	0.063	0	0.9	0.902
NDUFAF1	NDUFC1	9606.ENSP00000260361	9606.ENSP00000441126	0.064	0	0.9	0.902
NDUFAF1	NDUFB8	9606.ENSP00000260361	9606.ENSP00000299166	0.069	0	0.9	0.903
NDUFAF1	NDUFV3	9606.ENSP00000260361	9606.ENSP00000346196	0.083	0	0.9	0.904
NDUFAF1	NDUFC2	9606.ENSP00000260361	9606.ENSP00000281031	0.084	0	0.9	0.904
NDUFAF1	NDUFB9	9606.ENSP00000260361	9606.ENSP00000276689	0.173	0	0.9	0.913
NDUFAF1	NDUFS4	9606.ENSP00000260361	9606.ENSP00000296684	0.122	0	0.9	0.908
NDUFAF1	NDUFV1	9606.ENSP00000260361	9606.ENSP00000322450	0.124	0	0.9	0.908

NDUFAF1	NDUFB6	9606.ENSP00000260361	9606.ENSP00000369176	0.139	0.27	0.9	0.931
NDUFAF1	NDUFS8	9606.ENSP00000260361	9606.ENSP00000315774	0.086	0	0.9	0.904
NDUFAF1	NDUFS6	9606.ENSP00000260361	9606.ENSP00000274137	0.093	0	0.9	0.905
NDUFAF1	NDUFB10	9606.ENSP00000260361	9606.ENSP00000268668	0.163	0	0.9	0.912
NDUFAF1	NDUFV2	9606.ENSP00000260361	9606.ENSP00000327268	0.456	0	0.9	0.943
NDUFAF1	NDUFS2	9606.ENSP00000260361	9606.ENSP00000356972	0.214	0	0.9	0.918
NDUFAF1	NDUFAF3	9606.ENSP00000260361	9606.ENSP00000323076	0.092	0.913	0.6	0.965
NDUFAF1	NDUFS1	9606.ENSP00000260361	9606.ENSP00000392709	0.136	0.913	0.9	0.991
NDUFAF1	NDUFS3	9606.ENSP00000260361	9606.ENSP00000263774	0.099	0.992	0.9	0.999
NDUFAF1	NDUFB11	9606.ENSP00000260361	9606.ENSP00000276062	0.07	0.992	0.9	0.999
NDUFAF1	NDUFS5	9606.ENSP00000260361	9606.ENSP00000362060	0.07	0.992	0.9	0.999
NDUFAF2	NDUFS6	9606.ENSP00000296597	9606.ENSP00000274137	0.14	0	0.8	0.82
NDUFAF2	NDUFV2	9606.ENSP00000296597	9606.ENSP00000327268	0.149	0	0.6	0.645
NDUFAF2	NDUFV1	9606.ENSP00000296597	9606.ENSP00000322450	0.085	0	0.6	0.618
NDUFAF2	NDUFV3	9606.ENSP00000296597	9606.ENSP00000346196	0.048	0	0.8	0.801
NDUFAF2	NDUFS1	9606.ENSP00000296597	9606.ENSP00000392709	0.088	0	0.6	0.619
NDUFAF2	NDUFS4	9606.ENSP00000296597	9606.ENSP00000296684	0.246	0	0.8	0.842
NDUFAF3	NDUFB4	9606.ENSP00000323076	9606.ENSP00000184266	0.143	0	0.6	0.642
NDUFAF3	NDUFB7	9606.ENSP00000323076	9606.ENSP00000215565	0.409	0	0.6	0.753
NDUFAF3	NDUFS7	9606.ENSP00000323076	9606.ENSP00000233627	0.303	0.913	0.6	0.973
NDUFAF3	NDUFB3	9606.ENSP00000323076	9606.ENSP00000237889	0.096	0	0.6	0.623
NDUFAF3	NDUFB5	9606.ENSP00000323076	9606.ENSP00000259037	0.064	0	0.6	0.609
NDUFAF3	NDUFS3	9606.ENSP00000323076	9606.ENSP00000263774	0.195	0.992	0.6	0.997
NDUFAF3	NDUFB10	9606.ENSP00000323076	9606.ENSP00000268668	0.186	0.913	0.6	0.969
NDUFAF3	NDUFB11	9606.ENSP00000323076	9606.ENSP00000276062	0.178	0.271	0.6	0.739
NDUFAF3	NDUFB9	9606.ENSP00000323076	9606.ENSP00000276689	0.144	0	0.6	0.643
NDUFAF3	NDUFC2	9606.ENSP00000323076	9606.ENSP00000281031	0.127	0	0.6	0.636
NDUFAF3	NDUFB8	9606.ENSP00000323076	9606.ENSP00000299166	0.104	0	0.6	0.626
NDUFAF3	NDUFS8	9606.ENSP00000323076	9606.ENSP00000315774	0.343	0.992	0.6	0.997
NDUFAF3	NDUFB2	9606.ENSP00000323076	9606.ENSP00000419087	0.065	0	0.6	0.61
NDUFAF3	NDUFB1	9606.ENSP00000323076	9606.ENSP00000330787	0.08	0	0.6	0.616
NDUFAF3	NDUFC1	9606.ENSP00000323076	9606.ENSP00000441126	0.087	0	0.6	0.619
NDUFAF3	NDUFB6	9606.ENSP00000323076	9606.ENSP00000369176	0.191	0	0.6	0.662
NDUFAF3	NDUFS5	9606.ENSP00000323076	9606.ENSP00000362060	0.108	0.913	0	0.919
NDUFAF3	NDUFS2	9606.ENSP00000323076	9606.ENSP00000356972	0.116	0.992	0.6	0.996
NDUFAF3	NDUFAF4	9606.ENSP00000323076	9606.ENSP00000358272	0.104	0.992	0.8	0.998
NDUFAF4	NDUFB4	9606.ENSP00000358272	9606.ENSP00000184266	0.133	0	0.6	0.638
NDUFAF4	NDUFB7	9606.ENSP00000358272	9606.ENSP00000215565	0.07	0	0.6	0.612
NDUFAF4	NDUFS7	9606.ENSP00000358272	9606.ENSP00000233627	0.062	0.992	0.6	0.996
NDUFAF4	NDUFB3	9606.ENSP00000358272	9606.ENSP00000237889	0.101	0	0.6	0.625
NDUFAF4	NDUFB5	9606.ENSP00000358272	9606.ENSP00000259037	0.091	0.2	0.6	0.683
NDUFAF4	NDUFS3	9606.ENSP00000358272	9606.ENSP00000263774	0.083	0.768	0.6	0.907
NDUFAF4	NDUFB10	9606.ENSP00000358272	9606.ENSP00000268668	0.088	0.2	0.6	0.682
NDUFAF4	NDUFB11	9606.ENSP00000358272	9606.ENSP00000276062	0.08	0.263	0.6	0.705

NDUFAF4	NDUFB9	9606.ENSP00000358272	9606.ENSP00000276689	0.088	0	0.6	0.619
NDUFAF4	NDUFC2	9606.ENSP00000358272	9606.ENSP00000281031	0.065	0.263	0.6	0.7
NDUFAF4	NDUFB8	9606.ENSP00000358272	9606.ENSP00000299166	0.087	0	0.6	0.619
NDUFAF4	NDUFS8	9606.ENSP00000358272	9606.ENSP00000315774	0.063	0.274	0.6	0.704
NDUFAF4	NDUFB1	9606.ENSP00000358272	9606.ENSP00000330787	0.093	0	0.6	0.622
NDUFAF4	NDUFS2	9606.ENSP00000358272	9606.ENSP00000356972	0.069	0.294	0.6	0.714
NDUFAF4	NDUFB2	9606.ENSP00000358272	9606.ENSP00000419087	0.062	0	0.6	0.608
NDUFAF4	NDUFC1	9606.ENSP00000358272	9606.ENSP00000441126	0.186	0	0.6	0.66
NDUFAF4	NDUFB6	9606.ENSP00000358272	9606.ENSP00000369176	0.152	0	0.6	0.646
NDUFAF4	NDUFS5	9606.ENSP00000358272	9606.ENSP00000362060	0.102	0.992	0	0.992
NDUFB1	NDUFB4	9606.ENSP00000330787	9606.ENSP00000184266	0.865	0.872	0.9	0.998
NDUFB1	NDUFB7	9606.ENSP00000330787	9606.ENSP00000215565	0.334	0.842	0.9	0.988
NDUFB1	NDUFS7	9606.ENSP00000330787	9606.ENSP00000233627	0.288	0.842	0.9	0.987
NDUFB1	NDUFB3	9606.ENSP00000330787	9606.ENSP00000237889	0.552	0.822	0.9	0.991
NDUFB1	NDUFB5	9606.ENSP00000330787	9606.ENSP00000259037	0.287	0.936	0.9	0.995
NDUFB1	NDUFS3	9606.ENSP00000330787	9606.ENSP00000263774	0.247	0.967	0.9	0.997
NDUFB1	NDUFB10	9606.ENSP00000330787	9606.ENSP00000268668	0.203	0.838	0.9	0.986
NDUFB1	NDUFS6	9606.ENSP00000330787	9606.ENSP00000274137	0.284	0.914	0.9	0.993
NDUFB1	NDUFB11	9606.ENSP00000330787	9606.ENSP00000276062	0.145	0.782	0.9	0.979
NDUFB1	NDUFB9	9606.ENSP00000330787	9606.ENSP00000276689	0.356	0.857	0.9	0.989
NDUFB1	NDUFC2	9606.ENSP00000330787	9606.ENSP00000281031	0.372	0.836	0.9	0.988
NDUFB1	NDUFS4	9606.ENSP00000330787	9606.ENSP00000296684	0.442	0.821	0.9	0.989
NDUFB1	NDUFB8	9606.ENSP00000330787	9606.ENSP00000299166	0.363	0.823	0.9	0.987
NDUFB1	NDUFS8	9606.ENSP00000330787	9606.ENSP00000315774	0.279	0.821	0.9	0.985
NDUFB1	NDUFV1	9606.ENSP00000330787	9606.ENSP00000322450	0.294	0.842	0.9	0.987
NDUFB1	NDUFV2	9606.ENSP00000330787	9606.ENSP00000327268	0.364	0.842	0.9	0.989
NDUFB1	NDUFV3	9606.ENSP00000330787	9606.ENSP00000346196	0.08	0.8	0.9	0.98
NDUFB1	NDUFS2	9606.ENSP00000330787	9606.ENSP00000356972	0.129	0.842	0.9	0.985
NDUFB1	NDUFB2	9606.ENSP00000330787	9606.ENSP00000419087	0.16	0.851	0.9	0.986
NDUFB1	NDUFS1	9606.ENSP00000330787	9606.ENSP00000392709	0.269	0.842	0.9	0.987
NDUFB1	NDUFS5	9606.ENSP00000330787	9606.ENSP00000362060	0.436	0.842	0.9	0.99
NDUFB1	NDUFB6	9606.ENSP00000330787	9606.ENSP00000369176	0.482	0.823	0.9	0.99
NDUFB1	NDUFC1	9606.ENSP00000330787	9606.ENSP00000441126	0.822	0.842	0.9	0.996
NDUFB10	NDUFB4	9606.ENSP00000268668	9606.ENSP00000184266	0.481	0.969	0.9	0.998
NDUFB10	NDUFB7	9606.ENSP00000268668	9606.ENSP00000215565	0.944	0.991	0.9	0.999
NDUFB10	NDUFS7	9606.ENSP00000268668	9606.ENSP00000233627	0.864	0.994	0.9	0.999
NDUFB10	NDUFB3	9606.ENSP00000268668	9606.ENSP00000237889	0.664	0.976	0.9	0.999
NDUFB10	NDUFB5	9606.ENSP00000268668	9606.ENSP00000259037	0.926	0.967	0.9	0.999
NDUFB10	NDUFS3	9606.ENSP00000268668	9606.ENSP00000263774	0.944	0.998	0.9	0.999
NDUFB10	NDUFV3	9606.ENSP00000268668	9606.ENSP00000346196	0.189	0.814	0.9	0.983
NDUFB10	NDUFC1	9606.ENSP00000268668	9606.ENSP00000441126	0.66	0.844	0.9	0.994
NDUFB10	NDUFB2	9606.ENSP00000268668	9606.ENSP00000419087	0.519	0.901	0.9	0.994
NDUFB10	NDUFB11	9606.ENSP00000268668	9606.ENSP00000276062	0.93	0.882	0.9	0.999
NDUFB10	NDUFB9	9606.ENSP00000268668	9606.ENSP00000276689	0.948	0.99	0.9	0.999

NDUFB10	NDUFS5	9606.ENSP00000268668	9606.ENSP00000362060	0.878	0.998	0.9	0.999
NDUFB10	NDUFS1	9606.ENSP00000268668	9606.ENSP00000392709	0.329	0.993	0.9	0.999
NDUFB10	NDUFS6	9606.ENSP00000268668	9606.ENSP00000274137	0.929	0.992	0.9	0.999
NDUFB10	NDUFV2	9606.ENSP00000268668	9606.ENSP00000327268	0.896	0.99	0.9	0.999
NDUFB10	NDUFC2	9606.ENSP00000268668	9606.ENSP00000281031	0.848	0.95	0.9	0.999
NDUFB10	NDUFS4	9606.ENSP00000268668	9606.ENSP00000296684	0.881	0.998	0.9	0.999
NDUFB10	NDUFS8	9606.ENSP00000268668	9606.ENSP00000315774	0.934	0.95	0.9	0.999
NDUFB10	NDUFB8	9606.ENSP00000268668	9606.ENSP00000299166	0.932	0.901	0.9	0.999
NDUFB10	NDUFS2	9606.ENSP00000268668	9606.ENSP00000356972	0.28	0.987	0.9	0.999
NDUFB10	NDUFV1	9606.ENSP00000268668	9606.ENSP00000322450	0.512	0.99	0.9	0.999
NDUFB10	NDUFB6	9606.ENSP00000268668	9606.ENSP00000369176	0.911	0.985	0.9	0.999
NDUFB11	NDUFB4	9606.ENSP00000276062	9606.ENSP00000184266	0.193	0.884	0.9	0.989
NDUFB11	NDUFB7	9606.ENSP00000276062	9606.ENSP00000215565	0.935	0.909	0.9	0.999
NDUFB11	NDUFS7	9606.ENSP00000276062	9606.ENSP00000233627	0.823	0.885	0.9	0.997
NDUFB11	NDUFB3	9606.ENSP00000276062	9606.ENSP00000237889	0.334	0.857	0.9	0.989
NDUFB11	NDUFB5	9606.ENSP00000276062	9606.ENSP00000259037	0.832	0.884	0.9	0.997
NDUFB11	NDUFS3	9606.ENSP00000276062	9606.ENSP00000263774	0.896	0.996	0.9	0.999
NDUFB11	NDUFS6	9606.ENSP00000276062	9606.ENSP00000274137	0.508	0.854	0.9	0.992
NDUFB11	NDUFV3	9606.ENSP00000276062	9606.ENSP00000346196	0.07	0.437	0.9	0.943
NDUFB11	NDUFC1	9606.ENSP00000276062	9606.ENSP00000441126	0.177	0.788	0.9	0.981
NDUFB11	NDUFB2	9606.ENSP00000276062	9606.ENSP00000419087	0.28	0.789	0.9	0.983
NDUFB11	NDUFS2	9606.ENSP00000276062	9606.ENSP00000356972	0.238	0.884	0.9	0.99
NDUFB11	NDUFS1	9606.ENSP00000276062	9606.ENSP00000392709	0.125	0.948	0.9	0.995
NDUFB11	NDUFC2	9606.ENSP00000276062	9606.ENSP00000281031	0.695	0.843	0.9	0.994
NDUFB11	NDUFV1	9606.ENSP00000276062	9606.ENSP00000322450	0.575	0.909	0.9	0.995
NDUFB11	NDUFV2	9606.ENSP00000276062	9606.ENSP00000327268	0.704	0.884	0.9	0.996
NDUFB11	NDUFS5	9606.ENSP00000276062	9606.ENSP00000362060	0.323	0.997	0.9	0.999
NDUFB11	NDUFB9	9606.ENSP00000276062	9606.ENSP00000276689	0.867	0.959	0.9	0.999
NDUFB11	NDUFS4	9606.ENSP00000276062	9606.ENSP00000296684	0.876	0.849	0.9	0.997
NDUFB11	NDUFS8	9606.ENSP00000276062	9606.ENSP00000315774	0.846	0.915	0.9	0.998
NDUFB11	NDUFB8	9606.ENSP00000276062	9606.ENSP00000299166	0.853	0.887	0.9	0.998
NDUFB11	NDUFB6	9606.ENSP00000276062	9606.ENSP00000369176	0.859	0.869	0.9	0.998
NDUFB2	NDUFB4	9606.ENSP00000419087	9606.ENSP00000184266	0.698	0.901	0.9	0.996
NDUFB2	NDUFB7	9606.ENSP00000419087	9606.ENSP00000215565	0.573	0.885	0.9	0.994
NDUFB2	NDUFS7	9606.ENSP00000419087	9606.ENSP00000233627	0.221	0.901	0.9	0.991
NDUFB2	NDUFB3	9606.ENSP00000419087	9606.ENSP00000237889	0.5	0.868	0.9	0.992
NDUFB2	NDUFB5	9606.ENSP00000419087	9606.ENSP00000259037	0.691	0.901	0.9	0.996
NDUFB2	NDUFS3	9606.ENSP00000419087	9606.ENSP00000263774	0.331	0.901	0.9	0.992
NDUFB2	NDUFS6	9606.ENSP00000419087	9606.ENSP00000274137	0.442	0.844	0.9	0.99
NDUFB2	NDUFB9	9606.ENSP00000419087	9606.ENSP00000276689	0.66	0.901	0.9	0.996
NDUFB2	NDUFC2	9606.ENSP00000419087	9606.ENSP00000281031	0.548	0.867	0.9	0.993
NDUFB2	NDUFS4	9606.ENSP00000419087	9606.ENSP00000296684	0.327	0.844	0.9	0.988
NDUFB2	NDUFB8	9606.ENSP00000419087	9606.ENSP00000299166	0.605	0.901	0.9	0.995
NDUFB2	NDUFS8	9606.ENSP00000419087	9606.ENSP00000315774	0.388	0.821	0.9	0.988

NDUFB2	NDUFV1	9606.ENSP00000419087	9606.ENSP00000322450	0.103	0.901	0.9	0.99
NDUFB2	NDUFV2	9606.ENSP00000419087	9606.ENSP00000327268	0.467	0.901	0.9	0.994
NDUFB2	NDUFV3	9606.ENSP00000419087	9606.ENSP00000346196	0.066	0.8	0.9	0.979
NDUFB2	NDUFS2	9606.ENSP00000419087	9606.ENSP00000356972	0.091	0.901	0.9	0.99
NDUFB2	NDUFS5	9606.ENSP00000419087	9606.ENSP00000362060	0.869	0.901	0.9	0.998
NDUFB2	NDUFB6	9606.ENSP00000419087	9606.ENSP00000369176	0.72	0.901	0.9	0.996
NDUFB2	NDUFS1	9606.ENSP00000419087	9606.ENSP00000392709	0.065	0.901	0.9	0.989
NDUFB2	NDUFC1	9606.ENSP00000419087	9606.ENSP00000441126	0.679	0.844	0.9	0.994
NDUFB3	NDUFB4	9606.ENSP00000237889	9606.ENSP00000184266	0.889	0.868	0.9	0.998
NDUFB3	NDUFB7	9606.ENSP00000237889	9606.ENSP00000215565	0.821	0.946	0.9	0.998
NDUFB3	NDUFS7	9606.ENSP00000237889	9606.ENSP00000233627	0.66	0.957	0.9	0.998
NDUFB3	NDUFV3	9606.ENSP00000237889	9606.ENSP00000346196	0.144	0.8	0.9	0.981
NDUFB3	NDUFS8	9606.ENSP00000237889	9606.ENSP00000315774	0.67	0.82	0.9	0.993
NDUFB3	NDUFS2	9606.ENSP00000237889	9606.ENSP00000356972	0.246	0.868	0.9	0.989
NDUFB3	NDUFS1	9606.ENSP00000237889	9606.ENSP00000392709	0.441	0.868	0.9	0.992
NDUFB3	NDUFV1	9606.ENSP00000237889	9606.ENSP00000322450	0.578	0.868	0.9	0.993
NDUFB3	NDUFB9	9606.ENSP00000237889	9606.ENSP00000276689	0.852	0.868	0.9	0.997
NDUFB3	NDUFB8	9606.ENSP00000237889	9606.ENSP00000299166	0.678	0.868	0.9	0.995
NDUFB3	NDUFS3	9606.ENSP00000237889	9606.ENSP00000263774	0.55	0.955	0.9	0.997
NDUFB3	NDUFC1	9606.ENSP00000237889	9606.ENSP00000441126	0.687	0.842	0.9	0.994
NDUFB3	NDUFS6	9606.ENSP00000237889	9606.ENSP00000274137	0.681	0.9	0.9	0.996
NDUFB3	NDUFV2	9606.ENSP00000237889	9606.ENSP00000327268	0.725	0.868	0.9	0.996
NDUFB3	NDUFC2	9606.ENSP00000237889	9606.ENSP00000281031	0.756	0.862	0.9	0.996
NDUFB3	NDUFS5	9606.ENSP00000237889	9606.ENSP00000362060	0.945	0.94	0.9	0.999
NDUFB3	NDUFB5	9606.ENSP00000237889	9606.ENSP00000259037	0.832	0.947	0.9	0.999
NDUFB3	NDUFB6	9606.ENSP00000237889	9606.ENSP00000369176	0.924	0.868	0.9	0.998
NDUFB3	NDUFS4	9606.ENSP00000237889	9606.ENSP00000296684	0.917	0.856	0.9	0.998
NDUFB4	NDUFV3	9606.ENSP00000184266	9606.ENSP00000346196	0.106	0.816	0.9	0.982
NDUFB4	NDUFS8	9606.ENSP00000184266	9606.ENSP00000315774	0.283	0.842	0.9	0.987
NDUFB4	NDUFB8	9606.ENSP00000184266	9606.ENSP00000299166	0.585	0.901	0.9	0.995
NDUFB4	NDUFC1	9606.ENSP00000184266	9606.ENSP00000441126	0.892	0.868	0.9	0.998
NDUFB4	NDUFS6	9606.ENSP00000184266	9606.ENSP00000274137	0.44	0.963	0.9	0.997
NDUFB4	NDUFC2	9606.ENSP00000184266	9606.ENSP00000281031	0.501	0.955	0.9	0.997
NDUFB4	NDUFB6	9606.ENSP00000184266	9606.ENSP00000369176	0.777	0.901	0.9	0.997
NDUFB4	NDUFS2	9606.ENSP00000184266	9606.ENSP00000356972	0.175	0.987	0.9	0.998
NDUFB4	NDUFS5	9606.ENSP00000184266	9606.ENSP00000362060	0.692	0.993	0.9	0.999
NDUFB4	NDUFS7	9606.ENSP00000184266	9606.ENSP00000233627	0.361	0.986	0.9	0.999
NDUFB4	NDUFB9	9606.ENSP00000184266	9606.ENSP00000276689	0.756	0.929	0.9	0.998
NDUFB4	NDUFV1	9606.ENSP00000184266	9606.ENSP00000322450	0.241	0.993	0.9	0.999
NDUFB4	NDUFS3	9606.ENSP00000184266	9606.ENSP00000263774	0.295	0.998	0.9	0.999
NDUFB4	NDUFB7	9606.ENSP00000184266	9606.ENSP00000215565	0.399	0.987	0.9	0.999
NDUFB4	NDUFB5	9606.ENSP00000184266	9606.ENSP00000259037	0.547	0.987	0.9	0.999
NDUFB4	NDUFS1	9606.ENSP00000184266	9606.ENSP00000392709	0.179	0.986	0.9	0.998
NDUFB4	NDUFS4	9606.ENSP00000184266	9606.ENSP00000296684	0.619	0.988	0.9	0.999

NDUFB4	NDUFV2	9606.ENSP00000184266	9606.ENSP00000327268	0.406	0.986	0.9	0.999
NDUFB5	NDUFB7	9606.ENSP00000259037	9606.ENSP00000215565	0.872	0.977	0.9	0.999
NDUFB5	NDUFS7	9606.ENSP00000259037	9606.ENSP00000233627	0.681	0.988	0.9	0.999
NDUFB5	NDUFV3	9606.ENSP00000259037	9606.ENSP00000346196	0.195	0.816	0.9	0.983
NDUFB5	NDUFS8	9606.ENSP00000259037	9606.ENSP00000315774	0.865	0.842	0.9	0.997
NDUFB5	NDUFC2	9606.ENSP00000259037	9606.ENSP00000281031	0.84	0.963	0.9	0.999
NDUFB5	NDUFS4	9606.ENSP00000259037	9606.ENSP00000296684	0.942	0.899	0.9	0.999
NDUFB5	NDUFV2	9606.ENSP00000259037	9606.ENSP00000327268	0.906	0.986	0.9	0.999
NDUFB5	NDUFB6	9606.ENSP00000259037	9606.ENSP00000369176	0.888	0.97	0.9	0.999
NDUFB5	NDUFS3	9606.ENSP00000259037	9606.ENSP00000263774	0.852	0.981	0.9	0.999
NDUFB5	NDUFV1	9606.ENSP00000259037	9606.ENSP00000322450	0.276	0.986	0.9	0.998
NDUFB5	NDUFS6	9606.ENSP00000259037	9606.ENSP00000274137	0.89	0.968	0.9	0.999
NDUFB5	NDUFC1	9606.ENSP00000259037	9606.ENSP00000441126	0.827	0.868	0.9	0.997
NDUFB5	NDUFS1	9606.ENSP00000259037	9606.ENSP00000392709	0.828	0.989	0.9	0.999
NDUFB5	NDUFS2	9606.ENSP00000259037	9606.ENSP00000356972	0.821	0.929	0.9	0.998
NDUFB5	NDUFB9	9606.ENSP00000259037	9606.ENSP00000276689	0.9	0.987	0.9	0.999
NDUFB5	NDUFS5	9606.ENSP00000259037	9606.ENSP00000362060	0.748	0.993	0.9	0.999
NDUFB5	NDUFB8	9606.ENSP00000259037	9606.ENSP00000299166	0.897	0.958	0.9	0.999
NDUFB6	NDUFB7	9606.ENSP00000369176	9606.ENSP00000215565	0.904	0.986	0.9	0.999
NDUFB6	NDUFS7	9606.ENSP00000369176	9606.ENSP00000233627	0.618	0.969	0.9	0.998
NDUFB6	NDUFS3	9606.ENSP00000369176	9606.ENSP00000263774	0.89	0.969	0.9	0.999
NDUFB6	NDUFS6	9606.ENSP00000369176	9606.ENSP00000274137	0.622	0.986	0.9	0.999
NDUFB6	NDUFB9	9606.ENSP00000369176	9606.ENSP00000276689	0.933	0.964	0.9	0.999
NDUFB6	NDUFC2	9606.ENSP00000369176	9606.ENSP00000281031	0.867	0.938	0.9	0.999
NDUFB6	NDUFS4	9606.ENSP00000369176	9606.ENSP00000296684	0.94	0.955	0.9	0.999
NDUFB6	NDUFB8	9606.ENSP00000369176	9606.ENSP00000299166	0.931	0.901	0.9	0.999
NDUFB6	NDUFS8	9606.ENSP00000369176	9606.ENSP00000315774	0.825	0.864	0.9	0.997
NDUFB6	NDUFV1	9606.ENSP00000369176	9606.ENSP00000322450	0.428	0.966	0.9	0.997
NDUFB6	NDUFV2	9606.ENSP00000369176	9606.ENSP00000327268	0.805	0.985	0.9	0.999
NDUFB6	NDUFV3	9606.ENSP00000369176	9606.ENSP00000346196	0.22	0.816	0.9	0.984
NDUFB6	NDUFS2	9606.ENSP00000369176	9606.ENSP00000356972	0.241	0.976	0.9	0.998
NDUFB6	NDUFS5	9606.ENSP00000369176	9606.ENSP00000362060	0.777	0.995	0.9	0.999
NDUFB6	NDUFC1	9606.ENSP00000369176	9606.ENSP00000441126	0.779	0.844	0.9	0.996
NDUFB6	NDUFS1	9606.ENSP00000369176	9606.ENSP00000392709	0.351	0.971	0.9	0.997
NDUFB7	NDUFV3	9606.ENSP00000215565	9606.ENSP00000346196	0.198	0.816	0.9	0.984
NDUFB7	NDUFC1	9606.ENSP00000215565	9606.ENSP00000441126	0.65	0.868	0.9	0.994
NDUFB7	NDUFV1	9606.ENSP00000215565	9606.ENSP00000322450	0.902	0.987	0.9	0.999
NDUFB7	NDUFS8	9606.ENSP00000215565	9606.ENSP00000315774	0.942	0.985	0.9	0.999
NDUFB7	NDUFB8	9606.ENSP00000215565	9606.ENSP00000299166	0.947	0.901	0.9	0.999
NDUFB7	NDUFS2	9606.ENSP00000215565	9606.ENSP00000356972	0.703	0.987	0.9	0.999
NDUFB7	NDUFS1	9606.ENSP00000215565	9606.ENSP00000392709	0.365	0.986	0.9	0.999
NDUFB7	NDUFS3	9606.ENSP00000215565	9606.ENSP00000263774	0.913	0.998	0.9	0.999
NDUFB7	NDUFS6	9606.ENSP00000215565	9606.ENSP00000274137	0.945	0.992	0.9	0.999
NDUFB7	NDUFV2	9606.ENSP00000215565	9606.ENSP00000327268	0.931	0.987	0.9	0.999

NDUFB7	NDUFC2	9606.ENSP00000215565	9606.ENSP00000281031	0.925	0.955	0.9	0.999
NDUFB7	NDUFS4	9606.ENSP00000215565	9606.ENSP00000296684	0.966	0.987	0.9	0.999
NDUFB7	NDUFB9	9606.ENSP00000215565	9606.ENSP00000276689	0.969	0.991	0.9	0.999
NDUFB7	NDUFS5	9606.ENSP00000215565	9606.ENSP00000362060	0.947	0.995	0.9	0.999
NDUFB7	NDUFS7	9606.ENSP00000215565	9606.ENSP00000233627	0.945	0.993	0.9	0.999
NDUFB8	NDUFS7	9606.ENSP00000299166	9606.ENSP00000233627	0.916	0.91	0.9	0.999
NDUFB8	NDUFS3	9606.ENSP00000299166	9606.ENSP00000263774	0.879	0.998	0.9	0.999
NDUFB8	NDUFS6	9606.ENSP00000299166	9606.ENSP00000274137	0.897	0.914	0.9	0.999
NDUFB8	NDUFB9	9606.ENSP00000299166	9606.ENSP00000276689	0.955	0.958	0.9	0.999
NDUFB8	NDUFC2	9606.ENSP00000299166	9606.ENSP00000281031	0.827	0.867	0.9	0.997
NDUFB8	NDUFS4	9606.ENSP00000299166	9606.ENSP00000296684	0.933	0.985	0.9	0.999
NDUFB8	NDUFV3	9606.ENSP00000299166	9606.ENSP00000346196	0.12	0.8	0.9	0.98
NDUFB8	NDUFC1	9606.ENSP00000299166	9606.ENSP00000441126	0.221	0.844	0.9	0.986
NDUFB8	NDUFS8	9606.ENSP00000299166	9606.ENSP00000315774	0.525	0.837	0.9	0.991
NDUFB8	NDUFV1	9606.ENSP00000299166	9606.ENSP00000322450	0.663	0.906	0.9	0.996
NDUFB8	NDUFS1	9606.ENSP00000299166	9606.ENSP00000392709	0.555	0.901	0.9	0.995
NDUFB8	NDUFS2	9606.ENSP00000299166	9606.ENSP00000356972	0.845	0.901	0.9	0.998
NDUFB8	NDUFS5	9606.ENSP00000299166	9606.ENSP00000362060	0.742	0.988	0.9	0.999
NDUFB8	NDUFV2	9606.ENSP00000299166	9606.ENSP00000327268	0.817	0.901	0.9	0.998
NDUFB9	NDUFS7	9606.ENSP00000276689	9606.ENSP00000233627	0.946	0.99	0.9	0.999
NDUFB9	NDUFS3	9606.ENSP00000276689	9606.ENSP00000263774	0.915	0.985	0.9	0.999
NDUFB9	NDUFS6	9606.ENSP00000276689	9606.ENSP00000274137	0.943	0.99	0.9	0.999
NDUFB9	NDUFV3	9606.ENSP00000276689	9606.ENSP00000346196	0.161	0.8	0.9	0.981
NDUFB9	NDUFC1	9606.ENSP00000276689	9606.ENSP00000441126	0.662	0.868	0.9	0.995
NDUFB9	NDUFV2	9606.ENSP00000276689	9606.ENSP00000327268	0.954	0.987	0.9	0.999
NDUFB9	NDUFS4	9606.ENSP00000276689	9606.ENSP00000296684	0.959	0.986	0.9	0.999
NDUFB9	NDUFC2	9606.ENSP00000276689	9606.ENSP00000281031	0.889	0.952	0.9	0.999
NDUFB9	NDUFS1	9606.ENSP00000276689	9606.ENSP00000392709	0.879	0.995	0.9	0.999
NDUFB9	NDUFV1	9606.ENSP00000276689	9606.ENSP00000322450	0.942	0.987	0.9	0.999
NDUFB9	NDUFS5	9606.ENSP00000276689	9606.ENSP00000362060	0.915	0.998	0.9	0.999
NDUFB9	NDUFS2	9606.ENSP00000276689	9606.ENSP00000356972	0.879	0.963	0.9	0.999
NDUFB9	NDUFS8	9606.ENSP00000276689	9606.ENSP00000315774	0.952	0.986	0.9	0.999
NDUFC1	NDUFS7	9606.ENSP00000441126	9606.ENSP00000233627	0.478	0.868	0.9	0.992
NDUFC1	NDUFS3	9606.ENSP00000441126	9606.ENSP00000263774	0.488	0.868	0.9	0.992
NDUFC1	NDUFS6	9606.ENSP00000441126	9606.ENSP00000274137	0.394	0.868	0.9	0.991
NDUFC1	NDUFC2	9606.ENSP00000441126	9606.ENSP00000281031	0.185	0.862	0.9	0.987
NDUFC1	NDUFS4	9606.ENSP00000441126	9606.ENSP00000296684	0.557	0.842	0.9	0.992
NDUFC1	NDUFS8	9606.ENSP00000441126	9606.ENSP00000315774	0.293	0.842	0.9	0.987
NDUFC1	NDUFV1	9606.ENSP00000441126	9606.ENSP00000322450	0.143	0.868	0.9	0.987
NDUFC1	NDUFV2	9606.ENSP00000441126	9606.ENSP00000327268	0.557	0.868	0.9	0.993
NDUFC1	NDUFV3	9606.ENSP00000441126	9606.ENSP00000346196	0.144	0.8	0.9	0.981
NDUFC1	NDUFS2	9606.ENSP00000441126	9606.ENSP00000356972	0.183	0.868	0.9	0.988
NDUFC1	NDUFS5	9606.ENSP00000441126	9606.ENSP00000362060	0.686	0.868	0.9	0.995
NDUFC1	NDUFS1	9606.ENSP00000441126	9606.ENSP00000392709	0.063	0.868	0.9	0.986

NDUFC2	NDUFS7	9606.ENSP00000281031	9606.ENSP00000233627	0.641	0.964	0.9	0.998
NDUFC2	NDUFS3	9606.ENSP00000281031	9606.ENSP00000263774	0.682	0.96	0.9	0.998
NDUFC2	NDUFS6	9606.ENSP00000281031	9606.ENSP00000274137	0.862	0.963	0.9	0.999
NDUFC2	NDUFV3	9606.ENSP00000281031	9606.ENSP00000346196	0.151	0.813	0.9	0.982
NDUFC2	NDUFS8	9606.ENSP00000281031	9606.ENSP00000315774	0.674	0.868	0.9	0.995
NDUFC2	NDUFS1	9606.ENSP00000281031	9606.ENSP00000392709	0.288	0.955	0.9	0.996
NDUFC2	NDUFS2	9606.ENSP00000281031	9606.ENSP00000356972	0.337	0.955	0.9	0.996
NDUFC2	NDUFV2	9606.ENSP00000281031	9606.ENSP00000327268	0.418	0.955	0.9	0.997
NDUFC2	NDUFV1	9606.ENSP00000281031	9606.ENSP00000322450	0.34	0.955	0.9	0.996
NDUFC2	NDUFS5	9606.ENSP00000281031	9606.ENSP00000362060	0.867	0.955	0.9	0.999
NDUFC2	NDUFS4	9606.ENSP00000281031	9606.ENSP00000296684	0.818	0.949	0.9	0.998
NDUFS1	NDUFS7	9606.ENSP00000392709	9606.ENSP00000233627	0.922	0.998	0.9	0.999
NDUFS1	NDUFS3	9606.ENSP00000392709	9606.ENSP00000263774	0.806	0.998	0.9	0.999
NDUFS1	NDUFS6	9606.ENSP00000392709	9606.ENSP00000274137	0.3	0.998	0.9	0.999
NDUFS1	NDUFS4	9606.ENSP00000392709	9606.ENSP00000296684	0.878	0.998	0.9	0.999
NDUFS1	NDUFS8	9606.ENSP00000392709	9606.ENSP00000315774	0.753	0.995	0.9	0.999
NDUFS1	NDUFV1	9606.ENSP00000392709	9606.ENSP00000322450	0.988	0.998	0.9	0.999
NDUFS1	NDUFV2	9606.ENSP00000392709	9606.ENSP00000327268	0.879	0.998	0.9	0.999
NDUFS1	NDUFV3	9606.ENSP00000392709	9606.ENSP00000346196	0.092	0.927	0.9	0.992
NDUFS1	NDUFS2	9606.ENSP00000392709	9606.ENSP00000356972	0.95	0.995	0.9	0.999
NDUFS1	NDUFS5	9606.ENSP00000392709	9606.ENSP00000362060	0.514	0.998	0.9	0.999
NDUFS2	NDUFS7	9606.ENSP00000356972	9606.ENSP00000233627	0.891	0.998	0.9	0.999
NDUFS2	NDUFS3	9606.ENSP00000356972	9606.ENSP00000263774	0.876	0.998	0.9	0.999
NDUFS2	NDUFS6	9606.ENSP00000356972	9606.ENSP00000274137	0.265	0.998	0.9	0.999
NDUFS2	NDUFS4	9606.ENSP00000356972	9606.ENSP00000296684	0.758	0.997	0.9	0.999
NDUFS2	NDUFS8	9606.ENSP00000356972	9606.ENSP00000315774	0.775	0.995	0.9	0.999
NDUFS2	NDUFV1	9606.ENSP00000356972	9606.ENSP00000322450	0.923	0.998	0.9	0.999
NDUFS2	NDUFV2	9606.ENSP00000356972	9606.ENSP00000327268	0.889	0.998	0.9	0.999
NDUFS2	NDUFV3	9606.ENSP00000356972	9606.ENSP00000346196	0.132	0.815	0.9	0.982
NDUFS2	NDUFS5	9606.ENSP00000356972	9606.ENSP00000362060	0.349	0.998	0.9	0.999
NDUFS3	NDUFS7	9606.ENSP00000263774	9606.ENSP00000233627	0.905	0.998	0.9	0.999
NDUFS3	NDUFV1	9606.ENSP00000263774	9606.ENSP00000322450	0.849	0.998	0.9	0.999
NDUFS3	NDUFS8	9606.ENSP00000263774	9606.ENSP00000315774	0.947	0.998	0.9	0.999
NDUFS3	NDUFV2	9606.ENSP00000263774	9606.ENSP00000327268	0.949	0.998	0.9	0.999
NDUFS3	NDUFV3	9606.ENSP00000263774	9606.ENSP00000346196	0.117	0.998	0.9	0.999
NDUFS3	NDUFS4	9606.ENSP00000263774	9606.ENSP00000296684	0.882	0.998	0.9	0.999
NDUFS3	NDUFS6	9606.ENSP00000263774	9606.ENSP00000274137	0.72	0.998	0.9	0.999
NDUFS3	NDUFS5	9606.ENSP00000263774	9606.ENSP00000362060	0.606	0.998	0.9	0.999
NDUFS4	NDUFS7	9606.ENSP00000296684	9606.ENSP00000233627	0.781	0.994	0.9	0.999
NDUFS4	NDUFS6	9606.ENSP00000296684	9606.ENSP00000274137	0.911	0.995	0.9	0.999
NDUFS4	NDUFS8	9606.ENSP00000296684	9606.ENSP00000315774	0.903	0.997	0.9	0.999
NDUFS4	NDUFV1	9606.ENSP00000296684	9606.ENSP00000322450	0.626	0.998	0.9	0.999
NDUFS4	NDUFS5	9606.ENSP00000296684	9606.ENSP00000362060	0.825	0.995	0.9	0.999
NDUFS4	NDUFV2	9606.ENSP00000296684	9606.ENSP00000327268	0.919	0.997	0.9	0.999

NDUFS4	NDUFV3	9606.ENSP00000296684	9606.ENSP00000346196	0.102	0.982	0.9	0.998
NDUFS5	NDUFS7	9606.ENSP00000362060	9606.ENSP00000233627	0.798	0.998	0.9	0.999
NDUFS5	NDUFS6	9606.ENSP00000362060	9606.ENSP00000274137	0.758	0.998	0.9	0.999
NDUFS5	NDUFS8	9606.ENSP00000362060	9606.ENSP00000315774	0.765	0.998	0.9	0.999
NDUFS5	NDUFV1	9606.ENSP00000362060	9606.ENSP00000322450	0.62	0.998	0.9	0.999
NDUFS5	NDUFV2	9606.ENSP00000362060	9606.ENSP00000327268	0.732	0.995	0.9	0.999
NDUFS5	NDUFV3	9606.ENSP00000362060	9606.ENSP00000346196	0.148	0.982	0.9	0.998
NDUFS6	NDUFS7	9606.ENSP00000274137	9606.ENSP00000233627	0.797	0.996	0.9	0.999
NDUFS6	NDUFS8	9606.ENSP00000274137	9606.ENSP00000315774	0.877	0.998	0.9	0.999
NDUFS6	NDUFV2	9606.ENSP00000274137	9606.ENSP00000327268	0.882	0.998	0.9	0.999
NDUFS6	NDUFV3	9606.ENSP00000274137	9606.ENSP00000346196	0.175	0.998	0.9	0.999
NDUFS6	NDUFV1	9606.ENSP00000274137	9606.ENSP00000322450	0.739	0.998	0.9	0.999
NDUFS7	NQO1	9606.ENSP00000233627	9606.ENSP00000319788	0	0.71	0	0.71
NDUFS7	NDUFV3	9606.ENSP00000233627	9606.ENSP00000346196	0.186	0.887	0.9	0.99
NDUFS7	NDUFS8	9606.ENSP00000233627	9606.ENSP00000315774	0.962	0.998	0.9	0.999
NDUFS7	NDUFV1	9606.ENSP00000233627	9606.ENSP00000322450	0.947	0.997	0.9	0.999
NDUFS7	NDUFV2	9606.ENSP00000233627	9606.ENSP00000327268	0.888	0.995	0.9	0.999
NDUFS8	NDUFV3	9606.ENSP00000315774	9606.ENSP00000346196	0.2	0.8	0.9	0.982
NDUFS8	NDUFV1	9606.ENSP00000315774	9606.ENSP00000322450	0.967	0.998	0.9	0.999
NDUFS8	NDUFV2	9606.ENSP00000315774	9606.ENSP00000327268	0.921	0.994	0.9	0.999
NDUFV1	NDUFV3	9606.ENSP00000322450	9606.ENSP00000346196	0.112	0.998	0.9	0.999
NDUFV1	NDUFV2	9606.ENSP00000322450	9606.ENSP00000327268	0.894	0.998	0.9	0.999
NDUFV2	NDUFV3	9606.ENSP00000327268	9606.ENSP00000346196	0.134	0.998	0.9	0.999
NFE2L2	PRKCB	9606.ENSP00000380252	9606.ENSP00000305355	0	0	0.9	0.9
NFE2L2	PRKCA	9606.ENSP00000380252	9606.ENSP00000408695	0	0.27	0.9	0.923
NFKB1	TNFRSF1A	9606.ENSP00000226574	9606.ENSP00000162749	0.062	0	0.9	0.902
NFKB1	PDPK1	9606.ENSP00000226574	9606.ENSP00000344220	0.087	0.056	0.65	0.672
NFKB1	PARP1	9606.ENSP00000226574	9606.ENSP00000355759	0.062	0.694	0	0.7
NFKB1	ROCK1	9606.ENSP00000226574	9606.ENSP00000382697	0	0.12	0.8	0.816
NFKB1	ROCK2	9606.ENSP00000226574	9606.ENSP00000317985	0	0.12	0.8	0.816
NFKB1	RAF1	9606.ENSP00000226574	9606.ENSP00000251849	0.061	0.12	0.8	0.82
NFKB1	PRKCQ	9606.ENSP00000226574	9606.ENSP00000263125	0.062	0.104	0.8	0.817
NFKB1	STAT3	9606.ENSP00000226574	9606.ENSP00000264657	0.094	0.638	0	0.658
NFKB1	TDP2	9606.ENSP00000226574	9606.ENSP00000367440	0.066	0	0.9	0.902
NFKB1	TLR2	9606.ENSP00000226574	9606.ENSP00000260010	0.119	0.072	0.8	0.822
NFKB1	PRKCZ	9606.ENSP00000226574	9606.ENSP00000367830	0	0.12	0.9	0.908
NFKB1	TERT	9606.ENSP00000226574	9606.ENSP00000309572	0.062	0	0.9	0.902
NFKB1	XPO1	9606.ENSP00000226574	9606.ENSP00000384863	0.134	0.078	0.9	0.913
NFKB1	PSMB5	9606.ENSP00000226574	9606.ENSP00000355325	0.085	0.141	0.9	0.914
NFKB1	SYK	9606.ENSP00000226574	9606.ENSP00000364907	0.062	0.12	0.9	0.91
NFKB1	TYK2	9606.ENSP00000226574	9606.ENSP00000431885	0.073	0.12	0.9	0.911
NFKB1	PSMB8	9606.ENSP00000226574	9606.ENSP00000364016	0.096	0.141	0.9	0.915
NFKB1	NLRP3	9606.ENSP00000226574	9606.ENSP00000337383	0.071	0	0.9	0.903
NFKB1	PRKCA	9606.ENSP00000226574	9606.ENSP00000408695	0	0.176	0.9	0.914

NFKB1	SRC	9606.ENSP00000226574	9606.ENSP00000362680	0.063	0.12	0.9	0.91
NFKB1	RPS6KA5	9606.ENSP00000226574	9606.ENSP00000479667	0	0.27	0.9	0.923
NFKB1	PIK3R1	9606.ENSP00000226574	9606.ENSP00000428056	0	0.062	0.9	0.902
NFKB1	PRKCD	9606.ENSP00000226574	9606.ENSP00000378217	0.089	0.104	0.9	0.911
NFKB1	PIK3CA	9606.ENSP00000226574	9606.ENSP00000263967	0.074	0.266	0.9	0.926
NFKB1	PPARG	9606.ENSP00000226574	9606.ENSP00000287820	0	0.282	0.9	0.925
NFKB1	TNF	9606.ENSP00000226574	9606.ENSP00000398698	0.108	0	0.9	0.907
NFKB1	NR3C1	9606.ENSP00000226574	9606.ENSP00000231509	0.064	0.683	0.9	0.967
NFKB1	RELA	9606.ENSP00000226574	9606.ENSP00000384273	0.134	0.998	0.9	0.999
NLRP1	NLRP3	9606.ENSP00000478516	9606.ENSP00000337383	0.085	0	0.9	0.904
NLRP3	SYK	9606.ENSP00000337383	9606.ENSP00000364907	0.129	0.345	0	0.405
NLRP3	RELA	9606.ENSP00000337383	9606.ENSP00000384273	0	0	0.9	0.9
NOS1	NOS3	9606.ENSP00000477999	9606.ENSP00000297494	0	0	0.8	0.8
NOS1	PRKCB	9606.ENSP00000477999	9606.ENSP00000305355	0.062	0	0.9	0.902
NOS1	NOS2	9606.ENSP00000477999	9606.ENSP00000327251	0	0.147	0.8	0.822
NOS1	TNNI3	9606.ENSP00000477999	9606.ENSP00000341838	0	0	0.9	0.9
NOS1	PRKCA	9606.ENSP00000477999	9606.ENSP00000408695	0.062	0.213	0.9	0.919
NOS2	STAT3	9606.ENSP00000327251	9606.ENSP00000264657	0	0	0.9	0.9
NOS2	SLC27A2	9606.ENSP00000327251	9606.ENSP00000267842	0	0	0.9	0.9
NOS2	PAOX	9606.ENSP00000327251	9606.ENSP00000278060	0	0	0.9	0.9
NOS2	NOS3	9606.ENSP00000327251	9606.ENSP00000297494	0	0.164	0.8	0.825
NOS2	RAC1	9606.ENSP00000327251	9606.ENSP00000348461	0	0.462	0	0.461
NOS2	RXRA	9606.ENSP00000327251	9606.ENSP00000419692	0	0.067	0.9	0.902
NOS2	PIK3R1	9606.ENSP00000327251	9606.ENSP00000428056	0.062	0	0.9	0.902
NOS2	PPARA	9606.ENSP00000327251	9606.ENSP00000385523	0	0.067	0.9	0.902
NOS2	TYK2	9606.ENSP00000327251	9606.ENSP00000431885	0	0.077	0.9	0.903
NOS2	SRC	9606.ENSP00000327251	9606.ENSP00000362680	0.065	0.242	0.9	0.922
NOS2	RELA	9606.ENSP00000327251	9606.ENSP00000384273	0	0.524	0.9	0.95
NOS2	TNF	9606.ENSP00000327251	9606.ENSP00000398698	0.063	0	0.9	0.902
NOS3	PDPK1	9606.ENSP00000297494	9606.ENSP00000344220	0.056	0	0.65	0.655
NOS3	PRKCD	9606.ENSP00000297494	9606.ENSP00000378217	0	0.06	0.9	0.901
NOS3	PTPN11	9606.ENSP00000297494	9606.ENSP00000340944	0	0.171	0.9	0.913
NOTUM	WNT3A	9606.ENSP00000387310	9606.ENSP00000284523	0	0.346	0.6	0.727
NOTUM	SHH	9606.ENSP00000387310	9606.ENSP00000297261	0.062	0	0.9	0.902
NOX1	NOX4	9606.ENSP00000362057	9606.ENSP00000263317	0	0.282	0.8	0.85
NOX1	RAC1	9606.ENSP00000362057	9606.ENSP00000348461	0.063	0.077	0.9	0.905
NOX4	PTPN1	9606.ENSP00000263317	9606.ENSP00000360683	0.049	0	0.9	0.9
NQO1	ODC1	9606.ENSP00000319788	9606.ENSP00000234111	0	0	0.8	0.8
NR1D1	NR2E3	9606.ENSP00000246672	9606.ENSP00000482504	0.062	0.499	0	0.51
NR1D1	RORA	9606.ENSP00000246672	9606.ENSP00000261523	0.11	0	0.9	0.907
NR1H2	PPARD	9606.ENSP00000253727	9606.ENSP00000310928	0.062	0.417	0	0.429
NR1H2	PPARG	9606.ENSP00000253727	9606.ENSP00000287820	0.062	0.417	0	0.429
NR1H2	PPARA	9606.ENSP00000253727	9606.ENSP00000385523	0.062	0.417	0	0.429
NR1H2	NR1H3	9606.ENSP00000253727	9606.ENSP00000477707	0	0	0.9	0.9

NR1H2	PLIN1	9606.ENSP00000253727	9606.ENSP00000300055	0	0	0.9	0.9
NR1H2	SCD	9606.ENSP00000253727	9606.ENSP00000359380	0	0	0.9	0.9
NR1H2	RXRA	9606.ENSP00000253727	9606.ENSP00000419692	0.062	0.912	0.9	0.991
NR1H2	RXRG	9606.ENSP00000253727	9606.ENSP00000352900	0.062	0.946	0.9	0.994
NR1H2	RXRB	9606.ENSP00000253727	9606.ENSP00000363817	0.081	0.964	0.9	0.996
NR1H3	PPARG	9606.ENSP00000477707	9606.ENSP00000287820	0.062	0.417	0	0.429
NR1H3	PLIN1	9606.ENSP00000477707	9606.ENSP00000300055	0	0	0.9	0.9
NR1H3	PPARD	9606.ENSP00000477707	9606.ENSP00000310928	0.052	0.417	0	0.423
NR1H3	RXRG	9606.ENSP00000477707	9606.ENSP00000352900	0.062	0.947	0.9	0.994
NR1H3	SCD	9606.ENSP00000477707	9606.ENSP00000359380	0.049	0	0.9	0.9
NR1H3	RXRB	9606.ENSP00000477707	9606.ENSP00000363817	0.062	0.973	0.9	0.997
NR1H3	RELA	9606.ENSP00000477707	9606.ENSP00000384273	0.063	0.057	0.9	0.903
NR1H3	PPARA	9606.ENSP00000477707	9606.ENSP00000385523	0.063	0.739	0.9	0.973
NR1H3	RXRA	9606.ENSP00000477707	9606.ENSP00000419692	0.063	0.986	0.9	0.998
NR1H4	RXRG	9606.ENSP00000447149	9606.ENSP00000352900	0.051	0.301	0.9	0.927
NR1H4	RXRB	9606.ENSP00000447149	9606.ENSP00000363817	0.062	0.469	0.9	0.945
NR1H4	RXRA	9606.ENSP00000447149	9606.ENSP00000419692	0.062	0.895	0.9	0.989
NR1I2	RXRB	9606.ENSP00000336528	9606.ENSP00000363817	0	0.57	0	0.57
NR1I2	RXRA	9606.ENSP00000336528	9606.ENSP00000419692	0.062	0.975	0	0.975
NR1I3	RXRA	9606.ENSP00000356959	9606.ENSP00000419692	0.062	0.866	0	0.869
NR2F2	PPARG	9606.ENSP00000377721	9606.ENSP00000287820	0	0	0.9	0.9
NR3C1	PGR	9606.ENSP00000231509	9606.ENSP00000325120	0	0	0.6	0.6
NR3C1	SMARCA2	9606.ENSP00000231509	9606.ENSP00000265773	0.067	0.518	0	0.531
NR3C1	NR3C2	9606.ENSP00000231509	9606.ENSP00000350815	0	0.486	0.6	0.785
NR3C1	PIK3CA	9606.ENSP00000231509	9606.ENSP00000263967	0.082	0.061	0.9	0.906
NR3C1	RXRA	9606.ENSP00000231509	9606.ENSP00000419692	0	0	0.9	0.9
NR3C1	PIK3R1	9606.ENSP00000231509	9606.ENSP00000428056	0.062	0.07	0.9	0.905
NR3C1	RARA	9606.ENSP00000231509	9606.ENSP00000254066	0	0.228	0.9	0.919
NR3C1	PER2	9606.ENSP00000231509	9606.ENSP00000254657	0	0.116	0.9	0.907
NR3C1	TNF	9606.ENSP00000231509	9606.ENSP00000398698	0	0	0.9	0.9
NR3C1	STAT3	9606.ENSP00000231509	9606.ENSP00000264657	0	0.837	0	0.837
NR3C1	RELA	9606.ENSP00000231509	9606.ENSP00000384273	0	0.839	0.9	0.983
NR3C1	SMARCA4	9606.ENSP00000231509	9606.ENSP00000395654	0	0.883	0.9	0.987
NR3C2	PGR	9606.ENSP00000350815	9606.ENSP00000325120	0.062	0	0.6	0.608
NR4A1	RXRG	9606.ENSP00000440864	9606.ENSP00000352900	0	0.376	0.9	0.934
NR4A1	RXRB	9606.ENSP00000440864	9606.ENSP00000363817	0	0.181	0.9	0.914
NR4A1	RXRA	9606.ENSP00000440864	9606.ENSP00000419692	0	0.722	0.9	0.971
NR4A1	RPS6KA5	9606.ENSP00000440864	9606.ENSP00000479667	0	0.6	0	0.6
NTRK1	PIK3CA	9606.ENSP00000431418	9606.ENSP00000263967	0.062	0.124	0.9	0.91
NTRK1	STAT3	9606.ENSP00000431418	9606.ENSP00000264657	0	0.159	0.9	0.912
NTRK1	PIK3CB	9606.ENSP00000431418	9606.ENSP00000289153	0.062	0.124	0.8	0.821
NTRK1	PTPN11	9606.ENSP00000431418	9606.ENSP00000340944	0.062	0.378	0.9	0.936
NTRK1	RET	9606.ENSP00000431418	9606.ENSP00000347942	0.062	0.392	0	0.405
NTRK1	RAC1	9606.ENSP00000431418	9606.ENSP00000348461	0	0.14	0.9	0.91

NTRK1	PIK3CD	9606.ENSP00000431418	9606.ENSP00000366563	0.062	0.104	0.8	0.817
NTRK1	PRKCZ	9606.ENSP00000431418	9606.ENSP00000367830	0	0	0.9	0.9
NTRK1	PTPN6	9606.ENSP00000431418	9606.ENSP00000391592	0.062	0.533	0	0.543
NTRK1	PIK3R1	9606.ENSP00000431418	9606.ENSP00000428056	0	0.664	0.9	0.965
ODC1	PSMB5	9606.ENSP00000234111	9606.ENSP00000355325	0.078	0	0.9	0.903
ODC1	PSMB8	9606.ENSP00000234111	9606.ENSP00000364016	0	0	0.9	0.9
OPRD1	OPRM1	9606.ENSP00000234961	9606.ENSP00000394624	0.069	0.501	0.3	0.646
OPRD1	STAT6	9606.ENSP00000234961	9606.ENSP00000300134	0	0.056	0.9	0.901
OPRM1	STAT6	9606.ENSP00000394624	9606.ENSP00000300134	0	0.056	0.9	0.901
P2RX3	P2RX7	9606.ENSP00000263314	9606.ENSP00000330696	0	0	0.8	0.8
PABPC1	RPS6KB1	9606.ENSP00000313007	9606.ENSP00000225577	0.066	0	0.9	0.902
PAFAH1B2	PLA2G2C	9606.ENSP00000435289	9606.ENSP00000247992	0	0	0.65	0.65
PAFAH1B2	PLA2G7	9606.ENSP00000435289	9606.ENSP00000274793	0	0	0.8	0.8
PAFAH1B2	PLA2G1B	9606.ENSP00000435289	9606.ENSP00000312286	0	0	0.65	0.65
PAFAH1B2	PLA2G6	9606.ENSP00000435289	9606.ENSP00000333142	0	0	0.65	0.65
PAFAH1B2	PLA2G4A	9606.ENSP00000435289	9606.ENSP00000356436	0	0	0.65	0.65
PAFAH1B2	PLA2G5	9606.ENSP00000435289	9606.ENSP00000364249	0	0	0.65	0.65
PAFAH1B2	PLA2G2A	9606.ENSP00000435289	9606.ENSP00000383364	0	0	0.65	0.65
PAFAH1B2	PLA2G10	9606.ENSP00000435289	9606.ENSP00000393847	0	0	0.65	0.65
PAFAH1B2	PLA2G4B	9606.ENSP00000435289	9606.ENSP00000396045	0	0	0.65	0.65
PAOX	SLC27A2	9606.ENSP00000278060	9606.ENSP00000267842	0	0	0.9	0.9
PARP1	POLK	9606.ENSP00000355759	9606.ENSP00000241436	0.063	0	0.6	0.609
PARP1	PARP2	9606.ENSP00000355759	9606.ENSP00000250416	0.098	0.678	0.3	0.778
PARP1	RARA	9606.ENSP00000355759	9606.ENSP00000254066	0.066	0.397	0	0.412
PARP1	POLB	9606.ENSP00000355759	9606.ENSP00000265421	0	0.754	0.6	0.897
PARP1	PRKDC	9606.ENSP00000355759	9606.ENSP00000313420	0.143	0.498	0	0.551
PARP1	TOP1	9606.ENSP00000355759	9606.ENSP00000354522	0.063	0.691	0	0.698
PARP1	POLA1	9606.ENSP00000355759	9606.ENSP00000368349	0.208	0.462	0	0.556
PARP1	TOP2A	9606.ENSP00000355759	9606.ENSP00000411532	0.176	0.389	0	0.475
PARP1	RELA	9606.ENSP00000355759	9606.ENSP00000384273	0	0.688	0	0.688
PARP1	PCNA	9606.ENSP00000355759	9606.ENSP00000368458	0.341	0.753	0.6	0.929
PARP2	POLK	9606.ENSP00000250416	9606.ENSP00000241436	0.066	0	0.6	0.61
PARP2	PCNA	9606.ENSP00000250416	9606.ENSP00000368458	0.224	0	0.6	0.676
PARP2	POLB	9606.ENSP00000250416	9606.ENSP00000265421	0	0.486	0.6	0.785
PASK	YWHAG	9606.ENSP00000351475	9606.ENSP00000306330	0.049	0.533	0	0.536
PBRM1	SMARCA2	9606.ENSP00000378307	9606.ENSP00000265773	0.154	0.812	0.6	0.931
PBRM1	SMARCA4	9606.ENSP00000378307	9606.ENSP00000395654	0.203	0.971	0.6	0.99
PCNA	POLK	9606.ENSP00000368458	9606.ENSP00000241436	0.067	0.918	0.6	0.966
PCNA	POLB	9606.ENSP00000368458	9606.ENSP00000265421	0	0.892	0.65	0.96
PCNA	SAE1	9606.ENSP00000368458	9606.ENSP00000270225	0.828	0	0	0.828
PCNA	PLK1	9606.ENSP00000368458	9606.ENSP00000300093	0.751	0	0	0.751
PCNA	PRKDC	9606.ENSP00000368458	9606.ENSP00000313420	0.17	0.416	0	0.495
PCNA	TYMS	9606.ENSP00000368458	9606.ENSP00000315644	0.959	0.115	0	0.962
PCNA	RAD52	9606.ENSP00000368458	9606.ENSP00000351284	0	0.706	0	0.706

PCNA	VCP	9606.ENSP00000368458	9606.ENSP00000351777	0.093	0	0.6	0.621
PCNA	TOP1	9606.ENSP00000368458	9606.ENSP00000354522	0.087	0.955	0	0.957
PCNA	TTK	9606.ENSP00000368458	9606.ENSP00000358813	0.549	0	0	0.549
PCNA	POLL	9606.ENSP00000368458	9606.ENSP00000359181	0	0.955	0	0.955
PCNA	POLH	9606.ENSP00000368458	9606.ENSP00000361310	0.108	0.996	0.6	0.998
PCNA	POLA1	9606.ENSP00000368458	9606.ENSP00000368349	0.847	0.681	0.72	0.985
PCNA	TOP2A	9606.ENSP00000368458	9606.ENSP00000411532	0.662	0.216	0	0.724
PCNA	POLM	9606.ENSP00000368458	9606.ENSP00000379174	0	0.943	0	0.943
PDCD4	RPS6KB1	9606.ENSP00000280154	9606.ENSP00000225577	0	0	0.9	0.9
PDE10A	PDE4D	9606.ENSP00000438284	9606.ENSP00000345502	0.062	0	0.5	0.51
PDE10A	PDE5A	9606.ENSP00000438284	9606.ENSP00000347046	0	0.339	0.5	0.655
PDE10A	PDE7A	9606.ENSP00000438284	9606.ENSP00000385632	0	0.339	0.5	0.655
PDE2A	PDE5A	9606.ENSP00000334910	9606.ENSP00000347046	0	0	0.5	0.499
PDE2A	PDE4D	9606.ENSP00000334910	9606.ENSP00000345502	0.062	0	0.5	0.51
PDE2A	PDE7A	9606.ENSP00000334910	9606.ENSP00000385632	0	0.212	0.5	0.589
PDE3A	PDE3B	9606.ENSP00000351957	9606.ENSP00000282096	0	0	0.9	0.9
PDE3B	PIK3CG	9606.ENSP00000282096	9606.ENSP00000352121	0.062	0.27	0.9	0.925
PDE4B	PDE4D	9606.ENSP00000332116	9606.ENSP00000345502	0	0.273	0.54	0.651
PDE4D	PDE7A	9606.ENSP00000345502	9606.ENSP00000385632	0	0	0.5	0.499
PDGFRA	TYK2	9606.ENSP00000257290	9606.ENSP00000431885	0	0.122	0.6	0.633
PDGFRA	STAT6	9606.ENSP00000257290	9606.ENSP00000300134	0	0.111	0.6	0.629
PDGFRA	PIK3CD	9606.ENSP00000257290	9606.ENSP00000366563	0	0.097	0.8	0.811
PDGFRA	PTK2	9606.ENSP00000257290	9606.ENSP00000341189	0	0.124	0.8	0.817
PDGFRA	SRC	9606.ENSP00000257290	9606.ENSP00000362680	0.062	0.122	0.8	0.82
PDGFRA	PIK3CB	9606.ENSP00000257290	9606.ENSP00000289153	0	0.097	0.8	0.811
PDGFRA	SPHK1	9606.ENSP00000257290	9606.ENSP00000313681	0	0	0.9	0.9
PDGFRA	STAT3	9606.ENSP00000257290	9606.ENSP00000264657	0	0.704	0.6	0.876
PDGFRA	PTPN11	9606.ENSP00000257290	9606.ENSP00000340944	0.063	0.684	0.8	0.935
PDGFRA	PIK3CA	9606.ENSP00000257290	9606.ENSP00000263967	0	0.312	0.9	0.928
PDGFRA	PDGFRB	9606.ENSP00000257290	9606.ENSP00000261799	0.287	0.777	0.9	0.982
PDGFRA	PIK3R1	9606.ENSP00000257290	9606.ENSP00000428056	0.062	0.815	0.9	0.981
PDGFRB	RAF1	9606.ENSP00000261799	9606.ENSP00000251849	0	0.705	0	0.705
PDGFRB	TNK2	9606.ENSP00000261799	9606.ENSP00000371341	0.062	0.486	0	0.497
PDGFRB	PTPN6	9606.ENSP00000261799	9606.ENSP00000391592	0.063	0.4	0	0.413
PDGFRB	STAT6	9606.ENSP00000261799	9606.ENSP00000300134	0.063	0.111	0.6	0.637
PDGFRB	TYK2	9606.ENSP00000261799	9606.ENSP00000431885	0	0.279	0.6	0.699
PDGFRB	PIK3CD	9606.ENSP00000261799	9606.ENSP00000366563	0	0.097	0.8	0.811
PDGFRB	PTK2	9606.ENSP00000261799	9606.ENSP00000341189	0	0.281	0.8	0.85
PDGFRB	RAC1	9606.ENSP00000261799	9606.ENSP00000348461	0	0.078	0.9	0.903
PDGFRB	SPHK1	9606.ENSP00000261799	9606.ENSP00000313681	0	0	0.9	0.9
PDGFRB	PLAU	9606.ENSP00000261799	9606.ENSP00000361850	0.069	0	0.9	0.902
PDGFRB	PIK3CG	9606.ENSP00000261799	9606.ENSP00000352121	0	0.097	0.9	0.905
PDGFRB	PTPN2	9606.ENSP00000261799	9606.ENSP00000311857	0	0.228	0.9	0.919
PDGFRB	PIK3CB	9606.ENSP00000261799	9606.ENSP00000289153	0	0.097	0.9	0.905

PDGFRB	YES1	9606.ENSP00000261799	9606.ENSP00000462468	0.049	0.331	0.9	0.93
PDGFRB	PIK3CA	9606.ENSP00000261799	9606.ENSP00000263967	0	0.258	0.9	0.922
PDGFRB	S1PR1	9606.ENSP00000261799	9606.ENSP00000305416	0.097	0.27	0.9	0.928
PDGFRB	PTPN1	9606.ENSP00000261799	9606.ENSP00000360683	0	0.68	0.9	0.966
PDGFRB	STAT3	9606.ENSP00000261799	9606.ENSP00000264657	0	0.629	0.9	0.961
PDGFRB	SRC	9606.ENSP00000261799	9606.ENSP00000362680	0.062	0.885	0.9	0.988
PDGFRB	PTPN11	9606.ENSP00000261799	9606.ENSP00000340944	0.063	0.899	0.9	0.989
PDGFRB	PIK3R1	9606.ENSP00000261799	9606.ENSP00000428056	0	0.925	0.9	0.992
PDK1	RPS6KB1	9606.ENSP00000376352	9606.ENSP00000225577	0	0.686	0	0.686
PDPK1	RIPK2	9606.ENSP00000344220	9606.ENSP00000220751	0.06	0.145	0.9	0.912
PDPK1	RPS6KB1	9606.ENSP00000344220	9606.ENSP00000225577	0.105	0.78	0.9	0.978
PDPK1	PRKCQ	9606.ENSP00000344220	9606.ENSP00000263125	0.064	0.479	0.9	0.947
PDPK1	PIK3CA	9606.ENSP00000344220	9606.ENSP00000263967	0.086	0.142	0.9	0.914
PDPK1	PIK3CB	9606.ENSP00000344220	9606.ENSP00000289153	0.064	0.142	0.8	0.825
PDPK1	PRKCB	9606.ENSP00000344220	9606.ENSP00000305355	0	0.27	0.9	0.923
PDPK1	PRKCE	9606.ENSP00000344220	9606.ENSP00000306124	0.064	0.616	0.9	0.96
PDPK1	RAC1	9606.ENSP00000344220	9606.ENSP00000348461	0.049	0	0.6	0.603
PDPK1	RELA	9606.ENSP00000344220	9606.ENSP00000384273	0.049	0	0.65	0.652
PDPK1	PRKCA	9606.ENSP00000344220	9606.ENSP00000408695	0	0	0.9	0.9
PDPK1	TGFBR1	9606.ENSP00000344220	9606.ENSP00000364133	0.062	0.145	0.9	0.912
PDPK1	PIK3CD	9606.ENSP00000344220	9606.ENSP00000366563	0.064	0.142	0.8	0.825
PDPK1	PIK3CG	9606.ENSP00000344220	9606.ENSP00000352121	0.064	0.142	0.8	0.825
PDPK1	SRC	9606.ENSP00000344220	9606.ENSP00000362680	0.062	0.298	0.9	0.928
PDPK1	PIK3R1	9606.ENSP00000344220	9606.ENSP00000428056	0.062	0	0.9	0.902
PDPK1	PRKCD	9606.ENSP00000344220	9606.ENSP00000378217	0.064	0.616	0.9	0.96
PDPK1	RPS6KA3	9606.ENSP00000344220	9606.ENSP00000368884	0.068	0.726	0.9	0.972
PDPK1	PRKCZ	9606.ENSP00000344220	9606.ENSP00000367830	0.053	0.791	0.9	0.978
PER2	PPP1CC	9606.ENSP00000254657	9606.ENSP00000341779	0	0.138	0.9	0.91
PFKFB3	PRKCG	9606.ENSP00000443319	9606.ENSP00000263431	0	0.542	0	0.542
PFKFB3	PRKCB	9606.ENSP00000443319	9606.ENSP00000305355	0	0.542	0	0.542
PFKFB3	PRKCA	9606.ENSP00000443319	9606.ENSP00000408695	0	0.542	0	0.542
PGR	PIK3CA	9606.ENSP00000325120	9606.ENSP00000263967	0	0.061	0.8	0.804
PGR	STAT3	9606.ENSP00000325120	9606.ENSP00000264657	0	0.592	0	0.592
PGR	PIK3CB	9606.ENSP00000325120	9606.ENSP00000289153	0	0.061	0.8	0.804
PGR	PIK3CD	9606.ENSP00000325120	9606.ENSP00000366563	0	0.061	0.8	0.804
PGR	PIK3R1	9606.ENSP00000325120	9606.ENSP00000428056	0	0.07	0.8	0.806
PGR	SRC	9606.ENSP00000325120	9606.ENSP00000362680	0.062	0.749	0.9	0.974
PI4KB	YWHAG	9606.ENSP00000357869	9606.ENSP00000306330	0	0.449	0	0.449
PI4KB	PIK3C2B	9606.ENSP00000357869	9606.ENSP00000356155	0.114	0	0.9	0.907
PI4KB	PIK3R1	9606.ENSP00000357869	9606.ENSP00000428056	0.066	0.244	0.65	0.731
PI4KB	PTK2B	9606.ENSP00000357869	9606.ENSP00000380638	0.093	0.058	0.9	0.907
PIK3C2B	PIK3CA	9606.ENSP00000356155	9606.ENSP00000263967	0.062	0.105	0.9	0.908
PIK3C2B	PIK3CB	9606.ENSP00000356155	9606.ENSP00000289153	0.062	0.122	0.54	0.588
PIK3C2B	PIK3CG	9606.ENSP00000356155	9606.ENSP00000352121	0.062	0.102	0.54	0.578

PIK3C2B	PIK3CD	9606.ENSP00000356155	9606.ENSP00000366563	0.062	0.146	0.54	0.599
PIK3C2B	PIK3R1	9606.ENSP00000356155	9606.ENSP00000428056	0.063	0.303	0.9	0.929
PIK3CA	RPS6KB1	9606.ENSP00000263967	9606.ENSP00000225577	0.065	0.354	0.9	0.934
PIK3CA	RAF1	9606.ENSP00000263967	9606.ENSP00000251849	0.062	0.104	0.8	0.817
PIK3CA	PRKCQ	9606.ENSP00000263967	9606.ENSP00000263125	0.064	0.151	0.9	0.913
PIK3CA	TXK	9606.ENSP00000263967	9606.ENSP00000264316	0.062	0.104	0.6	0.634
PIK3CA	YES1	9606.ENSP00000263967	9606.ENSP00000462468	0.062	0.104	0.6	0.634
PIK3CA	THRA	9606.ENSP00000263967	9606.ENSP00000264637	0	0.061	0.8	0.804
PIK3CA	THRB	9606.ENSP00000263967	9606.ENSP00000379904	0	0.061	0.8	0.804
PIK3CA	PIK3CD	9606.ENSP00000263967	9606.ENSP00000366563	0.062	0	0.8	0.804
PIK3CA	S1PR1	9606.ENSP00000263967	9606.ENSP00000305416	0	0	0.9	0.9
PIK3CA	PIK3CB	9606.ENSP00000263967	9606.ENSP00000289153	0.062	0	0.9	0.902
PIK3CA	PTPN6	9606.ENSP00000263967	9606.ENSP00000391592	0	0.066	0.9	0.902
PIK3CA	PIK3CG	9606.ENSP00000263967	9606.ENSP00000352121	0.062	0.056	0.9	0.903
PIK3CA	TYK2	9606.ENSP00000263967	9606.ENSP00000431885	0.062	0.104	0.9	0.908
PIK3CA	PTPN1	9606.ENSP00000263967	9606.ENSP00000360683	0.062	0.132	0.9	0.911
PIK3CA	PRKCZ	9606.ENSP00000263967	9606.ENSP00000367830	0.062	0.104	0.9	0.908
PIK3CA	SYK	9606.ENSP00000263967	9606.ENSP00000364907	0.063	0.104	0.9	0.908
PIK3CA	PRKCD	9606.ENSP00000263967	9606.ENSP00000378217	0.064	0.303	0.9	0.929
PIK3CA	RAC1	9606.ENSP00000263967	9606.ENSP00000348461	0	0.353	0.9	0.932
PIK3CA	RELA	9606.ENSP00000263967	9606.ENSP00000384273	0	0.27	0.9	0.923
PIK3CA	STAT3	9606.ENSP00000263967	9606.ENSP00000264657	0.049	0	0.9	0.9
PIK3CA	PTK2	9606.ENSP00000263967	9606.ENSP00000341189	0.072	0.297	0.9	0.929
PIK3CA	RET	9606.ENSP00000263967	9606.ENSP00000347942	0	0.097	0.9	0.905
PIK3CA	SRC	9606.ENSP00000263967	9606.ENSP00000362680	0.062	0.104	0.9	0.908
PIK3CA	PTPN11	9606.ENSP00000263967	9606.ENSP00000340944	0.064	0.683	0.9	0.967
PIK3CA	PIK3R1	9606.ENSP00000263967	9606.ENSP00000428056	0.084	0.998	0.9	0.999
PIK3CB	RAF1	9606.ENSP00000289153	9606.ENSP00000251849	0.062	0.104	0.8	0.817
PIK3CB	TXK	9606.ENSP00000289153	9606.ENSP00000264316	0.062	0.104	0.6	0.634
PIK3CB	THRA	9606.ENSP00000289153	9606.ENSP00000264637	0	0.061	0.8	0.804
PIK3CB	PTPN6	9606.ENSP00000289153	9606.ENSP00000391592	0	0.066	0.6	0.61
PIK3CB	TYK2	9606.ENSP00000289153	9606.ENSP00000431885	0.062	0.104	0.6	0.634
PIK3CB	RET	9606.ENSP00000289153	9606.ENSP00000347942	0	0.097	0.6	0.623
PIK3CB	YES1	9606.ENSP00000289153	9606.ENSP00000462468	0.062	0.104	0.6	0.634
PIK3CB	THRB	9606.ENSP00000289153	9606.ENSP00000379904	0	0.061	0.8	0.804
PIK3CB	PIK3CD	9606.ENSP00000289153	9606.ENSP00000366563	0.062	0	0.8	0.804
PIK3CB	PTPN11	9606.ENSP00000289153	9606.ENSP00000340944	0.062	0.066	0.8	0.809
PIK3CB	PTK2	9606.ENSP00000289153	9606.ENSP00000341189	0.072	0.143	0.8	0.827
PIK3CB	PRKCD	9606.ENSP00000289153	9606.ENSP00000378217	0.064	0.353	0.8	0.868
PIK3CB	PIK3CG	9606.ENSP00000289153	9606.ENSP00000352121	0.052	0.056	0.9	0.902
PIK3CB	PRKCZ	9606.ENSP00000289153	9606.ENSP00000367830	0.062	0.104	0.9	0.908
PIK3CB	SYK	9606.ENSP00000289153	9606.ENSP00000364907	0.063	0.104	0.9	0.908
PIK3CB	RAC1	9606.ENSP00000289153	9606.ENSP00000348461	0	0.151	0.9	0.911
PIK3CB	SRC	9606.ENSP00000289153	9606.ENSP00000362680	0.062	0.104	0.9	0.908

PIK3CB	PIK3R1	9606.ENSP00000289153	9606.ENSP00000428056	0.064	0.946	0.9	0.994
PIK3CD	RAF1	9606.ENSP00000366563	9606.ENSP00000251849	0.062	0.104	0.8	0.817
PIK3CD	TXK	9606.ENSP00000366563	9606.ENSP00000264316	0.09	0.104	0.6	0.645
PIK3CD	THRA	9606.ENSP00000366563	9606.ENSP00000264637	0	0.061	0.8	0.804
PIK3CD	PTPN11	9606.ENSP00000366563	9606.ENSP00000340944	0.062	0.066	0.8	0.809
PIK3CD	PTK2	9606.ENSP00000366563	9606.ENSP00000341189	0.072	0.143	0.8	0.827
PIK3CD	RET	9606.ENSP00000366563	9606.ENSP00000347942	0	0.097	0.6	0.623
PIK3CD	PIK3CG	9606.ENSP00000366563	9606.ENSP00000352121	0.106	0.282	0.8	0.86
PIK3CD	SRC	9606.ENSP00000366563	9606.ENSP00000362680	0.062	0.104	0.8	0.817
PIK3CD	SYK	9606.ENSP00000366563	9606.ENSP00000364907	0.097	0.104	0.8	0.824
PIK3CD	PTPN6	9606.ENSP00000366563	9606.ENSP00000391592	0.077	0.066	0.6	0.625
PIK3CD	TYK2	9606.ENSP00000366563	9606.ENSP00000431885	0.062	0.104	0.6	0.634
PIK3CD	YES1	9606.ENSP00000366563	9606.ENSP00000462468	0.062	0.104	0.6	0.634
PIK3CD	THRB	9606.ENSP00000366563	9606.ENSP00000379904	0	0.061	0.8	0.804
PIK3CD	PRKCZ	9606.ENSP00000366563	9606.ENSP00000367830	0.062	0.104	0.8	0.817
PIK3CD	PRKCD	9606.ENSP00000366563	9606.ENSP00000378217	0.085	0.151	0.8	0.831
PIK3CD	PIK3R1	9606.ENSP00000366563	9606.ENSP00000428056	0.064	0.986	0.8	0.997
PIK3CG	PTPN11	9606.ENSP00000352121	9606.ENSP00000340944	0.053	0.476	0	0.482
PIK3CG	PTK2	9606.ENSP00000352121	9606.ENSP00000341189	0.066	0.076	0.8	0.812
PIK3CG	SRC	9606.ENSP00000352121	9606.ENSP00000362680	0.062	0.104	0.6	0.634
PIK3CG	PRKCZ	9606.ENSP00000352121	9606.ENSP00000367830	0.062	0.104	0.9	0.908
PIK3CG	SYK	9606.ENSP00000352121	9606.ENSP00000364907	0.148	0.317	0.9	0.936
PIK3CG	PIK3R1	9606.ENSP00000352121	9606.ENSP00000428056	0.063	0.537	0.9	0.952
PIK3R1	RPS6KB1	9606.ENSP00000428056	9606.ENSP00000225577	0.065	0.109	0.9	0.909
PIK3R1	RAF1	9606.ENSP00000428056	9606.ENSP00000251849	0	0.102	0.8	0.812
PIK3R1	TLR2	9606.ENSP00000428056	9606.ENSP00000260010	0	0.491	0	0.491
PIK3R1	TXK	9606.ENSP00000428056	9606.ENSP00000264316	0	0.292	0.6	0.704
PIK3R1	THRA	9606.ENSP00000428056	9606.ENSP00000264637	0.07	0.427	0.8	0.884
PIK3R1	STAT3	9606.ENSP00000428056	9606.ENSP00000264657	0	0.28	0.9	0.924
PIK3R1	S1PR1	9606.ENSP00000428056	9606.ENSP00000305416	0.063	0	0.9	0.902
PIK3R1	PTPN11	9606.ENSP00000428056	9606.ENSP00000340944	0.062	0.912	0.9	0.991
PIK3R1	PTK2	9606.ENSP00000428056	9606.ENSP00000341189	0	0.875	0.9	0.986
PIK3R1	RET	9606.ENSP00000428056	9606.ENSP00000347942	0.053	0.569	0.9	0.955
PIK3R1	RAC1	9606.ENSP00000428056	9606.ENSP00000348461	0	0.679	0.9	0.966
PIK3R1	PTPN1	9606.ENSP00000428056	9606.ENSP00000360683	0	0.095	0.9	0.905
PIK3R1	SRC	9606.ENSP00000428056	9606.ENSP00000362680	0	0.889	0.9	0.988
PIK3R1	TGFBR1	9606.ENSP00000428056	9606.ENSP00000364133	0	0.518	0	0.518
PIK3R1	SYK	9606.ENSP00000428056	9606.ENSP00000364907	0	0.518	0.9	0.949
PIK3R1	PRKCZ	9606.ENSP00000428056	9606.ENSP00000367830	0.054	0.102	0.9	0.907
PIK3R1	PRKCD	9606.ENSP00000428056	9606.ENSP00000378217	0	0.059	0.9	0.901
PIK3R1	THRB	9606.ENSP00000428056	9606.ENSP00000379904	0.062	0.495	0.8	0.897
PIK3R1	PTK2B	9606.ENSP00000428056	9606.ENSP00000380638	0.076	0.462	0	0.481
PIK3R1	RELA	9606.ENSP00000428056	9606.ENSP00000384273	0	0	0.9	0.9
PIK3R1	PTPN6	9606.ENSP00000428056	9606.ENSP00000391592	0	0.749	0.9	0.973

PIK3R1	YES1	9606.ENSP00000428056	9606.ENSP00000462468	0	0.316	0.6	0.714
PIK3R1	TYK2	9606.ENSP00000428056	9606.ENSP00000431885	0	0.263	0.9	0.923
PIM1	RORA	9606.ENSP00000362608	9606.ENSP00000261523	0	0	0.9	0.9
PIM1	STAT3	9606.ENSP00000362608	9606.ENSP00000264657	0.053	0.27	0.9	0.924
PIM1	RORC	9606.ENSP00000362608	9606.ENSP00000327025	0	0	0.9	0.9
PIM1	PIM2	9606.ENSP00000362608	9606.ENSP00000365692	0.076	0.105	0.8	0.82
PIN1	PLK1	9606.ENSP00000247970	9606.ENSP00000300093	0	0.675	0	0.675
PIN1	PKM	9606.ENSP00000247970	9606.ENSP00000320171	0.062	0.477	0	0.488
PIN1	YWHAG	9606.ENSP00000247970	9606.ENSP00000306330	0	0	0.9	0.9
PIN1	PRKCZ	9606.ENSP00000247970	9606.ENSP00000367830	0	0.06	0.9	0.901
PIN1	PRKCD	9606.ENSP00000247970	9606.ENSP00000378217	0.059	0	0.9	0.901
PIN1	PRKCA	9606.ENSP00000247970	9606.ENSP00000408695	0	0	0.9	0.9
PIN1	PRKCB	9606.ENSP00000247970	9606.ENSP00000305355	0	0	0.9	0.9
PIN1	RAF1	9606.ENSP00000247970	9606.ENSP00000251849	0.072	0.707	0.9	0.97
PLA2G10	PLA2G7	9606.ENSP00000393847	9606.ENSP00000274793	0	0	0.65	0.65
PLA2G10	PLA2G1B	9606.ENSP00000393847	9606.ENSP00000312286	0.063	0	0.6	0.609
PLA2G10	PTGS1	9606.ENSP00000393847	9606.ENSP00000354612	0.054	0	0.65	0.654
PLA2G10	PTGS2	9606.ENSP00000393847	9606.ENSP00000356438	0.054	0	0.65	0.654
PLA2G10	PLA2G5	9606.ENSP00000393847	9606.ENSP00000364249	0	0	0.6	0.6
PLA2G10	PLA2G2A	9606.ENSP00000393847	9606.ENSP00000383364	0	0	0.6	0.6
PLA2G1B	PLA2G7	9606.ENSP00000312286	9606.ENSP00000274793	0	0	0.65	0.65
PLA2G1B	PLA2G5	9606.ENSP00000312286	9606.ENSP00000364249	0.052	0	0.6	0.604
PLA2G1B	PLA2G2A	9606.ENSP00000312286	9606.ENSP00000383364	0	0.27	0.6	0.695
PLA2G1B	PTGS1	9606.ENSP00000312286	9606.ENSP00000354612	0	0	0.65	0.65
PLA2G1B	PTGS2	9606.ENSP00000312286	9606.ENSP00000356438	0	0	0.65	0.65
PLA2G2A	PLA2G7	9606.ENSP00000383364	9606.ENSP00000274793	0	0	0.65	0.65
PLA2G2A	PTGS1	9606.ENSP00000383364	9606.ENSP00000354612	0.062	0	0.65	0.657
PLA2G2A	PTGS2	9606.ENSP00000383364	9606.ENSP00000356438	0.062	0	0.65	0.657
PLA2G2A	PLA2G5	9606.ENSP00000383364	9606.ENSP00000364249	0.062	0	0.6	0.608
PLA2G2C	PLA2G7	9606.ENSP00000247992	9606.ENSP00000274793	0.062	0	0.65	0.657
PLA2G2C	PTGS1	9606.ENSP00000247992	9606.ENSP00000354612	0	0	0.65	0.65
PLA2G2C	PTGS2	9606.ENSP00000247992	9606.ENSP00000356438	0	0	0.65	0.65
PLA2G4A	PRKCG	9606.ENSP00000356436	9606.ENSP00000263431	0	0	0.6	0.6
PLA2G4A	PLA2G7	9606.ENSP00000356436	9606.ENSP00000274793	0	0	0.65	0.65
PLA2G4A	PRKCB	9606.ENSP00000356436	9606.ENSP00000305355	0	0	0.6	0.6
PLA2G4A	PTGS1	9606.ENSP00000356436	9606.ENSP00000354612	0.153	0	0.65	0.691
PLA2G4A	PLA2G4B	9606.ENSP00000356436	9606.ENSP00000396045	0.049	0	0.5	0.504
PLA2G4A	PRKCA	9606.ENSP00000356436	9606.ENSP00000408695	0	0.27	0.6	0.695
PLA2G4A	PTGS2	9606.ENSP00000356436	9606.ENSP00000356438	0.232	0	0.65	0.72
PLA2G4A	SYK	9606.ENSP00000356436	9606.ENSP00000364907	0.062	0.112	0.9	0.909
PLA2G4B	PRKCG	9606.ENSP00000396045	9606.ENSP00000263431	0.062	0.056	0.6	0.614
PLA2G4B	PLA2G7	9606.ENSP00000396045	9606.ENSP00000274793	0	0	0.65	0.65
PLA2G4B	PRKCB	9606.ENSP00000396045	9606.ENSP00000305355	0.062	0.056	0.6	0.614
PLA2G4B	PTGS1	9606.ENSP00000396045	9606.ENSP00000354612	0.049	0	0.65	0.652

PLA2G4B	PTGS2	9606.ENSP00000396045	9606.ENSP00000356438	0.049	0	0.65	0.652
PLA2G4B	PRKCA	9606.ENSP00000396045	9606.ENSP00000408695	0.062	0.056	0.6	0.614
PLA2G5	PLA2G7	9606.ENSP00000364249	9606.ENSP00000274793	0	0	0.65	0.65
PLA2G5	PTGS1	9606.ENSP00000364249	9606.ENSP00000354612	0.055	0	0.65	0.655
PLA2G5	PTGS2	9606.ENSP00000364249	9606.ENSP00000356438	0.055	0	0.65	0.655
PLA2G6	PRKCG	9606.ENSP00000333142	9606.ENSP00000263431	0.063	0	0.8	0.804
PLA2G6	PLA2G7	9606.ENSP00000333142	9606.ENSP00000274793	0.064	0	0.65	0.658
PLA2G6	PRKCB	9606.ENSP00000333142	9606.ENSP00000305355	0.063	0	0.8	0.804
PLA2G6	PRKCE	9606.ENSP00000333142	9606.ENSP00000306124	0	0	0.8	0.8
PLA2G6	PRKCD	9606.ENSP00000333142	9606.ENSP00000378217	0	0	0.8	0.8
PLA2G6	PTGS1	9606.ENSP00000333142	9606.ENSP00000354612	0.062	0.244	0.65	0.73
PLA2G6	PRKCA	9606.ENSP00000333142	9606.ENSP00000408695	0.063	0	0.8	0.804
PLA2G6	PTGS2	9606.ENSP00000333142	9606.ENSP00000356438	0.062	0.244	0.65	0.73
PLAU	SRC	9606.ENSP00000361850	9606.ENSP00000362680	0.069	0.056	0.9	0.904
PLIN1	PPARG	9606.ENSP00000300055	9606.ENSP00000287820	0.064	0	0.9	0.902
PLIN1	PPP1CC	9606.ENSP00000300055	9606.ENSP00000341779	0	0	0.9	0.9
PLIN1	RXRB	9606.ENSP00000300055	9606.ENSP00000363817	0.062	0	0.9	0.902
PLIN1	RXRA	9606.ENSP00000300055	9606.ENSP00000419692	0.062	0	0.9	0.902
PLK1	POLA1	9606.ENSP00000300093	9606.ENSP00000368349	0.37	0.153	0	0.444
PLK1	TYMS	9606.ENSP00000300093	9606.ENSP00000315644	0.626	0	0	0.626
PLK1	ROCK2	9606.ENSP00000300093	9606.ENSP00000317985	0.085	0.059	0.9	0.906
PLK1	TOP2A	9606.ENSP00000300093	9606.ENSP00000411532	0.859	0.27	0	0.892
PLK1	TTK	9606.ENSP00000300093	9606.ENSP00000358813	0.93	0.11	0	0.935
PLK1	WEE1	9606.ENSP00000300093	9606.ENSP00000402084	0.196	0.606	0.9	0.965
PNP	SIRT2	9606.ENSP00000354532	9606.ENSP00000249396	0	0	0.9	0.9
PNP	TYMP	9606.ENSP00000354532	9606.ENSP00000379038	0	0	0.8	0.8
POLA1	TERT	9606.ENSP00000368349	9606.ENSP00000309572	0.089	0.243	0.72	0.79
POLA1	TYMS	9606.ENSP00000368349	9606.ENSP00000315644	0.849	0.056	0	0.851
POLA1	TTK	9606.ENSP00000368349	9606.ENSP00000358813	0.839	0	0	0.839
POLA1	TOP2A	9606.ENSP00000368349	9606.ENSP00000411532	0.586	0.245	0	0.674
POLB	TDP1	9606.ENSP00000265421	9606.ENSP00000337353	0	0.741	0	0.741
POLH	POLK	9606.ENSP00000361310	9606.ENSP00000241436	0.143	0.144	0.3	0.441
POLH	VCP	9606.ENSP00000361310	9606.ENSP00000351777	0.062	0	0.6	0.608
POLL	PRKDC	9606.ENSP00000359181	9606.ENSP00000313420	0.062	0	0.9	0.902
POLL	POLM	9606.ENSP00000359181	9606.ENSP00000379174	0.063	0	0.9	0.902
POLM	PRKDC	9606.ENSP00000379174	9606.ENSP00000313420	0.062	0	0.9	0.902
PPARA	RXRG	9606.ENSP00000385523	9606.ENSP00000352900	0.062	0.333	0.9	0.931
PPARA	RXRB	9606.ENSP00000385523	9606.ENSP00000363817	0.058	0.294	0.9	0.927
PPARA	RELA	9606.ENSP00000385523	9606.ENSP00000384273	0	0.282	0.9	0.925
PPARA	TNF	9606.ENSP00000385523	9606.ENSP00000398698	0	0	0.9	0.9
PPARA	RXRA	9606.ENSP00000385523	9606.ENSP00000419692	0.062	0.885	0.9	0.988
PPARD	RXRG	9606.ENSP00000310928	9606.ENSP00000352900	0	0.332	0.9	0.93
PPARD	RXRB	9606.ENSP00000310928	9606.ENSP00000363817	0.072	0.417	0.9	0.941
PPARD	RXRA	9606.ENSP00000310928	9606.ENSP00000419692	0.062	0.684	0.9	0.967

PPARG	TNFRSF1A	9606.ENSP00000287820	9606.ENSP00000162749	0.062	0	0.9	0.902
PPARG	SMARCA2	9606.ENSP00000287820	9606.ENSP00000265773	0	0.078	0.6	0.615
PPARG	SMARCA4	9606.ENSP00000287820	9606.ENSP00000395654	0	0.298	0.6	0.707
PPARG	RXRG	9606.ENSP00000287820	9606.ENSP00000352900	0	0.472	0.9	0.944
PPARG	RXRB	9606.ENSP00000287820	9606.ENSP00000363817	0.049	0.483	0.9	0.946
PPARG	SREBF2	9606.ENSP00000287820	9606.ENSP00000354476	0	0	0.9	0.9
PPARG	TNF	9606.ENSP00000287820	9606.ENSP00000398698	0	0	0.9	0.9
PPARG	RXRA	9606.ENSP00000287820	9606.ENSP00000419692	0.062	0.981	0.9	0.998
PPARG	RELA	9606.ENSP00000287820	9606.ENSP00000384273	0	0.768	0.9	0.975
PPP1CC	RAF1	9606.ENSP00000341779	9606.ENSP00000251849	0	0.327	0.9	0.929
PPP1CC	PRKCE	9606.ENSP00000341779	9606.ENSP00000306124	0.066	0.147	0.8	0.826
PPP1CC	ROCK2	9606.ENSP00000341779	9606.ENSP00000317985	0.064	0.117	0.8	0.82
PPP1CC	TRPM8	9606.ENSP00000341779	9606.ENSP00000323926	0.062	0	0.8	0.804
PPP1CC	VDR	9606.ENSP00000341779	9606.ENSP00000447173	0	0.494	0	0.494
PPP1CC	RPS6KA3	9606.ENSP00000341779	9606.ENSP00000368884	0	0	0.6	0.6
PPP1CC	VCP	9606.ENSP00000341779	9606.ENSP00000351777	0.051	0.527	0	0.532
PPP1CC	TGFBR1	9606.ENSP00000341779	9606.ENSP00000364133	0.063	0.117	0.6	0.64
PPP1CC	ROCK1	9606.ENSP00000341779	9606.ENSP00000382697	0.097	0.117	0.8	0.826
PRKCA	RPS6KB1	9606.ENSP00000408695	9606.ENSP00000225577	0	0	0.9	0.9
PRKCA	SPHK2	9606.ENSP00000408695	9606.ENSP00000245222	0.062	0	0.8	0.804
PRKCA	RAF1	9606.ENSP00000408695	9606.ENSP00000251849	0	0.229	0.9	0.919
PRKCA	RARA	9606.ENSP00000408695	9606.ENSP00000254066	0	0.213	0.9	0.917
PRKCA	PRKCQ	9606.ENSP00000408695	9606.ENSP00000263125	0	0	0.5	0.499
PRKCA	PRKCG	9606.ENSP00000408695	9606.ENSP00000263431	0	0.489	0.8	0.893
PRKCA	SLC6A3	9606.ENSP00000408695	9606.ENSP00000270349	0	0	0.8	0.8
PRKCA	PRKCB	9606.ENSP00000408695	9606.ENSP00000305355	0	0.179	0.8	0.828
PRKCA	PRKCE	9606.ENSP00000408695	9606.ENSP00000306124	0.062	0.48	0.5	0.734
PRKCA	YWHAG	9606.ENSP00000408695	9606.ENSP00000306330	0	0.27	0.9	0.923
PRKCA	TERT	9606.ENSP00000408695	9606.ENSP00000309572	0	0.213	0.9	0.917
PRKCA	SPHK1	9606.ENSP00000408695	9606.ENSP00000313681	0.062	0	0.9	0.902
PRKCA	PRKCH	9606.ENSP00000408695	9606.ENSP00000329127	0	0.179	0.5	0.571
PRKCA	PTK2	9606.ENSP00000408695	9606.ENSP00000341189	0	0	0.8	0.8
PRKCA	RET	9606.ENSP00000408695	9606.ENSP00000347942	0	0	0.9	0.9
PRKCA	RAC1	9606.ENSP00000408695	9606.ENSP00000348461	0	0.47	0.9	0.944
PRKCA	RXRG	9606.ENSP00000408695	9606.ENSP00000352900	0	0	0.9	0.9
PRKCA	SRC	9606.ENSP00000408695	9606.ENSP00000362680	0	0.229	0.9	0.919
PRKCA	RXRB	9606.ENSP00000408695	9606.ENSP00000363817	0	0	0.9	0.9
PRKCA	PRKCZ	9606.ENSP00000408695	9606.ENSP00000367830	0	0.385	0.9	0.935
PRKCA	PRKCD	9606.ENSP00000408695	9606.ENSP00000378217	0	0	0.8	0.8
PRKCA	PTK2B	9606.ENSP00000408695	9606.ENSP00000380638	0	0	0.9	0.9
PRKCA	RELA	9606.ENSP00000408695	9606.ENSP00000384273	0	0	0.9	0.9
PRKCA	TBXA2R	9606.ENSP00000408695	9606.ENSP00000393333	0	0.496	0.9	0.947
PRKCA	TOP2A	9606.ENSP00000408695	9606.ENSP00000411532	0	0.574	0	0.574
PRKCA	RXRA	9606.ENSP00000408695	9606.ENSP00000419692	0	0	0.9	0.9

PRKCB	RPS6KB1	9606.ENSP00000305355	9606.ENSP00000225577	0	0	0.9	0.9
PRKCB	SPHK2	9606.ENSP00000305355	9606.ENSP00000245222	0.062	0	0.8	0.804
PRKCB	RAF1	9606.ENSP00000305355	9606.ENSP00000251849	0	0.061	0.9	0.902
PRKCB	PRKCQ	9606.ENSP00000305355	9606.ENSP00000263125	0.09	0	0.8	0.81
PRKCB	TYR	9606.ENSP00000305355	9606.ENSP00000263321	0.063	0	0.8	0.804
PRKCB	PRKCG	9606.ENSP00000305355	9606.ENSP00000263431	0.085	0	0.8	0.809
PRKCB	SLC6A3	9606.ENSP00000305355	9606.ENSP00000270349	0	0	0.8	0.8
PRKCB	PRKCH	9606.ENSP00000305355	9606.ENSP00000329127	0	0	0.5	0.499
PRKCB	PRKCE	9606.ENSP00000305355	9606.ENSP00000306124	0.062	0	0.5	0.51
PRKCB	TOP2A	9606.ENSP00000305355	9606.ENSP00000411532	0	0.574	0	0.574
PRKCB	PTK2	9606.ENSP00000305355	9606.ENSP00000341189	0	0	0.8	0.8
PRKCB	PRKCD	9606.ENSP00000305355	9606.ENSP00000378217	0	0.274	0.8	0.848
PRKCB	PRKCZ	9606.ENSP00000305355	9606.ENSP00000367830	0.062	0.061	0.9	0.904
PRKCB	TBXA2R	9606.ENSP00000305355	9606.ENSP00000393333	0	0.056	0.9	0.901
PRKCB	SPHK1	9606.ENSP00000305355	9606.ENSP00000313681	0.062	0	0.9	0.902
PRKCB	PTK2B	9606.ENSP00000305355	9606.ENSP00000380638	0.123	0	0.9	0.908
PRKCB	SRC	9606.ENSP00000305355	9606.ENSP00000362680	0	0.061	0.9	0.902
PRKCB	RELA	9606.ENSP00000305355	9606.ENSP00000384273	0	0	0.9	0.9
PRKCD	RAF1	9606.ENSP00000378217	9606.ENSP00000251849	0.066	0.275	0.9	0.926
PRKCD	VHL	9606.ENSP00000378217	9606.ENSP00000256474	0	0.651	0	0.651
PRKCD	PRKCQ	9606.ENSP00000378217	9606.ENSP00000263125	0	0.274	0.8	0.848
PRKCD	PRKCG	9606.ENSP00000378217	9606.ENSP00000263431	0	0	0.5	0.499
PRKCD	STAT3	9606.ENSP00000378217	9606.ENSP00000264657	0.062	0.462	0.9	0.945
PRKCD	SHH	9606.ENSP00000378217	9606.ENSP00000297261	0	0	0.9	0.9
PRKCD	PRKCE	9606.ENSP00000378217	9606.ENSP00000306124	0	0	0.8	0.8
PRKCD	PRKDC	9606.ENSP00000378217	9606.ENSP00000313420	0.091	0.525	0	0.55
PRKCD	SPHK1	9606.ENSP00000378217	9606.ENSP00000313681	0	0.105	0.9	0.906
PRKCD	RAC1	9606.ENSP00000378217	9606.ENSP00000348461	0.062	0.425	0	0.438
PRKCD	SRC	9606.ENSP00000378217	9606.ENSP00000362680	0.091	0.504	0.9	0.951
PRKCD	PRKCZ	9606.ENSP00000378217	9606.ENSP00000367830	0.063	0.275	0.9	0.926
PRKCD	TBXA2R	9606.ENSP00000378217	9606.ENSP00000393333	0	0	0.9	0.9
PRKCD	YES1	9606.ENSP00000378217	9606.ENSP00000462468	0.063	0.117	0.9	0.91
PRKCD	RELA	9606.ENSP00000378217	9606.ENSP00000384273	0.062	0	0.9	0.902
PRKCD	PTK2B	9606.ENSP00000378217	9606.ENSP00000380638	0.081	0.444	0.9	0.944
PRKCE	SPHK2	9606.ENSP00000306124	9606.ENSP00000245222	0	0	0.8	0.8
PRKCE	RAF1	9606.ENSP00000306124	9606.ENSP00000251849	0.066	0.327	0.8	0.863
PRKCE	TRPA1	9606.ENSP00000306124	9606.ENSP00000262209	0.062	0.104	0.8	0.817
PRKCE	PRKCQ	9606.ENSP00000306124	9606.ENSP00000263125	0	0	0.8	0.8
PRKCE	PRKCG	9606.ENSP00000306124	9606.ENSP00000263431	0.077	0	0.5	0.519
PRKCE	SPHK1	9606.ENSP00000306124	9606.ENSP00000313681	0	0	0.8	0.8
PRKCE	TRPV1	9606.ENSP00000306124	9606.ENSP00000459962	0	0	0.8	0.8
PRKCE	TBXA2R	9606.ENSP00000306124	9606.ENSP00000393333	0	0	0.9	0.9
PRKCE	PTK2B	9606.ENSP00000306124	9606.ENSP00000380638	0.09	0.153	0.9	0.916
PRKCE	SRC	9606.ENSP00000306124	9606.ENSP00000362680	0.063	0.477	0.9	0.946

PRKCG	SPHK2	9606.ENSP00000263431	9606.ENSP00000245222	0.062	0	0.8	0.804
PRKCG	RAF1	9606.ENSP00000263431	9606.ENSP00000251849	0	0.061	0.8	0.804
PRKCG	RARA	9606.ENSP00000263431	9606.ENSP00000254066	0	0	0.9	0.9
PRKCG	PRKCQ	9606.ENSP00000263431	9606.ENSP00000263125	0	0	0.5	0.499
PRKCG	PRKCH	9606.ENSP00000263431	9606.ENSP00000329127	0	0	0.5	0.499
PRKCG	TOP2A	9606.ENSP00000263431	9606.ENSP00000411532	0	0.574	0	0.574
PRKCG	PTK2	9606.ENSP00000263431	9606.ENSP00000341189	0	0	0.8	0.8
PRKCG	SLC6A3	9606.ENSP00000263431	9606.ENSP00000270349	0	0	0.8	0.8
PRKCG	SPHK1	9606.ENSP00000263431	9606.ENSP00000313681	0.062	0	0.8	0.804
PRKCG	RXRG	9606.ENSP00000263431	9606.ENSP00000352900	0	0	0.9	0.9
PRKCG	RXRA	9606.ENSP00000263431	9606.ENSP00000419692	0	0	0.9	0.9
PRKCG	RXRB	9606.ENSP00000263431	9606.ENSP00000363817	0	0	0.9	0.9
PRKCG	TBXA2R	9606.ENSP00000263431	9606.ENSP00000393333	0	0.056	0.9	0.901
PRKCG	PTK2B	9606.ENSP00000263431	9606.ENSP00000380638	0	0	0.9	0.9
PRKCG	SRC	9606.ENSP00000263431	9606.ENSP00000362680	0	0.061	0.9	0.902
PRKCH	RAF1	9606.ENSP00000329127	9606.ENSP00000251849	0.066	0.117	0.6	0.641
PRKCH	TBXA2R	9606.ENSP00000329127	9606.ENSP00000393333	0	0	0.9	0.9
PRKCH	PTK2B	9606.ENSP00000329127	9606.ENSP00000380638	0.056	0.153	0.9	0.913
PRKCH	SRC	9606.ENSP00000329127	9606.ENSP00000362680	0.063	0.354	0.9	0.934
PRKCQ	RAF1	9606.ENSP00000263125	9606.ENSP00000251849	0.066	0.117	0.6	0.641
PRKCQ	PTPN6	9606.ENSP00000263125	9606.ENSP00000391592	0.085	0.058	0.8	0.812
PRKCQ	RELA	9606.ENSP00000263125	9606.ENSP00000384273	0.049	0	0.8	0.801
PRKCQ	PTPN11	9606.ENSP00000263125	9606.ENSP00000340944	0.062	0.058	0.8	0.807
PRKCQ	TBXA2R	9606.ENSP00000263125	9606.ENSP00000393333	0	0	0.9	0.9
PRKCQ	PTK2B	9606.ENSP00000263125	9606.ENSP00000380638	0.056	0.169	0.9	0.914
PRKCQ	SRC	9606.ENSP00000263125	9606.ENSP00000362680	0.063	0.117	0.9	0.91
PRKCQ	RASGRP1	9606.ENSP00000263125	9606.ENSP00000310244	0.049	0	0.9	0.9
PRKCZ	TNFRSF1A	9606.ENSP00000367830	9606.ENSP00000162749	0	0.063	0.9	0.902
PRKCZ	RIPK2	9606.ENSP00000367830	9606.ENSP00000220751	0	0	0.9	0.9
PRKCZ	RAF1	9606.ENSP00000367830	9606.ENSP00000251849	0	0.27	0.9	0.923
PRKCZ	VHL	9606.ENSP00000367830	9606.ENSP00000256474	0	0.709	0	0.709
PRKCZ	SPHK1	9606.ENSP00000367830	9606.ENSP00000313681	0	0.056	0.9	0.901
PRKCZ	ROCK2	9606.ENSP00000367830	9606.ENSP00000317985	0	0	0.9	0.9
PRKCZ	PTPN11	9606.ENSP00000367830	9606.ENSP00000340944	0.082	0.078	0.9	0.907
PRKCZ	SRC	9606.ENSP00000367830	9606.ENSP00000362680	0	0.281	0.9	0.925
PRKCZ	TGFBR1	9606.ENSP00000367830	9606.ENSP00000364133	0.062	0	0.6	0.608
PRKCZ	ROCK1	9606.ENSP00000367830	9606.ENSP00000382697	0	0	0.9	0.9
PRKCZ	TBXA2R	9606.ENSP00000367830	9606.ENSP00000393333	0	0.056	0.9	0.901
PRKCZ	TNF	9606.ENSP00000367830	9606.ENSP00000398698	0	0.056	0.9	0.901
PRKCZ	RELA	9606.ENSP00000367830	9606.ENSP00000384273	0	0.68	0.9	0.966
PRKDC	TOP1	9606.ENSP00000313420	9606.ENSP00000354522	0.083	0.498	0	0.52
PRKDC	TDP2	9606.ENSP00000313420	9606.ENSP00000367440	0.08	0	0.9	0.904
PRKDC	TDP1	9606.ENSP00000313420	9606.ENSP00000337353	0.079	0	0.9	0.904
PSEN1	PSEN2	9606.ENSP00000326366	9606.ENSP00000355747	0	0.501	0.8	0.896

PSEN1	PSENEN	9606.ENSP00000326366	9606.ENSP00000468411	0.062	0.982	0.9	0.998
PSEN2	PSENEN	9606.ENSP00000355747	9606.ENSP00000468411	0.062	0.908	0.8	0.981
PSMB5	UBLCP1	9606.ENSP00000355325	9606.ENSP00000296786	0.265	0.249	0	0.425
PSMB5	SHH	9606.ENSP00000355325	9606.ENSP00000297261	0	0	0.9	0.9
PSMB5	VCP	9606.ENSP00000355325	9606.ENSP00000351777	0.199	0.35	0	0.457
PSMB5	RELA	9606.ENSP00000355325	9606.ENSP00000384273	0	0	0.9	0.9
PSMB5	PSMB8	9606.ENSP00000355325	9606.ENSP00000364016	0	0.708	0.8	0.939
PSMB8	UBLCP1	9606.ENSP00000364016	9606.ENSP00000296786	0.265	0.249	0	0.424
PSMB8	SHH	9606.ENSP00000364016	9606.ENSP00000297261	0	0	0.9	0.9
PSMB8	VCP	9606.ENSP00000364016	9606.ENSP00000351777	0.168	0.365	0	0.449
PSMB8	RELA	9606.ENSP00000364016	9606.ENSP00000384273	0.062	0	0.9	0.902
PTAFR	STAT3	9606.ENSP00000362965	9606.ENSP00000264657	0	0	0.65	0.65
PTGDR	PTGER2	9606.ENSP00000303424	9606.ENSP00000245457	0.076	0	0.5	0.518
PTGER1	PTGFR	9606.ENSP00000292513	9606.ENSP00000359793	0	0.213	0.3	0.425
PTGES	PTGS1	9606.ENSP00000342385	9606.ENSP00000354612	0.062	0	0.9	0.902
PTGES	PTGS2	9606.ENSP00000342385	9606.ENSP00000356438	0.063	0	0.9	0.902
PTGS1	PTGS2	9606.ENSP00000354612	9606.ENSP00000356438	0.14	0.27	0.8	0.863
PTGS1	TBXAS1	9606.ENSP00000354612	9606.ENSP00000389414	0.091	0	0.9	0.905
PTGS2	STAT3	9606.ENSP00000356438	9606.ENSP00000264657	0	0	0.9	0.9
PTGS2	TBXAS1	9606.ENSP00000356438	9606.ENSP00000389414	0.073	0	0.9	0.903
PTK2	RPS6KB1	9606.ENSP00000341189	9606.ENSP00000225577	0	0	0.9	0.9
PTK2	STAT3	9606.ENSP00000341189	9606.ENSP00000264657	0	0.678	0	0.678
PTK2	ROCK2	9606.ENSP00000341189	9606.ENSP00000317985	0	0.078	0.9	0.903
PTK2	PTPN11	9606.ENSP00000341189	9606.ENSP00000340944	0.064	0.869	0.9	0.986
PTK2	ROCK1	9606.ENSP00000341189	9606.ENSP00000382697	0	0.243	0.9	0.921
PTK2	PTK2B	9606.ENSP00000341189	9606.ENSP00000380638	0	0.409	0.9	0.938
PTK2	RAC1	9606.ENSP00000341189	9606.ENSP00000348461	0.065	0.13	0.9	0.911
PTK2	SYK	9606.ENSP00000341189	9606.ENSP00000364907	0.07	0.505	0.9	0.95
PTK2	RET	9606.ENSP00000341189	9606.ENSP00000347942	0	0.749	0.9	0.973
PTK2	YES1	9606.ENSP00000341189	9606.ENSP00000462468	0.09	0.836	0.9	0.983
PTK2	SRC	9606.ENSP00000341189	9606.ENSP00000362680	0.062	0.931	0.9	0.993
PTK2B	PTPN11	9606.ENSP00000380638	9606.ENSP00000340944	0.064	0.311	0.9	0.929
PTK2B	RAC1	9606.ENSP00000380638	9606.ENSP00000348461	0.065	0	0.9	0.902
PTK2B	SRC	9606.ENSP00000380638	9606.ENSP00000362680	0.062	0.789	0.9	0.978
PTK2B	SYK	9606.ENSP00000380638	9606.ENSP00000364907	0.097	0.298	0.9	0.931
PTK2B	PTPN6	9606.ENSP00000380638	9606.ENSP00000391592	0.121	0.494	0	0.536
PTK2B	YES1	9606.ENSP00000380638	9606.ENSP00000462468	0.062	0.184	0.9	0.916
PTPN1	STAT3	9606.ENSP00000360683	9606.ENSP00000264657	0.066	0.499	0	0.512
PTPN1	PTPN11	9606.ENSP00000360683	9606.ENSP00000340944	0.097	0.139	0.8	0.83
PTPN1	PTPRF	9606.ENSP00000360683	9606.ENSP00000353030	0	0.212	0.8	0.835
PTPN1	TYK2	9606.ENSP00000360683	9606.ENSP00000431885	0.062	0.298	0.9	0.928
PTPN1	SRC	9606.ENSP00000360683	9606.ENSP00000362680	0.086	0.875	0.9	0.987
PTPN11	STAT3	9606.ENSP00000340944	9606.ENSP00000264657	0.062	0.497	0.6	0.794
PTPN11	PTPN7	9606.ENSP00000340944	9606.ENSP00000309116	0.062	0	0.9	0.902

PTPN11	PTPN2	9606.ENSP00000340944	9606.ENSP00000311857	0.065	0.154	0.9	0.913
PTPN11	YES1	9606.ENSP00000340944	9606.ENSP00000462468	0.064	0.246	0.6	0.693
PTPN11	PTPRF	9606.ENSP00000340944	9606.ENSP00000353030	0.051	0.141	0.8	0.822
PTPN11	PTPN6	9606.ENSP00000340944	9606.ENSP00000391592	0	0.273	0.9	0.924
PTPN11	TYK2	9606.ENSP00000340944	9606.ENSP00000431885	0.062	0.298	0.8	0.856
PTPN11	SYK	9606.ENSP00000340944	9606.ENSP00000364907	0.062	0.078	0.9	0.905
PTPN11	RET	9606.ENSP00000340944	9606.ENSP00000347942	0.063	0.228	0.9	0.921
PTPN11	SRC	9606.ENSP00000340944	9606.ENSP00000362680	0.064	0.747	0.9	0.974
PTPN2	STAT3	9606.ENSP00000311857	9606.ENSP00000264657	0.066	0.771	0.9	0.976
PTPN2	STAT6	9606.ENSP00000311857	9606.ENSP00000300134	0.066	0.285	0.9	0.927
PTPN2	PTPN7	9606.ENSP00000311857	9606.ENSP00000309116	0.064	0	0.9	0.902
PTPN2	TYK2	9606.ENSP00000311857	9606.ENSP00000431885	0.062	0.078	0.8	0.811
PTPN2	SRC	9606.ENSP00000311857	9606.ENSP00000362680	0.063	0.298	0.9	0.928
PTPN2	PTPN6	9606.ENSP00000311857	9606.ENSP00000391592	0.062	0.735	0.9	0.972
PTPN6	STAT6	9606.ENSP00000391592	9606.ENSP00000300134	0.097	0.23	0.9	0.924
PTPN6	PTPN7	9606.ENSP00000391592	9606.ENSP00000309116	0.155	0	0.9	0.911
PTPN6	SRC	9606.ENSP00000391592	9606.ENSP00000362680	0.062	0.691	0	0.698
PTPN6	SYK	9606.ENSP00000391592	9606.ENSP00000364907	0.23	0.87	0.9	0.989
PTPN6	PTPRC	9606.ENSP00000391592	9606.ENSP00000411355	0.221	0.295	0	0.427
PTPN6	TYK2	9606.ENSP00000391592	9606.ENSP00000431885	0.089	0.482	0.9	0.948
PTPRC	S1PR4	9606.ENSP00000411355	9606.ENSP00000246115	0.56	0	0	0.56
PTPRC	TLR8	9606.ENSP00000411355	9606.ENSP00000312082	0.346	0.148	0	0.419
PTPRC	TLR1	9606.ENSP00000411355	9606.ENSP00000354932	0.351	0.148	0	0.424
RAC1	RPS6KB1	9606.ENSP00000348461	9606.ENSP00000225577	0	0.302	0.9	0.927
RAC1	STAT3	9606.ENSP00000348461	9606.ENSP00000264657	0	0.282	0.9	0.925
RAC1	S1PR1	9606.ENSP00000348461	9606.ENSP00000305416	0.062	0	0.9	0.902
RAC1	ROCK2	9606.ENSP00000348461	9606.ENSP00000317985	0	0.182	0.9	0.914
RAC1	TBXA2R	9606.ENSP00000348461	9606.ENSP00000393333	0	0.056	0.9	0.901
RAC1	S1PR3	9606.ENSP00000348461	9606.ENSP00000365006	0	0	0.9	0.9
RAC1	YES1	9606.ENSP00000348461	9606.ENSP00000462468	0	0.14	0.9	0.91
RAC1	SYK	9606.ENSP00000348461	9606.ENSP00000364907	0.066	0.187	0.9	0.917
RAC1	SRC	9606.ENSP00000348461	9606.ENSP00000362680	0	0.345	0.9	0.931
RAD52	TERT	9606.ENSP00000351284	9606.ENSP00000309572	0	0.569	0	0.569
RAF1	STK3	9606.ENSP00000251849	9606.ENSP00000429744	0.051	0.76	0	0.762
RAF1	RELA	9606.ENSP00000251849	9606.ENSP00000384273	0.112	0.056	0.8	0.817
RAF1	SRC	9606.ENSP00000251849	9606.ENSP00000362680	0	0.62	0.8	0.92
RAF1	YWHAG	9606.ENSP00000251849	9606.ENSP00000306330	0	0.888	0.9	0.988
RARA	SRC	9606.ENSP00000254066	9606.ENSP00000362680	0.077	0.488	0	0.508
RARA	THRA	9606.ENSP00000254066	9606.ENSP00000264637	0.07	0	0.9	0.903
RARA	VDR	9606.ENSP00000254066	9606.ENSP00000447173	0	0	0.9	0.9
RARA	THRB	9606.ENSP00000254066	9606.ENSP00000379904	0	0.213	0.9	0.917
RARA	RARB	9606.ENSP00000254066	9606.ENSP00000332296	0	0.475	0.9	0.945
RARA	RARG	9606.ENSP00000254066	9606.ENSP00000388510	0.081	0.674	0.9	0.967
RARA	RXRG	9606.ENSP00000254066	9606.ENSP00000352900	0.062	0.88	0.9	0.987

RARA	RXRB	9606.ENSP00000254066	9606.ENSP00000363817	0.088	0.875	0.9	0.987
RARA	RXRA	9606.ENSP00000254066	9606.ENSP00000419692	0.095	0.982	0.9	0.998
RARB	THRA	9606.ENSP00000332296	9606.ENSP00000264637	0	0	0.9	0.9
RARB	RARG	9606.ENSP00000332296	9606.ENSP00000388510	0	0	0.9	0.9
RARB	THRB	9606.ENSP00000332296	9606.ENSP00000379904	0	0	0.9	0.9
RARB	VDR	9606.ENSP00000332296	9606.ENSP00000447173	0	0	0.9	0.9
RARB	RXRG	9606.ENSP00000332296	9606.ENSP00000352900	0.063	0.675	0.9	0.966
RARB	RXRB	9606.ENSP00000332296	9606.ENSP00000363817	0.052	0.749	0.9	0.974
RARB	RXRA	9606.ENSP00000332296	9606.ENSP00000419692	0.062	0.955	0.9	0.995
RARG	THRA	9606.ENSP00000388510	9606.ENSP00000264637	0	0	0.9	0.9
RARG	RXRG	9606.ENSP00000388510	9606.ENSP00000352900	0.062	0.489	0.9	0.947
RARG	RXRB	9606.ENSP00000388510	9606.ENSP00000363817	0.062	0.754	0.9	0.974
RARG	THRB	9606.ENSP00000388510	9606.ENSP00000379904	0	0	0.9	0.9
RARG	VDR	9606.ENSP00000388510	9606.ENSP00000447173	0.062	0	0.9	0.902
RARG	RXRA	9606.ENSP00000388510	9606.ENSP00000419692	0.085	0.351	0.9	0.935
RBP4	TTR	9606.ENSP00000360522	9606.ENSP00000237014	0.25	0.951	0.6	0.984
RELA	TNFRSF1A	9606.ENSP00000384273	9606.ENSP00000162749	0.065	0	0.9	0.902
RELA	TLR2	9606.ENSP00000384273	9606.ENSP00000260010	0.062	0.06	0.8	0.808
RELA	STAT3	9606.ENSP00000384273	9606.ENSP00000264657	0.108	0.835	0	0.846
RELA	ROCK2	9606.ENSP00000384273	9606.ENSP00000317985	0	0.056	0.8	0.803
RELA	TGM2	9606.ENSP00000384273	9606.ENSP00000355330	0.062	0.486	0	0.497
RELA	SRC	9606.ENSP00000384273	9606.ENSP00000362680	0.063	0.056	0.9	0.903
RELA	SYK	9606.ENSP00000384273	9606.ENSP00000364907	0.062	0.056	0.9	0.903
RELA	TDP2	9606.ENSP00000384273	9606.ENSP00000367440	0.056	0	0.9	0.901
RELA	ROCK1	9606.ENSP00000384273	9606.ENSP00000382697	0	0.056	0.8	0.803
RELA	RXRA	9606.ENSP00000384273	9606.ENSP00000419692	0.09	0.732	0	0.746
RELA	TYK2	9606.ENSP00000384273	9606.ENSP00000431885	0.065	0.056	0.9	0.904
RELA	SMARCA4	9606.ENSP00000384273	9606.ENSP00000395654	0.083	0	0.9	0.904
RELA	XPO1	9606.ENSP00000384273	9606.ENSP00000384863	0.06	0	0.9	0.901
RELA	RPS6KA5	9606.ENSP00000384273	9606.ENSP00000479667	0	0.632	0.9	0.961
RELA	TNF	9606.ENSP00000384273	9606.ENSP00000398698	0	0.501	0.9	0.948
RET	STAT3	9606.ENSP00000347942	9606.ENSP00000264657	0	0.779	0	0.779
RET	SRC	9606.ENSP00000347942	9606.ENSP00000362680	0.062	0.436	0.9	0.942
ROCK1	ROCK2	9606.ENSP00000382697	9606.ENSP00000317985	0.084	0.684	0.9	0.968
ROCK1	SRC	9606.ENSP00000382697	9606.ENSP00000362680	0	0	0.9	0.9
ROCK2	SRC	9606.ENSP00000317985	9606.ENSP00000362680	0	0	0.9	0.9
RORA	RORB	9606.ENSP00000261523	9606.ENSP00000366093	0	0	0.8	0.8
RORA	RORC	9606.ENSP00000261523	9606.ENSP00000327025	0.062	0	0.9	0.902
RORA	STAT3	9606.ENSP00000261523	9606.ENSP00000264657	0	0.059	0.9	0.901
RORB	RORC	9606.ENSP00000366093	9606.ENSP00000327025	0.049	0.308	0.8	0.856
RORC	STAT3	9606.ENSP00000327025	9606.ENSP00000264657	0	0.345	0.9	0.931
RPS6KA3	RPS6KA5	9606.ENSP00000368884	9606.ENSP00000479667	0	0	0.9	0.9
RPS6KB1	RXRG	9606.ENSP00000225577	9606.ENSP00000352900	0.054	0.068	0.9	0.904
RPS6KB1	RXRB	9606.ENSP00000225577	9606.ENSP00000363817	0.062	0.068	0.9	0.904

RPS6KB1	RXRA	9606.ENSP00000225577	9606.ENSP00000419692	0.062	0.068	0.9	0.904
RPS6KB1	TGFBR1	9606.ENSP00000225577	9606.ENSP00000364133	0.056	0.15	0.9	0.912
RPS6KB1	TERT	9606.ENSP00000225577	9606.ENSP00000309572	0.062	0.297	0.9	0.928
RXRA	THRA	9606.ENSP00000419692	9606.ENSP00000264637	0.062	0.873	0.8	0.974
RXRA	RXRG	9606.ENSP00000419692	9606.ENSP00000352900	0	0.273	0.9	0.924
RXRA	SCD	9606.ENSP00000419692	9606.ENSP00000359380	0.057	0	0.9	0.901
RXRA	SRC	9606.ENSP00000419692	9606.ENSP00000362680	0.076	0.482	0	0.501
RXRA	RXRB	9606.ENSP00000419692	9606.ENSP00000363817	0.07	0	0.9	0.903
RXRA	THRB	9606.ENSP00000419692	9606.ENSP00000379904	0.053	0.975	0.8	0.994
RXRA	SMARCA4	9606.ENSP00000419692	9606.ENSP00000395654	0.085	0.298	0.9	0.93
RXRA	TNF	9606.ENSP00000419692	9606.ENSP00000398698	0	0	0.9	0.9
RXRA	VDR	9606.ENSP00000419692	9606.ENSP00000447173	0	0.981	0.9	0.998
RXRB	THRA	9606.ENSP00000363817	9606.ENSP00000264637	0.096	0.317	0.8	0.865
RXRB	RXRG	9606.ENSP00000363817	9606.ENSP00000352900	0	0	0.9	0.9
RXRB	SCD	9606.ENSP00000363817	9606.ENSP00000359380	0	0	0.9	0.9
RXRB	THRB	9606.ENSP00000363817	9606.ENSP00000379904	0	0.244	0.8	0.842
RXRB	VDR	9606.ENSP00000363817	9606.ENSP00000447173	0	0.241	0.9	0.92
RXRG	THRA	9606.ENSP00000352900	9606.ENSP00000264637	0.065	0.317	0.8	0.861
RXRG	THRB	9606.ENSP00000352900	9606.ENSP00000379904	0	0.384	0.8	0.871
RXRG	VDR	9606.ENSP00000352900	9606.ENSP00000447173	0	0.472	0.9	0.944
S1PR1	S1PR4	9606.ENSP00000305416	9606.ENSP00000246115	0.11	0	0.8	0.814
S1PR1	STAT3	9606.ENSP00000305416	9606.ENSP00000264657	0	0	0.9	0.9
S1PR1	YES1	9606.ENSP00000305416	9606.ENSP00000462468	0	0.147	0.9	0.911
S1PR1	SRC	9606.ENSP00000305416	9606.ENSP00000362680	0	0.241	0.9	0.92
S1PR3	SRC	9606.ENSP00000365006	9606.ENSP00000362680	0	0	0.9	0.9
SAE1	UBA2	9606.ENSP00000270225	9606.ENSP00000246548	0.732	0.995	0.9	0.999
SCD	VCP	9606.ENSP00000359380	9606.ENSP00000351777	0.063	0.498	0	0.51
SCN5A	SCN9A	9606.ENSP00000410257	9606.ENSP00000386306	0	0.403	0.54	0.713
SHH	VCP	9606.ENSP00000297261	9606.ENSP00000351777	0	0	0.6	0.6
SIGMAR1	SQLE	9606.ENSP00000277010	9606.ENSP00000265896	0.072	0.781	0	0.788
SLC18A2	SLC18A3	9606.ENSP00000298472	9606.ENSP00000363229	0.065	0	0.8	0.805
SLC18A3	SLC5A7	9606.ENSP00000363229	9606.ENSP00000264047	0.489	0	0	0.489
SMARCA2	SMARCA4	9606.ENSP00000265773	9606.ENSP00000395654	0.046	0.877	0.9	0.987
SMARCA4	VHL	9606.ENSP00000395654	9606.ENSP00000256474	0	0.8	0	0.8
SMARCA4	TERT	9606.ENSP00000395654	9606.ENSP00000309572	0.063	0.501	0.9	0.949
SMARCA4	TOP2A	9606.ENSP00000395654	9606.ENSP00000411532	0.19	0.363	0	0.462
SMARCA4	VDR	9606.ENSP00000395654	9606.ENSP00000447173	0	0.078	0.9	0.903
SOAT1	STS	9606.ENSP00000356591	9606.ENSP00000217961	0	0	0.8	0.8
SPHK1	SPHK2	9606.ENSP00000313681	9606.ENSP00000245222	0	0	0.8	0.8
SPHK1	SYK	9606.ENSP00000313681	9606.ENSP00000364907	0	0.056	0.9	0.901
SPHK2	TYK2	9606.ENSP00000245222	9606.ENSP00000431885	0	0.056	0.9	0.901
SPTLC1	SPTLC2	9606.ENSP00000262554	9606.ENSP00000216484	0.264	0.88	0.9	0.99
SQLE	SREBF2	9606.ENSP00000265896	9606.ENSP00000354476	0.137	0	0.9	0.91
SRC	TNFRSF1A	9606.ENSP00000362680	9606.ENSP00000162749	0	0.682	0	0.682

SRC	STAT3	9606.ENSP00000362680	9606.ENSP00000264657	0.061	0.883	0.9	0.988
SRC	TNK2	9606.ENSP00000362680	9606.ENSP00000371341	0.062	0.838	0	0.842
SRC	VDR	9606.ENSP00000362680	9606.ENSP00000447173	0	0.785	0	0.785
SRC	TRPV1	9606.ENSP00000362680	9606.ENSP00000459962	0	0.056	0.8	0.803
SRC	SYK	9606.ENSP00000362680	9606.ENSP00000364907	0.053	0.675	0.9	0.966
SRC	YES1	9606.ENSP00000362680	9606.ENSP00000462468	0	0.8	0.9	0.979
SRD5A1	UGT2B7	9606.ENSP00000274192	9606.ENSP00000304811	0.063	0	0.65	0.658
SRD5A1	SRD5A2	9606.ENSP00000274192	9606.ENSP00000477587	0	0	0.8	0.8
SRD5A2	UGT2B7	9606.ENSP00000477587	9606.ENSP00000304811	0.062	0	0.65	0.657
STAT3	TNFRSF1A	9606.ENSP00000264657	9606.ENSP00000162749	0.109	0	0.65	0.675
STAT3	STAT6	9606.ENSP00000264657	9606.ENSP00000300134	0.062	0.094	0.8	0.815
STAT3	YES1	9606.ENSP00000264657	9606.ENSP00000462468	0.061	0.14	0.9	0.912
STAT3	TNF	9606.ENSP00000264657	9606.ENSP00000398698	0	0	0.65	0.65
STAT3	SYK	9606.ENSP00000264657	9606.ENSP00000364907	0	0.886	0	0.886
STAT3	TYK2	9606.ENSP00000264657	9606.ENSP00000431885	0.062	0.14	0.9	0.912
STAT6	TYK2	9606.ENSP00000300134	9606.ENSP00000431885	0.085	0.14	0.9	0.914
SYK	TNFRSF1A	9606.ENSP00000364907	9606.ENSP00000162749	0.062	0.478	0	0.489
SYK	TXK	9606.ENSP00000364907	9606.ENSP00000264316	0.098	0	0.5	0.529
SYK	TBXA2R	9606.ENSP00000364907	9606.ENSP00000393333	0	0.056	0.9	0.901
SYK	YES1	9606.ENSP00000364907	9606.ENSP00000462468	0.053	0	0.9	0.901
TAS1R1	TAS2R31	9606.ENSP00000331867	9606.ENSP00000375093	0	0	0.6	0.6
TBXA2R	TGM2	9606.ENSP00000393333	9606.ENSP00000355330	0	0	0.9	0.9
TDP1	VCP	9606.ENSP00000337353	9606.ENSP00000351777	0.052	0.472	0	0.478
TDP1	TOP1	9606.ENSP00000337353	9606.ENSP00000354522	0.101	0.772	0	0.786
TGFBR1	XPO1	9606.ENSP00000364133	9606.ENSP00000384863	0.062	0	0.65	0.657
THRA	THRB	9606.ENSP00000264637	9606.ENSP00000379904	0.062	0	0.9	0.902
TLR1	TLR2	9606.ENSP00000354932	9606.ENSP00000260010	0.508	0.651	0.9	0.981
TLR2	TLR8	9606.ENSP00000260010	9606.ENSP00000312082	0.529	0	0	0.529
TLR2	TLR9	9606.ENSP00000260010	9606.ENSP00000353874	0.076	0	0.8	0.807
TNF	TNFRSF1A	9606.ENSP00000398698	9606.ENSP00000162749	0.062	0.994	0.9	0.999
TNNC1	TNNT2	9606.ENSP00000232975	9606.ENSP00000236918	0.371	0.974	0.9	0.998
TNNC1	TNNI3	9606.ENSP00000232975	9606.ENSP00000341838	0.177	0.981	0.9	0.998
TNNI3	TNNT2	9606.ENSP00000341838	9606.ENSP00000236918	0.65	0.947	0.9	0.997
TOP1	TOP2A	9606.ENSP00000354522	9606.ENSP00000411532	0.111	0.919	0	0.925
TOP2A	UBA2	9606.ENSP00000411532	9606.ENSP00000246548	0.67	0	0	0.67
TOP2A	TYMS	9606.ENSP00000411532	9606.ENSP00000315644	0.83	0	0	0.83
TOP2A	TTK	9606.ENSP00000411532	9606.ENSP00000358813	0.949	0	0	0.949
TOP2A	XPO1	9606.ENSP00000411532	9606.ENSP00000384863	0.267	0.462	0	0.589
TPO	TYR	9606.ENSP00000318820	9606.ENSP00000263321	0.062	0	0.9	0.902
TTK	TYMS	9606.ENSP00000358813	9606.ENSP00000315644	0.816	0	0	0.816
TTL	TUBB1	9606.ENSP00000233336	9606.ENSP00000217133	0.06	0	0.9	0.901
UBLCP1	VCP	9606.ENSP00000296786	9606.ENSP00000351777	0.136	0.368	0	0.43
XPO1	YWHAG	9606.ENSP00000384863	9606.ENSP00000306330	0.083	0	0.9	0.904

Table S4: Hub targets clustered as seven clusters using "Cluster FI Network" in Cytoscape			
TargetName	module	Degree	
S1PR2	0	7	
HTR1E	0	3	
TYK2	0	21	
IGF1R	0	24	
DUSP3	0	5	
PTPN2	0	12	
CNR2	0	2	
PTK2B	0	33	
CHRM4	0	2	
LPAR1	0	21	
LPAR3	0	17	
LPAR2	0	18	
CHRNA4	0	1	
DNM1	0	16	
LRRK2	0	22	
RIPK2	0	8	
PTK2	0	43	
F2R	0	15	
GRM5	0	11	
GRIA2	0	13	
HTR5A	0	8	
SLC6A3	0	4	
ESR2	0	21	
SYK	0	30	
FOS	0	30	
MAP2K1	0	34	
CAMK2A	0	21	
BCL2A1	0	3	
CHRM2	0	8	
CXCL8	0	18	
PPP1CC	0	11	
SPHK1	0	12	
MAPK10	0	25	
GRIN1	0	19	
KDR	0	17	
ITGB2	0	18	
JAK2	0	41	
KIT	0	19	
IKBKG	0	19	

NOS1	0	10	
IKBKB	0	17	
JAK3	0	28	
PRKCZ	0	32	
PIK3CD	0	39	
MTOR	0	24	
PIK3CG	0	28	
PDPK1	0	29	
PTPN11	0	40	
CASP9	0	6	
NOS2	0	15	
GSK3B	0	25	
S1PR1	0	17	
NOS3	0	18	
PIK3CB	0	45	
BRAF	0	19	
STAT3	0	57	
PIK3CA	0	55	
AKT3	0	29	
MAPK3	0	74	
RAF1	0	34	
NFKB1	0	44	
RPS6KB1	0	27	
MAPK1	0	81	
AKT1	0	62	
RELA	0	50	
ADRA2B	0	6	
PRKCD	0	44	
PRKCE	0	31	
ADRA2C	0	8	
PRKCG	0	33	
PRKCQ	0	26	
GNAO1	0	22	
ADRA2A	0	9	
CXCL12	0	28	
F2	0	21	
CHRM1	0	19	
CCR5	0	12	
HTR2C	0	4	
BTK	0	22	
CNR1	0	3	
GNAI3	0	46	

GNAI1	0	52	
PRKCA	0	46	
PRKCB	0	43	
INSR	0	22	
APP	0	14	
ACHE	0	1	
SRC	0	80	
NTRK1	0	18	
HCK	0	31	
PIK3R1	0	62	
LYN	0	55	
FGR	0	33	
YES1	0	29	
PTPN6	0	28	
PTPN1	0	16	
RAC1	0	40	
JAK1	0	35	
LCK	0	47	
HSP90AA1	0	51	
YWHAG	0	17	
EGFR	0	59	
ERBB2	0	23	
PDGFRB	0	29	
MAPK14	0	49	
ABL1	0	30	
MAPK8	0	45	
NDUFAF4	1	38	
NDUFAF3	1	38	
NDUFAF1	1	47	
MT-ND2	1	48	
NDUFA1	1	48	
MT-ND6	1	47	
NDUFA5	1	48	
NDUFA3	1	48	
MT-ND3	1	48	
MT-ND1	1	48	
MT-ND5	1	48	
NDUFC1	1	48	
MT-ND4L	1	46	
MT-ND4	1	48	
NDUFA11	1	48	
NDUFV3	1	46	

NDUFB2	1	48	
NDUFA4	1	45	
NDUFB1	1	48	
NDUFB8	1	48	
NDUFS4	1	46	
NDUFB3	1	48	
NDUFB7	1	48	
NDUFB4	1	48	
NDUFV1	1	46	
APOBEC3G	1	23	
NDUFAB1	1	50	
NDUFA6	1	50	
NDUFA13	1	51	
NDUFS1	1	48	
NDUFB6	1	50	
NDUFA8	1	50	
NDUFS5	1	50	
NDUFS2	1	50	
NDUFA12	1	48	
NDUFV2	1	48	
NDUFS8	1	50	
NDUFA7	1	50	
NDUFC2	1	50	
NDUFB9	1	50	
NDUFB11	1	49	
NDUFS6	1	48	
NDUFB10	1	50	
NDUFA9	1	50	
NDUFS3	1	50	
NDUFB5	1	50	
NDUFA10	1	50	
NDUFA2	1	50	
NDUFS7	1	50	
APOBEC3A	1	23	
MAPT	1	65	
DNMT1	2	9	
POLA1	2	14	
CCNH	2	18	
PARP1	2	15	
CDK9	2	16	
PLK1	2	22	
KIF11	2	20	

AURKB	2	20	
CCNB1	2	31	
CDK1	2	38	
TOP2A	2	22	
CDC25B	2	13	
TTK	2	19	
TYMS	2	15	
FEN1	2	19	
CCNB3	2	20	
CCNE2	2	20	
CCNA1	2	24	
CHEK1	2	25	
PCNA	2	31	
CDC25A	2	25	
CCNB2	2	27	
CDK7	2	20	
PRKDC	2	13	
AURKA	2	26	
CDK5	2	21	
TERT	2	17	
CCNA2	2	32	
CDK2	2	37	
CCNE1	2	23	
MDM2	2	25	
CDK4	2	27	
CCND1	2	37	
PPARD	3	11	
THRA	3	18	
THRB	3	20	
PPARA	3	17	
NR1H3	3	15	
RORA	3	7	
HDAC2	3	27	
PPARG	3	25	
FABP4	3	5	
KDM1A	3	14	
SMARCA4	3	19	
PGR	3	21	
EZH2	3	27	
NCOR1	3	27	
HDAC4	3	13	
KAT2B	3	26	

NR3C1	3	29	
FABP1	3	11	
HIF1A	3	23	
RXRA	3	37	
RARG	3	20	
NR2F2	3	4	
RXRB	3	25	
RXRG	3	24	
RARB	3	18	
HDAC3	3	36	
RARA	3	27	
ESR1	3	44	
AR	3	33	
NCOR2	3	34	
HDAC1	3	42	
JUN	3	43	
EP300	3	60	
HSD11B1	4	4	
CES2	4	3	
CES1	4	3	
PTGS1	4	9	
PTGS2	4	12	
ALOX5	4	12	
CYP2C19	4	13	
ALOX15	4	11	
PLA2G4A	4	22	
PLA2G1B	4	12	
PLA2G4B	4	16	
PLA2G5	4	11	
CYP2C9	4	12	
CYP19A1	4	4	
HSD17B3	4	3	
CYP17A1	4	4	
CYP1A2	4	10	
CYP3A4	4	16	
MAOA	4	3	
MAOB	4	3	
UGT2B7	4	8	
CA4	5	1	
CA14	5	1	
CA5B	6	1	
CA5A	6	1	

Table S5: SARS-CoV-2 proteins interacting with hub target genes of ginger compounds using BioGRID database			
**ViralProtein**	**HumanProtein**	**Tech**	
E	APP	High Throughput	
E	PTPN1	High Throughput	
E	PTPN2	High Throughput	
E	SRC	High Throughput	
E	YES1	High Throughput	
E	PRKCD	High Throughput	
E	PTPN1	High Throughput	
E	PTPN2	High Throughput	
E	RAC1	High Throughput	
E	NDUFAF4	High Throughput	
E	NDUFA9	High Throughput	
E	NDUFA10	High Throughput	
E	NDUFS1	High Throughput	
E	NDUFS2	High Throughput	
E	NDUFV2	High Throughput	
E	NDUFV3	High Throughput	
E	NDUFAF3	High Throughput	
E	NDUFAF4	High Throughput	
E	NDUFB11	High Throughput	
E	NDUFS2	High Throughput	
E	NDUFS3	High Throughput	
E	CES1	High Throughput	
M	JAK2	High Throughput	
M	APP	High Throughput	
M	JAK1	High Throughput	
M	PRKCA	High Throughput	
M	PRKCQ	High Throughput	
M	PTPN1	High Throughput	
M	PTPN2	High Throughput	
M	RAC1	High Throughput	
M	SRC	High Throughput	
M	EGFR	High Throughput	
M	ERBB2	High Throughput	
M	IGF1R	High Throughput	
M	LYN	High Throughput	
M	PIK3CB	High Throughput	
M	PRKCD	High Throughput	
M	SRC	High Throughput	

M	YES1	High Throughput	
M	IGF1R	High Throughput	
M	INSR	High Throughput	
M	PTPN1	High Throughput	
M	PTPN2	High Throughput	
M	RAC1	High Throughput	
M	NDUFS2	High Throughput	
M	NDUFS8	High Throughput	
M	NDUFA10	High Throughput	
M	NDUFA11	High Throughput	
M	NDUFAF3	High Throughput	
M	NDUFAF4	High Throughput	
M	NDUFB11	High Throughput	
M	PRKDC	High Throughput	
M	CDK5	High Throughput	
M	KDM1A	High Throughput	
M	NR2F2	High Throughput	
M	CES1	High Throughput	
M	PLA2G4A	High Throughput	
N	GSK3B	High Throughput	
N	GSK3B	High Throughput	
N	GSK3B	Low Throughput	
nsp1	POLA1	High Throughput	
nsp1	POLA1	High Throughput	
nsp1	POLA1	High Throughput	
nsp1	POLA1	High Throughput	
nsp13	RAC1	High Throughput	
nsp13	SRC	High Throughput	
nsp13	CCNB1	High Throughput	
nsp13	CES1	High Throughput	
nsp14	SRC	High Throughput	
nsp14	JUN	High Throughput	
nsp14	CES1	High Throughput	
nsp15	TYMS	High Throughput	
nsp16	NFKB1	High Throughput	
nsp16	NFKB1	High Throughput	
nsp16	NDUFA10	High Throughput	
nsp16	NDUFS1	High Throughput	
nsp16	NDUFS2	High Throughput	
nsp16	NDUFV2	High Throughput	
nsp16	NDUFV3	High Throughput	
nsp16	CDK5	High Throughput	

nsp2	AKT1	High Throughput	
nsp2	PPP1CC	High Throughput	
nsp2	NR2F2	High Throughput	
nsp3	NDUFS1	High Throughput	
nsp3	CCNB1	High Throughput	
nsp3	CCNB2	High Throughput	
nsp3	TTK	High Throughput	
nsp3	CCNB2	High Throughput	
nsp4	EGFR	High Throughput	
nsp4	ERBB2	High Throughput	
nsp4	APP	High Throughput	
nsp4	PRKCA	High Throughput	
nsp4	PRKCD	High Throughput	
nsp4	PTPN1	High Throughput	
nsp4	PTPN2	High Throughput	
nsp4	RAC1	High Throughput	
nsp4	SRC	High Throughput	
nsp4	YES1	High Throughput	
nsp4	EGFR	High Throughput	
nsp4	IGF1R	High Throughput	
nsp4	LYN	High Throughput	
nsp4	PRKCD	High Throughput	
nsp4	PRKCQ	High Throughput	
nsp4	SRC	High Throughput	
nsp4	YES1	High Throughput	
nsp4	APP	High Throughput	
nsp4	PRKCD	High Throughput	
nsp4	PTPN1	High Throughput	
nsp4	PTPN2	High Throughput	
nsp4	SRC	High Throughput	
nsp4	NDUFAF4	High Throughput	
nsp4	CCNB2	High Throughput	
nsp4	CES1	High Throughput	
nsp5	PPP1CC	High Throughput	
nsp5	SRC	High Throughput	
nsp5	HDAC2	High Throughput	
nsp5	HDAC2	High Throughput	
nsp5	CES1	High Throughput	
nsp6	APP	High Throughput	
nsp6	PRKCA	High Throughput	
nsp6	PRKCD	High Throughput	
nsp6	PRKCQ	High Throughput	

nsp6	PTPN1	High Throughput	
nsp6	PTPN2	High Throughput	
nsp6	RAC1	High Throughput	
nsp6	SRC	High Throughput	
nsp6	PRKCD	High Throughput	
nsp6	SRC	High Throughput	
nsp6	PRKCD	High Throughput	
nsp6	PTPN1	High Throughput	
nsp6	PTPN2	High Throughput	
nsp6	SRC	High Throughput	
nsp6	NDUFA12	High Throughput	
nsp6	NDUFA2	High Throughput	
nsp6	NDUFA9	High Throughput	
nsp6	NDUFB8	High Throughput	
nsp6	NDUFS1	High Throughput	
nsp6	NDUFS2	High Throughput	
nsp6	NDUFS3	High Throughput	
nsp6	NDUFS7	High Throughput	
nsp6	NDUFV1	High Throughput	
nsp6	NDUFV2	High Throughput	
nsp6	NDUFA5	High Throughput	
nsp6	NDUFB8	High Throughput	
nsp6	NDUFS1	High Throughput	
nsp6	NDUFS2	High Throughput	
nsp6	NDUFS3	High Throughput	
nsp6	NDUFV1	High Throughput	
nsp6	NDUFV2	High Throughput	
nsp6	CES1	High Throughput	
nsp6	PLA2G4A	High Throughput	
nsp7	JAK1	High Throughput	
nsp7	PTPN1	High Throughput	
nsp7	CDK9	High Throughput	
nsp7	PRKDC	High Throughput	
nsp7	NCOR2	High Throughput	
nsp8	PRKDC	High Throughput	
nsp9	NDUFS1	High Throughput	
nsp9	NDUFS5	High Throughput	
nsp9	NDUFS8	High Throughput	
ORF10	NDUFS5	High Throughput	
ORF14	PTPN1	High Throughput	
ORF14	LYN	High Throughput	
ORF14	MAPK1	High Throughput	

ORF14	NDUFA12	High Throughput	
ORF14	NDUFA2	High Throughput	
ORF14	NDUFA5	High Throughput	
ORF14	NDUFA9	High Throughput	
ORF14	NDUFB10	High Throughput	
ORF14	NDUFS1	High Throughput	
ORF14	NDUFS3	High Throughput	
ORF14	NDUFS7	High Throughput	
ORF14	NDUFS8	High Throughput	
ORF14	NDUFV1	High Throughput	
ORF14	NDUFV2	High Throughput	
ORF14	PARP1	High Throughput	
ORF14	CDK1	High Throughput	
ORF14	PCNA	High Throughput	
ORF14	TOP2A	High Throughput	
ORF14	NR2F2	High Throughput	
ORF3a	PTPN11	High Throughput	
ORF3a	ERBB2	High Throughput	
ORF3a	JAK1	High Throughput	
ORF3a	PTPN1	High Throughput	
ORF3a	RAC1	High Throughput	
ORF3a	SRC	High Throughput	
ORF3a	EGFR	High Throughput	
ORF3a	ERBB2	High Throughput	
ORF3a	LYN	High Throughput	
ORF3a	SRC	High Throughput	
ORF3a	YES1	High Throughput	
ORF3a	MAPK1	High Throughput	
ORF3a	RAC1	High Throughput	
ORF3a	CES1	High Throughput	
ORF3b	APP	High Throughput	
ORF3b	JAK1	High Throughput	
ORF3b	SRC	High Throughput	
ORF3b	YES1	High Throughput	
ORF3b	LYN	High Throughput	
ORF3b	YES1	High Throughput	
ORF3b	LYN	High Throughput	
ORF3b	RAC1	High Throughput	
ORF3b	SRC	High Throughput	
ORF3b	YES1	High Throughput	
ORF3b	NDUFA10	High Throughput	
ORF3b	NDUFS1	High Throughput	

ORF3b	NDUFV2	High Throughput	
ORF3b	NDUFV3	High Throughput	
ORF3b	CES1	High Throughput	
ORF6	PTPN1	High Throughput	
ORF6	RAC1	High Throughput	
ORF6	SRC	High Throughput	
ORF6	PTPN1	High Throughput	
ORF6	RAC1	High Throughput	
ORF6	NDUFS1	High Throughput	
ORF6	NDUFS2	High Throughput	
ORF6	NDUFV2	High Throughput	
ORF6	NDUFV3	High Throughput	
ORF6	KIF11	High Throughput	
ORF6	PRKDC	High Throughput	
ORF6	KIF11	High Throughput	
ORF6	CES1	High Throughput	
ORF7a	ERBB2	High Throughput	
ORF7a	APP	High Throughput	
ORF7a	PRKCA	High Throughput	
ORF7a	PRKCD	High Throughput	
ORF7a	PTPN1	High Throughput	
ORF7a	PTPN2	High Throughput	
ORF7a	RAC1	High Throughput	
ORF7a	SRC	High Throughput	
ORF7a	PRKCD	High Throughput	
ORF7a	PRKCQ	High Throughput	
ORF7a	SRC	High Throughput	
ORF7a	PTPN1	High Throughput	
ORF7a	PTPN2	High Throughput	
ORF7a	RAC1	High Throughput	
ORF7a	SRC	High Throughput	
ORF7a	NDUFA12	High Throughput	
ORF7a	NDUFA12	High Throughput	
ORF7a	NDUFA2	High Throughput	
ORF7a	NDUFA9	High Throughput	
ORF7a	NDUFV1	High Throughput	
ORF7a	NDUFV1	High Throughput	
ORF7a	CES1	High Throughput	
ORF7b	ERBB2	High Throughput	
ORF7b	LPAR1	High Throughput	
ORF7b	PTPN11	High Throughput	
ORF7b	EGFR	High Throughput	

ORF7b	ERBB2	High Throughput	
ORF7b	APP	High Throughput	
ORF7b	JAK1	High Throughput	
ORF7b	PPP1CC	High Throughput	
ORF7b	PRKCA	High Throughput	
ORF7b	PRKCD	High Throughput	
ORF7b	PRKCQ	High Throughput	
ORF7b	PTPN1	High Throughput	
ORF7b	PTPN2	High Throughput	
ORF7b	RAC1	High Throughput	
ORF7b	SRC	High Throughput	
ORF7b	YES1	High Throughput	
ORF7b	EGFR	High Throughput	
ORF7b	ERBB2	High Throughput	
ORF7b	IGF1R	High Throughput	
ORF7b	LYN	High Throughput	
ORF7b	PRKCD	High Throughput	
ORF7b	PRKCQ	High Throughput	
ORF7b	SRC	High Throughput	
ORF7b	YES1	High Throughput	
ORF7b	APP	High Throughput	
ORF7b	LYN	High Throughput	
ORF7b	PIK3R1	High Throughput	
ORF7b	PRKCD	High Throughput	
ORF7b	PTPN1	High Throughput	
ORF7b	PTPN2	High Throughput	
ORF7b	RAC1	High Throughput	
ORF7b	SRC	High Throughput	
ORF7b	YES1	High Throughput	
ORF7b	NDUFB11	High Throughput	
ORF7b	NDUFA10	High Throughput	
ORF7b	NDUFA12	High Throughput	
ORF7b	NDUFS3	High Throughput	
ORF7b	NDUFS7	High Throughput	
ORF7b	NDUFV1	High Throughput	
ORF7b	NDUFV1	High Throughput	
ORF7b	CES1	High Throughput	
ORF7b	MAOA	High Throughput	
ORF8	IGF1R	High Throughput	
ORF8	APP	High Throughput	
ORF8	PTPN1	High Throughput	
ORF8	SRC	High Throughput	

ORF8	PTPN1	High Throughput	
ORF8	PTPN2	High Throughput	
ORF8	RAC1	High Throughput	
ORF8	DNMT1	High Throughput	
ORF8	DNMT1	High Throughput	
ORF8	CES1	High Throughput	
ORF9b	DNM1	High Throughput	
ORF9b	PTPN1	High Throughput	
ORF9b	PTPN1	High Throughput	
ORF9b	NDUFAF4	High Throughput	
ORF9b	NDUFA6	High Throughput	
ORF9b	NDUFA9	High Throughput	
ORF9b	NDUFA10	High Throughput	
ORF9b	NDUFB9	High Throughput	
ORF9b	NDUFS1	High Throughput	
ORF9b	NDUFS2	High Throughput	
ORF9b	NDUFS3	High Throughput	
ORF9b	NDUFV2	High Throughput	
ORF9b	NDUFV3	High Throughput	
ORF9b	NDUFAF4	High Throughput	
ORF9c	NDUFAF1	High Throughput	
ORF9c	NDUFB9	High Throughput	
ORF9c	NDUFAF1	High Throughput	
ORF9c	NDUFB9	High Throughput	
S	EGFR	High Throughput	
S	ERBB2	High Throughput	
S	PTPN1	High Throughput	
S	RAC1	High Throughput	
S	SRC	High Throughput	
S	YES1	High Throughput	
S	EGFR	High Throughput	
S	ERBB2	High Throughput	
S	LYN	High Throughput	
S	PIK3CB	High Throughput	
S	PRKCD	High Throughput	
S	PRKCQ	High Throughput	
S	SRC	High Throughput	
S	YES1	High Throughput	
S	PTPN1	High Throughput	
S	RAC1	High Throughput	
S	NDUFAF4	High Throughput	
S	NDUFS2	High Throughput	

Table S6: Docking scores in (Kcal/mol) for SARS-CoV-2 Mpro and RBD and human ACE2 with ginger compounds			
**Compound**	**Mpro (6Y84)**	**RBD (6M0JE)**	**ACE2 (1R4L)**
(6)-Gingerdiacetate	-7.2328	-6.2191	-8.1921
(10)-Gingerol	-6.7918	-6.9106	-8.114
(8)-Gingerol	-6.7389	-6.5476	-7.8647
(6)-shogaol	-6.1582	-5.8945	-7.5118
(8)-Shogaol	-6.584	-5.9758	-7.2153
(6)-Gingerol	-6.2926	-6.2803	-6.7709
Zingiberenol	-5.8462	-5.1164	-6.1243
Nerolidol	-5.792	-5.3034	-6.4142
α-Farnesene	-5.7554	-5.5307	-6.1044
ar-Curcumene	-5.5962	-5.2284	-5.9591
β-Bisabolene	-5.5082	-5.2359	-6.1165
α-Zingiberene	-5.4864	-5.2347	-5.9894
β-Zingiberene	-5.4501	-5.1675	-6.0783
β-Sesquiphellandrene	-5.4453	-5.1663	-6.0733
β-Elemene	-5.307	-4.798	-5.7672
γ-Selinene	-5.1582	-4.9339	-5.2838
Zerumbone	-5.103	-4.5892	-5.4953
Neral	-4.9867	-4.7886	-5.4816
Bornyl acetate	-4.8517	-4.7988	-5.1337
Linalool	-4.8225	-4.708	-5.295
Nonanal	-4.7958	-4.5891	-5.7295
1-Nonanol	-4.7569	-4.6324	-5.8145
Geranial	-4.6718	-4.8028	-5.4556
Myrcene	-4.6488	-4.5261	-5.5324
p-Cymene	-4.6306	-4.2256	-4.7942
Limonene	-4.5928	-4.2711	-4.8667
α-Terpineol	-4.4171	-4.3611	-4.9214
1,8-Cineole	-4.3645	-4.5889	-4.5029
β-Phellandrene	-4.3221	-4.2105	-4.8254
Borneol	-4.3149	-4.2858	-4.4798
Camphene	-4.2797	-4.2815	-4.8244
Isoborneol	-4.2258	-4.2627	-4.5493

α-Pinene	-4.1992	-4.1915	-4.7444
β-Pinene	-4.1944	-4.3972	-4.6227
